# Iridium(iii)-bis(imidazolinyl)phenyl catalysts for enantioselective C–H functionalization with ethyl diazoacetate†
†Electronic supplementary information (ESI) available: Experimental procedures and computational details are provided. CCDC 1408224. For ESI and crystallographic data in CIF or other electronic format see DOI: 10.1039/c6sc00190d


**DOI:** 10.1039/c6sc00190d

**Published:** 2016-02-05

**Authors:** N. Mace Weldy, A. G. Schafer, C. P. Owens, C. J. Herting, A. Varela-Alvarez, S. Chen, Z. Niemeyer, D. G. Musaev, M. S. Sigman, H. M. L. Davies, S. B. Blakey

**Affiliations:** a Department of Chemistry , Emory University , 1515 Dickey Drive , Atlanta , Georgia 30322 , USA . Email: sblakey@emory.edu; b Emerson Center for Scientific Computation , Emory University , 1515 Dickey Drive , Atlanta , Georgia 30322 , USA; c Department of Chemistry , University of Utah , 315 South 1400 East , Salt Lake City , Utah 84112 , USA

## Abstract

The intermolecular enantioselective C–H functionalization with acceptor-only metallocarbenes is reported using a new family of Ir(iii)-bis(imidazolinyl)phenyl catalysts, developed based on the interplay of experimental and computational insights. The reaction is tolerant of a variety of diazoacetate precursors and is found to be heavily influenced by the steric and electronic properties of the substrate. Phthalan and dihydrofuran derivatives are functionalized in good yields and excellent enantioselectivities.

## Abstract 2

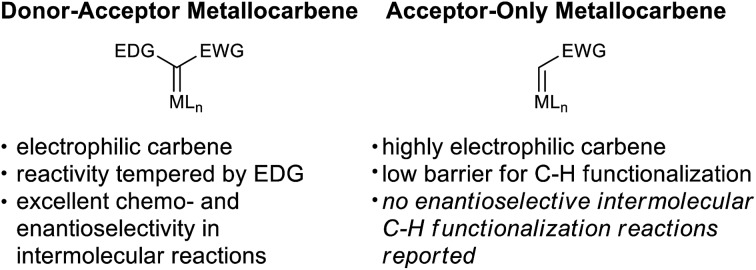
The intermolecular enantioselective C–H functionalization with acceptor-only metallocarbenes is reported using a new family of Ir(iii)-bis(imidazolinyl)phenyl catalysts.

## Introduction

**Fig. 1:**
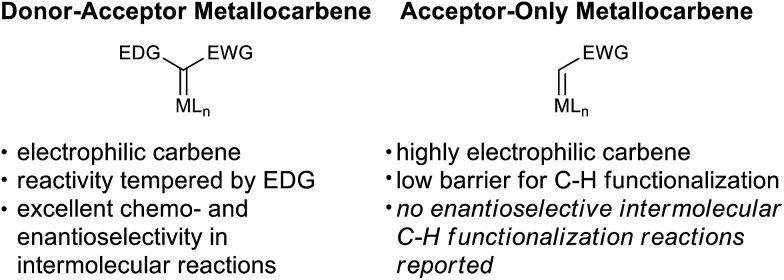
Comparison of metallocarbene classes.

C–H functionalization methods continue to provide elegant solutions to synthetic challenges of natural products and pharmaceuticals.^1^ In recent years, insertion of metallocarbenes has become particularly powerful.^2^ The discovery of the donor–acceptor class of carbenes and the development of new dirhodium tetracarboxylate catalysts revolutionized intermolecular C–H insertion reactions.^3^ The donor group tempers the electrophilicity of the metallocarbene and allows for exquisite regio- and enantioselectivity in these complex reactions (Fig. 1).

In contrast, there are no reports of enantioselective intermolecular C–H functionalization reactions utilizing acceptor-only carbene precursors even though these reagents are readily available. Although many catalysts have been developed to promote the mechanistically related enantioselective alkene cyclopropanation using ethyl diazoacetate,^4^ C–H functionalization with this reagent remains limited to a racemic manifold.^5^

The challenge in developing catalysts for acceptor-only carbene insertion reactions is two-fold. First, these metallocarbenes are highly electrophilic and reactive. The barrier for the Rh_2_(OAc)_4_ catalyzed C–H insertion reaction of methyl diazoacetate into the relatively strong C–H bonds of cyclopentane has been calculated to be only 3.5 kcal mol^–1^.^6^ This low barrier to carbene transfer presents an inherent chemoselectivity challenge. Second, the acceptor-only carbene itself is not prochiral, and for enantioselective C–H insertion reactions, the catalyst must provide an environment that differentiates among orientations of the incoming substrate.

Herein, we report the development of an iridium catalyst that promotes enantioselective intermolecular C–H insertion reactions using the prototypical acceptor-only carbene precursor ethyl diazoacetate. Our catalyst design sought to take advantage of the ability of third row transition metals to participate in increased back-bonding to suppress the metallocarbene electrophilicity while also increasing barriers to subsequent group transfer process required for enantioselective catalysis.

## Results and discussion

The broad utility of group 9 metal complexes as catalysts for group transfer chemistry led us to adopt iridium as the metal of choice for this study.^7^ We identified the bis(oxazolinyl)phenyl (phebox) ligand system extensively studied by Nishiyama and coworkers as a readily accessible, modular platform for catalyst development.^8^ In our initial experiments, we observed Ir(iii)-phebox **1** is indeed capable of catalyzing the C–H insertion reaction of ethyl diazoacetate into tetrahydrofuran with good yield (80%) and promising enantioselectivity (eqn (1), 75 : 25 er).

Additionally, we demonstrated that it is not necessary to utilize tetrahydrofuran as solvent, and when it is used as a substrate in PhCF_3_, the enantioselectivity increases (eqn (2), 81 : 19 er). Diethyl maleate is observed as the major byproduct in this reaction.
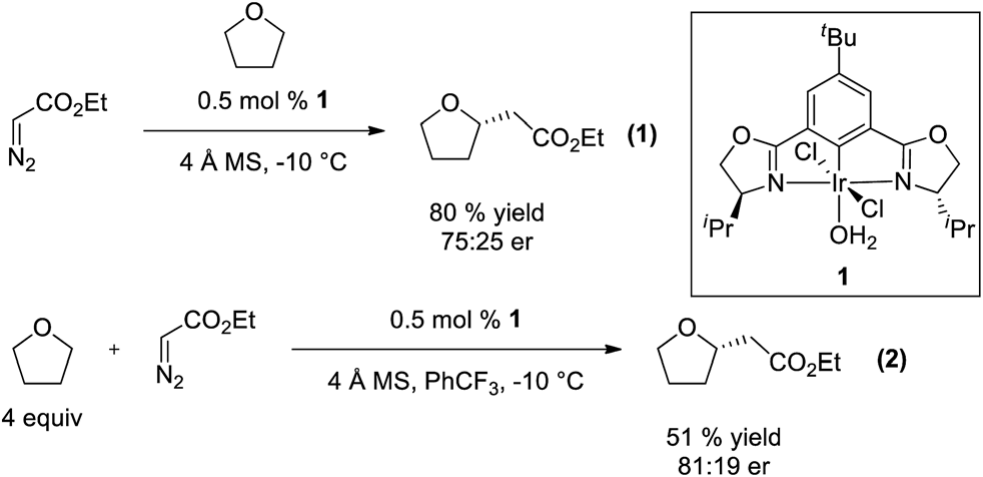


**Fig. 2:**
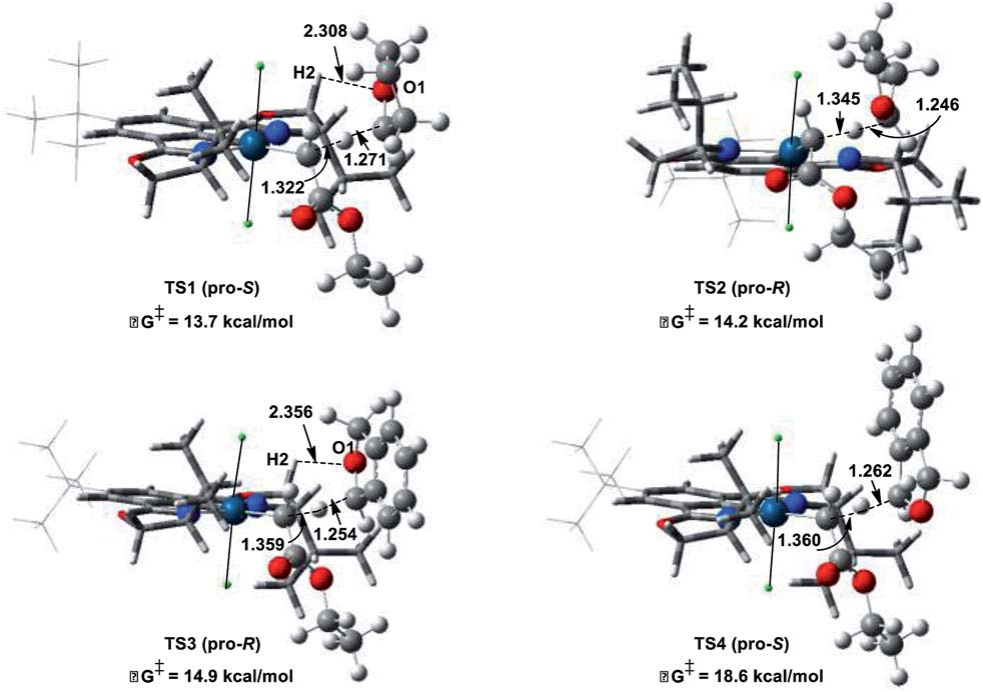
Calculated pro-*R* and pro-*S* transition states of ethyl diazoacetate insertion into tetrahydrofuran (top) and phthalan (bottom) with Ir(iii)-phebox **1**.

To gain insight and direct catalyst evolution, the structure and free energy of the pro-*S* and pro-*R* transition states for Ir(iii)-phebox **1** catalyzed ethyl diazoacetate insertion into tetrahydrofuran were calculated using the CPCM-M06L/{LANL08(f)_Ir_ + [6-31G(d,p)]} level of theory (Fig. 2, top). A small ΔΔ*G*^‡^ value of 0.5 kcal mol^–1^ was calculated between the pro-*S* and pro-*R* transition states **TS1** and **TS2**. Calculations also indicated that the favored transition state **TS1** involves a short O1–H2 distance of 2.308 Å due to an electrostatic interaction between the ligand and substrate. When tetrahydrofuran was replaced with phthalan as the substrate in the calculations, the ΔΔ*G*^‡^ between **TS3** and **TS4** was predicted to increase to 3.7 kcal mol^–1^.

**Scheme 1:**
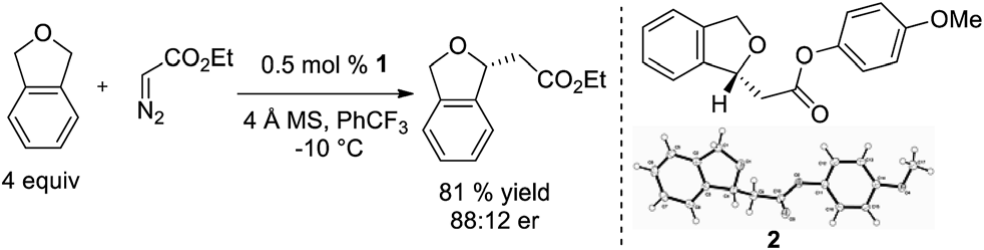
Ir(iii)-phebox **1** catalyzed enantioselective insertion of ethyl diazoacetate into phthalan.

Based on this model, we exposed phthalan to reaction conditions with ethyl diazoacetate, and an increased yield of 81% and er of 88 : 12 were observed (Scheme 1). Additionally, subsequent crystallization of the 4-MeO-Ph ester derivative **2** established that the computational model correctly predicted the major enantiomer formed in this reaction.

Encouraged by the qualitative accuracy of the computational predictions, we utilized this model for further catalyst optimization. Due to the calculated electrostatic interaction between H2 of the ligand and O1 of the substrate in favored transition states **TS1** and **TS3**, we hypothesized that modulating the charge density on H2 could provide further stabilization of the favored transition state and improve enantioselectivity.

**Scheme 2:**
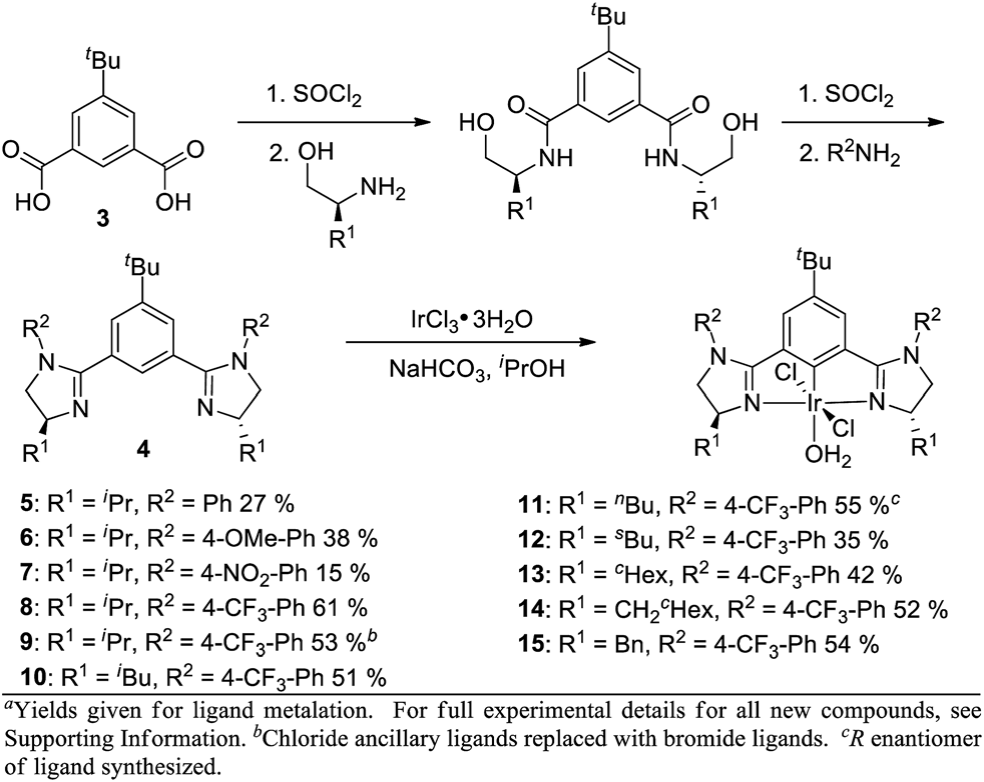
Ir(iii)-bis(imidazolinyl)phenyl (phebim) complex synthesis^a^.

In order to influence the charge density on H2, we shifted our attention to the bis(imidazolinyl)phenyl (phebim) ligand system (**4**) in an effort to modulate the electronics of the imidazoline through an aryl ring at the R^2^ position (Scheme 2).^9^ The ligand can be prepared in two steps from commercially available amino alcohols and 5-*tert*-butylisophthalic acid (**3**).9*b* Metalation under standard conditions with IrCl_3_·3H_2_O afforded Ir(iii)-phebim complexes **5–15**.8d,f

R^2^-Ph-substituted Ir(iii)-phebim **5** was found to catalyze ethyl diazoacetate insertion into phthalan in similar yield and er to phebox **1** (Table 1, entry 1; Scheme 1). Electronic variation at the 4-position of the phenyl ring at R^2^ had a small but measurable impact on enantioselectivity, with both 4-NO_2_-Ph-substituted catalyst **7** and 4-CF_3_-Ph-substituted catalyst **8** giving the best er of 90 : 10 (entries 2–4). This observation is consistent with the electrostatic control hypothesis suggested by the computational model. Due to the lower yield of the metalation step for **7** (15%), 4-CF_3_-Ph was adopted as the optimal substituent at R^2^ (61%). Complex **9** with Br ancilliary ligands improved the yield by 10%, but decreased enantioselectivity (entry 5). The greatest impact on enantioselectivity was achieved by modulating ligand substituent R^1^, with ^i^Bu-substituted catalyst **10** and CH_2_-^c^Hex-substituted catalyst **14** each improving the er to 95 : 5 (entries 6 and 10). Though equivalent in enantioselectivity, the use of catalyst **10** gave an enhanced yield relative to **14**.

**Table 1:** Ir(iii)-phebim optimization for C–H insertion of ethyl diazoacetate into phthalan^*a*^

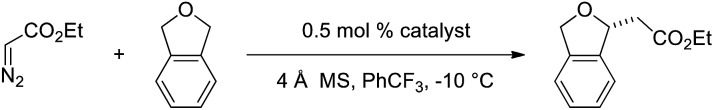			
Entry	Catalyst	Yield^*b*^ (%)	er^*c*^
1	**5**	85	88 : 12
2	**6**	26	89 : 11
3	**7**	70	90 : 10
4	**8**	73	90 : 10
5	**9**	83	88 : 12
6	**10**	95	95 : 5
7	**11**	54	13 : 87^*d*^
8	**12**	81	92 : 8
9	**13**	83	90 : 10
10	**14**	80	95 : 5
11	**15**	36	86 : 14

^*a*^General conditions: 0.29 M solution of ethyl diazoacetate in PhCF_3_ was added over 5 h to a mixture of catalyst, phthalan (4 equiv.), and 4 Å MS in PhCF_3_ at –10 °C.

^*b*^Isolated yields.

^*c*^Determined by chiral HPLC.

^*d*^Catalyst **11** is a member of the opposite enantio-series.

**Fig. 3:**
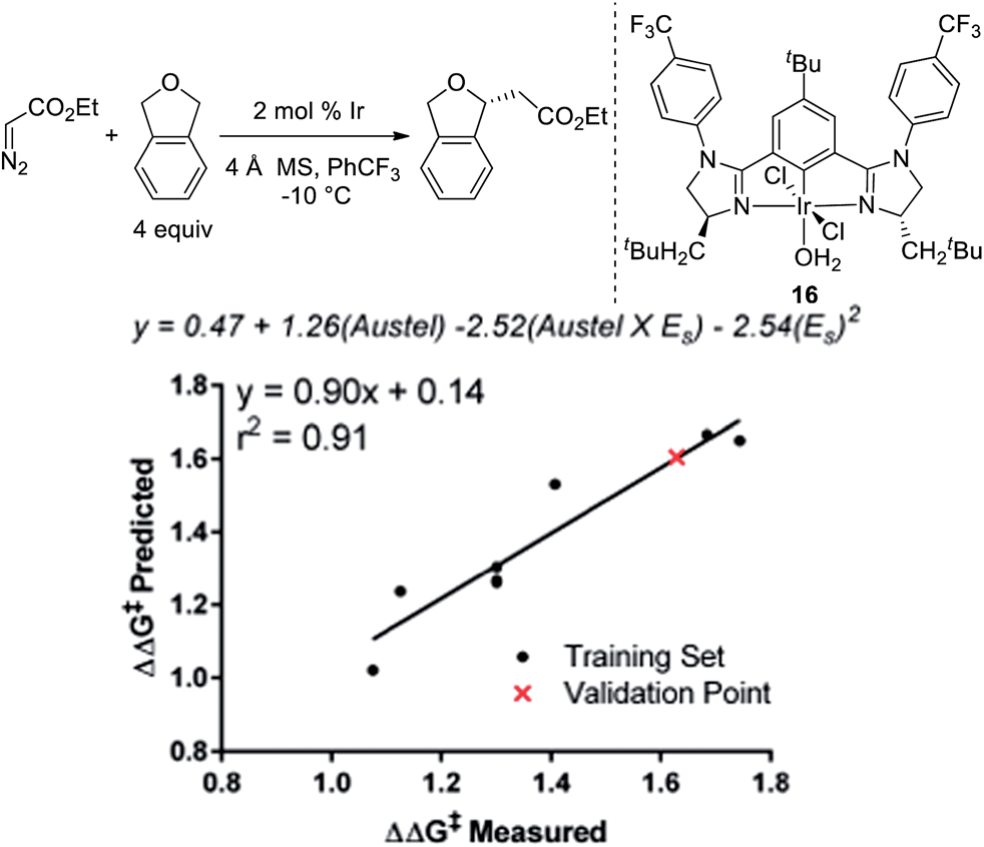
Linear regression modeling of Ir(iii)-phebim R^1^-substitution.

To further explore if catalysts **10** and **14** were optimal in terms of enantioselectivity for the insertion of ethyl diazoacetate into phthalan, we utilized linear regression mathematical modeling techniques. This was accomplished by collecting steric parameters describing the R^1^ substituent, including Sterimol, Charton/Taft, and Austel values.^10^ While the use of Sterimol and Charton values has been explored for analyzing enantioselective reactions,^11^ the Austel value has not been used previously. Simply stated, this value is a measure of branching in alkyl chains.^12^ By applying multivariate regression using this parameter set and the observed enantioselectivity, a predictive model was found as depicted in Fig. 3.

Of particular interest is the conflicting steric terms required to effectively model the reaction. As R^1^ increases in size, the equation suggests that a maximum can be reached balancing the level of branching as determined by the Austel value and the relative spherical size as determined by the Charton/Taft value (*E*_S_). Based on this model, a third comparable catalyst with CH_2_-^*t*^Bu-substitution (**16**) was predicted to give ethyl diazoacetate insertion into phthalan with a modest reduction in er (94 : 6). This result was confirmed experimentally with catalyst **16** resulting in a yield of 92% and an er of 94 : 6. The modeling and subsequent experiment suggests that the unusual CH_2_^c^Hex substituent on the imidazoline ring of the catalyst is indeed optimal for the reaction.

Seeking further improvements in selectivity, and based on the strong influence of the nature of the ester in donor–acceptor metallocarbene C–H insertion, a range of ester substituents were examined for acceptor-only metallocarbene C–H insertion into phthalan with the most selective Ir(iii)-phebim catalysts **10** and **14**.2b,^13^ In our system, using methyl diazoacetate resulted in a modest drop in enantioselectivity to 94 : 6 er with catalyst **14** (Table 2, entry 2). The electronically unique reagent, 2,2,2-trichloroethyl diazoacetate, was found to give the same er as ethyl diazoacetate with **14** (95 : 5 er, entry 4). Similarly, 2-trimethylsilyl diazoacetate gave C–H insertion in good yield and an er of 94 : 6 (entry 6). Phenyl diazoacetate gave an enantioselectivity of 93 : 7 (entry 8), and 4-MeO and 4-CF_3_ substituted phenyl diazoacetates were tolerated as well, with no significant improvements in yield or er (entries 9–12). In all cases, CH_2_-^c^Hex-substituted Ir(iii)-phebim **14** provided slightly higher enantioselectivity than ^i^Bu-substituted Ir(iii)-phebim **10**, though often coupled with a slightly diminished yield. The consistently higher enantioselectivity led us to establish **14** as the optimal catalyst to further investigate the substrate scope of this methodology.

**Table 2:** Diazoacetate scope for Ir(iii)-phebim catalyzed enantioselective C–H insertion into phthalan^*a*^

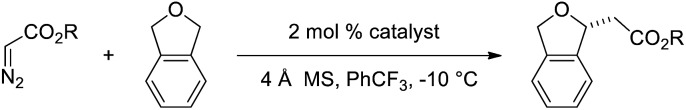				
Entry	R	Catalyst	Yield^*b*^ (%)	er^*c*^
1^*d*^	Me	**10**	72	92 : 8
2^*d*^	Me	**14**	69	94 : 6
3	CH_2_CCl_3_	**10**	84	94 : 6
4	CH_2_CCl_3_	**14**	71	95 : 5
5	CH_2_CH_2_Si(CH_3_)_3_	**10**	62	93 : 7
6	CH_2_CH_2_Si(CH_3_)_3_	**14**	67	94 : 6
7	Ph	**10**	65	91 : 9
8	Ph	**14**	68	93 : 7
9	4-OMe-Ph	**10**	57	91 : 9
10	4-OMe-Ph	**14**	70	92 : 8
11	4-CF_3_-Ph	**10**	78	90 : 10
12	4-CF_3_-Ph	**14**	69	92 : 8

^*a*^General conditions: 0.29 M solution of ethyl diazoacetate in PhCF_3_ was added over 5 h to a mixture of catalyst, phthalan (4 equiv.), and 4 Å MS in PhCF_3_ at –10 °C.

^*b*^Isolated yields.

^*c*^Determined by chiral HPLC.

^*d*^0.5 mol% catalyst loading.

With respect to substrate scope, we subjected a variety of phthalan derivatives to standard C–H insertion conditions with catalyst **14** and ethyl diazoacetate and observed strong electronic and steric influences on reaction efficiency and regioselectivity (Table 3).

**Table 3:** Substrate scope for Ir(iii)-phebim 14 catalyzed enantioselective C–H insertion with ethyl diazoacetate^*a*^ ^,^^*b*^

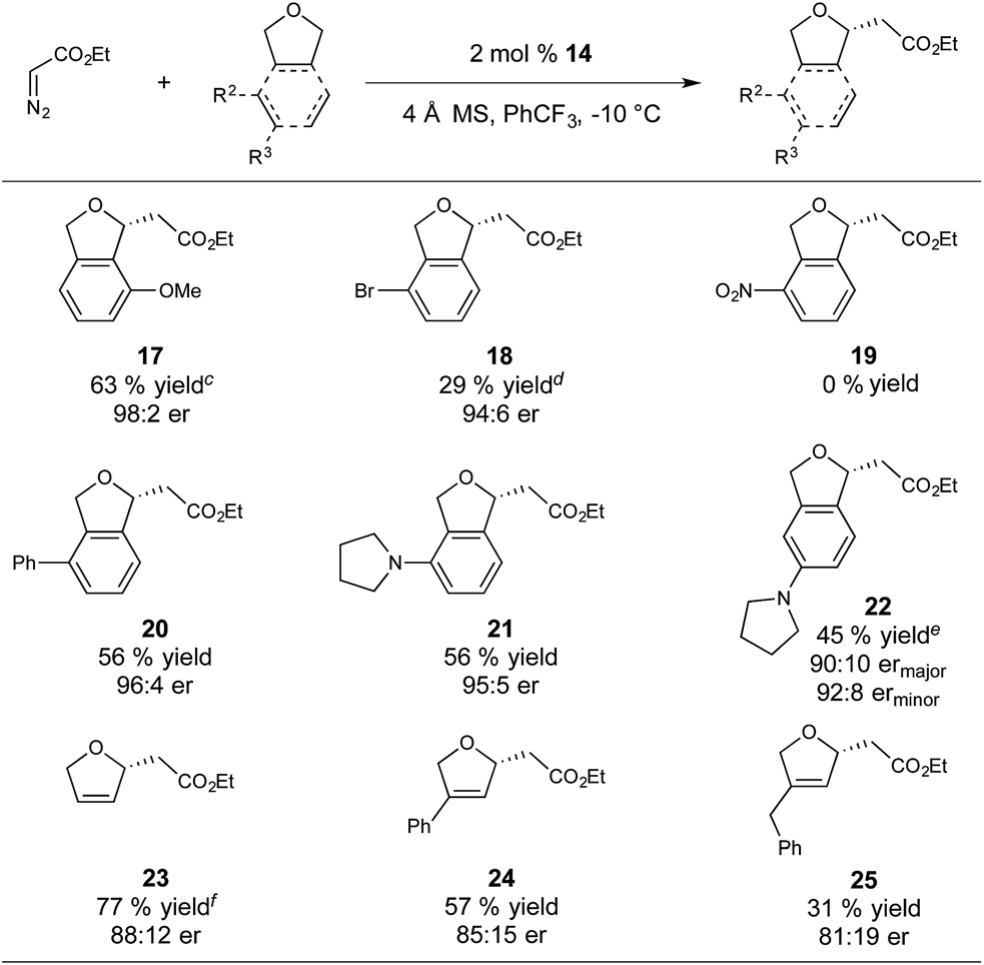

^*a*^General conditions: 0.29 M solution of ethyl diazoacetate in PhCF_3_ was added over 5 h to a mixture of catalyst, substrate (4 equiv.), and 4 Å MS in PhCF_3_ at –10 °C.

^*b*^Isolated yields are based on EDA.

^*c*^The 2,6-regioisomer is also observed in an additional 19% yield and 94 : 6 er.

^*d*^The 2,6-regioisomer is also observed in an additional 10% yield.

^*e*^Product was isolated as a 4 : 1 mixture of the 2,5 (major) and 2,4 (minor) regioisomers.

^*f*^0.5 mol% catalyst loading.

In introducing a methoxy substituent at the 3-position of phthalan, the catalyst showed selectivity for insertion at the electronically activated position to give 2,3-substituted phthalan **17** (63% yield), with an excellent er of 98 : 2 (Table 3). The competing 2,6-substituted regioisomer (sterically favored) was formed in only 19% yield and 94 : 6 er. The weakly electron withdrawing 3-Br substituent deactivated the substrate towards C–H insertion and delivered 2,6-substituted regioisomer **18** as the major product (39% yield, 3 : 1 regioselectivity) with excellent enantioselectivity (94 : 6). In this case steric factors override the weak electronic bias for insertion adjacent to the bromine substituent. Strongly electron withdrawing NO_2_-substitution at the 3-position resulted in no product formation. Using 3-Ph-substituted phthalan as a substrate afforded good enantioselectivity of 96 : 4 and yielded the sterically favored 2,6-isomer **20** as the sole product in 56% yield. 3-Pyrrolidinyl-phthalan also showed complete selectivity for the sterically favored 2,6-isomer **21**, with the significant steric influence of pyrrolidinyl-substitution at the 3-position overriding the electron-donating effect. Moving the pyrrolidinyl substituent to the 4-position (sterically neutral with respect to potential insertion sites) gave a majority of the electronically favored 2,5-isomer **22**, with the 2,4-isomer observed as a minor component of the reaction mixture (45% yield, 4 : 1 regioselectivity, 90 : 10 er_major_, 92 : 8 er_minor_). Importantly, the Ir(iii)-phebim catalyst **14** is active in the presence of these basic amines with no signs of catalyst inhibition or reaction of the metallocarbene with the amine. This observation is particularly significant for potential applications in pharmaceutically relevant molecules.

Insertion into 2,5-dihydrofuran gave **23** in 77% yield and an er of 88 : 12, with no competing cyclopropanation observed. Consistent with our observations for phthalan-derived substrates, regioselectivity of the insertion is strongly influenced by steric factors. Using 3-Ph-substituted 2,5-dihydrofuran produced **24** in 57% yield and 85 : 15 er. A greater reduction of yield to 31% was observed when using 3-Bn-substituted 2,5-dihydrofuran to give **25** in 81 : 19 er.

**Scheme 3:**
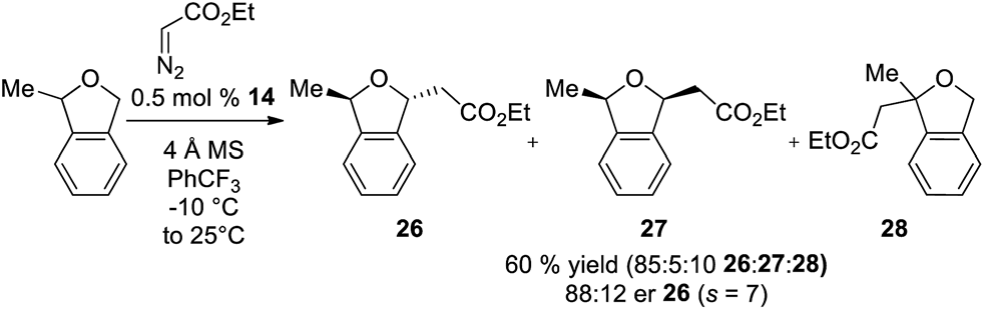
Ir(iii)-phebim 14 catalyzed kinetic resolution of *rac*-2-Me-phthalan with ethyl diazoacetate.

In addition to enantioselective insertion, kinetic resolution of *rac*-2-Me-phthalan resulted in formation of the *anti*-2,7-substituted product **26** primarily, which is consistent with what one would expect based on the computed transition states (Scheme 3 and Fig. 2). Minor amounts of the *syn*-isomer **27** and 3° C–H insertion product **28** were also formed. The major product **26** was formed in an er of 88 : 12.

## Conclusions

In summary, using a combination of computational study and catalyst design based on fundamental organometallic principles, we have developed an iridium complex capable of mediating highly selective intermolecular functionalization of ethereal C–H bonds with the prototypical acceptor-only carbene precursor ethyl diazoacetate. A variety of phthalan and dihydrofuran derivatives were competent substrates, exhibiting a strong steric and electronic influence on reaction yield and regioselectivity. These results provide a foundation for further expansion in this area, such as C–H insertion with novel, unexplored classes of acceptor-only carbene precursors.

## Supplementary Material

Supplementary information

Crystal structure data
