# Supplementary material for: Iridium(iii)-bis(imidazolinyl)phenyl catalysts for enantioselective C–H functionalization with ethyl diazoacetate
Source: Chem Sci. 2016 Feb 5;7(5):3142–6. doi: 10.1039/c6sc00190d (PMC6005272; doi:10.1039/c6sc00190d)

Electronic Supplementary Material (ESI) for Chemical Science.
This journal is © The Royal Society of Chemistry 2016

Iridium(III)-bis(imidazolinyl)phenyl Catalysts for Enantioselective C–H Functionalization with Ethyl Diazoacetate

Nina Mace Weldy,[†] Andrew G. Schafer,[†] Clayton P. Owens,[†] Cameron J. Herting,[†] Adrian Varela-Alvarez,[‡] Shentan Chen,[‡] Zach Niemeyer,[§] Djamaladdin G. Musaev,*[,‡] Matthew S. Sigman,*[,§] Huw M. L. Davies,*[,†] and Simon B. Blakey*[,†]

[†]Department of Chemistry, Emory University, 1515 Dickey Drive, Atlanta, Georgia 30322, United States
[‡]Emerson Center for Scientific Computation, Emory University, 1515 Dickey Drive, Atlanta, Georgia 30322, United States
[§]Department of Chemistry, University of Utah, 315 South 1400 East, Salt Lake City, Utah 84112, United States

Table of Contents

I. General Considerations 1
II. Procedures and Characterization Data
   a) Catalyst Synthesis 2
   b) Diazoacetate Synthesis 18
   c) Substrate Synthesis 21
   d) Preliminary Results Described in Equation 1 and Scheme 1 27
   e) Catalyst Optimization 28
   f) Acceptor-Only Diazoacetate Scope 28
   g) Substrate Scope 32
III. Kinetic Isotope Effect
   a) Kinetic Isotope Effect by ^{1}H NMR 37
   b) Kinetic Isotope Effect by Mass Spectrometry 41
IV. X-Ray Crystallographic Information 42
V. Computational Details 44
VI. Linear Regression Mathematical Modeling Details 52
VII. References 54
VIII. HPLC Data 56
IX. NMR Spectra 95

I. General Considerations

^{1}H and ^{13}C NMR spectra were recorded on a Varian Inova 600 spectrometer (600 MHz ^{1}H, 150 MHz ^{13}C) and a Varian Inova 400 spectrometer (400 MHz ^{1}H, 100 MHz ^{13}C) at room temperature in $CDCl_3$ (neutralized and dried with anhydrous K_2CO_3) with internal $CHCl_3$ as the reference (7.27 ppm for ^{1}H and 77.23 ppm for ^{13}C) or $(CD_3)_2SO$ with internal $(CH_3)_2SO$ as the reference (2.50 ppm for ^{1}H and 39.51 ppm for ^{13}C). Chemical shifts (δ values) were reported in parts per million (ppm) and coupling constants (*J* values) in Hz. Multiplicity is indicated using the following abbreviations: s = singlet, d = doublet, t = triplet, q = quartet, qn = quintet, sext = sextet, m = multiplet, br = broad signal.). Infrared (IR) spectra were recorded using Thermo Electron Corporation Nicolet 380 FT-IR spectrometer. High-resolution mass spectra were obtained using a Thermo Electron Corporation Finigan LTQFTMS (at the Mass Spectrometry Facility, Emory University). X-ray diffraction studies were carried out in the X-ray Crystallography Laboratory at Emory University on a Bruker Smart 1000 CCD diffractometer. Gas chromatography (GC) was carried out on an Agilent 6850 Network GC System equipped with a CHIRASIL-DEX CB column. High performance liquid chromatography (HPLC) was carried out on an Agilent 1100 Series equipped with Chirasil Chiralpak OJ-H and OD-H columns and a variable wavelength detector. We acknowledge the use of shared instrumentation provided by grants from the NIH and the NSF. Analytical thin layer chromatography (TLC) was performed on precoated glass backed EMD 0.25 mm silica gel 60 plates. Visualization was accomplished with UV light or ethanolic anisaldehyde, followed by heating. Flash column chromatography was carried out using Silicycle® silica gel 60 (40-63 μm).

All reactions were conducted with anhydrous solvents in oven-dried and nitrogen-charged glassware. Anhydrous solvents were purified by passage through activated alumina using a *Glass Contours* solvent purification system unless otherwise noted. $PhCF_3$ was dried over activated 4 Å molecular sieves and deoxygenated by bubbling through N_2. Solvents for workup, extraction and column chromatography were used as received from commercial suppliers. All reagents used were purchased from commercial vendors and used as received unless otherwise noted. Phthalan was obtained from Alfa Aesar or Sigma Aldrich and distilled before use. Ethyl diazoacetate (EDA) was obtained from Sigma Aldrich containing 14 % (w/w %) dichloromethane and used without further purification. 4 Å powdered molecular sieves were activated by heating to 100 ° C under reduced pressure (0.2 torr) for 12 hours. Pyridine and Et_3N were purified by distillation from calcium hydride. $IrCl_3 \cdot 3H_2O$ and $IrBr_3 \cdot 4H_2O$ were purchased from Strem. Amino alcohols were purchased from Sigma Aldrich or Combi-Blocks. Full crystallographic data (CIF) is available as a separate file.

II. Procedures and Characterization Data

a) Catalyst Synthesis

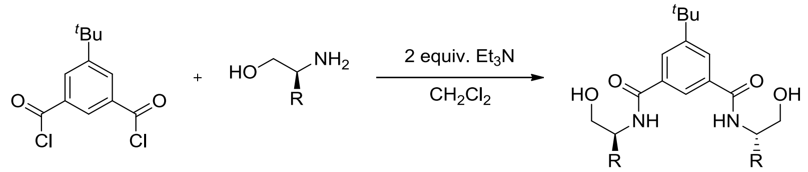

General Procedure A

A procedure was adapted from the literature as follows:[1] A 0.5 M solution of 5-*tert*-butylisophthaloyl chloride (1.0 equiv.) in CH_2Cl_2 was added dropwise to a 0 °C mixture of amino alcohol (2.0 equiv.) and Et_3N (2.0 equiv.) in CH_2Cl_2 (0.29 M). The reaction mixture was warmed to ambient temperature and stirred approximately 20 h. Water was added (5 mL/1 mmol acyl chloride), and the mixture was stirred for 10 minutes. The phases were separated and the organic phase was dried over Na_2SO_4 and concentrated *in vacuo*. The resulting solid was purified as indicated to give the title amide.

General Procedure B

A procedure was adapted from the literature as follows:[2] A round bottom flask was charged with amino alcohol or amino alcohol·TFA salt (2.0 equiv) in isopropyl acetate (0.13 M in acyl chloride). The mixture was heated to 65 °C with vigorous stirring, and aqueous 1.5 M $KHCO_3$ (10 equiv.) was added. 5-*tert*-Butyl isophthaloyl dichloride (1.0 equiv) was added as a solid in three equal portions over the course of 30 minutes, and the mixture was aged at 65 °C for 12 h. The mixture was cooled to room temperature and poured into a separatory funnel. The phases were separated, and the aqueous phase was extracted with isopropyl acetate (3 x). The organics were combined, dried over sodium sulfate, filtered, and concentrated *in vacuo*. The resulting solid was purified as indicated to give the title amide.

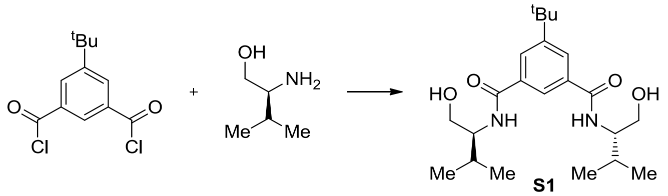

5-(*tert*-butyl)-*N*1,*N*3-bis((*S*)-1-hydroxy-3-methylbutan-2-yl)isophthalamide (S1). Prepared by general procedure **A** using 5-*tert*-butylisophthaloyl chloride (518 mg, 2.0 mmol) in CH_2Cl_2 (4 mL), L-valinol (413 mg, 4.0 mmol), and Et_3N (0.55 mL, 4.0 mmol) in CH_2Cl_2 (7 mL). Recrystallization from boiling Et_2O/CH_2Cl_2 afforded the title amide as a white flaky solid (582 mg, 74 %); **mp** 132-134 ° C; **IR** (thin film, cm^{-1}) 3303, 2963, 1636, 1537, 1214, 749; **^{1}H NMR** (400 MHz, $CDCl_3$) δ 7.84 (s, 1H), 7.72 (s, 2H), 7.18 (d, J = 9.8 Hz, 2H), 3.93 (dd, J = 9.2, 5.2 Hz, 2H), 3.84 (dd, J = 11.6, 3.1 Hz, 2H), 3.75 (dd, J = 7.3, 11.3 Hz, 2H), 1.94 (dq, J = 13.7, 6.8 Hz, 2H), 1.19 (s, 9H), 0.96 (t, J = 6.6 Hz, 12H); **^{13}C NMR** (100 MHz, $CDCl_3$) δ 168.9, 152.1, 134.8, 127.5, 63.3, 58.0, 35.0, 31.3, 29.7, 19.7, 19.5; **HRMS** [+APCI] calculated for $C_{22}H_{37}N_2O_4$ 393.2748, found 393.2743 $[M+H]^+$; **$[\alpha]^{20}_D$** -47.5 ° (c 1.0, $CHCl_3$).

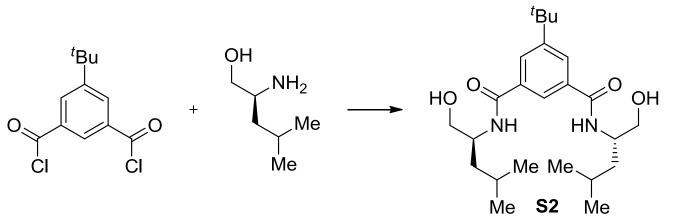

S2

5-(*tert*-butyl)-*N*1,*N*3-bis((*S*)-1-hydroxy-4-methylpentan-2-yl)isophthalamide (S2). Prepared by general procedure **A** using 5-*tert*-butylisophthaloyl chloride (1.04 g, 4.0 mmol) in CH_2Cl_2 (8 mL), (*S*)-leucinol (943 mg, 1.03 mL, 8.0 mmol), and Et_3N (1.16 mL, 8.0 mmol) in CH_2Cl_2 (14 mL). Recrystallization from boiling CH_2Cl_2 afforded the title amide as a white solid (1.36 g, 80 %); **mp** 161-164 ° C; **IR** (thin film, cm^{-1}) 3291, 2957, 2870, 1637, 1543; **^{1}H NMR** (400 MHz, $CDCl_3$) δ 7.85 (bs, 1H), 7.77 (bs, 2H), 6.93 (bs, 2H), 4.28 (bs, 2H), 3.82 (dd, *J* = 11.3, 3.2 Hz, 2H), 3.66 (dd, *J* = 11.0, 6.5 Hz, 2H), 3.08-3.05 (m, 2H), 1.67 (dq, *J* = 13.6, 6.7 Hz, 2H), 1.59-1.52 (m, 2H), 1.43-1.37 (m, 2H), 1.23 (s, 9H), 0.94 (d, *J* = 6.5 Hz, 12H); **^{13}C NMR** (100 MHz, $CDCl_3$) δ 168.6, 154.9, 134.6, 127.1, 121.6, 65.5, 50.6, 40.2, 30.9, 25.0, 22.9, 22.4; **HRMS** [+APCI] calculated for $C_{24}H_{41}N_2O_4$ 421.3061, found 421.3060 $[M+H]^+$; **$[\alpha]^{20}_D$** -43.7 ° (*c* 1.0, $CHCl_3$).

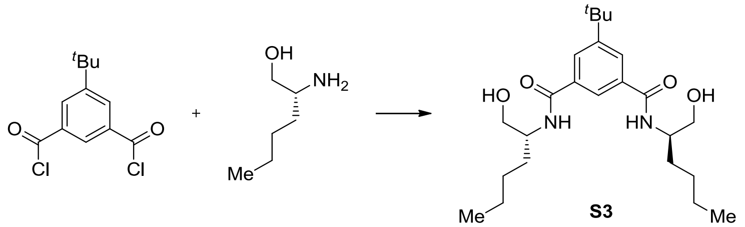

S3

5-(*tert*-butyl)-*N*1,*N*3-bis((*R*)-1-hydroxyhexan-2-yl)isophthalamide (S3). Prepared by general procedure **B** using 5-*tert*-butylisophthaloyl dichloride (504 mg, 2.0 mmol), (*R*)-2-amino-1-hexanol (509 mg, 4.3 mmol), $KHCO_3$ (13 mL, 19.4 mmol). Mixed solvent recrystallization (EtOAc/hexanes) afforded the title compound as a white amorphous solid (463 mg, 57 %); **IR** (thin film, cm^{-1}) 3294, 2957, 2871, 1633, 1537, 1460, 1260, 1063; **^{1}H NMR** (400 MHz, $CDCl_3$) δ 7.86-7.69 (m, 3H), 7.43 (d, *J* = 7.9 Hz, 2H), 4.78 (br s, 2H), 4.11 (br s, 2H), 3.78-3.76 (m, 2H), 3.70-3.62 (m, 2H), 1.56-1.49 (m, 4H) 1.30-1.23 (m, 10H), 1.14 (s, 9H) 0.84 (t, *J* = 6.9 Hz, 6H); **^{13}C NMR** (100 MHz, $CDCl_3$) δ 206.3, 168.8, 134.7, 127.5, 67.7, 64.9, 52.7, 35.0, 31.3, 28.6, 22.8, 14.2; **HMRS** (+APCI) calculated for $C_{24}H_{40}N_2O_4Na$ 443.2880, found 443.2880 $[M+Na]^+$. **$[\alpha]^{20}_D$** +68.4 ° (*c* 1.0, $CHCl_3$).

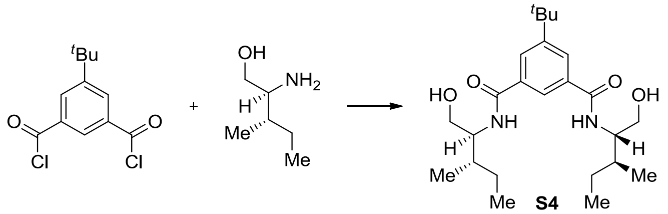

S4

5-(*tert*-butyl)-*N*1,*N*3-bis((2*S*,3*S*)-1-hydroxy-3-methylpentan-2-yl)isophthalamide (S4). Prepared by general procedure **A** using 5-*tert*-butylisophthaloyl chloride (518 mg, 2.0 mmol) in CH_2Cl_2 (4 mL), (*S*)-isoleucinol (472 mg, 4.0 mmol), and Et_3N (0.55 mL, 4.0 mmol) in CH_2Cl_2 (7 mL). Recrystallization from boiling Et_2O/CH_2Cl_2 furnished the title amide as a white flaky

solid (454 mg, 54 %); **mp** 94-97 ° C; **IR** (thin film, cm^{-1}) 3304, 2963, 2341, 2145, 1636, 1539, 1214, 751; **^{1}H NMR** (400 MHz, $CDCl_3$) δ 7.96 (s, 1H), 7.81 (d, *J* = 1.4 Hz, 2H), 7.79 (d, *J* = 1.5 Hz, 2H), 7.31 (d, *J* = 8.5 Hz, 2H), 4.04-3.97 (m, 2H), 3.84-3.74 (m, 4H), 3.06 (q, *J* = 7.3 Hz, 2H), 1.80-1.70 (m, 2H), 1.57-1.47 (m, 2H), 1.29 (t, *J* = 7.3 Hz, 2H), 1.2 (s, 9H), 0.93 (d, *J* = 6.7 Hz, 6H), 0.88 (t, *J* = 7.0 Hz, 6H); **^{13}C NMR** (100 MHz, $CDCl_3$) δ 168.7, 152.0, 134.7, 127.8, 122.0, 62.8, 56.7, 46.4, 36.1, 35.0, 31.3, 26.2, 15.6, 11.6, 8.8; **HRMS** [+APCI] calculated for $C_{24}H_{41}N_2O_4$ 421.3061, found 421.3060 $[M+H]^+$; $[\alpha]^{20}_D$ -62.0 ° (*c* 1.0, $CHCl_3$).

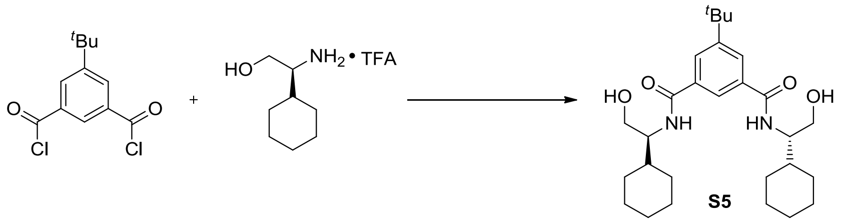

5-(*tert*-butyl)-*N*1,*N*3-bis((*S*)-1-cyclohexyl-2-hydroxyethyl)isophthalamide (S5). Prepared by general procedure **B** using (*S*)-2-amino-2-cyclohexylethanol·TFA salt (2.57 g, 10 mmol), in isopropyl acetate (40 mL, 0.13 M in acyl chloride), aqueous 1.5 M $KHCO_3$ (35 mL, 50 mmol, 10 equiv.), and 5-*tert*-Butyl isophthaloyl dichloride (1.3 g, 5.0 mmol). The resulting amide was obtained as a white solid (2.34 g, 99 %); **mp** 115-117 ° C; **IR** (thin film, cm^{-1}) 3289, 2927, 2853, 1706, 1640, 1535, 1157, 916; **^{1}H NMR** (600 MHz, $CDCl_3$) δ 7.85 (br s, 1H), 7.83 (br s, 2H), 6.69 (d, *J* = 6.7 Hz, 2H), 4.30 (br s, 2H), 3.82 (d, *J* = 10.0 Hz, 2H), 3.67-3.64 (m, 2H), 3.46 (dd, *J* = 6.0, 3.6 Hz, 2H), 3.18 (br s, 2H), 1.82 (d, *J* = 12.4 Hz, 2H), 1.73-1.62 (m, 8H), 1.28 (s, 9H), 1.21-112 (m, 6H), 1.00-0.87 (m, 4H); **^{13}C NMR** (100 MHz, $CDCl_3$) δ 168.4, 134.6, 127.2, 62.9, 61.7, 56.8, 56.4, 38.9, 38.1, 31.0, 29.2, 26.0, 25.8; **HRMS** [+NSI] calculated for $C_{28}H_{45}N_2O_4$ 473.3379, found 473.3374 $[M+H]^+$; $[\alpha]^{20}_D$ -28.5 ° (*c* 1.0, $CHCl_3$).

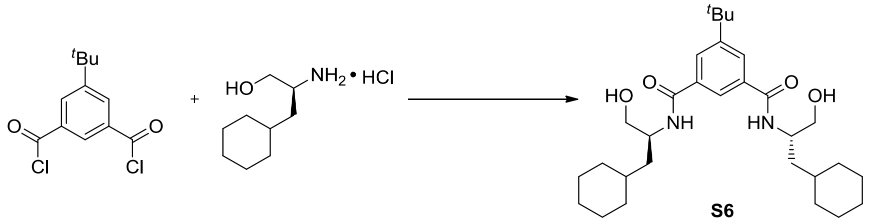

5-(*tert*-butyl)-*N*1,*N*3-bis((*S*)-1-cyclohexyl-3-hydroxypropan-2-yl)isophthalamide (S6). Prepared by general procedure **B** using (*S*)-2-amino-3-cyclohexylpropanol hydrochloride (1.08 g, 5.2 mmol, 2.0 equiv) in isopropyl acetate (20 mL, 0.13 M in acyl chloride), 1.5 M $KHCO_3$ (17 mL, 26 mmol, 10 equiv.), and 5-*tert*-Butyl isophthaloyl dichloride (671 mg, 2.6 mmol, 1.0 equiv). Diethyl ether (40 mL) was added to the crude solid with stirring, and the solid was collected by vacuum filtration to give the title amide as a white solid (1.25 g, 96 %); **mp** 88-90 ° C; **IR** (thin film, cm^{-1}) 3253, 2918, 2848, 1628, 1547, 1029, 902; **^{1}H NMR** (600 MHz, $CDCl_3$) δ 7.91 (d, *J* = 15.7 Hz, 2H), 7.25 (s, 1H), 6.29 (d, *J* = 7.6 Hz, 2H), 4.33-4.28 (m, 2H), 3.8 (dd, *J* = 11.0, 2.9 Hz, 2H), 3.66 (dd, *J* = 11.0, 5.7 Hz, 2H), 1.75-1.62 (m, 6H), 1.54-1.46 (m, 8H), 1.35 (s, 9H), 1.23-1.13 (m, 8H), 1.01-0.88 (m, 4H); **^{13}C NMR** (150 MHz, $(CD_3)_2SO$) δ 166.4, 151.1, 135.2, 126.9, 124.2, 64.3, 49.3, 35.2, 34.4, 34.0, 32.6, 31.5, 26.6, 26.2; **HRMS** [+APCI] calculated for $C_{30}H_{49}N_2O_4$ 501.3687, found 501.3685 $[M+H]^+$; $[\alpha]^{20}_D$ -15.5 ° (*c* 0.5, $CHCl_3$).

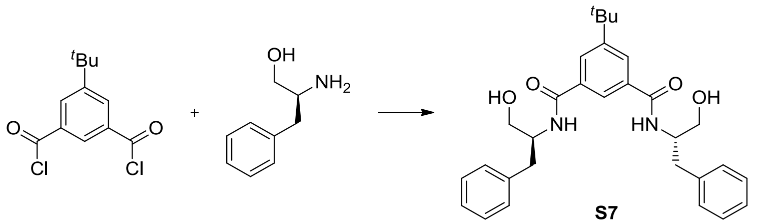

5-(*tert*-butyl)-*N*1,*N*3-bis((*S*)-1-hydroxy-3-phenylpropan-2-yl)isophthalamide (S7). Prepared by general procedure **B** using 5-*tert*-butylisophthaloyl dichloride (500 mg, 1.9 mmol) in isopropyl acetate (1.5 mL, 0.13 M in acyl chloride), (*S*)-2-amino-3-phenyl-1-propanol (590 mg, 3.9 mmol), 1.5 M $KHCO_3$ (13 mL, 19.4 mmol). Mixed solvent recrystallization (EtOAc/hexanes) afforded the title compound as a white solid (703 mg, 74 %); **mp** 76-79 ° C; **IR** (thin film, cm^{-1}) 3283, 2964, 1636, 1542, 1041; **^{1}H NMR** (400 MHz, $CDCl_3$) δ 7.73 (s, 1H), 7.61 (s, 2H), 7.28-7.19 (m, 10H), 7.08 (d, *J* = 12.8 Hz, 2H), 4.31 (br s, 2H), 3.76 (dd, *J* = 11.1, 2.9 Hz, 2H), 3.64 (dd, *J* = 11.6, 6.1 Hz, 2H), 2.96-2.86 (m, 4H), 1.15 (s, 9H); **^{13}C NMR** (100 MHz, $CDCl_3$) δ 172.5, 168.2, 151.9, 137.7, 134.4, 129.3, 128.6, 127.2, 126.6, 122.1, 63.6, 53.7, 37.1, 34.8, 30.9; **HMRS** (+NSI) calculated for $C_{30}H_{36}N_2O_4$ 489.2748, found 489.2743 $[M+H]^+$; **$[\alpha]^{20}_D$** -84.6 ° (*c* 1.0, $CHCl_3$).

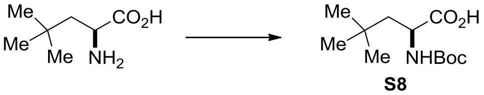

(*S*)-2-((*tert*-butoxycarbonyl)amino)-4,4-dimethylpentanoic acid (S8). Adapting a procedure from Christoffers and Schuster,[3] a 25 mL round bottom flask equipped with a stir bar was charged with (*S*)-2-amino-4,4-dimethylpentanoic acid (500 mg, 3.5 mmol, 1.0 equiv), H_2O (4 mL), MeOH (5 mL) and cooled to 0 º C with stirring. Na_2CO_3 (376 mg, 3.6 mmol, 1.0 equiv) was added slowly, followed by a solution of Boc_2O (799 mg, 3.7 mmol, 1.1 equiv) in MeOH (1.25 mL). The suspension became mostly clear, after which a white precipitate formed. The mixture was permitted to come to 23 º C and then stirred overnight. Water (5 mL) and then citric acid were added slowly at 23 º C until approximately pH 3 was achieved. The resulting solution was extracted with CH_2Cl_2, and the combined organic layers were dried with Na_2SO_4 and concentrated *in vacuo* to afford a white sticky solid (769 mg, 91 % yield) which was used without further purification; **mp** 96-98 º C; **IR** (neat, cm^{-1}) 3245, 3117, 2955, 1731, 1652, 1387, 1366, 1241, 1165, 902; **^{1}H NMR** (400 MHz, $CDCl_3$) δ 4.84 (d, *J* = 12 Hz, 1H), 4.34 (dt, *J* = 4, 8 Hz, 1H), 1.81 (dd, *J* = 4, 12 Hz, 1H), 1.44 (s, 9H), 0.98 (s, 9H); **^{13}C NMR** (100 MHz, $CDCl_3$) δ 178.9, 155.4, 80.8, 51.3, 45.9, 30.7, 29.5, 28.3; **HRMS** (+NSI) calculated for $C_{12}H_{24}O_4N$ 246.1699, found 246.1699 $[M+H]^+$; **$[\alpha]^{20}_D$** -21.6 ° (c 1.1, $CHCl_3$).

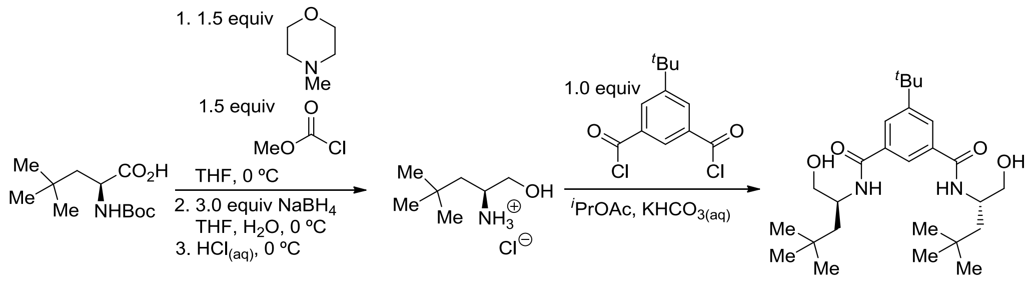

S9 S10 S11

5-(*tert*-butyl)-*N*[1],*N*[3]-bis((*S*)-1-hydroxy-4,4-dimethylpentan-2-yl)isophthalamide (S11). Adapting a procedure from Guaragna and coworkers,[4] a 100 mL round bottom flask equipped with a stir bar was flame dried and placed under N_2. The flask was charged with **S9** (340 mg, 1.4 mmol, 1.0 equiv) and THF (12 mL). The solution was stirred and cooled to 0 º C. *N*-methyl morpholine (230 µL, 2.1 mmol, 1.5 equiv) was then added dropwise, followed by methyl chloroformate (160 µL, 2.1 mmol, 1.5 equiv) also dropwise. The reaction was allowed to come to 23 º C, at which time a white precipitate was observed to have formed. After 2 h at 23 º C, the reaction was filtered through Celite and recooled to 0 ºC. $NaBH_4$ (158 mg, 4.2 mmol, 3.0 equiv) in H_2O (0.80 mL) was then added to the solution dropwise with stirring. Vigorous bubbling was observed. The reaction was permitted to stir another 30 min at 23 º C. Saturated NH_4Cl (aq) was then added, the mixture was extracted with Et_2O, and the organic layers were washed with saturated $CuSO_4$ (aq) and finally dried with Na_2SO_4. Concentration *in vacuo* afforded a clear oil (355 mg) which was used without further purification; **HRMS** (+NSI) calculated for $C_{12}H_{26}O_3N$ 232.1907, found 232.1908 $[M+H]^+$.

A 25 mL round bottom flask equipped with a stir bar was charged with the oil (355 mg, 1.0 mmol) and cooled to 0 ºC. Concentrated HCl (aq) (2 mL) was added and the mixture was vigorously stirred. Significant bubbling was observed. The reaction was then concentrated *in vacuo* after bubbling ceased to afford a white powder (226 mg) which was used without further purification; **HRMS** (+NSI) calculated for $C_7H_{18}ON$ 132.1383, found 132.1382$[M+H]^+$.

A 25 mL round bottom flask equipped with a stir bar and a condenser was flame dried and placed under N_2 atmosphere. The flask was charged with **S10** (226 mg, 2.3 equiv) and ^{i}PrOAc (8 mL) and vigorously stirred. $KHCO_3$ (4 mL, 1.5 M in H_2O, 6.0 mmol, 10.2 equiv) was added dropwise and the mixture was heated to 65 º C. Once reaching 65 º C, 5-(*tert*-butyl)-isophthaloyl dichloride (152 mg, 0.59 mmol, 1.0 equiv) was added slowly over 5 min. The mixture was then heated at 65 º C with stirring for 4 h under N_2. The reaction was then cooled to 23 º C, extracted with ^{i}PrOAc, dried with Na_2SO_4, concentrated *in vacuo*, and purified by flash chromatography (7:3 → 1:1 hexanes:EtOAc) to yield the title compound as a white solid (238 mg, 38% yield over 3 steps); $\mathbf{R_f}$ 0.08 (75:25 EtOAc:hexanes); **mp** 136-140 º C; **IR** (neat, cm^{-1}) 3243, 2952, 2867, 1630, 1550, 1466, 1365, 1261, 1047, 908; ^{1}**H NMR** (400 MHz, $CDCl_3$) δ 7.87 (t, *J* = 1.6 Hz, 1H), 7.72 (d, *J* = 1.6 Hz, 2H), 7.26 (d, *J* = 8.0 Hz, 2H), 4.32 (m, 2H), 3.74 (dd, *J* = 4.0, 11.2 Hz, 2H), 3.60 (dd, *J* = 6.8, 11.2 Hz, 2H), 1.55 (dd, *J* = 8.4, 14.4 Hz, 2H), 1.43 (dd, *J* = 3.2, 14.4 Hz, 2H), 1.18 (s, 9H), 0.94 (s, 18H); ^{13}**C NMR** (100 MHz, $CDCl_3$) δ 168.1 151.8, 134.7, 127.1, 121.9, 66.8, 49.5, 44.8, 34.8, 31.0, 30.4, 29.8; **HRMS** (+NSI) calculated for $C_{26}H_{44}$ N_2O_4Na 471.3199, found 471.3187 $[M+Na]^+$; $[\alpha]^{20}_D$ -38.3 ° (c 1.2, $CHCl_3$).

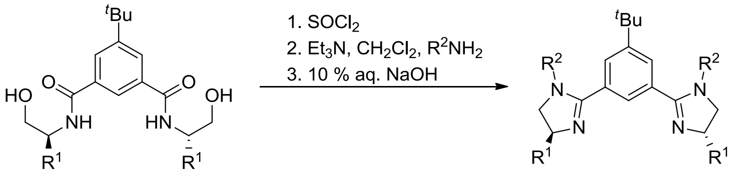

General Procedure C

A procedure was adapted from the literature as follows:[5] A 10 mL flask was charged with amide (1.0 equiv) fitted with a reflux condenser, and evacuated and backfilled with dry nitrogen. $SOCl_2$ (16 equiv) was added via syringe, and the mixture was refluxed for approximately 7 h. Excess $SOCl_2$ was removed directly under high vacuum. The residue was dissolved in dry CH_2Cl_2 (0.18 M) and cooled to 0 °C. Et_3N (6.0 equiv) and the primary aniline (2.2 equiv) were added sequentially, and the mixture was stirred overnight at ambient temperature. 10 % aqueous NaOH (15 equiv) was added and stirred for 30 minutes. The phases were separated, and the aqueous phase was extracted with CH_2Cl_2 (3 x). The combined organics were dried over Na_2SO_4, filtered, and concentrated *in vacuo*. The residue was purified by flash column chromatography on silica gel as indicated to give the title compound.

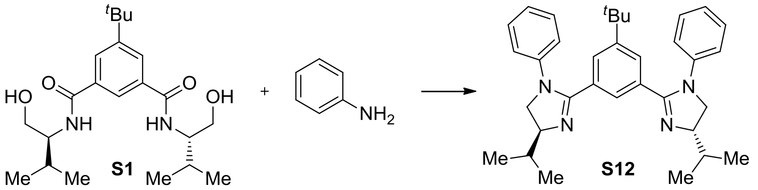

(4*S*,4'*S*)-2,2'-(5-(*tert*-butyl)-1,3-phenylene)bis(4-isopropyl-1-phenyl-4,5-dihydro-1H-imidazole) (S12). Prepared by general procedure **C** using amide **S1** (200 mg, 0.51 mmol), $SOCl_2$ (0.6 mL), Et_3N (0.43 mL, 3.1 mmol), CH_2Cl_2 (2.8 mL), aniline (104 mg, 1.1 mmol), and 10 % aq. NaOH (3 mL). Flash chromatography (EtOAc → 9:1 EtOAc:MeOH) afforded the title ligand as a light brown amorphous solid (230 mg, 89 %); $\mathbf{R_f}$ 0.08 (9:1 EtOAc:MeOH); **IR** (thin film, cm^{-1}) 2957, 2871, 1577, 1495, 1253, 730, 695; ^{1}**H NMR** (400 MHz, $CDCl_3$) δ 7.79 (s, 1H), 7.23 (d, *J* = 1.5 Hz, 2H), 7.12 (t, *J* = 7.9 Hz, 4H), 6.96 (t, *J* = 7.3 Hz, 2H), 6.73 (d, *J* = 7.6 Hz, 4H), 4.20-4.07 (m, 4H), 3.62 (dd, *J* = 8.8, 7.0 Hz, 2H), 1.97-1.91 (m, 2H), 0.99 (d, *J* = 6.7 Hz, 6H), 0.92 (d, *J* = 7.0 Hz, 6H), 0.88 (s, 9H); ^{13}**C NMR** (100 MHz, $CDCl_3$) δ 161.8, 150.7, 142.2, 128.9, 128.4, 124.3, 123.5, 69.0, 56.0, 34.6, 33.0, 30.8, 18.9, 17.8; **HRMS** [+APCI] calculated for $C_{34}H_{43}N_4$ 507.3482, found 507.3479 $[M+H]^+$; $[\alpha]^{20}_D$ +42.7 ° (*c* 1.0, $CHCl_3$).

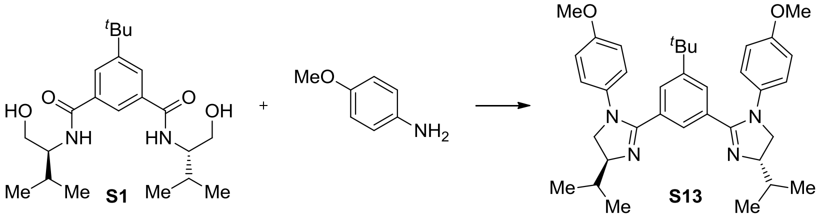

(4*S*,4'*S*)-2,2'-(5-(*tert*-butyl)-1,3-phenylene)bis(4-isopropyl-1-(4-methoxyphenyl)-4,5-dihydro-1H-imidazole) (S13). Prepared by general procedure **C** using amide **S1** (200 mg, 0.51 mmol), $SOCl_2$ (0.6 mL), Et_3N (0.43 mL, 3.1 mmol), CH_2Cl_2 (2.8 mL), 4-methoxyaniline (138 mg, 1.1 mmol), and 10 % aq. NaOH (3 mL). Flash chromatography (9:1 → 4:1 → 7:3 acetone:MeOH) afforded the title ligand as a white amorphous solid (229 mg, 79 %); $\mathbf{R_f}$ 0.13

(8:2 acetone:MeOH); **IR** (thin film, cm^{-1}) 2957, 1510, 1244; **^{1}H NMR** (400 MHz, $CDCl_3$) δ 7.77 (s, 1H), 7.25 (s, 2H), 6.73 (d, *J* = 8.9 Hz, 4H), 6.67 (d, *J* = 8.9 Hz, 4H), 4.12-4.01 (m, 4H), 3.69 (s, 6H), 3.49 (t, *J* = 7.1 Hz, 2H), 1.94 (dd, *J* = 12.3, 6.1 Hz, 2H), 1.00 (d, *J* = 7.0 Hz, 6H), 0.92 (d, *J* = 7.0 Hz, 6H), 0.90 (s, 9H); **^{13}C NMR** (100 MHz, $CDCl_3$) δ 162.2, 156.9, 128.3, 127.4, 125.8, 114.3, 77.4, 56.9, 55.7, 34.6, 33.1, 30.9, 19.0, 17.8; **HRMS** [+APCI] calculated for $C_{36}H_{47}N_4O_2$ 567.3694, found 567.3689 $[M+H]^+$; **$[\alpha]^{20}_D$** +77.8 ° (*c* 1.0, $CHCl_3$).

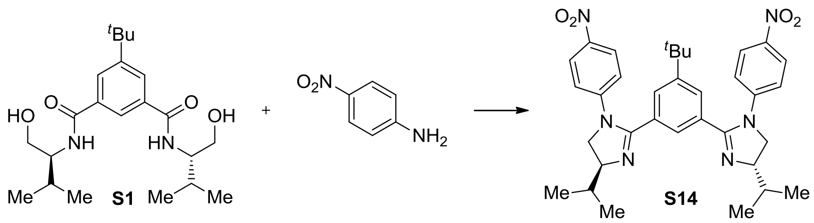

(4*S*,4'*S*)-2,2'-(5-(*tert*-butyl)-1,3-phenylene)bis(4-isopropyl-1-(4-nitrophenyl)-4,5-dihydro-1H-imidazole) (S14). Prepared by general procedure C using amide **S1** (200 mg, 0.51 mmol), $SOCl_2$ (0.6 mL), Et_3N (0.43 mL, 3.1 mmol), CH_2Cl_2 (3 mL), 4-nitroaniline (155 mg, 1.1 mmol), and 10 % aq. NaOH (3 mL). Flash chromatography (EtOAc → 95:5 → 93:7 EtOAc:MeOH) afforded the title ligand as a yellow solid (238 mg, 78 %); **R_f** 0.55 (95:5 EtOAc:MeOH); **mp** 120-122 ° C; **IR** (thin film, cm^{-1}) 2960, 1590, 1504, 1327; **^{1}H NMR** (400 MHz, $CDCl_3$) δ 7.98 (d, *J* = 8.6 Hz, 4H), 7.73 (s, 1H), 7.38 (s, 2H), 6.69 (d, *J* = 9.0 Hz, 4H), 4.19 (t, *J* = 9.8 Hz, 2H), 4.13-4.07 (m, 2H), 3.76 (dd, *J* = 8.6, 7.0 Hz, 2H), 1.92 (dd, *J* = 13.1, 6.8 Hz, 2H), 1.03 (s, 9H), 1.01 (d, *J* = 7.0 Hz, 6H), 0.91 (d, *J* = 6.7 Hz, 6H); **^{13}C NMR** (100 MHz, $CDCl_3$) δ 159.0, 152.1, 147.5, 141.8, 131.3, 128.1, 124.7, 119.8, 70.5, 55.4, 34.9, 33.0, 31.0, 19.1, 18.2; **HRMS** [+NSI] calculated for $C_{34}H_{41}N_6O_4$ 597.3184, found 597.3182 $[M+H]^+$; **$[\alpha]^{20}_D$** -9.8 ° (*c* 0.3, $CHCl_3$).

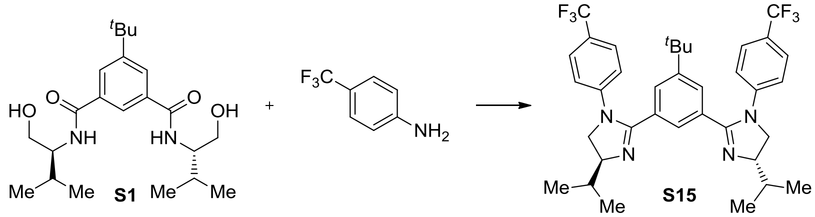

(4*S*,4'*S*)-2,2'-(5-(*tert*-butyl)-1,3-phenylene)bis(4-isopropyl-1-(4-(trifluoromethyl)phenyl)-4,5-dihydro-1H-imidazole) (S15). Prepared according to general procedure **C** using amide **S1** (200 mg, 0.51 mmol), $SOCl_2$ (0.6 mL), Et_3N (0.43 mL, 3.1 mmol), CH_2Cl_2 (3 mL), 4-trifluoromethylaniline (180 mg, 1.1 mmol), and 10 % aq. NaOH (3 mL). Flash chromatography (95:5 → 9:1 EtOAc:MeOH) afforded the title ligand as a light yellow amorphous solid (274 mg, 84 %); **R_f** 0.50 (9:1 EtOAc:MeOH); **IR** (thin film) 2960, 1613, 1324, 1121, 1072; **^{1}H NMR** (400 MHz, $CDCl_3$) δ 7.75 (s, 1H), 7.33 (d, *J* = 8.6 Hz, 4H), 7.29 (d, *J* = 1.2 Hz, 2H), 6.72 (d, *J* = 8.6 Hz, 4H), 4.17 (t, *J* = 9.8 Hz, 2H), 4.06 (dt, *J* = 10.6, 6.3 Hz, 2H), 3.66 (dd, *J* = 8.8, 6.8 Hz, 2H), 1.89 (dq, *J* = 13.0, 6.5 Hz, 2H), 1.00 (d, *J* = 6.7 Hz, 6H), 0.95 (s, 9H), 0.90 (d, *J* = 6.7 Hz, 6H); **^{13}C NMR** (100 MHz, $CDCl_3$) δ 160.1, 151.3, 145.4, 131.0, 128.1, 126.6, 125.9, 124.7, 122.9, 121.5, 70.3, 55.5, 34.7, 33.1, 30.8, 19.0, 17.9; **HRMS** [+NSI] calculated for $C_{36}H_{41}F_6N_4$ 643.3229, found 643.3211 $[M+H]^+$; **$[\alpha]^{20}_D$** +10.6 ° (*c* 0.4, $CHCl_3$).

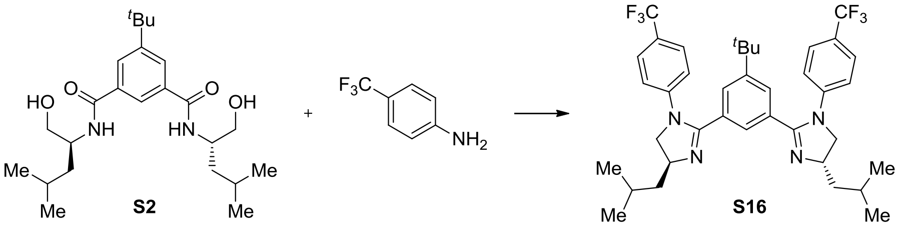

(4*S*,4'*S*)-2,2'-(5-(*tert*-butyl)-1,3-phenylene)bis(4-isobutyl-1-(4-(trifluoromethyl)phenyl)-4,5-dihydro-1H-imidazole) (S16). Prepared by general procedure **C** using amide **S2** (950 mg, 2.3 mmol), $SOCl_2$ (2.8 mL), Et_3N (2.0 mL, 14.3 mmol), CH_2Cl_2 (13 mL), 4-trifluoromethylaniline (180 mg, 1.12 mmol), and 10 % aq. NaOH (14 mL). Flash chromatography (4:5 EtOAc:hexanes) afforded the title ligand as a colorless oil (793 mg, 52 %); $\mathbf{R_f}$ 0.66 (1:1 hexanes:EtOAc); **IR** (thin film) 2959, 2158, 2009, 1613, 1324, 1122, 750; ^{1}**H NMR** (400 MHz, $CDCl_3$) δ 7.76 (s, 1H), 7.33 (d, *J* = 8.6 Hz, 4H), 7.24 (s, 2H), 6.71 (d, *J* = 8.6 Hz, 4H), 4.31-4.23 (m, 2H), 4.17 (t, *J* = 9.2 Hz, 2H), 3.63 (t, *J* = 8.2 Hz, 2H), 1.83 (dt, *J* = 13.3, 6.7 Hz, 2H), 1.74 (dt, *J* = 13.3, 6.7 Hz, 2H), 1.43-1.41 (m, 2H), 0.98-0.94 (m, 21H); ^{13}**C NMR** (100 MHz, $CDCl_3$): δ 159.8, 151.2, 145.4, 131.1, 128.1, 126.7, 125.9, 121.1, 63.1, 58.8, 46.1, 34.7, 30.8, 25.5, 23.2, 22.8; **HRMS** [+NSI] calculated for $C_{38}H_{45}N_4F_6$ 671.3543, found 671.3523 $[M+H]^+$; $\mathbf{[\alpha]^{20}_D}$ -31.1 ° (*c* 1.0, $CHCl_3$).

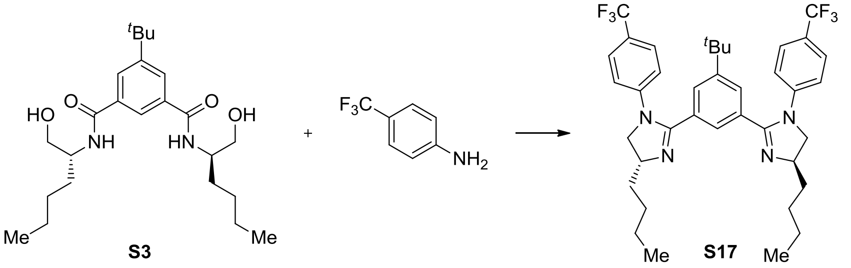

(4*R*,4'*R*)-2,2'-(5-(*tert*-butyl)-1,3-phenylene)bis(4-butyl-1-(4-(trifluoromethyl)phenyl)-4,5-dihydro-1H-imidazole) (S17). Prepared according to general procedure **C** using amide **S3** (193 mg, 0.43 mmol), $SOCl_2$ (0.7 mL), Et_3N (0.4 mL, 2.9 mmol), CH_2Cl_2 (2.3 mL), 4-trifluoromethylaniline (0.12 mL, 0.96 mmol), and 10 % aq. NaOH (2.5 mL). Flash chromatography (EtOAc → 9:1 EtOAc:methanol) afforded the title ligand as an oil (110 mg, 38 %); $\mathbf{R_f}$ 0.32 (9:1 EtOAc:methanol); **IR** (thin film, cm^{-1}) 2962, 2933, 2873, 1612, 1522, 1323, 1123, 1070; ^{1}**H NMR** (400 MHz, $CDCl_3$) δ 7.79 (s, 1H), 7.33 (d, *J* = 8.5 Hz, 4H), 7.24 (s, 2H), 6.72 (d, *J* = 8.2 Hz, 4H), 4.24-4.14 (m, 4H), 3.66 (t, *J* = 7.3 Hz, 2H), 1.83-1.77 (m, 2H), 1.59-1.52 (m, 2H), 1.43-133 (m, 8H), 0.94 (t, *J* = 4.9 Hz, 9H), 0.91 (t, *J* = 6.9 Hz, 6H); ^{13}**C NMR** (150 MHz, $CDCl_3$) δ 159.6, 156.2, 151.0, 145.1, 130.9, 127.8, 126.4, 125.6, 121.0, 64.5, 58.0, 36.0, 34.5, 30.6, 30.5, 27.9, 22.7, 13.9; **HMRS** (+NSI) calculated for $C_{38}H_{45}N_4F_6$ 671.3554, found 671.3550 $[M+H]^+$; $\mathbf{[\alpha]^{20}_D}$ +8.1 ° (*c* 1.0, $CHCl_3$).

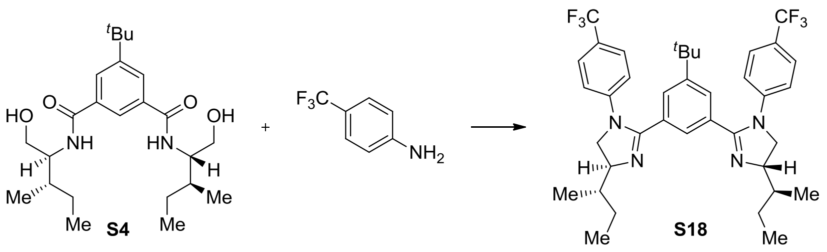

(*S*)-4-((*S*)-*sec*-butyl)-2-(3-(*tert*-butyl)-5-((*S*)-4-((*S*)-*sec*-butyl)-1-(4-(trifluoromethyl)phenyl)-4,5-dihydro-1H-imidazol-2-yl)phenyl)-4-methyl-1-(4-(trifluoromethyl)phenyl)-4,5-dihydro-1H-imidazole. (S18). Prepared by general procedure **C** using amide **S4** (215 mg, 0.51 mmol), $SOCl_2$ (0.6 mL), Et_3N (0.43 mL, 3.1 mmol), CH_2Cl_2 (3 mL), 4-trifluoromethylaniline (180 mg, 1.1 mmol), and 10 % aq. NaOH (3 mL). Flash chromatography (EtOAc → 95:5 → 9:1 EtOAc:MeOH) afforded the title ligand as a white amorphous solid (220 mg, 64 %); **R$_f$** 0.61 (95:5 EtOAc:MeOH); **IR** (thin film) 2965, 1614, 1537, 1325, 1130, 751; **^{1}H NMR** (600 MHz, $CDCl_3$) δ 8.10 (s, 1H), 7.45 (d, *J* = 8.1 Hz, 4H), 7.17 (s, 2H), 7.06 (d, *J* = 5.7 Hz, 4H), 4.40-4.37 (m, 2H), 4.32 (t, *J* = 10.0 Hz, 2H), 3.85 (t, *J* = 8.3 Hz, 2H), 1.93 (s, 2H), 1.59-1.52 (m, 2H), 1.27-1.22 (m, 2H), 0.93 (t, *J* = 7.6 Hz, 12H), 0.81 (s, 9H); **^{13}C NMR** (150 MHz, $CDCl_3$) δ 173.6, 161.9, 151.6, 142.4, 129.5, 126.4, 124.4. 123.7, 122.6, 64.9, 54.9, 38.3, 34.4, 30.2, 25.5, 21.4, 13.6, 11.4; **HRMS** [+APCI] calculated for $C_{38}H_{45}N_4F_6$ 671.3543, found 671.3540 $[M+H]^+$; **$[\alpha]^{20}_D$** +30.2 ° (*c* 1.0, $CHCl_3$).

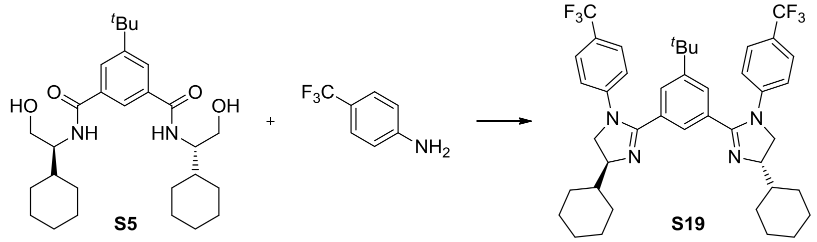

(4*S*,4'*S*)-2,2'-(5-(*tert*-butyl)-1,3-phenylene)bis(4-cyclohexyl-1-(4-(trifluoromethyl)phenyl)-4,5-dihydro-1H-imidazole) (S19). Prepared by general procedure **C** using amide **S5** (482 mg, 1.0 mmol), $SOCl_2$ (1.2 mL), Et_3N (0.86 mL, 6.2 mmol), CH_2Cl_2 (6 mL), 4-trifluoromethylaniline (362 mg, 2.2 mmol), and 10 % aq. NaOH (6 mL). Flash chromatography (EtOAc → 98:2 → 95:5 EtOAc:MeOH) afforded the title ligand as a light brown amorphous solid (155 mg, 21 %); **R$_f$** 0.13 (EtOAc); **IR** (thin film) 2929, 2855, 2366, 2043, 1709, 1613, 1325, 753; **^{1}H NMR** (600 MHz, $CDCl_3$) δ 7.78 (s, 1H), 7.32 (d, *J* = 8.6 Hz, 4H), 7.25 (d, *J* = 4.8 Hz, 2H), 6.71 (d, *J* = 8.6 Hz, 4H), 4.15 (t, *J* = 10.0 Hz, 2H), 4.05 (dt, *J* = 10.0, 6.7 Hz, 2H), 3.69 (dd, *J* = 8.8, 7.4 Hz, 2H), 1.90 (d, *J* = 12.4 Hz, 2H), 1.76 (t, *J* = 11.2 Hz, 4H), 1.66 (d, *J* = 11.9 Hz, 4H), 1.59 (dtd, *J* = 14.8, 7.4, 3.1 Hz, 2H), 1.30-1.20 (m, 4H), 1.15 (tt, *J* = 12.8, 2.9 Hz, 2H), 1.05 (dqd, *J* = 24.2, 12.1, 3.2 Hz, 4H), 0.93 (s, 9H); **^{13}C NMR** (150 MHz, $CDCl_3$) δ 160.0, 151.2, 145.4, 131.0, 128.1, 126.6, 125.8, 125.2, 123.4, 121.4, 69.6, 55.7, 43.1, 34.7, 30.8, 29.6, 28.5, 26.7, 26.3; **HRMS** [+APCI] calculated for $C_{42}H_{49}F_6N_4$ 723.3861, found 723.3864 $[M+H]^+$; **$[\alpha]^{20}_D$** -11.8 ° (*c* 0.3, $CHCl_3$).

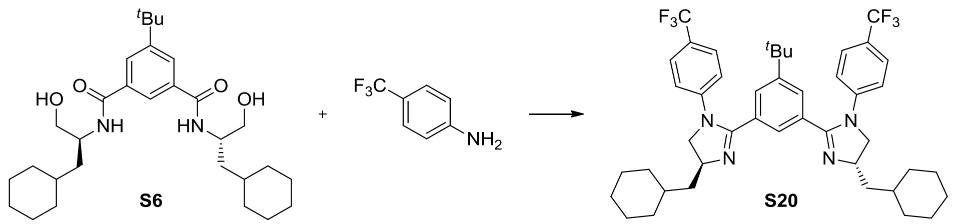

(4*S*,4'*S*)-2,2'-(5-(*tert*-butyl)-1,3-phenylene)bis(4-(cyclohexylmethyl)-1-(4-(trifluoromethyl)phenyl)-4,5-dihydro-1H-imidazole) (S20). Prepared by general procedure **C** using amide **S6** (130 mg, 0.26 mmol), $SOCl_2$ (0.31 mL), Et_3N (0.4 mL, 2.9 mmol), CH_2Cl_2 (1.4 mL), 4-trifluoromethylaniline (51 μL, 0.56 mmol), and 10 % aq. NaOH (1.5 mL). Flash chromatography (1:1 hexanes:EtOAc → 3:7 hexanes:EtOAc → EtOAc) afforded the title ligand as a white amorphous solid (123 mg, 63 %); $\mathbf{R_f}$ 0.57 (3:7 hexanes/EtOAc); **IR** (thin film) 3843, 3019, 2344, 2147, 1710, 1214, 744; **^{1}H NMR** (600 MHz, $CDCl_3$) δ 7.80 (s, 1H), 7.32 (d, *J* = 8.6 Hz, 4H), 7.23 (t, *J* = 1.4 Hz, 2H), 6.71 (d, *J* = 8.6 Hz, 4H), 4.31 (dt, *J* = 15.9, 7.8 Hz, 2H), 4.17 (t, *J* = 9.3 Hz, 2H), 3.62 (t, *J* = 8.3 Hz, 2H), 1.82-1.73 (m, 6H), 1.72-1.63 (m, 8H), 1.50 (ddd, *J* = 10.6, 7.0, 3.8 Hz, 2H), 1.38 (dt, *J* = 13.6, 7.0 Hz, 2H), 1.28-1.23 (m, 4H), 1.19-1.15 (m, 2H) 1.00-0.96 (m, 4H), 0.93 (s, 9H); **^{13}C NMR** (150 MHz, $CDCl_3$) δ 159.7, 145.5, 131.3, 128.0, 126.7, 125.8, 121.1, 62.5, 58.9, 44.8, 34.9, 33.9, 33.5, 30.8, 26.8, 26.5; **HRMS** [+APCI] calculated for $C_{44}H_{53}F_6N_4$ 751.4169, found 751.4172 $[M+H]^+$; **$[\alpha]^{20}_D$** - 26.3 ° (*c* 1.0, $CHCl_3$).

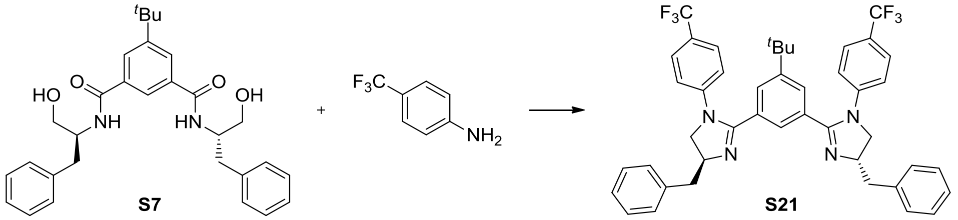

(4*S*,4'*S*)-2,2'-(5-(*tert*-butyl)-1,3-phenylene)bis(4-benzyl-1-(4-(trifluoromethyl)phenyl)-4,5-dihydro-1H-imidazole) (S21). Prepared according to general procedure C, using amide **S7** (420 mg, 0.86 mmol), $SOCl_2$ (1.1 mL), Et_3N (0.73 mL, 5.2 mmol), CH_2Cl_2 (4.7 mL), 4-trifluoromethylaniline (0.25 mL, 2.0 mmol), and 10 % aq. NaOH (5 mL). Flash chromatography (EtOAc → 9:1 EtOAc:MeOH) afforded the title compound as a yellow amorphous solid (303 mg, 48 %); $\mathbf{R_f}$ 0.24 (9:1 EtOAc:methanol); **IR** (thin film, cm^{-1}) 2969, 2195, 1611, 1521, 1376, 1321, 1163; **^{1}H NMR** (400 MHz, $CDCl_3$) δ 7.72 (t, *J* = 1.5 Hz, 1H), 7.29 (dd, *J* = 4.9, 3.4 Hz, 6H), 7.21-7.15 (m, 10H), 6.54 (d, *J* = 8.5 Hz, 4H), 4.58-4.51 (m, 2H), 4.11 (t, *J* = 9.6 Hz, 2H), 3.70 (dd, *J* = 9.5, 6.1 Hz, 2H), 3.16 (dd, *J* = 13.7, 4.6 Hz, 2H), 2.81 (dd, *J* = 13.6, 8.1 Hz, 2H), 0.98 (s, 9H); **^{13}C NMR** (100 MHz, $CDCl_3$) δ 160.6, 151.5, 145.3, 137.9, 131.1, 129.7, 128.6, 128.1, 128.06, 126.7, 126.5, 125.8, 124.8, 124.5, 123.0, 121.3, 65.5, 57.3, 42.0, 34.8, 30.8; **HMRS** (+NSI) calculated for $C_{44}H_{41}N_4F_6$ 739.3229, found 739.3227 $[M+H]^+$; **$[\alpha]^{20}_D$** +164.4 ° (*c* 1.0, $CHCl_3$).

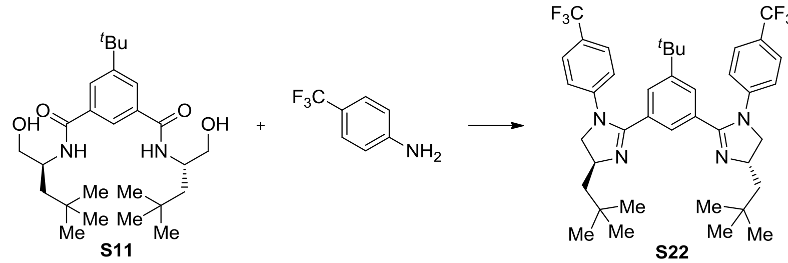

S11 **S22**

(4*S*,4'*S*)-2,2'-(5-(*tert*-butyl)-1,3-phenylene)bis(4-neopentyl-1-(4-(trifluoromethyl)phenyl)-4,5-dihydro-1H-imidazole) (S22). Prepared according to general procedure C, using amide **S11** (95 mg, 0.21 mmol, 1.0 equiv), $SOCl_2$ (1.0 mL), Et_3N (200 μL, 1.43 mmol, 6.8 equiv), CH_2Cl_2 (2.0 mL), 4-trifluoromethylaniline (75 μL, 0.598, 2.8 equiv), and 10 % aq. NaOH (3 mL). Flash chromatography (8:2 hexanes:EtOAc → EtOAc) afforded the title compound as an amorphous off-white solid (99 mg, 67% yield); $\mathbf{R_f}$ 0.05 (very streaky, 100% EtOAc). **IR** (neat, cm^{-1}) 2955, 2902, 1612, 1521, 1321, 1117, 1070, 834, 731; ^{1}**H NMR** (400 MHz, $CDCl_3$) δ 7.76 (t, *J* =1.6 Hz, 1H), 7.34 (d, *J* = 8.0 Hz, 4H), 7.26 (d, *J* = 1.6 Hz, 2H), 6.73 (d, *J* = 8.0 Hz, 4H), 4.25 (dd, *J* =8.0, 14.0 Hz, 4H), 3.66 (m, 2H), 1.94 (dd, *J* = 4.0, 14.0 Hz, 2H), 1.46 (dd, *J* = 8.0, 14.0 Hz, 2H), 1.00 (s, 18H), 0.96 (s, 9H); ^{13}**C NMR** (100 MHz, $CDCl_3$) δ 159.1, 150.9, 145.2, 130.8, 127.7, 126.4, 125.6 (q, *J* = 4 Hz), 124.0 (q, *J* = 33 Hz), 122.7, 120.8, 62.0, 60.4, 51.0, 34.5, 30.6, 30.4, 30.0; ^{19}**F NMR** (375 MHz, $CDCl_3$) δ –65.2 (s, 6F); (–164.9, C_6F_6); **HRMS** (+NSI) calculated for $C_{40}H_{49}N_4F_6$ 699.3856, found 699.3850 $[M+H]^+$; $[\alpha]^{20}_D$ -100.8 ° (c 1.1, $CHCl_3$).

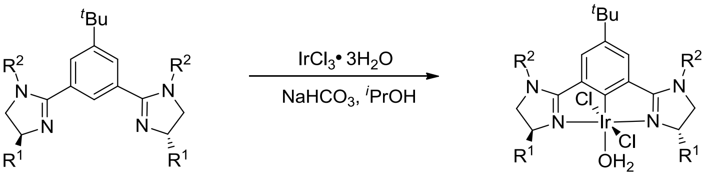

General procedure D. A dry round bottom flask was charged with ligand (1.0 equiv.), $IrCl_3{\cdot}3H_2O$ or $IrBr_3{\cdot}4H_2O$ (1.1 equiv.), and $NaHCO_3$ (1.1 equiv.). The flask was fitted with a reflux condenser and evacuated and backfilled with nitrogen. ^{i}PrOH (0.03 M) was added, and the mixture was refluxed until the ligand was consumed as judged by thin-layer chromatography. The reaction mixture was cooled to room temperature and concentrated *in vacuo*. The crude solid was purified by flash column chromatography on silica gel or by preparative thin layer chromatography as indicated to give the iridium(III) phebim complex.

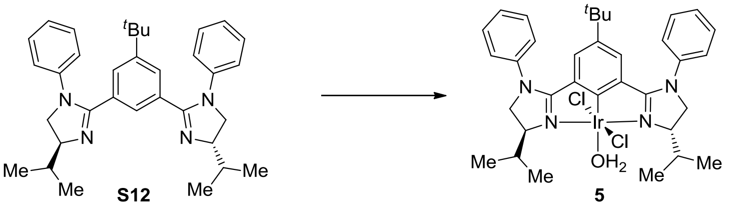

S12 **5**

[(*S*, *S*)-*t*-ButylPhebim-Ph-*i*-Pr]IrCl$_2$(H$_2$O) (5). Prepared by general procedure **D** using ligand **S12** (139 mg, 0.27 mmol), $IrCl_3{\cdot}3H_2O$ (106 mg, 0.30 mmol), and $NaHCO_3$ (25 mg, 0.30 mmol). The reaction was refluxed in ^{i}PrOH (9 mL) for 5.5 h. Flash chromatography (1:1 hexanes:EtOAc) afforded iridium(III) phebim **5** as a dark red solid (59 mg, 27 %); $\mathbf{R_f}$ 0.67 (1:1 hexanes:EtOAc); **mp** 192 ° C; **IR** (thin film, cm^{-1}) 3353, 2954, 1493, 1288, 730, 696; ^{1}**H NMR** (600 MHz, $CDCl_3$) δ 7.40 (t, *J* = 7.9 Hz, 4H), 7.31-7.27 (m, 6H), 6.52 (s, 2H), 4.41 (dd, *J* = 11.1,

9.4 Hz, 2H), 4.30-4.27 (m, 2H), 4.05 (dd, J = 9.3, 5.6 Hz, 2H), 2.94 (br s, 2H), 2.54-2.50 (m, 2H), 0.98 (dd, J = 14.8, 6.9 Hz, 12H), 0.79 (s, 9H); **^{13}C NMR** (150 MHz, $CDCl_3$) δ 170.5, 141.3, 132.2, 129.4, 127.3, 126.4, 125.4, 68.0, 54.9, 34.4, 31.4, 29.7, 19.4, 15.4; **HRMS** [+NSI] calculated for $C_{34}H_{41}Cl_2IrN_4$ 768.23320, found 768.23158 $[M\text{-}H_2O]^+$; **$[\alpha]^{20}_D$** +24.9 ° (c 0.1, $CHCl_3$).

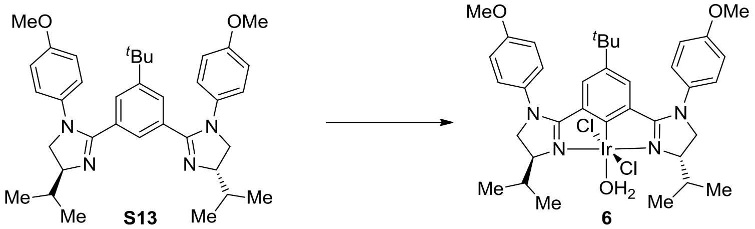

S13 6

[(*S*, *S*)-*t*-ButylPhebim-4-OMe-Ph-*i*-Pr]$IrCl_2(H_2O)$ (6). Prepared by general procedure **D** using ligand **S13** (35 mg, 0.062 mmol), $IrCl_3·3H_2O$ (24 mg, 0.068 mmol), and $NaHCO_3$ (6 mg, 0.068 mmol). The reaction was refluxed in ^{i}PrOH (2 mL) for 12 h. Preparative thin layer chromatography (2:3 hexanes:EtOAc) afforded iridium(III) phebim **6** as a dark red solid (20 mg, 38 %); **R_f** 0.67 (2:3 hexanes:EtOAc); **mp** 190 ° C; **IR** (thin film, cm^{-1}) 3353, 2956, 1510, 1244, 1033, 749; **^{1}H NMR** (600 MHz, $CDCl_3$) δ 7.21 (d, J = 8.6 Hz, 4H), 6.91 (d, J = 9.1 Hz, 4H), 6.39 (s, 2H), 4.31-4.28 (m, 2H), 4.26-4.24 (m, 2H), 3.99 (dd, J = 8.8, 5.5 Hz, 2H), 3.81 (s, 6H), 2.50-2.48 (m, 2H), 2.32 (br s, 2H), 1.00 (d, J = 6.2 Hz, 6H), 0.95 (d, J = 6.7 Hz, 6H), 0.79 (s, 9H); **^{13}C NMR** (150 MHz, $CDCl_3$) δ 170.8, 159.0, 142.7, 134.1, 132.3, 128.4, 125.3, 114.7, 68.0, 55.9, 55.2, 34.4, 31.4, 29.9, 19.4, 15.5; **HRMS** [+APCI] calculated for $C_{36}H_{43}N_4Cl_2Ir$ 826.2387, found 826.2397 $[M\text{-}H_2O]^+$; **$[\alpha]^{20}_D$** +41.8 ° (c 0.1, $CHCl_3$).

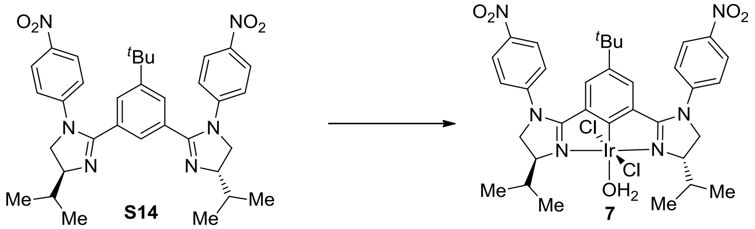

S14 7

[(*S*, *S*)-*t*-ButylPhebim-4-NO_2-Ph-*i*-Pr]$IrCl_2(H_2O)$ (7). Prepared by general procedure **D** using ligand **S14** (44 mg, 0.074 mmol), $IrCl_3·3H_2O$ (29 mg, 0.081 mmol), and $NaHCO_3$ (7 mg, 0.081 mmol). The reaction was refluxed in ^{i}PrOH (2.5 mL) for 24 h. Preparative thin layer chromatography (1:1 hexanes:EtOAc) afforded iridium(III) phebim **7** as a dark red solid (10 mg, 15 %); **R_f** 0.48 (2:3 hexanes:EtOAc); **mp** 210 ° C; **IR** (thin film, cm^{-1}) 3357, 2957, 1595, 1519, 1323, 1111, 752; **^{1}H NMR** (600 MHz, $CDCl_3$) δ 8.29 (d, 4H, J = 9.1 Hz), 7.34 (d, J = 9.1 Hz, 4H), 7.01 (s, 2H), 4.58 (t, J = 10.2 Hz, 3H), 4.32 (br s, 2H), 4.09 (dd, J = 9.8, 6.0 Hz, 2H), 2.50 (br s, 2H), 1.01-0.95 (m, 15H), 0.89 (d, J = 6.7 Hz, 6H); **^{13}C NMR** (150 MHz, $CDCl_3$) δ 195.5, 187.4, 146.7, 144.7, 132.2, 125.9, 125.0, 123.4, 68.4, 54.6, 34.9, 31.7, 29.9, 29.4, 19.5, 15.3; **HRMS** [+APCI] calculated for $C_{34}H_{38}O_4N_6Ir$ 787.2578, found 787.2578 $[M\text{-}H_2O\text{-}Cl\text{-}HCl]^+$; **$[\alpha]^{20}_D$** +8.1 ° ($c$ 0.1, $CHCl_3$).

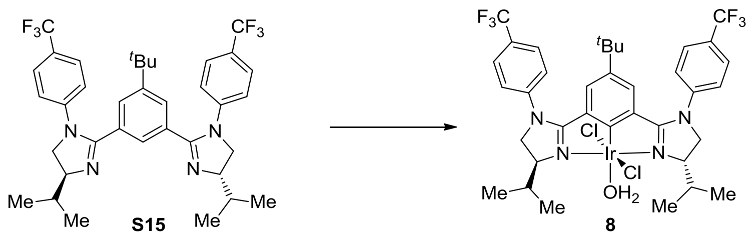

[(*S*, *S*)-*t*-ButylPhebim-4-CF$_3$-Ph-*i*-Pr]IrCl$_2$(H$_2$O) (8). Prepared by general procedure **D** using ligand **S15** (177 mg, 0.28 mmol), IrCl$_3$·3H$_2$O (109 mg, 0.31 mmol), and NaHCO$_3$ (26 mg, 0.31). The reaction was refluxed in ^{i}PrOH (9 mL) for 4.5 h. Flash chromatography (7:3 → 1:1 hexanes:EtOAc) afforded iridium(III) phebim **8** as a dark red solid (157 mg, 61 %); **R$_f$** 0.64 (1:1 hexanes:EtOAc); **mp** 222 ° C; **IR** (thin film, cm^{-1}) 2959, 2424, 2133, 1539, 1455, 1228, 782; **^{1}H NMR** (400 MHz, CDCl$_3$) δ 7.68 (d, *J* = 8.3 Hz, 4H), 7.38 (d, *J* = 8.3 Hz, 4H), 6.73 (s, 2H), 4.49 (t, *J* = 10.1 Hz, 2H), 4.35 (br s, 2H), 4.11-4.04 (m, 2H), 3.10 (br s, 2H), 2.54-2.51 (m, 2H), 0.98-0.93 (m, 12H), 0.89 (s, 9H); **^{13}C NMR** (100 MHz, CDCl$_3$) δ 170.3, 144.5, 143.0, 132.4, 128.6, 128.3, 126.5, 125.6, 125.2, 122.7, 68.3, 54.6, 34.6, 31.4, 29.6, 19.6, 15.5; **^{19}F NMR** (375 MHz, CDCl$_3$) δ –65.5 (s, 6F); (–164.9, C$_6$F$_6$); **HRMS** [+APCI] calculated for C$_{36}$H$_{39}$Cl$_2$F$_6$N$_4$Ir 904.2080, found 904.2089 [M-H$_2$O]$^+$; **[α]$^{20}_D$** +22.6 ° (*c* 0.3, CHCl$_3$).

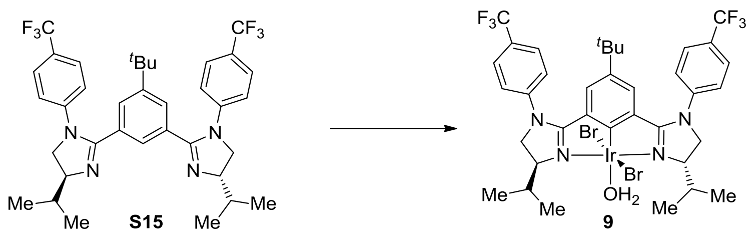

[(*S*, *S*)-*t*-ButylPhebim-4-CF$_3$-Ph-*i*-Pr]IrBr$_2$(H$_2$O) (9). Prepared by general procedure **D** using ligand **S15** (54 mg, 0.084 mmol), IrBr$_3$·4H$_2$O (47 mg, 0.092 mmol), and NaHCO$_3$ (8 mg, 0.092 mmol). The reaction was refluxed in ^{i}PrOH (3 mL) for 7.5 h. Flash chromatography (7:3 → 1:1 hexanes:EtOAc) afforded iridium(III) phebim **9** as a dark red solid (45 mg, 53 %); **R$_f$** 0.30 (7:3 hexanes:EtOAc); **mp** 186 ° C; **IR** (thin film, cm^{-1}) 3374, 2959, 1321, 1614, 1484, 1125, 1068, 845, 733; **^{1}H NMR** (400 MHz, CDCl$_3$) δ 7.68 (d, *J* = 8.2 Hz, 4H), 7.37 (d, *J* = 8.2 Hz, 4H), 6.75 (s, 2H), 4.52 (t, *J* = 10.1 Hz, 2H), 4.33 (br s, 2H), 4.08-4.05 (m, 2H), 2.52 (br s, 2H), 0.98 (app t, *J* = 7.6 Hz, 12H), 0.90 (s, 9H); **^{13}C NMR** (100 MHz, CDCl$_3$) δ 169.9, 144.5, 143.1, 132.2, 128.5, 126.5, 125.5, 125.0, 122.6, 68.5, 54.8, 34.5, 31.4, 29.3, 19.6, 15.7; **^{19}F NMR** (375 MHz, CDCl$_3$) δ –65.5 (s, 6F); (–164.9, C$_6$F$_6$); **HRMS** [+NSI] calculated for C$_{36}$H$_{40}$BrF$_6$IrN$_4$O 930.1919, found 930.2139 [M-HBr]$^+$; **[α]$^{20}_D$** +25.2 ° (*c* 0.1, CHCl$_3$).

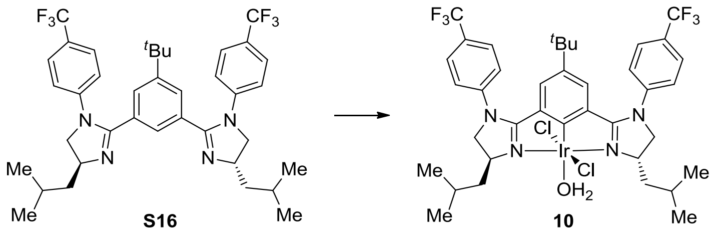

[(*S*, *S*)-*t*-ButylPhebim-4-CF$_3$-Ph-*i*-Bu]IrCl$_2$(H$_2$O) (10). Prepared by general procedure **D** using ligand **S16** (749 mg, 1.12 mmol), IrCl$_3$·3H$_2$O (445 mg, 1.23 mmol), and NaHCO$_3$ (103 mg,

1.23 mmol). The reaction was refluxed in ^{i}PrOH (37 mL) for 4.5 h. Flash chromatography (7:3 hexanes:EtOAc) afforded iridium(III) phebim **10** as a dark red solid (545 mg, 51 %); **R$_f$** 0.56 (7:3 hexanes:EtOAc); **mp** 218 ° C; **IR** (thin film, cm^{-1}) 3734, 3045, 2357, 2024, 1699, 1558, 1226, 782, 694; **^{1}H NMR** (400 MHz, $CDCl_3$) δ 7.68 (d, *J* = 8.6 Hz, 4H), 7.39 (d, *J* = 8.2 Hz, 4H), 6.65 (s, 2H), 4.53 (t, *J* = 10.6 Hz, 2H), 4.41-4.34 (m, 2H), 4.07 (dd, *J* = 9.2, 6.8 Hz, 2H), 2.77 (s, 2H), 2.20-2.13 (m, 2H), 1.74-1.65 (m, 4H), 1.02 (t, *J* = 6.5 Hz, 12H), 0.86 (s, 9H); **^{13}C NMR** (150 MHz, $CDCl_3$) δ 169.7, 144.4, 142.9, 132.2, 128.6, 126.4, 125.44, 125.31, 62.3, 60.0, 43.7, 34.4, 31.3, 25.7, 24.1, 21.8, 18.6; **^{19}F NMR** (375 MHz, $CDCl_3$) δ –65.6 (s, 6F); (–164.9, C_6F_6); **HRMS** [+APCI] calculated for $C_{38}H_{43}N_4Cl_2F_6Ir$ 932.2393, found 932.2400 $[M\text{-}H_2O]^+$; **$[\alpha]^{20}_D$** +15.9 ° (*c* 0.1, $CHCl_3$).

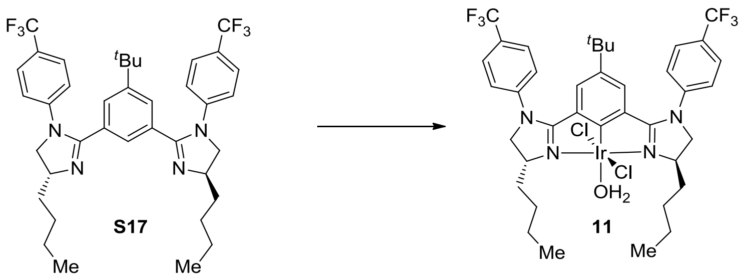

[(*R*, *R*)-*t*-ButylPhebim-4-CF$_3$Ph-*n*-Bu]IrCl$_2$(H$_2$O) (11). Prepared by general procedure **D** using **S17** (110 mg, 0.16 mmol), $IrCl_3{\cdot}3H_2O$ (65 mg, 0.18 mmol), and $NaHCO_3$ (17 mg, 0.20 mmol). The reaction was refluxed in ^{i}PrOH (5 mL) for 5 h. Preparative thin layer chromatography (7:3 hexanes:EtOAc) afforded iridium(III) phebim **11** as an orange solid (83 mg, 55 %); **R$_f$** 0.45 (7:3 hexanes:EtOAc); **mp** 230 ° C; **IR** (thin film, cm^{-1}) 3334, 2959, 2049, 1614, 1526, 1453, 1323, 1127, 1069; **^{1}H NMR** (400 MHz, $CDCl_3$) δ 7.60 (d, *J* = 9.2 Hz, 4H), 7.41 (d, *J* = 7.9 Hz, 4H), 6.68 (s, 2H), 4.54 (t, *J* = 9.8 Hz, 2H), 4.35 (br s, 2H), 4.07 (dd, *J* = 9.1, 6.7 Hz, 2H), 2.15 (br s, 2H), 1.82-1.74 (m, 2H), 1.47-1.24 (m, 9H) 0.91-0.82 (m, 14H); **^{13}C NMR** (100 MHz, $CDCl_3$) δ 171.7, 145.9, 143.2, 131.9, 126.5, 126.4, 126.1, 126.0, 64.7, 59.0, 34.2, 30.9, 26.9, 22.5, 13.9; **HMRS** (+APCI) calculated for $C_{38}H_{43}Cl_2F_6N_4Ir$ 932.2393, found 932.2388 $[M\text{-}H_2O]^+$; **$[\alpha]^{20}_D$** -18.9 ° (*c* 0.1, $CHCl_3$).

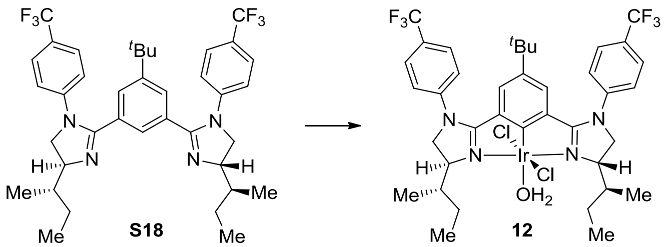

[(*S*, *S*)-*t*-ButylPhebim-4-CF$_3$-Ph-*s*-Bu]IrCl$_2$(H$_2$O) (12). Prepared by general procedure **D** using ligand **S18** (100 mg, 0.15 mmol), $IrCl_3{\cdot}3H_2O$ (60 mg, 0.17 mmol), and $NaHCO_3$ (15 mg, 0.17 mmol). The reaction was refluxed in ^{i}PrOH (5 mL) for 7.5 h. Flash chromatography (1:1 → 3:7 hexanes:EtOAc) afforded iridium(III) phebim complex **12** as a dark red solid (50 mg, 35 %); **R$_f$** 0.36 (1:1 hexanes:EtOAc); **mp** 190 ° C; **IR** (thin film, cm^{-1}) 3853, 3019, 2361, 1714, 1540, 1214, 745, 668; **^{1}H NMR** (600 MHz, $CDCl_3$) δ 7.67 (d, *J* = 8.2 Hz, 4H), 7.37 (d, *J* = 8.2 Hz, 4H), 6.71 (s, 2H), 4.47 (t, *J* = 10.1 Hz, 2H), 4.40 (br s, 2H), 4.10-4.04 (m, 2H), 2.62 (br s, 2H), 2.25 (s, 2H), 1.41-1.35 (m, 2H), 1.31-1.27 (m, 2H), 1.01 (t, *J* = 7.2 Hz, 6H), 0.94 (d, *J* = 6.3 Hz,

6H), 0.88 (s, 9H); **^{13}C NMR** (150 MHz, $CDCl_3$) δ 187.4, 144.4, 126.4, 125.5, 125.2, 66.9, 54.6, 36.4, 34.5, 31.3, 26.9, 14.4, 12.3; **^{19}F NMR** (375 MHz, $CDCl_3$) δ –65.6 (s, 6F); (–164.9, C_6F_6); **HRMS** [+APCI] calculated for $C_{38}H_{43}N_4Cl_2F_6Ir$ 932.2393, found 932.2403 $[M\text{-}H_2O]^+$; **$[\alpha]^{20}_D$** +26.5 ° (*c* 0.1, $CHCl_3$).

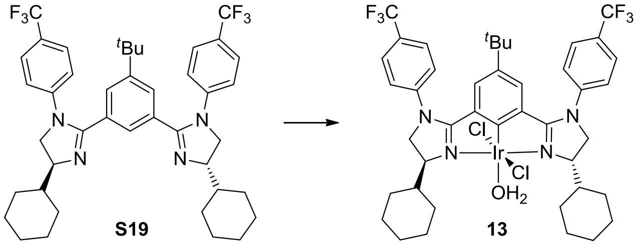

S19 13

[(*S*, *S*)-*t*-ButylPhebim-4-CF_3Ph-^{c}Hex]$IrCl_2(H_2O)$ (13). Prepared by general procedure **D** using ligand **S19** (139 mg, 0.19 mmol), $IrCl_3·3H_2O$ (75 mg, 0.21 mmol), and $NaHCO_3$ (18 mg, 0.21 mmol). The reaction was refluxed in ^{i}PrOH (6.5 mL) for 4.5 h. Preparative thin layer chromatography (7:3 hexanes:EtOAc) gave iridium(III) phebim **13** as a dark red solid (80 mg, 42 %); **R_f** 0.29 (7:3 hexanes:EtOAc); **mp** 216 ° C; **IR** (thin film, cm^{-1}) 3675, 2924, 2365, 1558, 1226, 781, 656; **^{1}H NMR** (600 MHz, $CDCl_3$) δ 7.67 (d, *J* = 8.5 Hz, 4H), 7.36 (d, *J* = 8.5 Hz, 4H), 6.71 (s, 2H), 4.49 (t, *J* = 10.2 Hz, 2H), 4.23-4.20 (m, 2H), 4.14-4.10 (m, 2H), 2.15 (td, *J* = 12.0, 2.4 Hz, 2H), 1.95 (d, *J* = 11.7 Hz, 2H), 1.80 (d, *J* = 12.9 Hz, 2H), 1.70-1.65 (m, 6H), 1.35 (q, *J* = 12.9 Hz, 2H), 1.27-1.22 (m, 4H), 1.14-1.02 (m, 4H), 0.88 (s, 9H); **^{13}C NMR** (150 MHz, $CDCl_3$) δ 171.4, 169.7, 144.4, 143.2, 132.1, 128.2 (q, $^2J_{C\text{-}F}$ = 33.0) 126.4, 125.4, 125.1, 123.1, 68.1, 60.6, 55.3, 39.7, 34.5, 31.3, 30.1, 26.7 (dd, $^1J_{C\text{-}F}$ = 178, $^2J_{C\text{-}F}$, 33.0), 21.2, 18.7, 14.4; **^{19}F NMR** (375 MHz, $CDCl_3$) δ –65.5 (s, 6F); (–164.9, C_6F_6); **HRMS** [+APCI] calculated for $C_{42}H_{47}N_4Cl_2F_6Ir$ 984.2706, found 984.2724 $[M\text{-}H_2O]^+$; **$[\alpha]^{20}_D$** + 28.4 ° (*c* 0.1, $CHCl_3$).

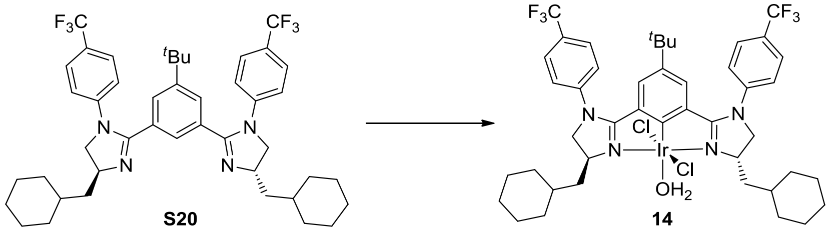

S20 14

[(*S*, *S*)-*t*-ButylPhebim-4-CF_3Ph-CH_2^{c}Hex]$IrCl_2(H_2O)$ (14). Prepared by general procedure D using ligand **S20** (595 mg, 0.79 mmol), $IrCl_3·3H_2O$ (314 mg, 0.89 mmol), and $NaHCO_3$ (75 mg, 0.89 mmol). The reaction was refluxed in ^{i}PrOH (26 mL) for 4.5 h. Flash chromatography (7:3 hexanes:EtOAc) afforded iridium(III) phebim **14** as a light orange solid (428 mg, 52 %); **R_f** 0.63 (7:3 hexanes:EtOAc); **mp** 236 ° C; **IR** (thin film, cm^{-1}) 3019, 2347, 2039, 1710, 1214, 745, 667; **^{1}H NMR** (600 MHz, $CDCl_3$) δ 7.68 (d, *J* = 8.4 Hz, 4H), 7.40 (d, *J* = 8.4 Hz, 4H), 6.70 (s, 2H), 4.56 (t, *J* = 9.7 Hz, 2H), 4.42-4.37 (m, 2H), 4.06 (dd, *J* = 9.0, 7.0 Hz, 2H), 2.20 (ddd, *J* = 13.6, 10.3, 2.7 Hz, 2H), 1.95 (br s, 2H), 1.87 (d, *J* = 12.8 Hz, 2H), 1.77-1.67 (m, 8H), 1.40-1.34 (m, 2H), 1.25-1.13 (m, 8H), 1.12-1.00 (m, 4H), 0.88 (s, 9H); **^{13}C NMR** (150 MHz, $CDCl_3$) δ 187.1, 169.6, 144.5, 143.1, 132.2, 126.5, 125.5, 125.4, 124.9, 123.1, 61.9, 60.2, 42.6, 35.4, 34.7, 34.6, 32.7, 31.4, 26.6, 26.5; **^{19}F NMR** (375 MHz, $CDCl_3$) δ –65.6 (s, 6F); (–164.9, C_6F_6); **HRMS** [+APCI] calculated for $C_{44}H_{51}N_4Cl_2F_6Ir$ 1012.3019, found 1012.3025 $[M\text{-}H_2O]^+$; **$[\alpha]^{20}_D$** +24.2 ° (*c* 0.1, $CHCl_3$).

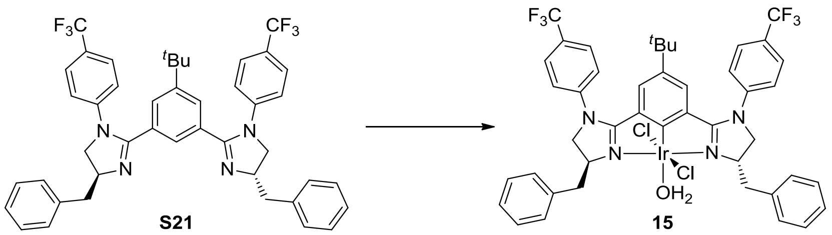

[(*S*, *S*)-*t*-ButylPhebim-p-CF_3Ph-Bn]$IrCl_2(H_2O)$ (15). Prepared by general procedure **D** using ligand **S21** (140 mg, 0.19 mmol), $IrCl_3 \cdot 3H_2O$ (78 mg, 0.22 mmol), and $NaHCO_3$ (18 mg, 0.22 mmol). The reaction was refluxed in ^{i}PrOH (6 mL) for 6 h. Preparative thin layer chromatography (7:3 hexanes:EtOAc) afforded iridium(III) phebim **15** as an orange solid (104 mg, 54 %); $\mathbf{R_f}$ 0.41 (7:3 hexanes:EtOAc); **mp** 180 ° C; **IR** (thin film, cm^{-1}) 3338, 2962, 2231, 2046, 1525, 1452, 1321, 1125, 1068; **^{1}H NMR** (400 MHz, $CDCl_3$) δ 7.69 (d, *J* = 8.6 Hz, 4H), 7.39 (d, *J* = 8.2 Hz, 4H), 7.35-7.32 (m, 4H), 7.29-7.23 (m, 6H), 6.71 (s, 2H), 4.63 (tdd, *J* = 7.1, 3.9 Hz, 2H), 4.28 (t, *J* = 10.1 Hz, 2H), 4.04 (dd, *J* = 9.9, 7.2 Hz, 2H), 3.68 (dd, *J* = 14.0, 3.9 Hz, 2H), 2.96 (dd, *J* = 14.0, 10.5 Hz, 2H), 0.84 (s, 9H); **^{13}C NMR** (100 MHz, $CDCl_3$) δ 174.2, 172.2, 146.2, 136.5, 131.9, 129.3, 128.9, 126.9, 126.5, 126.4, 126.3, 126.2, 65.8, 58.7, 40.9, 34.6, 30.9; **^{19}F NMR** (375 MHz, $CDCl_3$) δ –65.7 (s, 6F); (–164.9, C_6F_6); **HMRS** (+APCI) calculated for $C_{44}H_{38}ClF_6N_4Ir$ 964.2313, found 964.2306 $[M-H_2O-Cl-H]^+$; $[\alpha]^{20}_D$ + 20.5 ° (*c* 0.1, $CHCl_3$).

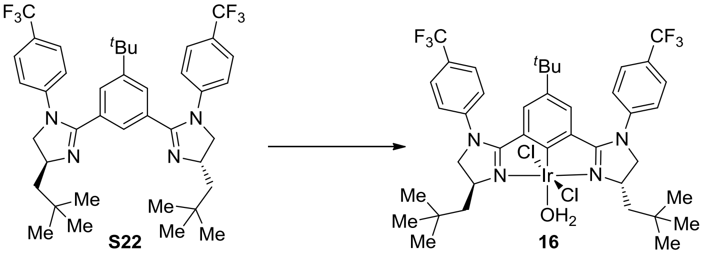

[(*S*, *S*)-*t*-ButylPhebim-p-CF_3Ph-$CH_2{}^t$Bu]$IrCl_2(H_2O)$ (16). Prepared by general procedure **D** using ligand **S22** (99 mg, 0.14 mmol, 1.0 equiv), $IrCl_3 \cdot 3H_2O$ (60 mg, 0.17 mmol, 1.2 equiv), and $NaHCO_3$ (15 mg, 0.17 mmol, 1.2 equiv). The reaction was refluxed in ^{i}PrOH (5 mL) for 5 h. Preparative thin layer chromatography (8:2 hexanes:acetone) afforded iridium(III) phebim **16** as a red solid (43 mg, 31% yield); $\mathbf{R_f}$ 0.20 (8:2 hexanes:acetone); **mp** $\geq$ 250 ° C; **IR** (neat, cm^{-1}) 2955, 2835, 1613, 1558, 1474, 1321, 1291, 1124, 1068, 846, 599; **^{1}H NMR** (400 MHz, $CDCl_3$) δ 7.68 (d, *J* = 8.0 Hz, 4H), 7.41 (d, *J* = 8.0 Hz, 4H), 6.69 (s, 2H), 4.63 (t, *J* = 10.0 Hz, 2H), 4.34 (q, *J* = 10.0 Hz, 2H), 4.04 (t, *J* = 10.0 Hz, 2H), 2.86 (s, 4H), 2.46 (d, *J* = 16 Hz, 2H), 1.67 (dd, *J* = 10.0, 16.0 Hz, 2H), 1.04 (s, 18H), 0.87 (s, 9H), **^{13}C NMR** (100 MHz, $CDCl_3$) δ 169.6, 144.3, 142.6, 132.1, 127.9 (q, *J* = 33.0 Hz), 126.2 (q, *J* = 4.0 Hz), 125.3, 125.2, 122.4, 62.0, 60.8, 48.7, 34.3, 31.2, 31.1, 30.0, 30.2; **^{19}F NMR** (375 MHz, $CDCl_3$) δ –65.6 (s, 6F); (–164.9, C_6F_6); **HRMS** (+NSI) calculated for $C_{40}H_{47}Cl_2F_6N_4Ir$ 960.2706, found 960.2725 $[M–H_2O]^+$; $[\alpha]^{20}_D$ + 55.3 ° (c 0.4, $CHCl_3$).

b) Diazoacetate Synthesis

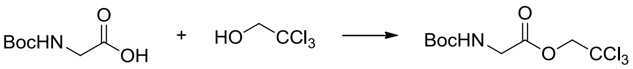

S23

2,2,2-trichloroethyl 2-((tert-butoxycarbonyl)amino)acetate (S23). Prepared via the method of Veri.[6] *N*-(*t*-butoxycarbonyl)glycine (3.3 g, 18.7 mmol) and 2,2,2-trichloroethanol (2.9 mL, 30.1 mmol) were stirred at 0 ºC in CH_2Cl_2 (60 mL). DMAP (0.49 g, 4.0 mmol) and *N*,*N*'-dicyclohexylcarbodiimide (4.9 g, 23.6 mmol) were added and the resulting mixture was stirred at 0 ºC for 3 h and then 23 ºC overnight. The resulting solution was filtered and the filtrate was concentrated. The concentrate was then taken up in 50 mL each of EtOAc and water. The organic phase was then washed with two 30 mL portions of saturated $NaHCO_3$(aq) and brine. Following concentration, the residue was purified by flash chromatography (8:2 hexanes:EtOAc) to yield a white crystalline solid (2.04 g, 36 % yield); $\mathbf{R_f}$ 0.65 (EtOAc); ^{1}**H NMR** (400 MHz, $CDCl_3$) δ 5.02 (s, 1H), 4.79 (s, 2H), 4.07 (d, *J* = 6.0 Hz, 2H), 1.46 (s, 9H). All spectral data matched those previously reported.[6,7]

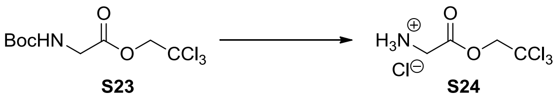

S23 **S24**

2-oxo-2-(2,2,2-trichloroethoxy)ethanaminium chloride (S24). A 4 M solution of HCl in EtOAc was cooled to 0 ºC in a 50 mL round bottom flask equipped with a stir bar. **S23** (0.91 g, 3.0 mmol) was added in one portion and stirred at 0 ºC for 90 min at which time the reaction was judged to be complete by TLC. The mixture was concentrated *in vacuo*, and the resulting solid was washed with dry Et_2O to yield a white crystalline solid in quantitative yield; $\mathbf{R_f}$ 0.55 (8:2 hexanes:EtOAc); ^{1}**H NMR** (400 MHz, D_2O) δ 5.00 (s, 2H), 4.10 (s, 2H). All spectral data matched those previously reported.[6]

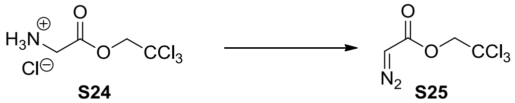

S24 **S25**

2,2,2-trichloroethyl 2-diazoacetate (S25). A 100 mL round bottom flask equipped with a stir bar was charged with **S24** (0.31 g, 1.3 mmol), CH_2Cl_2 (15 mL) and distilled water. The mixture was stirred and cooled to 0 ºC. $NaNO_2$ (0.45 g, 6.6 mmol) was added, followed by 10% H_2SO_4 (aq) (3 mL). The mixture was stirred at 0 ºC for 2 h at which time the reaction was judged to be complete by TLC. The organic layer was separated and washed with saturated $NaHCO_3$ (aq), dried with Na_2SO_4 and concentrated *in vacuo*. The residue was purified by flash chromatography (7:1 pentane:Et_2O) to afford a yellow oil (65.1 mg, 24 % yield); $\mathbf{R_f}$ 0.64 (7:3 hexanes:EtOAc). **IR** (neat, cm^{-1}) 2116, 1703, 1385, 1349, 1218, 1148, 1045, 796, 715, 572; ^{1}**H NMR** (400 MHz, $CDCl_3$) δ 4.79 (s, 2H), 4.90 (br s, 1H); ^{13}**C NMR** (100 MHz, $CDCl_3$) δ 164.5, 95.0, 73.6, 46.6; **HRMS** (+NSI) calculated for $C_4H_3Cl_3N_2O_2Na$ 238.9158, found 238.9164 $[M+Na]^+$.

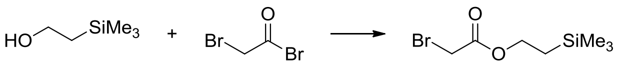

S26

2-(trimethylsilyl)ethyl 2-bromoacetate (S26). Prepared via the method of Caprioli and coworkers.[8] A 100 mL round bottom flask equipped with a stir bar was flame dried and placed under N_2 atmosphere. The flask was charged with 2-(trimethylsilyl)ethanol (1.7 mL, 11.5 mmol), pyridine (0.88 mL, 10.9 mmol), DMAP (124 mg, 1.0 mmol) and CH_2Cl_2 (20 mL). The mixture was stirred and cooled to 0 ºC. Bromoacetyl bromide (0.97 mL, 11.1 mmol) was then added dropwise. The ice bath was removed and the resulting solution was allowed to stir for 2 h at 23 ºC. The reaction was washed with two portions of 1 M HCl (aq) (15 mL each) followed by brine (15 mL). The organic layer was then dried with Na_2SO_4 and concentrated *in vacuo* to afford a brown oil (1.8 g, 68 % yield); **R$_f$** 0.68 (8:2 hexanes:Et_2O); **^{1}H NMR** (400 MHz, $CDCl_3$) δ 4.29-4.24 (m, 2H), 3.81 (s, 2H), 1.06-1.01 (m, 2H), 0.05 (s, 9H). All spectral data matched those previously reported.[8]

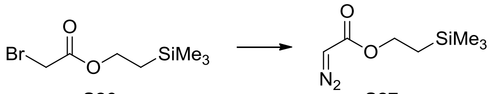

S26 S27

2-(trimethylsilyl)ethyl 2-diazoacetate (S27). A 100 mL round bottom flask equipped with a stir bar was flame dried and placed under N_2 atmosphere. The flask was then charged with **S26** (1.3 g, 5.4 mmol), THF (25 mL) and *N,N*'-ditosylhydrazine[9] (3.5 g, 10.2 mmol). The mixture was stirred and cooled to 0 ºC. DBU (3.7 mL, 24.7 mmol) was then added dropwise over 15 min. After stirring at 0 ºC for another 20 min, starting material was judged to be completely consumed by TLC. Saturated $NaHCO_3$ (aq) (25 mL) was added, followed by extraction with Et_2O. The organic layer was further washed with water (25 mL) and brine (25 mL), dried with $MgSO_4$, and concentrated *in vacuo*. The residue was purified by flash chromatography (8:2 hexanes:Et_2O) to yield a yellow oil (0.5 g, 50 % yield); **R$_f$** 0.7 (8:2 hexanes:Et_2O); **IR** (neat, cm^{-1}) 2106, 1695, 1390, 1351, 1248, 1175, 1043, 858, 836, 742; **^{1}H NMR** (400 MHz, $CDCl_3$) δ 4.69 (br s, 1H), 4.26-4.21 (m, 2H), 1.00-0.96 (m, 2H), 0.02 (s, 9H); **^{13}C NMR** (100 MHz, $CDCl_3$) δ -1.5, 17.5, 46.1, 63.1, 166.9; **HRMS** (+NSI) calculated for $C_7H_{14}N_2O_2SiNa$ 209.0722, found 209.0719 $[M+Na]^+$.

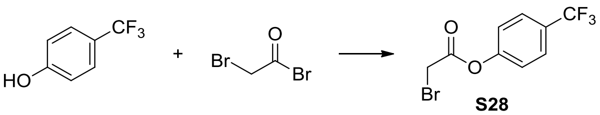

S28

4-(trifluoromethyl)phenyl 2-bromoacetate (S28). Prepared by the method of Candish and Lupton.[10] A 100 mL round bottom flask equipped with a stir bar was flame dried and placed under N_2 atmosphere. The flask was charged with CH_3CN (25 mL), pyridine (0.85 mL, 10.5 mmol, 2.1 equiv) and 4-(trifluoromethyl)phenol (800 mg, 4.9 mmol, 1.0 equiv). The mixture was then stirred and cooled to 0 ºC. Bromoacetyl bromide (0.68 mL, 7.8 mmol, 1.6 equiv) was then added dropwise. The resulting solution was allowed to stir 15 min at 0 ºC and then water (12 mL) was added. The mixture was extracted with CH_2Cl_2, dried with Na_2SO_4, concentrated *in vacuo*, and purified by flash chromatography (95:5 hexanes:EtOAc) to afford a slightly yellow oil (1.0 g, 72 % yield); **R$_f$** 0.57 (9:1 hexanes:EtOAc); **IR** (neat, cm^{-1}) 1766, 1611, 1320, 1259, 1203, 1101, 1062, 1017, 928, 853, 689, 586; **^{1}H NMR** (400 MHz, $CDCl_3$) δ 7.67 (d,

J = 12 Hz, 2H), 7.26 (d, J = 12 Hz, 2H); 4.07 (s, 2H); ^{13}**C NMR** (100 MHz, $CDCl_3$) δ 165.3, 152.7, 129.6 (q, J = 33 Hz), 126.9 (q, J = 4 Hz), 125.0, 121.6, 25.2; ^{19}**F NMR** (375 MHz, $CDCl_3$) δ –65.5 (s, 3F), (–164.9, C_6F_6).

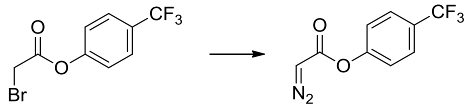

S28 S29

4-(trifluoromethyl)phenyl 2-diazoacetate (S29). A 100 mL round bottom flask equipped with a stir bar was flame dried and placed under N_2 atmosphere. The flask was charged with THF (25 mL), **S28** (1.1 g, 3.9 mmol) and *N,N'*-ditosylhydrazine[9] (3.4 g, 10.0 mmol), stirred and cooled to 0 ºC. DBU (3.7 mL, 24.7 mmol) was added dropwise over 15 min and the reaction was permitted to stir at 0 ºC for another 15 min once addition was complete. Saturated $NaHCO_3$ (aq) (25 mL) was added, and the resulting mixture was extracted with Et_2O, washed with brine, and dried with Na_2SO_4. Concentration *in vacuo* and purification by flash chromatography (hexanes → 95:5 hexanes:EtOAc) afforded a yellow oil (239 mg, 27 % yield); $\mathbf{R_f}$ 0.21 (9:1 hexanes:EtOAc); **IR** (neat, cm^{-1}) 3128, 2116, 1703, 1614, 1513, 1368, 1320, 1117, 1101, 1061, 1016, 725; ^{1}**H NMR** (400 MHz, $CDCl_3$) δ 7.65 (d, J = 8.0 Hz, 2H), 7.26 (d, J = 8.0 Hz, 2H), 5.04 (br s, 1H), ^{13}**C NMR** (100 MHz, $CDCl_3$) δ 152.9, 127.8, (q, J = 30.0 Hz), 126.7, (q, J = 4.0 Hz), 125.2, 122.5, 122.0, 47.0; ^{19}**F NMR** (375 MHz, $CDCl_3$) δ –65.4(s, 3F), (–164.9, C_6F_6); **HRMS** (+NSI) calculated for $C_9H_5N_2O_2F_3Na$ 253.0195, found 253.0198 $[M+Na]^+$.

c) Substrate Synthesis

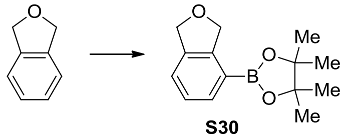

S30

2-(1,3-dihydroisobenzofuran-4-yl)-4,4,5,5-tetramethyl-1,3,2-dioxaborolane (S30). An oven-dried flask equipped with a reflux condenser was charged with phthalan (2.4 mL, 21.9 mmol, 1 equiv), $[Ir(OMe)(cod)]_2$ (39 mg, 0.06 mmol, 0.25 mol %), 3,4,7,8-tetramethyl-1,10-phenanthroline (62 mg, 0.26 mmol, 1 mol %), and bis(pinacolato)diboron (4.2 g, 16.7 mmol, 0.75 equiv) in tetrahydrofuran (108 mL). The mixture was heated to reflux for 22 h with vigorous stirring. The reaction mixture was cooled to room temperature and filtered through silica gel. The filtrate was concentrated *in vacuo* and flash chromatography (95:5 hexanes:EtOAc) afforded the title compound as a white solid (1.7 g, 32 %); **R_f** 0.55 (9:1 hexanes/EtOAc); **mp** 74-76 ° C; **IR** (thin film, cm^{-1}) 2978, 2857, 1603, 1350, 1129, 1047, 967; **^{1}H NMR** (400 MHz, $CDCl_3$) δ 7.68 (d, *J* = 7.0 Hz, 1H), 7.31 (d, *J* = 7.4 Hz, 1H), 7.27-7.23 (m, 1H), 5.23 (t, *J* = 2.0 Hz, 2H), 5.11-5.09 (m, 2H), 1.31 (s, 12H); **^{13}C NMR** (100 MHz, $CDCl_3$) δ 146.1, 138.2, 133.9, 126.5, 123.6, 83.8, 74.8, 73.2, 24.9; **HRMS** [+NSI] calculated for $C_{14}H_{19}O_3BNa$ 269.1319, found 269.1319 $[M+Na]^+$.

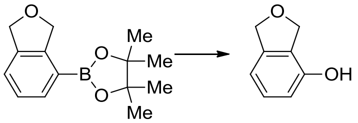

S30 S31

1,3-dihydroisobenzofuran-4-ol (S31). H_2O_2 (5 ml, 30 wt. % in H_2O) was added to a solution of **S30** (1.7 g, 6.9 mmol, 1 equiv) in H_2O (5 mL) at room temperature. The reaction was stirred until thin layer chromatography indicated complete consumption of starting material (1 h). The mixture was transferred to a separatory funnel and the aqueous layer was extracted with CH_2Cl_2 (3 x). The combined organic extracts were washed with brine, dried over $MgSO_4$ and concentrated *in vacuo*. Flash chromatography (7:3 hexanes:EtOAc) afforded the title compound as a white solid (518 mg, 55 %); **^{1}H NMR** (400 MHz, $CDCl_3$) δ 7.14-7.11 (m, 2H), 6.8 (d, *J* = 7.6 Hz, 1H), 6.63 (d, *J* = 8.2 Hz, 1H), 5.11 (d, *J* = 2.9 Hz, 4H). All spectral data matched those previously reported.[11]

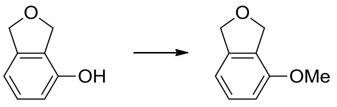

S31 S32

4-methoxy-1,3-dihydroisobenzofuran (S32). An oven-dried round bottom flask was charged with **S31** (518 mg, 3.8 mmol, 1 equiv) and K_2CO_3 (1.06 g, 7.7 mmol, 2 equiv) in MeCN (15 mL). MeI (390 µL, 6.3 mmol, 1.6 equiv) was added via syringe. The reaction mixture was heated to 35 ° C. Once thin layer chromatography indicated complete consumption of starting material (24 h), the reaction was quenched by the addition of NH_4Cl (aq). The biphasic mixture was transferred to a separatory funnel and the aqueous layer was extracted with CH_2Cl_2 (3 x). The combined organic extracts were washed with brine, dried over $MgSO_4$ and concentrated *in*

vacuo. Flash chromatography (8:2 pentane:Et_2O) afforded the title compound as a white solid (484 mg, 85 %); **^{1}H NMR** (400 MHz, $CDCl_3$) δ 7.24-7.21 (m, 1H), 6.81 (d, *J* = 7.5 Hz, 1H), 6.72 (d, *J* = 8.3 Hz, 1H), 5.09 (s, 4H), 3.82-3.81 (m, 3H). All spectral data matched those previously reported.[12]

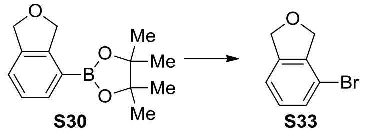

S30 S33

4-bromo-1,3-dihydroisobenzofuran (S33). Prepared according to literature procedure[13] using **S30** (740 mg, 3.0 mmol), $CuBr_2$ (2 g, 9.0 mmol), MeOH (38 mL), and H_2O (38 mL). After heating for 6 h, flash chromatography (95:5 hexanes:EtOAc) afforded the title compound as a white amorphous solid (275 mg, 46 % yield); **R_f** 0.65 (9:1 hexanes:EtOAc); **IR** (thin film, cm^{-1}) 2852, 1720, 1574, 1447, 1128, 1045, 881; **^{1}H NMR** (400 MHz, $CDCl_3$) δ 7.37-7.34 (m, 1H), 7.15-7.08 (m, 2H), 5.15 (d, *J* = 7.8 Hz, 2H), 5.06 (d, *J* = 7.8 Hz, 2H) 4.18 (t, *J* = 7.2 Hz, 2H); **^{13}C NMR** (100 MHz, $CDCl_3$) δ 141.0, 139.8, 130.2, 129.2, 119.7, 115.9, 74.7, 74.6; **HRMS** [+APCI] calculated for C_8H_8OBr 198.9753, found 198.9752 $[M+H]^+$.

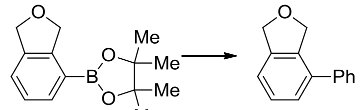

S30 S34

4-phenyl-1,3-dihydroisobenzofuran (S34). Prepared according to literature procedure[14] using **S30** (50 mg, 0.20 mmol, 1 equiv), $Pd(PPh_3)_4$ (12 mg, 5 mol %), K_2CO_3 (56 mg, 0.41 mmol, 2 equiv), and PhI (50 μL, 0.45 mmol, 2 equiv) in DMF (0.6 mL). After heating for 19 h, flash chromatography (9:1 hexanes:EtOAc) afforded the title compound as a white solid (34 mg, 85 % yield); **^{1}H NMR** (400 MHz, $CDCl_3$) δ 7.45-7.30 (m, 7H), 7.22 (d, *J* = 7.4 Hz, 1H), 5.19 (d, *J* = 2.0 Hz, 2H), 5.17 (d, *J* = 2.0 Hz, 2H). All spectral data matched those previously reported.[12]

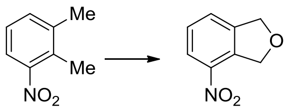

S35

4-nitro-1,3-dihydroisobenzofuran (S35). Adapting a procedure by Favor and coworkers,[15] a 250 mL round bottom flask equipped with a stir bar and condenser was charged with 1,2-dimethyl-3-nitrobenzene (7.9 mL, 59 mmol, 1.0 equiv) followed by *N*-bromosuccinimide (24 g, 135 mmol, 2.3 equiv) and CCl_4 (36 mL). The yellow suspension was stirred and benzoyl peroxide (150 mg, 0.01 equiv) was added. The suspension was stirred at reflux for 2 h, at which time another 500 mg (0.04 equiv) of benzoyl peroxide was added. The reaction was heated at reflux for another 2 h at which time it was cooled to room temperature, filtered to remove a white solid which was washed with additional CCl_4, and concentrated *in vacuo* to afford a yellow oil. The oil was immediately transferred to a clean 250 mL round bottom flask equipped with a condenser and stir bar. $PhCH_3$ (100 mL) was added, followed by neutral Al_2O_3 (75 g, Brockmann Activity I grade), and H_2O (5 mL) with vigorous stirring. The yellow suspension was heated to an external temperature of 120 ° C for 40 h. The reaction was cooled to room temperature, filtered, and concentrated *in vacuo* to afford a yellow oil. Flash chromatography on

silica gel (95:5 → 93:7 hexanes:EtOAc) yielded a yellow powder (2.4 g, 25% yield over 2 steps); **R$_f$** 0.21 (9:1 hexanes:EtOAc); **mp** 106-108 ° C; **IR** (neat, cm^{-1}) 2916, 2863, 1518, 1338, 1045, 904, 818, 732; **^{1}H NMR** (400 MHz, $CDCl_3$) δ 8.14 (d, *J* = 8.0 Hz, 1H), 7.56 (d, *J* = 8.0 Hz, 1H), 7.49 (t, *J* = 8.0 Hz, 1H), 5.53 (s, 2H), 5.20 (s, 2H); **^{13}C NMR** (100 MHz, $CDCl_3$) δ 143.3, 143.1, 136.3, 129.0, 127.0, 122.7, 74.8, 73.2; **HRMS** (+APCI) calculated for $C_8H_8O_3N$ 166.0499, found 166.0498 $[M+H]^+$.

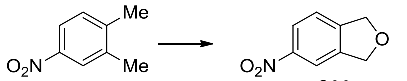

S36

5-nitro-1,3-dihydroisobenzofuran (S36). Adapting a procedure by Favor and coworkers,[15] a 250 mL round bottom flask equipped with a stir bar and condenser was charged with 1,2-dimethyl-4-nitrobenzene (8.0 g, 53 mmol, 1.0 equiv) followed by *N*-bromosuccinimide (24.6 g, 138 mmol, 2.6 equiv) and CCl_4 (40 mL). The yellow suspension was stirred and benzoyl peroxide (120 mg, 0.01 equiv) was added. The suspension was stirred at reflux for 2 h, at which time another 500 mg (0.04 equiv) of benzoyl peroxide was added. The reaction was heated at reflux for another 2 h at which time it was cooled to room temperature, filtered to remove a white solid which was washed with additional CCl_4, and concentrated *in vacuo* to afford a yellow oil. The oil was immediately transferred to a clean 250 mL round bottom flask equipped with a condenser and stir bar. $PhCH_3$ (100 mL) was added, followed by neutral Al_2O_3 (150 g, Brockmann Activity I grade), and H_2O (5 mL) with vigorous stirring. The now unstirrable yellow slurry was heated to an external temperature of 120 ° C for 4 h. The reaction was cooled to room temperature, filtered, and concentrated *in vacuo* to afford a yellow oil. Flash chromatography on silica gel (95:5 to 9:1 hexanes:EtOAc) yielded a yellow powder (2.04 g, 23% yield over 2 steps). **R$_f$** 0.24 (90:10 hexanes:EtOAc); **mp** 92-95 ºC; **IR** (neat, cm^{-1}) 3105, 3036, 2859, 1519, 1343, 1321, 1043, 898, 736; **^{1}H NMR** (400 MHz, $CDCl_3$) δ 8.18 (dd, *J* = 2.0, 8.4 Hz, 1H), 8.11 (d, *J* = 1.6 Hz 1H), 7.39 (d, *J* = 8.0 Hz, 1H), 5.18 (s, 4H); **^{13}C NMR** (100 MHz, $CDCl_3$) δ 148.0, 146.4, 141.0, 123.3, 121.7, 116.6, 73.2, 73.0; **HRMS** (+APCI) calculated for $C_8H_8O_3N$ 166.0499, found 166.0498 $[M+H]^+$.

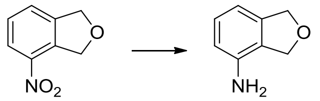

S35 **S37**

1,3-dihydroisobenzofuran-4-amine (S37). Adpating a procedure by Favor a coworkers,[15] a 100 mL round bottom flask was equipped with a stir bar and charged with 4-nitro-1,3-dihydroisobenzofuran **S35** (2.4 g, 14.8 mmol) followed by THF (50 mL). The yellow suspension was vigorously stirred and 2 disposable glass pipettes' volumes of Raney® Ni (2800, slurry in H_2O) were added. The suspension was vigorously stirred at 23 ºC for 19 h under a balloon of H_2. Filtration through Celite followed by concentration *in vacuo* afforded a yellow powder (1.8 g, 89% yield); **R$_f$** 0.16 (8:2 hexanes:EtOAc); **mp** 86-88 ° C; **IR** (neat, cm^{-1}) 3375, 3216, 3039, 2853, 1601, 1488, 1305, 1032, 995, 894, 757; **^{1}H NMR** (400 MHz, $CDCl_3$) δ 7.10 (t, *J* = 8.0 Hz, 1H), 6.67 (dd, *J* = 0.8, 8.0 Hz, 1H), 6.57 (dd, *J* = 0.8, 8.0 Hz, 1H), 5.12-5.10 (m, 2H), 5.04-5.02 (m, 2H), 3.54 (s, 2H); **^{13}C NMR** (100 MHz, $CDCl_3$) δ 140.3, 140.2, 128.7, 123.8, 113.4, 110.8, 74.2, 71.7; **HRMS** (+NSI) calculated for $C_8H_{10}ON$ 136.0757, found 136.0756 $[M+H]^+$.

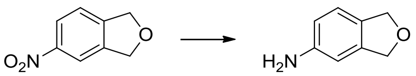

S36 S38

1,3-dihydroisobenzofuran-5-amine (S38). Adapting a procedure by Favor and coworkers,[15] a 100 mL round bottom flask equipped with a stir bar and charged with 5-nitro-1,3-dihydroisobenzofuran **S36** (2.0 g, 12.2 mmol) followed by THF (40 mL). The yellow suspension was vigorously stirred and 2 disposable glass pipettes' volumes of Raney® Ni (2800, slurry in H_2O) were added. The suspension was vigorously stirred at 23 ° C for 24 h under a balloon of H_2. Filtration through Celite followed by concentration afforded a yellow powder (1.6 g, 11.9 mmol, 98% yield); **R_f** 0.16 (8:2 hexanes:EtOAc); **mp** 104-106 ° C; **IR** (neat, cm^{-1}) 3330, 3233, 3021, 2850, 1607, 1491, 1367, 1295, 1026, 818; **^{1}H NMR** (400 MHz, $CDCl_3$) δ 7.00 (d, *J* = 8.0 Hz, 1H), 6.60 (dd, *J* = 2.0 Hz, 8.0 Hz, 1H), 6.55 (d, *J* = 2.0 Hz, 1H), 5.01 (s, 4H), 3.70 (s, 2H), **^{13}C NMR** (100 MHz, $CDCl_3$) δ 145.9, 140.5, 128.9, 121.5, 114.4, 107.3, 73.4, 73.3; **HRMS** (+NSI) calculated for $C_8H_{10}ON$ 136.0757, found 136.0756$[M+H]^+$.

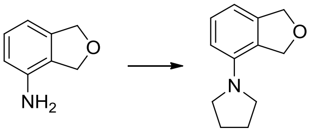

S37 S39

1-(1,3-dihydroisobenzofuran-4-yl)pyrrolidine (S39). Adapting a procedure by Xiong, Chen and coworkers,[16] a 25 mL round bottom flask equipped with a stir bar and reflux condenser was flame dried and placed under N_2 atmosphere. The flask was then charged with **S37** (399 mg, 3.0 mmol, 1.0 equiv), K_2CO (1.3 g, 9.4 mmol, 3 equiv), CH_3CN (10 mL), and 1,4-dibromobutane (0.4 mL, 3.4 mmol, 1.1 equiv). The reaction mixture was stirred and heated to a vigorous reflux for 22 h at which times another 0.1 mL of 1,4-dibromobutane was added. After 45 h total of vigorous reflux, the orange mixture was cooled to 23 ° C, filtered, and concentrated *in vacuo* to give a yellow oil. Flash chromatography on silica gel yielded a slightly yellow oil (305 mg, 55 % yield); **R_f** 0.44 (9:1 hexanes:EtOAc); **IR** (neat, cm^{-1}) 2963, 2842, 1593, 1482, 1458, 1359, 1048, 1024, 911, 758, 730, 703; **^{1}H NMR** (400 MHz, $CDCl_3$) δ 7.16 (t, *J* = 8.0 Hz, 1H), 6.60 (d, *J* = 8.0 Hz, 8.0 Hz, 1H), 6.46 (d, *J* = 8.0 Hz, 1H), 5.34 (m, 2H), 5.07 (m, 2H), 3.35 (m, 4H), 1.97 (m, 4H); **^{13}C NMR** (100 MHz, $CDCl_3$) δ 144.0, 140.8, 128.6, 123.1, 111.2, 109.1, 74.1, 73.7, 49.0, 25.3; **HRMS** (+NSI) calculated for $C_{12}H_{16}ON$ 190.1226, found 190.1227 $[M+H]^+$.

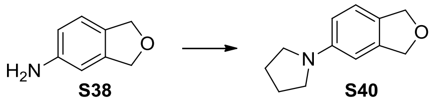

S38 S40

1-**(1,3-dihydroisobenzofuran-5-yl)pyrrolidine (S40)**. Adapting a procedure by Xiong, Chen and coworkers,[16] a 25 mL round bottom flask equipped with a stir bar and reflux condenser was flame dried and placed under N_2 atmosphere. The flask was then charged with **S38** (402 mg, 3.0 mmol, 1.0 equiv), K_2CO (1.3 g, 9.4 mmol, 3 equiv), CH_3CN (10 mL), and 1,4-dibromobutane (0.4 mL, 3.4 mmol, 1.1 equiv). The reaction mixture was stirred and heated to a vigorous reflux for 30 h at which time another 0.1 mL of 1,4-dibromobutane was added. After 45 h total of vigorous reflux, the orange mixture was cooled to 23 ° C, filtered, and concentrated *in vacuo* to an orange oil. Flash chromatography on silica gel yielded a light pink solid (241 mg, 43 % yield); **R_f** 0.44 (9:1 hexanes:EtOAc); **mp** 80-82 ° C; **IR** (neat, cm^{-1}) 2953, 2884, 2836, 1627,

1573, 1504, 1366, 1185, 1036, 892, 796; **^{1}H NMR** (400 MHz, $CDCl_3$) δ 7.10 (d, *J* = 8.0 Hz, 1H), 6.51 (dd, *J* = 2.0 Hz, 8.0 Hz, 1H), 6.45 (s, 1H), 5.10 (d, *J* = 2.0 Hz, 2H), 5.08 (d, *J* = 2.0 Hz, 2H), 3.30 (m, 4H), 2.03 (m, 4H); **^{13}C NMR** (100 MHz, $CDCl_3$) δ 147.7, 140.2, 125.5, 121.2, 111.0, 103.4, 73.6, 73.2, 47.8, 25.3; **HRMS** (+NSI) calculated for $C_{12}H_{16}ON$ 190.1226, found 190.1229 $[M+H]^+$.

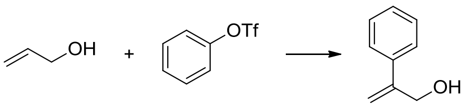

S41

2-phenylprop-2-en-1-ol (S41). Prepared by the method of Glorius and coworkers.[17] A 100 mL 2-necked round bottom flask equipped with a stir bar was flame dried and placed under N_2 atmosphere. The flask was charged with PhOTf (2.5 g, 9.4 mmol) followed by anhydrous DMF (15 mL), allyl alcohol (3.0 mL, 44.1 mmol), 1,3-bis(diphenylphosphino)propane (DPPP, 557 mg, 1.4 mmol) and $Pd(OAc)_2$ (215 mg, 0.96 mmol). DMF (15 mL) and Et_3N (4.6 mL, 33.0 mmol) were then added with stirring. The clear, light yellow solution was heated to 100 °C with stirring for 90 min and then cooled to 23 ºC. C_6H_6 and water were added to the mixture. The organic portion was extracted twice more with water and lastly washed with 5 wt % LiCl (aq) solution. The organic layer was then dried with Na_2SO_4, concentrated *in vacuo*, and purified by flash chromatography (9:1 → 7:3 hexanes:Et_2O) to yield a clear oil (541 mg, 43 % yield); **R_f** 0.38 (2:1 hexanes:Et_2O); **IR** (neat, cm^{-1}) 3331, 3062, 3055, 3010, 1686, 1631, 1599, 1573, 1494, 1044, 1023, 901, 778, 703; **^{1}H NMR** (400 MHz, $CDCl_3$) δ 7.46-7.45 (m, 2H), 7.38-7.35 (m, 2H), 7.32-7.30 (m, 1H), 5.48 (d, *J* = 0.8 Hz, 1H), 5.38 (d, *J* = 0.8 Hz, 1H), 4.55 (s, 2H); **^{13}C NMR** (100 MHz, $CDCl_3$) δ 147.2, 138.4, 128.5, 127.9, 126.1, 112.6, 65.0; **HRMS** (+NSI) calculated for $C_9H_{11}O$ 135.0810, found 135.0805$[M+H]^+$.

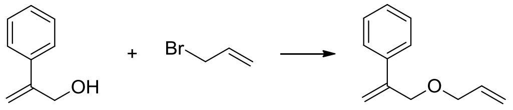

S41 **S42**

(3-(allyloxy)prop-1-en-2-yl)benzene (S42). Prepared by a procedure adapted from Mioskowski and coworkers.[18] A 100 mL round bottom flask was equipped with a stir bar, flame dried and placed under N_2 atmosphere. The flask was charged with NaH (60% in oil, 209 mg, 5.2 mmol) and anhydrous THF (5 mL). The flask was then cooled to 0 ºC and a solution of **S41** (526 mg, 3.9 mmol) in THF (5 mL) was added dropwise with stirring. After addition was complete, the flask was allowed to come to 23 ºC and stirred for an additional 1 h. The flask was cooled to 0 ºC again and a solution of allyl bromide (1.0 mL, 11.6 mmol) in THF (5 mL) was added dropwise. The flask was then stirred at 23 ºC overnight to yield a cloudy white mixture. The flask was cooled to 0 ºC and saturated NH_4Cl (aq) was added slowly. The mixture was extracted with Et_2O, and the organic portion was dried with Na_2SO_4, concentrated *in vacuo* and purified by flash chromatography (100 % hexanes) to afford a slightly yellow oil (530 mg, 77 % yield); **R_f** 0.9 (9:1 hexanes:CH_2Cl_2); **^{1}H NMR** (400 MHz, $CDCl_3$) δ 7.49-7.47 (m, 2H), 7.37-7.34 (m, 2H), 7.31-7.28 (m, 1H), 5.95 (ddt, *J* = 5.6, 10.3, 17.2 Hz, 1H), 5.54 (d, *J* = 0.6 Hz, 1H), 5.36 (d, *J* = 0.6 Hz, 1H), 5.38-5.17 (m, 2H), 4.39 (s, 2H), 4.06 (dt, *J* = 5.6, 1.3 Hz, 2H). All spectral data matched those previously reported.[18]

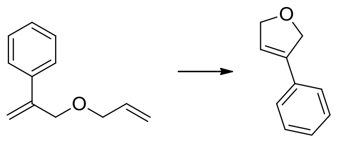

S42 S43

3-phenyl-2,5-dihydrofuran (S43). Adapting a procedure by Mioskowski and coworkers,[18] a 250 mL round bottom flask equipped with a stir bar was flame dried and placed under N_2 atmosphere. The flask was charged with **S42** (601 mg, 3.45 mmol), CH_2Cl_2 (170 mL), and Grubbs' II catalyst (142 mg, 0.17 mmol) to afford a clear, maroon solution. The solution was then heated to reflux for 6 h. The light brown solution was then cooled to 23 ºC, concentrated *in vacuo*, and then immediately purified by flash chromatography (95:5 → 9:1 hexanes:Et_2O) to afford a white crystalline solid (451 mg, 89 % yield); R_f 0.25 (9:1 hexanes:Et_2O); **^{1}H NMR** (400 MHz, $CDCl_3$) δ 7.36-7.28 (m, 5H), 6.24 (qn, *J* = 2.1 Hz, 1H), 5.02 (td, *J* = 4.8, 2.4 Hz, 2H), 4.86 (td, *J* = 4.8, 2.4 Hz, 2H). All spectral data matched those previously reported.[18]

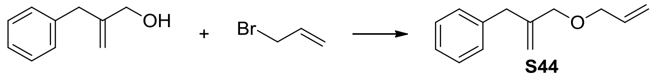

S44

(2-((allyloxy)methyl)allyl)benzene (S44). A 100 mL round bottom flask was equipped with a stir bar, flame dried and placed under N_2 atmosphere. The flask was charged with NaH (60% in oil, 502 mg, 12.6 mmol) and anhydrous THF (12 mL). The flask was then cooled to 0 ºC and a solution of 2-benzylprop-2-en-1-ol[19] (1.2 g, 8.2 mmol) in THF (12 mL) was added dropwise with stirring. After addition was complete, the flask was allowed to come to 23 ºC and stirred for an additional 1 h. The flask was cooled to 0 ºC again and a solution of allyl bromide (2.4 mL, 27.3 mmol) in THF (12 mL) was added dropwise. The flask was then stirred at 23 º C overnight to yield a cloudy white mixture. The flask was cooled to 0 º C and saturated NH_4Cl (aq) was added slowly. The mixture was extracted with Et_2O, and the organic portion was dried with Na_2SO_4, concentrated *in vacuo* and purified by flash chromatography (hexanes → 3:1 hexanes:CH_2Cl_2) to afford a clear oil (959 mg, 62 % yield); **R_f** 0.45 (1:1 hexanes:CH_2Cl_2); **IR** (neat, cm^{-1}) 3081, 3027, 2847, 1649, 1494, 1452, 1084, 905, 740, 698; **^{1}H NMR** (400 MHz, $CDCl_3$) δ 7.32-7.27 (m, 2H), 7.23-7.19 (m, 3H), 5.91 (ddt, *J* = 6, 10, 17.8 Hz, 1H), 5.28 (dq, *J* = 1.6, 17.2 Hz 1H), 5.18 (m, 1H), 5.11 (m, 1H), 4.92 (m, 1H), 3.94 (dt, *J* = 1.6, 5.6 Hz, 2H), 3.87 (m, 2H), 3.40, (s, 2H); **^{13}C NMR** (100 MHz, $CDCl_3$) δ 145.5, 139.1, 134.7, 129.0, 128.3, 126.1, 116.9, 113.3, 72.2, 70.9, 39.9; **HRMS** (+APCI) calculated for $C_{13}H_{17}O$ 189.1279, found 189.1273 $[M+H]^+$.

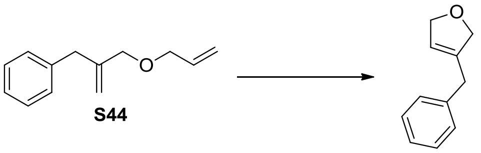

S44 S45

3-benzyl-2,5-dihydrofuran (S45). Adapting a procedure by Mioskowski and coworkers,[18] a 250 mL round bottom flask equipped with a stir bar was flame dried and placed under N_2 atmosphere. The flask was charged with **S44** (600 mg, 3.2 mmol), CH_2Cl_2 (150 mL), and Grubbs' II catalyst (113 mg, 0.13 mmol) to afford a clear, maroon solution. The solution was then heated to reflux for 6 h. The light brown solution was then cooled to 23 º C, concentrated *in vacuo*, and then immediately purified by flash chromatography (95:5 → 9:1 hexanes:Et_2O) to

afford a clear oil (438 mg, 2.7 mmol, 86 % yield); **R$_f$** 0.31 (9:1 hexanes:Et_2O). Note: Product only slightly observable under UV light; anisaldehyde stain (dark green upon heating) required for adequate visualization. **IR** (neat, cm^{-1}) 3027, 2840, 1662, 1602, 1495, 1453, 1067, 1006, 897, 731, 689, 645; **^{1}H NMR** (400 MHz, $CDCl_3$) δ 7.30-7.27 (m, 2H), 7.21-7.17 (m, 3H), 5.44 (m, 1H), 4.62 (m, 2H), 4.49 (m, 2H), 3.41 (s, 2H); **^{13}C NMR** (100 MHz, $CDCl_3$) δ 138.4, 128.7, 128.5, 126.4, 121.0 100.7, 76.7, 76.0, 33.9; **HRMS** (+NSI) calculated for $C_{11}H_{13}O$ 161.0966, found 161.0961 $[M+H]^+$.

d) Preliminary Results Described in Equation 1 and Scheme 1

General procedure E.

A round bottom flask was charged with iridium phebim catalyst (0.5 mol % or 2 mol %, as indicated), 4Å molecular sieves and a stir bar, then evacuated and backfilled with N_2. Substrate (4 equiv) and anhydrous $PhCF_3$ (1.7 M in substrate) were added via syringe, and the reaction mixture was cooled to -10 ° C in an ice/salt bath. A solution of ethyl diazoacetate (1 equiv, 13 % wt. CH_2Cl_2) in anhydrous $PhCF_3$ (0.29 M in diazoacetate) was added over 5 h via syringe pump. The temperature of the reaction mixture was maintained between -10 ° C and -5 ° C. After addition, once ethyl diazoacetate was completely consumed as judged by TLC, the mixture was concentrated *in vacuo*. The residue was purified as indicated to give the insertion product.

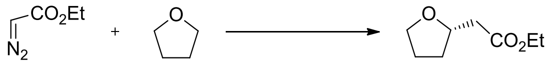

S46

(*S*)-ethyl 2-(tetrahydrofuran-2-yl)acetate (S46). Prepared by general procedure **E** using ethyl diazoacetate (100 mg, 0.75 mmol) in $PhCF_3$ (3 mL), THF (285 µL, 3.52 mmol) in $PhCF_3$ (2 mL), [(*S*,*S*)-^{t}BuPhebox-^{i}Pr]IrCl$_2$(H$_2$O) **1**[20] (2.8 mg, 0.5 mol %), and 4 Å MS (176 mg). Flash chromatography (8:2 → 7:3 pentane:Et_2O) afforded the title compound as a colorless oil (60 mg, 51 % yield, 81:19 er); **R$_f$** 0.45 (7:3 pentane:Et_2O); **IR** (thin film, cm^{-1}) 2979, 1734, 1065; **^{1}H NMR** (400 MHz, $CDCl_3$) δ 4.21 (q, *J* = 7.0 Hz, 1H), 4.10 (q, *J* = 7.2 Hz, 2H), 3.85-3.80 (m, 1H), 3.70 (q, *J* = 7.4 Hz, 1H), 2.53 (dd, *J* = 15.3, 7.4 Hz, 1H), 2.41 (dd, *J* = 15.3, 5.9 Hz, 1H), 2.08-2.00 (m, 1H), 1.92-1.80 (m, 2H), 1.55-1.46 (m, 1H), 1.21 (t, *J* = 7.2 Hz, 3H); **^{13}C NMR** (100 MHz, $CDCl_3$) δ 171.5, 75.4, 68.1, 60.6, 40.8, 31.4, 25.7, 14.3; **HRMS** [+APCI] calculated for $C_8H_{15}O_3$ 159.1016, found 159.1015 $[M+H]^+$; **$[\alpha]^{20}_D$** -2.0 (*c* 1.0, $CHCl_3$); **HPLC** (Daicel OD-H, 1 % IPA:HEX, 1 mL/min), λ 230 nm, t_R(maj) = 6.43 min, t_R(min) = 7.58 min.

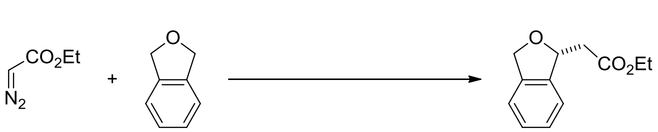

S47

(*R*)-ethyl 2-(1,3-dihydroisobenzofuran-1-yl)acetate (S47). Prepared by general procedure **E** using ethyl diazoacetate (100 mg, 0.75 mmol) in $PhCF_3$ (3 mL), phthalan (380 µL, 3.52 mmol) in $PhCF_3$ (2 mL), [(*S*,*S*)-^{t}BuPhebox-^{i}Pr]IrCl$_2$(H$_2$O) **1**[20] (2.8 mg, 0.5 mol %), and 4 Å MS (176 mg). Flash chromatography (8:2 pentane:Et_2O) afforded the title compound as a colorless oil (125 mg, 81 % yield, 88:12 er); **R$_f$** 0.26 (8:2 pentane:Et_2O); **IR** (thin film, cm^{-1}) 2980, 2858, 1729, 1158, 1036, 750; **^{1}H NMR** (400 MHz, $CDCl_3$) δ 7.29-7.16 (m, 4H), 5.65 (dd, *J* = 6.9, 2.6 Hz, 1H), 5.10

(qd, J = 12.2, 2.1 Hz, 2H), 4.18 (qd, J = 7.1, 1.5 Hz, 2H), 2.74 (qd, J = 14.5, 6.4 Hz, 2H), 1.25 (td, J = 7.1, 0.8 Hz, 3H); **^{13}C NMR** (100 MHz, $CDCl_3$) δ 170.9, 140.8, 139.3, 128.0, 127.5, 121.3, 121.2, 80.4, 72.8, 60.8, 41.8, 14.3; **HRMS** [+NSI] calculated for $C_{12}H_{15}O_3$ 207.1016, found 207.1019 $[M+H]^+$; **$[\alpha]^{20}_D$** +39.1 ° (c 1.0, $CHCl_3$); **HPLC** (Daicel OJ-H, 1 % IPA:HEX, 1 mL/min), λ 254 nm, t_R(min) = 17.5 min, t_R(maj) = 24.9 min.

e) Catalyst Optimization

Catalyst Optimization. Prepared by general procedure **E** using ethyl diazoacetate (100 mg, 0.75 mmol) in $PhCF_3$ (3 mL), phthalan (380 µL, 3.52 mmol) in $PhCF_3$ (2 mL), Ir(III) phebim catalyst (0.5 mol %), and 4 Å MS (176 mg). Flash chromatography (8:2 pentane:Et_2O) afforded the title compound as a colorless oil in the yield and enantioselectivity indicated. Compound was confirmed by ^{1}H NMR identical to above.

Table S1. Ir(III)-Phebim Optimization for C–H Insertion of Ethyl Diazoacetate into Phthalan

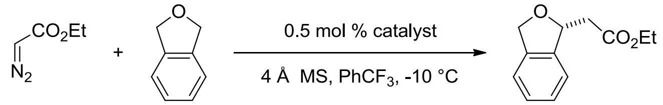

entry	catalyst	yield (%)[b]	er[c]
1	**5**	85	88:12
2	**6**	26	89:11
3	**7**	70	90:10
4	**8**	73	90:10
5	**9**	83	88:12
6	**10**	95	95:5
7	**11**	54	13:87[d]
8	**12**	81	92:8
9	**13**	83	90:10
10	**14**	80	95:5
11	**15**	36	86:14

[a]General conditions: 0.29 M solution of ethyl diazoacetate in $PhCF_3$ was added over 5 h to a mixture of catalyst, phthalan (4 equiv), and 4 Å MS in $PhCF_3$ at -10 °C. [b]Isolated yields. [c]Determined by chiral HPLC. [d]Catalyst **11** is a member of the opposite enantio-series.

f) Acceptor-Only Diazoacetate Scope

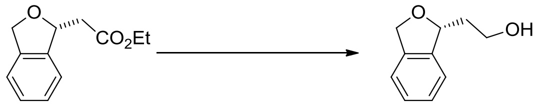

(*R*)-2-(1,3-dihydroisobenzofuran-1-yl)ethanol (S48) A solution of **S47** (0.44 mmol, 1.0 equiv) in THF (2 mL, 0.2 M) was cooled to 0 ° C. LAH (1.3 mL, 1 M in Et_2O, 3 equiv) was added dropwise. The reaction was warmed to room temperature. Once thin layer chromatography indicated complete consumption of starting material (1 h), the reaction mixture was poured into a vigorously stirring solution of Et_2O and a saturated aqueous Rochelle's salt solution. Et_2O was added and the resulting biphasic mixture was stirred vigorously for 3 h. The organic layer was separated, washed with brine, dried over $MgSO_4$ and concentrated *in vacuo*. Preparative thin layer chromatography (7:3 hexanes:EtOAc) afforded the title compound as a light yellow oil

(62 mg, 86 %); **R$_f$** 0.26 (7:3 hexanes:EtOAc); **IR** (thin film, cm^{-1}) 3398, 2855, 1461, 1046, 725; **^{1}H NMR** (600 MHz, $CDCl_3$) δ 7.28-7.21 (m, 3H), 7.15-7.12 (m, 1H), 5.41 (dd, *J* = 8.8, 1.8 Hz, 1H), 5.13 (dd, *J* = 12.3, 2.3 Hz, 1H), 5.05 (dd, *J* = 12.3, 1.2 Hz, 1H), 3.88-3.82 (m, 2H), 2.76 (br s, 1H), 2.17-2.12 (m, 1H), 1.94-1.89 (m, 1H); **^{13}C NMR** (150 MHz, $CDCl_3$) δ 141.4, 138.9, 127.6, 127.4, 121.0, 120.9, 83.7, 72.5, 60.8, 37.8; **HRMS** [+NSI] calculated for $C_{10}H_{13}O_2$ 165.0910, found 165.0909 $[M+H]^+$; **HPLC** (Daicel OJ-H, 5 % IPA:HEX, 1 mL/min), λ 210 nm, t_R(maj) = 14.2 min, t_R(min) = 15.9 min.

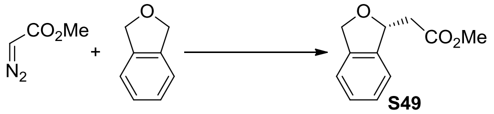

S49

(*R*)-methyl 2-(1,3-dihydroisobenzofuran-1-yl)acetate (S49). Prepared by general procedure **E** using methyl diazoacetate[21] (57 mg, 0.57 mmol) in $PhCF_3$ (2 mL), phthalan (255 µL, 2.3 mmol) in $PhCF_3$ (1.3 mL), **10** (2.7 mg, 0.5 mol %), and 4Å MS (118 mg). Flash chromatography (8:2 pentane:Et_2O) afforded the title compound as a colorless oil (79 mg, 72 % yield, 92:8 er). The title compound was subjected to the LAH reduction procedure above to give (*R*)-2-(1,3-dihydroisobenzofuran-1-yl)ethanol for HPLC analysis. **R$_f$** 0.6 (7:3 pentane:Et_2O); **IR** (thin film, cm^{-1}) 2951, 2859, 1733, 1436, 1365, 1161, 1036, 750; **^{1}H NMR** (400 MHz, $CDCl_3$) δ 7.28-7.16 (m, 4H), 5.67-5.64 (m, 1H), 5.16-5.05 (m, 2H), 3.73 (d, *J* = 0.8 Hz, 3H), 2.83-2.69 (m, 2H); **^{13}C NMR** (150 MHz, $CDCl_3$) δ 171.3, 140.6, 139.1, 127.9, 127.4, 121.1, 80.3, 72.7, 51.8, 41.4; **HRMS** [+NSI] calculated for $C_{11}H_{13}O_3$ 193.0859, found 193.0858 $[M+H]^+$; **$[\alpha]^{20}_D$** +45.8 ° (*c* 1.0, $CHCl_3$).

(*R*)-methyl 2-(1,3-dihydroisobenzofuran-1-yl)acetate (S49). Prepared by general procedure **E** using methyl diazoacetate[21] (56 mg, 0.55 mmol) in $PhCF_3$ (2 mL), phthalan (255 µL, 2.3 mmol) in $PhCF_3$ (1.3 mL), **14** (3 mg, 0.5 mol %), and 4Å MS (118 mg). Flash chromatography (8:2 pentane:Et_2O) afforded the title compound as a colorless oil (73 mg, 69 % yield, 94:6 er). Compound was confirmed by ^{1}H NMR identical to above. The title compound was subjected to the LAH reduction procedure above to give (*R*)-2-(1,3-dihydroisobenzofuran-1-yl)ethanol for HPLC analysis.

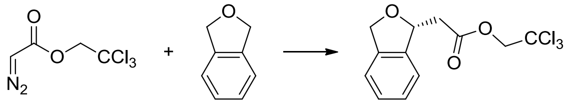

S25 **S50**

(*R*)-2,2,2-trichloroethyl 2-(1,3-dihydroisobenzofuran-1-yl)acetate (S50). Prepared by general procedure **E** using trichloroethyl diazoacetate **S25** (76 mg, 0.35 mmol) in $PhCF_3$ (1.2 mL), phthalan (155 µL, 1.57 mmol) in $PhCF_3$ (0.8 mL), **10** (7 mg, 2 mol %), and 4 Å MS (80 mg). Flash chromatography (95:5 pentane:Et_2O) afforded the title compound as a colorless oil (90 mg, 84 % yield, 94:6 er). The title compound was subjected to the LAH reduction procedure above to give (*R*)-2-(1,3-dihydroisobenzofuran-1-yl)ethanol for HPLC analysis. **R$_f$** 0.28 (95:5 pentane:Et_2O); **IR** (thin film, cm^{-1}) 3019, 2361, 1754, 1148, 1039, 749; **^{1}H NMR** (400 MHz, $CDCl_3$) δ 7.30-7.20 (m, 4H), 5.7 (td, *J* = 5.1, 2.3 Hz, 1H), 5.17 (dd, *J* = 12.1, 2.0 Hz, 1H), 5.08 (dd, *J* = 12.1, 1.6 Hz, 1H), 4.83 (dd, *J* = 12.1, 2.4 Hz, 1H), 4.75 (dd, *J* = 11.7, 2.4 Hz, 1H), 2.97 (dd, *J* = 16.0, 4.7 Hz, 1H), 2.88 (dd, *J* = 16.0, 8.2 Hz, 1H); **^{13}C NMR** (150 MHz, $CDCl_3$) δ

169.2, 140.1, 139.1, 128.0, 127.5,121.2, 121.1, 94.7, 79.7, 74.1, 72.8, 41.2; **HRMS** [+NSI] calculated for $C_{12}H_{12}O_3Cl_3$ 308.9847, found 308.9848 $[M+H]^+$; **[α]^{20}$_D$** +35.2 ° (*c* 1.0, $CHCl_3$).

***R*)-2,2,2-trichloroethyl 2-(1,3-dihydroisobenzofuran-1-yl)acetate (S50).** Prepared by general procedure **E** using trichloroethyl diazoacetate **S25** (76 mg, 0.35 mmol) in $PhCF_3$ (1.2 mL), phthalan (155 µL, 1.57 mmol) in $PhCF_3$ (0.8 mL), **14** (7 mg, 2 mol %), and 4 Å MS (80 mg). Flash chromatography (95:5 pentane:Et_2O) afforded the title compound as a colorless oil (77 mg, 71 % yield, 95:5 er). Compound was confirmed by ^{1}H NMR identical to above. The title compound was subjected to the LAH reduction procedure above to give (*R*)-2-(1,3-dihydroisobenzofuran-1-yl)ethanol for HPLC analysis.

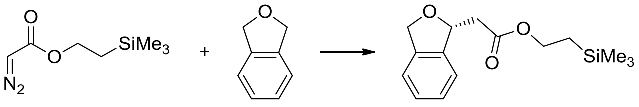

S27 **S51**

(*R*)-2-(trimethylsilyl)ethyl 2-(1,3-dihydroisobenzofuran-1-yl)acetate (S51). Prepared by general procedure **E** using trimethylsilyl ethyl diazoacetate **S27** (66 mg, 0.35 mmol) in $PhCF_3$ (1.2 mL), phthalan (155 µL, 1.57 mmol) in $PhCF_3$ (0.8 mL), **10** (7 mg, 2 mol %), and 4 Å MS (80 mg). Flash chromatography (95:5 pentane:Et_2O) afforded the title compound as a colorless oil (60 mg, 62 % yield, 93:7 er). The title compound was subjected to the LAH reduction procedure above to give (*R*)-2-(1,3-dihydroisobenzofuran-1-yl)ethanol for HPLC analysis. **R_f** 0.24 (95:5 pentane:Et_2O); **IR** (thin film, cm^{-1}) 2953, 2897, 1729, 1248, 1158, 1038, 833, 749; **^{1}H NMR** (600 MHz, $CDCl_3$) δ 7.28-7.17 (m, 4H), 5.67-5.64 (m, 1H), 5.16-5.04 (m, 2H), 4.22 (td, *J* = 8.6, 2.3 Hz, 2H), 2.77 (dd, *J* = 15.7, 4.7 Hz, 1H), 2.71 (dd, *J* = 15.7, 7.8 Hz, 1H), 1.01-0.97 (m, 2H) 0.03 (s, 9H); **^{13}C NMR** (150 MHz, $CDCl_3$) δ 170.9, 140.7, 139.2, 127.8, 127.4, 121.1, 121.0, 80.3, 72.7, 62.9, 41.8, 17.3, -1.5; **HRMS** [+NSI] calculated for $C_{15}H_{23}O_3Si$ 279.1411, found 279.1414 $[M+H]^+$; **[α]^{20}$_D$** +39.3 ° (*c* 1.0, $CHCl_3$).

(*R*)-2-(trimethylsilyl)ethyl 2-(1,3-dihydroisobenzofuran-1-yl)acetate (S51). Prepared by general procedure **E** using trimethylsilyl ethyl diazoacetate **S27** (66 mg, 0.35 mmol) in $PhCF_3$ (1.2 mL), phthalan (154 µL, 1.57 mmol) in $PhCF_3$ (0.8 mL), **14** (7 mg, 2 mol %), and 4 Å MS (80 mg). Flash chromatography (95:5 pentane/Et_2O) afforded the title compound as a colorless oil (66 mg, 67 % yield, 94:6 er). Compound was confirmed by ^{1}H NMR identical to above. The title compound was subjected to the LAH reduction procedure above to give (*R*)-2-(1,3-dihydroisobenzofuran-1-yl)ethanol for HPLC analysis.

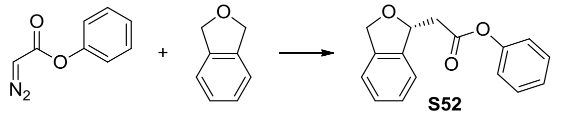

S52

(*R*)-phenyl 2-(1,3-dihydroisobenzofuran-1-yl)acetate (S52). Prepared by general procedure **E** using phenyl diazoacetate[9] (57 mg, 0.35 mmol) in $PhCF_3$ (1.2 mL), phthalan (154 µL, 1.57 mmol) in $PhCF_3$ (0.8 mL), **10** (7 mg, 2 mol %), and 4 Å MS (80 mg). Flash chromatography (8:2 pentane:Et_2O) afforded the title compound as a white solid (58 mg, 65 % yield, 91:9 er). The title compound was subjected to the LAH reduction procedure above to give (*R*)-2-(1,3-dihydroisobenzofuran-1-yl)ethanol for HPLC analysis. **R_f** 0.35 (8:2 hexanes:EtOAc); **mp** 46-48 ° C; **IR** (thin film, cm^{-1}) 3019, 2362, 1753, 1592, 1492, 1214, 1140, 919, 750; **^{1}H NMR** (600 MHz, $CDCl_3$) δ 7.39-7.19 (m, 7H), 7.07-7.04 (m, 2H), 5.78-5.75 (m,

1H), 5.22-5.09 (m, 2H), 3.06 (dd, J = 15.7, 4.7 Hz, 1H), 2.98 (dd, J = 15.7, 7.4 Hz, 1H); **^{13}C NMR** (150 MHz, $CDCl_3$) δ 172.5, 169.3, 158.3, 150.5, 140.4, 139.2, 129.4, 128.0, 127.5, 128.9, 121.5, 121.2, 121.1, 80.3, 72.9, 41.7; **HRMS** [+NSI] calculated for $C_{16}H_{15}O_3$ 255.1016, found 255.1014 $[M+H]^+$; **$[\alpha]^{20}_D$** + 34.0 ° (c 1.0, $CHCl_3$).

(*R*)-phenyl 2-(1,3-dihydroisobenzofuran-1-yl)acetate (S52). Prepared by general procedure **E** using phenyl diazoacetate[9] (57 mg, 0.35 mmol) in $PhCF_3$ (1.2 mL), phthalan (154 µL, 1.57 mmol) in $PhCF_3$ (0.8 mL), **14** (7 mg, 2 mol %), and 4 Å MS (80 mg). Flash chromatography (8:2 pentane:Et_2O) afforded the title compound as a white solid (61 mg, 68 % yield, 93:7 er). Compound was confirmed by ^{1}H NMR identical to above. The title compound was subjected to the LAH reduction procedure above to give (*R*)-2-(1,3-dihydroisobenzofuran-1-yl)ethanol for HPLC analysis.

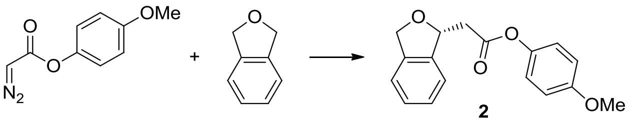

2

(*R*)-4-methoxyphenyl 2-(1,3-dihydroisobenzofuran-1-yl)acetate (2). Prepared by general procedure **E** using 4-methoxy-phenyl diazoacetate[10] (68 mg, 0.35 mmol) in $PhCF_3$ (1.2 mL), phthalan (154 µL, 1.57 mmol) in $PhCF_3$ (0.8 mL), **10** (7 mg, 2 mol %), and 4 Å MS (80 mg). Flash chromatography (8:2 pentane:Et_2O) afforded the title compound as a white solid (57 mg, 57 % yield, 91:9 er). The title compound was subjected to the LAH reduction procedure above to give (*R*)-2-(1,3-dihydroisobenzofuran-1-yl)ethanol for HPLC analysis. **R_f** 0.45 (7:3 hexanes:EtOAc); **mp** 44-47 ° C; **IR** (thin film, cm^{-1}) 3019, 2361, 1751, 1506, 1214, 920, 748; **^{1}H NMR** (600 MHz, $CDCl_3$) δ 7.31-7.26 (m, 4H), 6.98-6.95 (m, 2H), 6.87-6.84 (m, 2H), 5.76-5.74 (m, 1H), 5.19 (dd, J = 11.9, 2.6 Hz, 1H), 5.1 (d, J = 11.9 Hz, 1H), 3.78 (s, 3H), 3.02 (dd, J = 15.4, 4.8 Hz, 1H), 2.95 (dd, J = 15.4, 7.5 Hz, 1H); **^{13}C NMR** (150 MHz, $CDCl_3$) δ 172.5, 169.6, 157.2, 144.0, 140.4, 139.2, 128.0, 127.5, 122.3, 121.2, 121.1, 114.4, 80.3, 72.8, 55.6, 41.6; **HRMS** [+NSI] calculated for $C_{17}H_{17}O_4$ 285.1121, found 285.1118 $[M+H]^+$; **$[\alpha]^{20}_D$** +36.1 ° (c 1.0, $CHCl_3$).

(*R*)-4-methoxyphenyl 2-(1,3-dihydroisobenzofuran-1-yl)acetate (2). Prepared by general procedure **E** using 4-methoxy-phenyl diazoacetate[10] (68 mg, 0.35 mmol) in $PhCF_3$ (1.2 mL), phthalan (154 µL, 1.57 mmol) in $PhCF_3$ (0.8 mL), **14** (7 mg, 2 mol %), and 4 Å MS (80 mg). Flash chromatography (8:2 pentane:Et_2O) afforded the title compound as a white solid (70 mg, 70 % yield, 92:8 er). Compound was confirmed by ^{1}H NMR identical to above. The title compound was subjected to the LAH reduction procedure above to give (*R*)-2-(1,3-dihydroisobenzofuran-1-yl)ethanol for HPLC analysis.

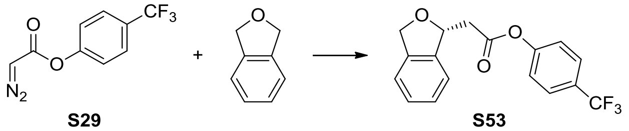

S29 **S53**

(*R*)-4-(trifluoromethyl)phenyl 2-(1,3-dihydroisobenzofuran-1-yl)acetate (S53). Prepared by general procedure **E** using 4-trifluoromethyl-phenyl diazoacetate **S29** (81 mg, 0.35 mmol) in $PhCF_3$ (1.2 mL), phthalan (154 µL, 1.57 mmol) in $PhCF_3$ (0.8 mL), **10** (7 mg, 2 mol %), and 4 Å MS (80 mg). Flash chromatography (9:1 pentane:Et_2O) afforded the title compound as a white solid (88 mg, 78 % yield, 90:10 er). The title compound was subjected to the LAH reduction

procedure above to give (*R*)-2-(1,3-dihydroisobenzofuran-1-yl)ethanol for HPLC analysis. **R_f** 0.5 (8:2 hexanes:EtOAc); **mp** 70-72 ° C; **IR** (thin film, cm^{-1}) 3019, 2925, 2363, 1710, 1362, 1214, 922, 754; **^{1}H NMR** (600 MHz, $CDCl_3$) δ 7.62 (d, *J* = 8.2 Hz, 2H), 7.33-7.28 (m, 2H), 7.27-7.25 (m, 2H), 7.17 (d, *J* = 8.2 Hz, 2H), 5.77-5.74 (m, 1H), 5.19 (dd, *J* = 12.3, 2.9 Hz, 1H), 5.11 (d, *J* = 12.3 Hz, 1H), 3.08 (dd, *J* = 15.2, 4.1 Hz, 1H), 2.98 (dd, *J* = 15.2, 8.2 Hz, 1H); **^{13}C NMR** (150 MHz, $CDCl_3$) δ 168.8, 156.4, 153.0, 140.0, 139.2, 128.1, 127.6, 126.8, 122.1, 121.2, 121.1, 115.4, 80.2, 72.9, 41.6, 29.7; **HRMS** [+NSI] calculated for $C_{17}H_{14}O_3F_3$ 323.0890, found 323.0890 $[M+H]^+$; **$[\alpha]^{20}_D$** + 7.7 ° (*c* 1.0, $CHCl_3$).

(*R*)-4-(trifluoromethyl)phenyl 2-(1,3-dihydroisobenzofuran-1-yl)acetate (S53). Prepared by general procedure **E** using 4-trifluoromethyl-phenyl diazoacetate **S29** (81 mg, 0.35 mmol) in $PhCF_3$ (1.2 mL), phthalan (154 µL, 1.57 mmol) in $PhCF_3$ (0.8 mL), **14** (7 mg, 2 mol %), and 4 Å MS (80 mg). Flash chromatography (9:1 pentane:Et_2O) afforded the title compound as a white solid (78 mg, 69 % yield, 92:8 er). Compound was confirmed by ^{1}H NMR identical to above. The title compound was subjected to the LAH reduction procedure above to give (*R*)-2-(1,3-dihydroisobenzofuran-1-yl)ethanol for HPLC analysis.

g) Substrate Scope

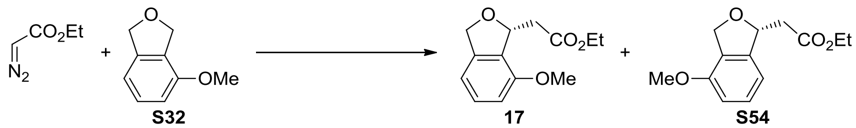

Prepared by general procedure **E** using ethyl diazoacetate (44 mg, 0.33 mmol) in $PhCF_3$ (1.1 mL), **S32** (200 mg, 1.3 mmol) in $PhCF_3$(0.75), **14** (7 mg, 2 mol %), and 4 Å MS (66 mg). Flash chromatography (9:1 → 8:2 pentane:Et_2O) afforded the title compounds as a mixture of separable regioisomers (49 mg, 63 % yield, 98:2 er **17**; 15 mg, 19 % yield, 94:6 er **S54**);

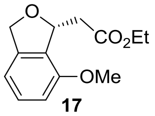

17

(*R*)-ethyl 2-(7-methoxy-1,3-dihydroisobenzofuran-1-yl)acetate (17). The title compound was isolated as 49 mg of a white amorphous solid (63 % yield, 98:2 er); **R_f** 0.28 (9:1 pentane: Et_2O); **IR** (thin film, cm^{-1}) 3019, 2032, 1730, 1614, 1485, 1270, 922, 749, 667; **^{1}H NMR** (600 MHz, $CDCl_3$) δ 7.26-7.24 (m, 1H), 6.79 (d, *J* = 7.5 Hz, 1H), 6.73 (d, *J* = 8.3 Hz, 1H), 5.72 (d, *J* = 9.2 Hz, 1H), 5.15 (dd, *J* = 12.3, 2.6 Hz, 1H), 5.04 (d, *J* = 12.3 Hz, 1H), 4.16 (q, *J* = 7.0 Hz, 2H), 3.81 (s, 3H), 3.1 (dd, *J* = 15.6, 2.9 Hz, 1H), 2.57 (dd, *J* = 15.6, 9.0 Hz, 1H), 1.23 (t, *J* = 7.2 Hz, 3H); **^{13}C NMR** (100 MHz, $CDCl_3$) δ 172.5, 171.3, 154.3, 141.2, 129.7, 113.2, 108.9, 79.9, 73.2, 60.5, 55.1, 39.9, 14.2; **HRMS** [+NSI] calculated for $C_{13}H_{17}O_4$ 237.1121, found 237.1119 $[M+H]^+$; **$[\alpha]^{20}_D$** + 69.5 ° (*c* 1.0, $CHCl_3$); **HPLC** (Daicel OJ-H, 0.5 % IPA:HEX, 1 mL/min), λ 230 nm, t_R(min) = 25.8 min, t_R(maj) = 33.2 min.

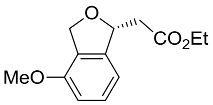

S54

(R)-ethyl 2-(4-methoxy-1,3-dihydroisobenzofuran-1-yl)acetate (S54). The title compound was isolated as 15 mg of a white amorphous solid (19 % yield, 94:6 er); **R_f** 0.30 (9:1 pentane:Et_2O);

IR (thin film, cm^{-1}) 3019, 2342, 1731, 1599, 1487, 1267, 1038, 924, 889, 751; **^{1}H NMR** (600 MHz, $CDCl_3$) δ 7.25-7.24 (m, 1H), 6.75 (t, *J* = 7.5 Hz, 2H), 5.66-5.64 (m, 1H), 5.12 (dd, *J* = 12.7, 2.6 Hz, 1H), 5.04 (d, *J* = 12.3 Hz, 1H), 4.18, (q, *J* = 7.0 Hz, 2H), 3.81 (s, 3H), 2.76 (dd, *J* = 15.8, 4.4 Hz, 1H), 2.7 (dd, *J* = 15.8, 8.3 Hz, 1H), 1.25 (t, *J* = 7.2 Hz, 3H); **^{13}C NMR** (100 MHz, $CDCl_3$) δ 172.5, 129.3, 113.2, 110.0, 109.3, 80.9, 71.3, 60.7, 55.2, 41.7, 14.2; **HRMS** [+NSI] calculated for $C_{13}H_{17}O_4$ 237.1121, found 237.1119 $[M+H]^+$; **$[\alpha]^{20}_D$** + 31.0 ° (*c* 1.0, $CHCl_3$); **HPLC** (Daicel OJ-H, 0.5 % IPA:HEX, 1 mL/min), λ 210 nm, t_R(min) = 24.3 min, t_R(maj) = 31.8 min.

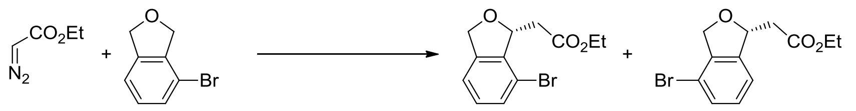

S33 **S55** **18**

(NEM-vi-25-top, NEM-vi-25-bottom). Prepared by general procedure **E** using ethyl diazoacetate (45 mg, 0.34 mmol) in $PhCF_3$ (1.2 mL), **S33** (272 mg, 1.37 mmol) in $PhCF_3$ (0.8 mL), **14** (8 mg, 2 mol %), and 4 Å MS (68 mg). Flash chromatography (100:0 → 95:5 pentane:Et_2O) afforded the title compounds as a mixture of separable regioisomers (28 mg, 29 % yield, 94:6 er **18**; 9.5 mg, 10 % yield **S55**);

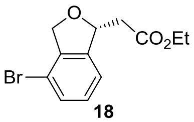

18

(R)-ethyl 2-(4-bromo-1,3-dihydroisobenzofuran-1-yl)acetate (18). The title compound was isolated as 28 mg of a white amorphous solid (29 % yield, 94:6 er); **R_f** 0.28 (9:1 hexanes:EtOAc); **IR** (thin film, cm^{-1}) 3019, 2359, 1733, 1361, 1214, 1042, 923, 750, 668; **^{1}H NMR** (400 MHz, $CDCl_3$) δ 7.39 (d, *J* = 6.6 Hz, 1H), 7.17-7.10 (m, 2H), 5.73 (t, *J* = 7.4 Hz, 1H), 5.10 (dd, *J* = 12.9, 2.7 Hz, 1H), 5.02 (dd, *J* = 12.9, 1.6 Hz, 1H), 2.76-2.74 (m, 2H), 1.25 (t, *J* = 7.2 Hz, 3H); **^{13}C NMR** (150 MHz, $CDCl_3$) δ 170.5, 142.6, 139.9, 130.8, 129.5, 120.0, 115.9, 81.5, 73.8, 60.8, 41.5, 29.7, 14.1; **HRMS** [+NSI] calculated for $C_{12}H_{14}O_3Br$ 285.0121, found 285.0120 $[M+H]^+$; **$[\alpha]^{20}_D$** + 12.8 ° (*c* 1.0, $CHCl_3$); **HPLC** (Daicel OJ-H, 1 % IPA:HEX, 1 mL/min), λ 210 nm, t_R(min) = 10.8 min, t_R(maj) = 17.0 min.

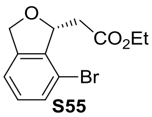

S55

(R)-ethyl 2-(7-bromo-1,3-dihydroisobenzofuran-1-yl)acetate (S55). The title compound was isolated as 9.5 mg of a white amorphous solid (10 % yield); **R_f** 0.26 (9:1 hexanes:EtOAc); **IR** (thin film, cm^{-1}) 3019, 2357, 2196, 1733, 1214, 920, 889, 753, 665; **^{1}H NMR** (400 MHz, $CDCl_3$) δ 7.40-7.38 (m, 1H), 7.16-7.15 (m, 2H), 5.67 (dt, *J* = 8.9, 2.4 Hz, 1H), 5.22 (dd, *J* = 12.5, 2.7 Hz, 1H), 5.08 (dd, *J* = 12.5, 0.8 Hz, 1H), 4.16 (q, *J* = 7.0 Hz, 2H), 3.18 (dd, *J* = 15.6, 2.7 Hz, 1H), 2.66 (dd, *J* = 15.6, 9.0 Hz, 1H), 1.24 (t, *J* = 7.0 Hz, 3H); **^{13}C NMR** (150 MHz, $CDCl_3$) δ 170.7, 141.6, 140.0, 131.0, 129.8, 120.0, 116.3, 81.7, 73.0, 60.7, 39.3, 14.1; **HRMS** [+NSI] calculated for $C_{12}H_{14}O_3Br$ 285.0121, found 285.0118 $[M+H]^+$; **$[\alpha]^{20}_D$** + 56.6 ° (*c* 0.5, $CHCl_3$).

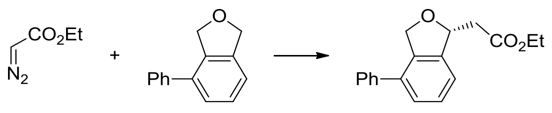

S34 20

(*R*)-ethyl 2-(4-phenyl-1,3-dihydroisobenzofuran-1-yl)acetate (20). Prepared by general procedure **E** using **S34** (102 mg, 0.51 mmol), ethyl diazoacetate (17 mg, 0.13 mmol), **14** (2.6 mg, 2 mol %), and 4 Å MS (27 mg). Flash chromatography (100 % pentane → 95:5 pentane:Et_2O) afforded the title compound as a colorless oil (20 mg, 56 % yield, 96:4 er); **R_f** 0.28 (9:1 pentane:Et_2O); **IR** (thin film, cm^{-1}) 3020, 2007, 1732, 1214, 920, 756; **^{1}H NMR** (400 MHz, $CDCl_3$) δ 7.44-7.32 (m, 7H), 7.17 (d, *J* = 7.0 Hz, 1H), 5.71 (t, *J* = 5.3 Hz, 1H), 5.21 (dd, *J* = 12.5, 2.3 Hz, 1H), 5.15 (dd, *J* = 12.9, 2.0 Hz, 1H), 4.20 (q, *J* = 7.3 Hz, 2H), 2.86-2.74 (m, 2H), 1.26 (t, *J* = 7.2 Hz, 3H); **^{13}C NMR** (150 MHz, $CDCl_3$) δ 170.8, 141.5, 139.8, 137.1, 136.1, 128.7, 128.2, 128.0, 127.8, 127.5, 120.0, 80.4, 72.5, 60.7, 41.6, 24.9, 14.2; **HRMS** [+NSI] calculated for $C_{18}H_{19}O_3$ 283.1329, found 283.1326 $[M+H]^+$; **$[\alpha]^{20}_D$** +6.9 ° (*c* 1.0, $CHCl_3$); **HPLC** (Daicel OJ-H, 1 % IPA:HEX, 1 mL/min), λ 254 nm, t_R(min) = 21.9 min, t_R(maj) = 24.9 min.

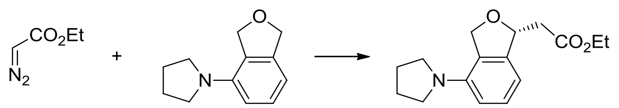

S39 21

(*R*)-ethyl 2-(4-(pyrrolidin-1-yl)-1,3-dihydroisobenzofuran-1-yl)acetate (21). Prepared by general procedure **E** using **S39** (220 mg, 0.92 mmol) in $PhCF_3$ (0.5 mL), ethyl diazoacetate (30 µL, 0.25 mmol) in $PhCF_3$ (0.7 mL), **14** (6.1 mg, 2 mol %), and 4 Å MS (75 mg). Flash chromatography (95:5 → 93:7 hexanes:EtOAc) afforded the title compound as a light yellow oil (45.3 mg, 56% yield, 95:5 er); **R_f** 0.41 (8:2 hexanes:EtOAc); **IR** (neat, cm^{-1}) 2969, 2871, 1729, 1594, 1486, 1458, 1367, 1152, 1030, 770; **^{1}H NMR** (400 MHz, $CDCl_3$) δ 7.15 (t, *J* = 8.0 Hz, 1H), 6.51 (d, *J* = 8.0 Hz, 1H), 6.46 (d, *J* = 8.0 Hz, 1H), 5.60 (m, 1H), 5.37 (dd, *J* = 2.8, 11.2, 1H); 5.28 (dd, *J* = 1.6, 11.2 Hz, 1H); 4.21 (q, *J* = 7.2 Hz, 2H); 3.33 (m, 4H); 2.80 (dd, *J* = 4.0, 16.0 Hz, 1H), 2.68 (dd, *J* = 8.4, 15.2 Hz, 1H), 1.95 (m, 4H), 1.28 (t, *J* = 8.0 Hz, 3H); **^{13}C NMR** (100 MHz, $CDCl_3$) δ 171.1, 144.0, 142.4, 128.8, 123.0, 111.8, 109.2, 80.3, 73.3, 60.6, 49.0, 41.8, 25.3, 14.2; **HRMS** (+NSI) calculated for $C_{16}H_{21}NO_3$ 275.1516, found 275.1510 $[M]^+$; **$[\alpha]^{20}_D$** + 9.0 ° (*c* 0.7, $CHCl_3$); **HPLC** (Daicel OJ-H, 10 % IPA:HEX, 1 mL/min), λ 254 nm, t_R(min) = 14.1 min, t_R(maj) = 31.3 min.

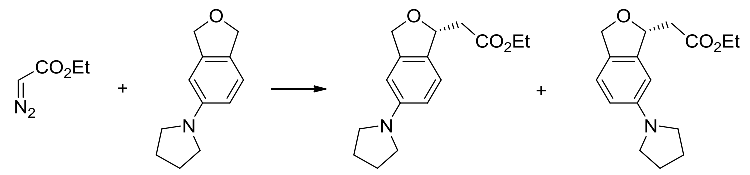

S40 22

(*R*)-ethyl 2-(5-(pyrrolidin-1-yl)-1,3-dihydroisobenzofuran-1-yl)acetate, mix of regioisomers (22). Prepared by general procedure **E** using **S40** (407 mg, 2.15 mmol) in $PhCF_3$ (1.2 mL), ethyl diazoacetate (65 µL, 0..54 mmol) in $PhCF_3$ (1.4 mL), **14** (8.6 mg, 2 mol %), and 4 Å MS (165 mg). Flash chromatography (95:5 → 90:10 hexanes:EtOAc) afforded the title compound as a light pink oil (67.8 mg, 0.246 mmol, 45% yield, 4:1 mix of regioisomers); **R_f** 0.5 (8:2 hexanes:EtOAc); **IR** (neat, cm^{-1}) 2968, 2840, 1729, 1620, 1577, 1506, 1447, 1369, 1156, 1038,

800; **^{1}H NMR** of major regioisomer (400 MHz, $CDCl_3$) δ 7.02 (d, *J* = 8.0 Hz, 1H); 6.48 (dd, *J* = 8.0, 4.0 Hz, 1H); 6.38 (d, *J* = 4.0 Hz, 1H); 5.60 (m, 1H); 5.10 (dd, *J* = 12.0, 2.4, Hz, 1H); 5.02 (d, *J* = 12.0 Hz, 1H); 4.20 (q, *J* = 8.0 Hz, 2H); 3.27 (m, 4H); 2.74 (dd, *J* = 12.0, 4.0 Hz, 1H); 2.69 (dd, *J* = 16.0, 8.0 Hz, 1H); 2.00 (m, 4H); 1.28 (t, *J* = 8.0 Hz, 3H); **^{1}H NMR** of minor regioisomer (400 MHz, $CDCl_3$) δ 7.06 (d, *J* = 8.0 Hz, 0.25H); 6.50 (dd, *J* = 8.0, 4.0 Hz, 0.25H); 6.34 (d, *J* = 4.0 Hz, 0.25H); 5.63 (m, 0.25H); 5.00 (d, *J* = 8.0 Hz, 0.25H); 4.21 (q, *J* = 8.0 Hz, 0.5H); 3.27 (m, 1H); 2.80 (dd, *J* = 16.0, 4.0 Hz, 0.25H); 2.00 (m, 1H); 1.29 (t, *J* = 8.0 Hz, 0.75H); **^{13}C NMR** (signals presumed to correspond to the major regioisomer, 100 MHz, $CDCl_3$) δ 171.1 148.0, 140.5, 127.3, 121.5, 111.0, 103.3, 80.1, 72.8, 60.5, 47.8, 42.1, 25.4, 14.2; **HRMS** (+NSI) calculated for $C_{16}H_{22}NO_3$ 276.1594, found 276.1587 $[M+H]^+$.

The title compound was subjected to the LAH reduction procedure above to give crude 2-(5-(pyrrolidin-1-yl)-1,3-dihydroisobenzofuran-1-yl)ethanol which was then derivatized as the *tert*-butyl-diphenylsilyl ether for HPLC analysis. The crude alcohol was dissolved in CH_2Cl_2 (3.0 mL) in an 8 mL screw cap vial. The mixture was stirred and recrystallized imidazole (22.6 mg, 0.331 mmol, 1.3 equiv) was added followed by TBDPSCl (80 µL, 0.308 mmol, 1.3 equiv) dropwise over 15 seconds at room temperature. Almost immediately, a white precipitate was observed. After 30 min, the reaction was filtered through celite, concentrated, and purified via column chromatography on silica gel (2.5:5:92.5 EtOAc:$PhCH_3$:hexanes) to afford the individual major (40.3 mg, 90:10 er) and minor (8.5 mg, 91:9 er) regioisomers. Major regioisomer: **R_f** 0.32 (5:10:85 EtOAc:$PhCH_3$:hexanes); **IR** (neat, cm^{-1}) 3070, 2929, 2855, 1662, 1506, 1371, 1107, 731, 700, 613; **^{1}H NMR** (400 MHz, $CDCl_3$) δ 7.71-7.68 (m, 4H), 7.44-7.36 (m, 6H), 6.97 (d, *J* = 8.0 Hz, 1H), 6.45 (dd, *J* = 2.0, 8.0 Hz, 1H), 6.38 (d, *J* = 2.0 Hz, 1H), 5.34 (m, 1H), 5.02 (dd, *J* = 2.0, 12.0, 1H), 4.97 (d, *J* = 12.0 Hz, 1H), 3.90 (m, 2H), 3.27 (m, 4H), 2.06 (m, 1H), 2.00 (m, 4H), 1.90 (m, 1H), 1.06 (s, 9H); **^{13}C NMR** (100 MHz, $CDCl_3$) δ 147.8, 140.6, 135.6, 1356, 134.1, 134.0, 129.5, 129.0, 127.6, 121.6, 110.9, 103.5, 80.5, 72.4, 60.9, 47.9, 39.7, 26.9, 25.4, 19.2; **HRMS** (+NSI) calculated for $C_{30}H_{37}NO_2Si$ 471.2594, found 471.2593 $[M]^+$; **$[\alpha]^{20}_D$** + 8.0 ° (*c* 0.5, $CHCl_3$); **HPLC** (Daicel OJ-H, 15 % IPA:HEX, 0.3 mL/min), λ 254 nm, t_R(min) = 26.0 min, t_R(maj) = 40.8 min. Minor regioisomer: **R_f** 0.39 (5:10:85 EtOAc:$PhCH_3$:hexanes); **IR** (neat, cm^{-1}) 2928, 2854, 1621, 1505, 1372, 1108, 732, 701, 613; **^{1}H NMR** (400 MHz, $CDCl_3$) δ 7.72-7.67 (m, 4H), 7.44-7.35 (m, 6H), 7.04 (d, *J* = 8.0 Hz, 1H), 6.47 (dd, *J* = 2.0, 8.0 Hz, 1H), 6.32 (d, *J* = 8.0 Hz, 1H), 5.36 (m, 1H), 4.99 (dd, *J* = 2.0, 10.0 Hz, 1H), 4.95 (d, *J* = 10.0 Hz, 1H), 3.93 (m, 2H); 3.26 (m, 4H), 2.08 (m, 1H), 2.02 (m, 4H), 1.94 (m, 1H), 1.06 (s, 9H); **^{13}C NMR** (100 MHz, $CDCl_3$) δ 135.6, 135.5, 134.1, 133.9, 129.5, 129.5, 127.6, 127.6, 121.4, 110.0, 86.6, 80.8, 72.1, 60.9, 56.9, 47.9, 39.3, 26.9, 25.4, 19.2; **HRMS** (+NSI) calculated for $C_{30}H_{37}NO_2Si$ 471.2594, found 471.2593 $[M]^+$; **$[\alpha]^{20}_D$** + 5.1 ° (*c* 0.4, $CHCl_3$); **HPLC** (Daicel OJ-H, 15 % IPA:HEX, 0.1 mL/min), λ 254 nm, t_R(min) = 51.8 min, t_R(maj) = 45.7 min.

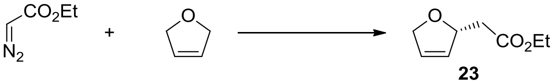

23

(*R*)-ethyl 2-(2,5-dihydrofuran-2-yl)acetate (23). Prepared according to general procedure **E** using ethyl diazoacetate (100 mg, 0.75 mmol) in $PhCF_3$ (3 mL), 2,5-dihydrofuran (226 µL, 3 mmol) in $PhCF_3$ (2 mL), **14** (6 mg, 0.5 mol %), and 4 Å MS (176 mg). Flash chromatography (8:2 pentane:Et_2O) afforded the title compound as a colorless oil (91 mg, 77 %, 88:12 er); **R_f** 0.31 (8:2 pentane:Et_2O); **IR** (thin film, cm^{-1}) 2983, 1731, 1179, 1028; **^{1}H NMR** (400 MHz,

$CDCl_3$) δ 5.88 (dd, *J* = 6.3, 2.0 Hz, 1H), 5.80-5.78 (m, 1H), 5.16-5.10 (m, 1H), 4.65-4.52 (m, 2H), 4.09 (q, *J* = 7.2 Hz, 2H), 2.54-2.43 (m, 2H), 1.2 (t, *J* = 7.0 Hz, 3H); **^{13}C NMR** (100 MHz, $CDCl_3$) δ 171.0, 128.8, 127.5, 82.4, 75.3, 60.6, 41.3, 14.3; **HRMS** [+NSI] calculated for $C_8H_{13}O_3$ 157.0865, found 157.0861 $[M+H]^+$; **$[\alpha]^{20}_D$** -10.9 ° (*c* 0.25, $CHCl_3$); **HPLC** (Daicel OJ-H, 1 % IPA:HEX, 0.7 mL/min), λ 210 nm, t_R(min) = 14.6 min, t_R(maj) = 15.9 min.

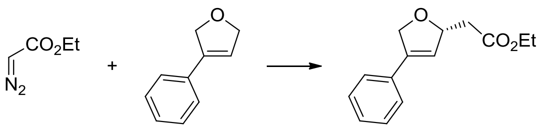

S43 **24**

(*R*)-ethyl 2-(4-phenyl-2,5-dihydrofuran-2-yl)acetate (24). Prepared according to general procedure **E** using ethyl diazoacetate (55 µL, 0.46 mmol) in $PhCF_3$ (1.8 mL), **S43** (304 mg, 2.0 mmol) in $PhCF_3$ (1.2 mL), **14** (11 mg, 2.0 mol %), and 4 Å MS (103 mg). Flash chromatography (95:5 → 85:15 hexanes:Et_2O) afforded the title compound as a clear oil (61 mg, 57 %, 85:15 er); **R_f** 0.23 (4:1 hexanes:Et_2O); **IR** (neat, cm^{-1}) 2980, 2850, 1727, 1372, 1159, 1078, 1033, 750, 691; **^{1}H NMR** (400 MHz, $CDCl_3$) δ 7.39-7.28 (m, 5H), 6.21 (app q, *J* = 2.2 Hz, 1H), 5.39 (m, 1H), 5.06 (ddd, *J* = 2.0, 5.6, 12.0 Hz, 1H), 4.98 (ddd, *J* = 2.4, 3.6, 12.0 Hz, 1H), 4.18 (q, *J* = 8.0 Hz, 2H), 3.41 (s, 2H), 2.69 (dd, *J* = 7.0, 16.0 Hz, 1H), 2.61 (dd, *J* = 6.0, 16.0 Hz, 1H), 1.28 (t, *J* = 8.0 Hz, 3H); **^{13}C NMR** (100 MHz, $CDCl_3$) δ 170.9, 139.4, 132.0, 128.6, 128.3, 125.8, 122.6, 83.5, 75.0, 60.6, 41.3, 14.2; **HRMS** (+NSI) calculated for $C_{14}H_{17}O_3$ 233.1178, found 233.1170$[M+H]^+$. **$[\alpha]^{20}_D$** -34.3° (c 1.0, $CHCl_3$); **HPLC** (Daicel OJ-H, 10 % IPA:HEX, 1.0 mL/min), λ 254 nm, t_R(min) = 16.2 min, t_R(maj) = 29.1 min.

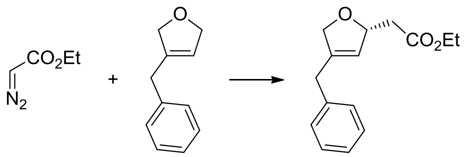

S45 **25**

(*R*)-ethyl 2-(4-benzyl-2,5-dihydrofuran-2-yl)acetate (25). Prepared according to general procedure **E** using ethyl diazoacetate (65 µL, 0.54 mmol) in $PhCF_3$ (2.1 mL), **S45** (410 mg, 2.6 mmol) in $PhCF_3$ (1.4 mL), **14** (13 mg, 2.0 mol %), and 4 Å MS (126 mg). Flash chromatography (95:5 → 85:15 hexanes:Et_2O) afforded the title compound as a clear oil (41.5 mg, 31% yield, 81:19 er); **R_f** 0.28 (8:2 hexanes:Et_2O); Note: Product only slightly observable under UV light; anisaldehyde stain (dark green upon heating) required for adequate visualization; **IR** (neat, cm^{-1}) 3027, 2980, 2845, 1729, 1494, 1262, 1153, 1065, 1027, 758, 700; **^{1}H NMR** (400 MHz, $CDCl_3$) δ 7.32-7.28 (m, 2H), 7.25-7.17 (m, 3H), 5.45 (m, 1H), 5.20 (m, 1H), 4.57-4.46 (m, 2H), 4.14 (q, *J* = 8.0 Hz, 2H), 3.42 (s, 2H), 2.56 (dd, *J* = 7.2, 15.2 Hz, 1H), 2.50 (dd, *J* = 5.6, 15.2 Hz, 1H), 1.25 (t, *J* = 8.0 Hz, 3H), **^{13}C NMR** (100 MHz, $CDCl_3$) δ 171.1, 141.0, 138.1, 128.6, 128.5, 126.4, 123.6, 82.8, 76.5, 60.5, 41.4, 33.8, 14.2; **HRMS** (+NSI) calculated for $C_{15}H_{18}O_3Na$ 269.1154, found 269.1146 $[M+Na]^+$. **$[\alpha]^{20}_D$** -31.6° (c 1.1, $CHCl_3$); **HPLC** (Daicel OJ-H 10 % IPA:HEX, 1.0 mL/min), λ 210 nm, t_R(min) = 12.9 min, t_R(maj) = 14.3 min.

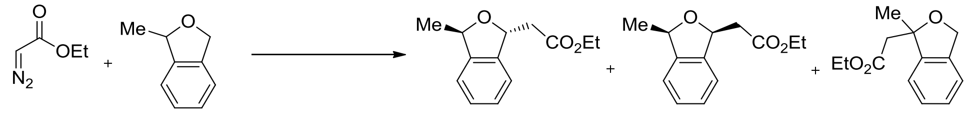

26 **27** **28**

ethyl 2-((1*R*,3*R*)-3-methyl-1,3-dihydroisobenzofuran-1-yl)acetate (26, 27, 28). Prepared by general procedure **E** using ethyl diazoacetate (117 mg, 0.88 mmol) in $PhCF_3$ (3 mL), 1,3-dihydro-1-methylisobenzofuran[22] (472 mg, 3.5 mmol) in $PhCF_3$ (2 mL), **14** (4.5 mg, 0.5 mol %), and 4 Å MS (176 mg). Ethyl diazoacetate solution was added to the substrate solution at -10 ° C and warmed to room temperature overnight. Flash chromatography (97:3 → 95:5 → 9:1 pentane:Et_2O) afforded the title compounds as a 85:5:10 mixture of **26**:**27**:**28** by GC and crude ^{1}H NMR. (116 mg, 60 % yield). **GC** (CHIRASIL DEX, 120 → 140 °C, 1 °C/min) t_r(**28**) = 18.596 min, t_r(**26**) = 21.839 min, t_r(**27**) = 23.556 min. Major product **26** was characterized as follows (88:12 er).

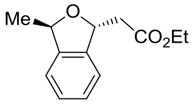

26

ethyl 2-((1R,3R)-3-methyl-1,3-dihydroisobenzofuran-1-yl)acetate (26). R_f 0.41 (9:1 pentane:Et_2O); **IR** (thin film, cm^{-1}) 3019, 2162, 2010, 1731, 1214, 1033, 920, 752, 668; **^{1}H NMR** (400 MHz, $CDCl_3$) δ 7.33-7.25 (m, 2H), 7.20-7.14 (m, 2H), 5.70 (td, *J* = 6.4, 2.9 Hz, 1H), 5.39 (qd, *J* = 6.3, 2.6 Hz, 1H), 4.19 (q, *J* = 7.2 Hz, 2H), 2.72 (d, *J* = 6.4 Hz, 2H), 1.48 (d, *J* = 6.4 Hz, 3H), 1.26 (t, *J* = 7.0 Hz, 3H); **^{13}C NMR** (150 MHz, $CDCl_3$) δ 170.9, 143.4, 140.7, 127.9, 127.5, 121.2, 121.0, 79.1, 79.0, 60.6, 41.8, 22.0, 14.2; **HRMS** [+NSI] calculated for $C_{13}H_{17}O_3$ 221.1172, found 221.1170 $[M+H]^+$; **$[\alpha]^{20}_D$** + 10.2 ° (*c* 1.0, $CHCl_3$); **HPLC** (Daicel OJ-H, 1 % IPA:HEX, 1 mL/min), λ 254 nm, t_R(min) = 8.4 min, t_R(maj) = 9.7 min.

III. Kinetic Isotope Effect

For mechanistic insight, a 1:1 mixture of tetrahydrofuran and its fully deuterated analogue was subjected to C–H insertion conditions with ethyl diazoacetate and Ir(III) phebox **1** in order to calculate a kinetic isotope effect of 1.8 (Scheme S1). This value is very close to those previously reported for Rh-mediated donor/acceptor C–H into cyclohexane (2.0) and tetrahydrofuran (3.0),[23] indicating consistency with a concerted C–H insertion mechanism.

Scheme S1. Kinetic Isotope Effect for C–H Insertion of Ethyl Diazoacetate into Tetrahydrofuran

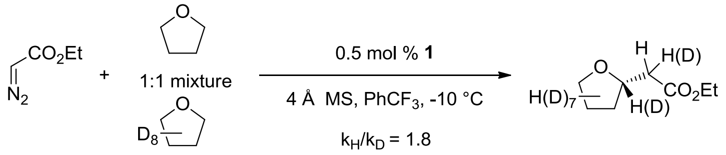

a) Kinetic Isotope Effect by ^{1}H NMR

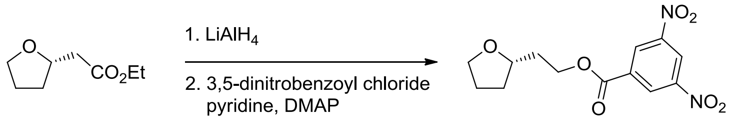

S56

(*S*)-2-(tetrahydrofuran-2-yl)ethyl 3,5-dinitrobenzoate (S56). A dry round bottom flask was charged with (*S*)-ethyl 2-(tetrahydrofuran-2-yl)acetate (48 mg, 0.30 mmol, 1.0 equiv) in THF (2 mL). The reaction mixture was cooled to 0 ° C. $LiAlH_4$ (0.9 mL, 1 M in Et_2O, 3.0 equiv) was added dropwise, and the reaction was stirred overnight. Once thin layer chromatography indicated complete consumption of starting material, the reaction mixture was poured into a vigorously stirring solution of Et_2O (10 mL) and a saturated aqueous Rochelle's salt solution (5 mL). Et_2O (20 mL) was added and the resulting biphasic mixture was stirred vigorously for approximately 3 h. The organic layer was separated, washed with brine, dried over $MgSO_4$ and concentrated in vacuo. The crude product was dissolved in CH_2Cl_2 (1.2 mL). Et_3N (0.12 mL, 0.9 mmol, 3.0 equiv) and DMAP (1 mg, 5.5 μmol, 20 mol %) were added to the solution under N_2 and stirred for 10 min. 3,5-dinitrobenzoyl chloride (138 mg, 0.6 mmol, 2.0 equiv) was added in one portion, and the mixture was stirred for 1.5 h. NH_4Cl (aq) was added to the reaction mixture and extracted with CH_2Cl_2 (3 x). The combined organic layers were washed with $NaHCO_3$ (aq). The aqueous layer was extracted with CH_2Cl_2 (3 x). The combined organic layers were dried with $MgSO_4$, filtered, and concentrated *in vacuo*. Purification by preparative TLC (8:2 hexanes:EtOAc, 2 runs) afforded the title compound as a light yellow oil (55 mg, 59 % yield over 2 steps); $\mathbf{R_f}$ 0.21 (8:2 hexanes:EtOAc); **IR** (thin film, cm^{-1}) 3102, 2967, 2874, 1730, 1543, 1345, 1280, 1167, 722; **^{1}H NMR** (400 MHz, $CDCl_3$) 9.20 (t, *J* = 2.1 Hz, 1H), 9.14 (d, *J* = 2.1 Hz, 2H), 4.56 (td, *J* = 6.6, 1.8 Hz, 2H), 4.03-3.96 (m, 1H), 3.91-3.85 (m, 1H), 3.74 (td, *J* = 7.9, 6.4 Hz, 1H), 2.12-1.98 (m, 3H), 1.96-1.88 (m, 2H), 1.59-1.50 (m, 1H); **^{13}C NMR** (150 MHz, $CDCl_3$) δ 162.7, 148.9, 134.3, 129.7, 129.6, 122.6, 122.5, 76.0, 68.1, 64.9, 34.8, 31.9, 25.8; **HRMS** [+NSI] calculated for $C_{13}H_{15}N_2O_7$ 311.0874, found 311.0871 $[M+H]^+$.

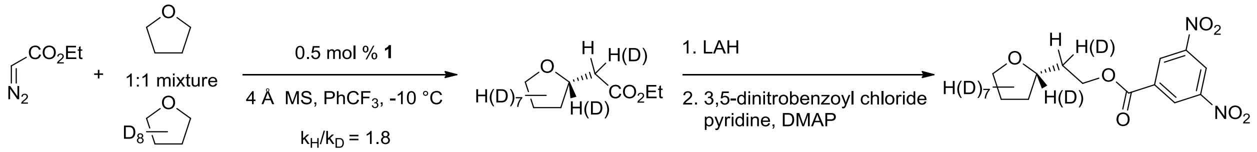

(*S*)-2-(tetrahydrofuran-2-yl)ethyl 3,5-dinitrobenzoate. Prepared by general procedure **E** using ethyl diazoacetate (336 mg, 2.5 mmol) in $PhCF_3$ (8.6 mL), THF (408 µL, 5.0 mmol) and d^8-THF (409 µL, 5.0 mmol) in $PhCF_3$ (5.7 mL), [(*S*,*S*)-^{t}BuPhebox-^{i}Pr]$IrCl_2(H_2O)$ **1**[20] (8 mg, 0.5 mol %), and 4 Å MS (502 mg). The crude residue was taken forward without further purification. A dry round bottom flask was charged with the crude residue and dissolved in Et_2O (5 mL). The reaction mixture was cooled to 0 ° C. $LiAlH_4$ (7.6 mL, 1 M in Et_2O, 3.0 equiv) was added dropwise, and the reaction was stirred overnight. Once thin layer chromatography indicated complete consumption of starting material, the reaction mixture was poured into a vigorously stirring solution of Et_2O (30 mL) and a saturated aqueous Rochelle's salt solution (15 mL). Et_2O (60 mL) was added and the resulting biphasic mixture was stirred vigorously for approximately 3 h. The organic layer was separated, washed with brine, dried over $MgSO_4$ and concentrated in vacuo. The crude product was dissolved in CH_2Cl_2 (8 mL). Pyridine (610 µL, 7.6 mmol, 3.0 equiv) and DMAP (31 mg, 0.26 mmol, 10 mol %) were added to the solution under N_2 and stirred for 10 min. 3,5-dinitrobenzoyl chloride (1.15 g, 5.0 mmol, 2.0 equiv) was added in one portion, and the mixture was stirred for 1.5 h. NH_4Cl (aq) was added to the reaction mixture and extracted with CH_2Cl_2 (3 x). The combined organic layers were washed with $NaHCO_3$ (aq). The aqueous layer was extracted with CH_2Cl_2 (3 x). The combined organic layers were dried with $MgSO_4$, filtered, and concentrated *in vacuo*. Flash chromatography (8:2 hexanes:EtOAc) afforded the title compound as a light yellow oil (192 mg, 25 % yield over 3 steps).

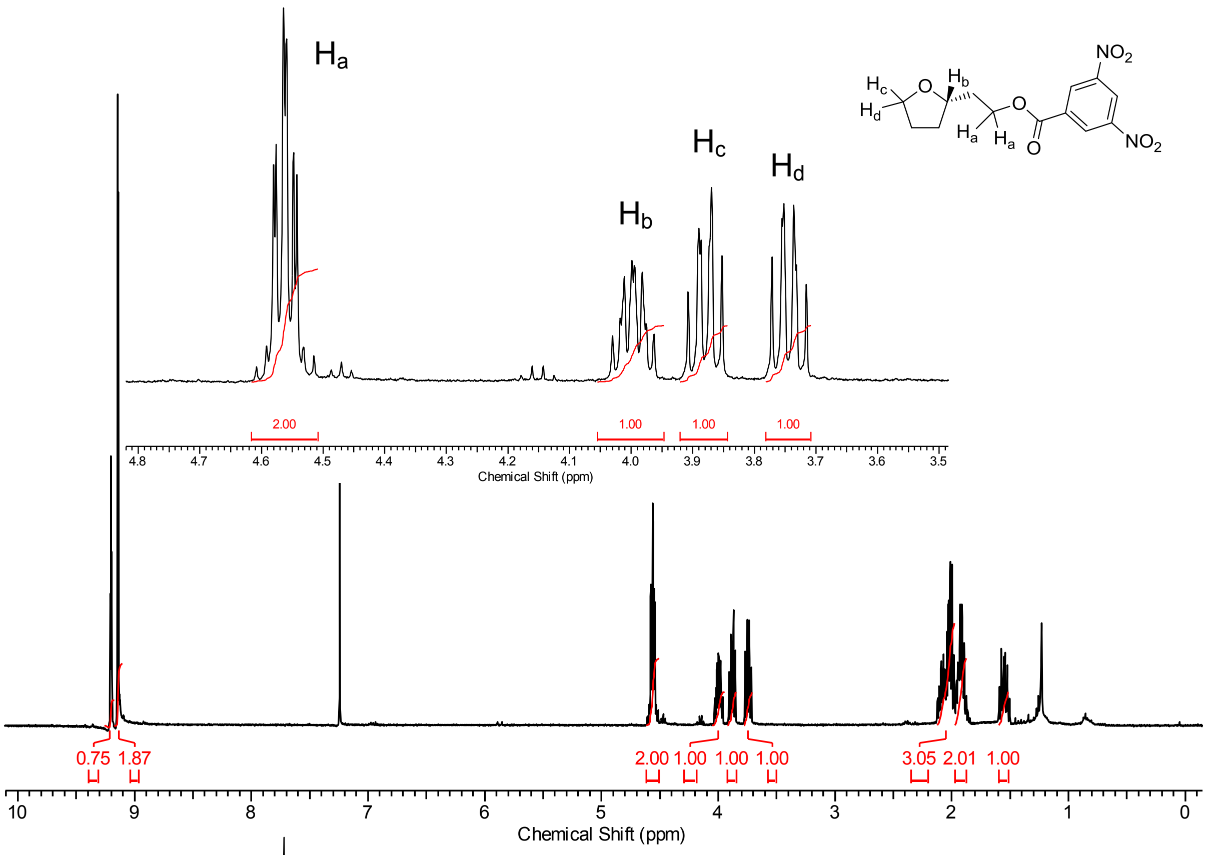
Ha
Hb
Hc
Hd
NO2
NO2
O
O
O
Chemical Shift (ppm)
0.75 1.87
2.00 1.00 1.00 1.00
3.05 2.01 1.00

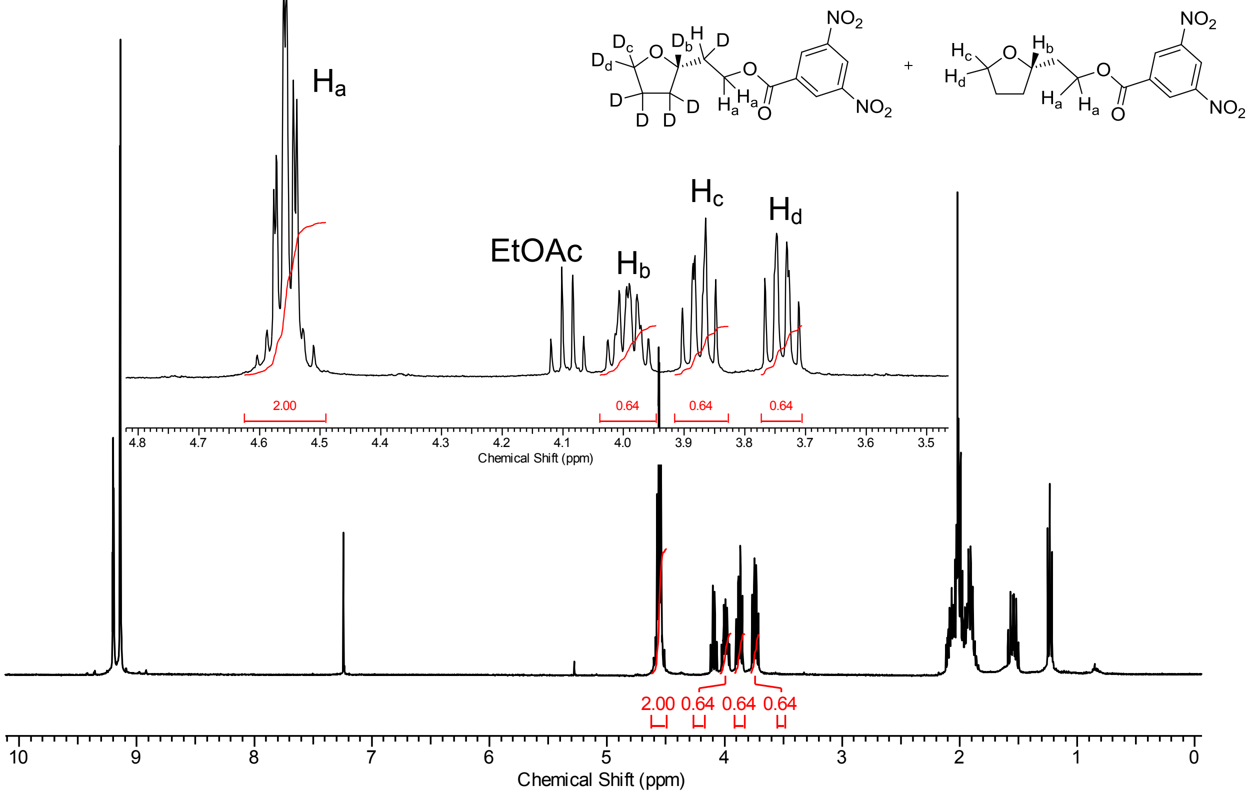
Ha
EtOAc
Hb
Hc
Hd
Dc
Dd
Db
D
+
Chemical Shift (ppm)
2.00 0.64 0.64 0.64

Calculations used for the determination of KIE value from ^{1}H NMR analysis:

$H_a = 2.0$

$H_b = 0.64$

$D_b = 1 – 0.64 = 0.36$

$H_c = 0.64$

$D_c = 1 – 0.64 = 0.36$

$H_d = 0.64$

$D_d = 1 – 0.64 = 0.36$

% H incorporated = 0.64 / 1.0 = 64 %

% D incorporated = 0.36 / 1.0 = 36 %

KIE = K_H / K_D = 0.64 / 0.36 = **1.8**

b) Kinetic Isotope Effect by Mass Spectrometry

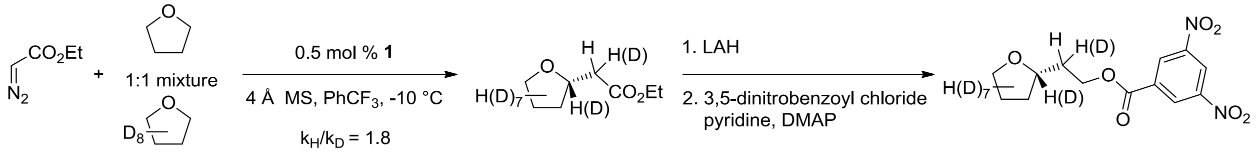

(*S*)-2-(tetrahydrofuran-2-yl)ethyl 3,5-dinitrobenzoate. Prepared by general procedure **E** using ethyl diazoacetate (100 mg, 0.88 mmol) in $PhCF_3$ (3 mL), THF (143 µL, 1.76 mmol) and d^8-THF (142 µL, 1.76 mmol) in $PhCF_3$ (2 mL), [(*S*,*S*)-^{t}BuPhebox-^{i}Pr]$IrCl_2(H_2O)$ **1**[20] (2.8 mg, 0.5 mol %), and 4 Å MS (176 mg). The crude residue was analyzed by ESI-MS.

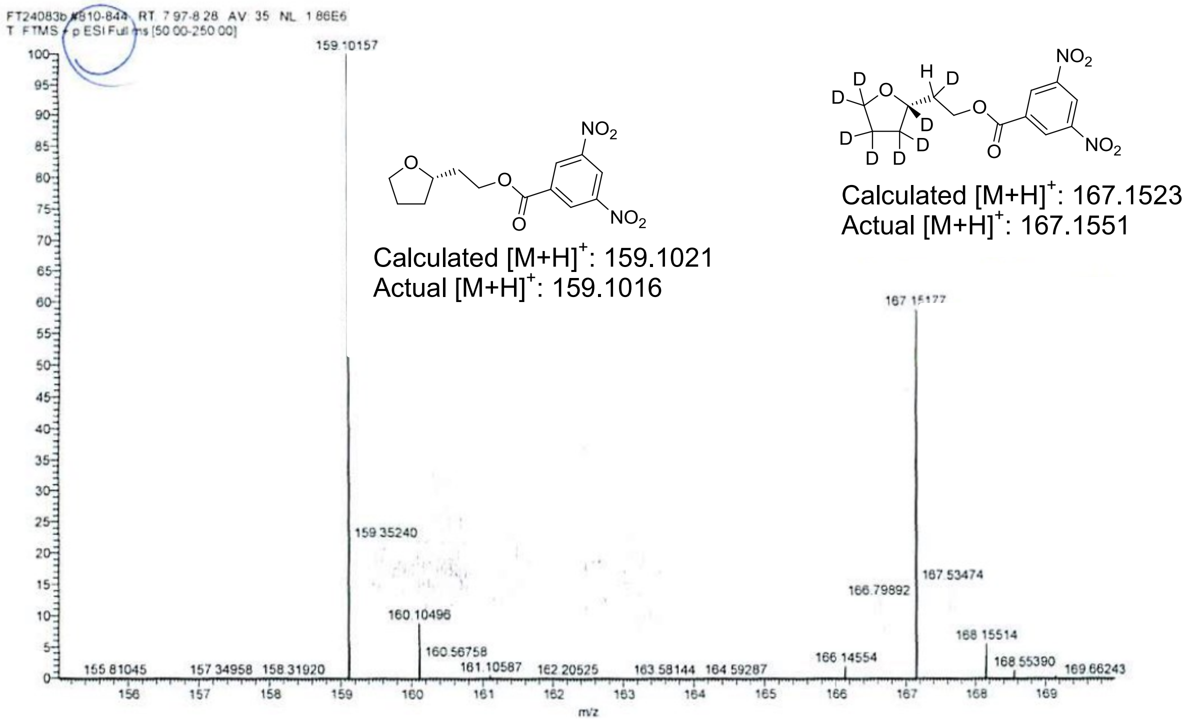

m/z	Intensity	Relative
159.09913	130501.1	7.00
159.10157	1864901.6	100.00
160.10496	162675.2	8.72
167.15177	1101378.0	59.06
168.15514	102915.4	5.52

KIE = K_H / K_D = 100.00 / 59.06 = **1.7**

IV. X-Ray Crystallographic Information

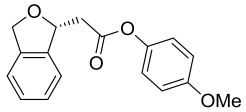

2

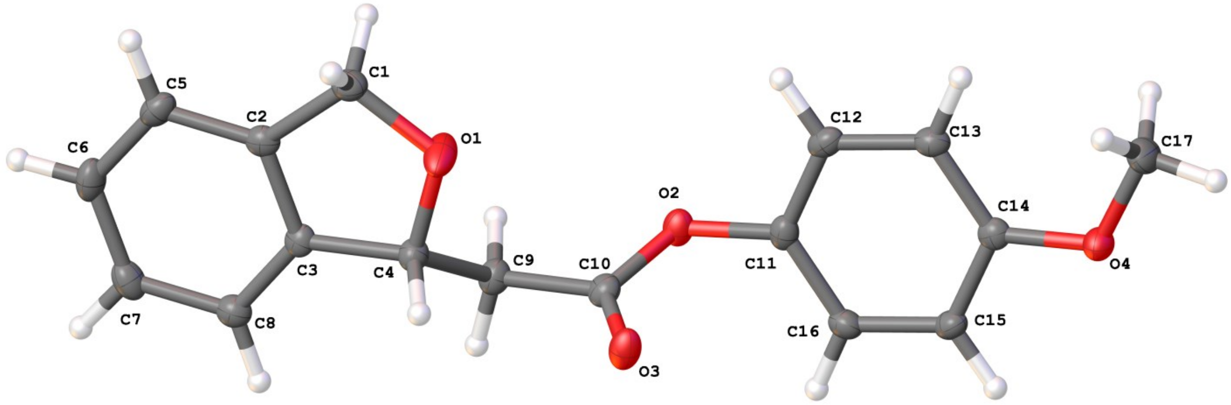

Table S2. Structural refinement data for (*R*)-4-methoxyphenyl 2-(1,3-dihydroisobenzofuran-1-yl)acetate (**2**).

	**2**
**Formula**	$C_{17}H_{16}O_4$
**Form. Wt. (g/mol)**	284.30
**T (K)**	100.02
**Crystal system**	Orthorhombic
**Space group**	$P2_12_12_1$
a **(Å)**	7.47301(10)
b **(Å)**	12.08020(15)
c **(Å)**	15.6344(2)
**α (°)**	90
**β (°)**	90
**γ (°)**	90
**V (Å^3)**	1411.40(3)
**Z**	4

**ρ_{calc} (g/cm)**	1.338
**μ/mm^{-1}**	0.781
**F(000)**	600.0
**Crystal size (mm^3)**	0.23 × 0.185 × 0.176
**Radiation**	CuKα (λ = 1.54178)
**2Θ range**	9.252 to 146.698
**Index ranges**	$-9 \leq h \leq 8$, $-15 \leq k \leq 14$, $-19 \leq l \leq 19$
**Reflns. Collected**	24525
**Unique reflns.**	2805 [R_{int} = 0.0236, R_{sigma} = 0.0108]
**Data/rest./par.**	2805/36/242
GOF	1.101
**Final R indexes [I>2σ (I)]**	R_1 = 0.0270, wR_2 = 0.0668
**All data**	R_1 = 0.0273, wR_2 = 0.0670
**Largest diff. peak/hole / e Å^{-3}**	0.17/-0.26
**Flack parameter**	0.06(4)

V. Computational Details

All calculations were performed using the Gaussian 09 software package.[24] The geometries of molecules were optimized without symmetry constraints at the level of theory using M06 functional[25] and a combined basis set (denoted as BSS) consisting of LANL08(f) basis sets associated with the Hay-Wadt relativistic effective core potentials (RECPs)[26] for Ir atom, and 6-31G(d,p) basis sets for all other atoms. All transition states were confirmed to have one imaginary frequency associated with the reaction coordinate. The nature of transition states was confirmed by performing IRC (intrinsic reaction coordinate) calculations. Solvent effects were incorporated by using the CPCM method[27] with all geometries optimized in dichloromethane solvent at the M06/BSS level of theory. The reported enthalpies and Gibbs free energies (in kcal/mol) were calculated at the standard conditions (298.15 K and 1 atm).

Table S3: The calculated geometries (Cartesian coordinates, in Å) of the reported transition states.

TS1 pro-*S*

C	2.17840000	0.74113000	-0.07264100
C	2.88575000	0.98729100	1.20243400
O	3.47961000	-0.10226100	1.69671700
C	3.99517000	-0.02076900	3.03565600
Ir	0.16018400	0.20326200	-0.04858300
Cl	0.20107800	-0.18205600	2.33414400
C	-1.80189000	-0.12218600	-0.04625300
C	-2.68537600	0.92468800	0.20542900
C	-4.06477600	0.69923300	0.22479500
C	-4.57181400	-0.58520500	-0.02353000
C	-3.67189800	-1.63888800	-0.27607300
C	-2.29856800	-1.40573300	-0.28045700
C	-1.91636800	2.12538900	0.46932600
O	-2.43305900	3.28529400	0.85369400
C	-1.30661600	4.11480400	1.25566800
C	-0.06558700	3.39684500	0.70362500
N	-0.61524500	2.07759000	0.37055600
C	-1.18218700	-2.30723600	-0.49598100
O	-1.30116700	-3.60110000	-0.76371300
C	0.03768400	-4.07292500	-1.08193600
C	0.97634400	-2.95038600	-0.61398200
N	0.03459200	-1.83852200	-0.42777600
C	-6.07321100	-0.88029100	-0.03162900
C	1.77082300	-3.23808000	0.66575400
C	0.59071400	4.06548300	-0.50871900
Cl	0.14342100	0.59834200	-2.42792000
O	2.87286200	2.07141100	1.75474400
H	-4.05103400	-2.64311600	-0.46585100
H	-4.73923700	1.52673600	0.43450600
C	-6.39359600	-1.95999200	1.00782100
C	-6.48488600	-1.37565300	-1.42265100
C	-6.90766600	0.35473200	0.29921700
H	2.20470500	-2.27104800	0.97329800
H	-1.31523400	4.16286200	2.34937800
H	-1.46862600	5.11751700	0.84984100
H	0.07567300	-4.23080700	-2.16466000
H	0.18512000	-5.03066000	-0.57440000
H	1.67923400	-2.67922300	-1.41321100
H	0.69849800	3.26300500	1.48220600
H	1.34376100	3.34841500	-0.87175300
H	3.24673300	0.46551800	3.67201800
H	4.89643700	0.60563800	3.03474100
H	-7.56251000	-1.58702100	-1.44826200
H	-5.96001000	-2.29688400	-1.70415000
H	-6.26920600	-0.61939000	-2.18878600
H	-7.47039300	-2.17627600	1.00910000
H	-6.11265200	-1.63066500	2.01667800
H	-5.86901500	-2.90117900	0.80340100
H	-7.97256100	0.09029000	0.28875600
H	-6.76177400	1.15944500	-0.43296800

H	-6.67648700	0.75010300	1.29680100
C	1.31303100	5.33267800	-0.07427500
H	1.83319700	5.79546600	-0.92139100
H	0.61233100	6.08015500	0.32408200
H	2.05669500	5.12112600	0.70479400
C	-0.38798100	4.33047900	-1.64265700
H	0.14054600	4.73238600	-2.51552000
H	-0.89426200	3.41004700	-1.95906600
H	-1.15071300	5.06982800	-1.36062200
C	2.90600900	-4.20777400	0.36837700
H	3.51618300	-4.37935200	1.26384400
H	2.52353300	-5.18615900	0.04362000
H	3.56640000	-3.83193900	-0.42500000
C	0.89766200	-3.73855500	1.80704300
H	1.48813300	-3.82526600	2.72794000
H	0.07172700	-3.04809700	2.01470000
H	0.48265700	-4.73482800	1.59941400
C	4.27912300	-1.43146700	3.47277700
H	4.70620300	-1.44019200	4.48083600
H	3.35289400	-2.01945200	3.48705800
H	4.98906600	-1.91799100	2.79321400
H	2.70577400	-0.44135500	-3.92467200
H	2.45432600	1.50287100	-0.81535600
C	3.86899000	-0.80203500	-1.14202100
O	3.42368600	-1.42444600	-2.25411900
C	3.66518600	-0.59836600	-3.42129600
C	4.30282500	0.68187600	-2.90178200
C	4.85987100	0.25327700	-1.54473100
H	2.84495900	-0.26273500	-0.61738900
H	4.09885300	-1.50039300	-0.32812000
H	4.32895100	-1.17211100	-4.07871100
H	5.07227800	1.06278900	-3.57769300
H	3.54312600	1.46293900	-2.77551100
H	5.83895800	-0.23333000	-1.65419200
H	4.96421900	1.06547100	-0.81975300

TS2 pro-*R*

C	2.16899000	0.66208000	-0.11179700
C	2.92007500	0.85364800	1.14961600
O	3.45418100	-0.26934400	1.62776300
C	4.01983600	-0.21812100	2.94726700
Ir	0.14497000	0.18509900	-0.05895700
Cl	0.20586400	-0.24428200	2.31460100
C	-1.82537500	-0.09754400	-0.04516300
C	-2.68261400	0.96425500	0.23170900
C	-4.06721500	0.77425000	0.23991600
C	-4.60605300	-0.48973200	-0.04554500
C	-3.73234600	-1.55889900	-0.32209700
C	-2.35330900	-1.36028600	-0.31582600
C	-1.88292300	2.13823100	0.52378400
O	-2.36698500	3.29707600	0.94892900
C	-1.21741800	4.08573000	1.36849400
C	0.00321600	3.35196200	0.79100700
N	-0.58405800	2.06121800	0.41324600

C	-1.26192800	-2.28482000	-0.55470900
O	-1.42322400	-3.56891000	-0.84303700
C	-0.10180400	-4.08236800	-1.16191800
C	0.87149400	-3.00058300	-0.66959100
N	-0.02990100	-1.85436200	-0.48191400
C	-6.11448900	-0.74576500	-0.07097100
C	1.64513100	-3.34132500	0.60918700
C	0.67872800	4.04527500	-0.39616700
Cl	0.07485100	0.63887100	-2.42808200
O	2.96978700	1.93454400	1.70693200
H	-4.13617300	-2.54743700	-0.54080000
H	-4.72126300	1.61325500	0.46810200
C	-6.46995000	-1.83650600	0.94517500
C	-6.52872400	-1.20459500	-1.47394500
C	-6.91917000	0.50412000	0.27674300
H	2.14188000	-2.40652500	0.92129800
H	-1.22113200	4.10422200	2.46306900
H	-1.35551600	5.10284100	0.99083700
H	-0.06213800	-4.22562800	-2.24659700
H	0.00984800	-5.05148400	-0.66718000
H	1.59519900	-2.75712000	-1.46011100
H	0.76314400	3.16957500	1.56392500
H	1.42002100	3.32608100	-0.77854800
H	3.32481500	0.31159300	3.60920200
H	4.95584000	0.35391400	2.91194300
H	-7.61099600	-1.38879600	-1.50966400
H	-6.02524900	-2.13321000	-1.76987100
H	-6.28980100	-0.43975800	-2.22439000
H	-7.55177300	-2.02586400	0.93455100
H	-6.18856600	-1.53385200	1.96220100
H	-5.96720100	-2.78624700	0.72586100
H	-7.99055400	0.26885000	0.24946600
H	-6.74309300	1.31952800	-0.43672700
H	-6.68876800	0.87243500	1.28481600
C	1.42442900	5.28269200	0.08296200
H	1.95717400	5.76250400	-0.74663800
H	0.73761500	6.03085300	0.50358600
H	2.16055000	5.02964700	0.85660800
C	-0.29032300	4.36627300	-1.52381700
H	0.24853600	4.78953600	-2.38002300
H	-0.81064100	3.46592100	-1.87350000
H	-1.04197500	5.10796000	-1.21900200
C	2.71460300	-4.38244000	0.30816500
H	3.32773500	-4.57598600	1.19682300
H	2.26913900	-5.34092100	0.00545700
H	3.38497700	-4.06071500	-0.50090000
C	0.74039300	-3.79317900	1.74629300
H	1.32707200	-3.93873600	2.66198200
H	-0.03088700	-3.04678100	1.96915100
H	0.25259500	-4.75292300	1.52511300
C	4.23758900	-1.64214200	3.37874200
H	4.70284800	-1.67319700	4.36935500
H	3.28098800	-2.17678500	3.43152500
H	4.89187400	-2.16948300	2.67437100

H	2.48480700	1.40557500	-0.85894500
O	4.90915300	-0.10411900	-1.14342900
C	3.80822100	-0.88532400	-1.23808000
C	3.41165800	-1.01244800	-2.68564400
C	3.90311500	0.31913500	-3.24820400
C	5.11569600	0.61767500	-2.38189200
H	5.24352900	1.67763700	-2.14012100
H	2.81920500	-0.36673000	-0.68461500
H	6.04948800	0.24282800	-2.81956900
H	3.12683000	1.08338900	-3.11657000
H	4.15573900	0.26972200	-4.31032400
H	3.95176800	-1.86569600	-3.11990100
H	2.33557900	-1.16543700	-2.82371000
H	3.87932800	-1.77661700	-0.60141700

TS3 pro-*S*

C	1.82574200	0.43358100	0.17313000
C	2.48459500	0.59898700	1.48835900
O	2.93710900	-0.55303200	1.99212000
C	3.41799200	-0.53742200	3.34571500
Ir	-0.21099000	0.10735200	0.04687600
Cl	-0.36109100	-0.36597000	2.40965700
C	-2.19604400	-0.01943700	-0.10800200
C	-2.98912600	1.10577800	0.11631500
C	-4.37516400	1.02233700	0.00797900
C	-4.98866300	-0.19619900	-0.34237900
C	-4.18502600	-1.32340800	-0.56853600
C	-2.79545800	-1.23025000	-0.44397000
C	-2.12917100	2.21071100	0.49554700
O	-2.56269500	3.39870900	0.89172300
C	-1.40074300	4.10168600	1.41475400
C	-0.18996300	3.26951600	0.96156700
N	-0.83597200	2.03354500	0.50058000
C	-1.76051400	-2.22995600	-0.62048100
O	-1.98448300	-3.49080200	-0.96464200
C	-0.67857000	-4.08299500	-1.20664500
C	0.32648500	-3.08748300	-0.60712200
N	-0.51164500	-1.89289400	-0.43662700
C	-6.51343800	-0.24386400	-0.46181700
C	0.97725700	-3.51901400	0.71248700
C	0.67035200	3.91441900	-0.12817600
Cl	-0.04118700	0.58886900	-2.30918800
O	2.55524400	1.67132400	2.05687500
H	-4.63529200	-2.27631400	-0.83865400
H	-4.98748100	1.90407800	0.19722600
C	-6.97415700	0.75258900	-1.53139600
C	-7.14448000	0.12942300	0.88397700
C	-7.02070900	-1.62865100	-0.85540000
H	1.50645200	-2.62752000	1.09126900
H	-1.50829200	4.13773700	2.50334000
H	-1.42181200	5.12088200	1.01838300
H	-0.56848100	-4.19179600	-2.29044800
H	-0.66871700	-5.07350900	-0.74312700
H	1.11738400	-2.85812700	-1.33418100

H	0.46132400	3.02044500	1.81066700
H	1.40448500	3.14693800	-0.41990900
H	2.69797700	0.00382100	3.97061400
H	4.37134800	0.00736100	3.38046100
H	-8.24007700	0.10603700	0.80913100
H	-6.85647100	1.13651400	1.20924100
H	-6.84370500	-0.57620400	1.66923900
H	-8.06738200	0.72162600	-1.63307000
H	-6.53644300	0.51161300	-2.50881400
H	-6.69488700	1.78373800	-1.28303500
H	-8.11544000	-1.60974500	-0.92704600
H	-6.75309700	-2.39098100	-0.11230800
H	-6.63181600	-1.94683400	-1.83148200
C	1.43253500	5.09872300	0.44954400
H	2.09382100	5.54123700	-0.30466300
H	0.75074800	5.89141800	0.78888300
H	2.05059800	4.79935900	1.30560700
C	-0.12829600	4.30410400	-1.36241500
H	0.54038300	4.69391000	-2.13956200
H	-0.66058700	3.44351600	-1.78598000
H	-0.86015600	5.09458600	-1.14397800
C	2.00083300	-4.61719000	0.45682700
H	2.54763200	-4.85823800	1.37694500
H	1.51916900	-5.54306100	0.11132000
H	2.73515400	-4.32391600	-0.30606800
C	-0.03672500	-3.94120800	1.76535600
H	0.47023000	-4.16123100	2.71310600
H	-0.76629000	-3.14707700	1.96219500
H	-0.57594100	-4.85284100	1.47162100
C	3.56270000	-1.97532700	3.76155600
H	3.96695800	-2.04169800	4.77680900
H	2.58678900	-2.47605900	3.74528400
H	4.23906300	-2.51205800	3.08465300
H	2.65947200	-0.03095900	-3.29993600
H	2.25972800	1.12284300	-0.56776300
C	3.42206400	-1.28144400	-0.81382200
O	3.00849500	-1.65417400	-2.05940200
C	3.48976000	-0.70344600	-3.02786500
C	4.59730400	0.00547500	-2.32181200
C	4.56299200	-0.36518200	-0.97696700
H	2.45123900	-0.67541100	-0.30090200
H	3.47260000	-2.11858500	-0.10661800
H	3.79628200	-1.25952900	-3.92059000
C	5.55097400	0.90150500	-2.78259500
H	5.58683600	1.19637300	-3.82882700
C	5.47189000	0.14557600	-0.05650300
H	5.43424900	-0.15052200	0.99053600
C	6.43494800	1.03630900	-0.52066900
H	7.16807800	1.44560600	0.17006700
C	6.47107000	1.41002900	-1.86678100
H	7.23378600	2.10681600	-2.20669100

TS4 pro-*R*

C	-1.90292200	0.69596900	-0.37666300
C	-2.46232800	0.95216300	-1.72266000
O	-2.84905900	-0.15308600	-2.35829700
C	-3.21125000	-0.01413900	-3.74039900
Ir	0.10411300	0.20744400	-0.17496400
Cl	0.39233900	-0.10385900	-2.55386200
C	2.05077700	-0.11826200	0.09508800
C	2.95649700	0.93922900	0.00848000
C	4.31589500	0.71674300	0.21182500
C	4.78692800	-0.57454200	0.52212900
C	3.86947700	-1.63104000	0.61592600
C	2.50788700	-1.39835500	0.39727600
C	2.22780400	2.14265000	-0.34922700
O	2.79117100	3.30676100	-0.63945200
C	1.73609700	4.14196800	-1.19426600
C	0.42693300	3.39957200	-0.87755200
N	0.92765800	2.08773000	-0.44943100
C	1.37736300	-2.30499700	0.42377500
O	1.46114400	-3.60493900	0.67526000
C	0.09188300	-4.08769800	0.75050200
C	-0.74836900	-2.97901900	0.10396600
N	0.18086600	-1.84244000	0.16731300
C	6.28653000	-0.77337000	0.75131200
C	-1.22396100	-3.25931700	-1.32670600
C	-0.45673000	4.05680500	0.18570000
Cl	-0.13370100	0.57055800	2.19530300
O	-2.48323400	2.06926400	-2.20695700
H	4.20941200	-2.63655400	0.85488800
H	5.01959300	1.54549200	0.13308900
C	6.74120000	0.10066600	1.92550800
C	7.05648600	-0.36901700	-0.51090300
C	6.63649700	-2.22352900	1.07633200
H	-1.63369300	-2.30591700	-1.70073600
H	1.92515900	4.22872400	-2.26884000
H	1.81807600	5.13081300	-0.73426600
H	-0.14435300	-4.23158800	1.81068800
H	0.04714600	-5.05103500	0.23534300
H	-1.63072900	-2.75899700	0.72101500
H	-0.17636900	3.25644300	-1.78540700
H	-1.26523100	3.33574700	0.38311400
H	-2.43971500	0.57624700	-4.24861000
H	-4.15808000	0.53749400	-3.80751100
H	8.13539100	-0.50569300	-0.35711300
H	6.89136800	0.68223000	-0.77624900
H	6.75638300	-0.98344800	-1.36973200
H	7.81565100	-0.03575100	2.10802900
H	6.20446600	-0.16646500	2.84524200
H	6.57062200	1.16730400	1.73510400
H	7.71937500	-2.31220100	1.22970200
H	6.36262100	-2.90591800	0.26125600
H	6.14543700	-2.56893500	1.99532900
C	-1.08391300	5.32535400	-0.37522000
H	-1.76565400	5.77786300	0.35457100

H	-0.32169800	6.07920100	-0.61800100
H	-1.65595200	5.11865600	-1.28877700
C	0.27948200	4.31757200	1.49074800
H	-0.41131800	4.71705900	2.24312000
H	0.71439600	3.39641700	1.89792600
H	1.08175500	5.05872600	1.36704900
C	-2.33882600	-4.29656500	-1.30505100
H	-2.72855100	-4.47025600	-2.31537300
H	-1.98109400	-5.26532100	-0.92741900
H	-3.17968800	-3.98572200	-0.66974200
C	-0.09675600	-3.68307600	-2.25691000
H	-0.47529300	-3.79503200	-3.28084000
H	0.70453700	-2.93561600	-2.28794900
H	0.33178400	-4.65215100	-1.96493700
C	-3.32104700	-1.40401600	-4.30405600
H	-3.62302000	-1.36455800	-5.35585200
H	-2.35483400	-1.91985000	-4.24227300
H	-4.06566800	-1.99185000	-3.75444900
H	-2.32382900	1.39367400	0.36372900
O	-4.78494300	-0.12732000	-0.11517200
C	-3.75022900	-0.85411200	0.39474200
C	-3.81900300	-0.76903400	1.86427200
C	-4.76157800	0.21248500	2.17413500
C	-5.33586600	0.73012600	0.89973000
H	-5.04861900	1.77142700	0.69164400
H	-2.63641900	-0.36251400	0.06143700
H	-6.42844000	0.66104500	0.83895700
H	-3.62254400	-1.81760500	-0.11627600
C	-5.02553900	0.55450100	3.49244700
H	-5.75940100	1.31843900	3.73880500
C	-3.11608200	-1.43384100	2.86426600
H	-2.36917900	-2.18885900	2.62704300
C	-4.32838800	-0.11474700	4.49590700
H	-4.52117000	0.13132600	5.53758700
C	-3.38656800	-1.09967200	4.18657800
H	-2.85677700	-1.60679500	4.98903600

VI. Linear Regression Mathematical Modeling Details

Modeling was performed using MATLAB[28] and a script similar to previous studies.[29] Initial modeling led to a model that incorporated Charton values and Sterimol B_5. Details of this model are:

$$y = 0.23 + 1.20\ Charton + 0.44\ B5 + 1.06\ (Charton \times B5)$$

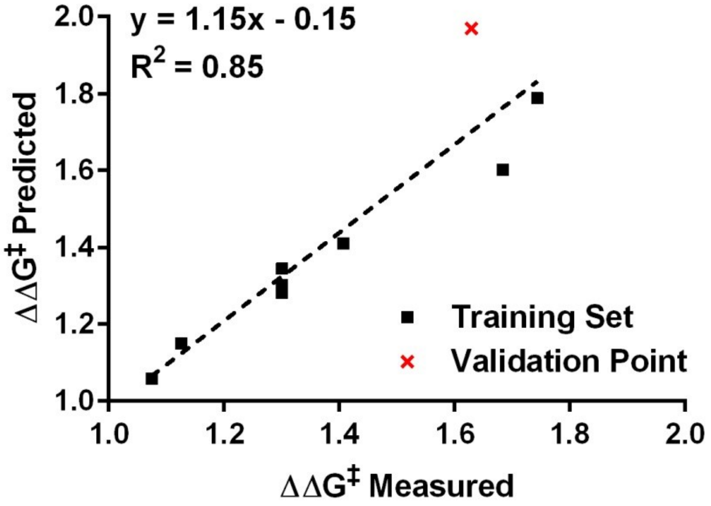

This model predicted a ΔΔG‡ of 1.97, equivalent to an enantiomeric ratio of 96.5 to 3.5. Because this model did not predict the measured ratio well, modeling was re-evaluated and a new model was found. This model is presented in the text, and the details repeated here:

$$y = 0.47 + 1.26(Austel) - 2.52(Austel \times E_s) - 2.54(E_s)^2$$

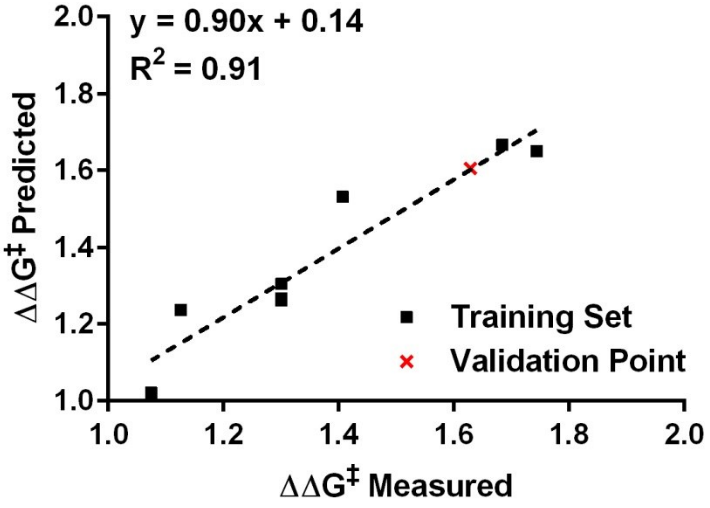

The values used to build these models are in Table S4. All values were collected from the same source.[30]

Table S4. Ir(III)-Phebim catalyst steric parameters.

Group	*R*	*S*	Charton	B_1	B_5	L	Austel	E_s
Bn	86	14	0.7	1.52	6.02	4.62	4.00	-1.61
^{n}Bu	87	13	0.68	1.52	4.54	6.17	3.00	-1.63
*i*Pr	90	10	0.76	1.9	3.17	4.11	3.00	-1.71
Et	90	10	0.56	1.52	3.17	4.11	2.00	-1.31
*c*Hex	90	10	0.87	1.91	3.49	6.17	3.00	-2.03
*s*Bu	91.5	8.5	1.02	1.9	3.49	4.92	4.00	-2.37
*i*Bu	94.5	5.5	0.98	1.52	4.45	4.92	4.00	-2.17
CH_2Hex	95	5	0.97	1.52	5.42	6.09	4.00	-2.22
CH_2*t*Bu	94	6	1.34	1.52	4.18	4.89	5.00	-2.98

The measured versus predicted values from both models are in Table S5. $CH_2{}^t$Bu was predicted after the model had been built with the initial eight groups.

Table S5. Comparison of measured and predicted $\Delta\Delta G^‡$ values from the mathematical model.

Group	Measured $\Delta\Delta G^‡$ (kcal/mol)	Predicted $\Delta\Delta G^‡$ (kcal/mol) Charton and Sterimol	Predicted $\Delta\Delta G^‡$ (kcal/mol) Austel and E_s
Bn	1.07	1.06	1.02
*n*Bu	1.13	1.15	1.24
*i*Pr	1.30	1.30	1.30
Et	1.30	1.28	1.26
*c*Hex	1.30	1.34	1.27
*s*Bu	1.41	1.41	1.53
*i*Bu	1.68	1.60	1.67
CH_2Hex	1.74	1.79	1.65
CH_2*t*Bu	1.63	1.97	1.61

VII. References

(1) Wu, L.-Y.; Hao, X.-Q.; Xu, Y.-X.; Jia, M.-Q.; Wang, Y.-N.; Gong, J.-F.; Song, M.-P. *Organometallics* **2009**, *28*, 3369.

(2) Davies, I. W.; Gerena, L.; Lu, N.; Larsen, R. D.; Reider, P. J. *J. Org. Chem.* **1996**, *61*, 9629.

(3) Christoffers, J.; Schuster, K. *Chirality* **2003**, *15*, 777.

(4) Guaragna, A.; Pedatella, S.; Pinto, V.; Palumbo, G. *Synthesis* **2006**, *2006*, 4013.

(5) Ma, K.; You, J. *Chem. Eur. J.* **2007**, *13*, 1863.

(6) Veri, E. Ph.D. Dissertation, RWTH Aachen University, **2006**.

(7) Pryor, K. E.; Rebek, J. *Org. Lett.* **1999**, *1*, 39.

(8) Norris, J. L.; Porter, N. A.; Caprioli, R. M. *Anal. Chem.* **2005**, *77*, 5036.

(9) Toma, T.; Shimokawa, J.; Fukuyama, T. *Org. Lett.* **2007**, *9*, 3195.

(10) Candish, L.; Lupton, D. W. *J. Am. Chem. Soc.* **2013**, *135*, 58.

(11) Martín-Matute, B.; Cárdenas, D. J.; Echavarren, A. M. *Angew. Chem., Int. Ed.* **2001**, *40*, 4754.

(12) García, D.; Foubelo, F.; Yus, M. *Tetrahedron* **2008**, *64*, 4275.

(13) Murphy, J. M.; Liao, X.; Hartwig, J. F. *J. Am. Chem. Soc.* **2007**, *129*, 15434.

(14) Mo, F.; Jiang, Y.; Qiu, D.; Zhang, Y.; Wang, J. *Angew. Chem. Int. Ed.* **2010**, *49*, 1846.

(15) Favor, D. A.; Johnson, D. S.; Powers, J. J.; Li, T.; Madabattula, R. *Tetrahedron Lett.* **2007**, *48*, 3039.

(16) Ling, X.; Xiong, Y.; Huang, R.; Zhang, X.; Zhang, S.; Chen, C. *J. Org. Chem.* **2013**, *78*, 5218.

(17) Piel, I.; Steinmetz, M.; Hirano, K.; Fröhlich, R.; Grimme, S.; Glorius, F. *Angew. Chem., Int. Ed.* **2011**, *50*, 4983.

(18) Briot, A.; Bujard, M.; Gouverneur, V.; Nolan, S. P.; Mioskowski, C. *Org. Lett.* **2000**, *2*, 1517.

(19) Nielsen, L.; Skrydstrup, T. *J. Am. Chem. Soc.* **2008**, *130*, 13145.

(20) Owens, C. P.; Varela-Alvarez, A.; Boyarskikh, V.; Musaev, D. G.; Davies, H. M. L.; Blakey, S. B. *Chem. Sci.* **2013**, *4*, 2590.

(21) Kornilova, T.; Ukolov, A.; Kostikov, R.; Zenkevich, I. *Russ. J. Gen. Chem.* **2012**, *82*, 1675.

(22) Hiroya, K.; Jouka, R.; Kameda, M.; Yasuhara, A.; Sakamoto, T. *Tetrahedron* **2001**, *57*, 9697.

(23) Davies, H. M. L.; Hansen, T.; Churchill, M. R. *J. Am. Chem. Soc.* **2000**, *122*, 3063.

(24) *Gaussian 09, Revision **D.01**,* Frisch, M. J.; Trucks, G. W.; Schlegel, H. B.; Scuseria, G. E.; Robb, M. A.; Cheeseman, J. R.; Scalmani, G.; Barone, V.; Mennucci, B.; Petersson, G. A.; Nakatsuji, H.; Caricato, M.; Li, X.; Hratchian, H. P.; Izmaylov, A. F.; Bloino, J.; Zheng, G.; Sonnenberg, J. L.; Hada, M.; Ehara, M.; Toyota, K.; Fukuda, R.; Hasegawa, J.; Ishida, M.; Nakajima, T.; Honda, Y.; Kitao, O.; Nakai, H.; Vreven, T.; Montgomery, J. A., Jr.; Peralta, J. E.; Ogliaro, F.; Bearpark, M.; Heyd, J. J.; Brothers, E.; Kudin, K. N.; Staroverov, V. N.; Kobayashi, R.; Normand, J.; Raghavachari, K.; Rendell, A.; Burant, J. C.; Iyengar, S. S.; Tomasi, J.; Cossi, M.; Rega, N.; Millam, M. J.; Klene, M.; Knox, J. E.; Cross, J. B.; Bakken, V.; Adamo,

C.; Jaramillo, J.; Gomperts, R.; Stratmann, R. E.; Yazyev, O.; Austin, A. J.; Cammi, R.; Pomelli, C.; Ochterski, J. W.; Martin, R. L.; Morokuma, K.; Zakrzewski, V. G.; Voth, G. A.; Salvador, P.; Dannenberg, J. J.; Dapprich, S.; Daniels, A. D.; Farkas, Ö.; Foresman, J. B.; Ortiz, J. V.; Cioslowski, J.; Fox, D. J. Gaussian, Inc., Wallingford CT, 2009.

(25) For M06 method see: (a) Zhao, Y.; Truhlar, D. G. *Theor. Chem. Acc.* **2008**, *120*, 215-241. (b) Zhao, Y.; Truhlar, D. G. *Acc. Chem. Res.* **2008**, *41*, 157-167. (c) Zhao, Y.; Truhlar, D. G. *J. Chem. Theory Comput.* **2009**, *5*, 324-333.

(26) (a) Hay, P. J.; Wadt, W. R. *J. Chem. Phys.* **1985**, *82* (1), 270-283; (b) Hay, P. J.; Wadt, W. R. *J. Chem. Phys.* **1985**, *82* (1), 299-310; (c) Wadt, W. R.; Hay, P. J. *J. Chem. Phys.* **1985**, *82* (1), 284-298.

(27) (a) Cances, E.; Mennucci, B.; Tomasi, J. *J. Chem. Phys.* **1997**, *107* (8), 3032-3041; (b) Mennucci, B.; Tomasi, J. *J. Chem. Phys.* **1997**, *106* (12), 5151-5158; (c) Scalmani, G.; Frisch, M. J. *J. Chem. Phys.* **2010**, *132* (11).

(28) *MATLAB and Statistics Toolbox Release 2014a*, The MathWorks, Inc.: Natick, Massachusetts, United States, 2014.

(29) (a) Milo, A.; Bess, E. N.; Sigman, M. S., Interrogating selectivity in catalysis using molecular vibrations. *Nature* **2014,** *507* (7491), 210-214; (b) Milo, A.; Neel, A. J.; Toste, F. D.; Sigman, M. S., A data-intensive approach to mechanistic elucidation applied to chiral anion catalysis. *Science* **2015,** *347* (6223), 737-743.

(30) Corwin Hansch, A. L., David Hoekman, *Exploring QSAR: Fundamentals and Applications in Chemistry and Biology*. American Chemical Society: Washington DC, 1995.

VIII. HPLC Data

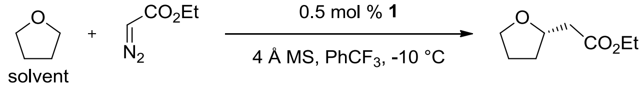

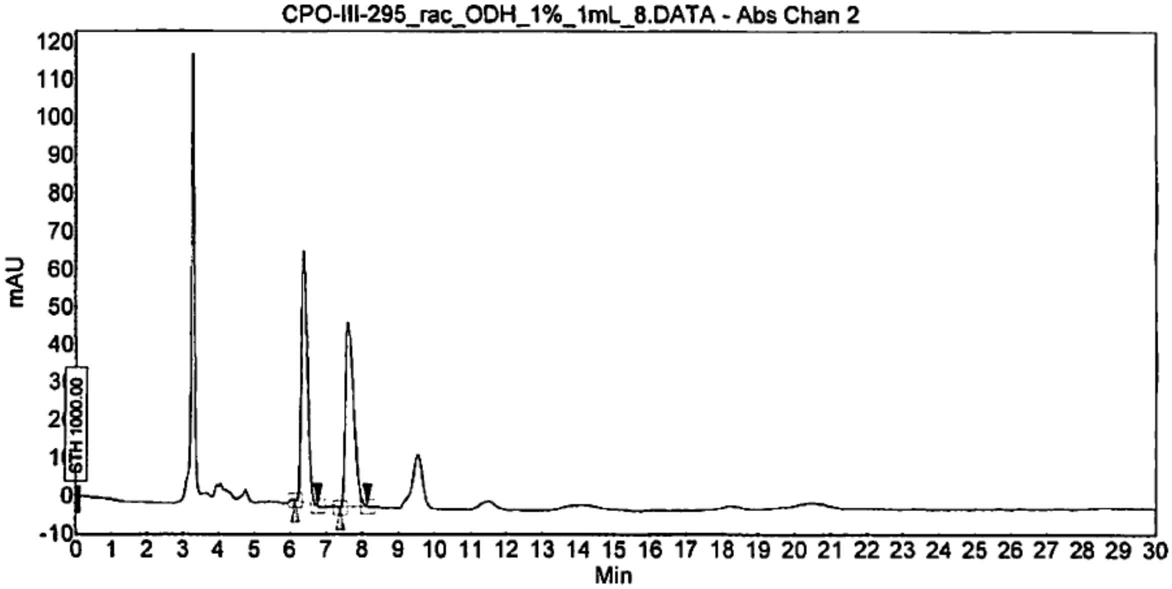

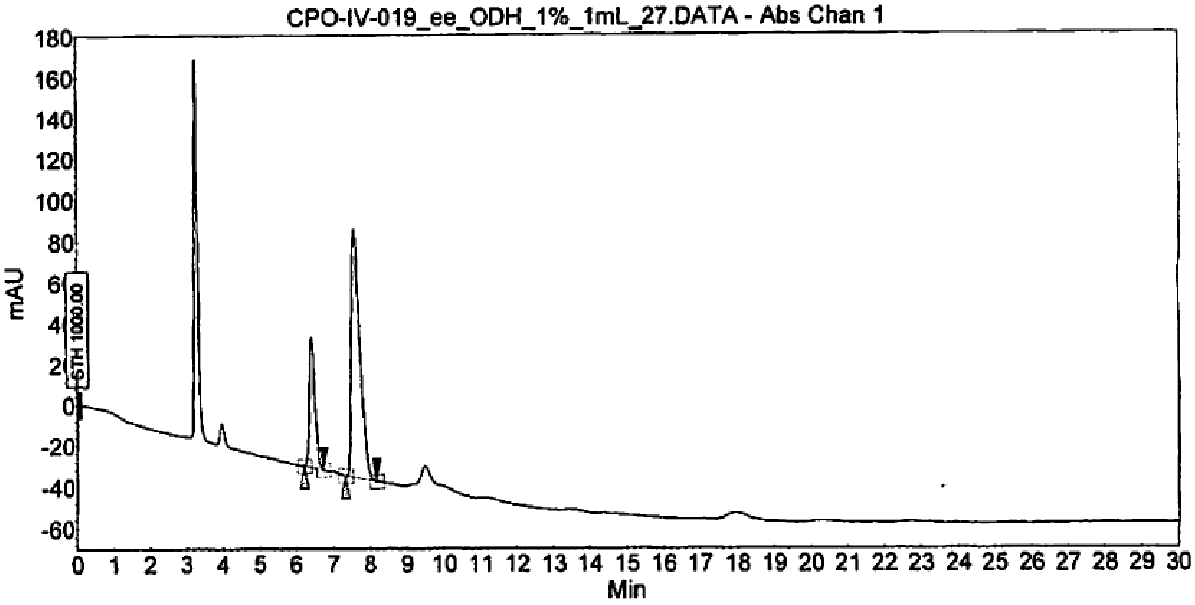

Peak results :

Index	Name	Time [Min]	Quantity [% Area]	Height [mAU]	Area [mAU.Min]	Area % [%]
1	UNKNOWN	6.43	25.47	63.8	11.0	25.470
2	UNKNOWN	7.58	74.53	121.1	32.3	74.530
Total			100.00	184.9	43.3	100.000

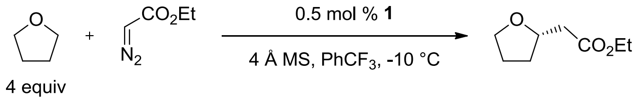

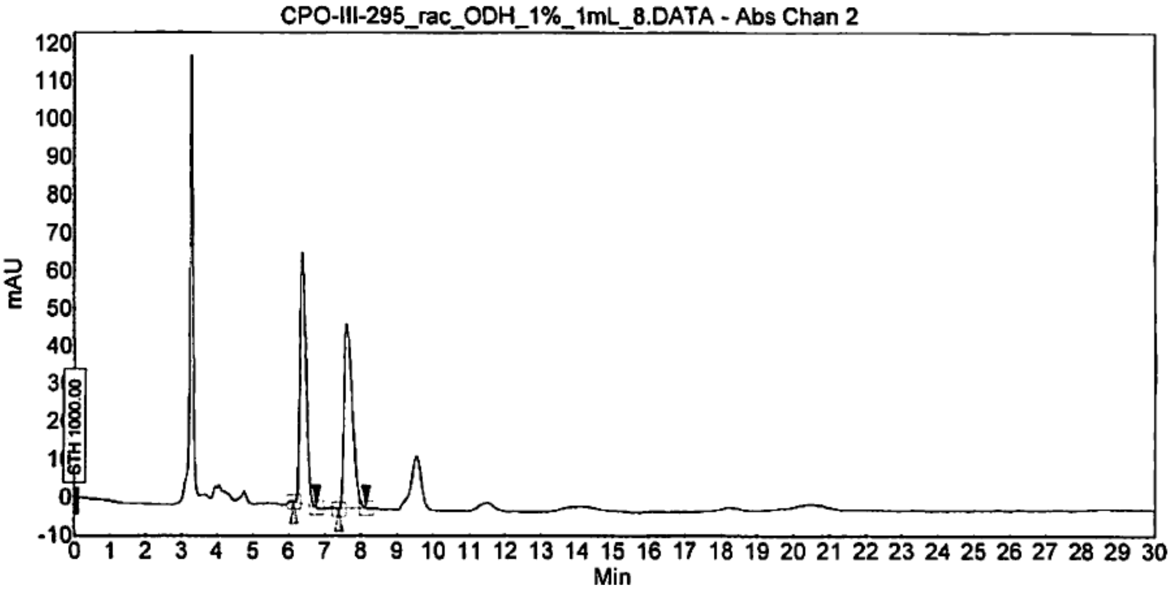

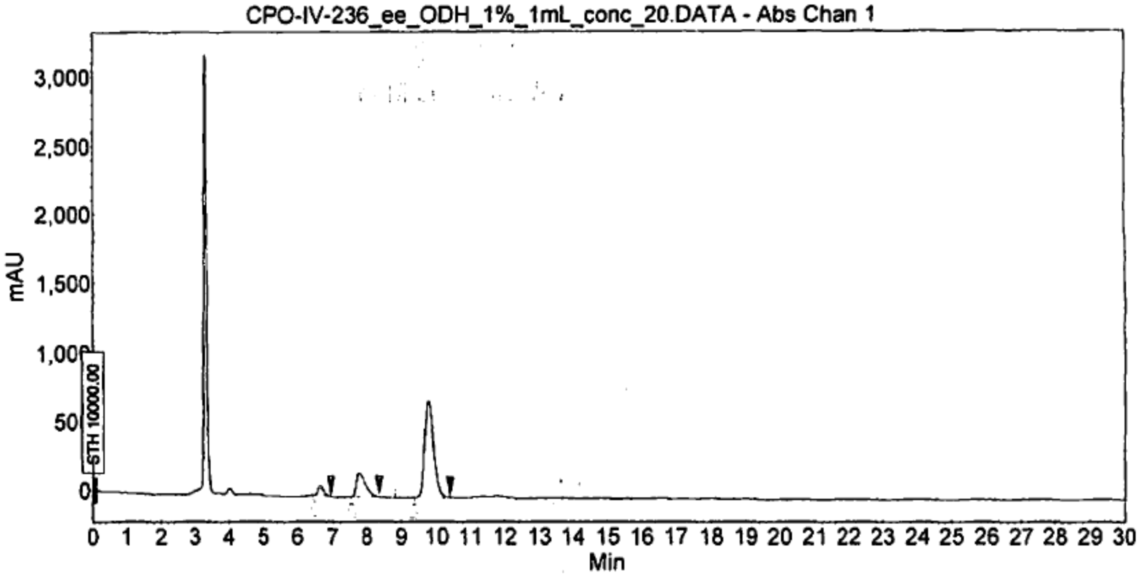

Peak results :

Index	Name	Time [Min]	Quantity [% Area]	Height [mAU]	Area [mAU.Min]	Area % [%]
1	UNKNOWN	6.64	4.77	73.7	13.2	4.772
2	UNKNOWN	7.75	19.86	174.6	54.8	19.865
3	UNKNOWN	9.78	75.36	705.8	207.8	75.363
Total			100.00	954.1	275.8	100.000

Catalyst Optimization Starting Materials and Side Products

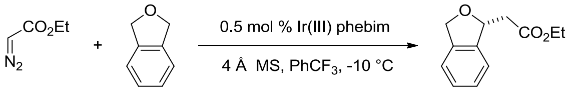

HPLC (Daicel OJ-H, 1 % IPA:HEX, 1 mL/min), λ 254 nm, t_R(phthalan) = 7.15 min, t_R(diethyl fumarate) = 7.36 min, t_R(ethyl diazoacetate) = 8.33 min, t_R(diethyl maleate) = 17.63 min.

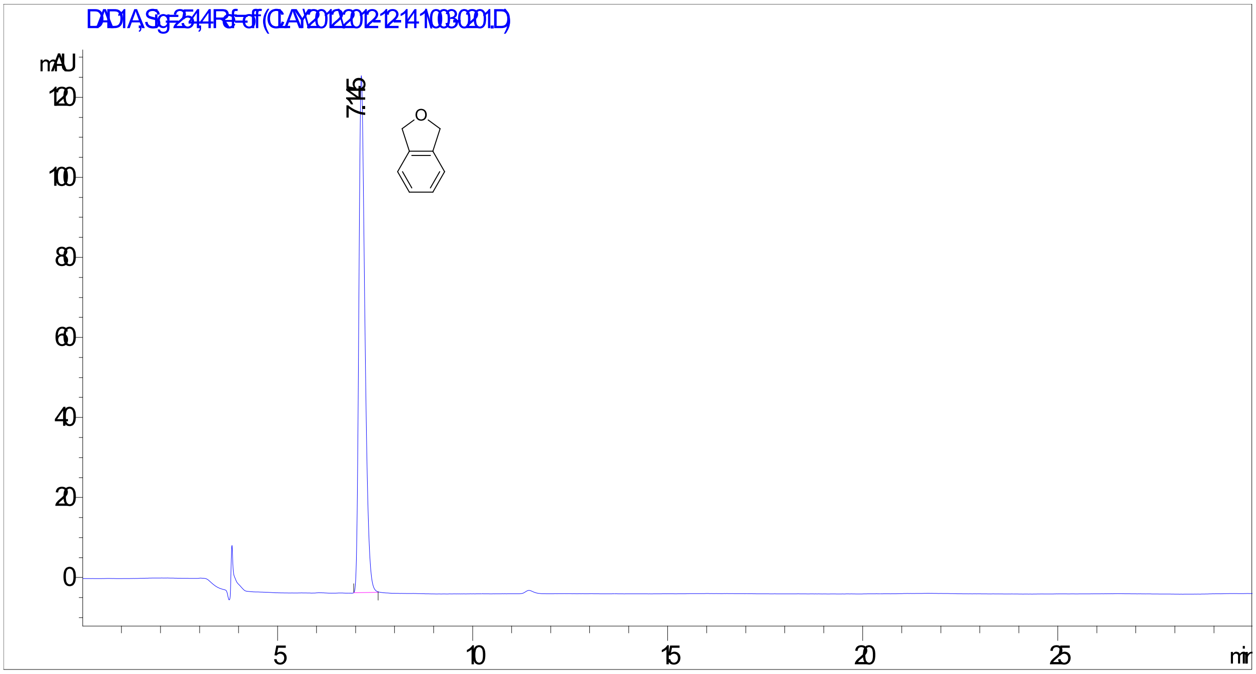

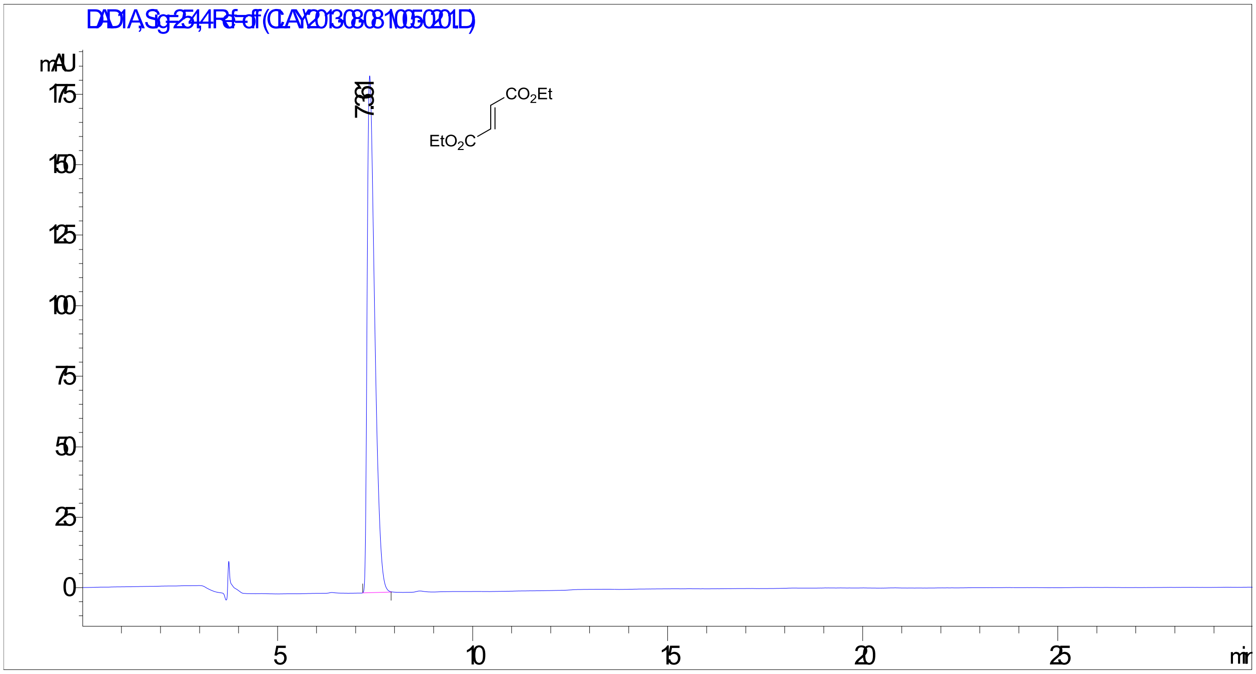

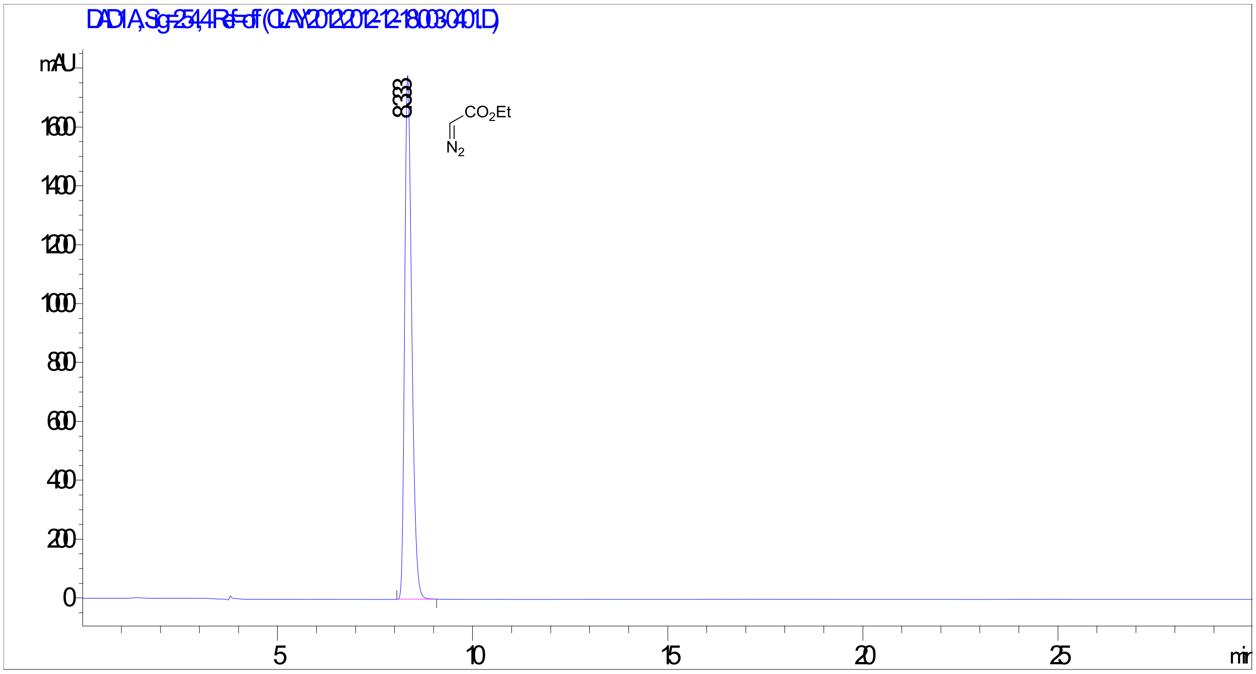
DAD1 A, Sig=254,4 Ref=off (CLAY\2012\2012-12-18\003-0401.D)
mAU
1600
1400
1200
1000
800
600
400
200
0
8.333
CO2Et
N2
5
10
15
20
25
min

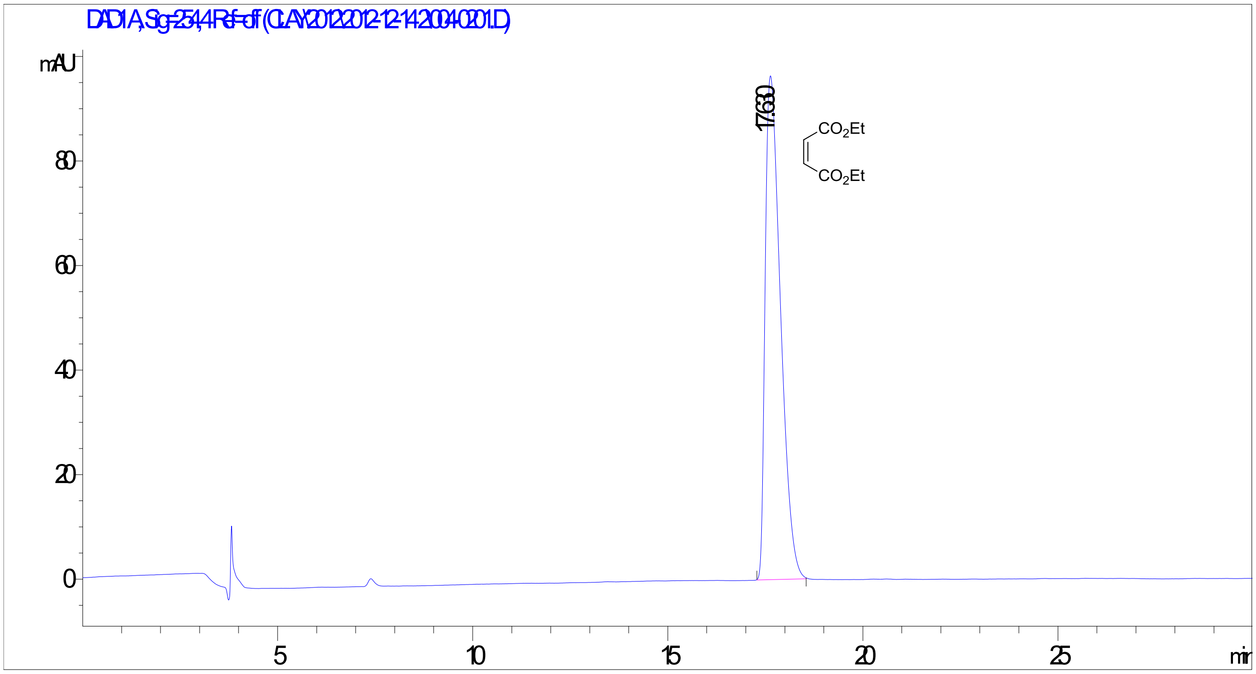
DAD1 A, Sig=254,4 Ref=off (CLAY\2012\2012-12-14\2004-0201.D)
mAU
80
60
40
20
0
17.630
CO2Et
CO2Et
5
10
15
20
25
min

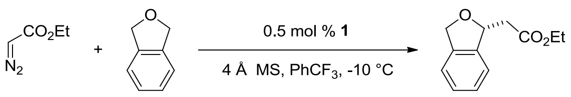

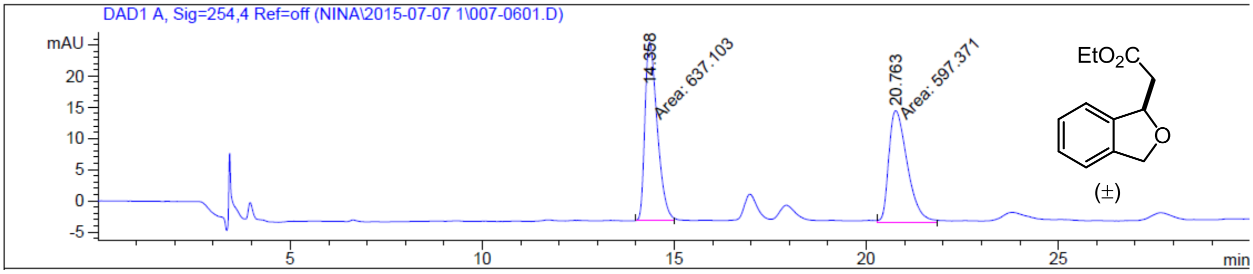

Signal 1: DAD1 A, Sig=254,4 Ref=off

Peak #	RetTime [min]	Type	Width [min]	Area [mAU*s]	Height [mAU]	Area %
1	14.358	MM	0.3721	637.10333	28.53423	51.6093
2	20.763	MM	0.5573	597.37115	17.86541	48.3907

Totals : 1234.47449 46.39964

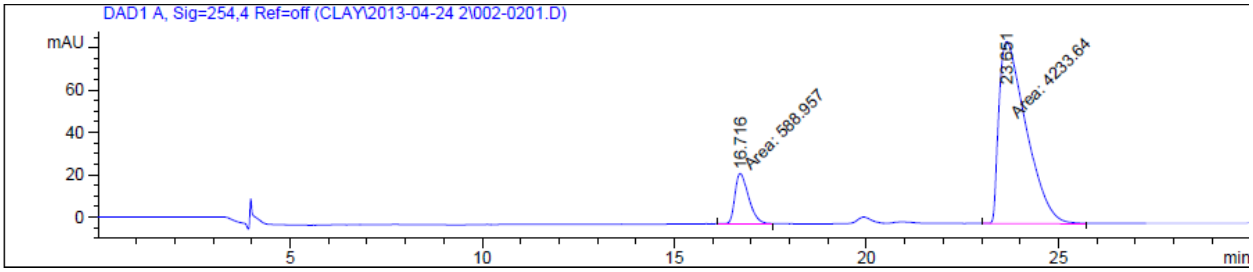

Signal 1: DAD1 A, Sig=254,4 Ref=off

Peak #	RetTime [min]	Type	Width [min]	Area [mAU*s]	Height [mAU]	Area %
1	16.716	MM	0.4118	588.95691	23.83761	12.2125
2	23.651	MM	0.8211	4233.63574	85.93274	87.7875

Totals : 4822.59265 109.77035

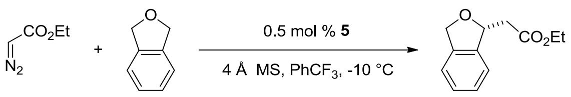

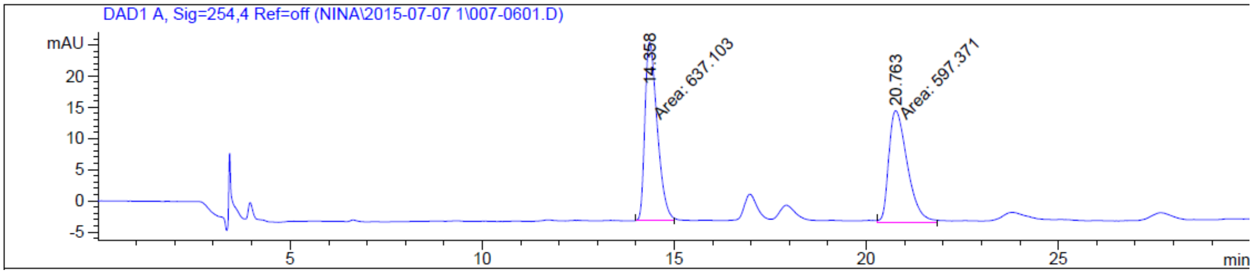

Signal 1: DAD1 A, Sig=254,4 Ref=off

Peak #	RetTime [min]	Type	Width [min]	Area [mAU*s]	Height [mAU]	Area %
1	14.358	MM	0.3721	637.10333	28.53423	51.6093
2	20.763	MM	0.5573	597.37115	17.86541	48.3907
Totals :				1234.47449	46.39964	

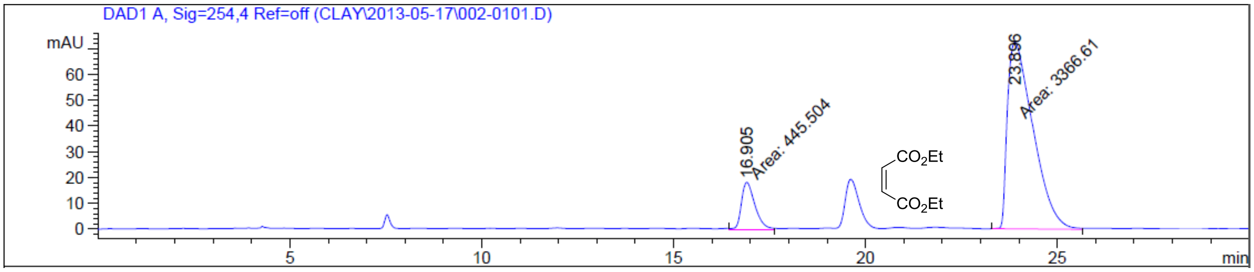

Signal 1: DAD1 A, Sig=254,4 Ref=off

Peak #	RetTime [min]	Type	Width [min]	Area [mAU*s]	Height [mAU]	Area %
1	16.905	MM	0.4060	445.50363	18.28667	11.6865
2	23.896	MM	0.7746	3366.60864	72.43604	88.3135
Totals :				3812.11227	90.72270	

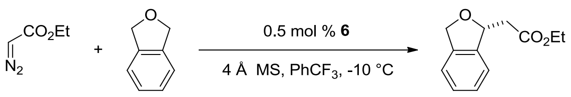

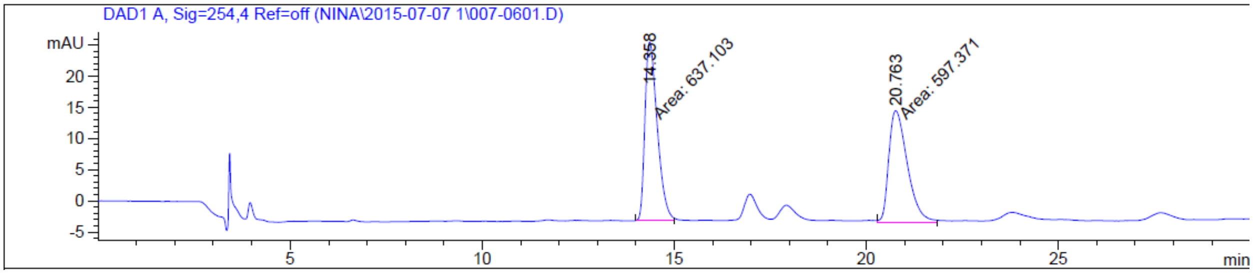

Signal 1: DAD1 A, Sig=254,4 Ref=off

Peak #	RetTime [min]	Type	Width [min]	Area [mAU*s]	Height [mAU]	Area %
1	14.358	MM	0.3721	637.10333	28.53423	51.6093
2	20.763	MM	0.5573	597.37115	17.86541	48.3907

Totals : 1234.47449 46.39964

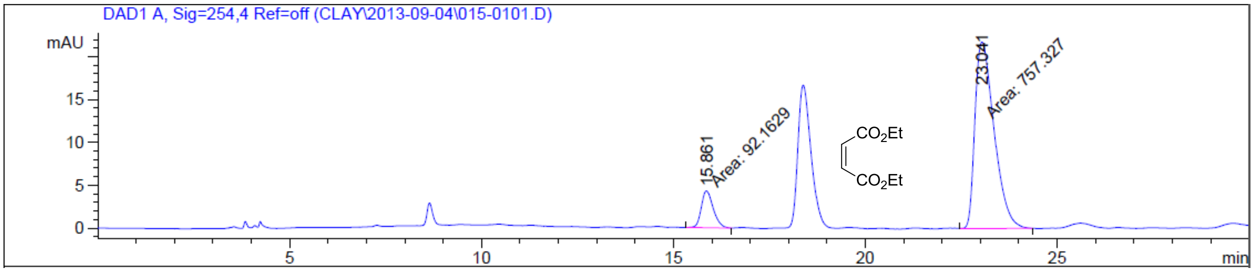

Signal 1: DAD1 A, Sig=254,4 Ref=off

Peak #	RetTime [min]	Type	Width [min]	Area [mAU*s]	Height [mAU]	Area %
1	15.861	MM	0.3580	92.16287	4.29099	10.8492
2	23.041	MM	0.5801	757.32733	21.75729	89.1508

Totals : 849.49020 26.04827

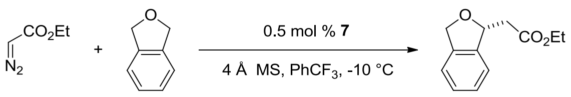

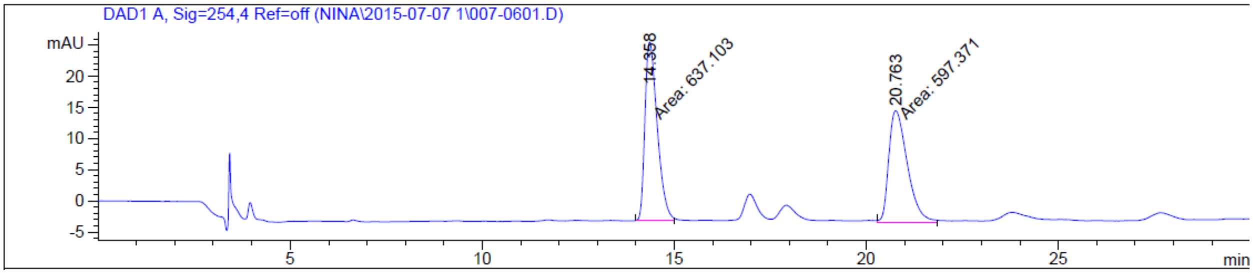

Signal 1: DAD1 A, Sig=254,4 Ref=off

Peak #	RetTime [min]	Type	Width [min]	Area [mAU*s]	Height [mAU]	Area %
1	14.358	MM	0.3721	637.10333	28.53423	51.6093
2	20.763	MM	0.5573	597.37115	17.86541	48.3907

Totals : 1234.47449 46.39964

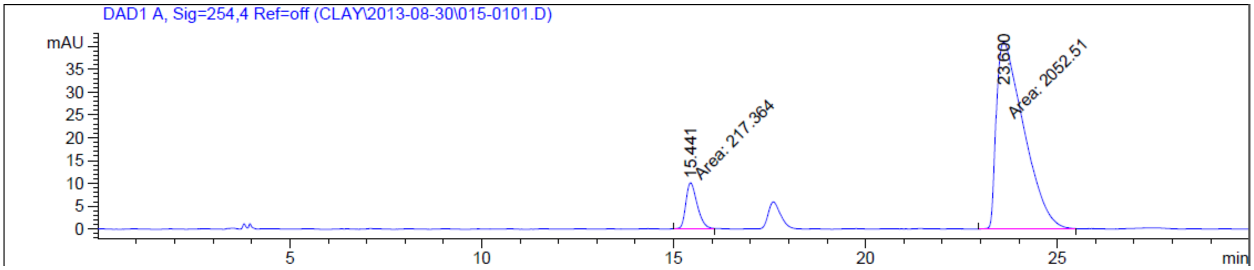

Signal 1: DAD1 A, Sig=254,4 Ref=off

Peak #	RetTime [min]	Type	Width [min]	Area [mAU*s]	Height [mAU]	Area %
1	15.441	MM	0.3544	217.36377	10.22279	9.5760
2	23.600	MM	0.8321	2052.51318	41.11047	90.4240

Totals : 2269.87695 51.33326

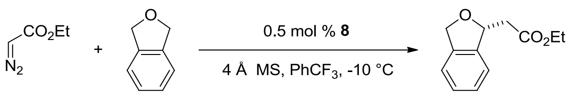

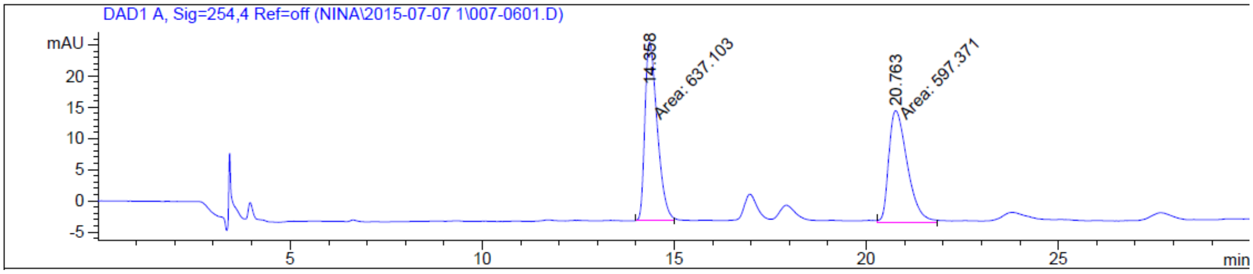

Signal 1: DAD1 A, Sig=254,4 Ref=off

Peak #	RetTime [min]	Type	Width [min]	Area [mAU*s]	Height [mAU]	Area %
1	14.358	MM	0.3721	637.10333	28.53423	51.6093
2	20.763	MM	0.5573	597.37115	17.86541	48.3907

Totals : 1234.47449 46.39964

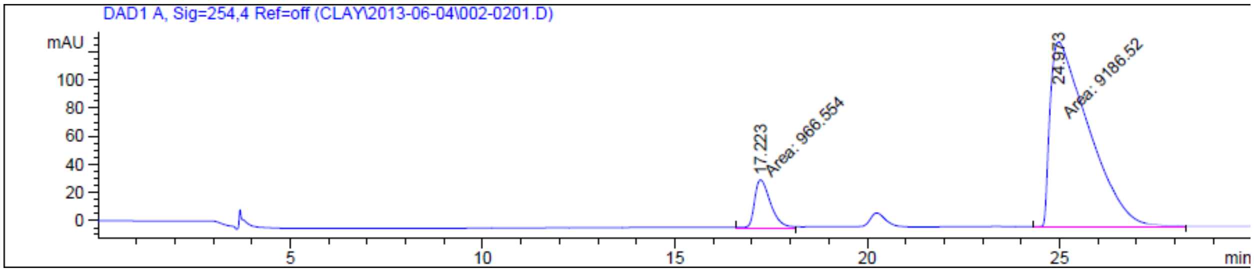

Signal 1: DAD1 A, Sig=254,4 Ref=off

Peak #	RetTime [min]	Type	Width [min]	Area [mAU*s]	Height [mAU]	Area %
1	17.223	MM	0.4743	966.55438	33.96584	9.5198
2	24.973	MM	1.1657	9186.52148	131.34679	90.4802

Totals : 1.01531e4 165.31262

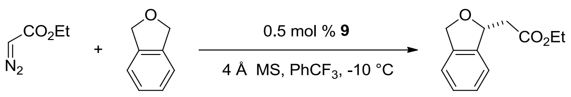

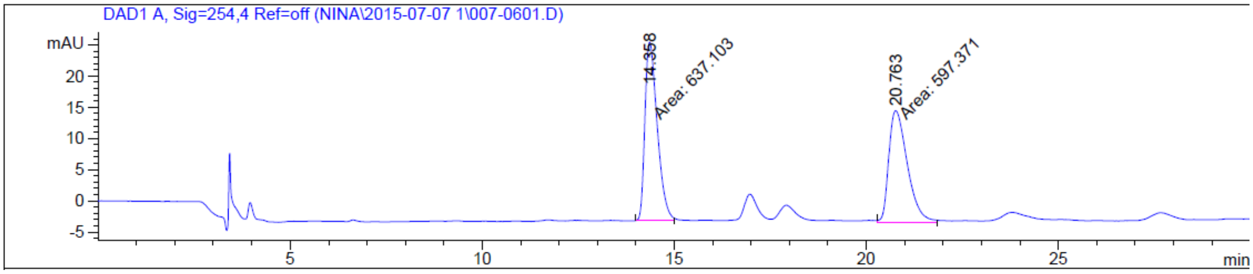

Signal 1: DAD1 A, Sig=254,4 Ref=off

Peak #	RetTime [min]	Type	Width [min]	Area [mAU*s]	Height [mAU]	Area %
1	14.358	MM	0.3721	637.10333	28.53423	51.6093
2	20.763	MM	0.5573	597.37115	17.86541	48.3907

Totals : 1234.47449 46.39964

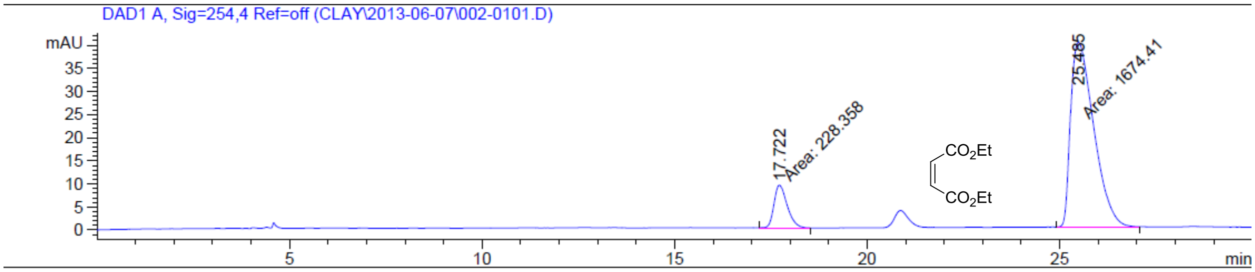

Signal 1: DAD1 A, Sig=254,4 Ref=off

Peak #	RetTime [min]	Type	Width [min]	Area [mAU*s]	Height [mAU]	Area %
1	17.722	MM	0.4066	228.35831	9.35954	12.0014
2	25.485	MM	0.6976	1674.41211	40.00420	87.9986

Totals : 1902.77042 49.36374

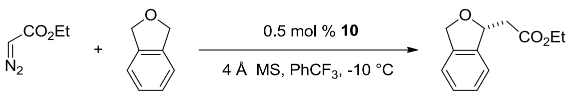

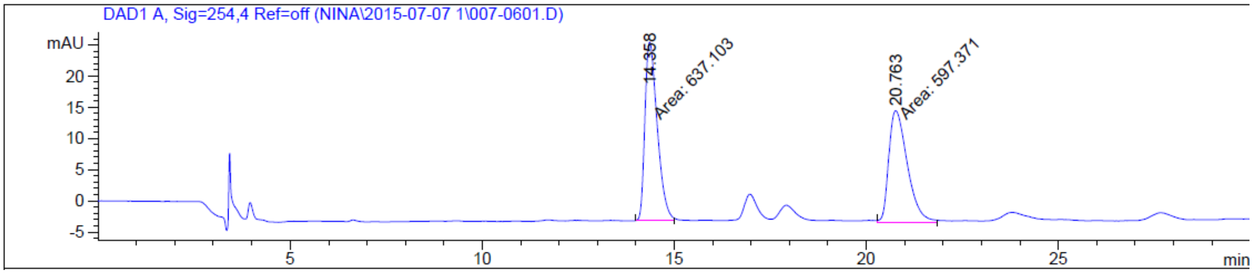

Signal 1: DAD1 A, Sig=254,4 Ref=off

Peak #	RetTime [min]	Type	Width [min]	Area [mAU*s]	Height [mAU]	Area %
1	14.358	MM	0.3721	637.10333	28.53423	51.6093
2	20.763	MM	0.5573	597.37115	17.86541	48.3907
Totals :				1234.47449	46.39964	

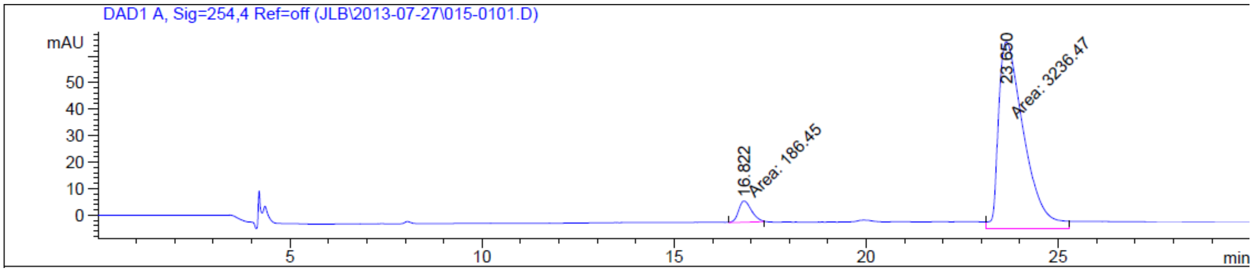

Signal 1: DAD1 A, Sig=254,4 Ref=off

Peak #	RetTime [min]	Type	Width [min]	Area [mAU*s]	Height [mAU]	Area %
1	16.822	MM	0.3869	186.44960	8.03215	5.4471
2	23.650	MM	0.7660	3236.47241	70.42204	94.5529
Totals :				3422.92201	78.45418	

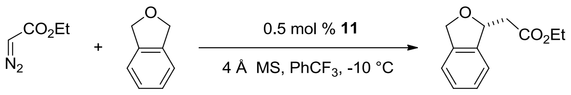

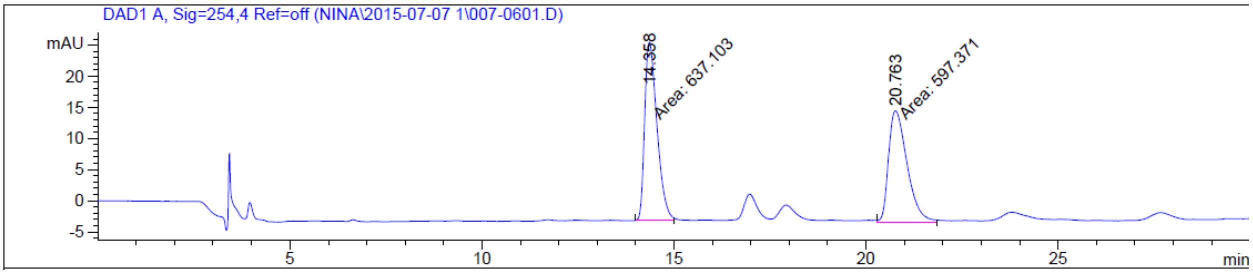

Signal 1: DAD1 A, Sig=254,4 Ref=off

Peak #	RetTime [min]	Type	Width [min]	Area [mAU*s]	Height [mAU]	Area %
1	14.358	MM	0.3721	637.10333	28.53423	51.6093
2	20.763	MM	0.5573	597.37115	17.86541	48.3907

Totals : 1234.47449 46.39964

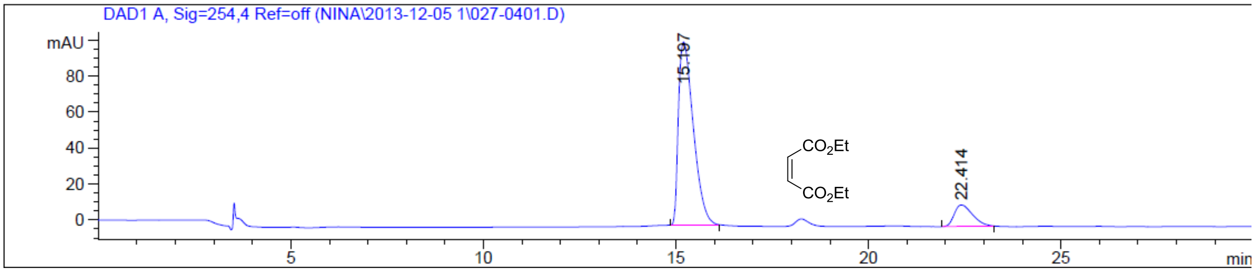

Signal 1: DAD1 A, Sig=254,4 Ref=off

Peak #	RetTime [min]	Type	Width [min]	Area [mAU*s]	Height [mAU]	Area %
1	15.197	BB	0.4221	2761.57886	101.93052	87.5291
2	22.414	BB	0.4955	393.46213	11.97085	12.4709

Totals : 3155.04099 113.90137

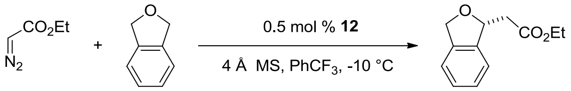

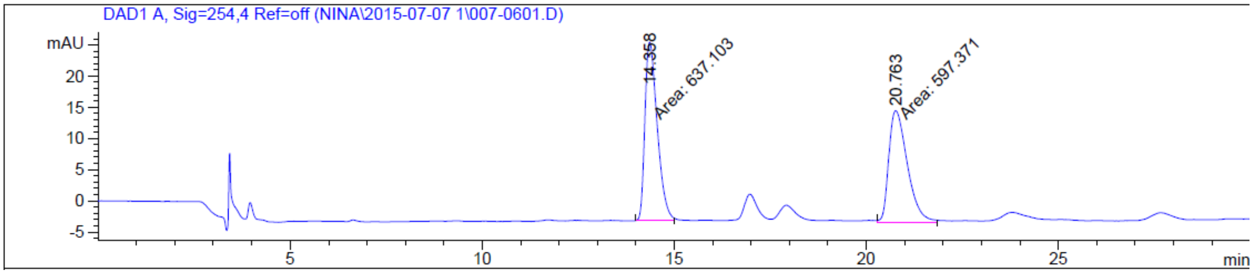

Signal 1: DAD1 A, Sig=254,4 Ref=off

Peak #	RetTime [min]	Type	Width [min]	Area [mAU*s]	Height [mAU]	Area %
1	14.358	MM	0.3721	637.10333	28.53423	51.6093
2	20.763	MM	0.5573	597.37115	17.86541	48.3907
Totals :				1234.47449	46.39964	

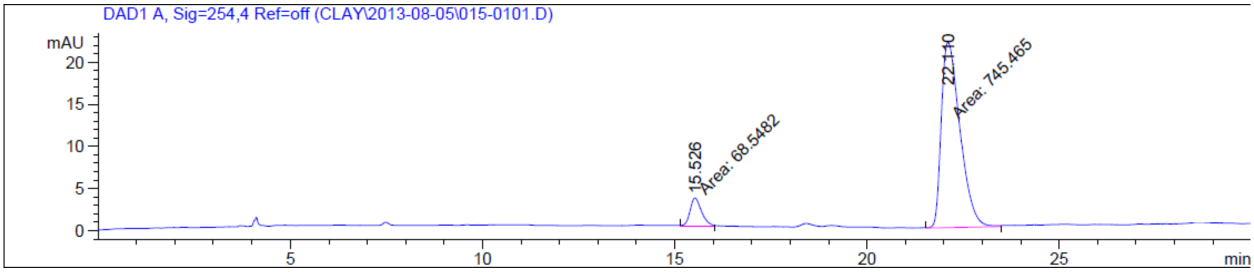

Signal 1: DAD1 A, Sig=254,4 Ref=off

Peak #	RetTime [min]	Type	Width [min]	Area [mAU*s]	Height [mAU]	Area %
1	15.526	MM	0.3446	68.54819	3.31566	8.4210
2	22.110	MM	0.5640	745.46497	22.02788	91.5790
Totals :				814.01316	25.34354	

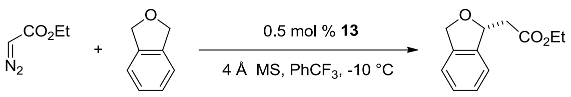

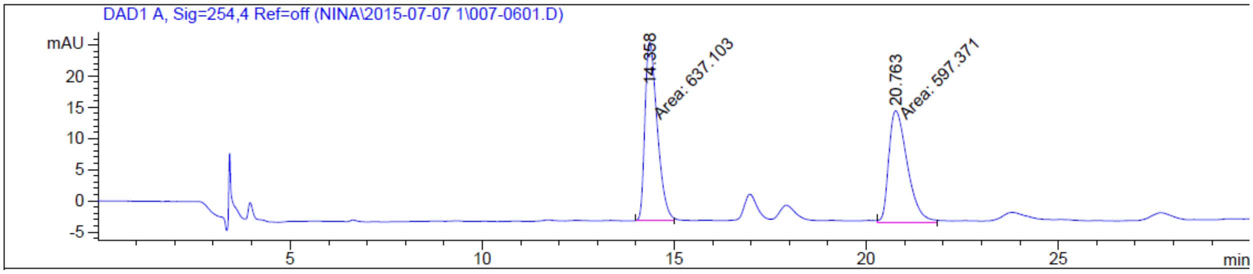

Signal 1: DAD1 A, Sig=254,4 Ref=off

Peak #	RetTime [min]	Type	Width [min]	Area [mAU*s]	Height [mAU]	Area %
1	14.358	MM	0.3721	637.10333	28.53423	51.6093
2	20.763	MM	0.5573	597.37115	17.86541	48.3907
Totals :				1234.47449	46.39964	

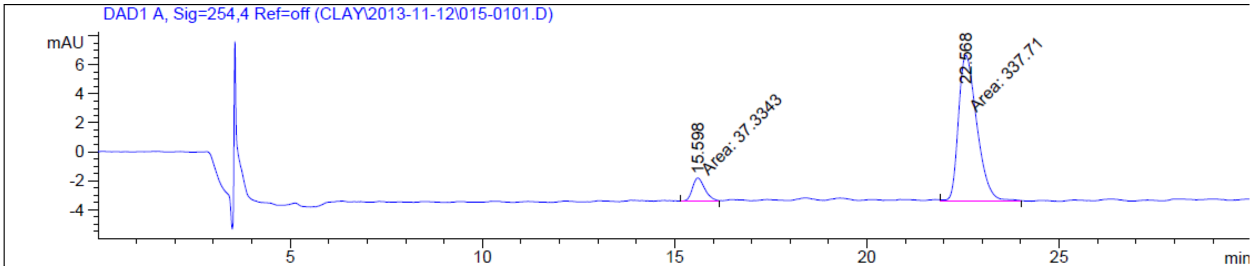

Signal 1: DAD1 A, Sig=254,4 Ref=off

Peak #	RetTime [min]	Type	Width [min]	Area [mAU*s]	Height [mAU]	Area %
1	15.598	MM	0.3850	37.33426	1.61613	9.9546
2	22.568	MM	0.5569	337.71008	10.10624	90.0454
Totals :				375.04435	11.72237	

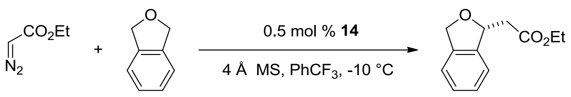

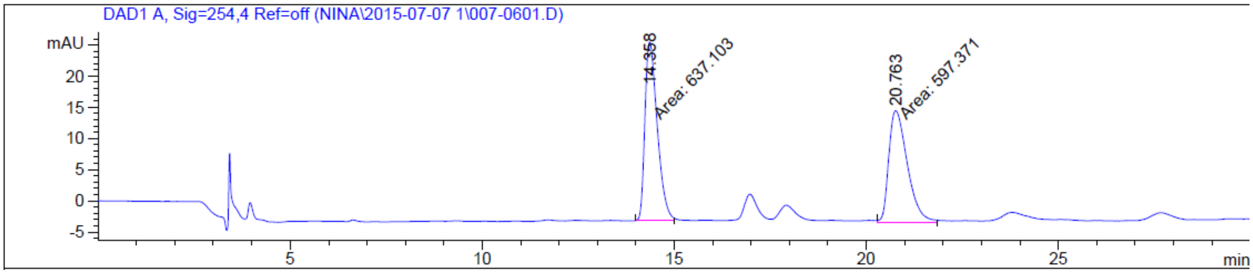

Signal 1: DAD1 A, Sig=254,4 Ref=off

Peak #	RetTime [min]	Type	Width [min]	Area [mAU*s]	Height [mAU]	Area %
1	14.358	MM	0.3721	637.10333	28.53423	51.6093
2	20.763	MM	0.5573	597.37115	17.86541	48.3907

Totals : 1234.47449 46.39964

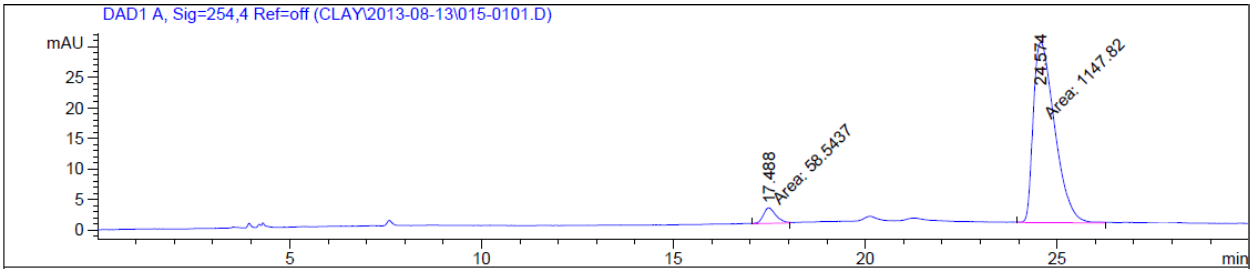

Signal 1: DAD1 A, Sig=254,4 Ref=off

Peak #	RetTime [min]	Type	Width [min]	Area [mAU*s]	Height [mAU]	Area %
1	17.488	MM	0.3842	58.54367	2.53953	4.8529
2	24.574	MM	0.6471	1147.81519	29.56103	95.1471

Totals : 1206.35886 32.10056

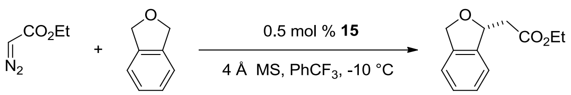

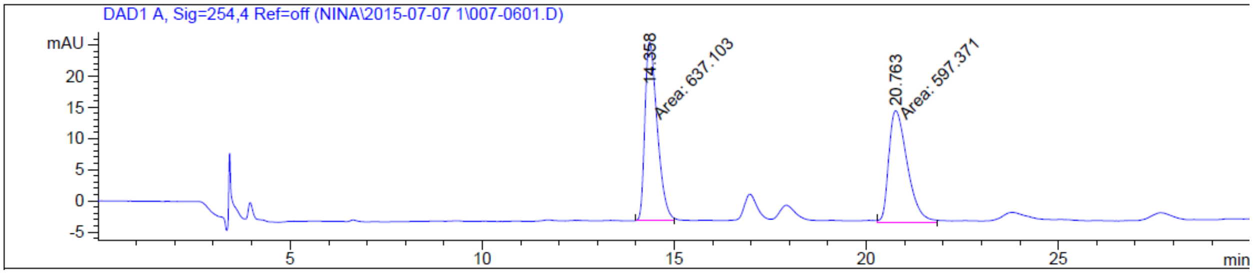

Signal 1: DAD1 A, Sig=254,4 Ref=off

Peak #	RetTime [min]	Type	Width [min]	Area [mAU*s]	Height [mAU]	Area %
1	14.358	MM	0.3721	637.10333	28.53423	51.6093
2	20.763	MM	0.5573	597.37115	17.86541	48.3907
Totals :				1234.47449	46.39964	

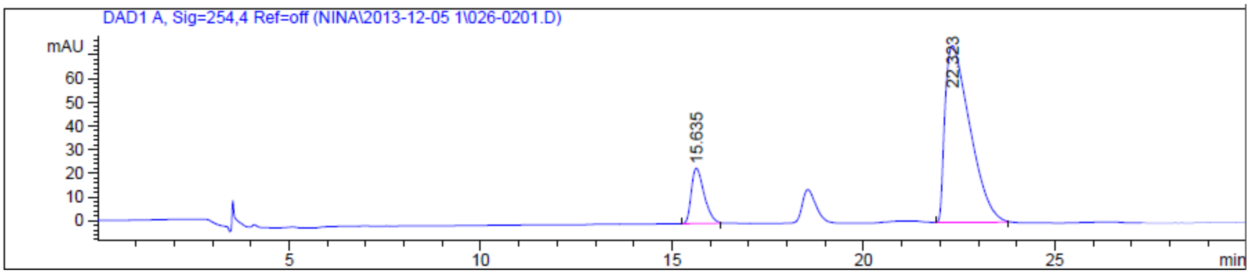

Signal 1: DAD1 A, Sig=254,4 Ref=off

Peak #	RetTime [min]	Type	Width [min]	Area [mAU*s]	Height [mAU]	Area %
1	15.635	BB	0.3600	545.68378	23.40274	13.9467
2	22.323	BB	0.6835	3366.96680	74.75256	86.0533
Totals :				3912.65057	98.15530	

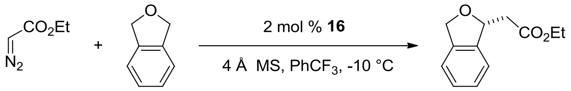

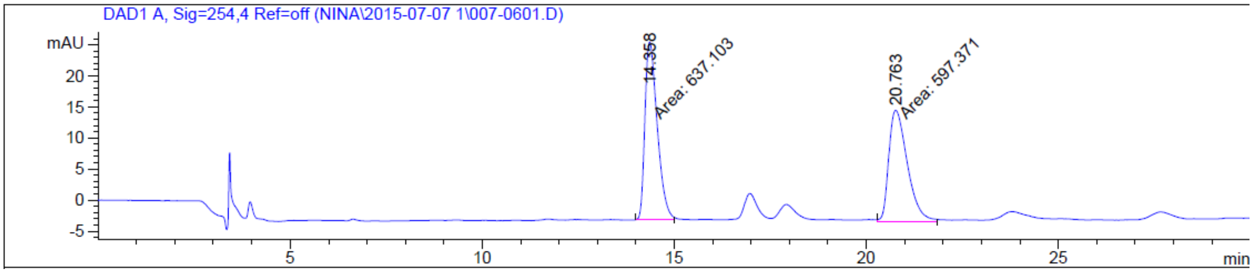

Signal 1: DAD1 A, Sig=254,4 Ref=off

Peak #	RetTime [min]	Type	Width [min]	Area [mAU*s]	Height [mAU]	Area %
1	14.358	MM	0.3721	637.10333	28.53423	51.6093
2	20.763	MM	0.5573	597.37115	17.86541	48.3907
Totals :				1234.47449	46.39964	

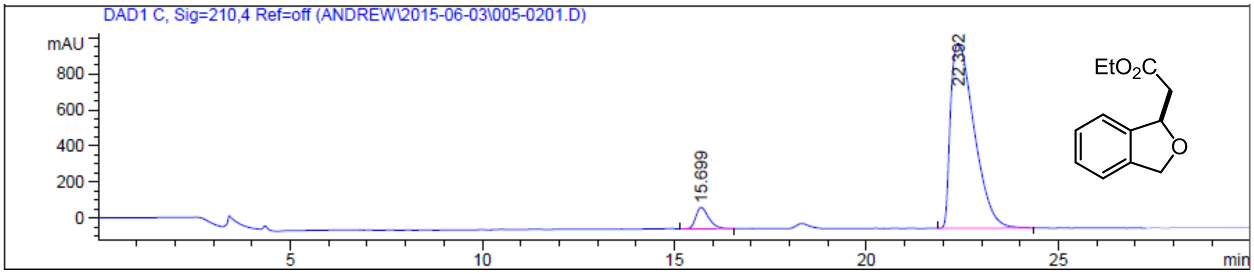

Signal 2: DAD1 C, Sig=210,4 Ref=off

Peak #	RetTime [min]	Type	Width [min]	Area [mAU*s]	Height [mAU]	Area %
1	15.699	VB	0.3596	2820.83472	119.39058	6.0683
2	22.392	VB	0.6665	4.36640e4	1026.11084	93.9317
Totals :				4.64849e4	1145.50142	

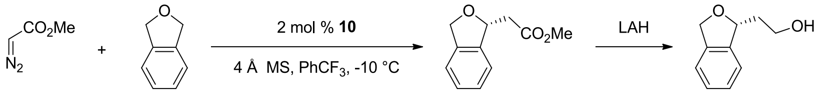

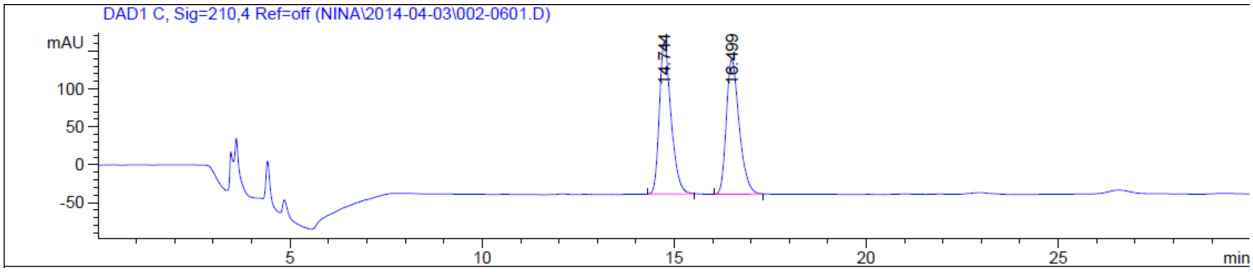

Signal 2: DAD1 C, Sig=210,4 Ref=off

Peak #	RetTime [min]	Type	Width [min]	Area [mAU*s]	Height [mAU]	Area %
1	14.744	BB	0.3209	4235.80615	200.15521	50.6009
2	16.499	BB	0.3535	4135.21143	177.66600	49.3991
Totals :				8371.01758	377.82121	

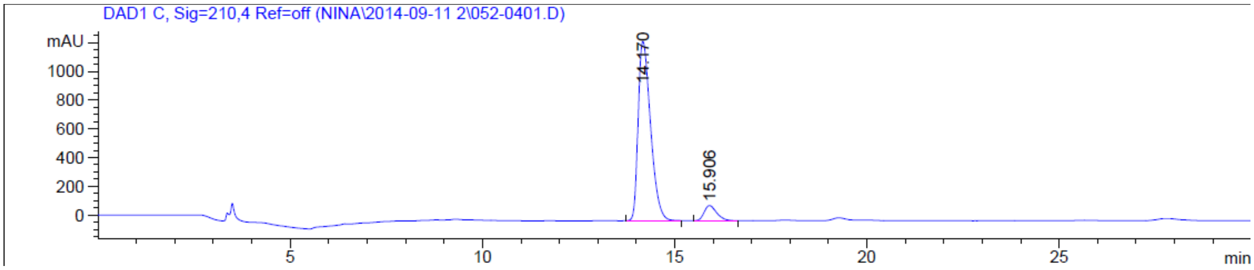

Signal 2: DAD1 C, Sig=210,4 Ref=off

Peak #	RetTime [min]	Type	Width [min]	Area [mAU*s]	Height [mAU]	Area %
1	14.170	BB	0.3471	2.79630e4	1249.38208	92.1776
2	15.906	BB	0.3437	2372.99487	104.97583	7.8224
Totals :				3.03360e4	1354.35791	

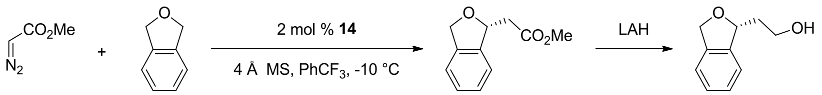

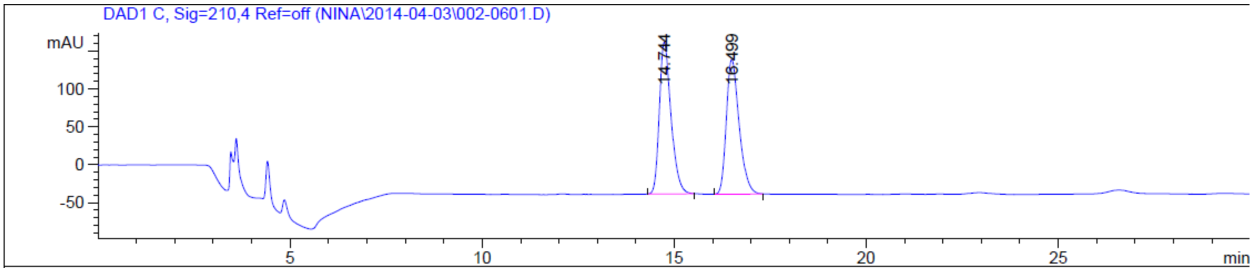

Signal 2: DAD1 C, Sig=210,4 Ref=off

Peak #	RetTime [min]	Type	Width [min]	Area [mAU*s]	Height [mAU]	Area %
1	14.744	BB	0.3209	4235.80615	200.15521	50.6009
2	16.499	BB	0.3535	4135.21143	177.66600	49.3991

Totals : 8371.01758 377.82121

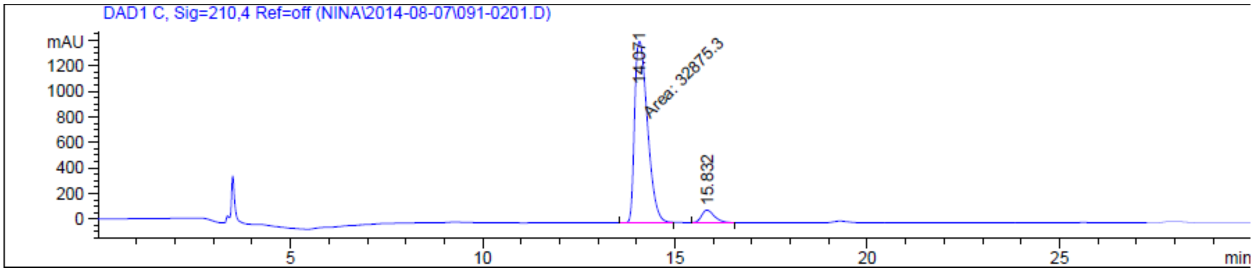

Signal 2: DAD1 C, Sig=210,4 Ref=off

Peak #	RetTime [min]	Type	Width [min]	Area [mAU*s]	Height [mAU]	Area %
1	14.071	MM	0.3855	3.28753e4	1421.50244	93.6980
2	15.832	BB	0.3458	2211.15039	97.77789	6.3020

Totals : 3.50864e4 1519.28033

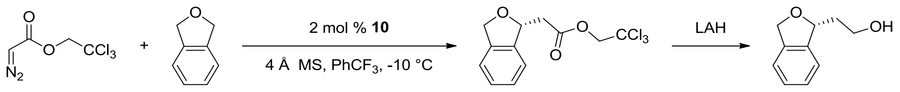

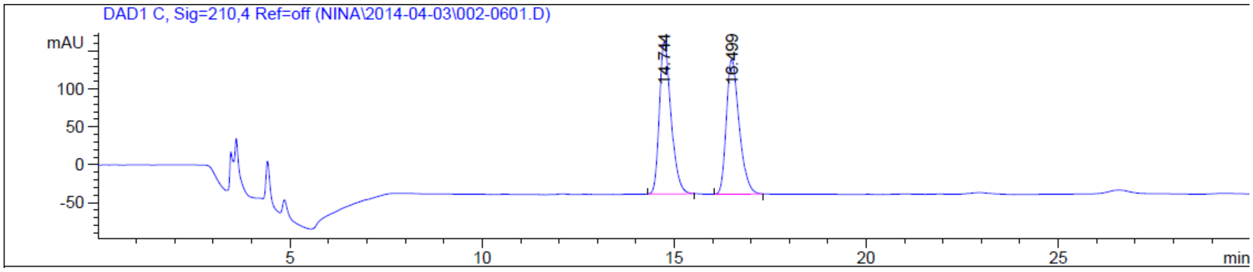

Signal 2: DAD1 C, Sig=210,4 Ref=off

Peak #	RetTime [min]	Type	Width [min]	Area [mAU*s]	Height [mAU]	Area %
1	14.744	BB	0.3209	4235.80615	200.15521	50.6009
2	16.499	BB	0.3535	4135.21143	177.66600	49.3991

Totals : 8371.01758 377.82121

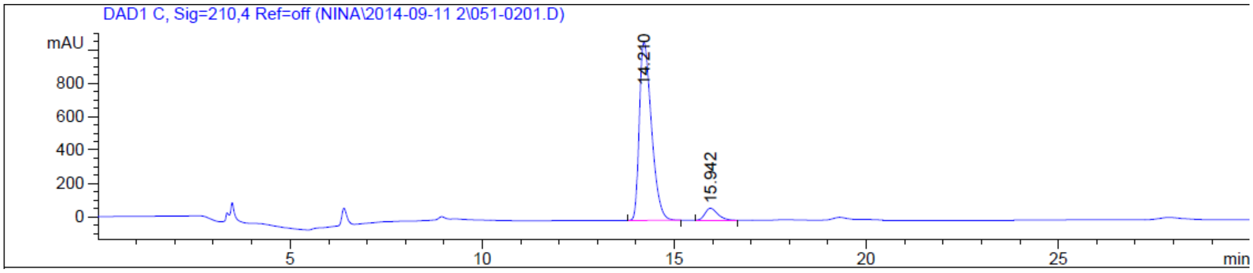

Signal 2: DAD1 C, Sig=210,4 Ref=off

Peak #	RetTime [min]	Type	Width [min]	Area [mAU*s]	Height [mAU]	Area %
1	14.210	BB	0.3410	2.34825e4	1066.14734	93.5235
2	15.942	BB	0.3435	1626.15161	71.98818	6.4765

Totals : 2.51086e4 1138.13552

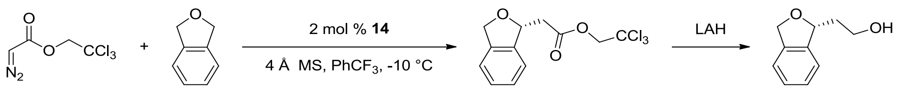

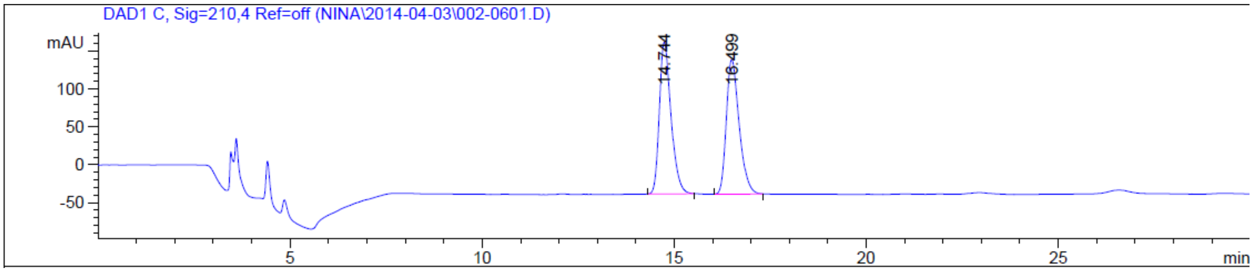

Signal 2: DAD1 C, Sig=210,4 Ref=off

Peak #	RetTime [min]	Type	Width [min]	Area [mAU*s]	Height [mAU]	Area %
1	14.744	BB	0.3209	4235.80615	200.15521	50.6009
2	16.499	BB	0.3535	4135.21143	177.66600	49.3991

Totals : 8371.01758 377.82121

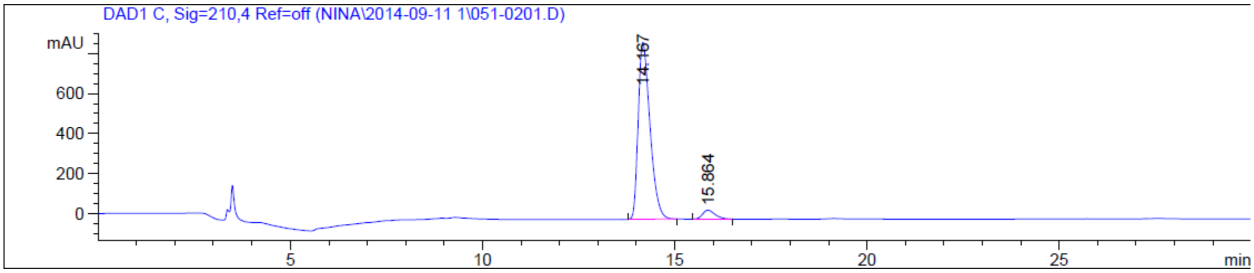

Signal 2: DAD1 C, Sig=210,4 Ref=off

Peak #	RetTime [min]	Type	Width [min]	Area [mAU*s]	Height [mAU]	Area %
1	14.167	BB	0.3265	1.87930e4	882.12140	95.0652
2	15.864	BB	0.3343	975.53076	44.05865	4.9348

Totals : 1.97685e4 926.18005

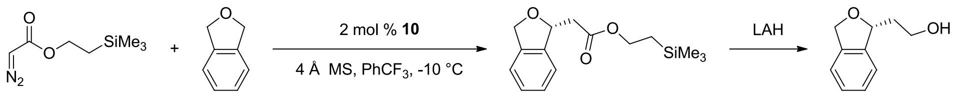

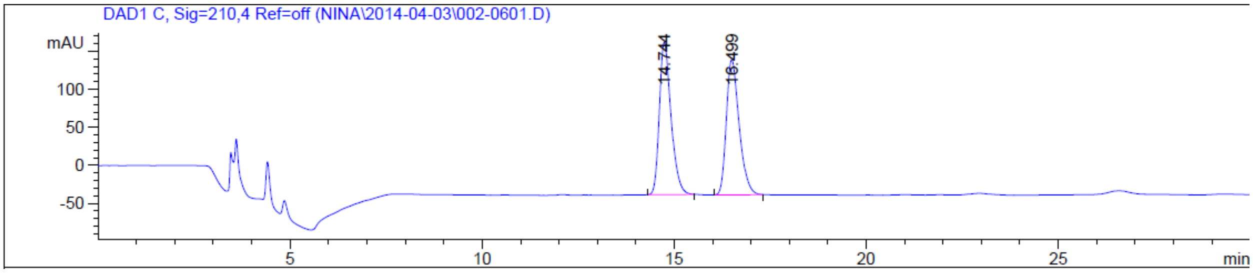

Signal 2: DAD1 C, Sig=210,4 Ref=off

Peak #	RetTime [min]	Type	Width [min]	Area [mAU*s]	Height [mAU]	Area %
1	14.744	BB	0.3209	4235.80615	200.15521	50.6009
2	16.499	BB	0.3535	4135.21143	177.66600	49.3991
Totals :				8371.01758	377.82121	

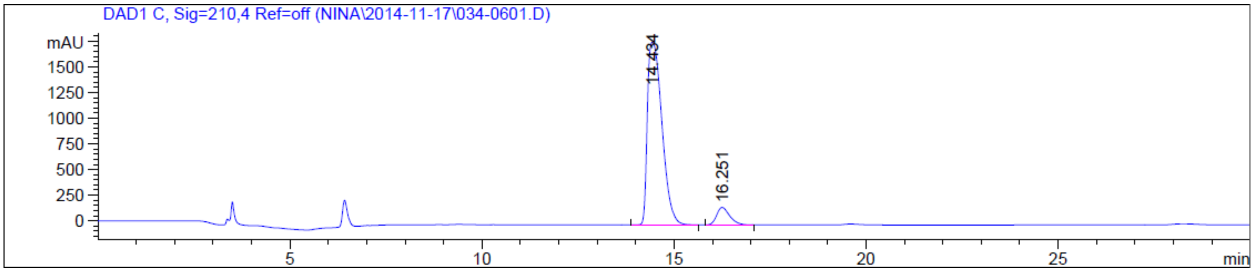

Signal 2: DAD1 C, Sig=210,4 Ref=off

Peak #	RetTime [min]	Type	Width [min]	Area [mAU*s]	Height [mAU]	Area %
1	14.434	BB	0.4205	4.70203e4	1777.95764	92.1341
2	16.251	BB	0.3618	4014.33569	169.81734	7.8659
Totals :				5.10347e4	1947.77498	

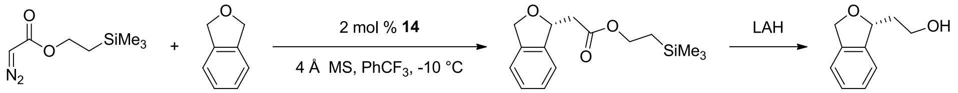

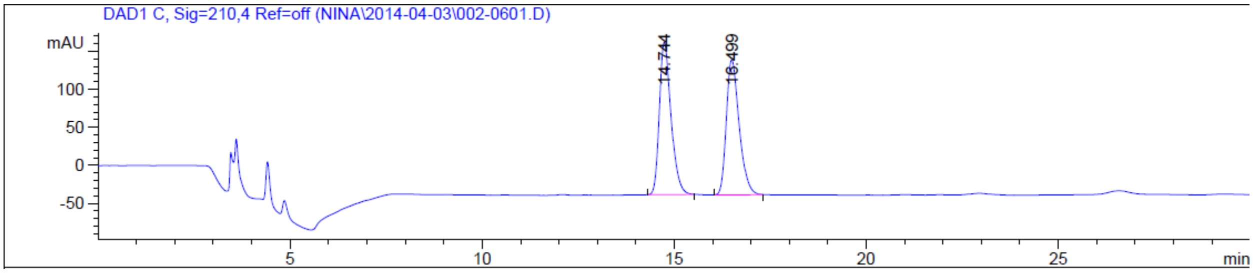

Signal 2: DAD1 C, Sig=210,4 Ref=off

Peak #	RetTime [min]	Type	Width [min]	Area [mAU*s]	Height [mAU]	Area %
1	14.744	BB	0.3209	4235.80615	200.15521	50.6009
2	16.499	BB	0.3535	4135.21143	177.66600	49.3991
Totals :				8371.01758	377.82121	

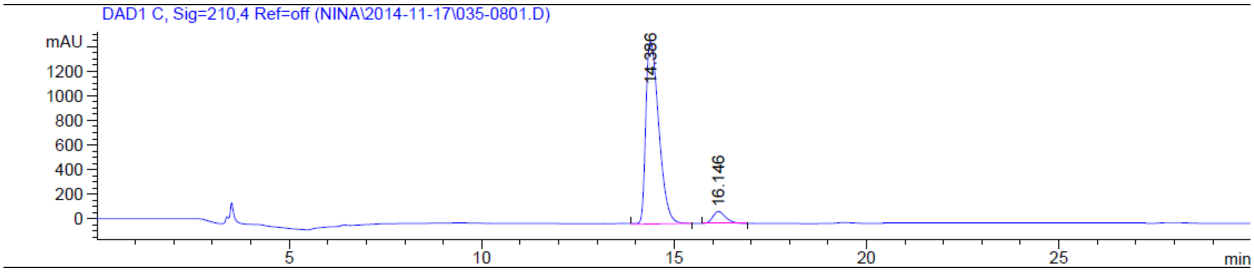

Signal 2: DAD1 C, Sig=210,4 Ref=off

Peak #	RetTime [min]	Type	Width [min]	Area [mAU*s]	Height [mAU]	Area %
1	14.386	BB	0.3706	3.51173e4	1481.32349	94.0331
2	16.146	BB	0.3497	2228.39624	97.13264	5.9669
Totals :				3.73457e4	1578.45612	

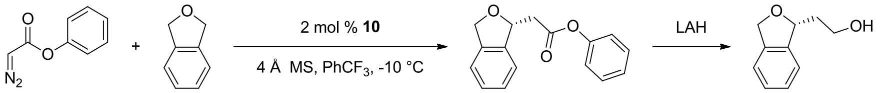

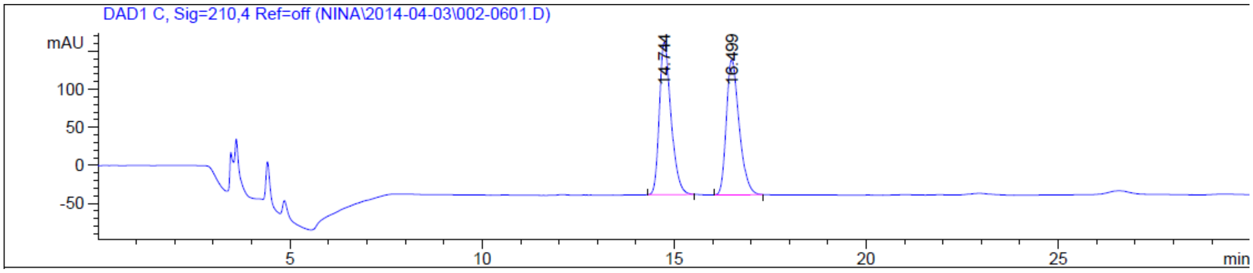

Signal 2: DAD1 C, Sig=210,4 Ref=off

Peak #	RetTime [min]	Type	Width [min]	Area [mAU*s]	Height [mAU]	Area %
1	14.744	BB	0.3209	4235.80615	200.15521	50.6009
2	16.499	BB	0.3535	4135.21143	177.66600	49.3991

Totals : 8371.01758 377.82121

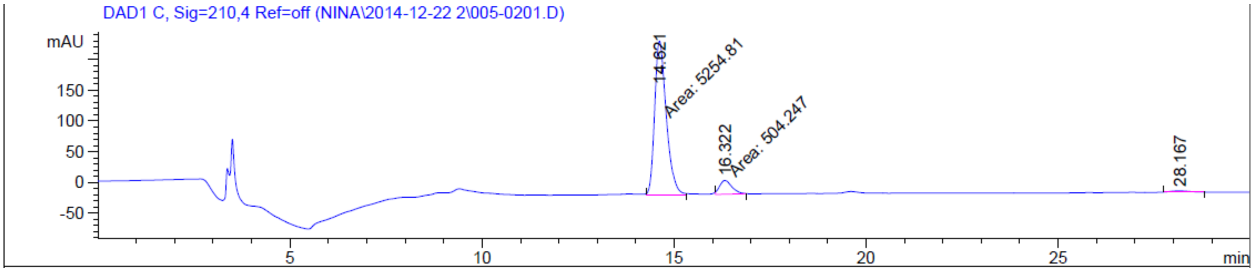

Signal 2: DAD1 C, Sig=210,4 Ref=off

Peak #	RetTime [min]	Type	Width [min]	Area [mAU*s]	Height [mAU]	Area %
1	14.621	MM	0.3505	5254.80566	249.89125	90.2423
2	16.322	MM	0.3724	504.24701	22.56823	8.6596
3	28.167	BB	0.3989	63.94430	1.93806	1.0981

Totals : 5822.99697 274.39754

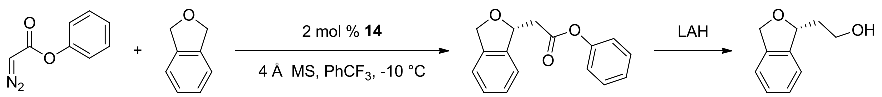

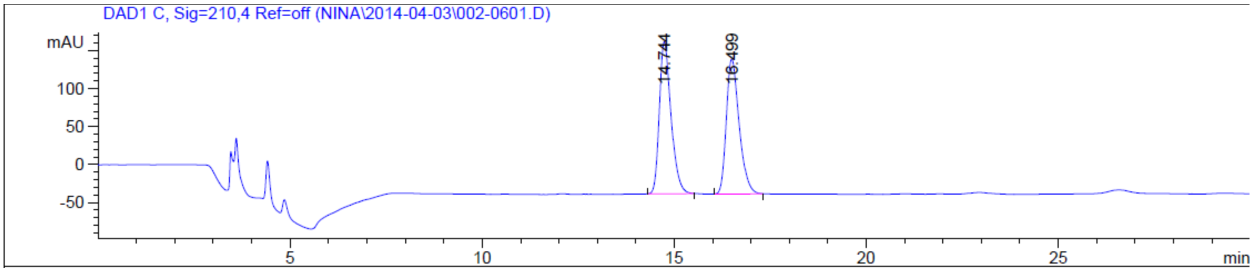

Signal 2: DAD1 C, Sig=210,4 Ref=off

Peak #	RetTime [min]	Type	Width [min]	Area [mAU*s]	Height [mAU]	Area %
1	14.744	BB	0.3209	4235.80615	200.15521	50.6009
2	16.499	BB	0.3535	4135.21143	177.66600	49.3991

Totals : 8371.01758 377.82121

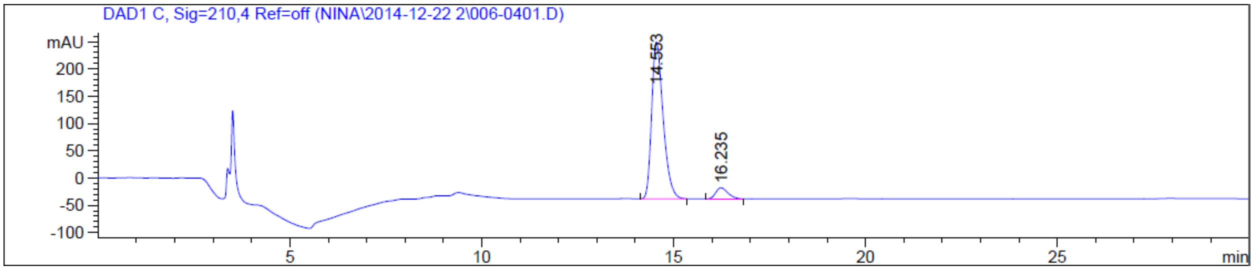

Signal 2: DAD1 C, Sig=210,4 Ref=off

Peak #	RetTime [min]	Type	Width [min]	Area [mAU*s]	Height [mAU]	Area %
1	14.553	BB	0.3181	6015.99414	287.48108	92.9144
2	16.235	BB	0.3452	458.77490	20.48825	7.0856

Totals : 6474.76904 307.96932

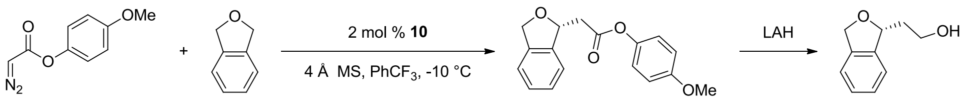

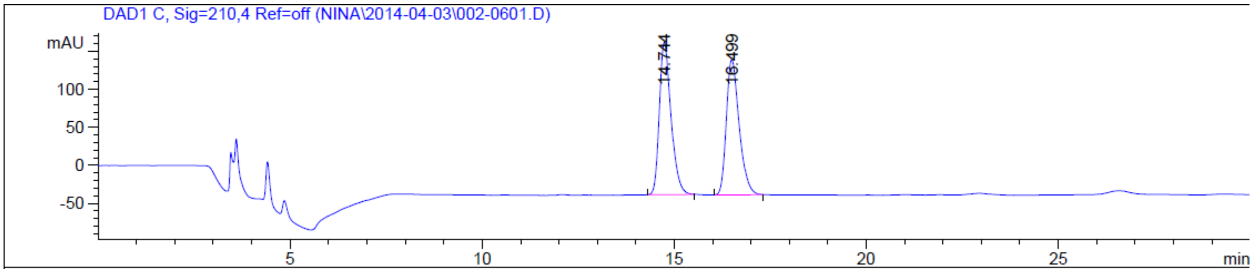

Signal 2: DAD1 C, Sig=210,4 Ref=off

Peak #	RetTime [min]	Type	Width [min]	Area [mAU*s]	Height [mAU]	Area %
1	14.744	BB	0.3209	4235.80615	200.15521	50.6009
2	16.499	BB	0.3535	4135.21143	177.66600	49.3991

Totals : 8371.01758 377.82121

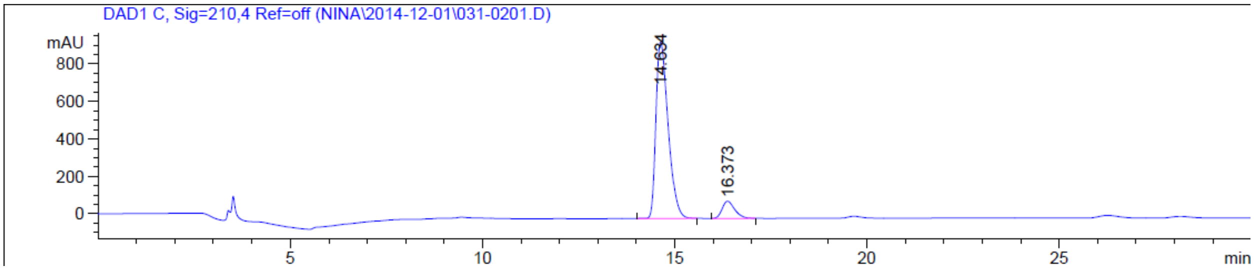

Signal 2: DAD1 C, Sig=210,4 Ref=off

Peak #	RetTime [min]	Type	Width [min]	Area [mAU*s]	Height [mAU]	Area %
1	14.634	BB	0.3427	2.08809e4	941.80194	90.7960
2	16.373	BB	0.3516	2116.70166	92.26576	9.2040

Totals : 2.29976e4 1034.06770

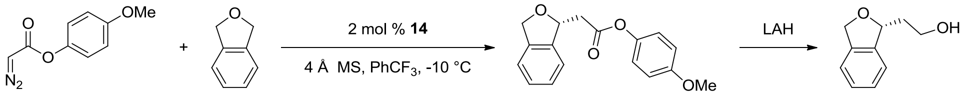

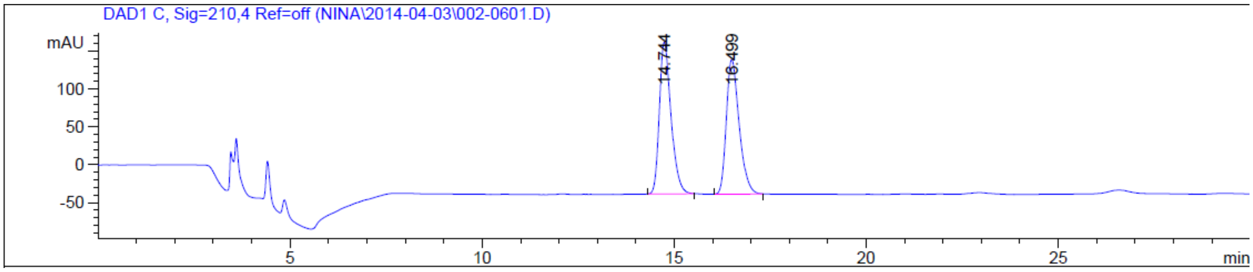

Signal 2: DAD1 C, Sig=210,4 Ref=off

Peak #	RetTime [min]	Type	Width [min]	Area [mAU*s]	Height [mAU]	Area %
1	14.744	BB	0.3209	4235.80615	200.15521	50.6009
2	16.499	BB	0.3535	4135.21143	177.66600	49.3991

Totals : 8371.01758 377.82121

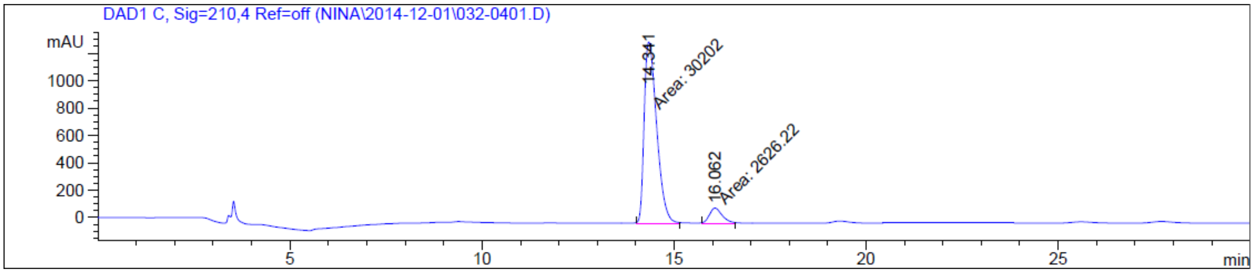

Signal 2: DAD1 C, Sig=210,4 Ref=off

Peak #	RetTime [min]	Type	Width [min]	Area [mAU*s]	Height [mAU]	Area %
1	14.341	MM	0.3796	3.02020e4	1325.91382	92.0001
2	16.062	MM	0.3899	2626.21826	112.25780	7.9999

Totals : 3.28282e4 1438.17162

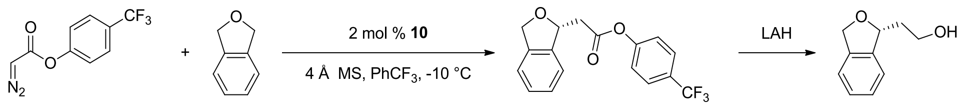

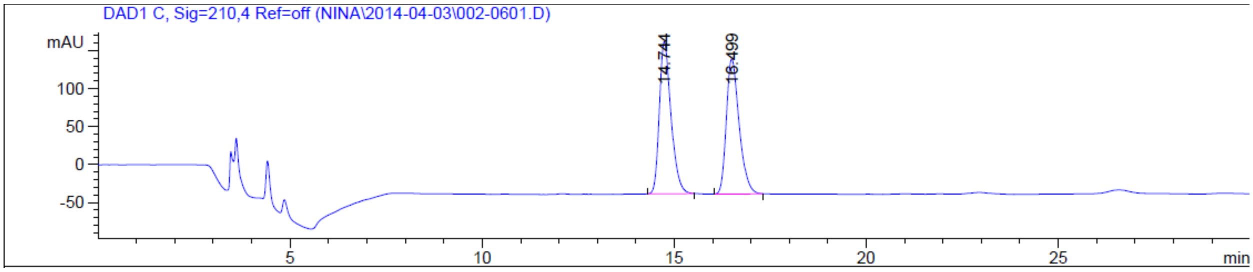

Signal 2: DAD1 C, Sig=210,4 Ref=off

Peak #	RetTime [min]	Type	Width [min]	Area [mAU*s]	Height [mAU]	Area %
1	14.744	BB	0.3209	4235.80615	200.15521	50.6009
2	16.499	BB	0.3535	4135.21143	177.66600	49.3991

Totals : 8371.01758 377.82121

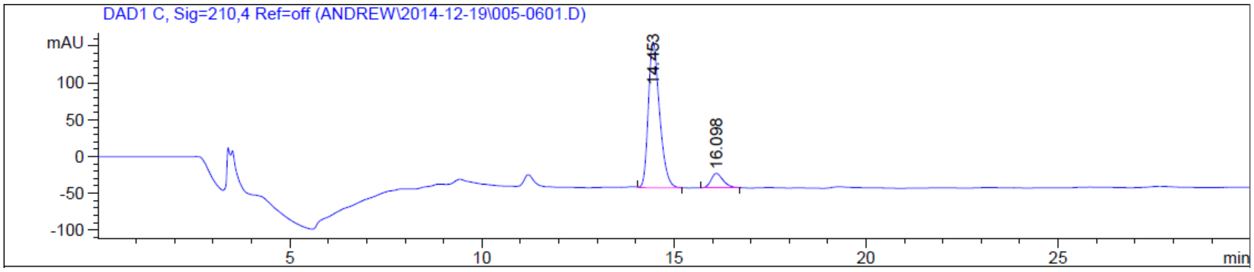

Signal 2: DAD1 C, Sig=210,4 Ref=off

Peak #	RetTime [min]	Type	Width [min]	Area [mAU*s]	Height [mAU]	Area %
1	14.453	VB	0.3110	4052.43457	197.78882	90.1528
2	16.098	BB	0.3404	442.64069	19.82982	9.8472

Totals : 4495.07526 217.61864

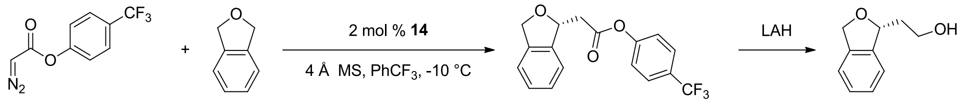

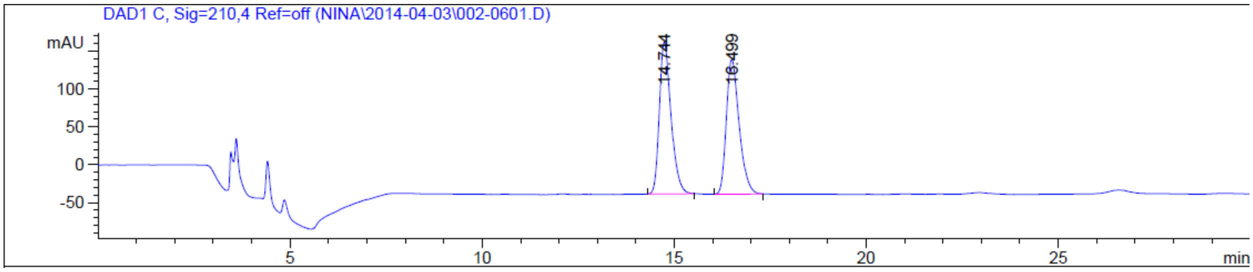

Signal 2: DAD1 C, Sig=210,4 Ref=off

Peak #	RetTime [min]	Type	Width [min]	Area [mAU*s]	Height [mAU]	Area %
1	14.744	BB	0.3209	4235.80615	200.15521	50.6009
2	16.499	BB	0.3535	4135.21143	177.66600	49.3991

Totals : 8371.01758 377.82121

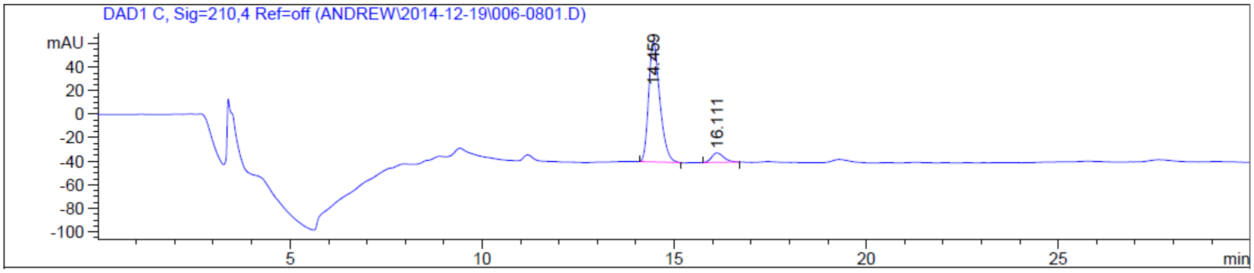

Signal 2: DAD1 C, Sig=210,4 Ref=off

Peak #	RetTime [min]	Type	Width [min]	Area [mAU*s]	Height [mAU]	Area %
1	14.459	BB	0.3112	2089.42212	101.92041	92.0745
2	16.111	BB	0.3353	179.85083	8.09250	7.9255

Totals : 2269.27295 110.01291

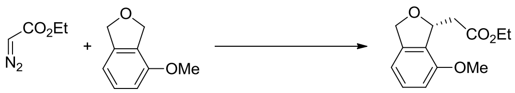

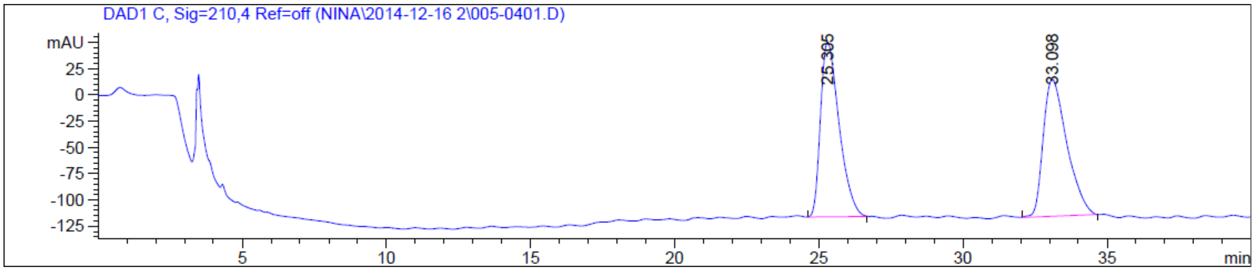

Signal 2: DAD1 C, Sig=210,4 Ref=off

Peak #	RetTime [min]	Type	Width [min]	Area [mAU*s]	Height [mAU]	Area %
1	25.305	BB	0.6898	7471.09521	166.39967	50.0894
2	33.098	BB	0.8667	7444.43311	130.26862	49.9106

Totals : 1.49155e4 296.66829

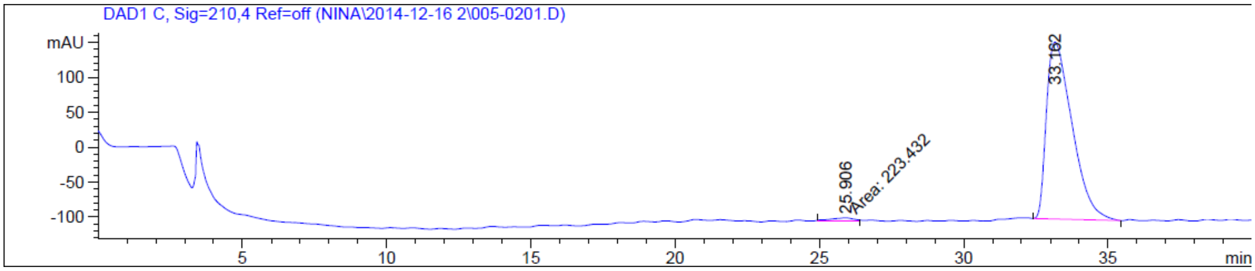

Signal 2: DAD1 C, Sig=210,4 Ref=off

Peak #	RetTime [min]	Type	Width [min]	Area [mAU*s]	Height [mAU]	Area %
1	25.906	MM	0.8960	223.43199	4.15602	1.4087
2	33.162	BB	0.9260	1.56370e4	253.36447	98.5913

Totals : 1.58605e4 257.52049

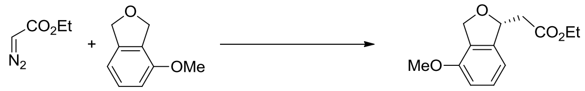

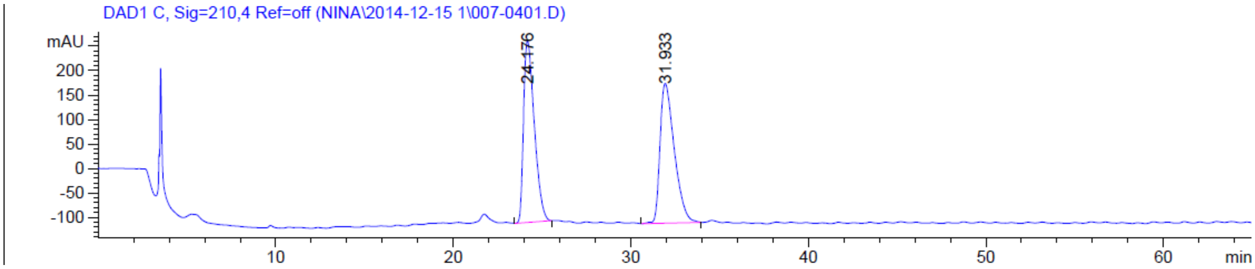

Signal 2: DAD1 C, Sig=210,4 Ref=off

Peak #	RetTime [min]	Type	Width [min]	Area [mAU*s]	Height [mAU]	Area %
1	24.176	VB	0.6499	1.56448e4	371.07114	49.4712
2	31.933	VB	0.8488	1.59792e4	285.59573	50.5288

Totals : 3.16240e4 656.66687

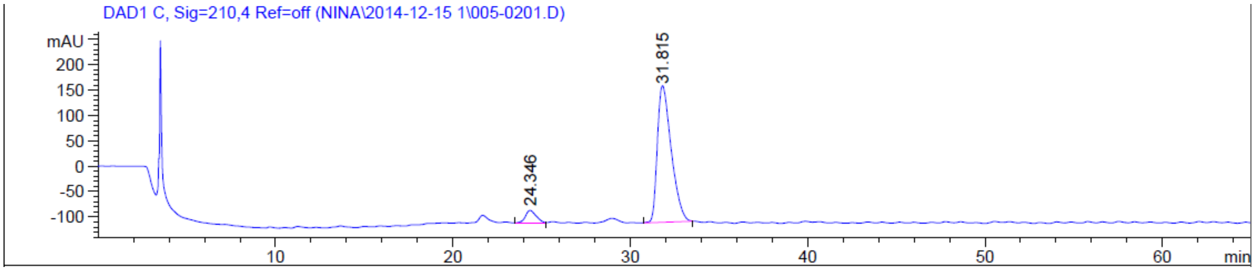

Signal 2: DAD1 C, Sig=210,4 Ref=off

Peak #	RetTime [min]	Type	Width [min]	Area [mAU*s]	Height [mAU]	Area %
1	24.346	VV	0.6209	1104.30969	25.31747	7.0068
2	31.815	BB	0.8269	1.46563e4	269.32123	92.9932

Totals : 1.57606e4 294.63870

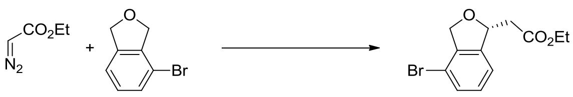

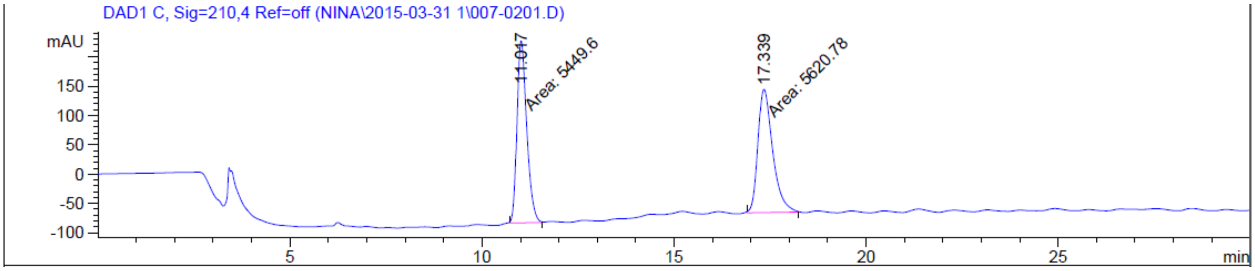

Signal 2: DAD1 C, Sig=210,4 Ref=off

Peak #	RetTime [min]	Type	Width [min]	Area [mAU*s]	Height [mAU]	Area %
1	11.017	MM	0.2929	5449.60352	310.11356	49.2269
2	17.339	MM	0.4460	5620.78320	210.05756	50.7731

Totals : 1.10704e4 520.17111

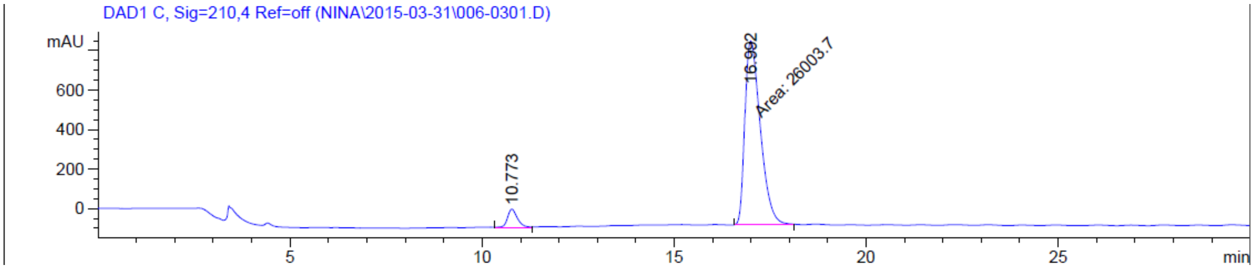

Signal 2: DAD1 C, Sig=210,4 Ref=off

Peak #	RetTime [min]	Type	Width [min]	Area [mAU*s]	Height [mAU]	Area %
1	10.773	BB	0.2783	1742.37476	93.96941	6.2797
2	16.992	MM	0.4654	2.60037e4	931.26843	93.7203

Totals : 2.77460e4 1025.23785

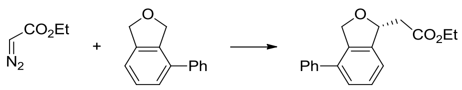

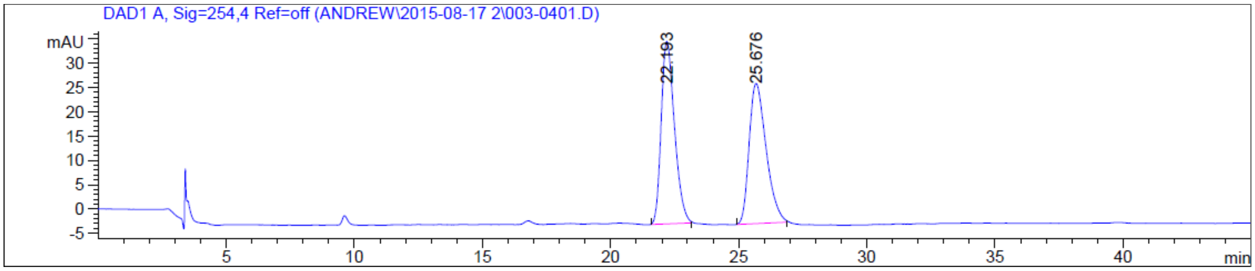

Signal 1: DAD1 A, Sig=254,4 Ref=off

Peak #	RetTime [min]	Type	Width [min]	Area [mAU*s]	Height [mAU]	Area %
1	22.193	BB	0.5485	1352.74438	37.52847	50.4551
2	25.676	BB	0.6962	1328.34192	28.79670	49.5449
Totals :				2681.08630	66.32518	

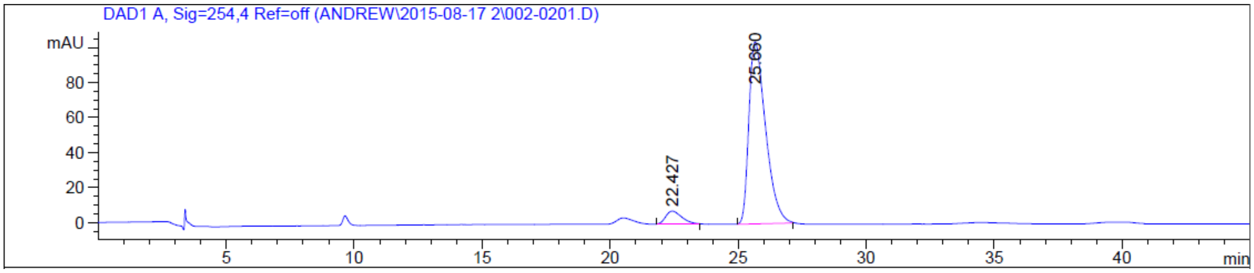

Signal 1: DAD1 A, Sig=254,4 Ref=off

Peak #	RetTime [min]	Type	Width [min]	Area [mAU*s]	Height [mAU]	Area %
1	22.427	BB	0.6231	314.62387	7.32547	6.2630
2	25.660	BB	0.6894	4708.87305	103.77440	93.7370
Totals :				5023.49692	111.09986	

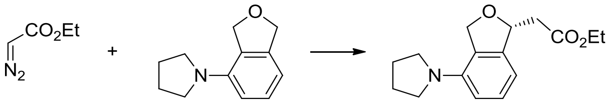

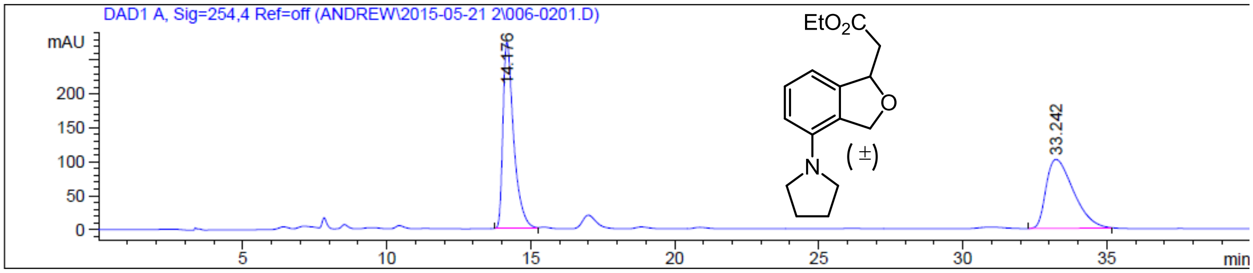

Signal 1: DAD1 A, Sig=254,4 Ref=off

Peak #	RetTime [min]	Type	Width [min]	Area [mAU*s]	Height [mAU]	Area %
1	14.176	BB	0.3916	7146.29053	274.65466	51.6179
2	33.242	BB	1.0062	6698.31885	101.45926	48.3821

Totals : 1.38446e4 376.11392

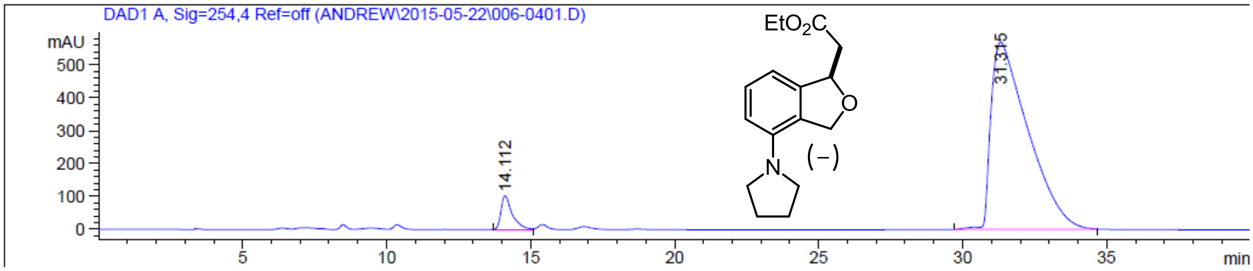

Signal 1: DAD1 A, Sig=254,4 Ref=off

Peak #	RetTime [min]	Type	Width [min]	Area [mAU*s]	Height [mAU]	Area %
1	14.112	BB	0.3956	2756.41089	102.55908	5.1337
2	31.315	BB	1.2708	5.09358e4	569.86713	94.8663

Totals : 5.36923e4 672.42621

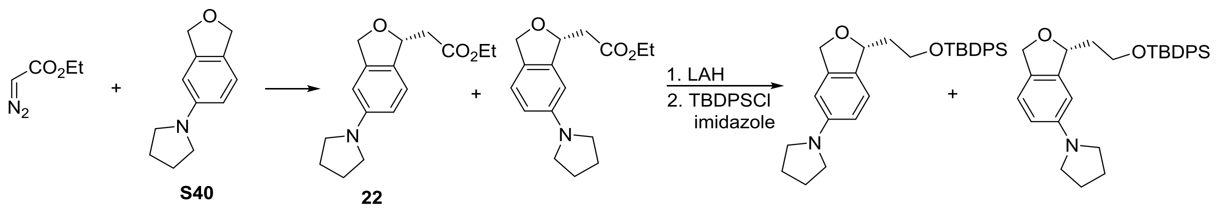

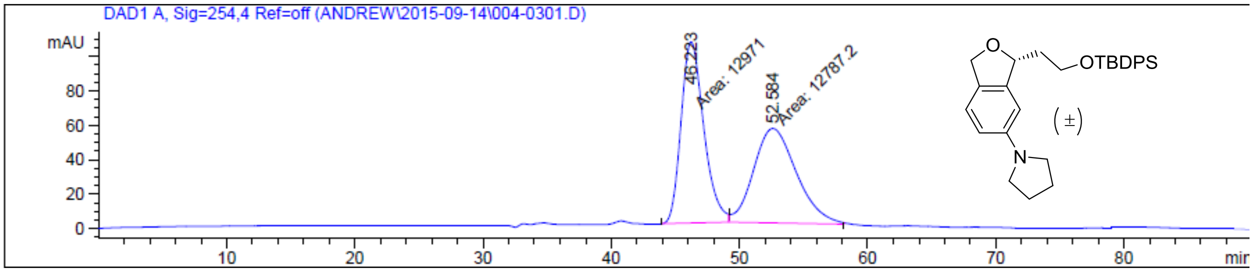

Signal 1: DAD1 A, Sig=254,4 Ref=off

Peak #	RetTime [min]	Type	Width [min]	Area [mAU*s]	Height [mAU]	Area %
1	46.223	MM	2.0558	1.29710e4	105.15704	50.3569
2	52.584	MM	3.8842	1.27872e4	54.86807	49.6431

Totals : 2.57582e4 160.02512

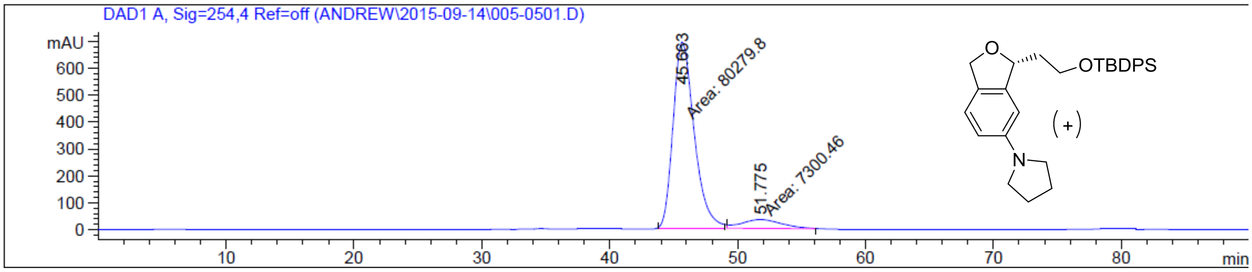

Signal 1: DAD1 A, Sig=254,4 Ref=off

Peak #	RetTime [min]	Type	Width [min]	Area [mAU*s]	Height [mAU]	Area %
1	45.663	MM	1.9242	8.02798e4	695.35242	91.6643
2	51.775	MM	3.6748	7300.46338	33.11041	8.3357

Totals : 8.75803e4 728.46283

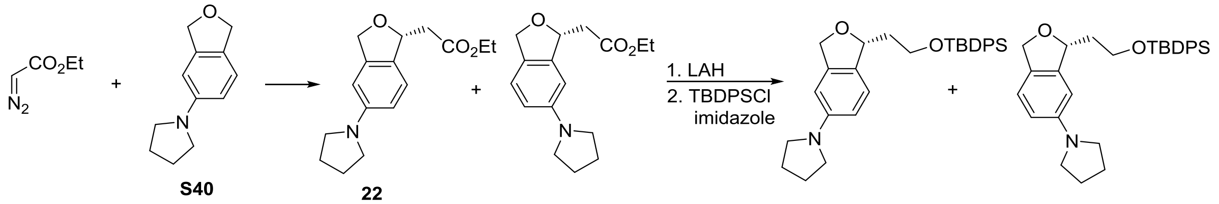

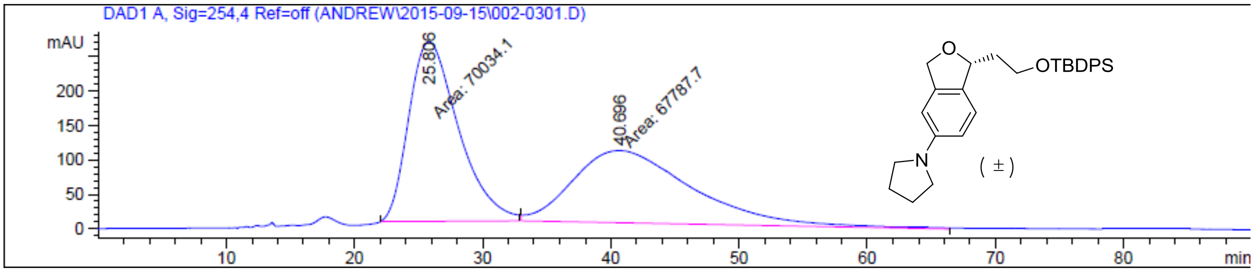

Signal 1: DAD1 A, Sig=254,4 Ref=off

Peak #	RetTime [min]	Type	Width [min]	Area [mAU*s]	Height [mAU]	Area %
1	25.806	MM	4.4932	7.00341e4	259.77750	50.8150
2	40.696	MM	10.8118	6.77877e4	104.49661	49.1850

Totals : 1.37822e5 364.27411

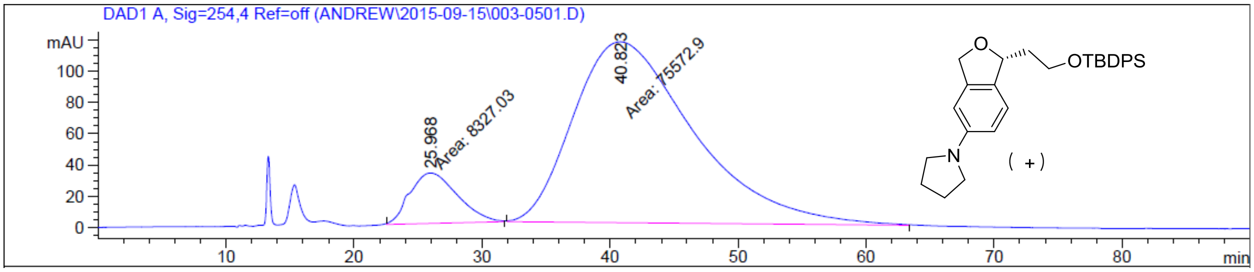

Signal 1: DAD1 A, Sig=254,4 Ref=off

Peak #	RetTime [min]	Type	Width [min]	Area [mAU*s]	Height [mAU]	Area %
1	25.968	MM	4.3103	8327.03418	32.19856	9.9250
2	40.823	MM	10.9090	7.55729e4	115.45967	90.0750

Totals : 8.39000e4 147.65823

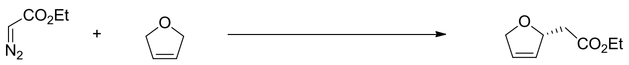

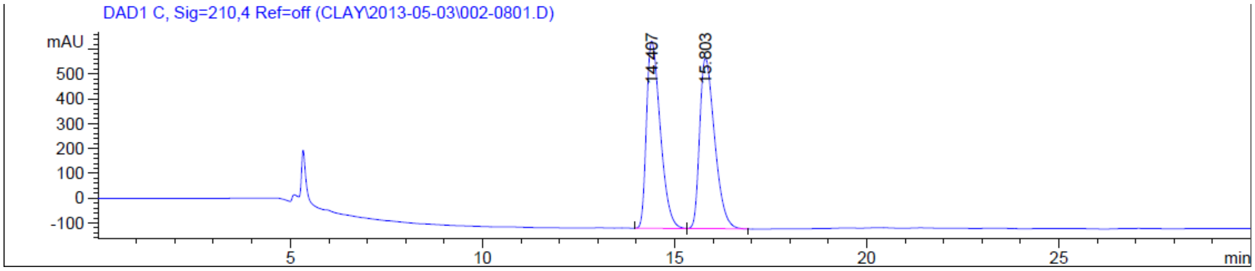

Signal 2: DAD1 C, Sig=210,4 Ref=off

Peak #	RetTime [min]	Type	Width [min]	Area [mAU*s]	Height [mAU]	Area %
1	14.407	BV	0.3824	1.86462e4	754.40552	49.8357
2	15.803	VB	0.4225	1.87691e4	687.53741	50.1643

Totals : 3.74153e4 1441.94293

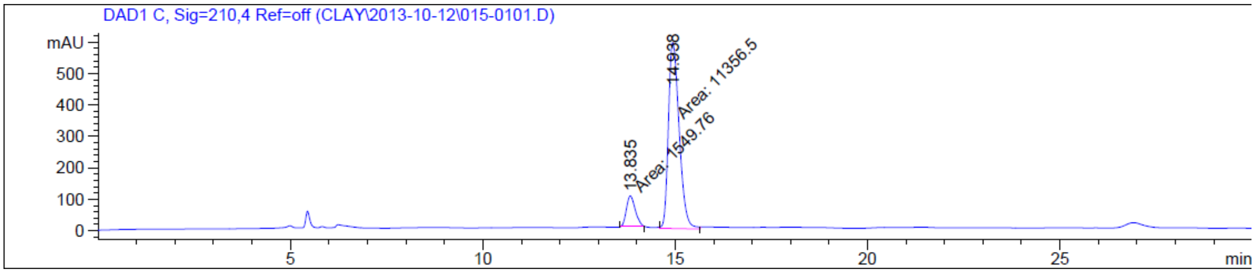

Signal 2: DAD1 C, Sig=210,4 Ref=off

Peak #	RetTime [min]	Type	Width [min]	Area [mAU*s]	Height [mAU]	Area %
1	13.835	MM	0.2662	1549.76001	97.01272	12.0078
2	14.938	MM	0.3203	1.13565e4	590.91559	87.9922

Totals : 1.29062e4 687.92831

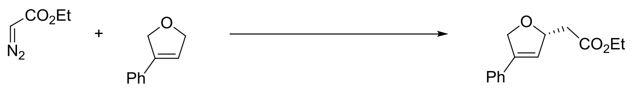

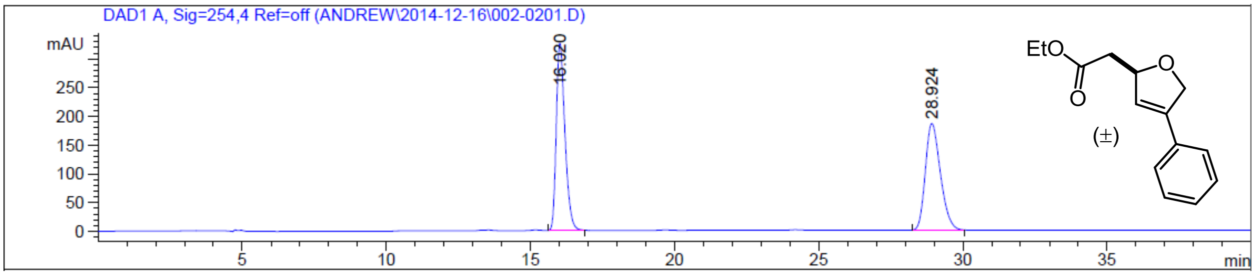

Signal 1: DAD1 A, Sig=254,4 Ref=off

Peak #	RetTime [min]	Type	Width [min]	Area [mAU*s]	Height [mAU]	Area %
1	16.020	BB	0.3194	6804.85840	326.16312	50.1674
2	28.924	BB	0.5601	6759.43701	185.95329	49.8326

Totals : 1.35643e4 512.11641

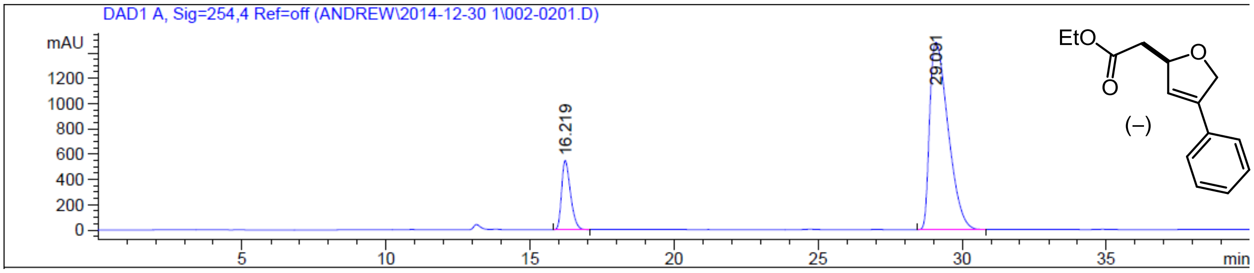

Signal 1: DAD1 A, Sig=254,4 Ref=off

Peak #	RetTime [min]	Type	Width [min]	Area [mAU*s]	Height [mAU]	Area %
1	16.219	BB	0.3288	1.17967e4	548.74457	15.1892
2	29.091	BB	0.6924	6.58683e4	1482.40454	84.8108

Totals : 7.76650e4 2031.14911

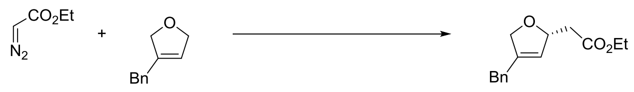

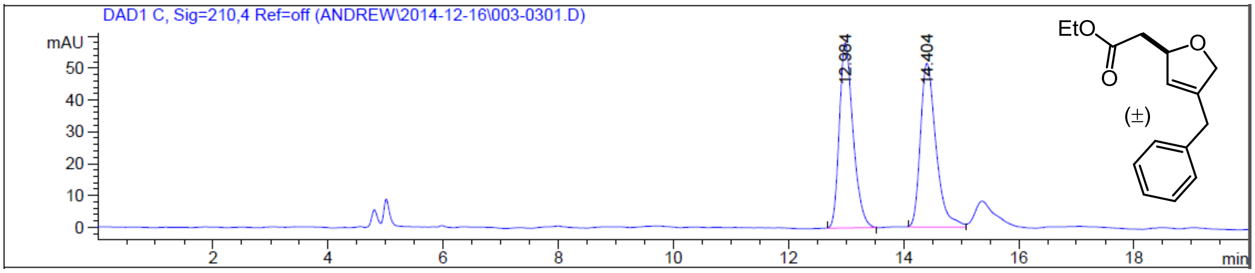

Signal 2: DAD1 C, Sig=210,4 Ref=off

Peak #	RetTime [min]	Type	Width [min]	Area [mAU*s]	Height [mAU]	Area %
1	12.984	BB	0.2515	965.21252	58.19901	49.8551
2	14.404	BB	0.2840	970.82379	51.47895	50.1449
Totals :				1936.03632	109.67796	

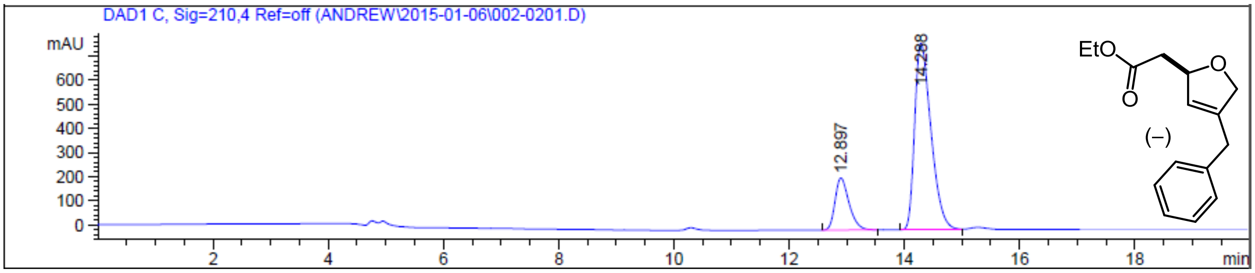

Signal 2: DAD1 C, Sig=210,4 Ref=off

Peak #	RetTime [min]	Type	Width [min]	Area [mAU*s]	Height [mAU]	Area %
1	12.897	BB	0.2543	3629.76367	215.68619	19.1662
2	14.288	BV	0.3048	1.53086e4	773.81763	80.8338
Totals :				1.89383e4	989.50381	

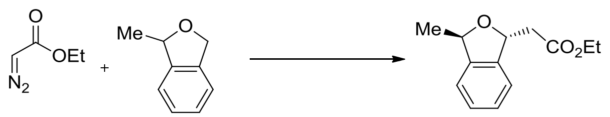

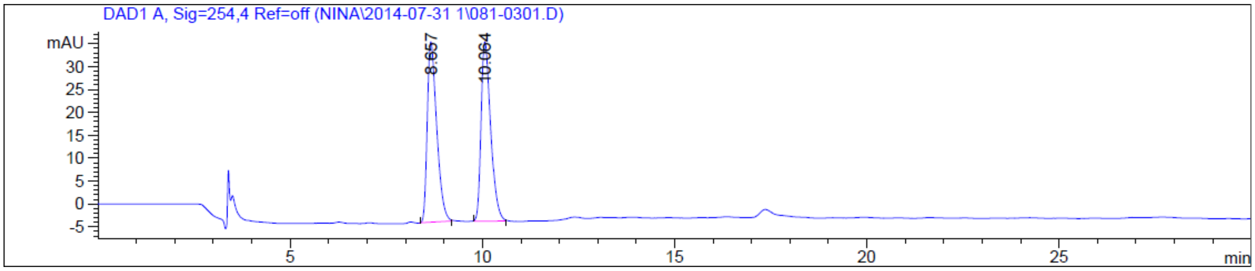

Signal 1: DAD1 A, Sig=254,4 Ref=off

Peak #	RetTime [min]	Type	Width [min]	Area [mAU*s]	Height [mAU]	Area %
1	8.657	BB	0.2588	677.86847	39.39429	49.9453
2	10.064	BB	0.2643	679.35242	39.17409	50.0547

Totals : 1357.22089 78.56838

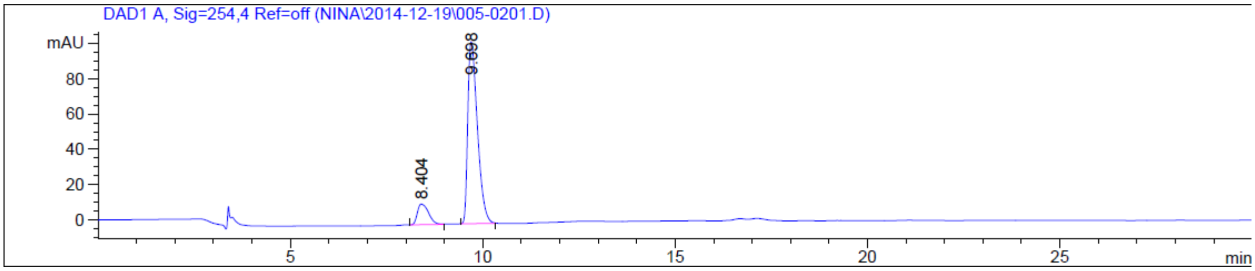

Signal 1: DAD1 A, Sig=254,4 Ref=off

Peak #	RetTime [min]	Type	Width [min]	Area [mAU*s]	Height [mAU]	Area %
1	8.404	BB	0.3193	231.25298	11.66624	11.3630
2	9.698	BB	0.2706	1803.88025	102.84212	88.6370

Totals : 2035.13322 114.50836

IX. NMR Spectra

5-(*tert*-butyl)-N1,N3-bis((*S*)-1-hydroxy-3-methylbutan-2-yl)isophthalamide (S1).

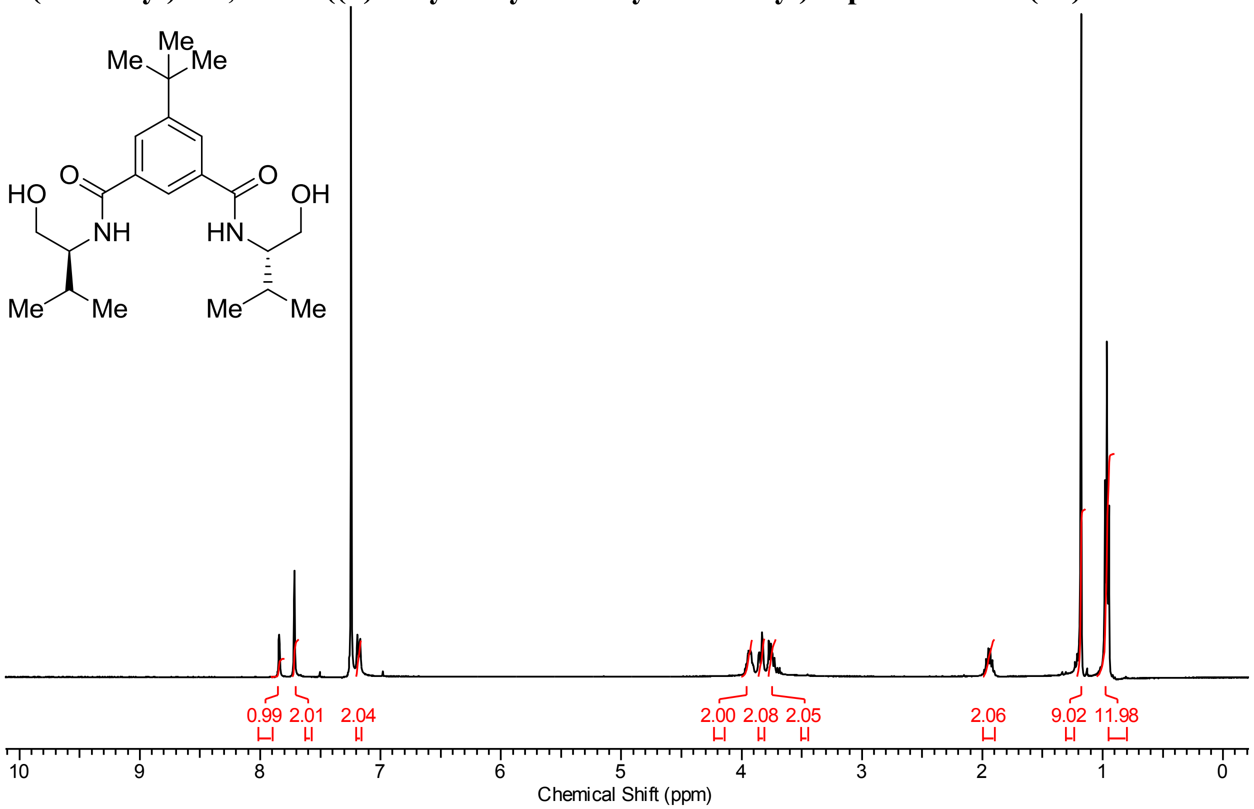

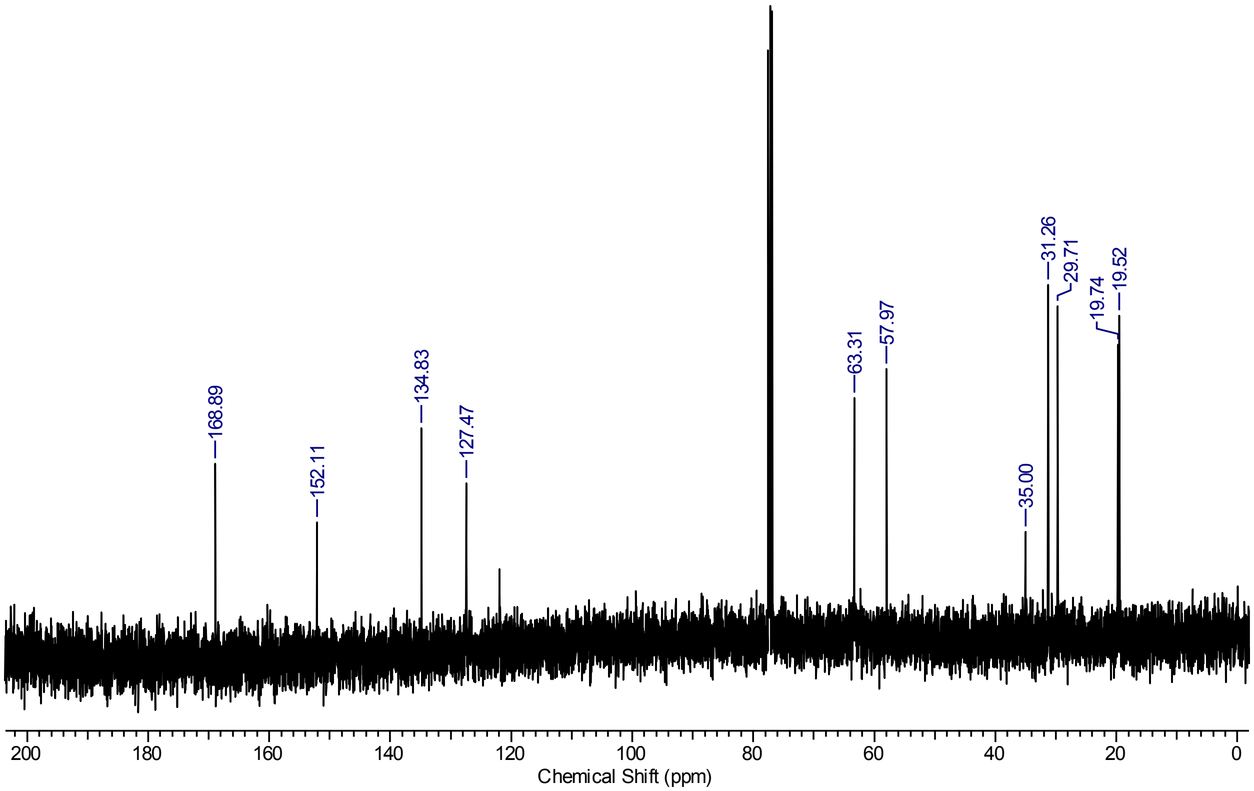

5-(*tert*-butyl)-N1,N3-bis((*S*)-1-hydroxy-4-methylpentan-2-yl)isophthalamide (S2).

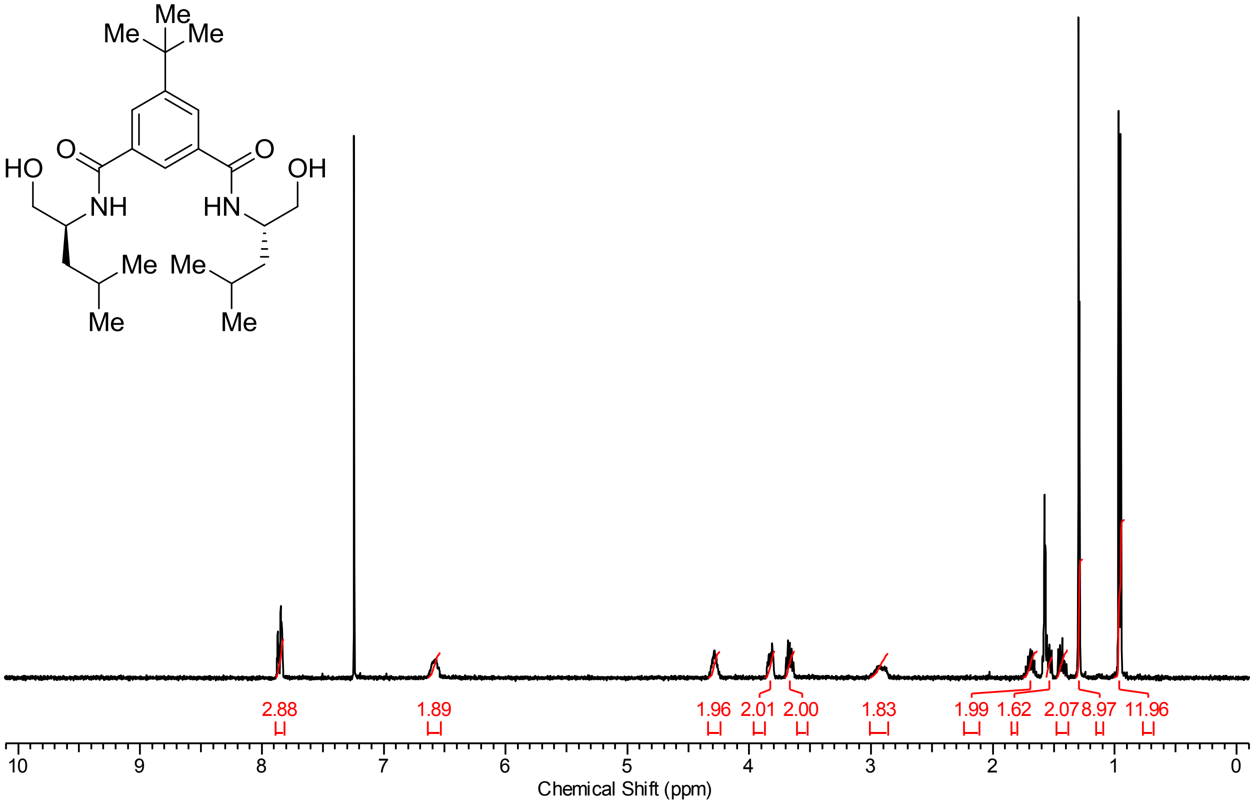

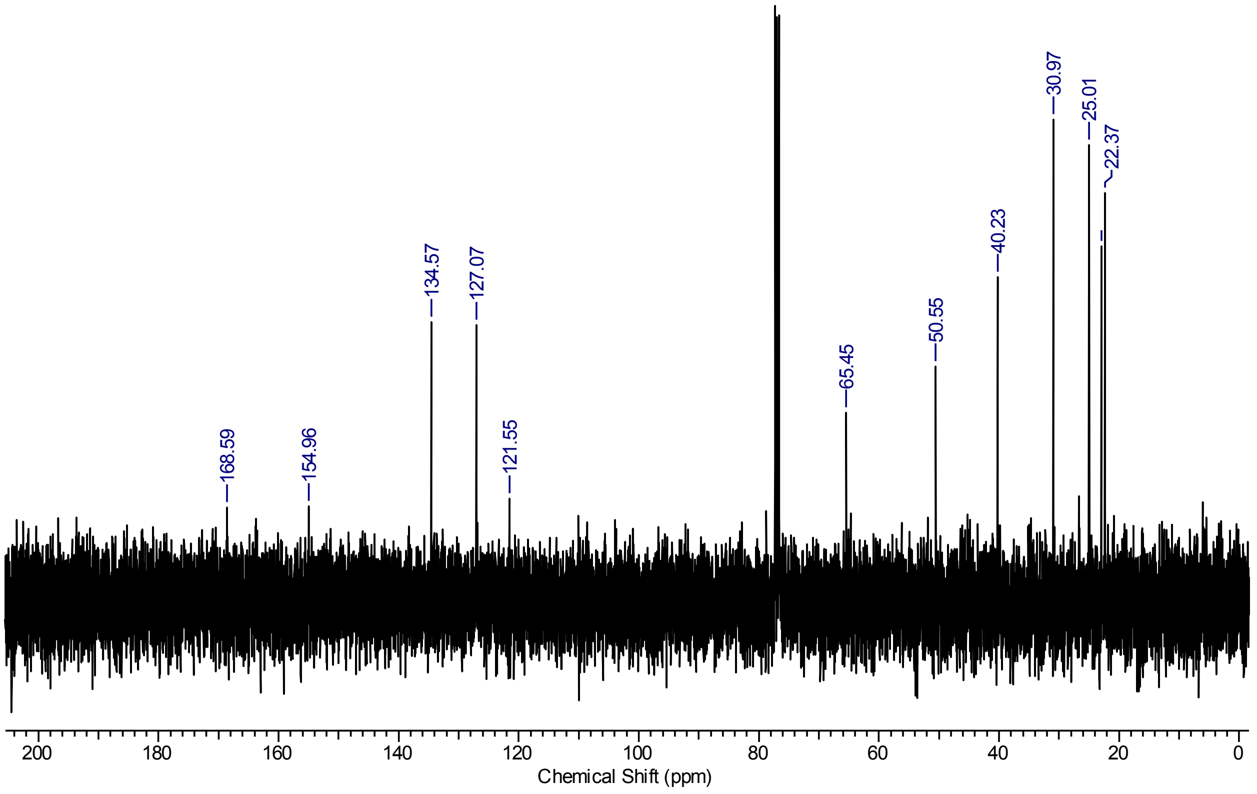

5-(*tert*-butyl)-*N*1,*N*3-bis((*R*)-1-hydroxyhexan-2-yl)isophthalamide (S3).

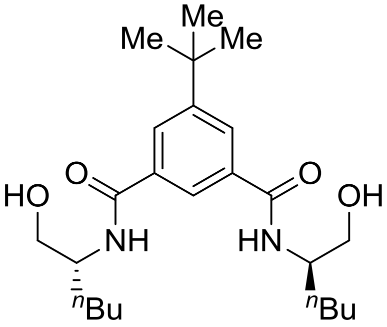

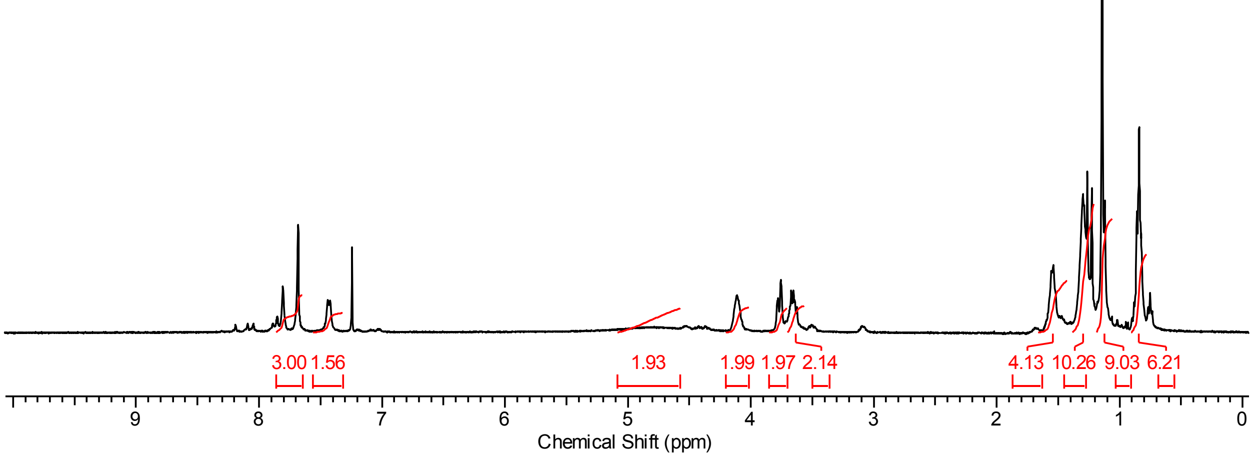

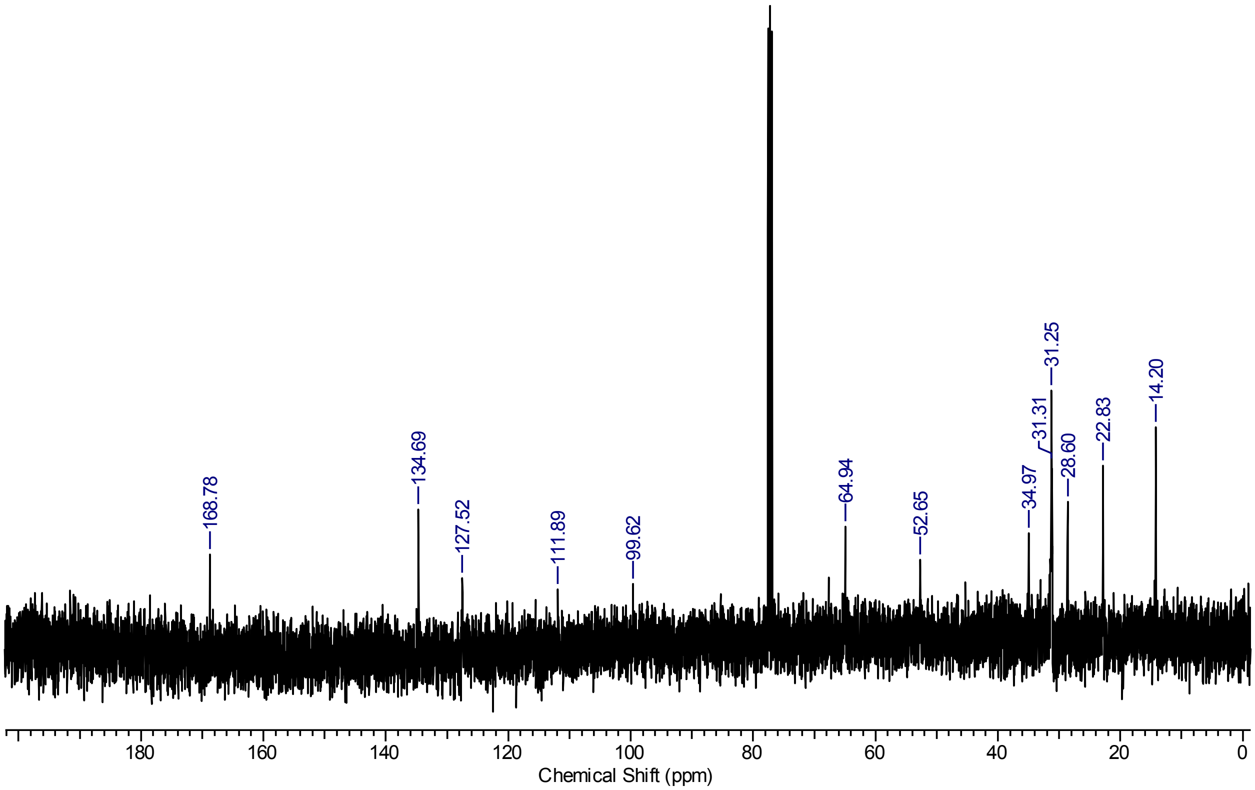

5-(*tert*-butyl)-N1,N3-bis((2*S*,3*S*)-1-hydroxy-3-methylpentan-2-yl)isophthalamide (S4).

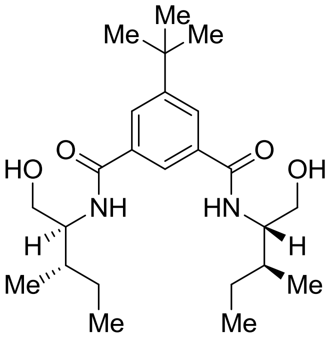

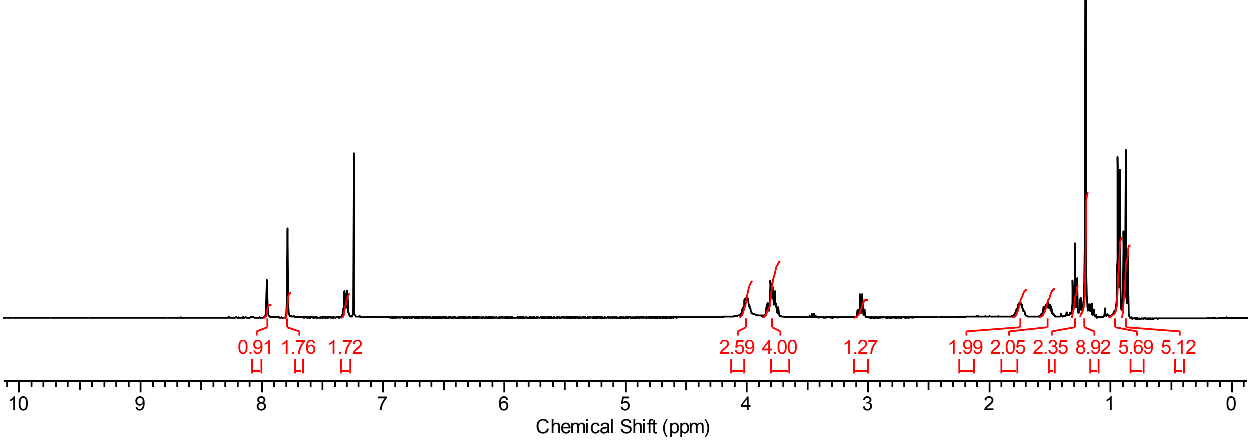

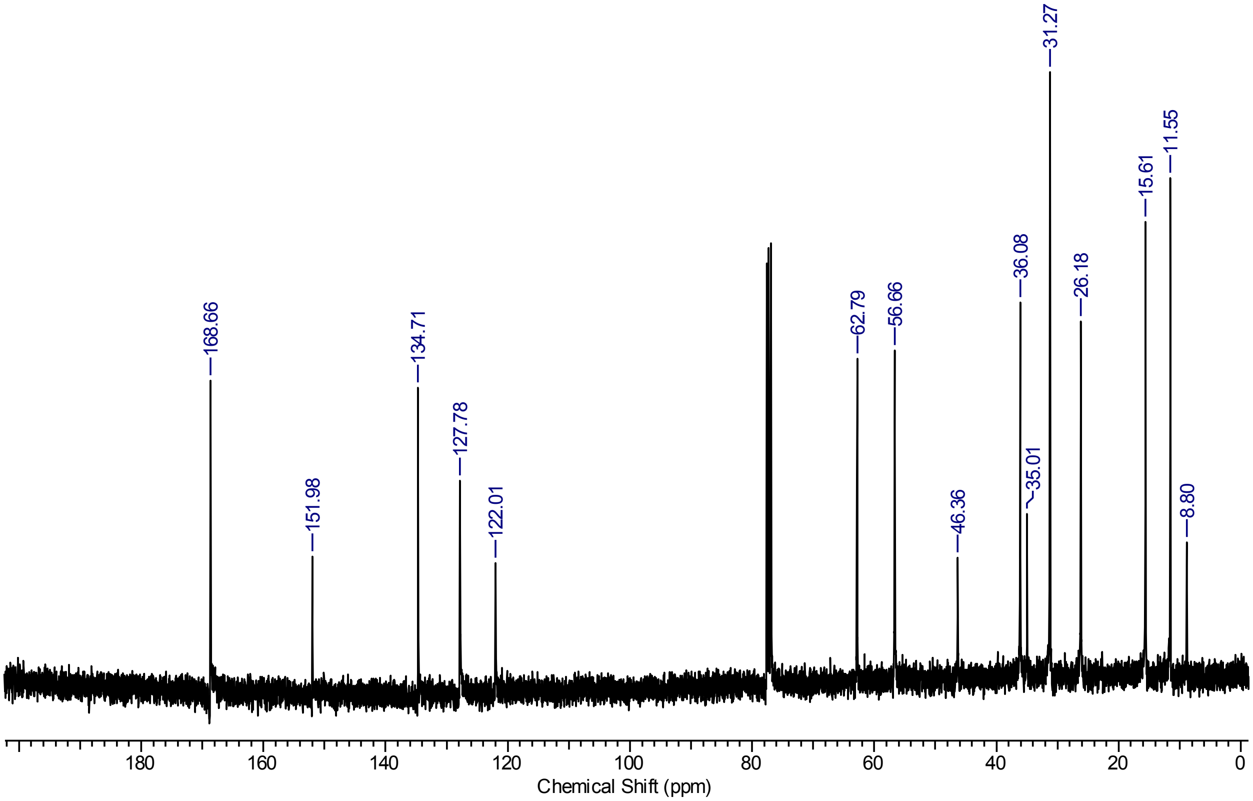

5-(*tert*-butyl)-N1,N3-bis((*S*)-1-cyclohexyl-2-hydroxyethyl)isophthalamide (S5).

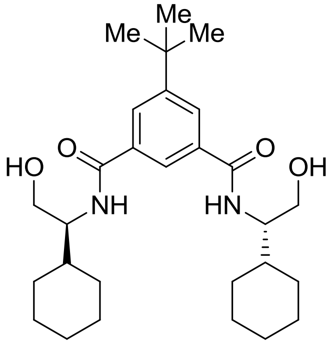

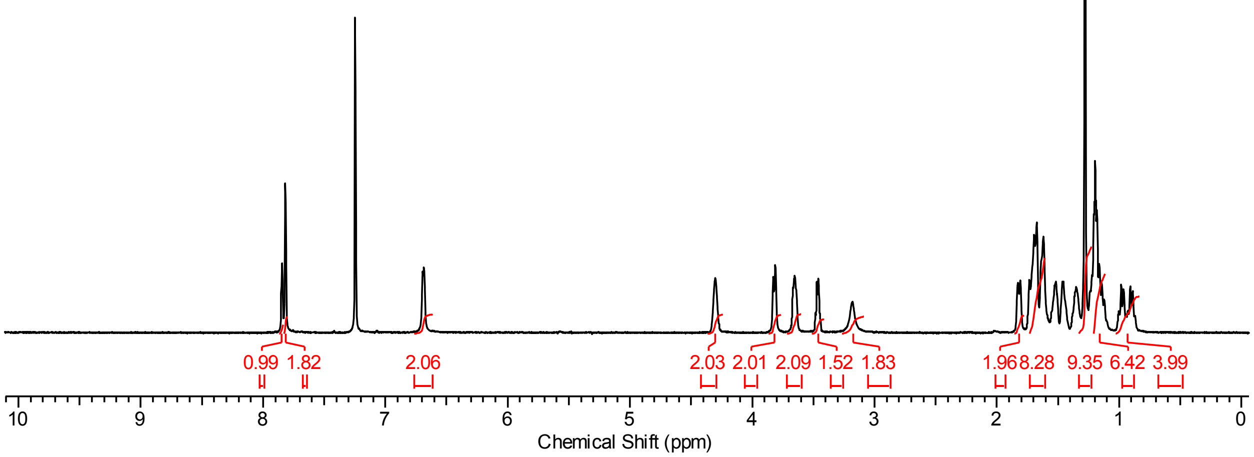

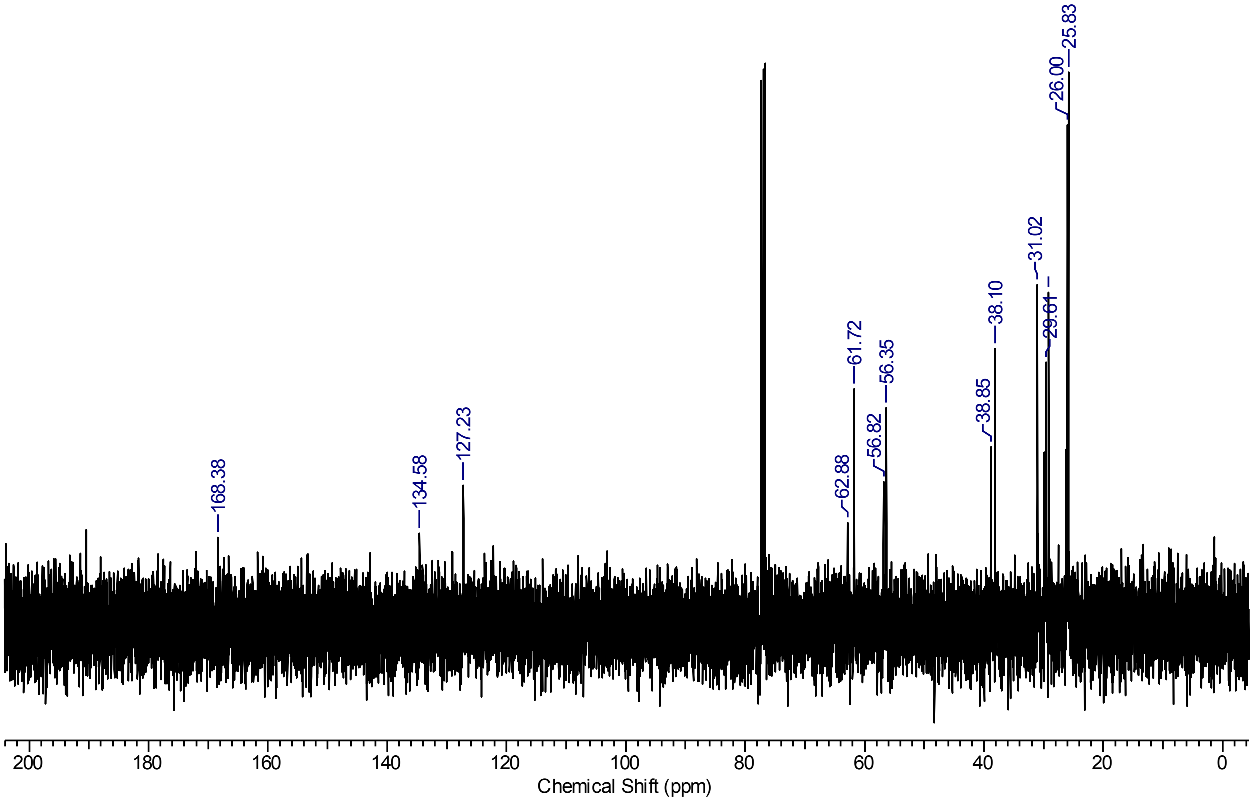

5-(*tert*-butyl)-N1,N3-bis((*S*)-1-cyclohexyl-3-hydroxypropan-2-yl)isophthalamide (S6).

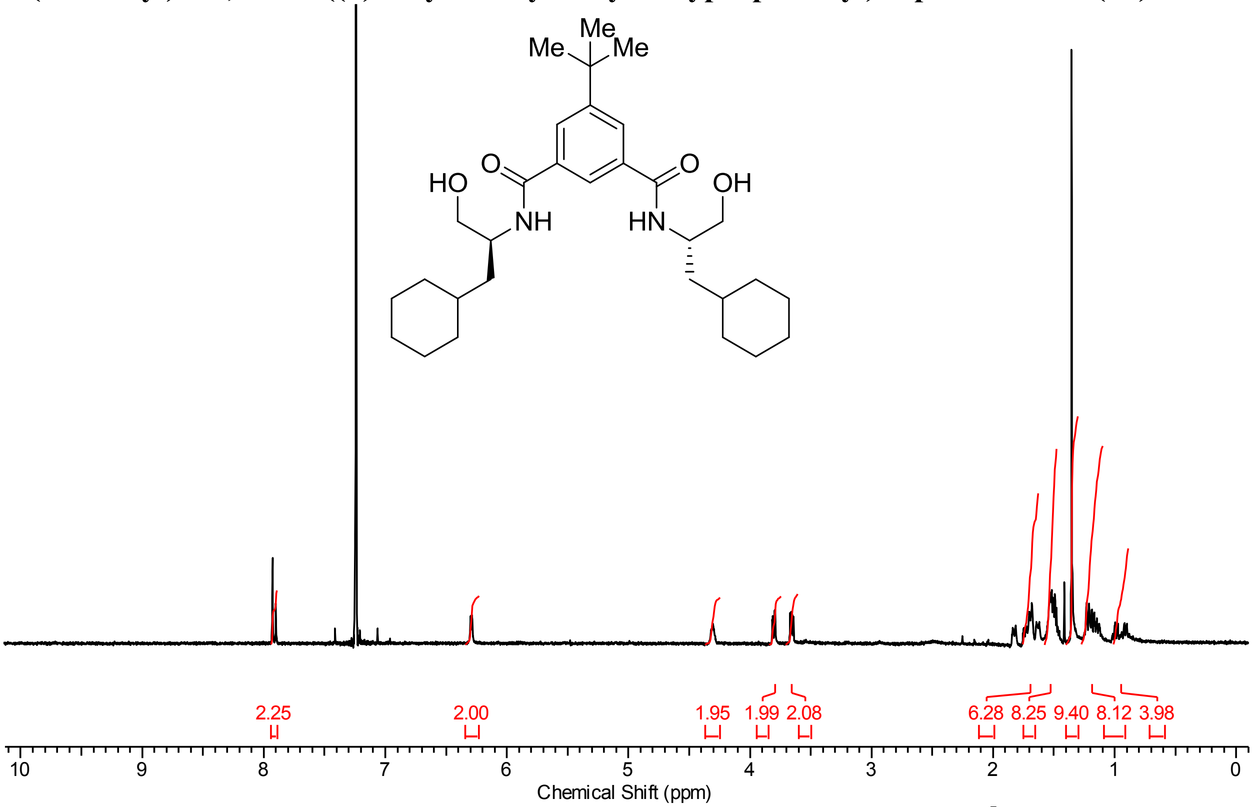

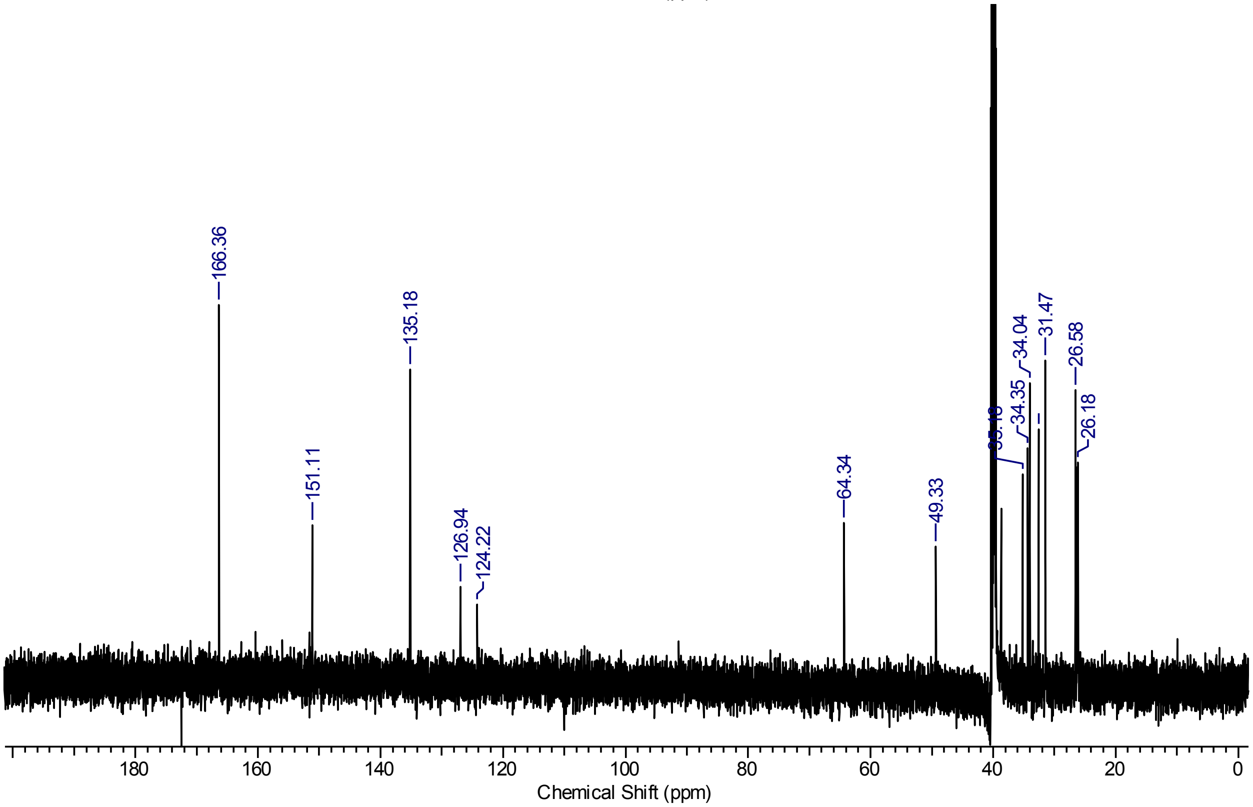

5-(*tert*-butyl)-*N*1,*N*3-bis((*S*)-1-hydroxy-3-phenylpropan-2-yl)isophthalamide (S7).

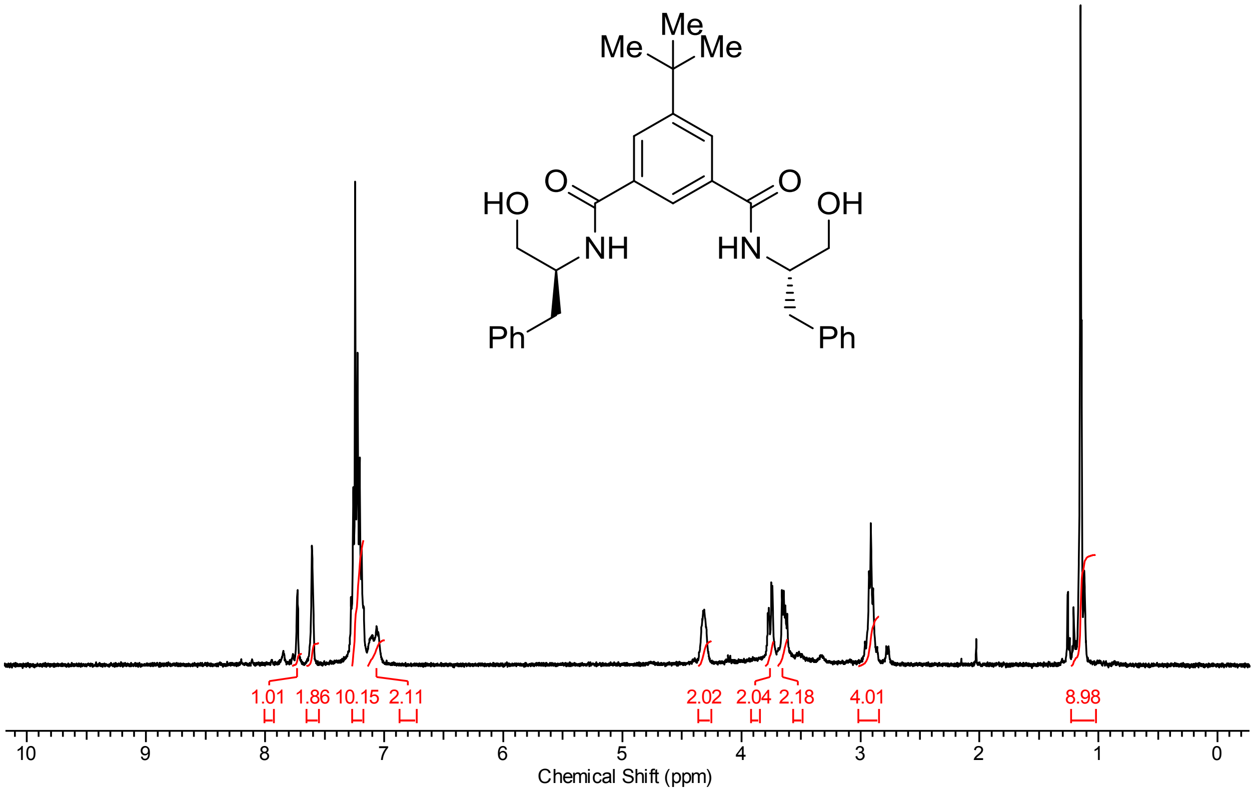

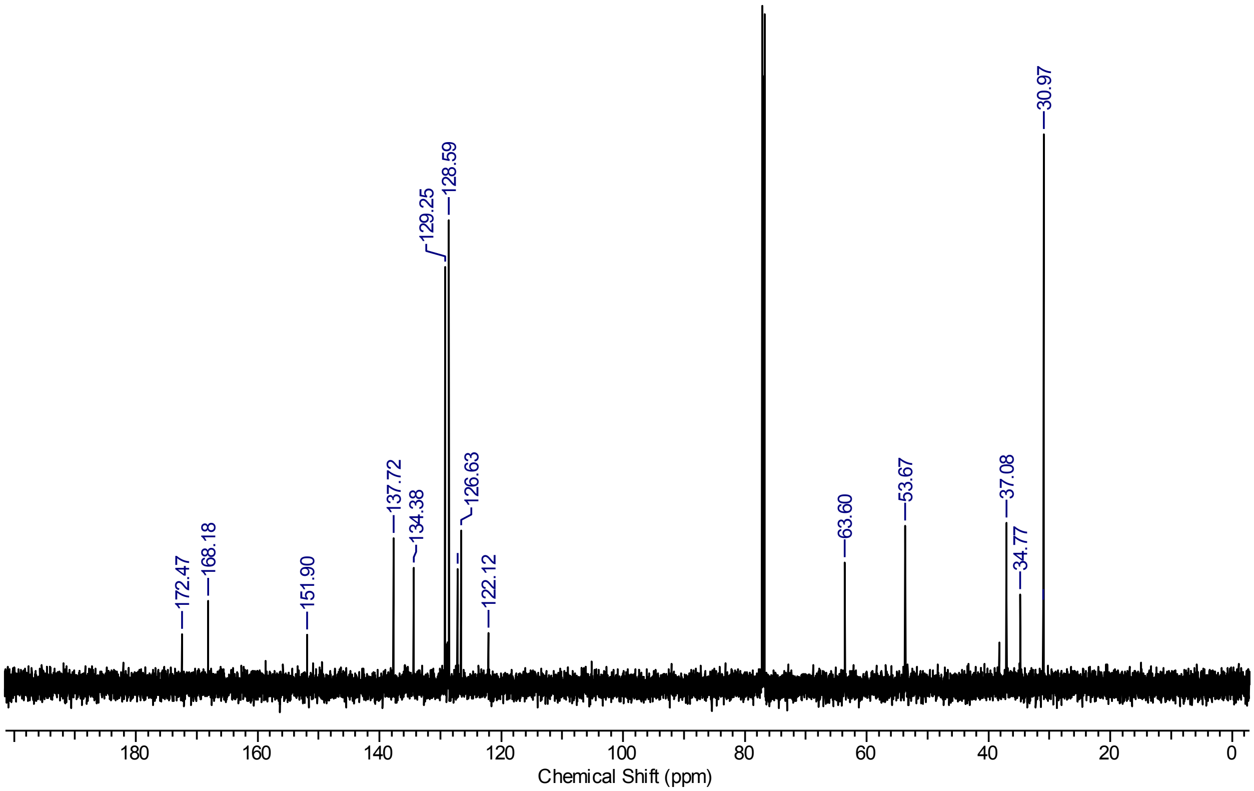

(*S*)-2-((*tert*-butoxycarbonyl)amino)-4,4-dimethylpentanoic acid (S8).

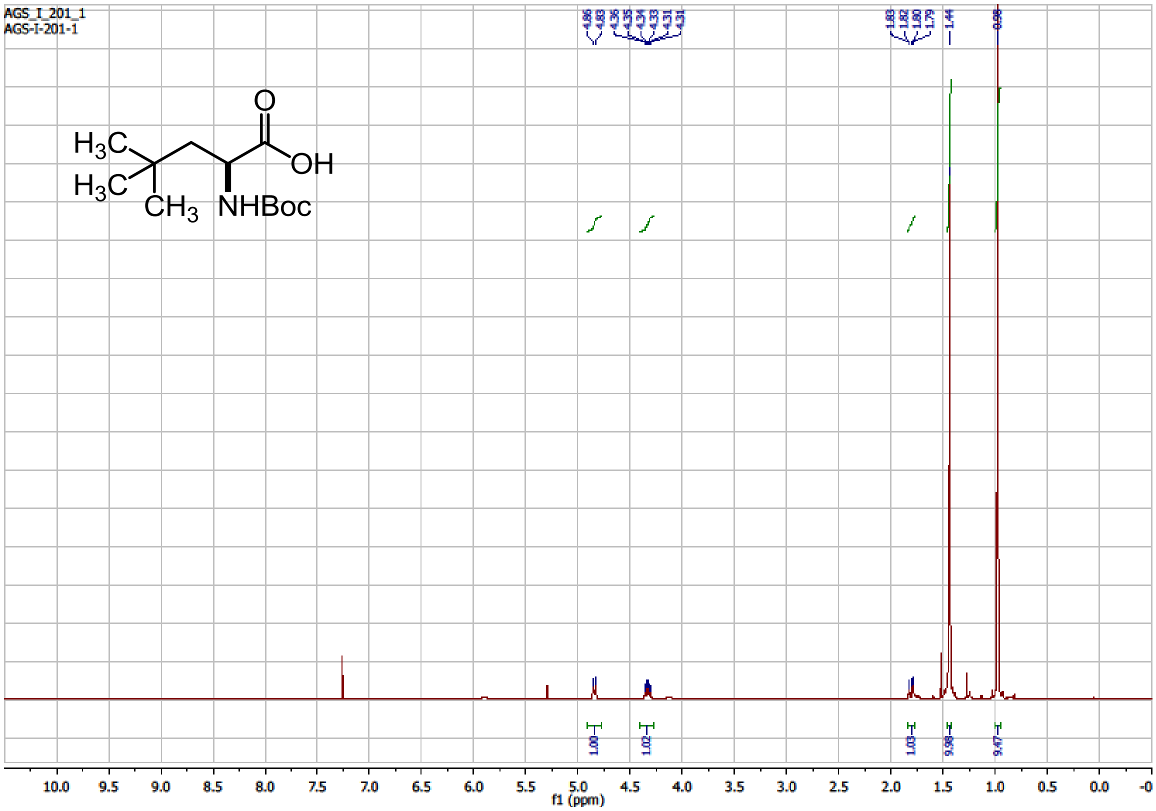

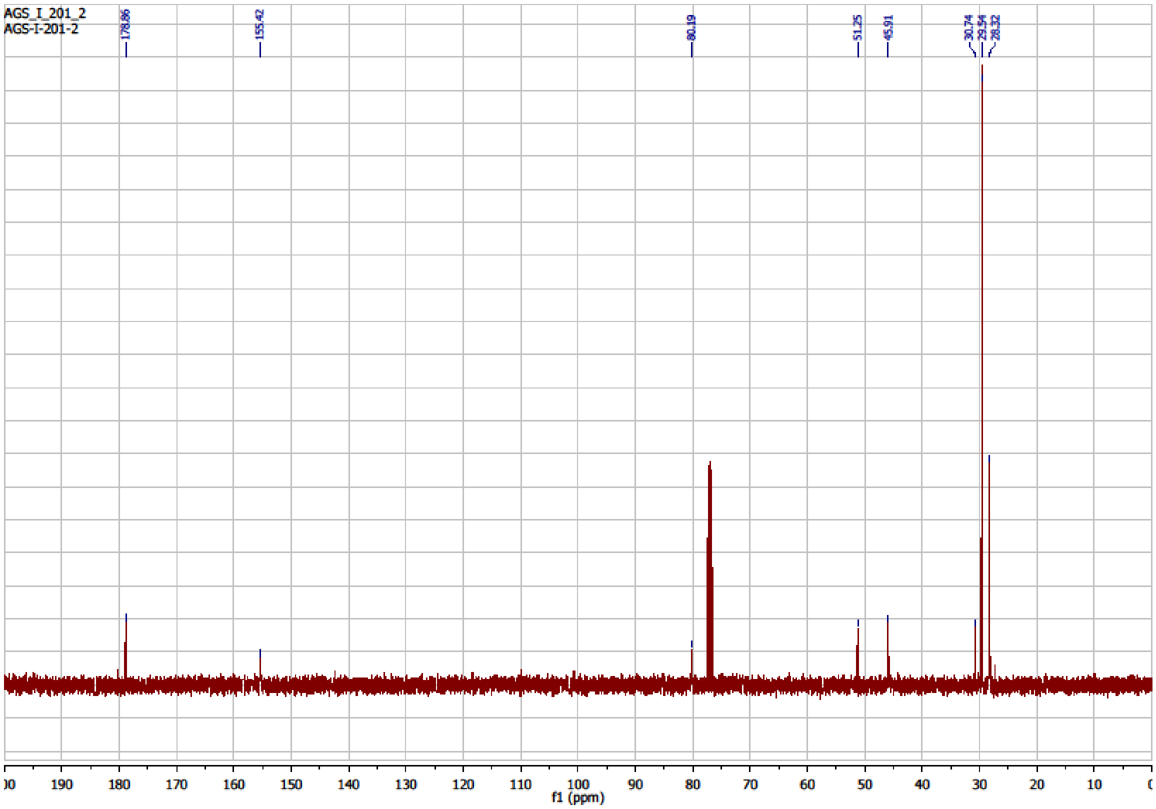

5-(*tert*-butyl)-N^1,N^3-bis((*S*)-1-hydroxy-4,4-dimethylpentan-2-yl)isophthalamide (S11).

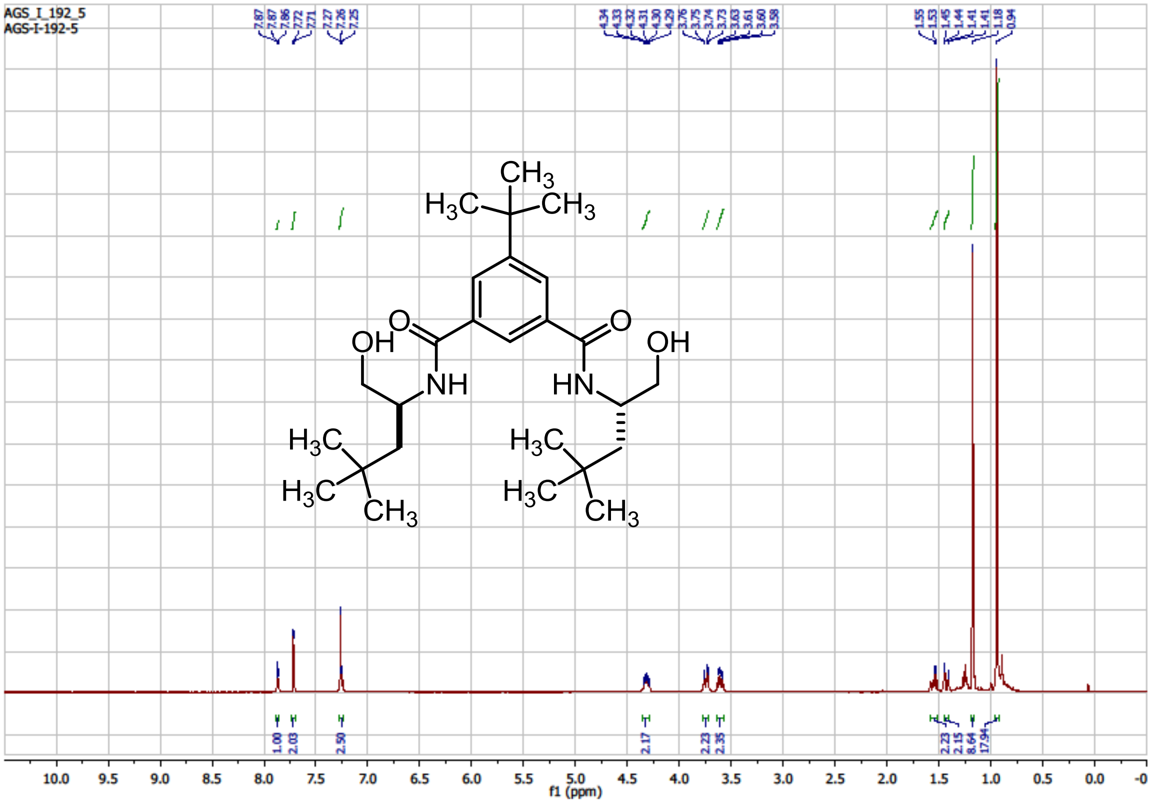

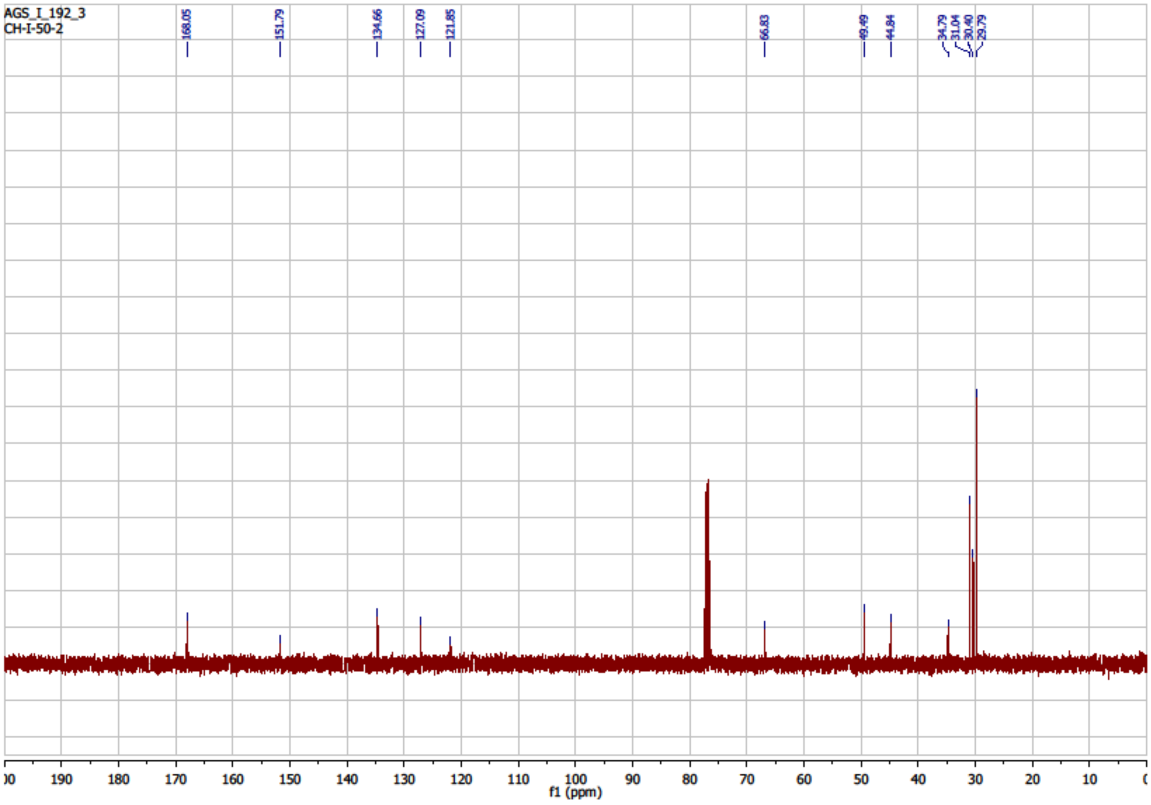

(4S,4'S)-2,2'-(5-(tert-butyl)-1,3-phenylene)bis(4-isopropyl-1-phenyl-4,5-dihydro-1H-imidazole) (S12).

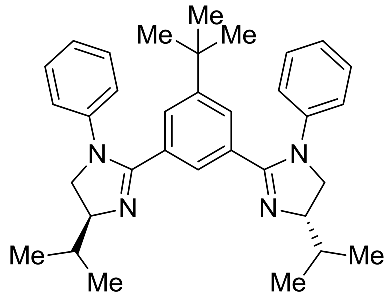

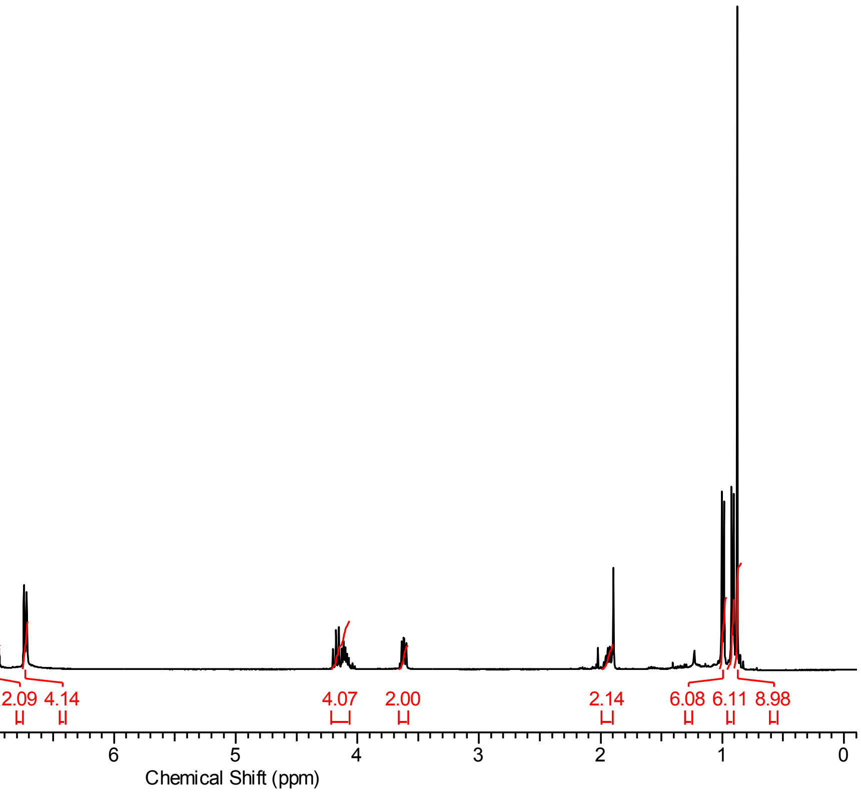

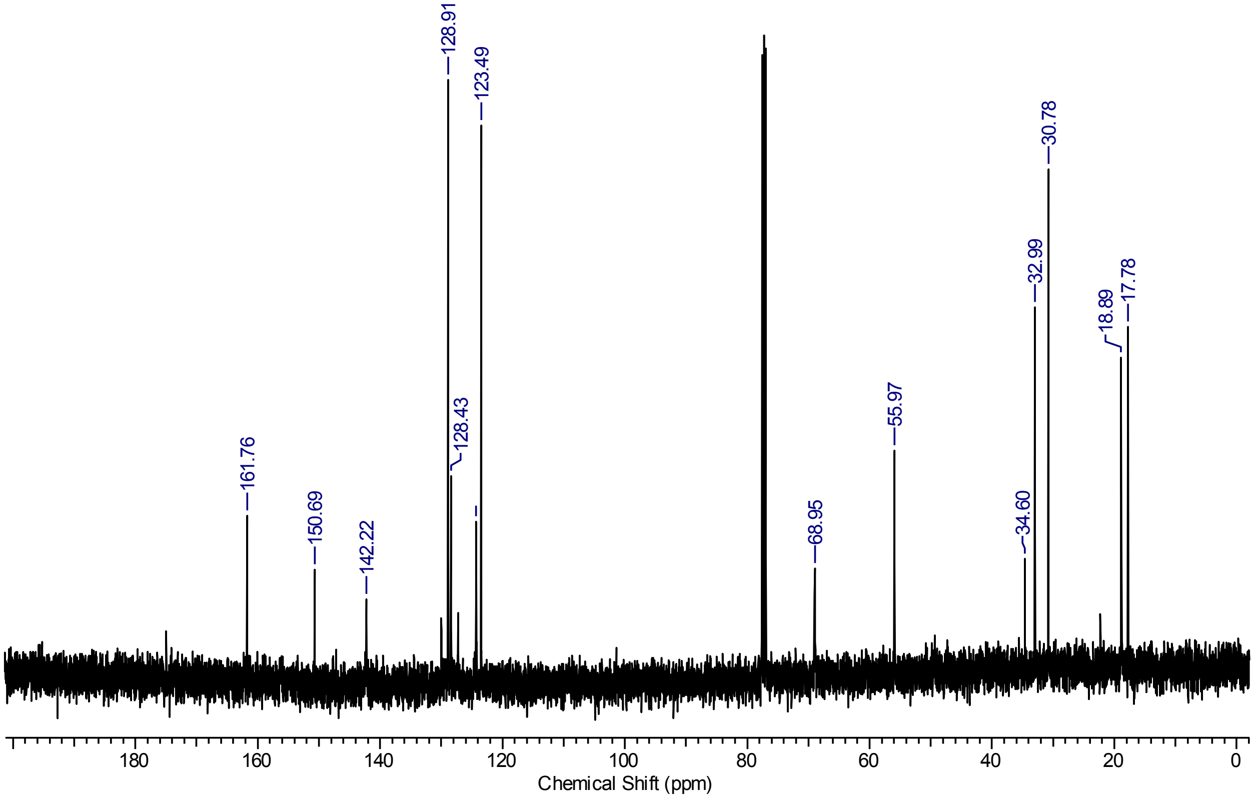

(4*S*,4'*S*)-2,2'-(5-(*tert*-butyl)-1,3-phenylene)bis(4-isopropyl-1-(4-methoxyphenyl)-4,5-dihydro-1H-imidazole) (S13).

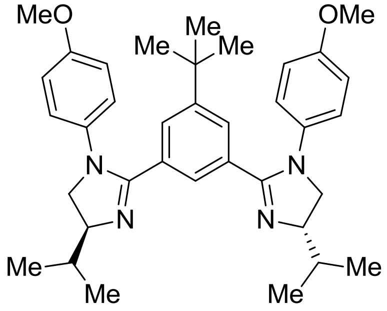

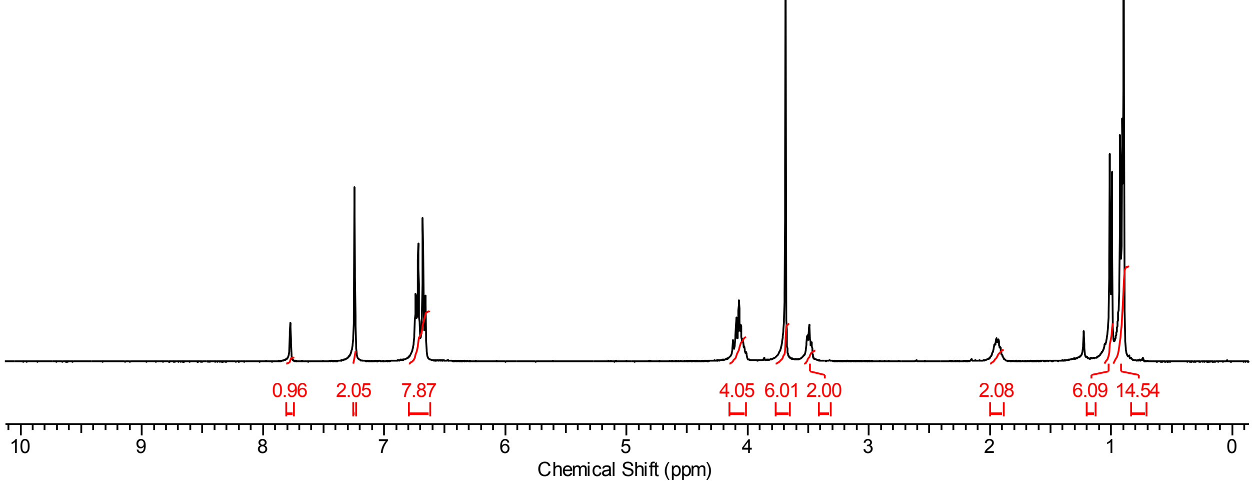

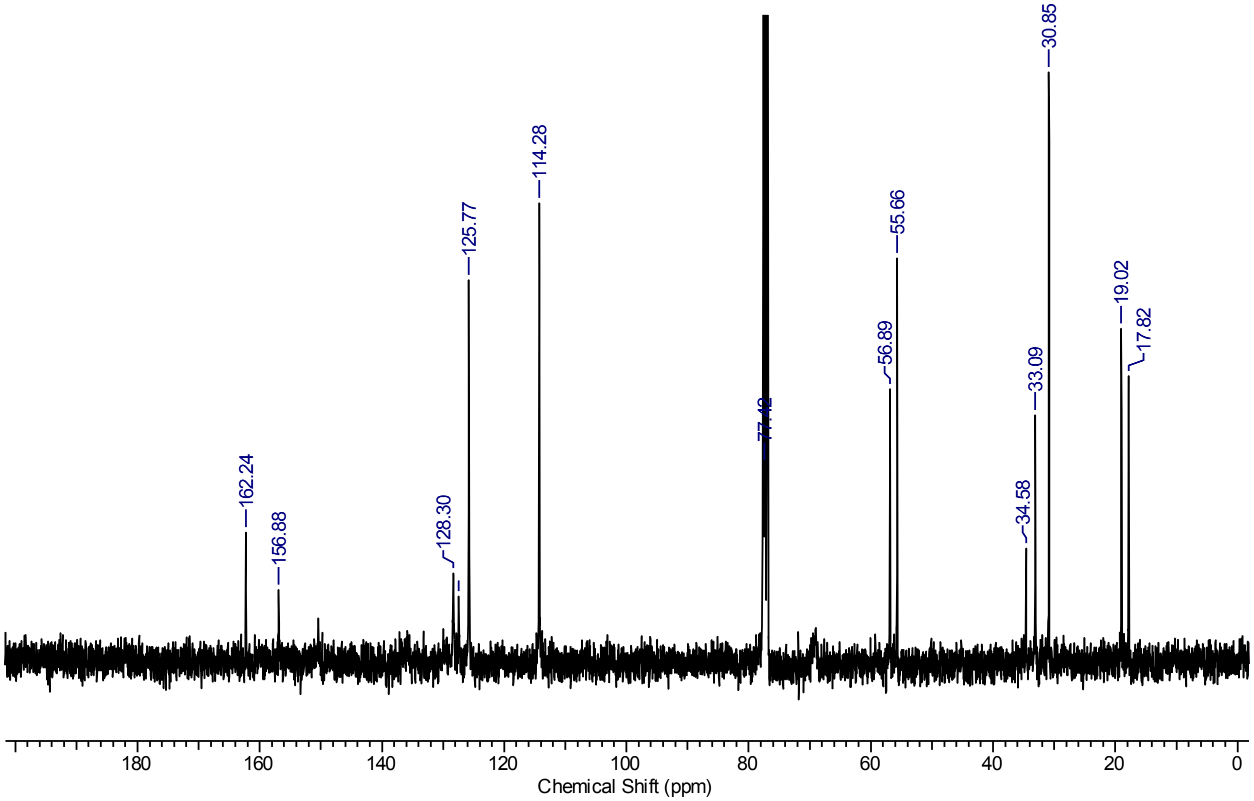

(4*S*,4'*S*)-2,2'-(5-(*tert*-butyl)-1,3-phenylene)bis(4-isopropyl-1-(4-nitrophenyl)-4,5-dihydro-1H-imidazole) (S14).

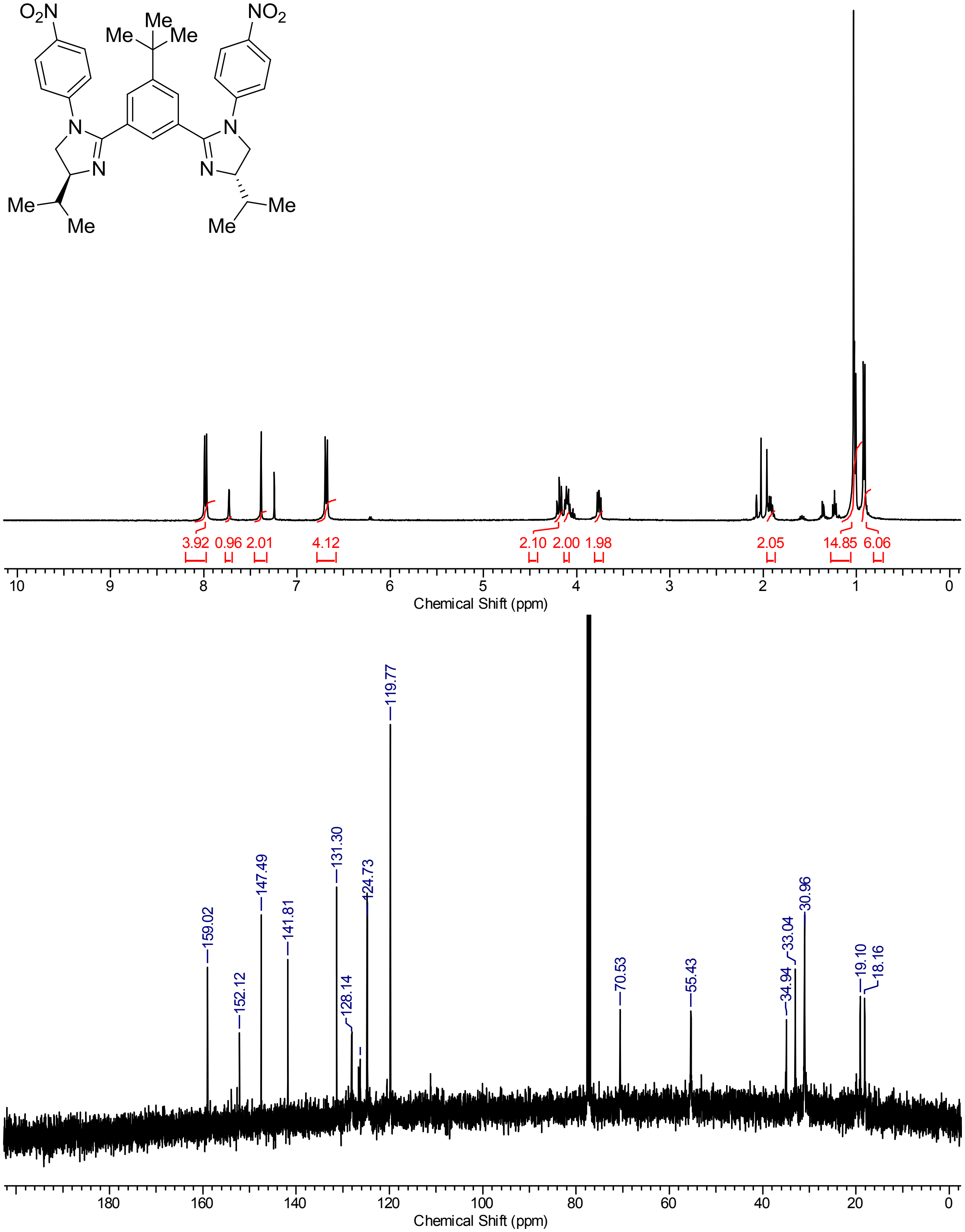

(4*S*,4'*S*)-2,2'-(5-(*tert*-butyl)-1,3-phenylene)bis(4-isopropyl-1-(4-(trifluoromethyl)phenyl)-4,5-dihydro-1H-imidazole) (S15).

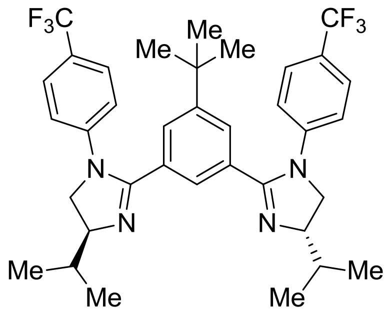

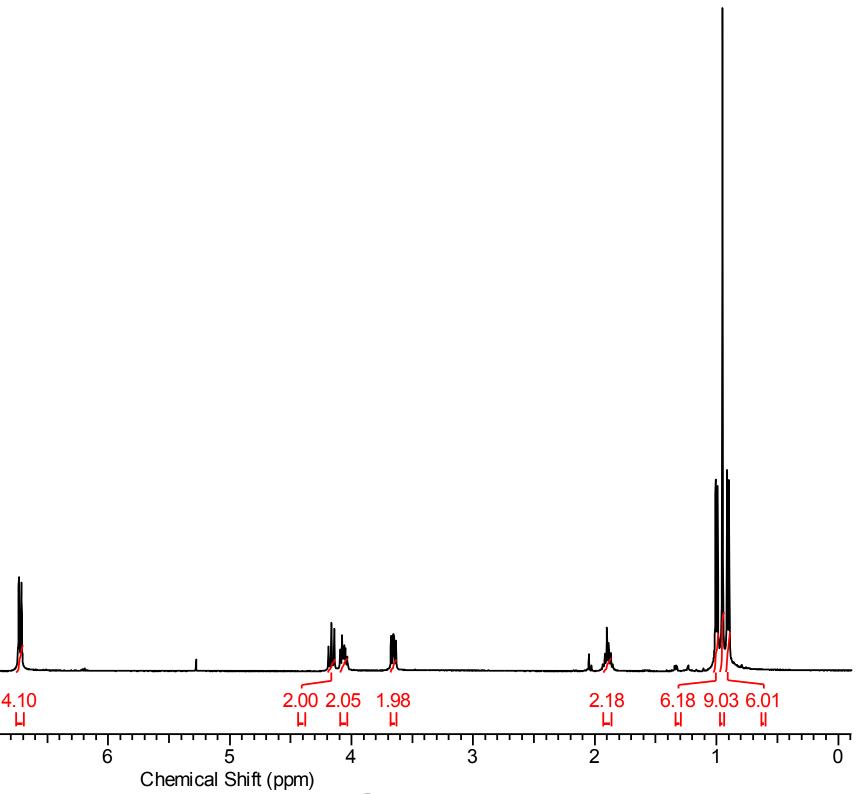

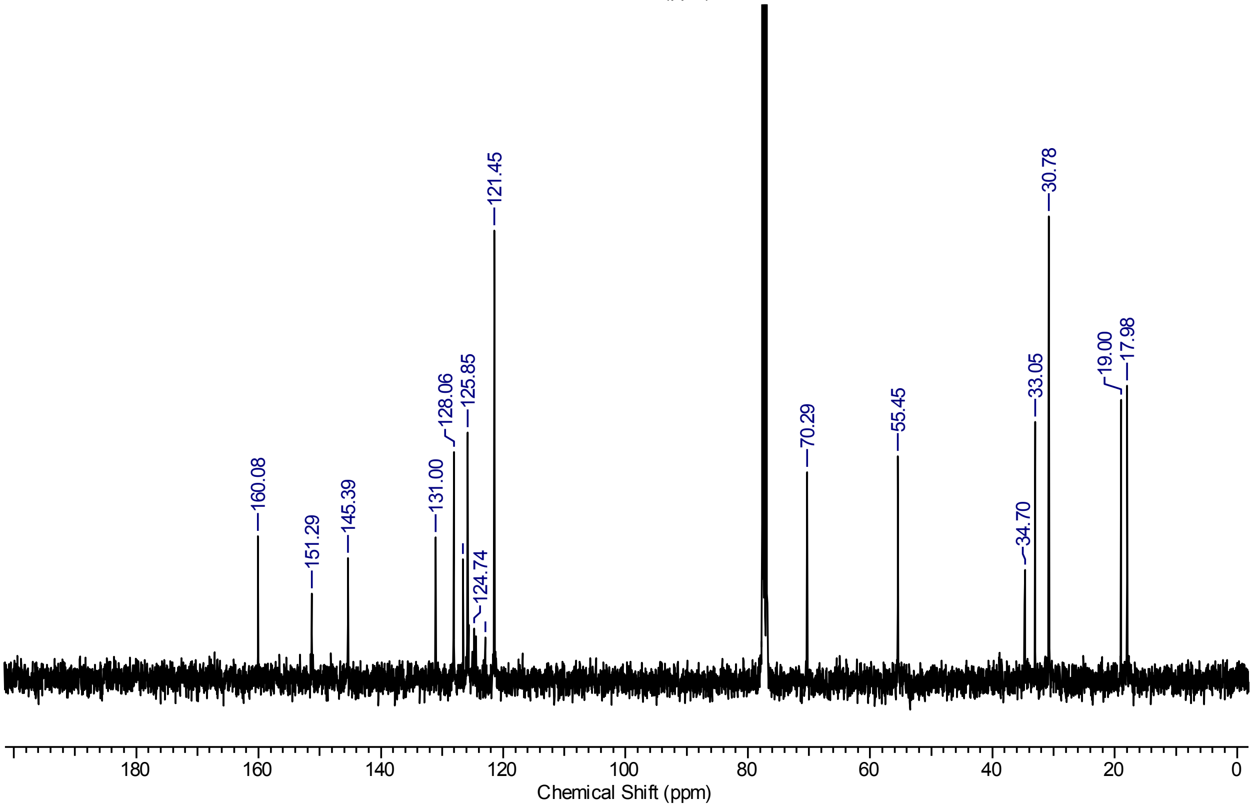

(4*S*,4'*S*)-2,2'-(5-(*tert*-butyl)-1,3-phenylene)bis(4-isobutyl-1-(4-(trifluoromethyl)phenyl)-4,5-dihydro-1H-imidazole) (S16).

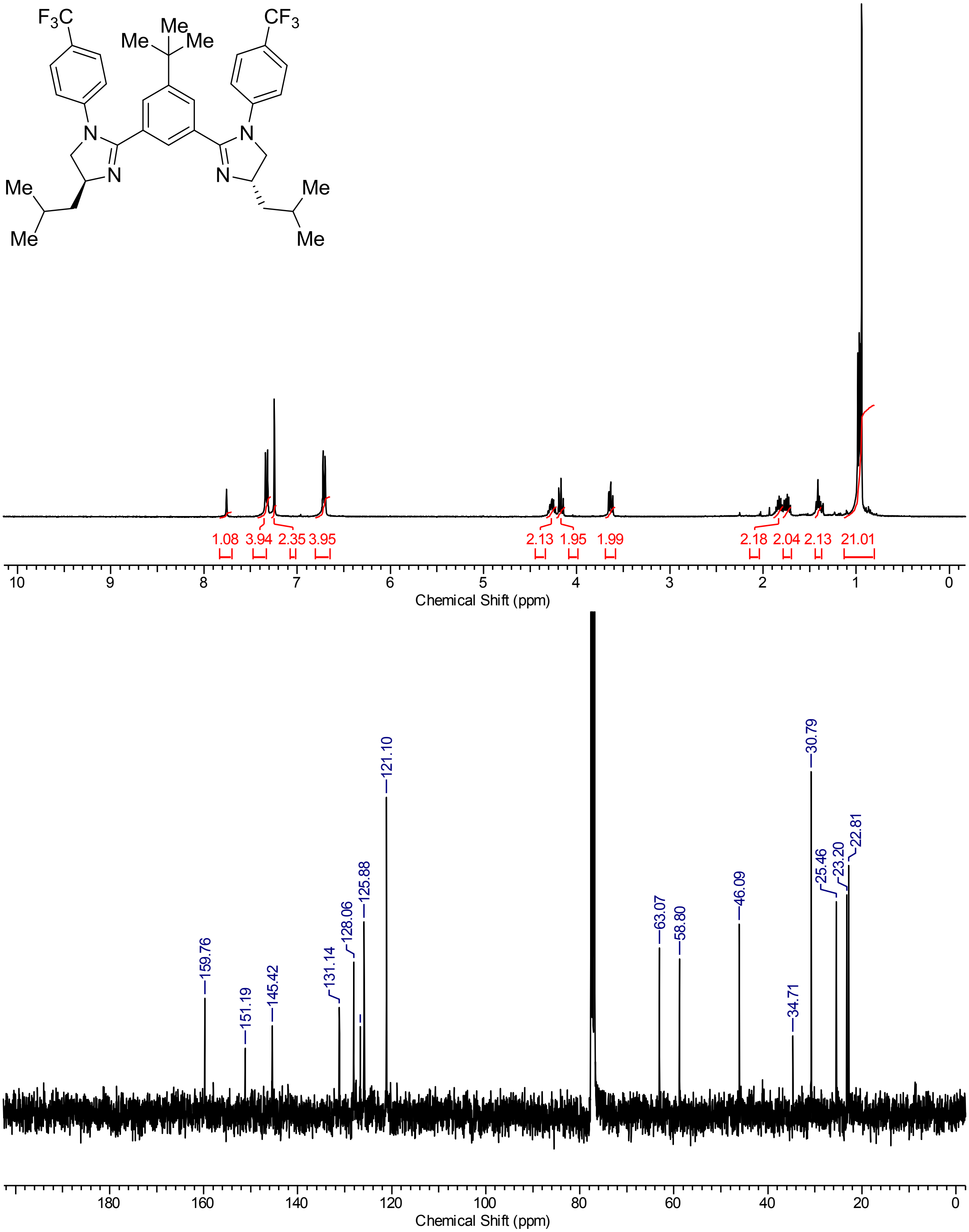

(4*R*,4'*R*)-2,2'-(5-(*tert*-butyl)-1,3-phenylene)bis(4-butyl-1-(4-(trifluoromethyl)phenyl)-4,5-dihydro-1H-imidazole) (S17).

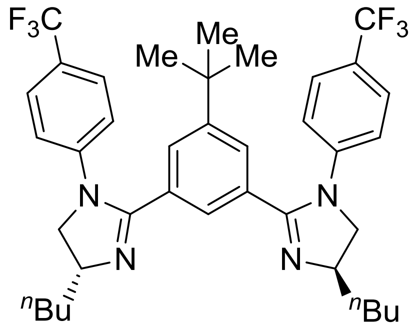

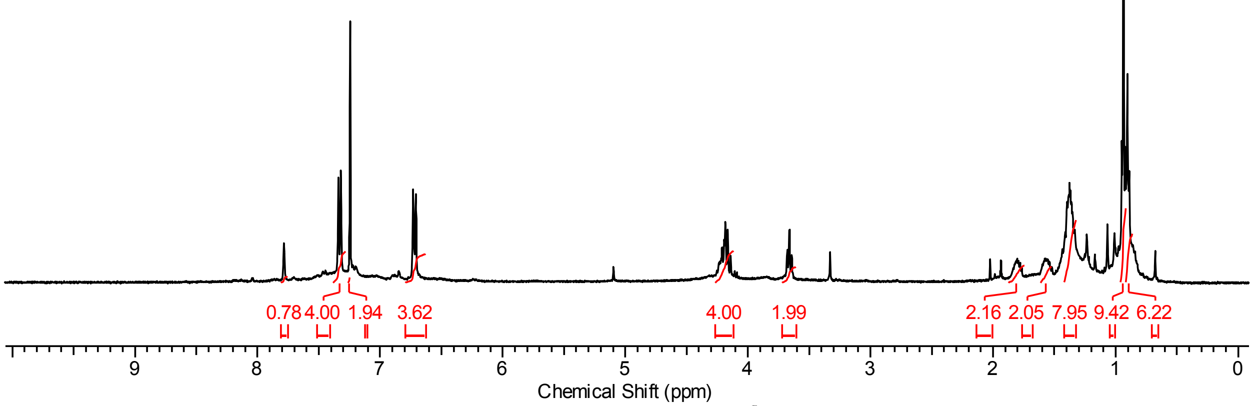

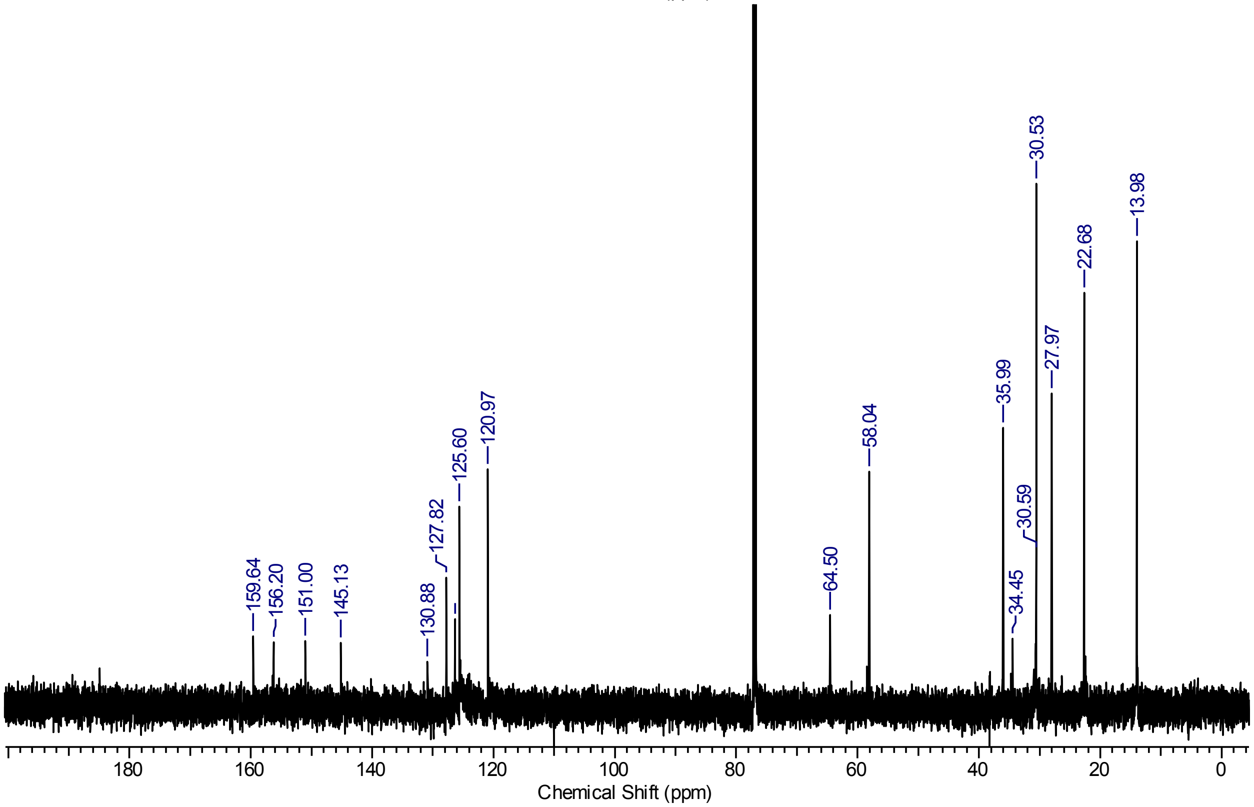

(*S*)-4-((*S*)-*sec*-butyl)-2-(3-(*tert*-butyl)-5-((*S*)-4-((*S*)-*sec*-butyl)-1-(4-(trifluoromethyl)phenyl)-4,5-dihydro-1H-imidazol-2-yl)phenyl)-4-methyl-1-(4-(trifluoromethyl)phenyl)-4,5-dihydro-1H-imidazole (S18).

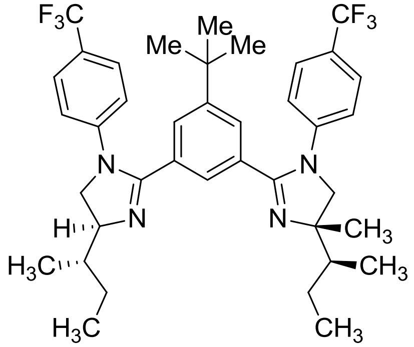

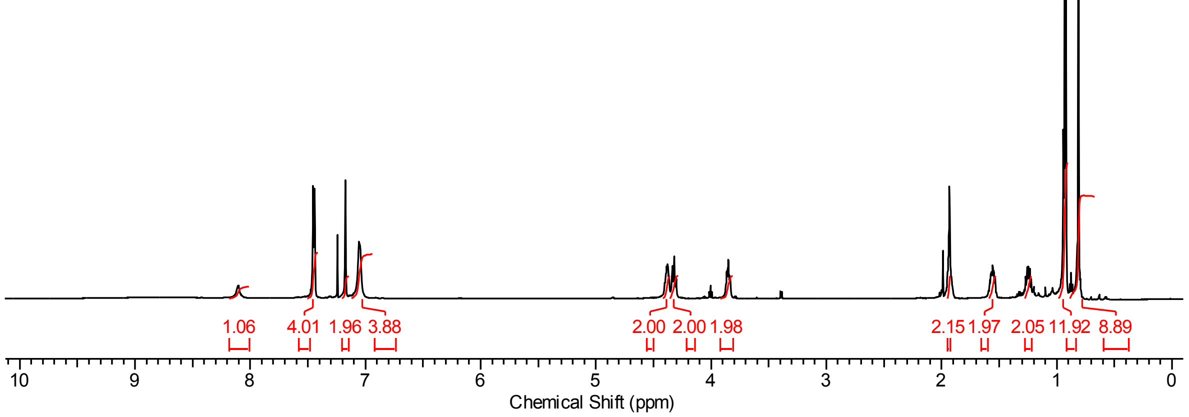

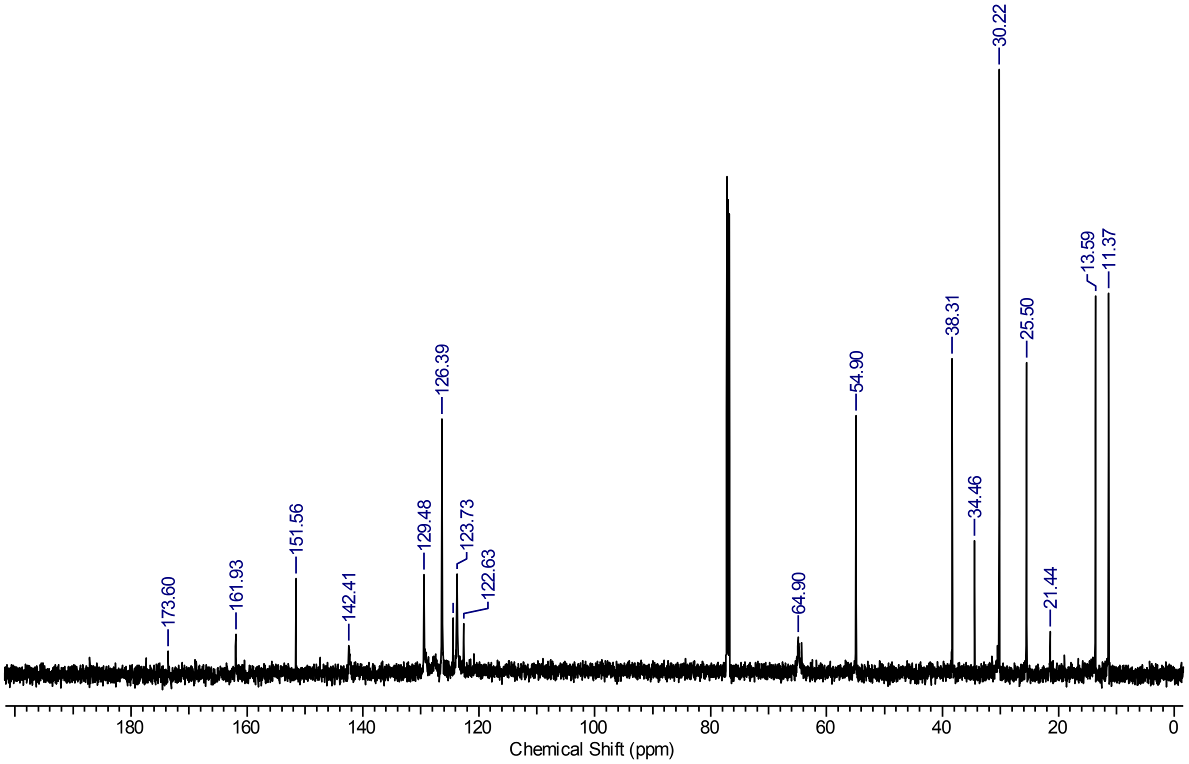

(4*S*,4'*S*)-2,2'-(5-(*tert*-butyl)-1,3-phenylene)bis(4-cyclohexyl-1-(4-(trifluoromethyl)phenyl)-4,5-dihydro-1H-imidazole) (S19).

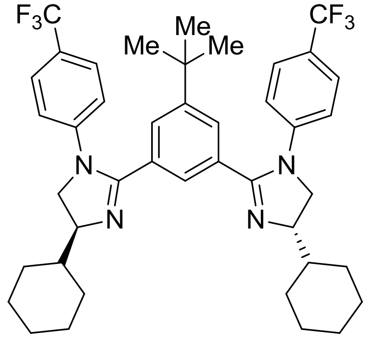

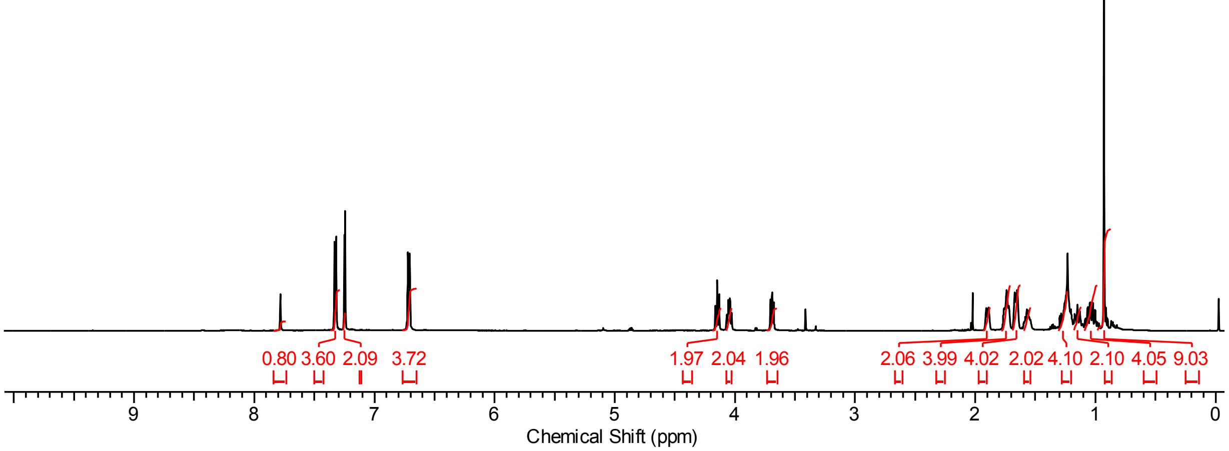

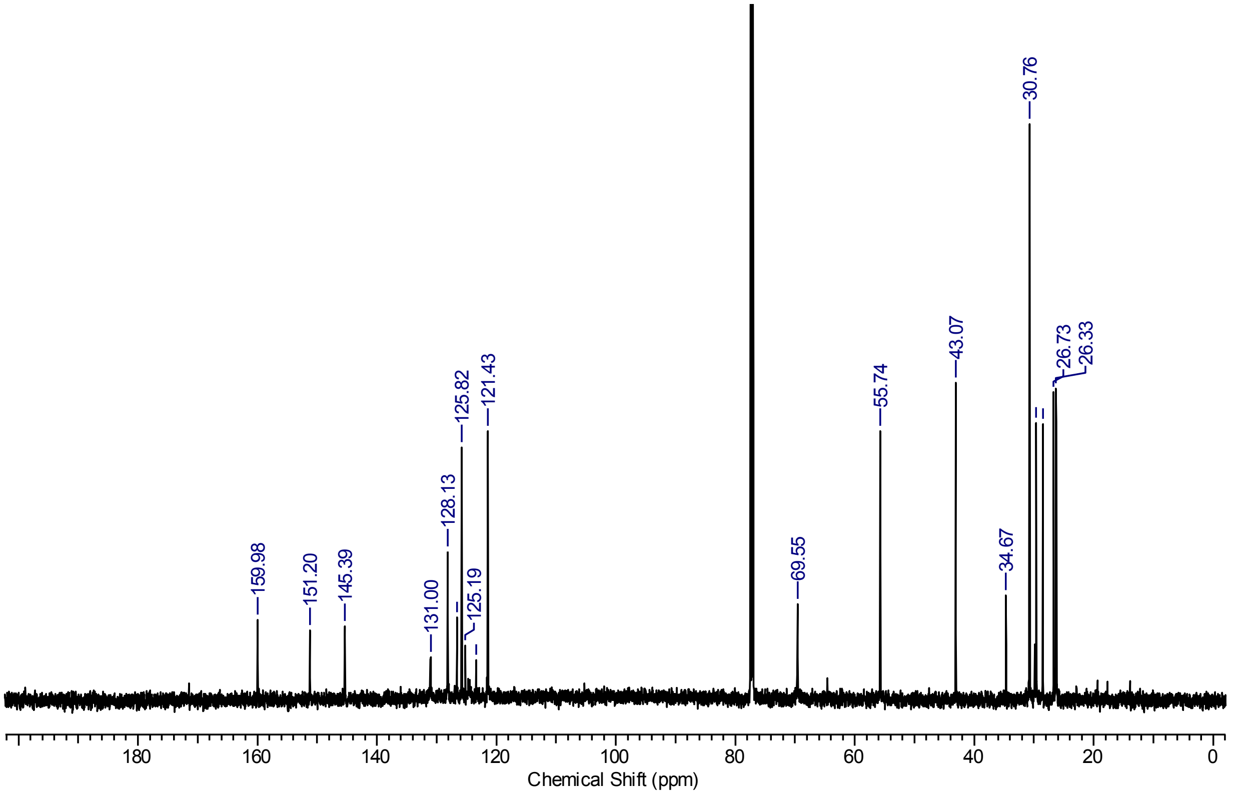

(4*S*,4'*S*)-2,2'-(5-(*tert*-butyl)-1,3-phenylene)bis(4-(cyclohexylmethyl)-1-(4-(trifluoromethyl)phenyl)-4,5-dihydro-1H-imidazole) (S20).

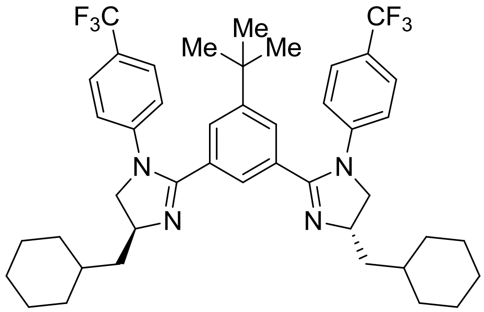

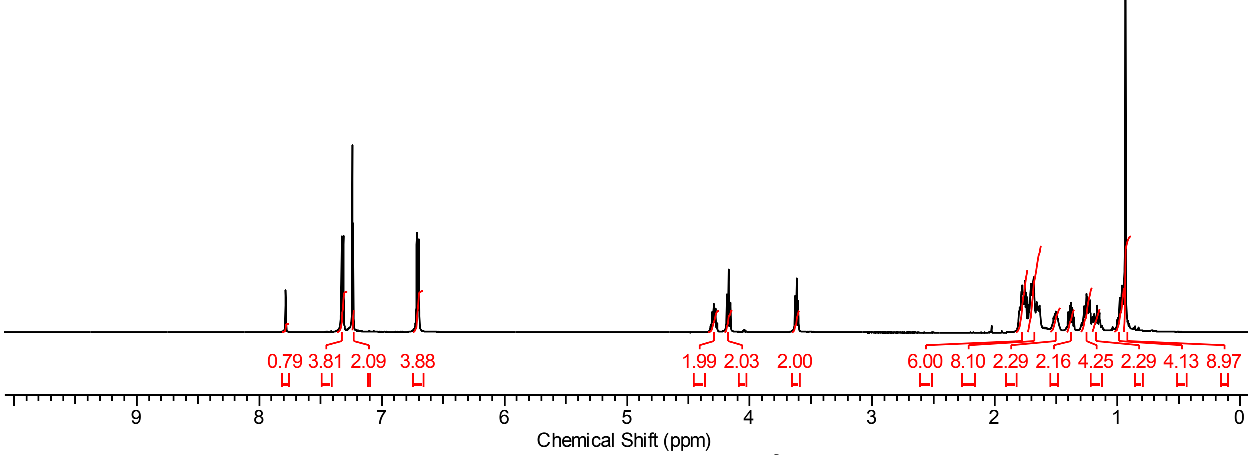

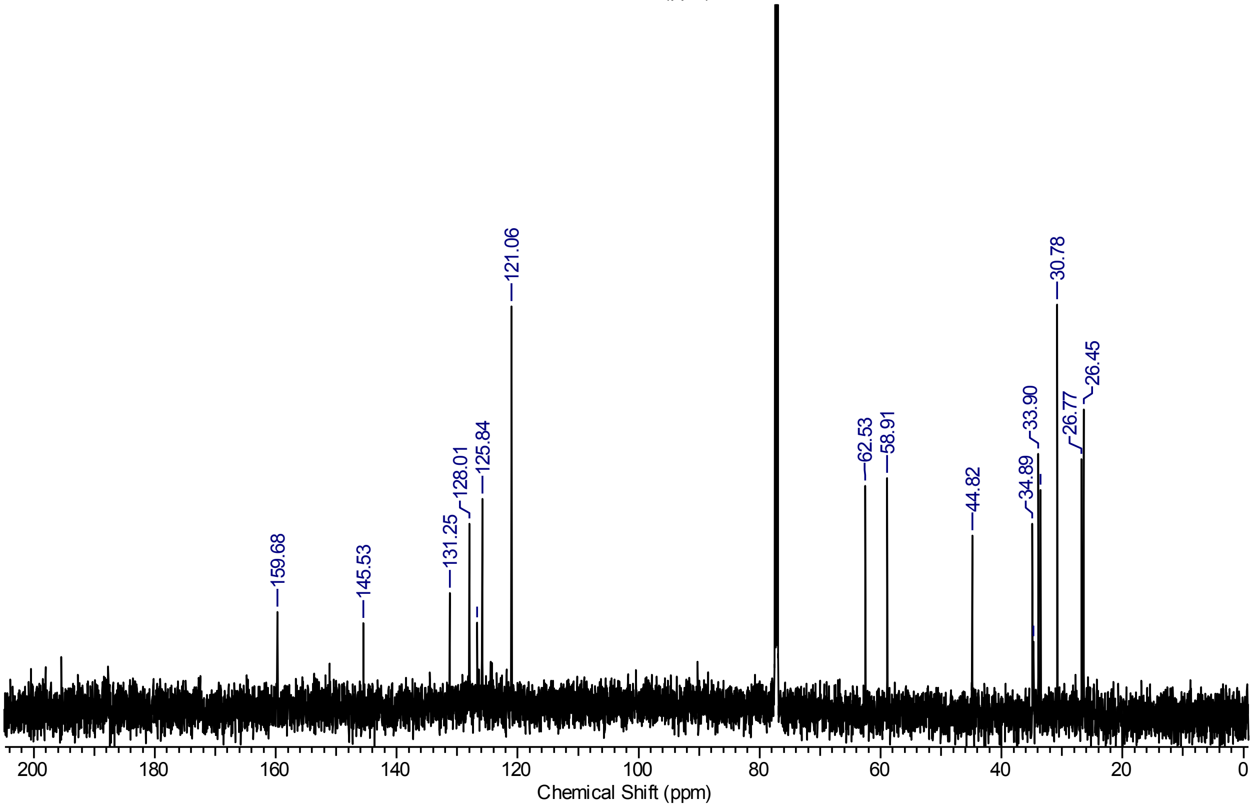

(4*S*,4'*S*)-2,2'-(5-(*tert*-butyl)-1,3-phenylene)bis(4-benzyl-1-(4-(trifluoromethyl)phenyl)-4,5-dihydro-1H-imidazole) (S21).

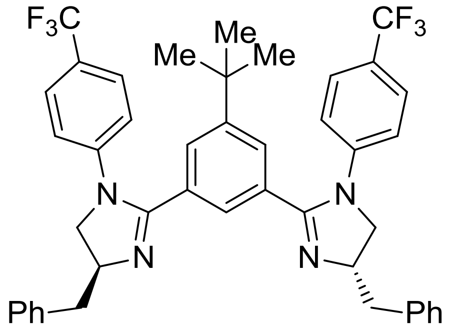

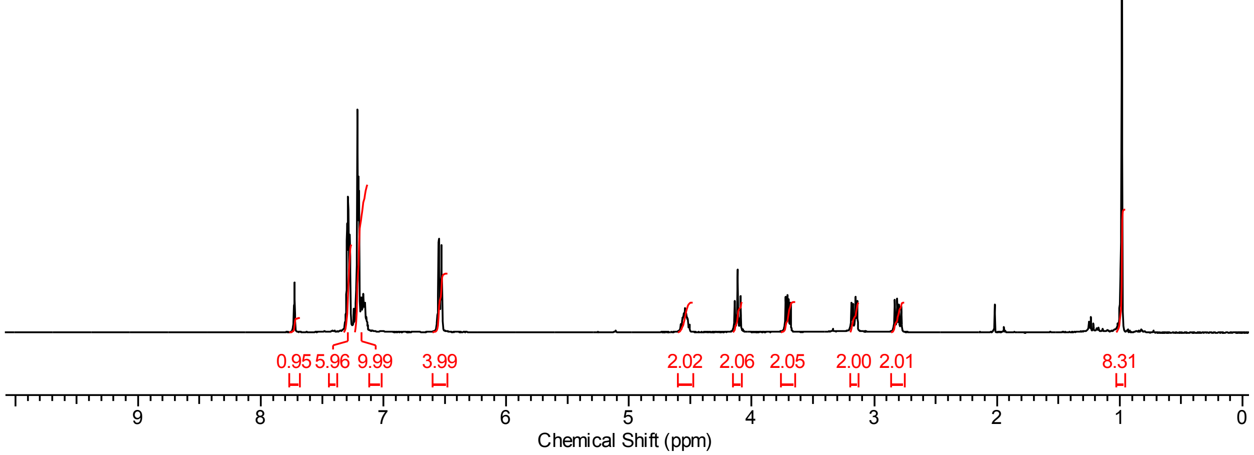

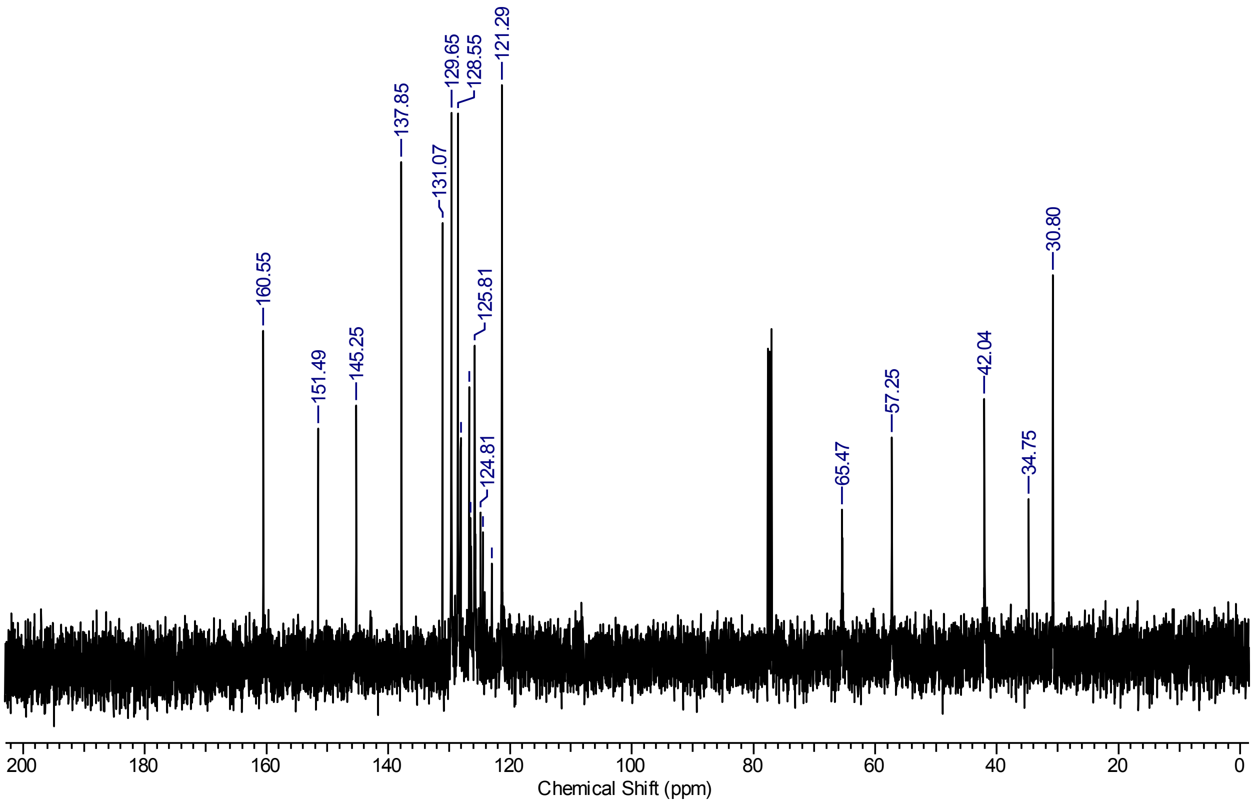

(4*S*,4'*S*)-2,2'-(5-(*tert*-butyl)-1,3-phenylene)bis(4-neopentyl-1-(4-(trifluoromethyl)phenyl)-4,5-dihydro-1H-imidazole) (S22).

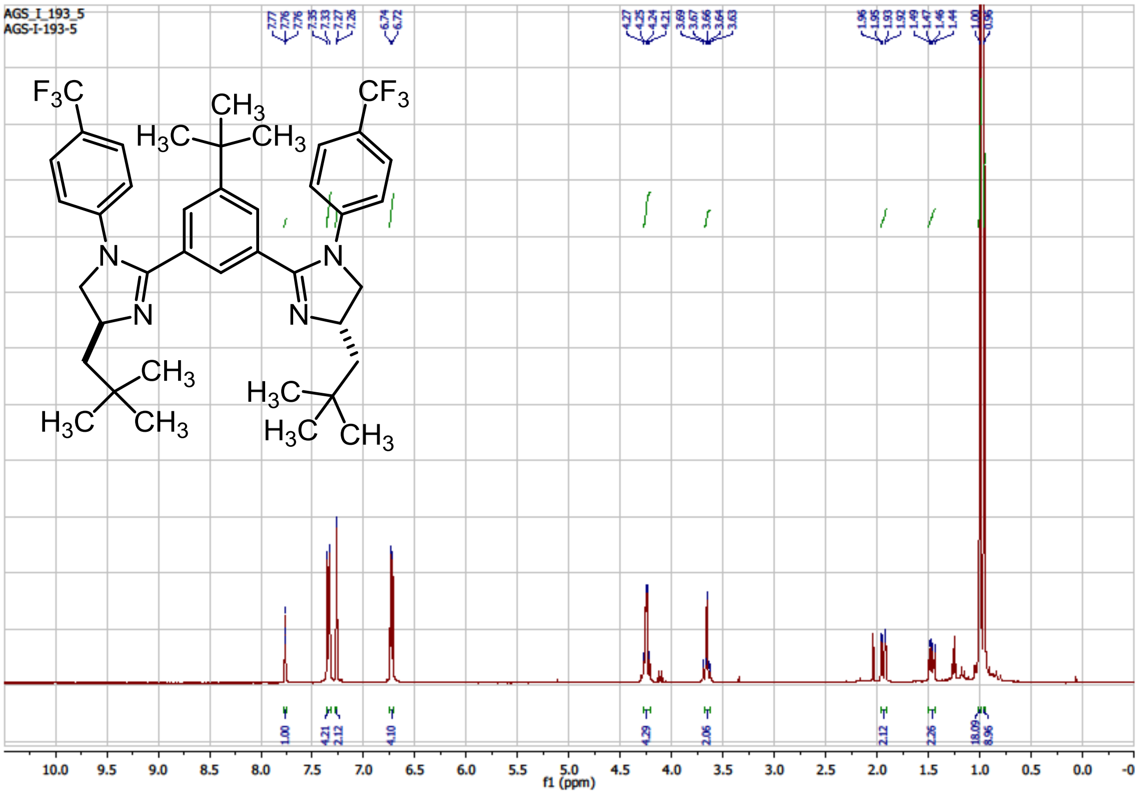

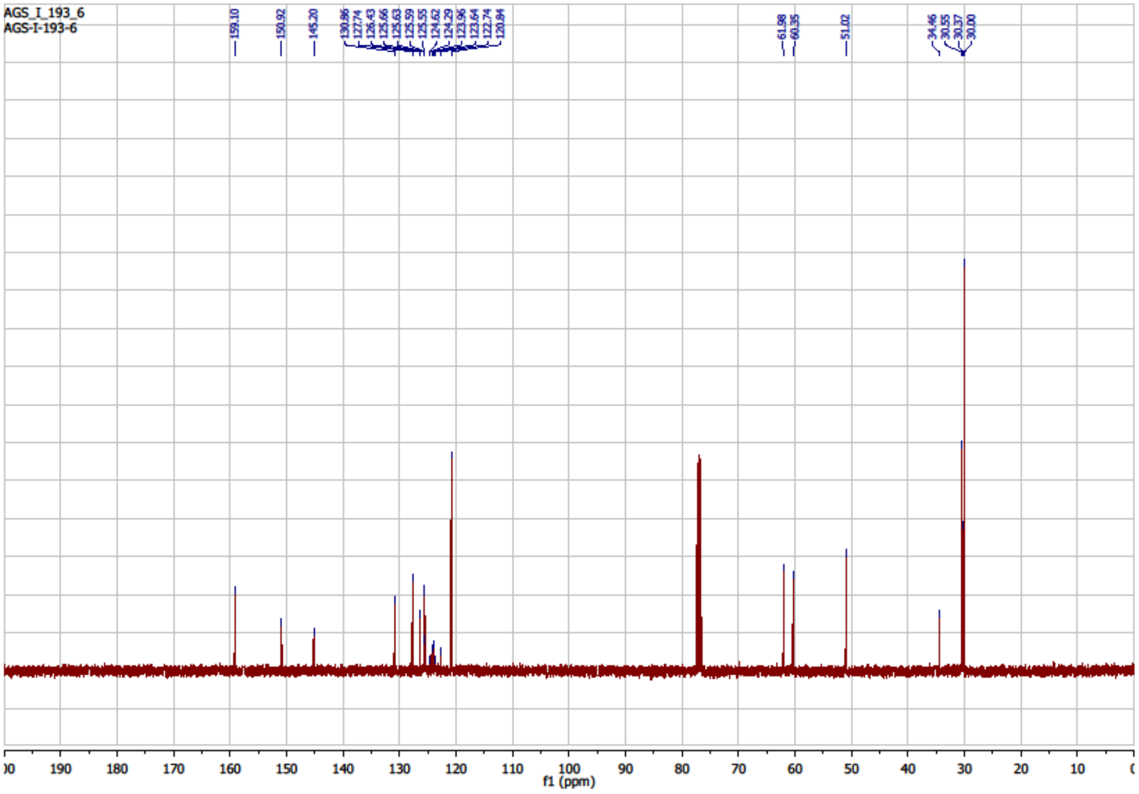

[(*R*, *R*)-*t*-ButylPhebim-Ph-*i*-Pr]$IrCl_2(H_2O)$ (5).

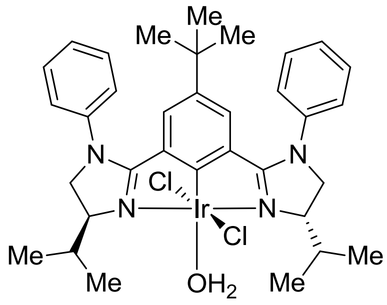

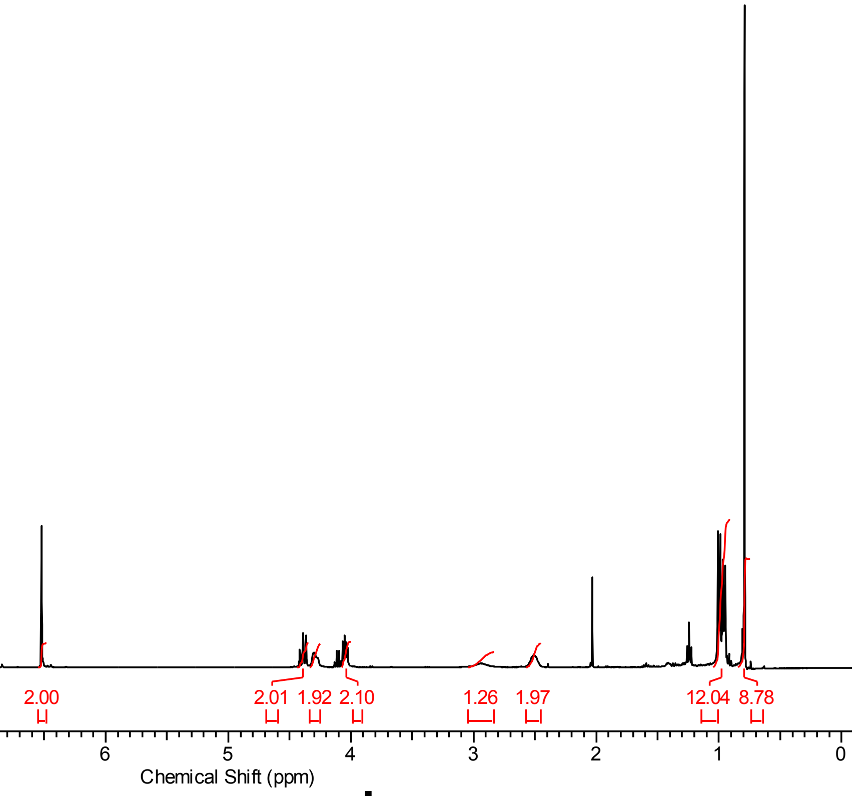

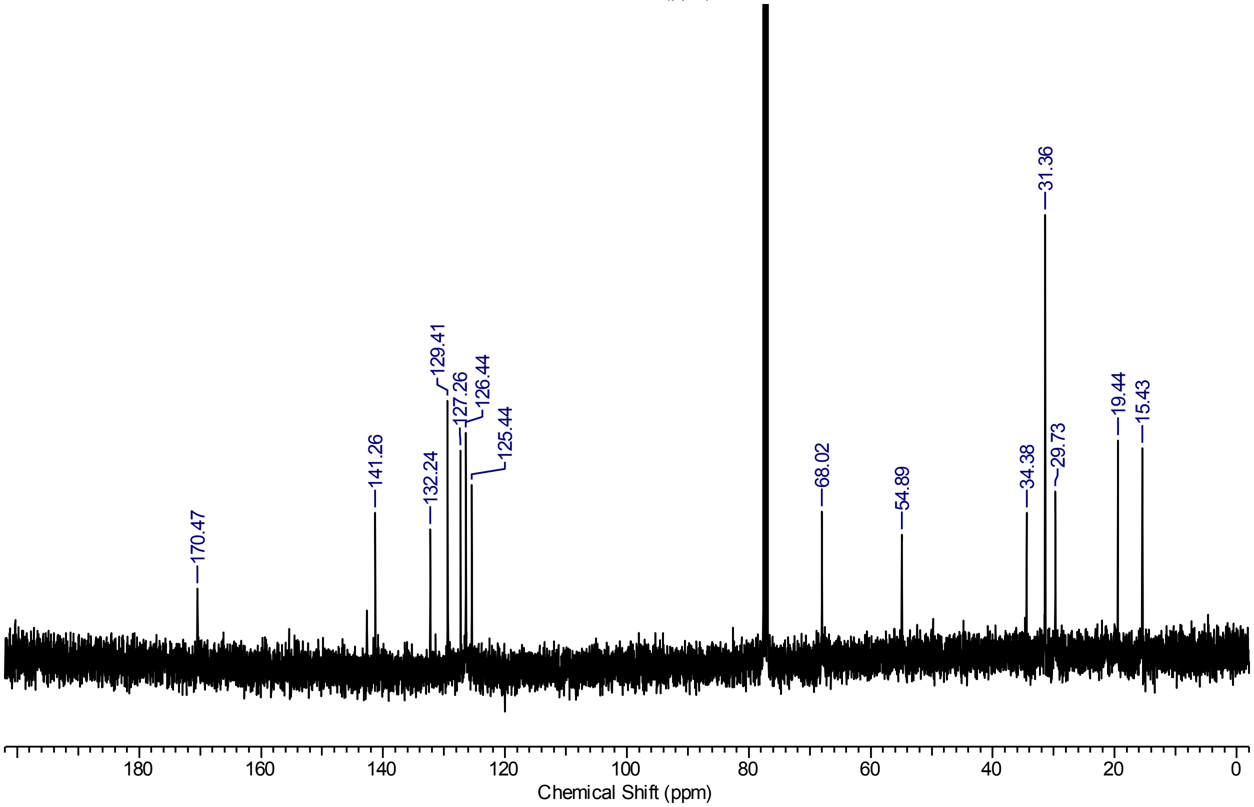

[(*R*, *R*)-*t*-ButylPhebim-4-OMe-Ph-*i*-Pr]$IrCl_2(H_2O)$ (6).

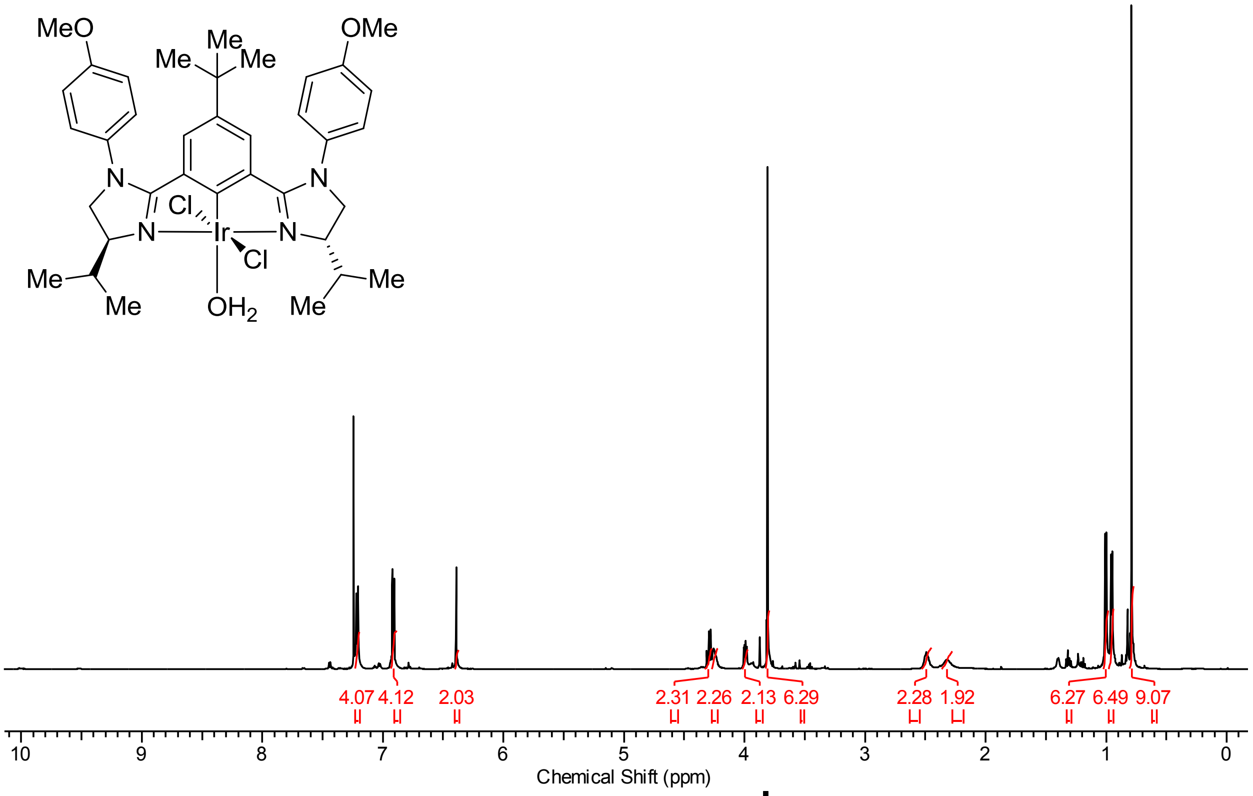

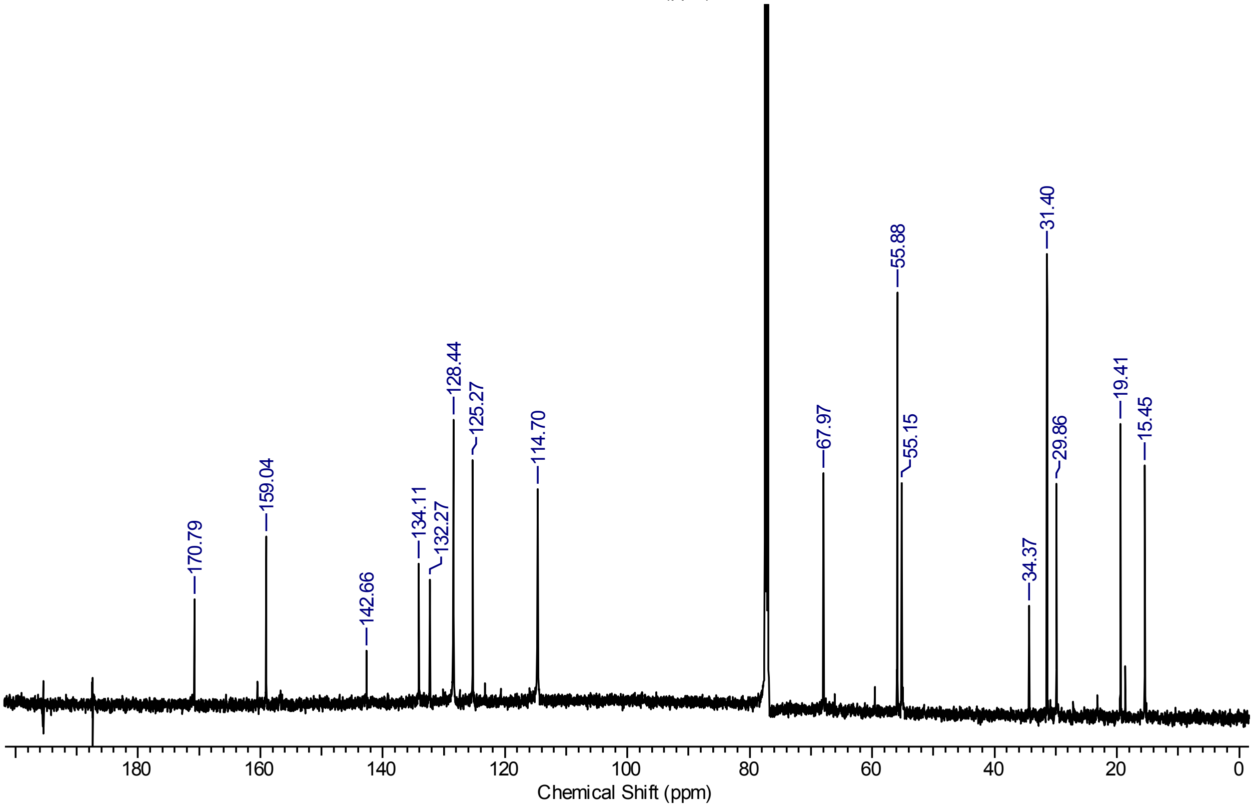

[(*R*, *R*)-*t*-ButylPhebim-4-NO_2-Ph-*i*-Pr]IrCl_2(H_2O) (7).

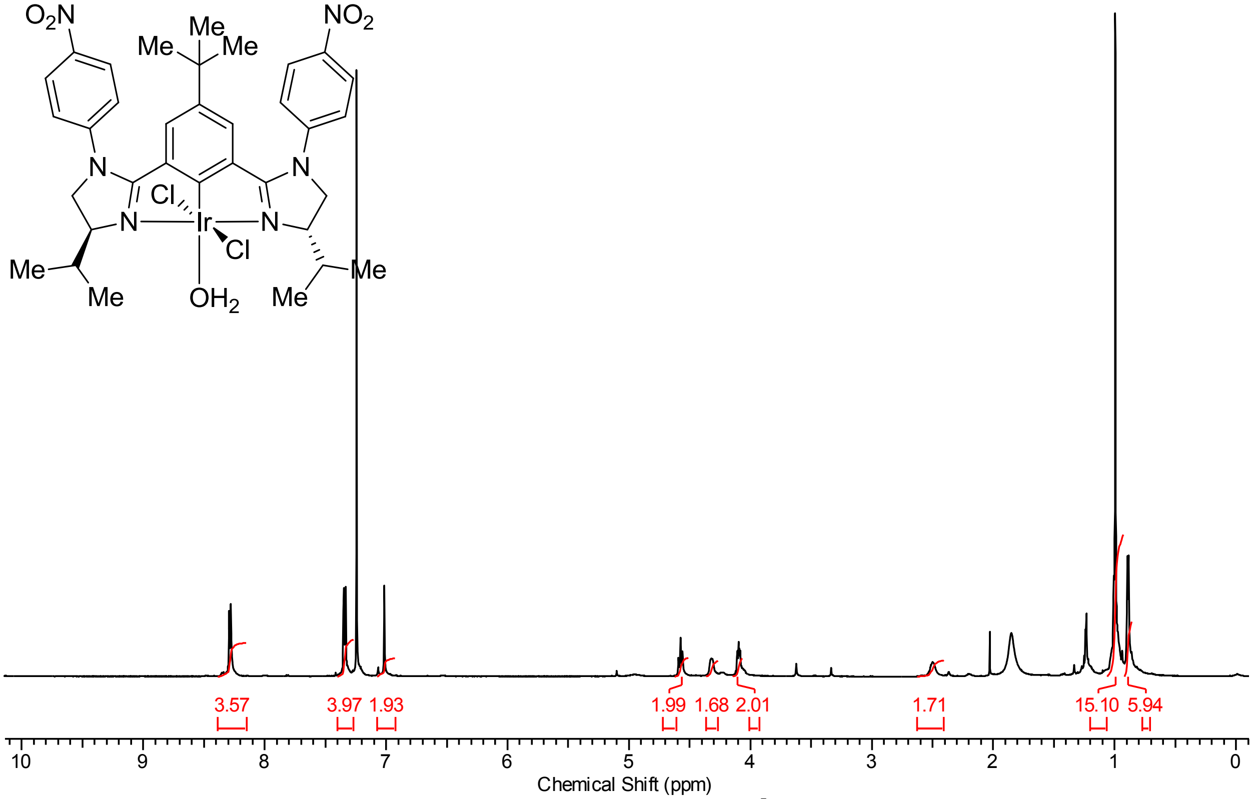

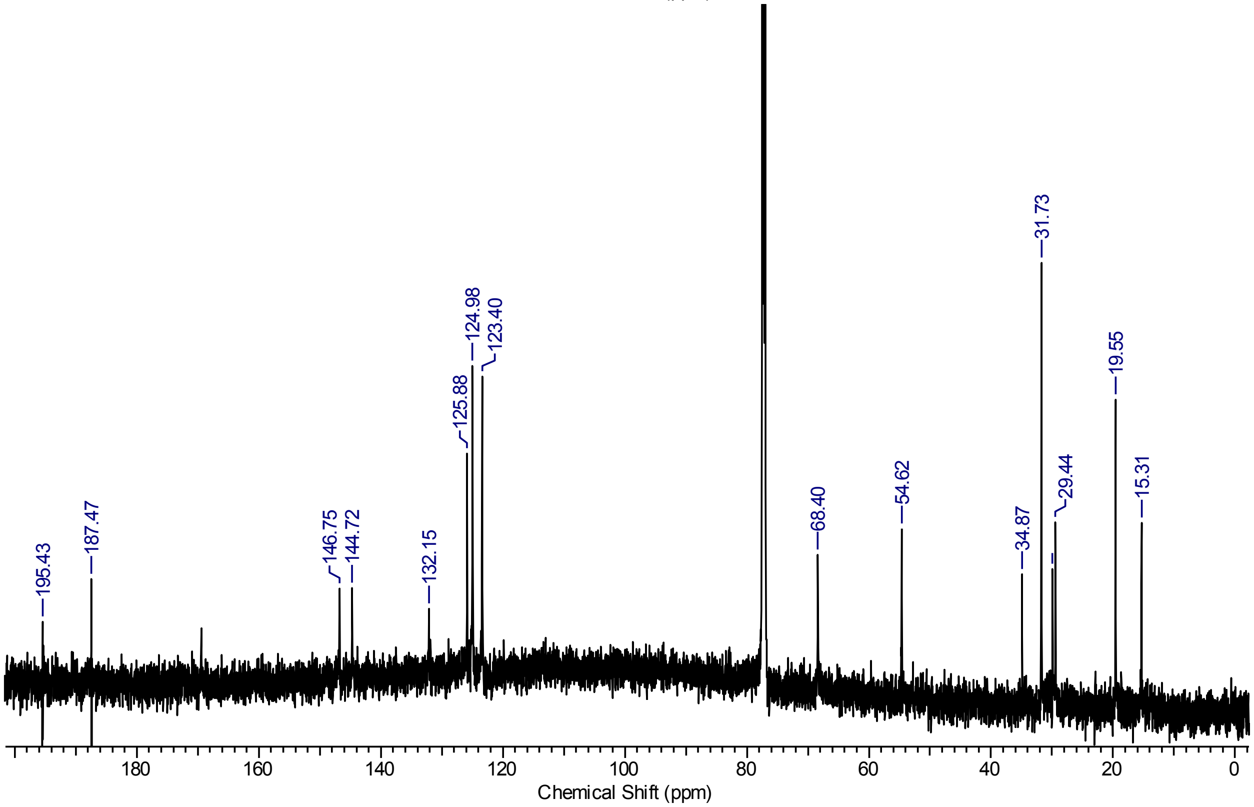

[(*R*, *R*)-*t*-ButylPhebim-4-CF_3-Ph-*i*-Pr]$IrCl_2(H_2O)$ (8).

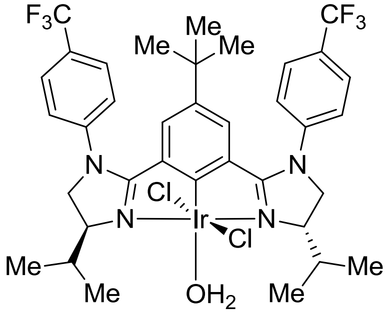

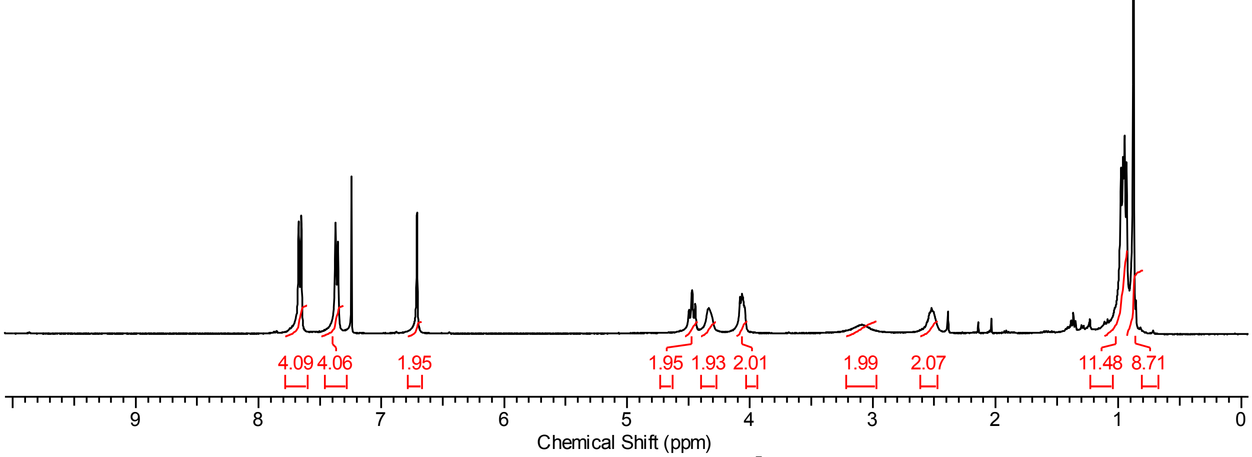

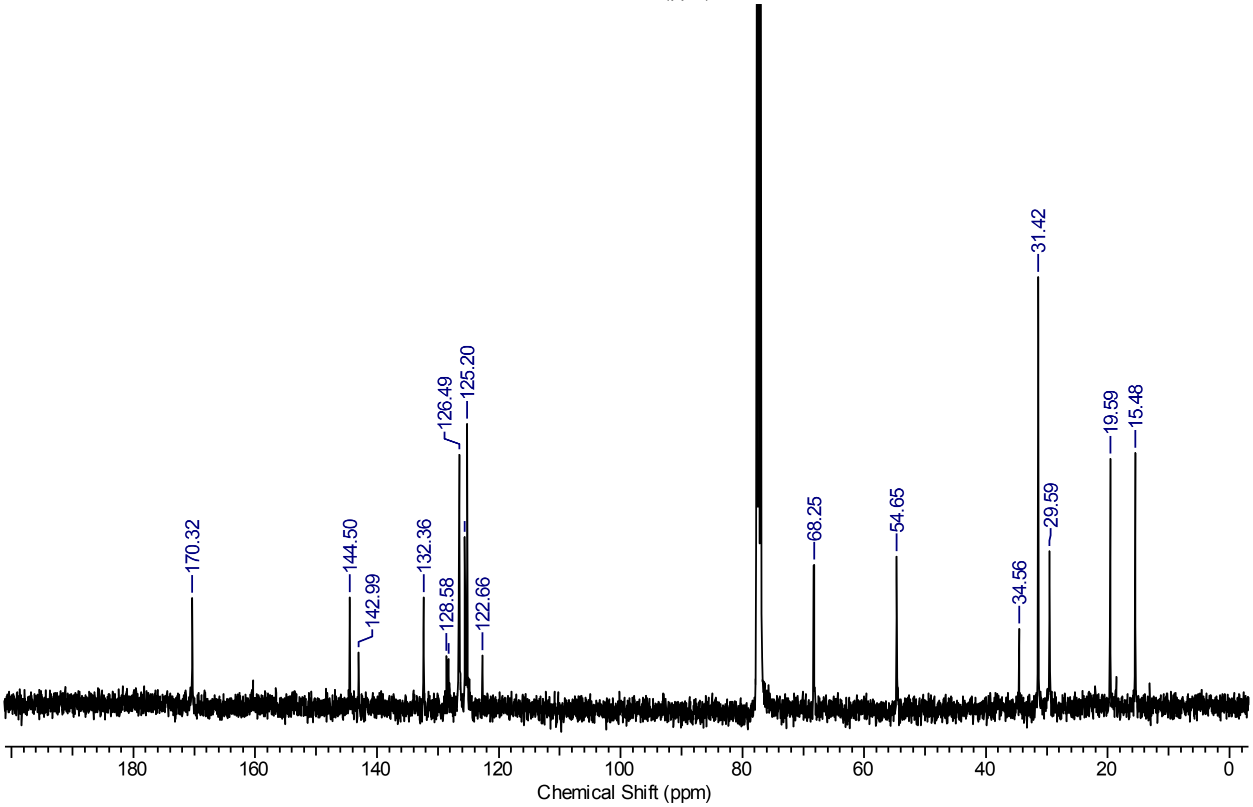

[(*R*, *R*)-*t*-ButylPhebim-4-CF$_3$-Ph-*i*-Pr]IrBr$_2$(H$_2$O) (9).

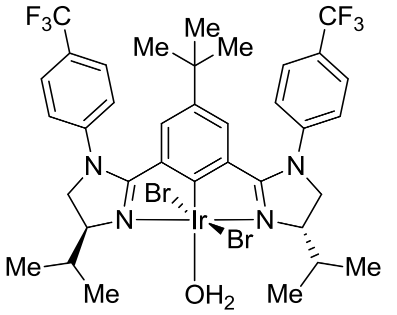

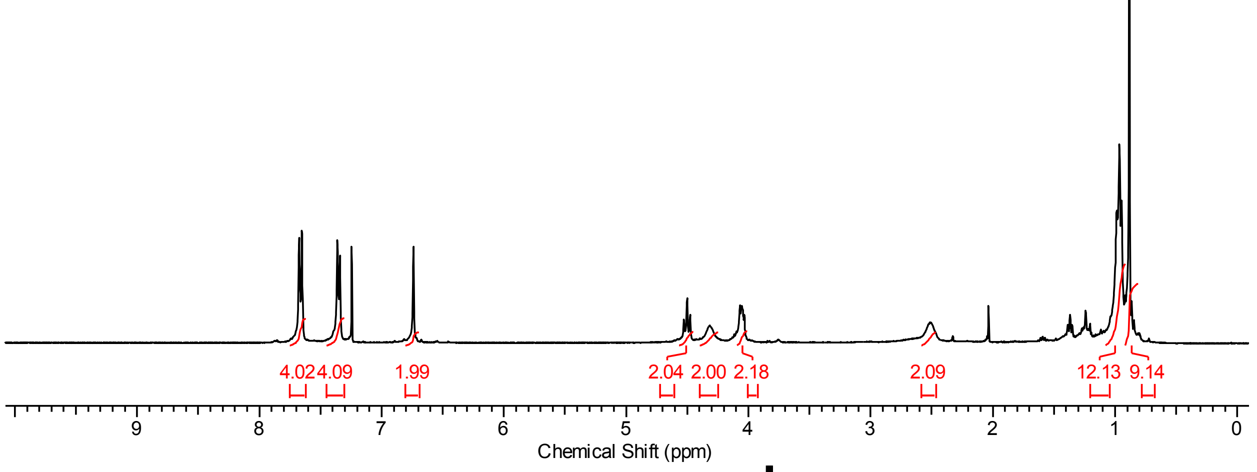

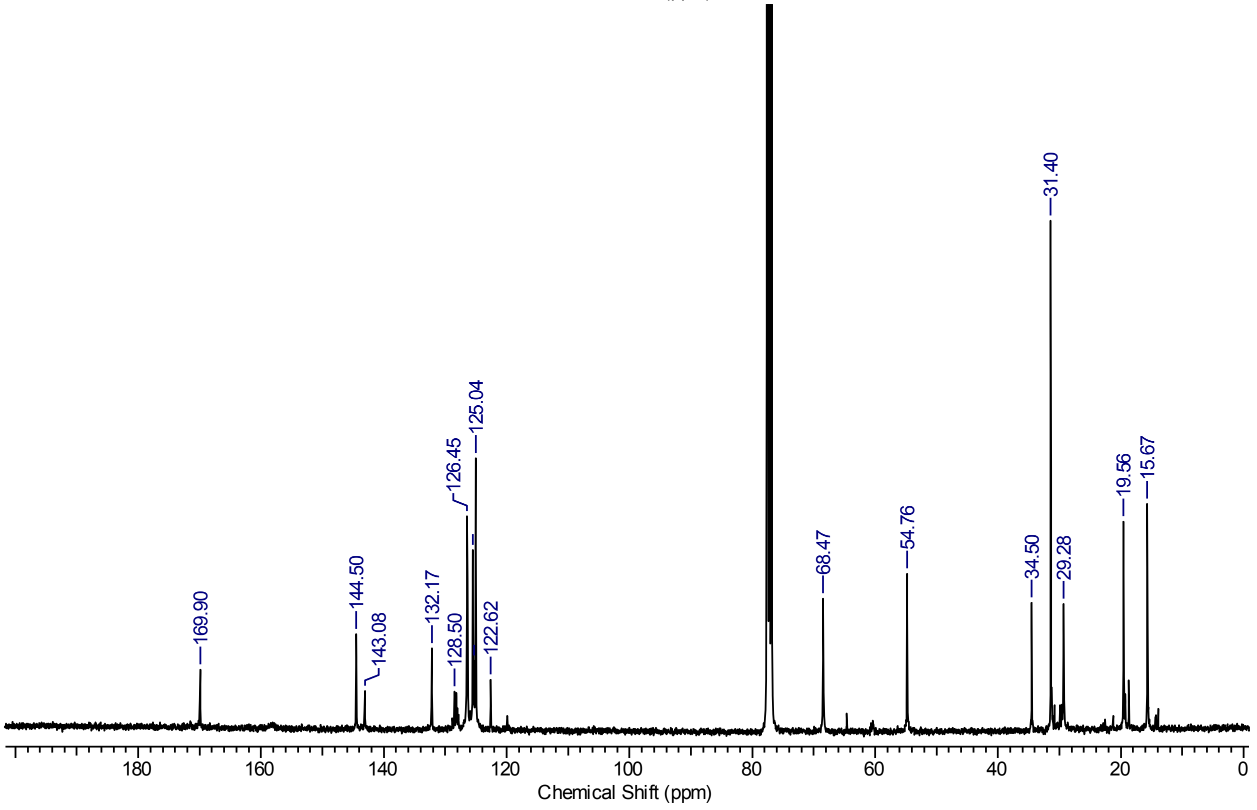

[(*R*, *R*)-*t*-ButylPhebim-4-CF_3-Ph-*i*-Bu]$IrCl_2(H_2O)$ (10).

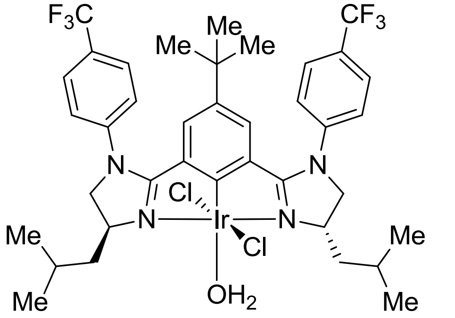

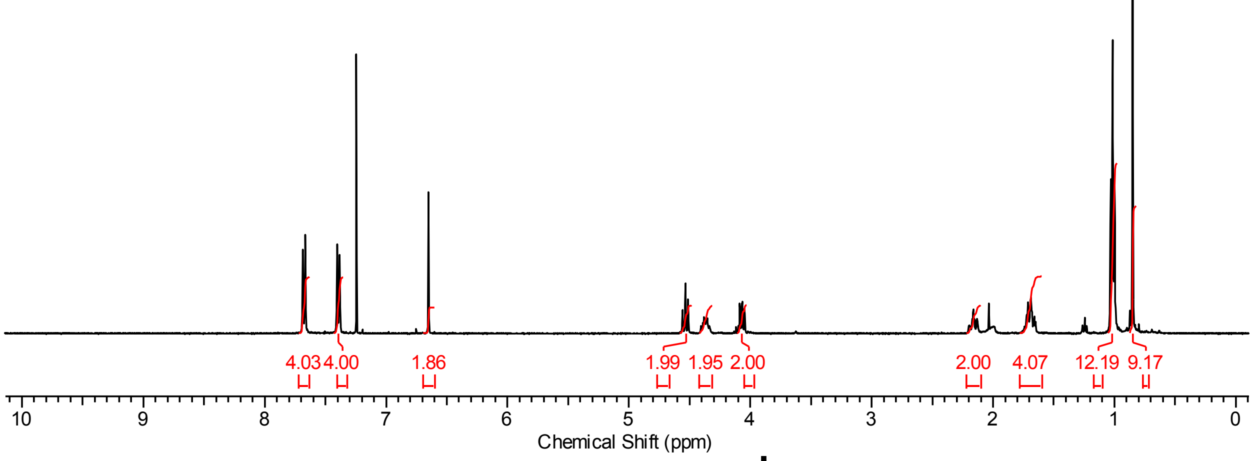

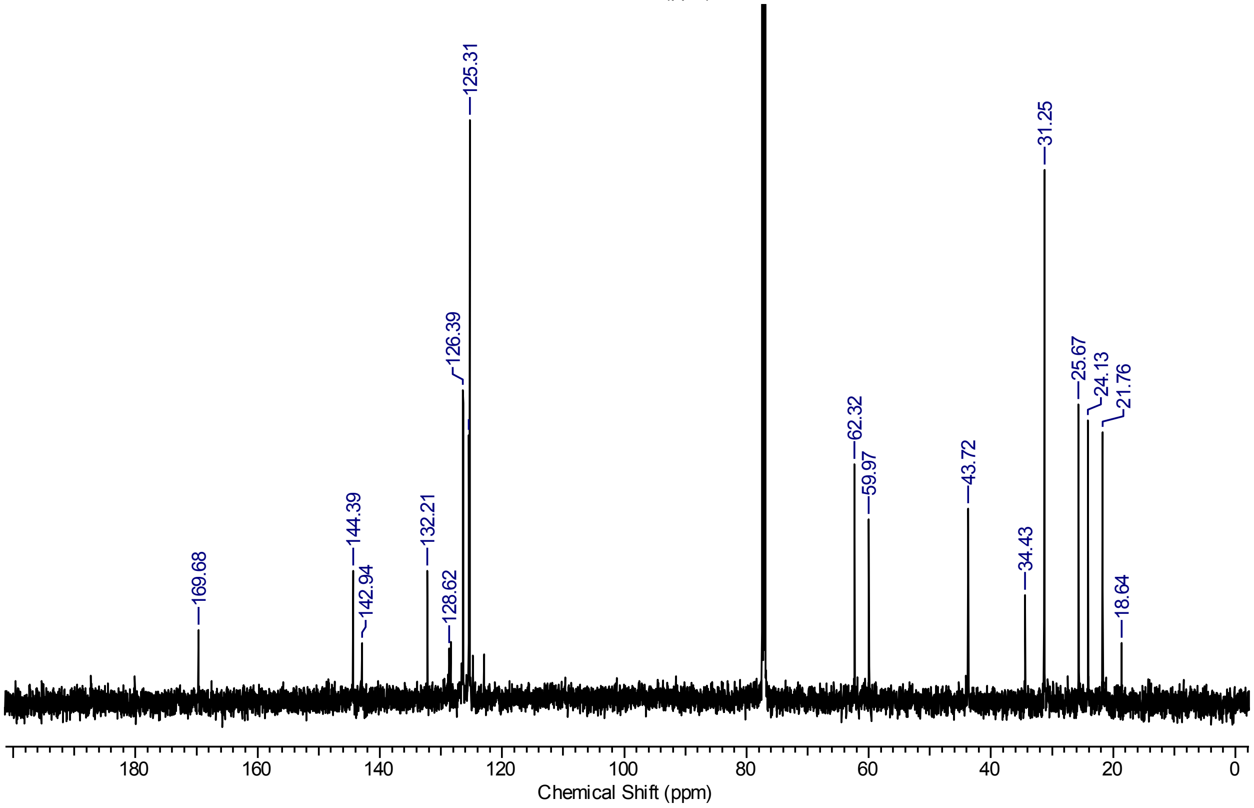

[(*R*, *R*)-*t*-ButylPhebim-4-CF_3Ph-*n*-Bu]IrCl_2(H_2O) (11).

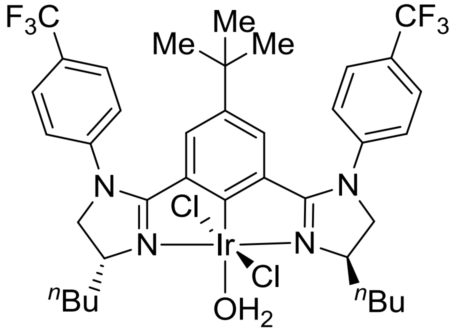

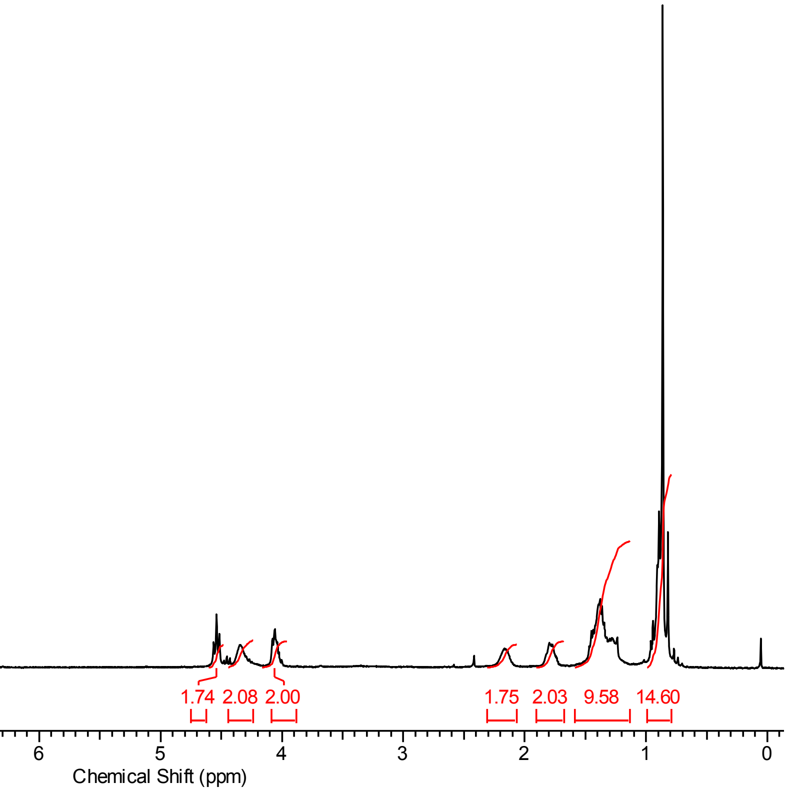

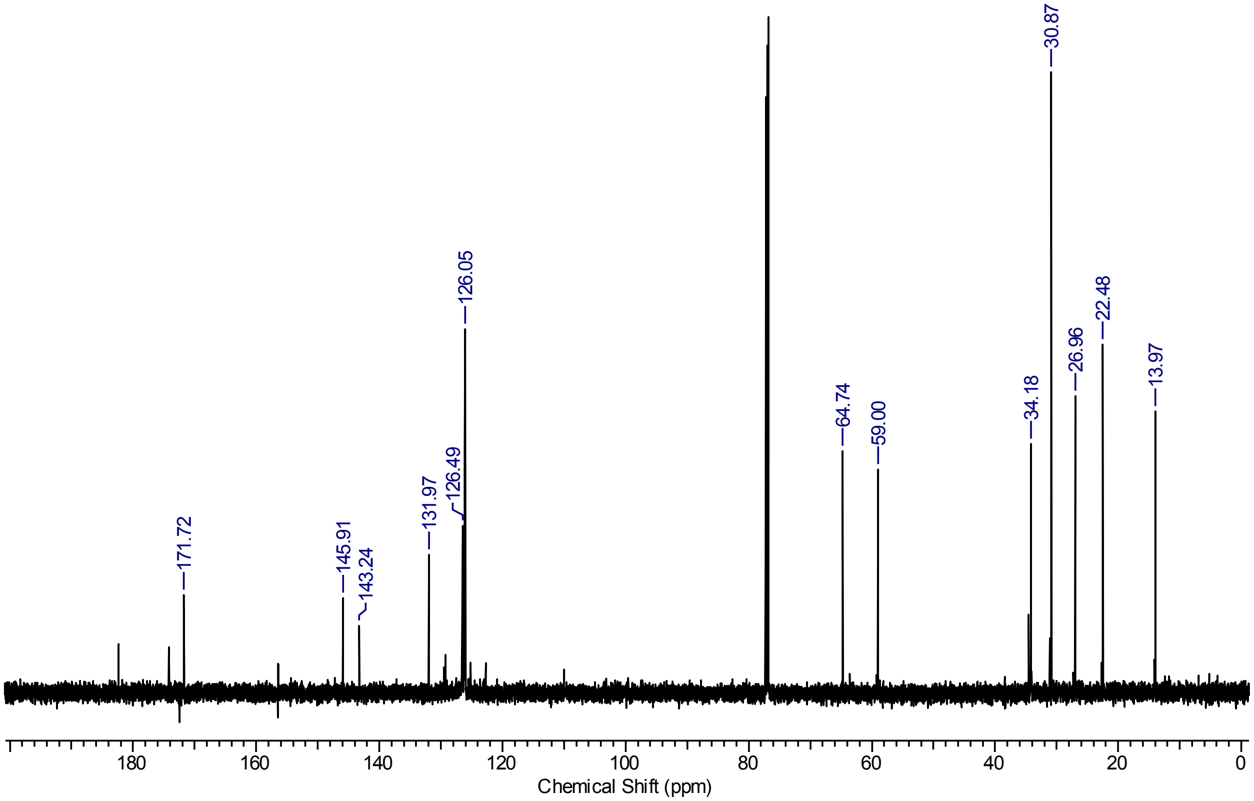

[(*R*, *R*)-*t*-ButylPhebim-4-CF$_3$-Ph-*s*-Bu]IrCl$_2$(H$_2$O) (12).

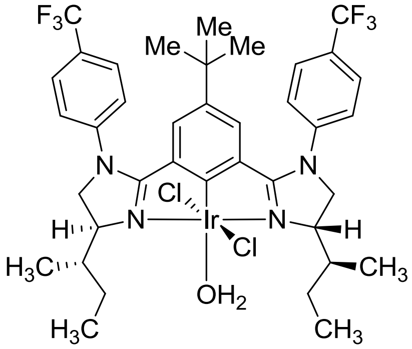

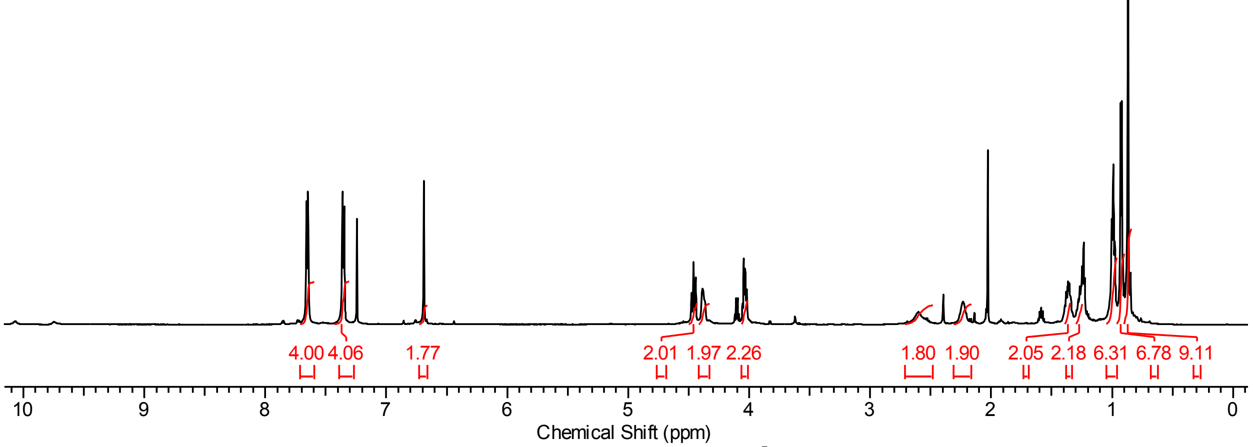

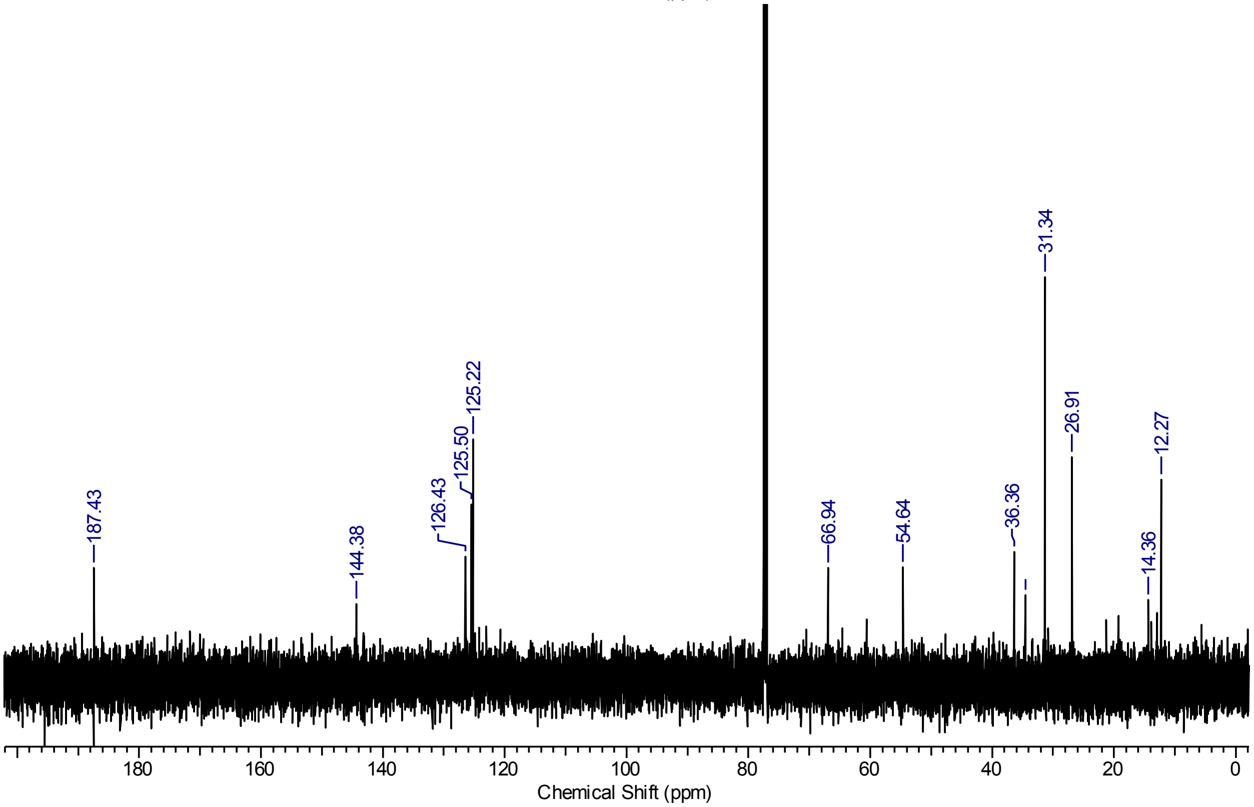

[(*R*, *R*)-*t*-ButylPhebim-4-CF_3Ph-^{c}Hex]$IrCl_2(H_2O)$ (13).

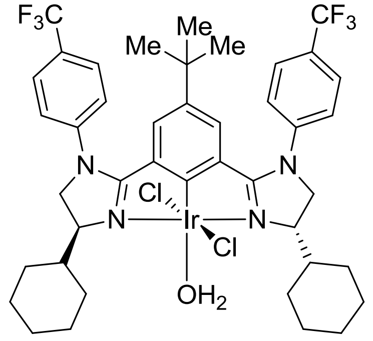

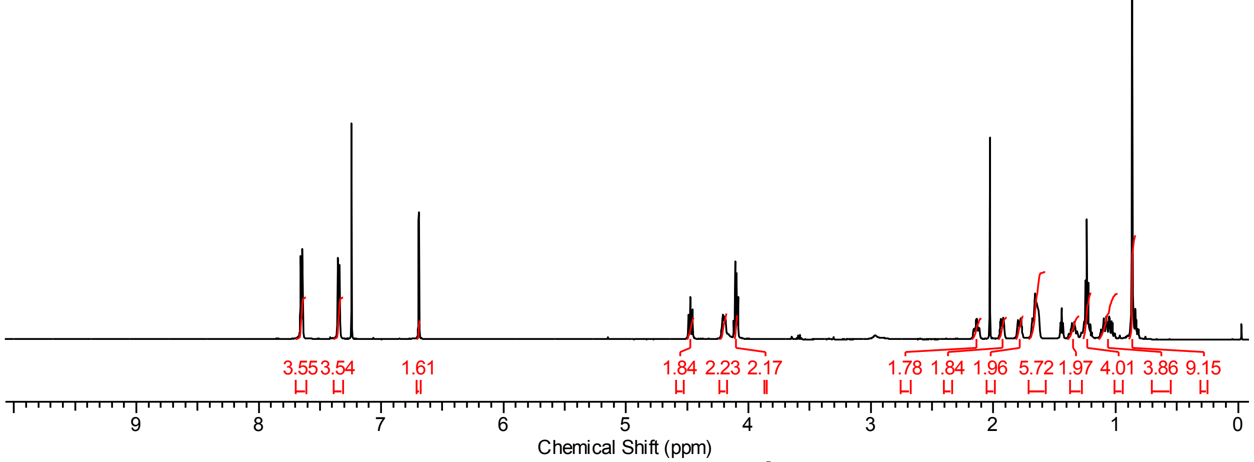

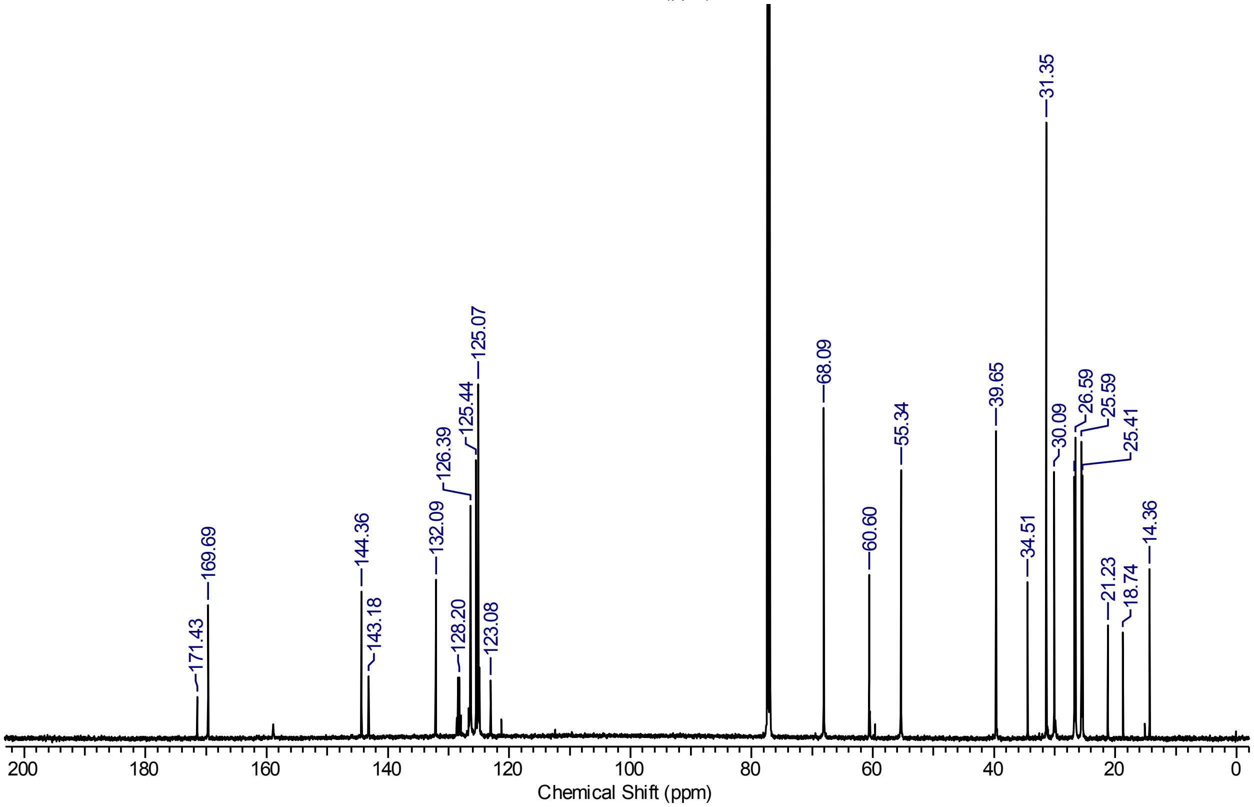

[(*R*, *R*)-*t*-ButylPhebim-4-CF$_3$Ph-CH$_2$^{c}Hex]IrCl$_2$(H$_2$O) (14).

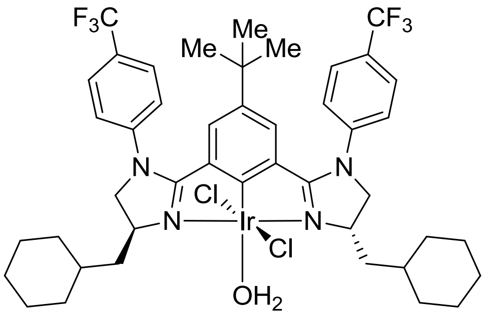

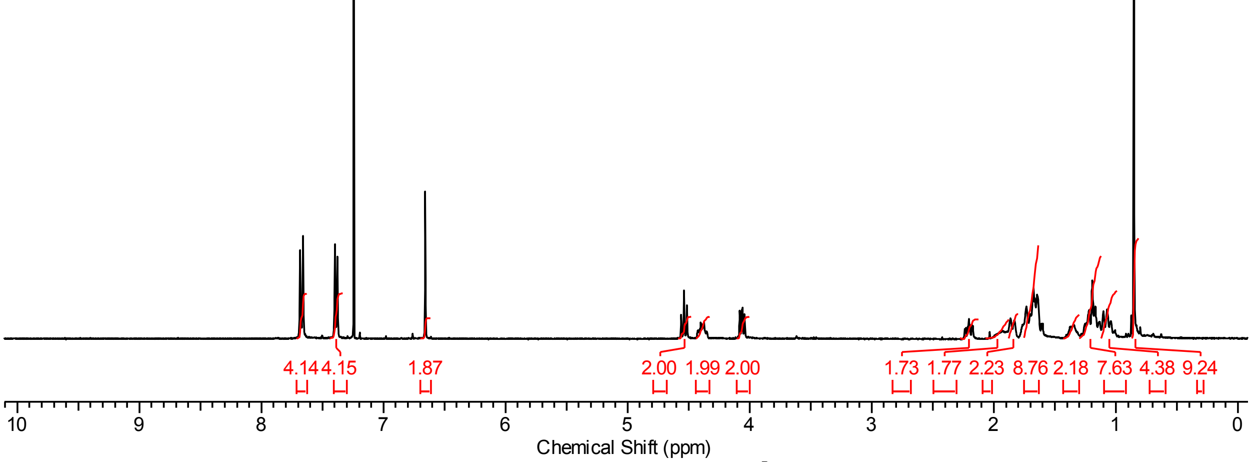

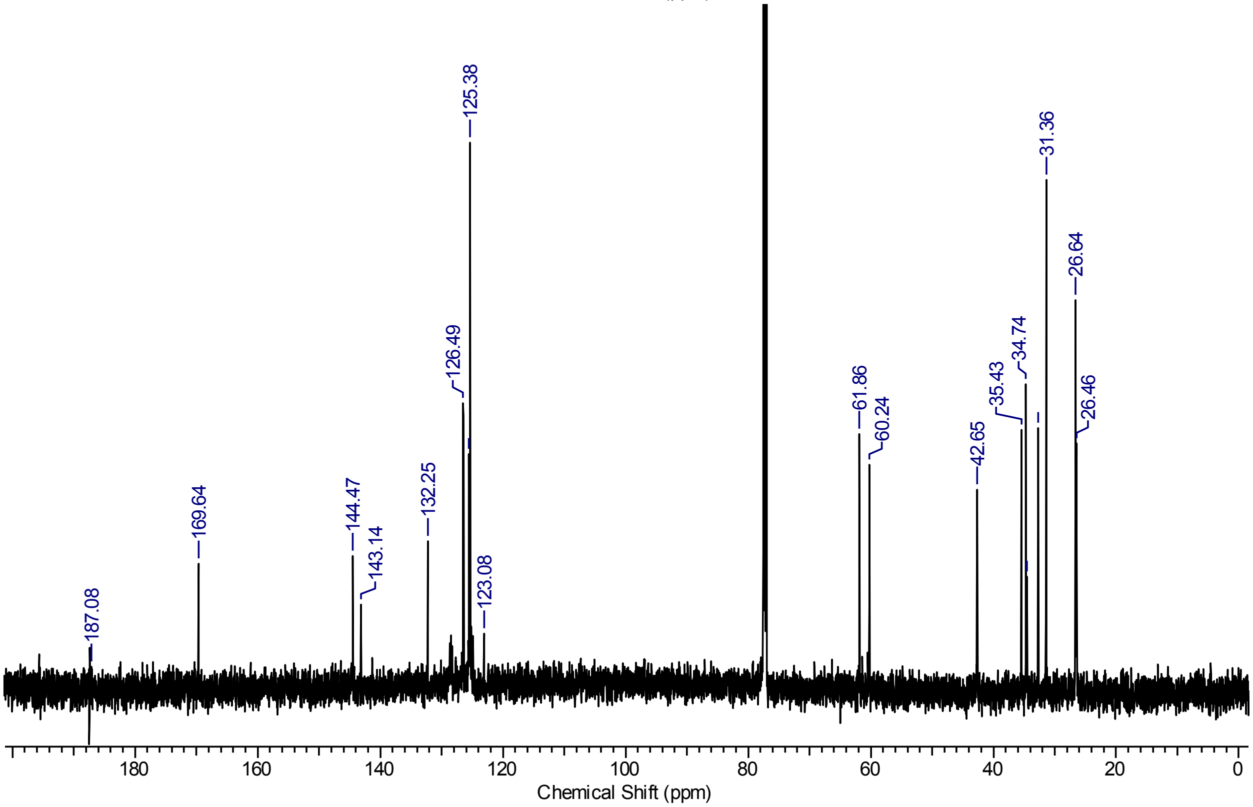

[(*S*, *S*)-*t*-ButylPhebim-p-CF_3Ph-Bn]$IrCl_2(H_2O)$ (15).

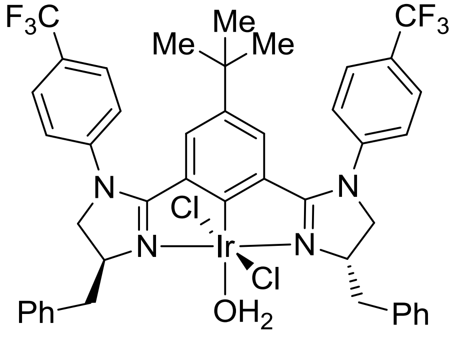

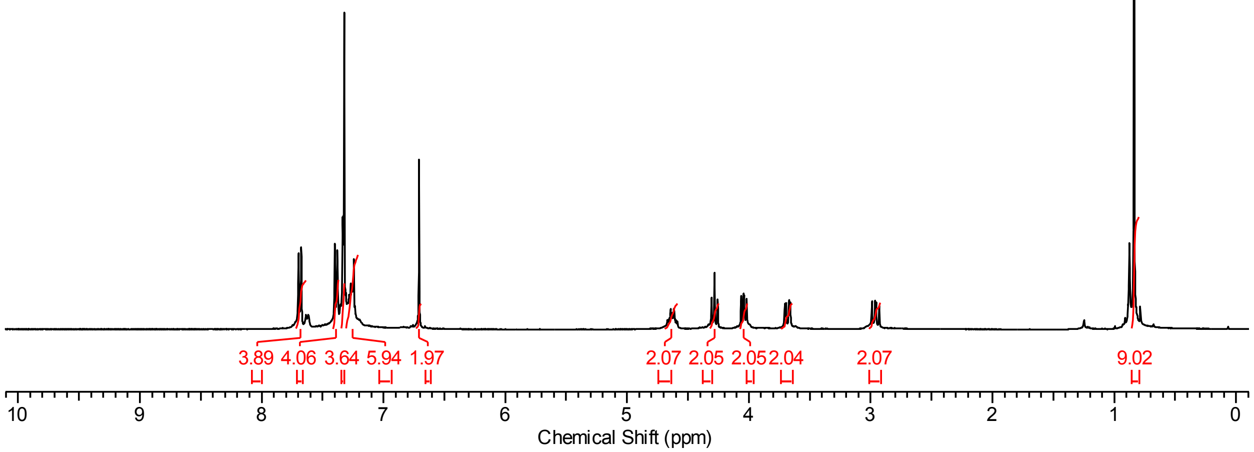

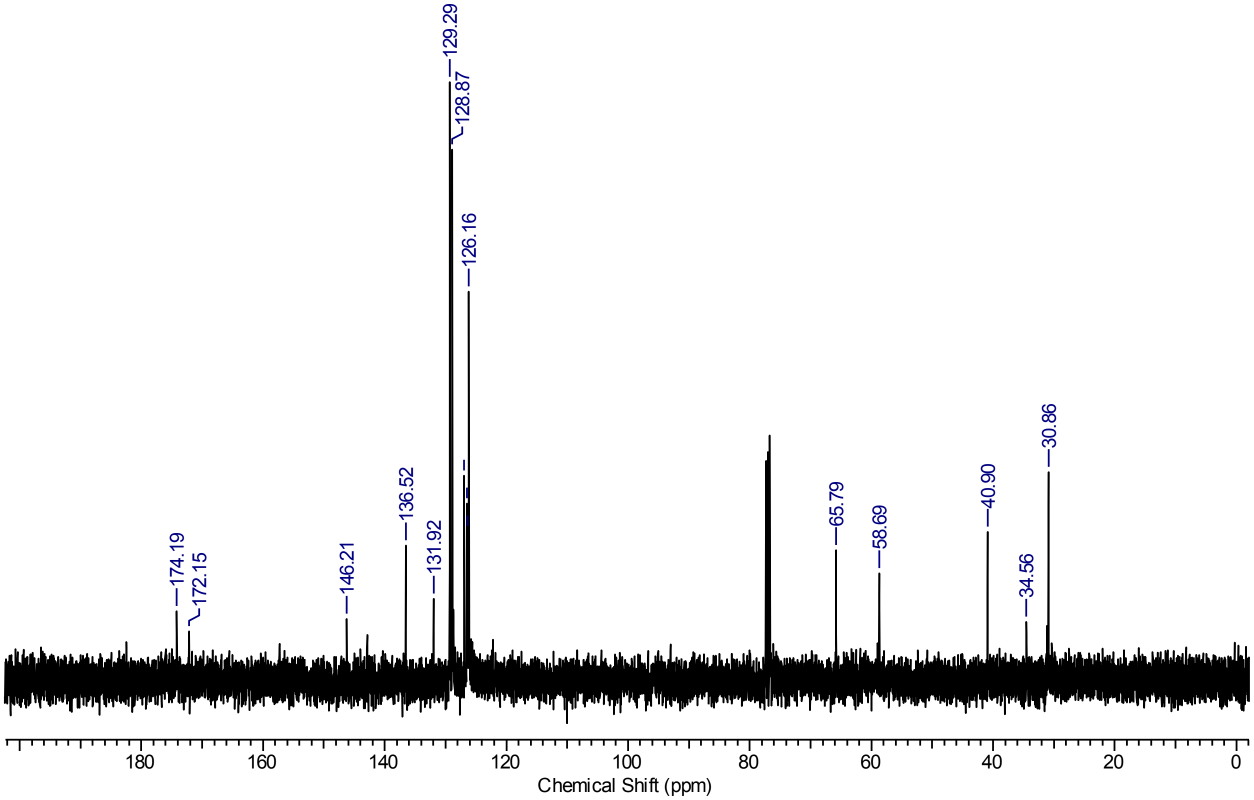

[(*S*, *S*)-*t*-ButylPhebim-p-CF$_3$Ph-CH$_2$^{t}Bu]IrCl$_2$(H$_2$O) (16).

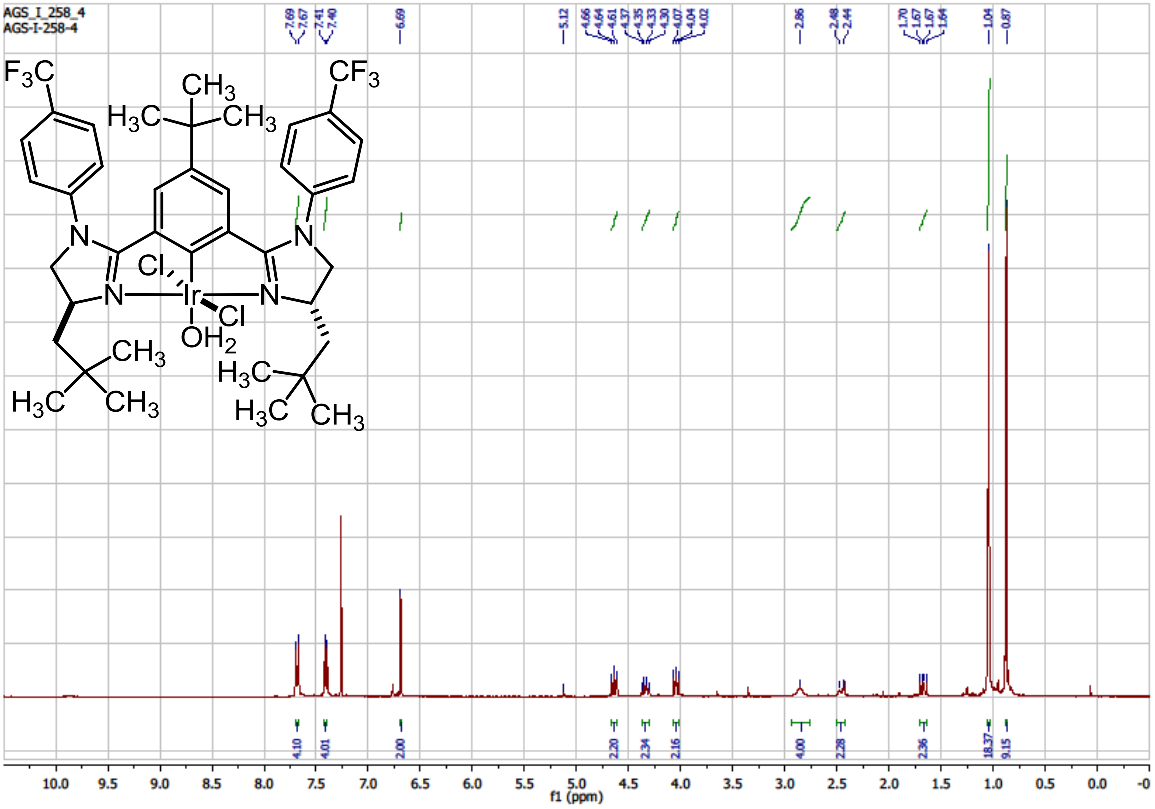

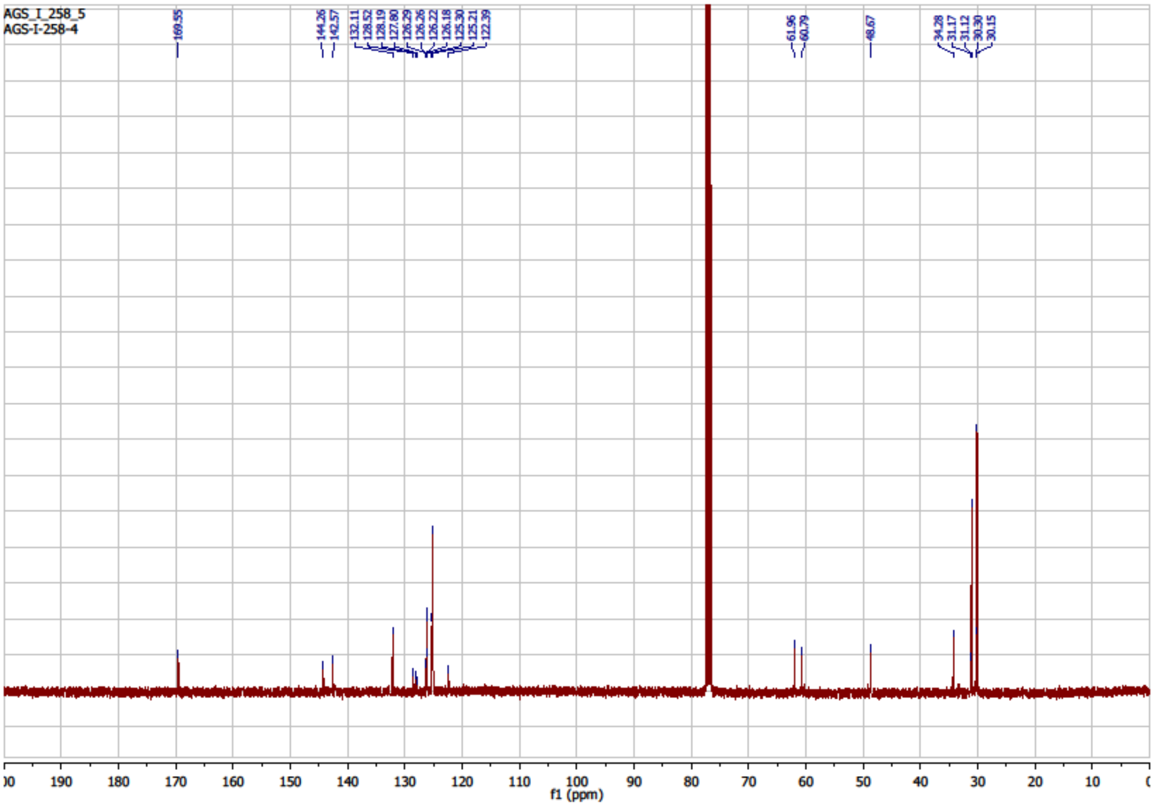

2,2,2-trichloroethyl 2-diazoacetate (S25).

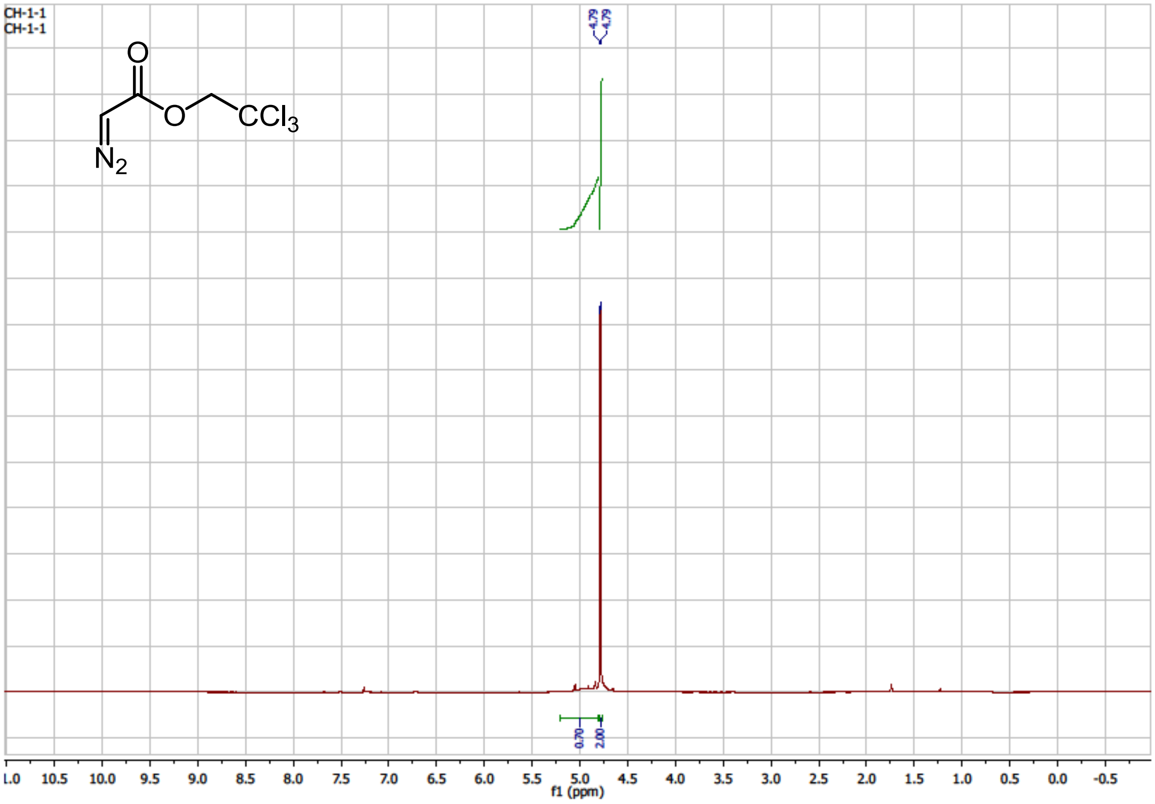

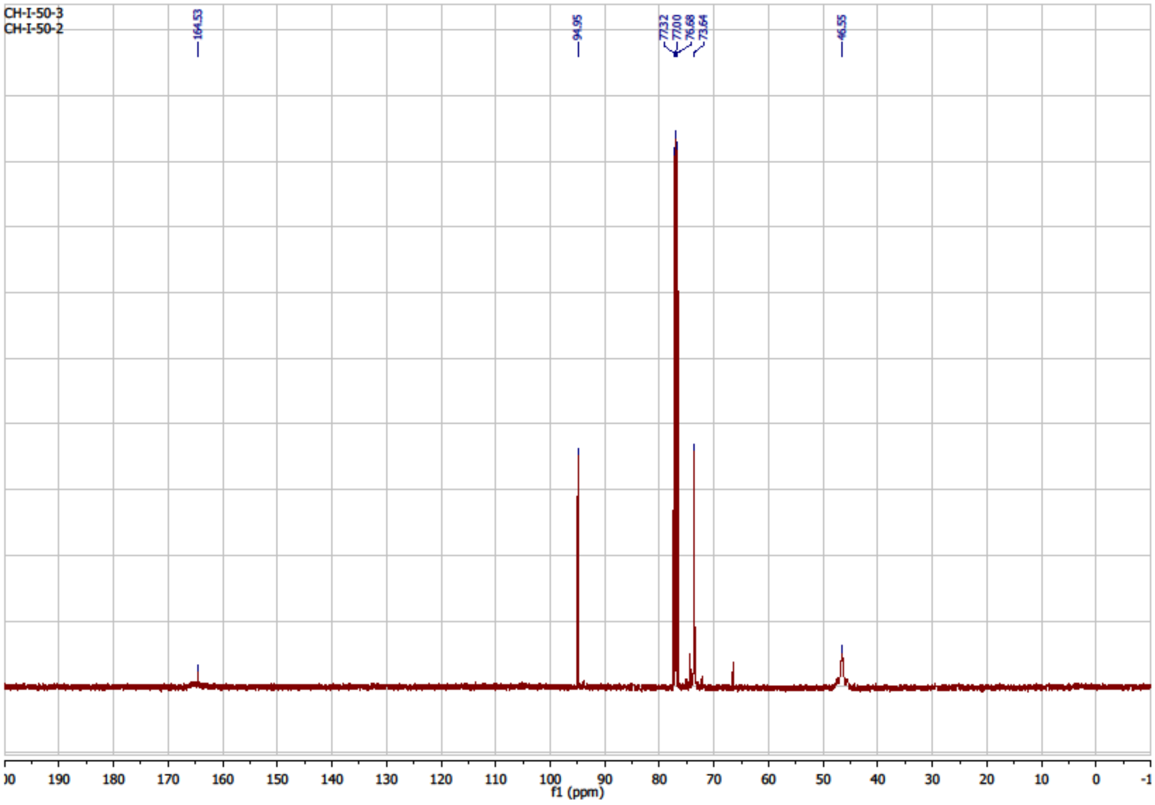

2-(trimethylsilyl)ethyl 2-diazoacetate (S27).

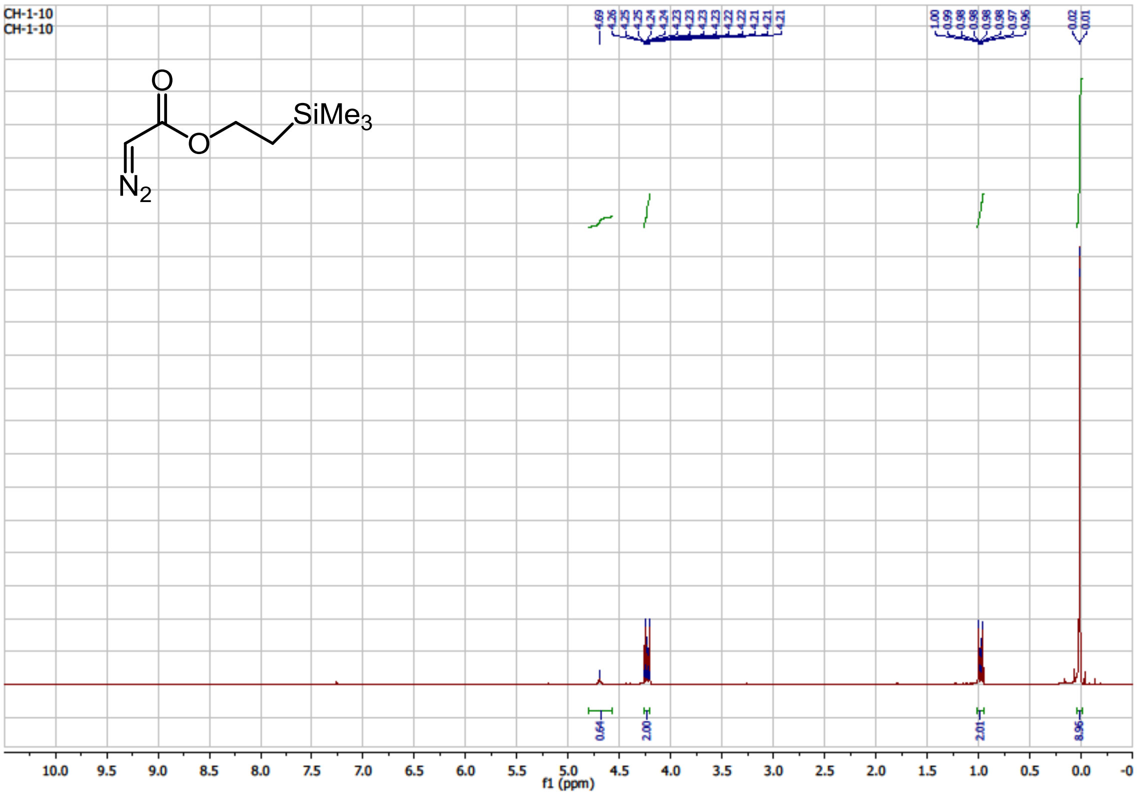

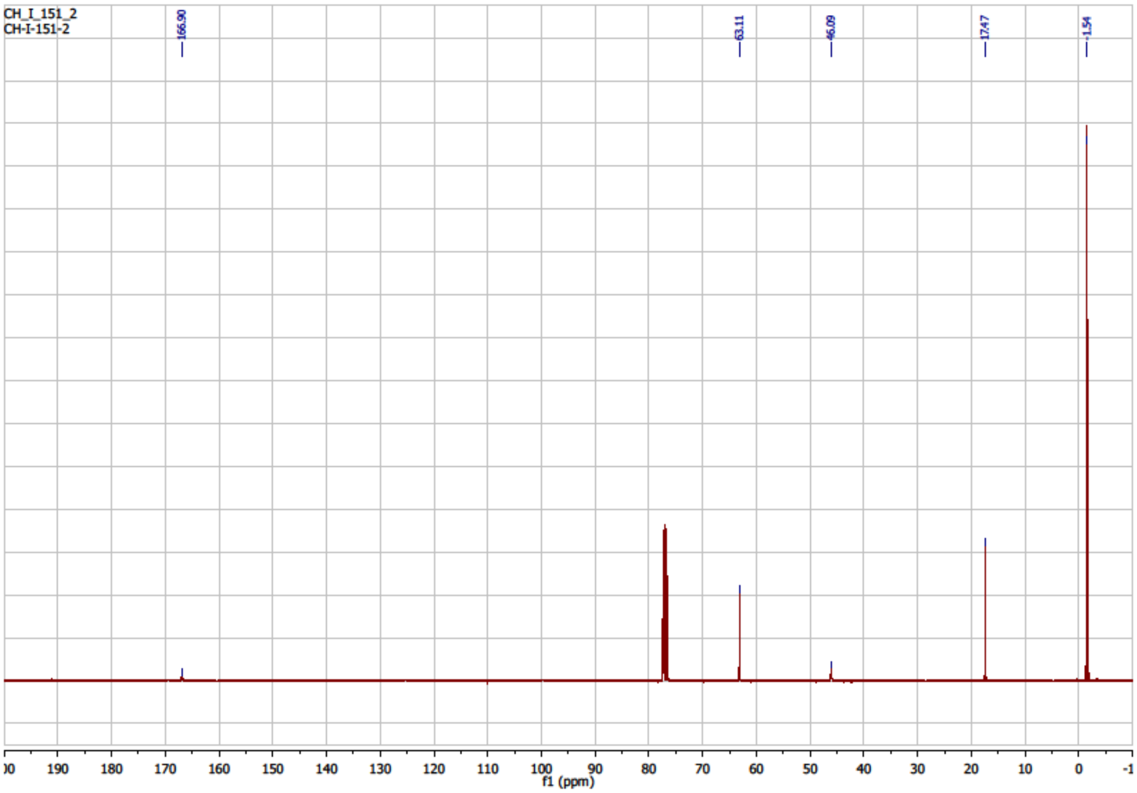

4-(trifluoromethyl)phenyl 2-bromoacetate (S28).

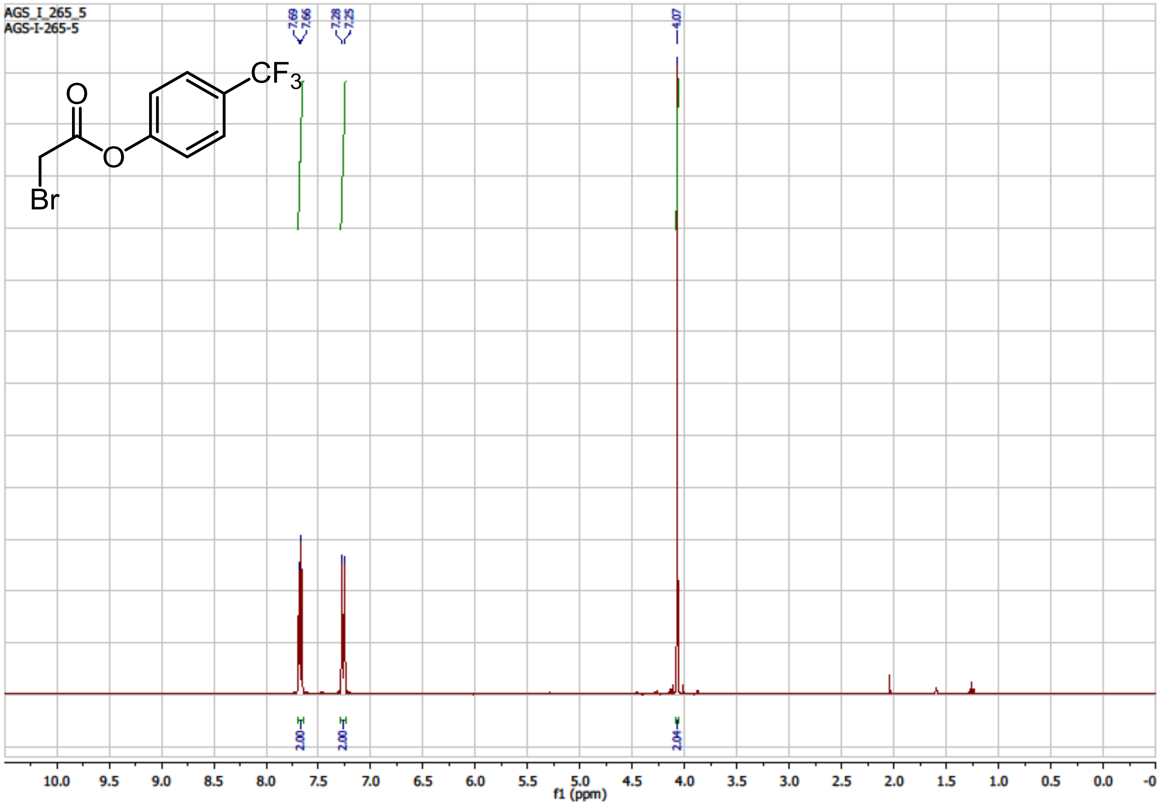

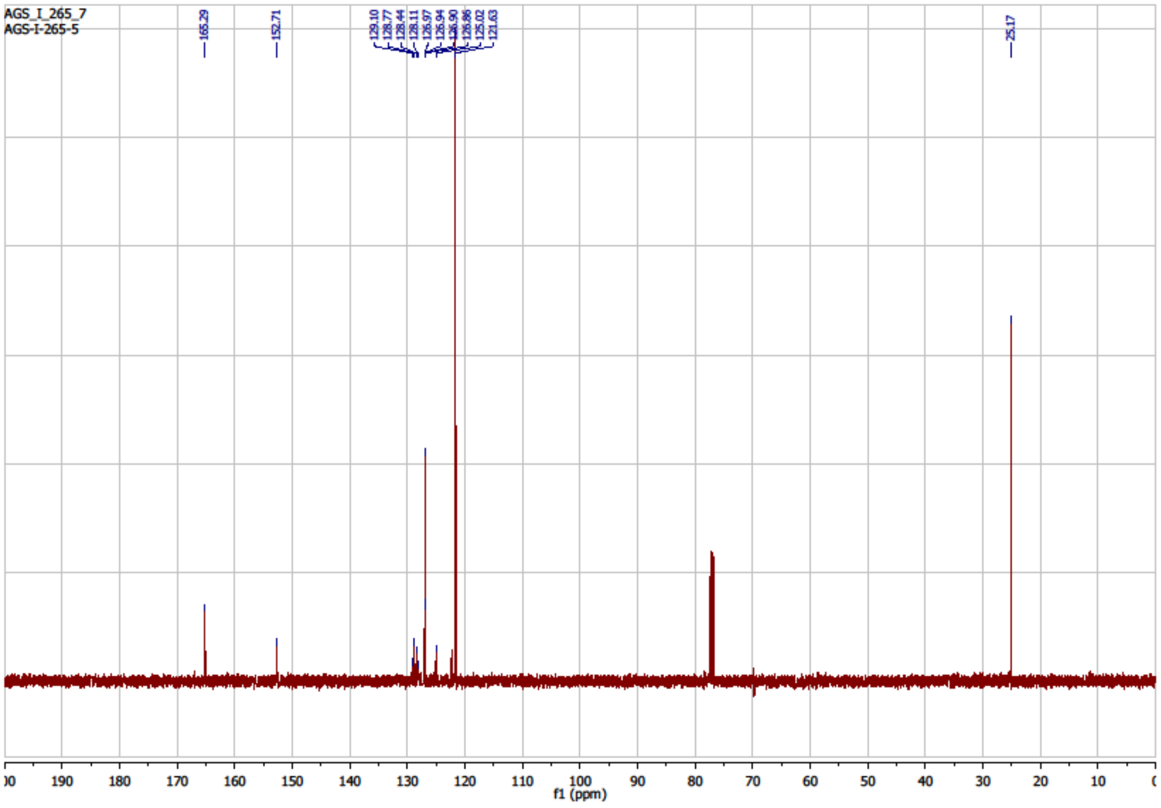

4-(trifluoromethyl)phenyl 2-diazoacetate (S29).

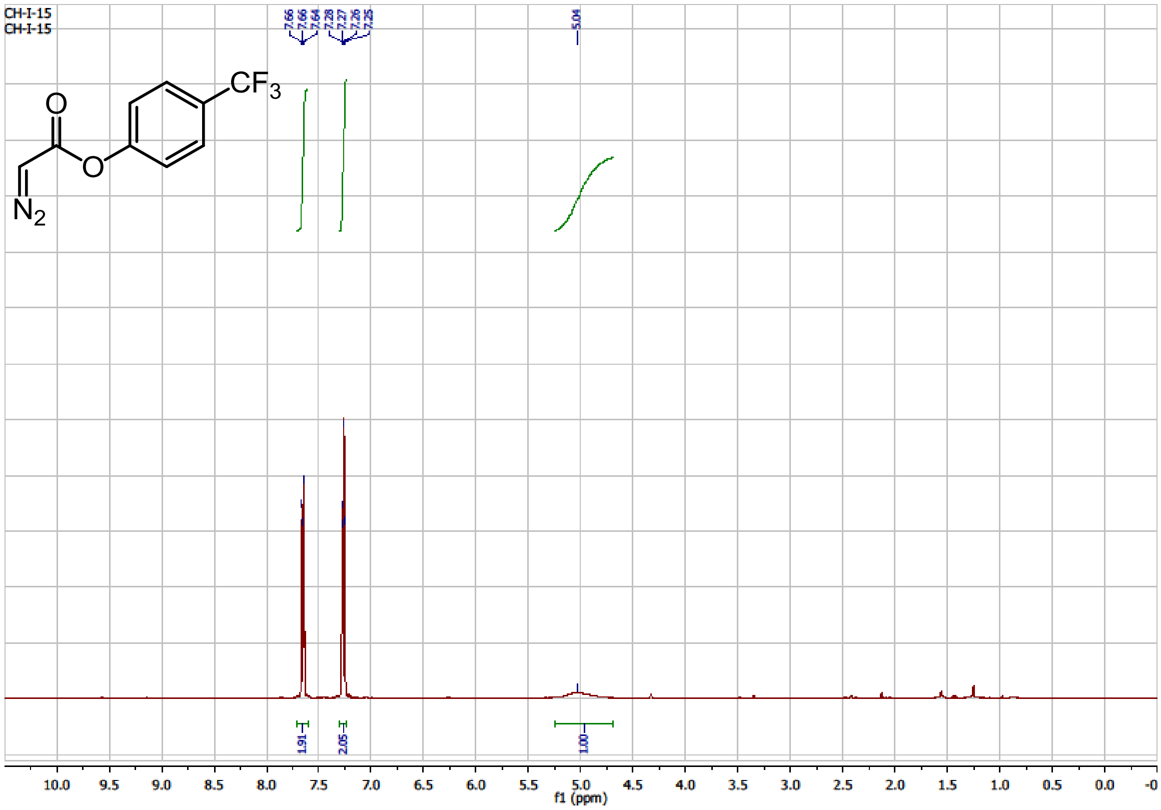

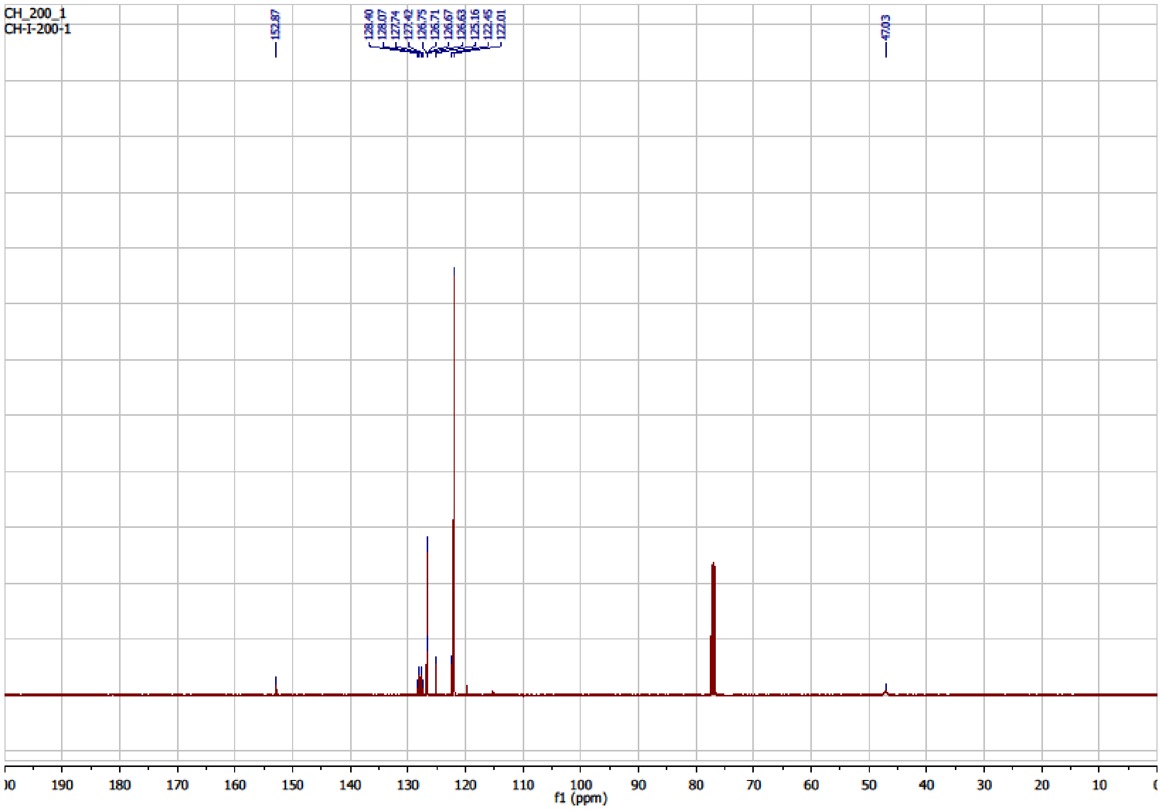

2-(1,3-dihydroisobenzofuran-4-yl)-4,4,5,5-tetramethyl-1,3,2-dioxaborolane (S30).

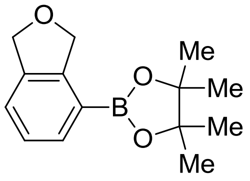

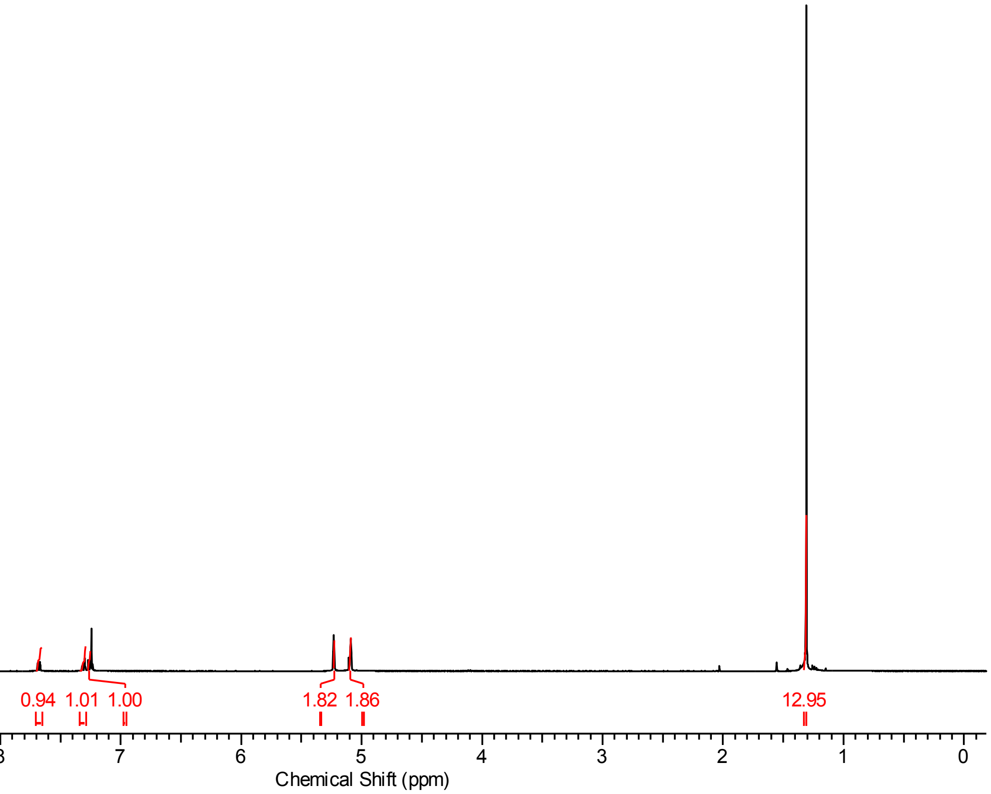

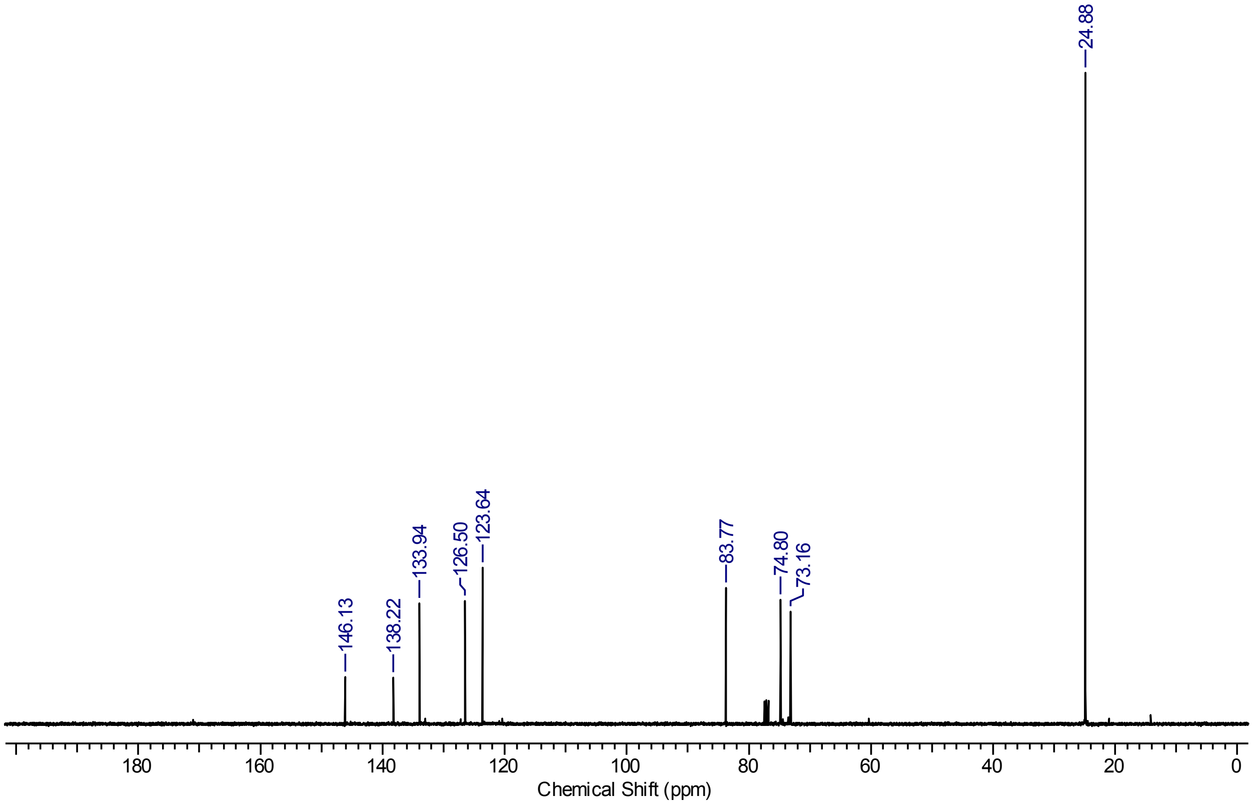

4-bromo-1,3-dihydroisobenzofuran (S33).

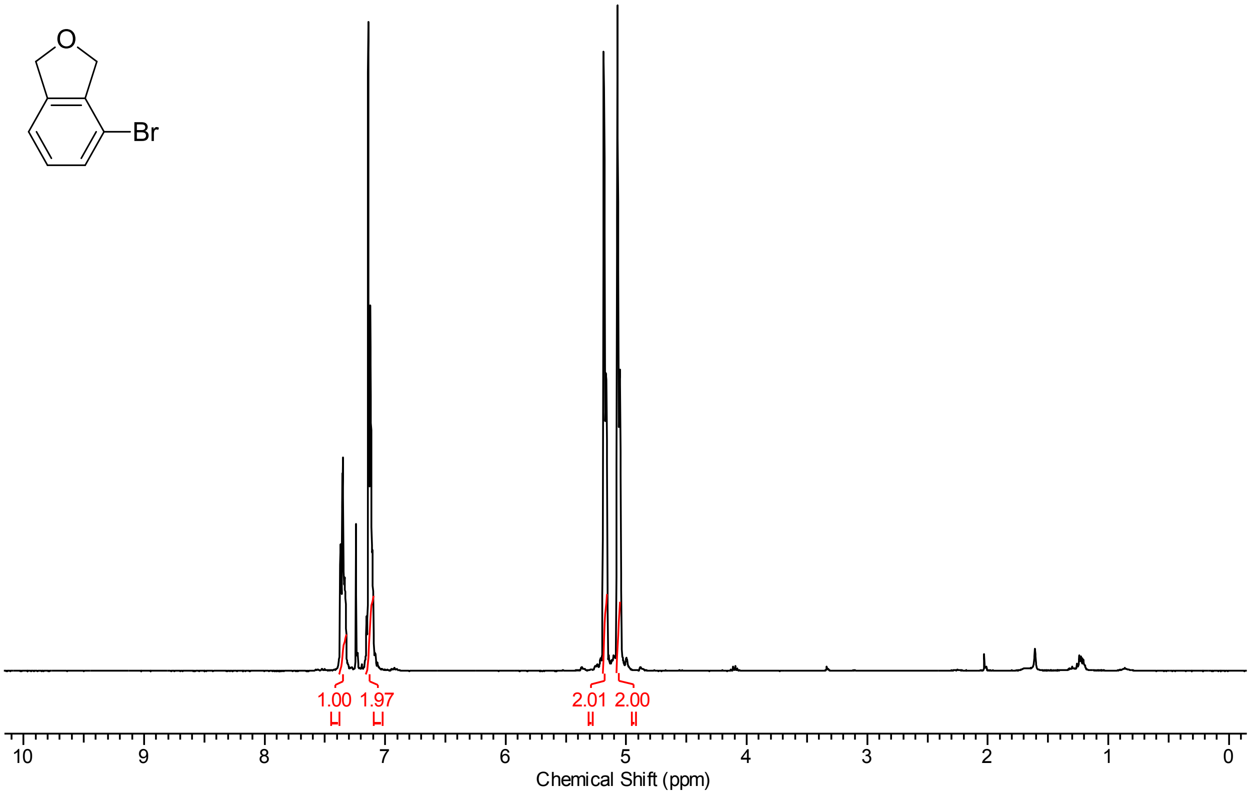

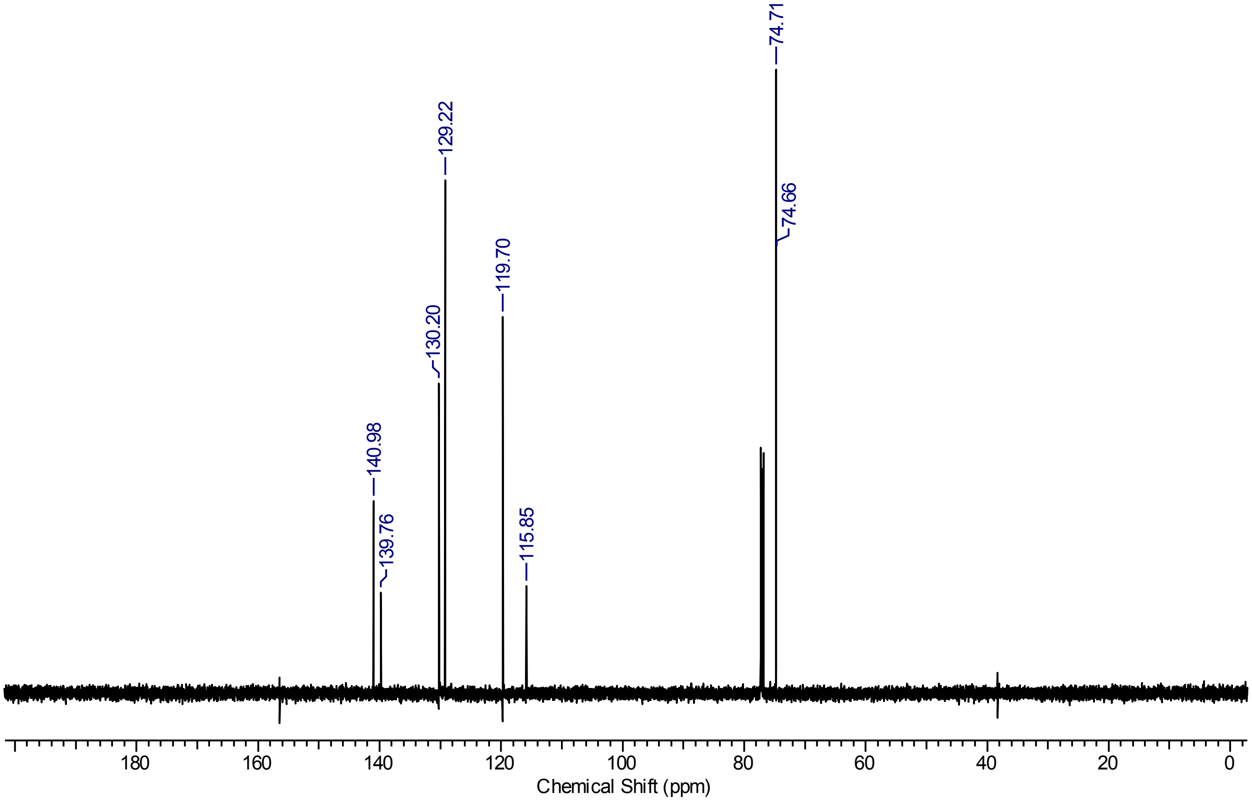

4-nitro-1,3-dihydroisobenzofuran (S35).

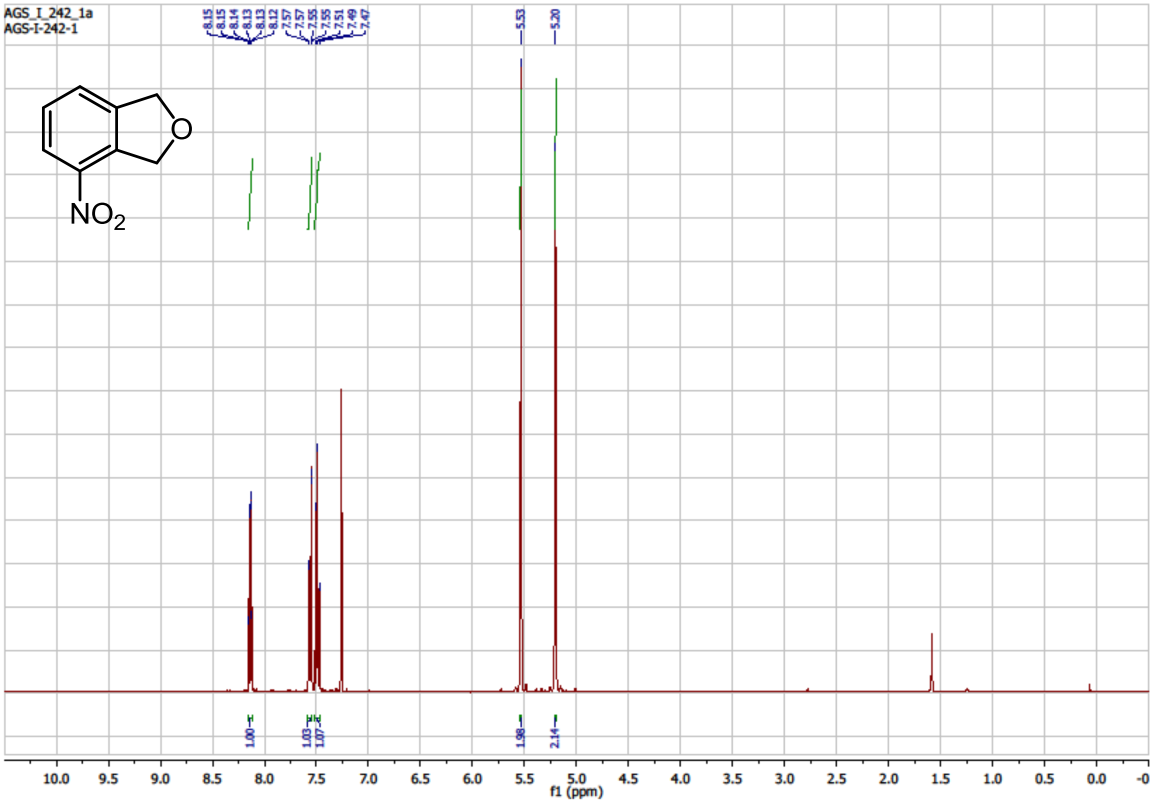

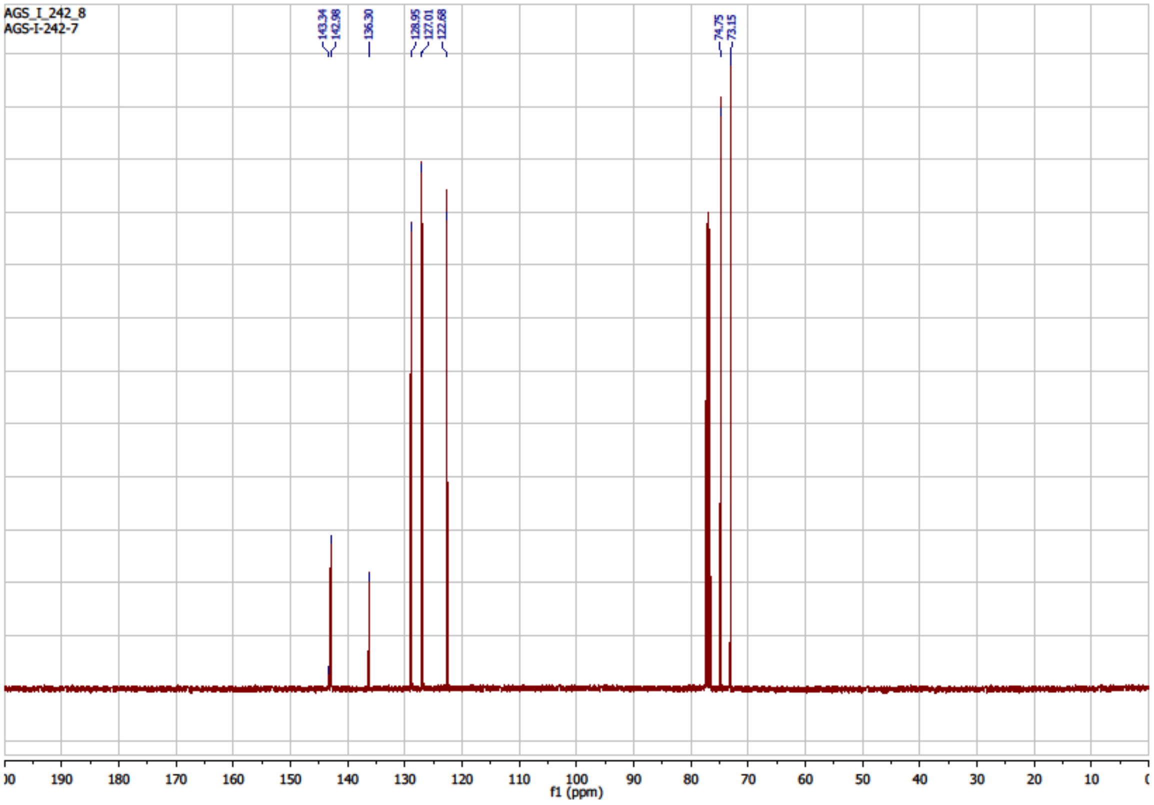

5-nitro-1,3-dihydroisobenzofuran (S36).

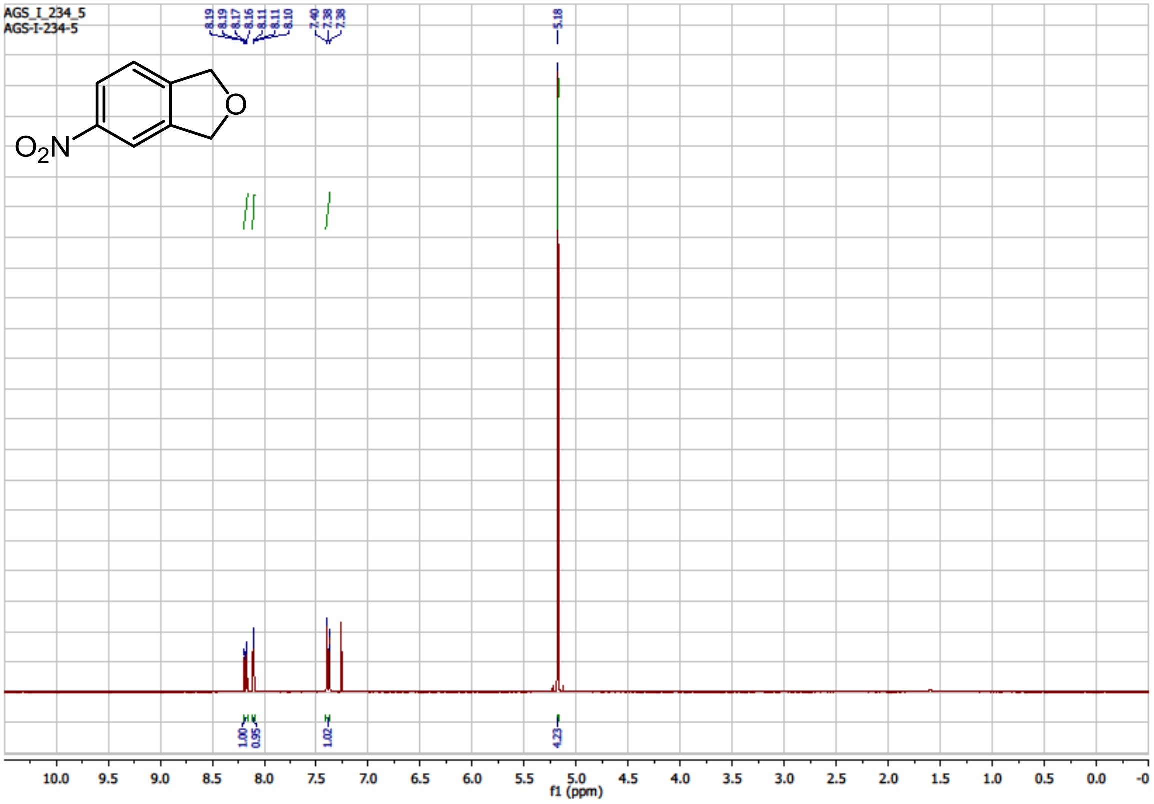

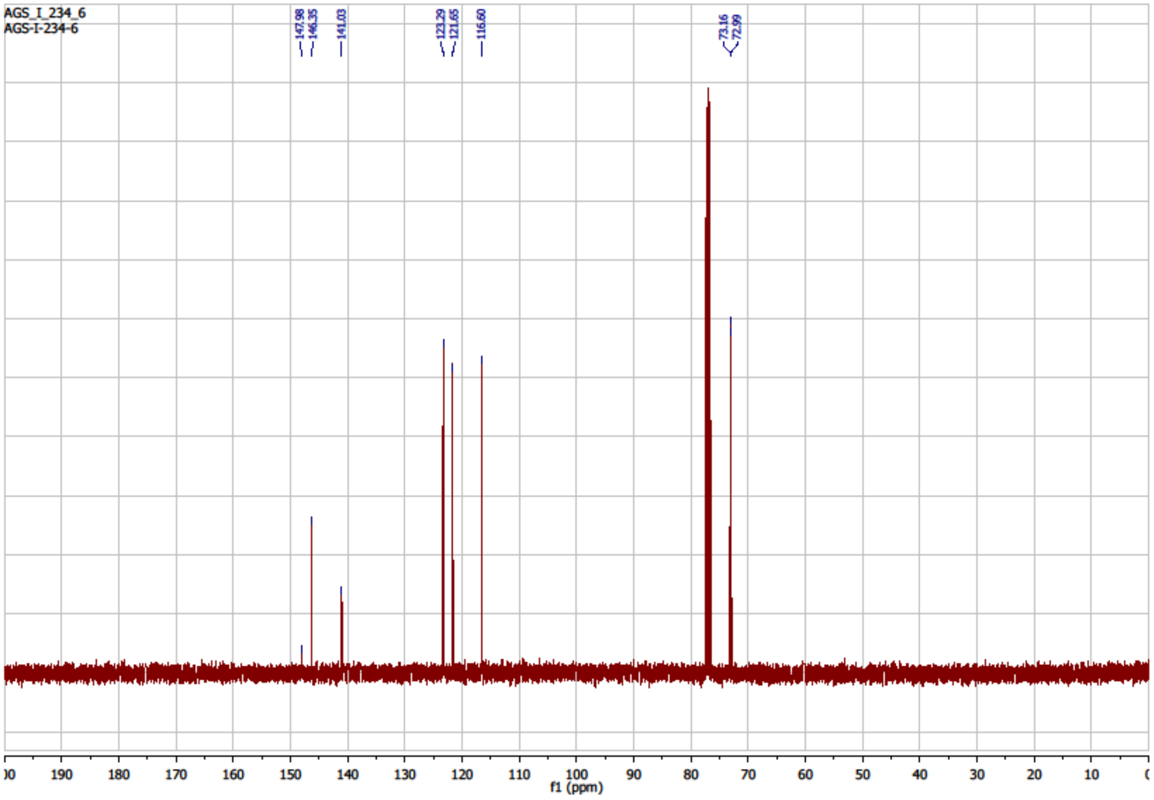

1,3-dihydroisobenzofuran-4-amine (S37).

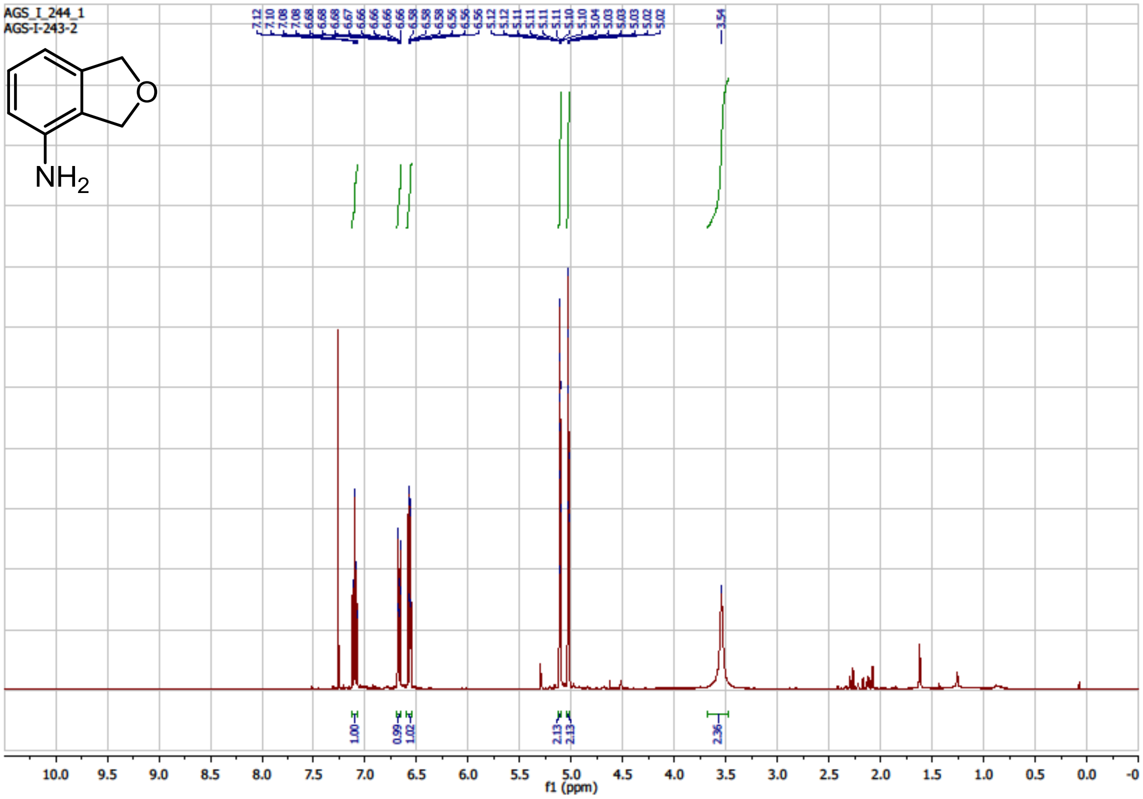

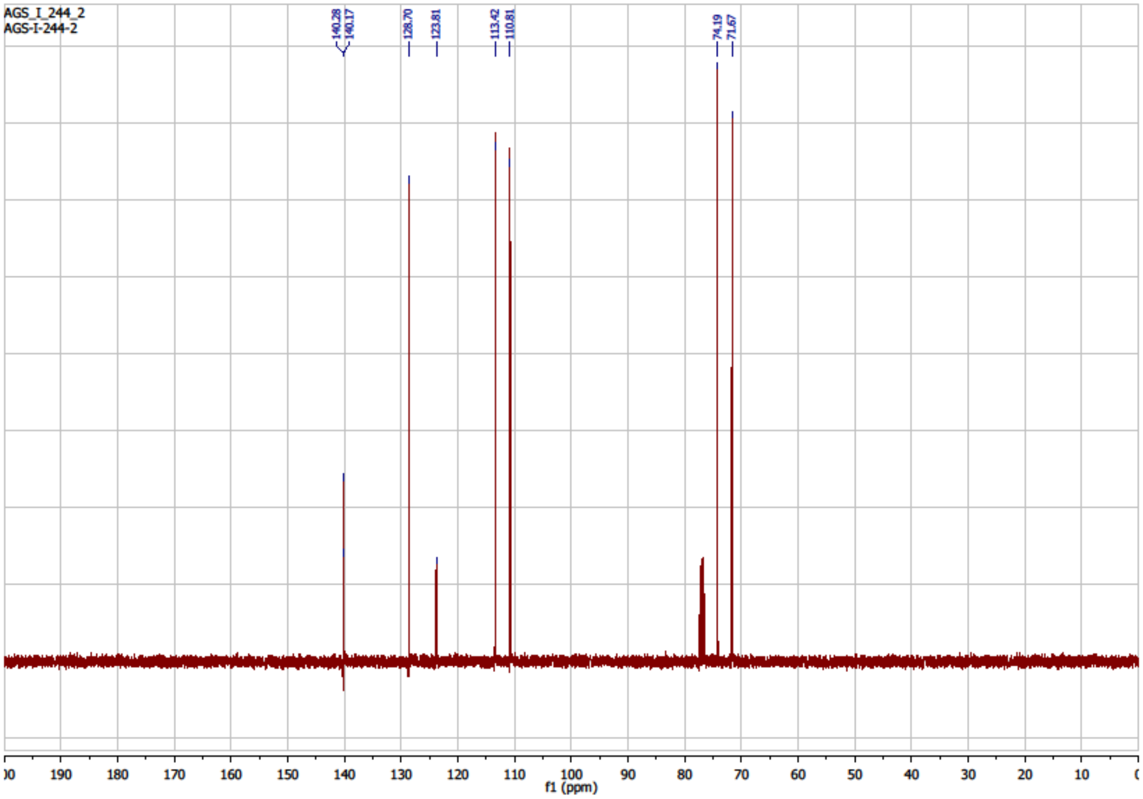

1,3-dihydroisobenzofuran-5-amine (S38).

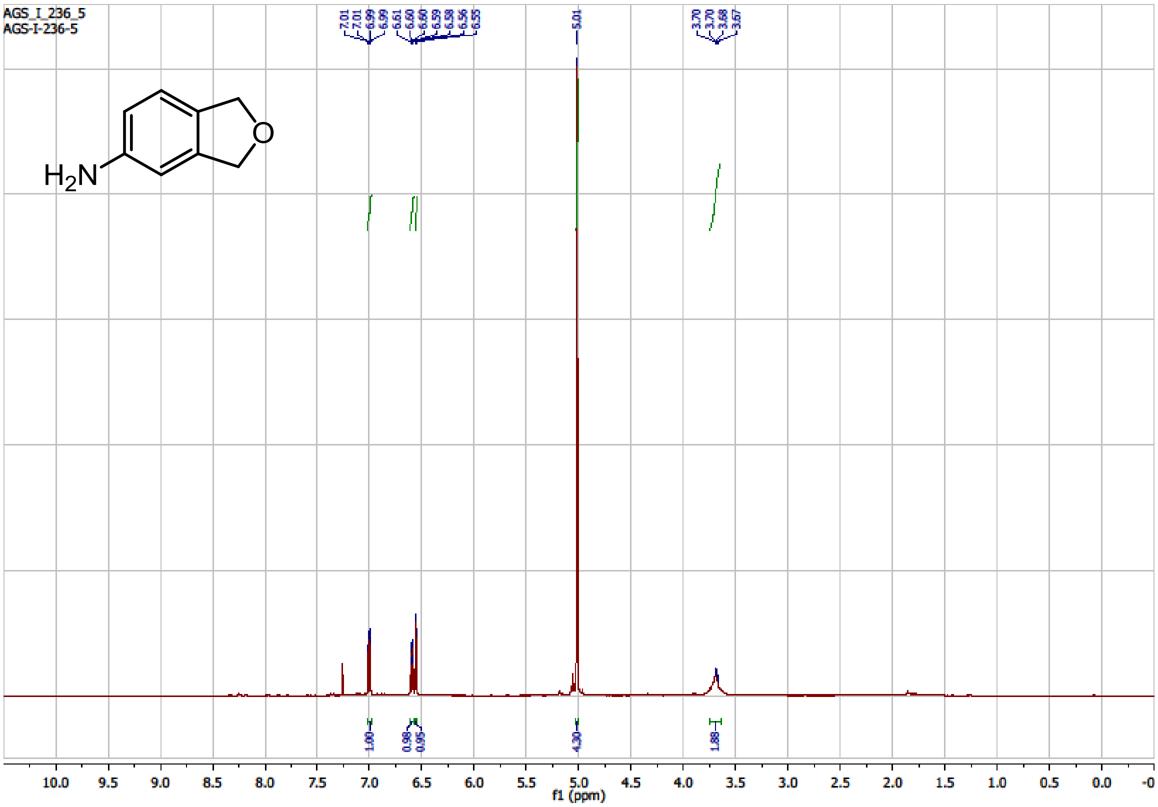

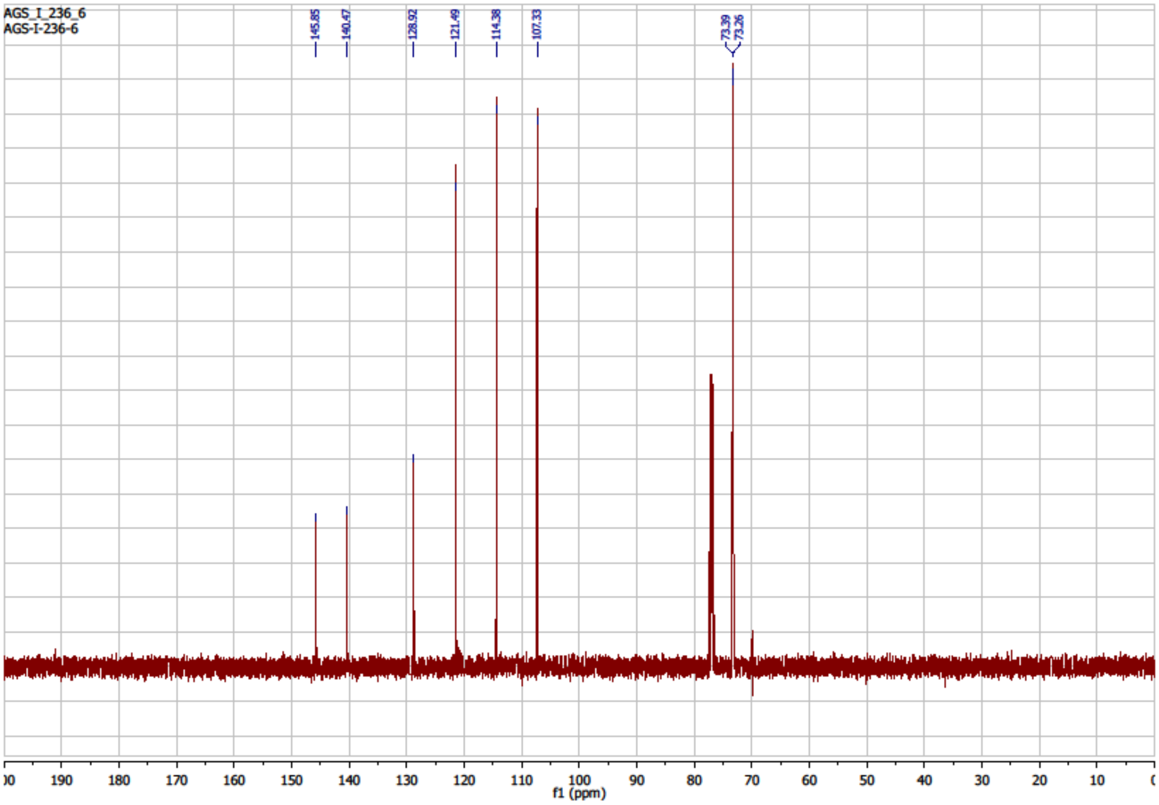

1-(1,3-dihydroisobenzofuran-4-yl)pyrrolidine (S39).

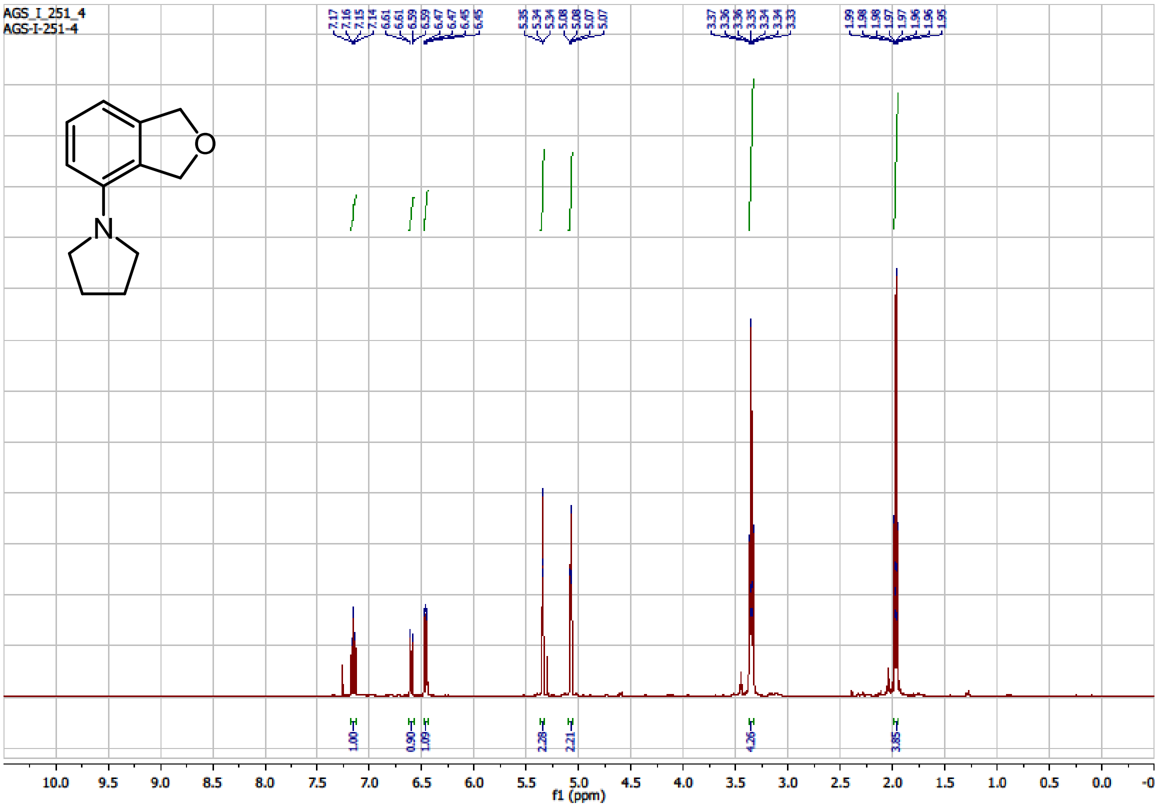

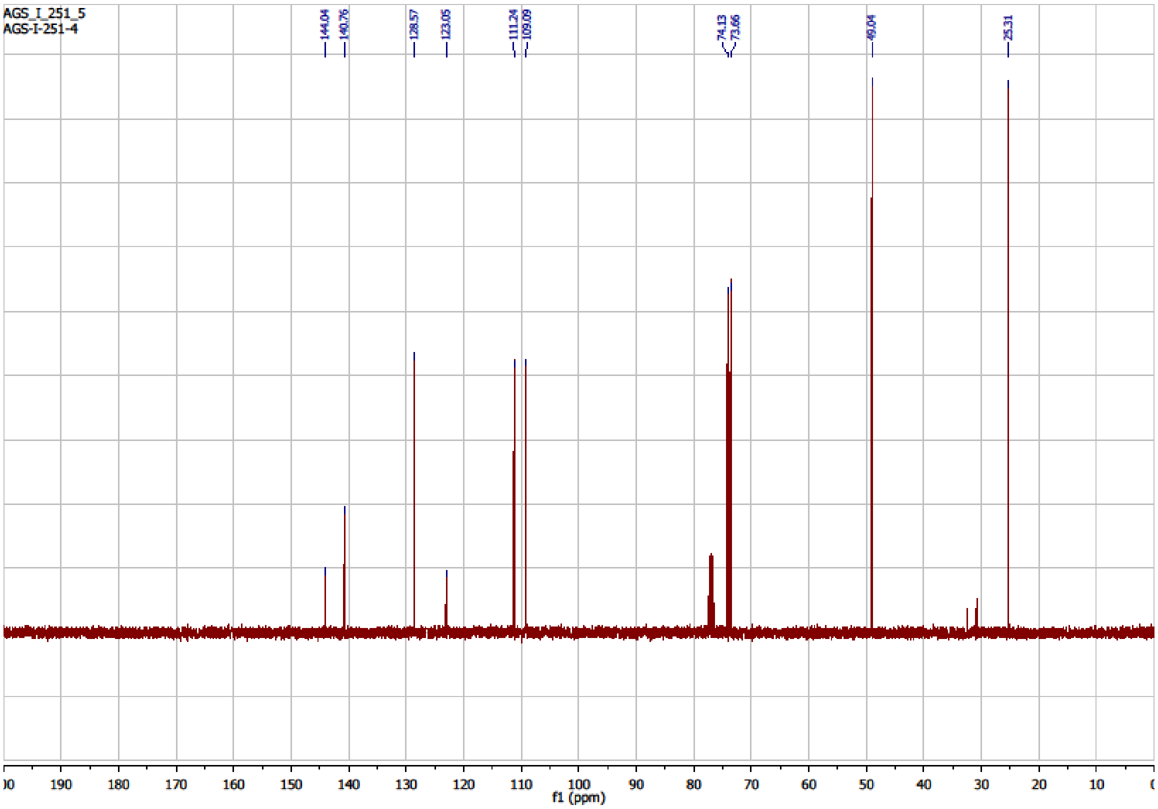

1-(1,3-dihydroisobenzofuran-5-yl)pyrrolidine (S40).

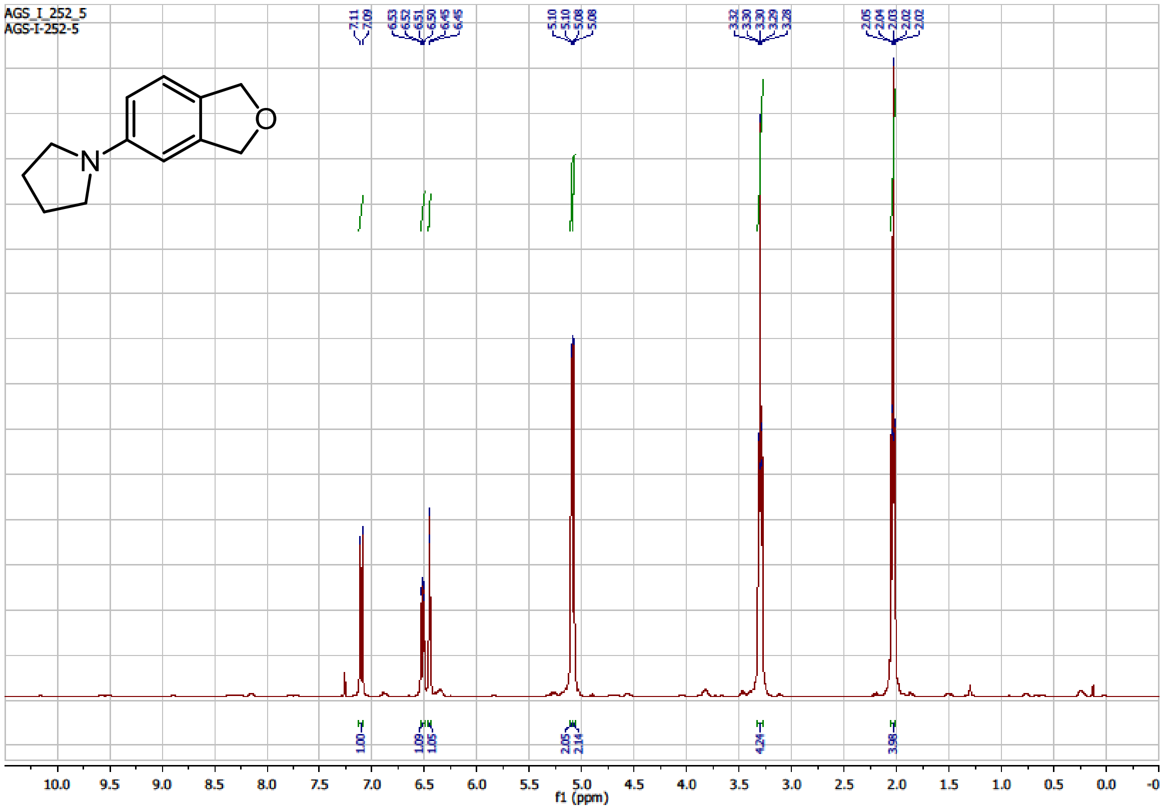

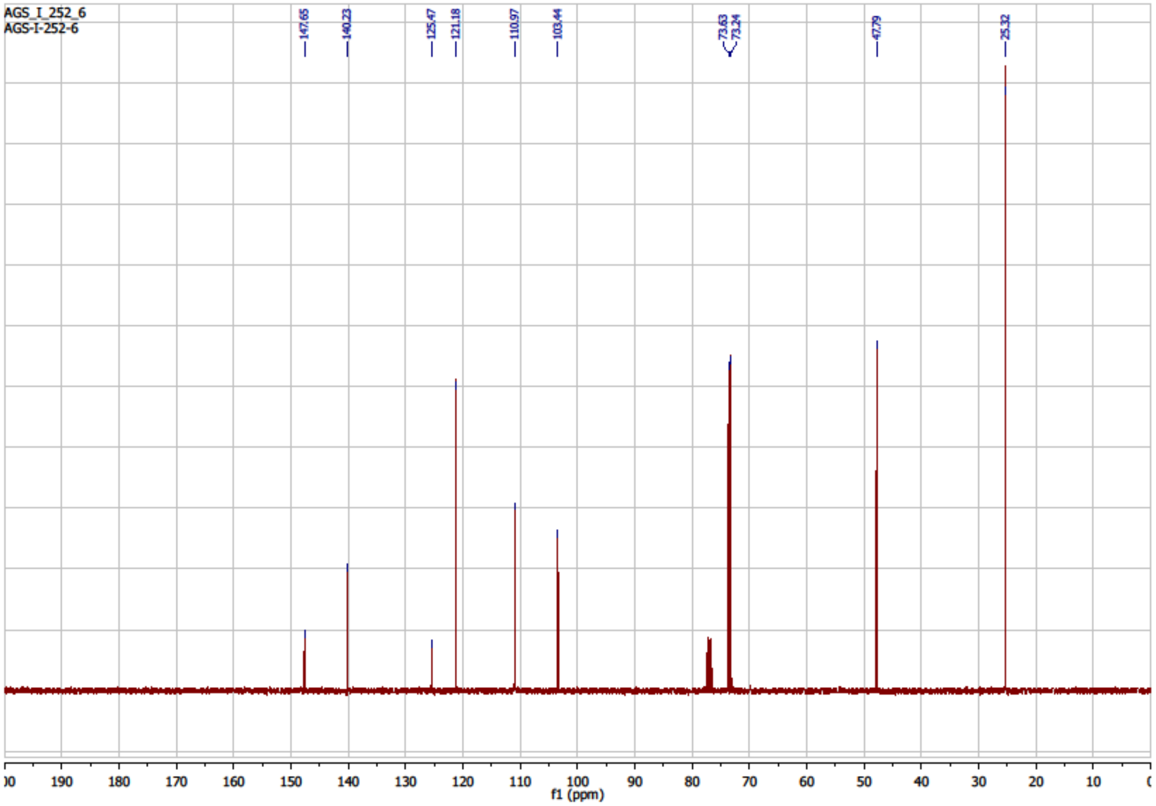

2-phenylprop-2-en-1-ol (S41).

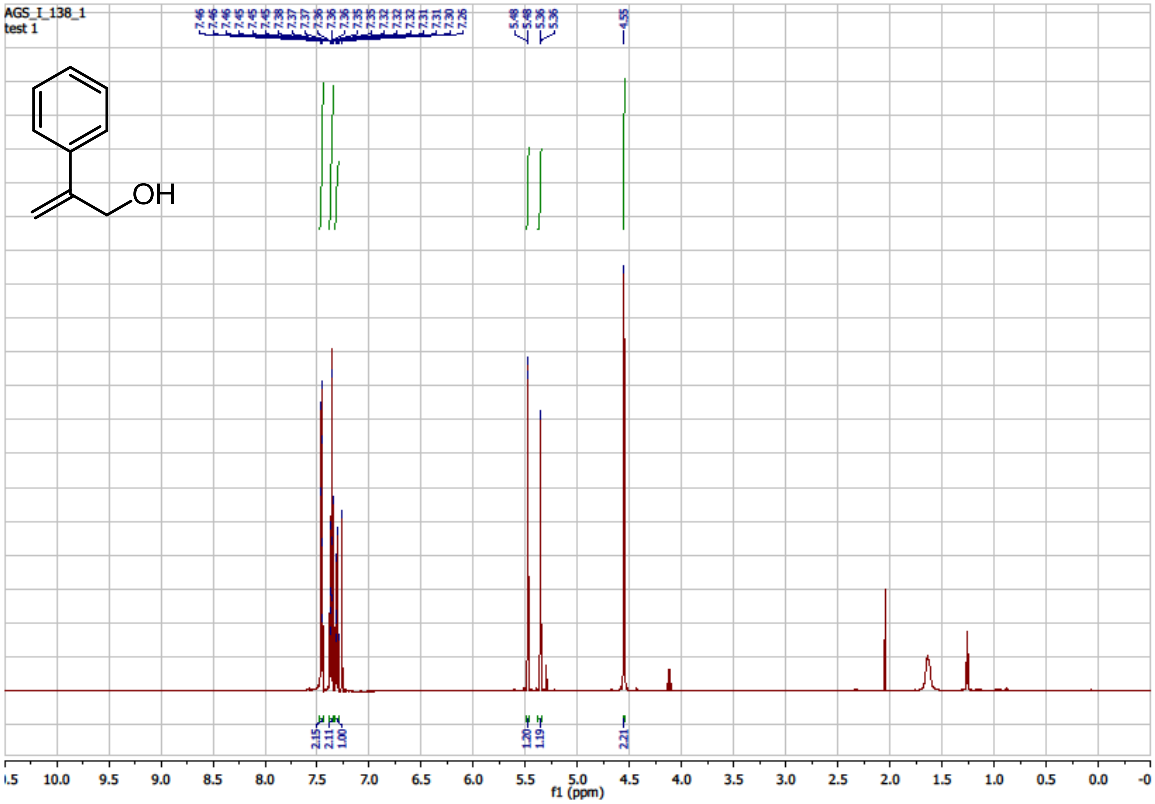

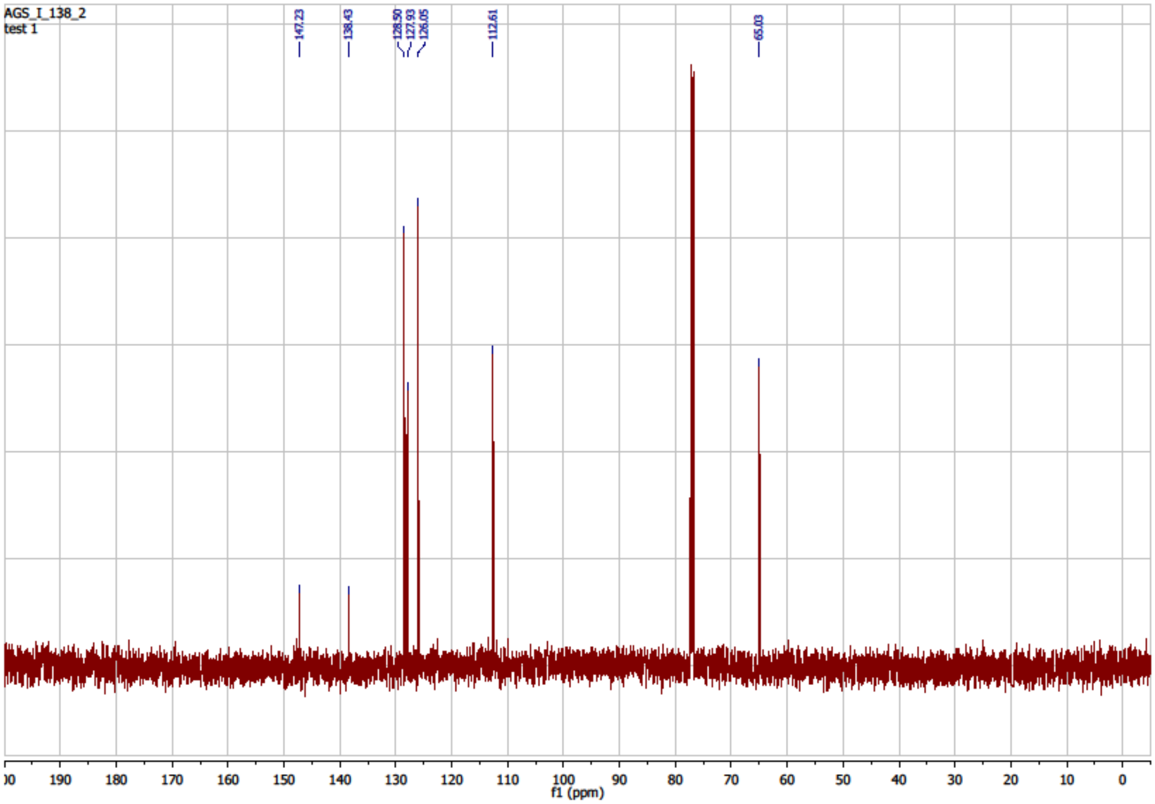

(2-((allyloxy)methyl)allyl)benzene (S44).

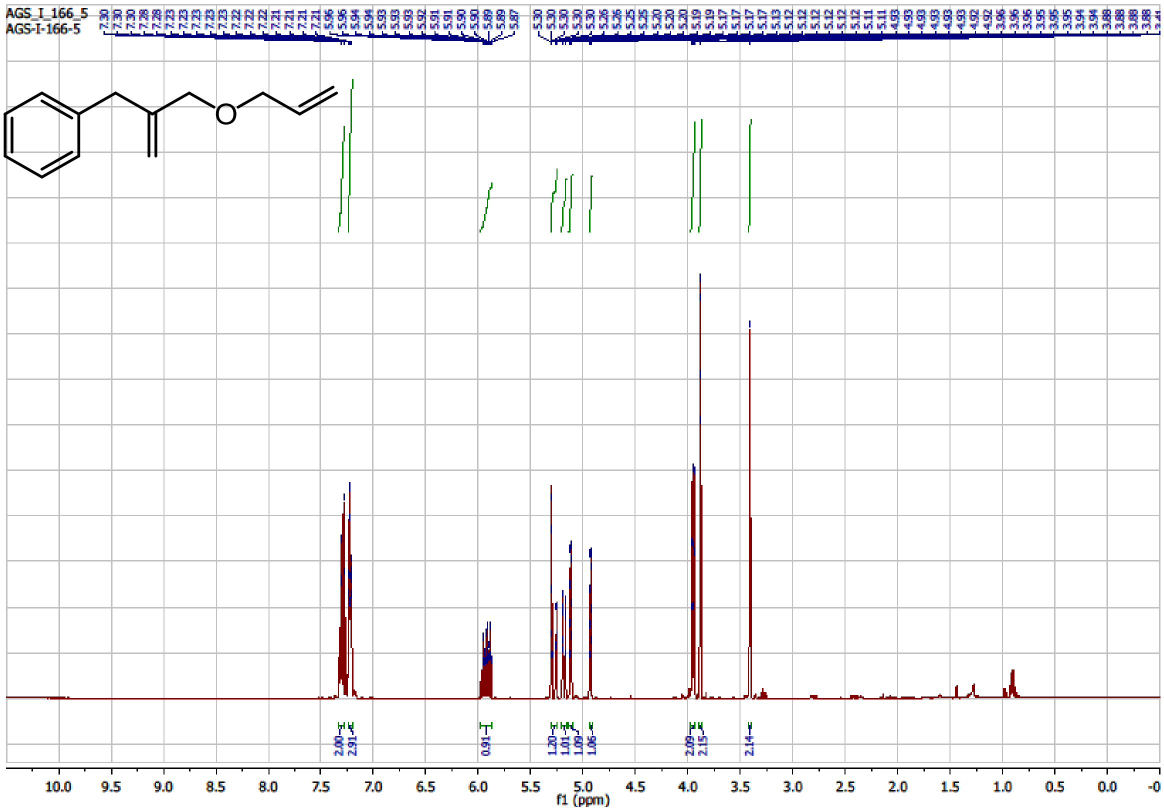

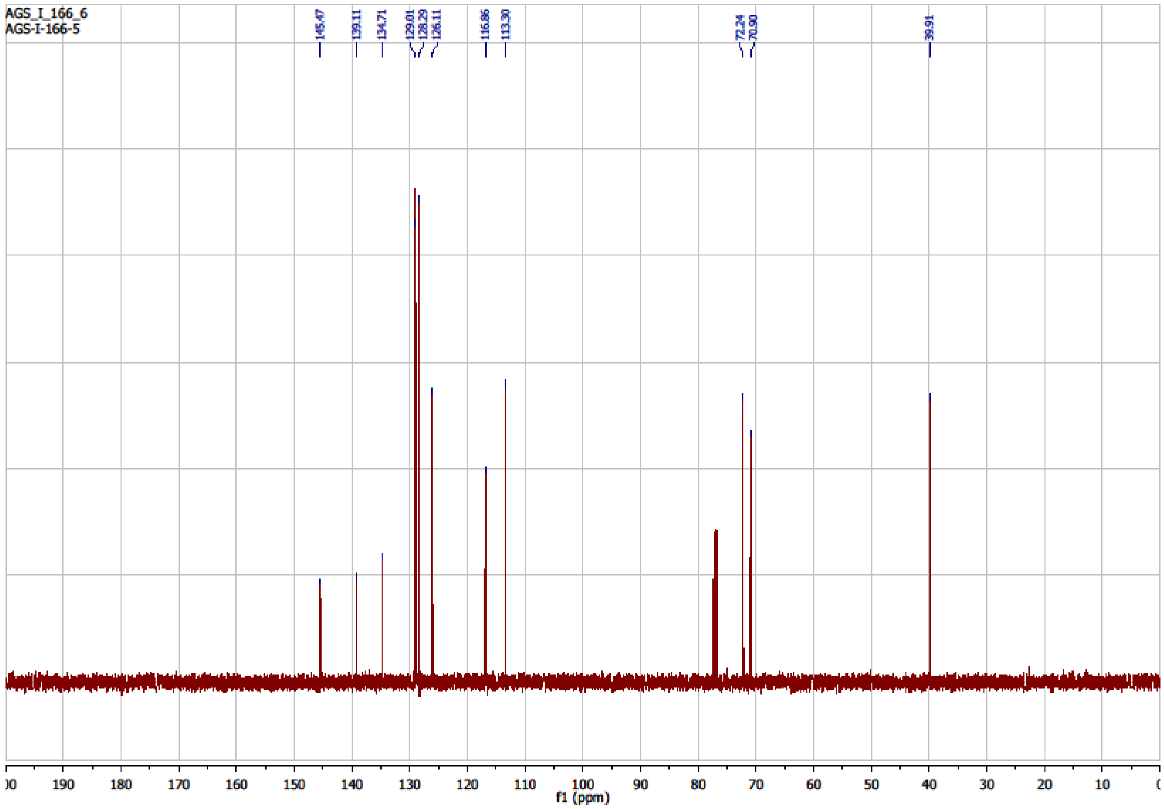

3-benzyl-2,5-dihydrofuran (S45).

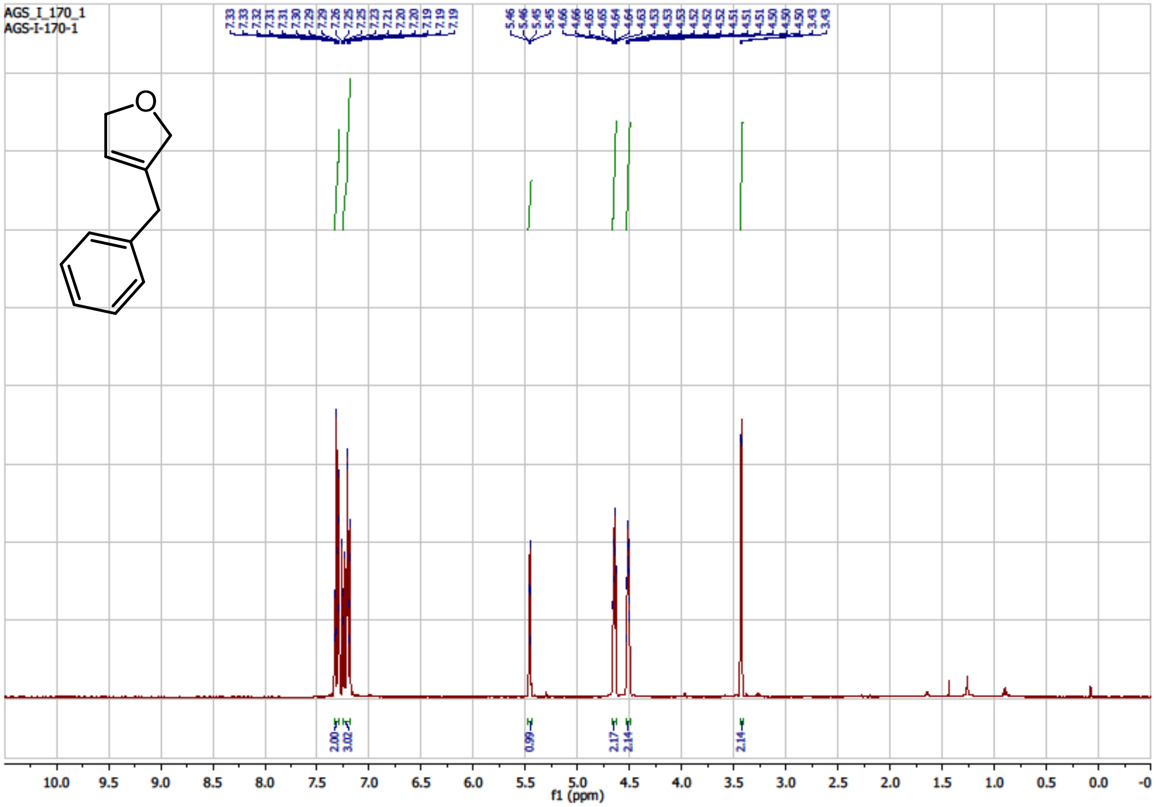

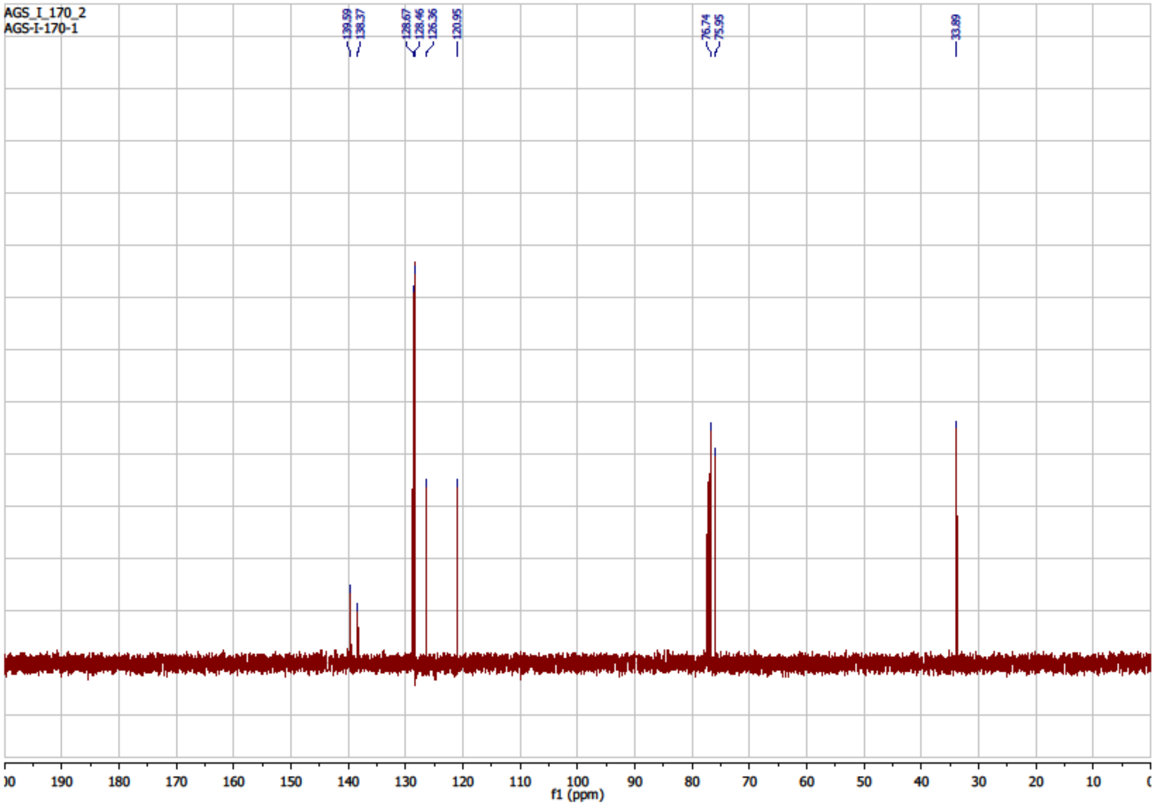

(*S*)-ethyl 2-(tetrahydrofuran-2-yl)acetate (S46).

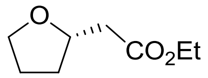

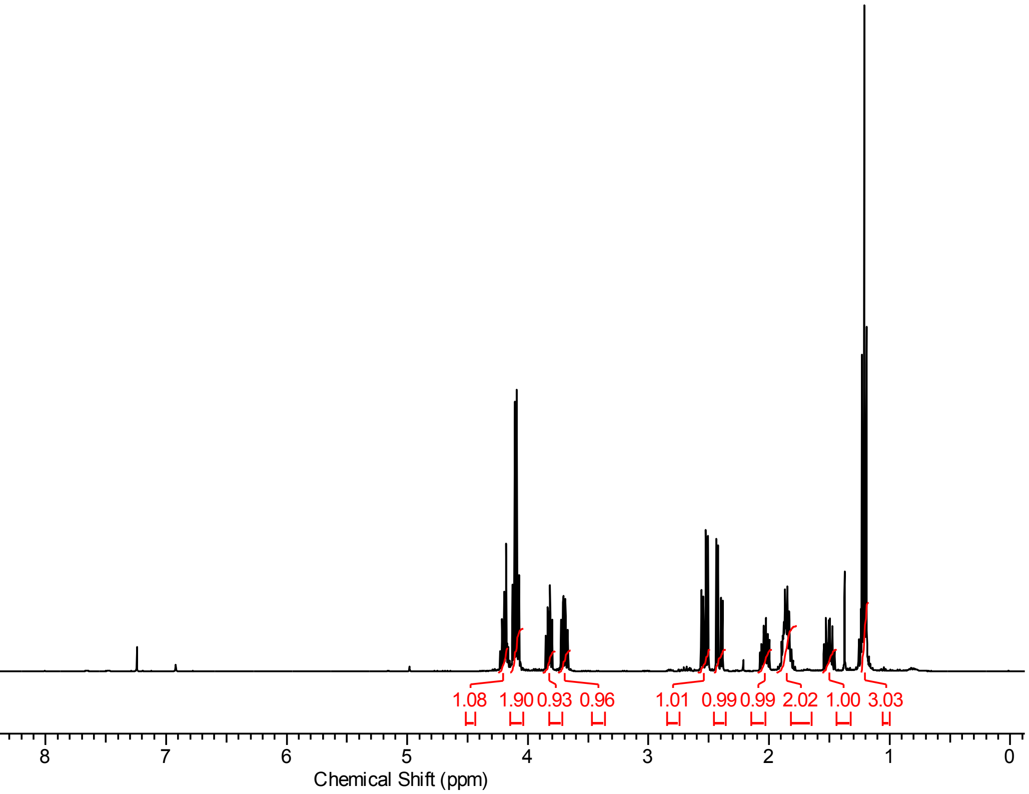

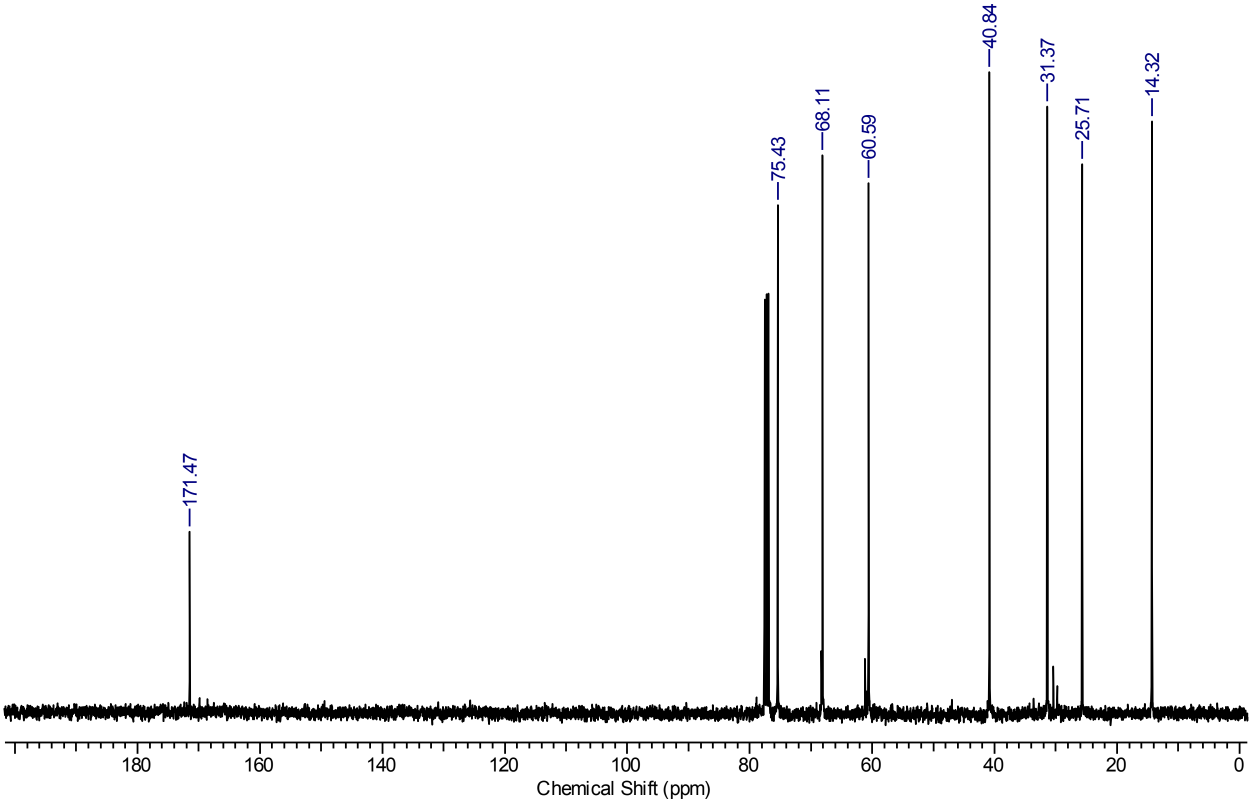

(*R*)-ethyl 2-(1,3-dihydroisobenzofuran-1-yl)acetate (S47).

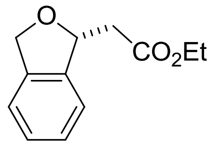

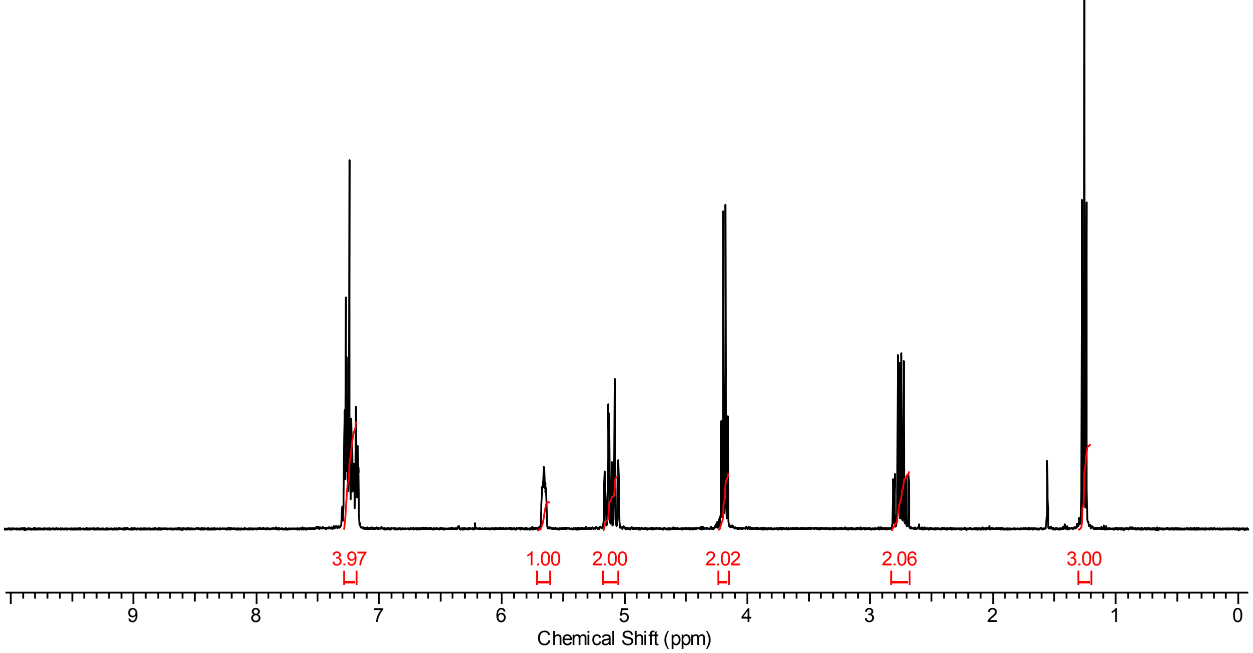

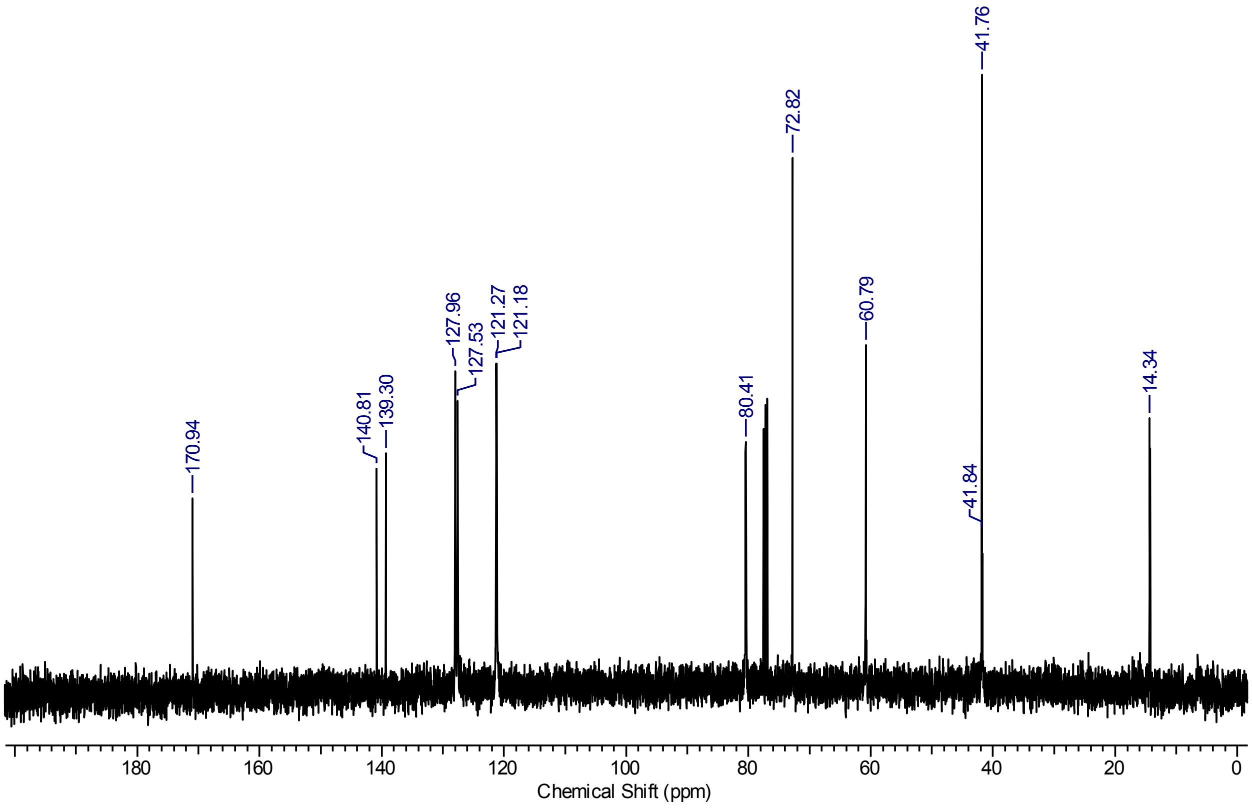

(*R*)-methyl 2-(1,3-dihydroisobenzofuran-1-yl)acetate (S48).

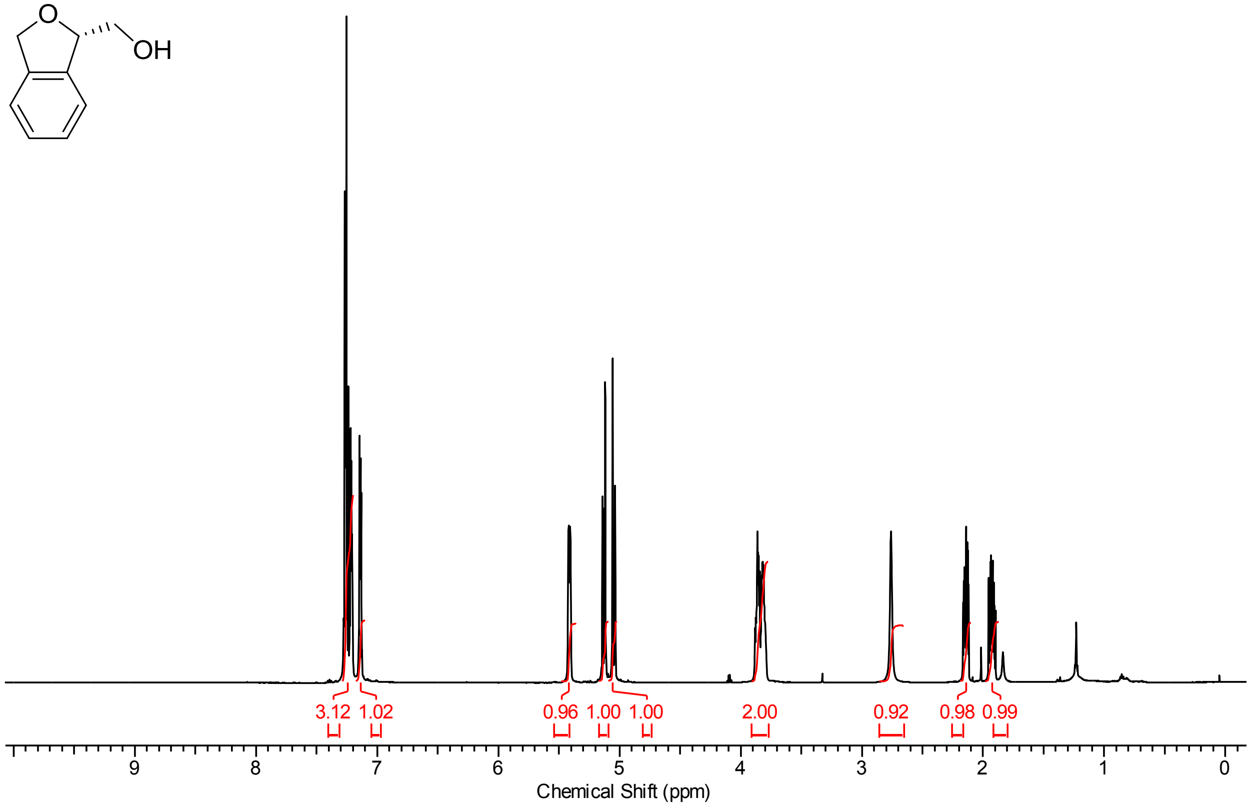

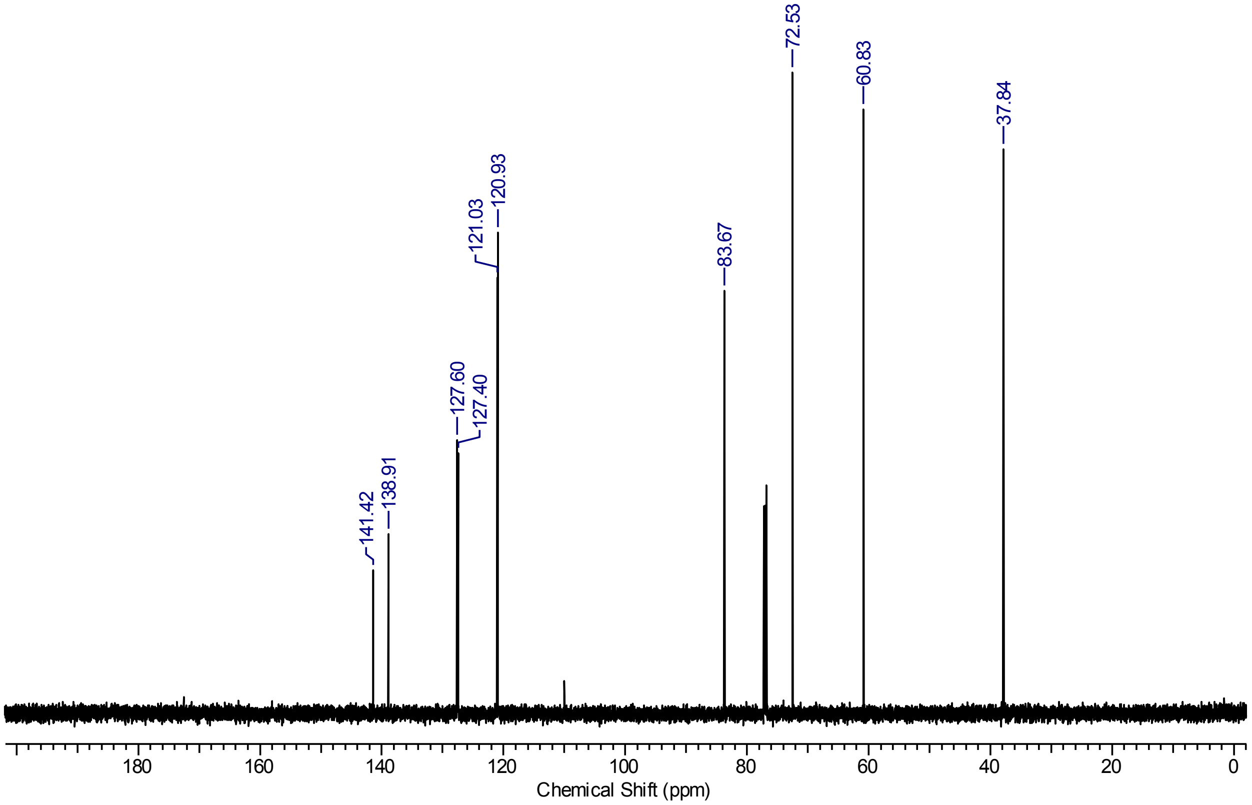

(*R*)-methyl 2-(1,3-dihydroisobenzofuran-1-yl)acetate (S49).

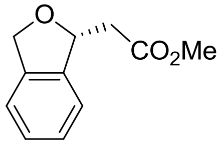

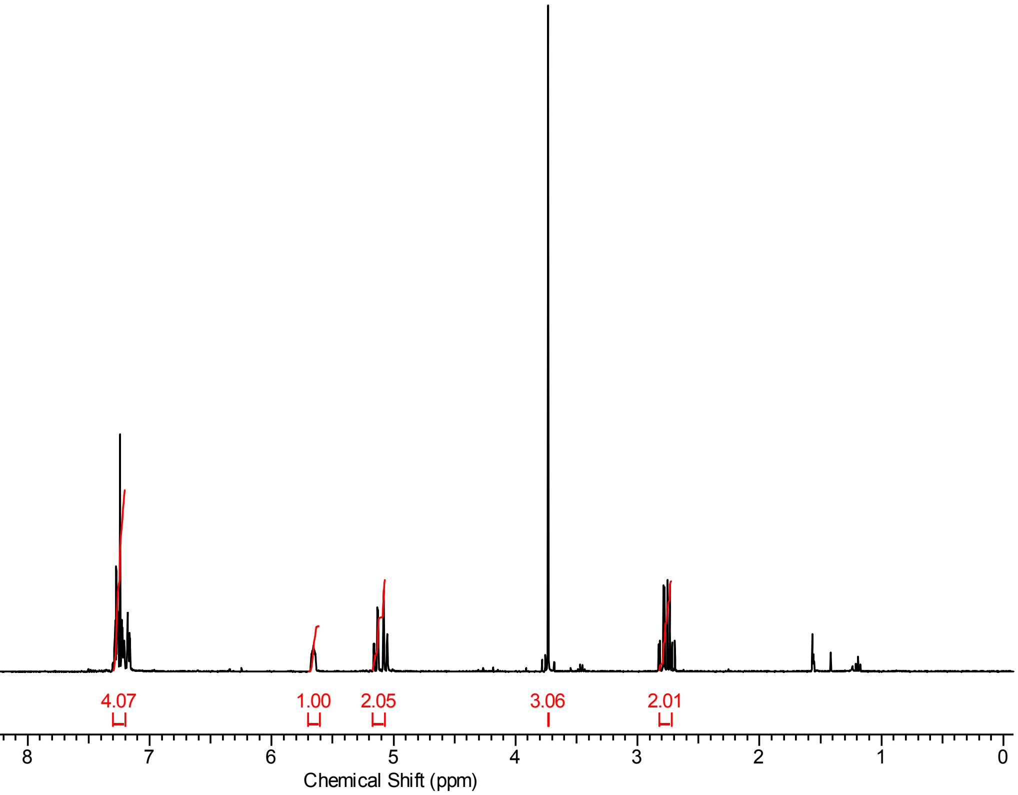

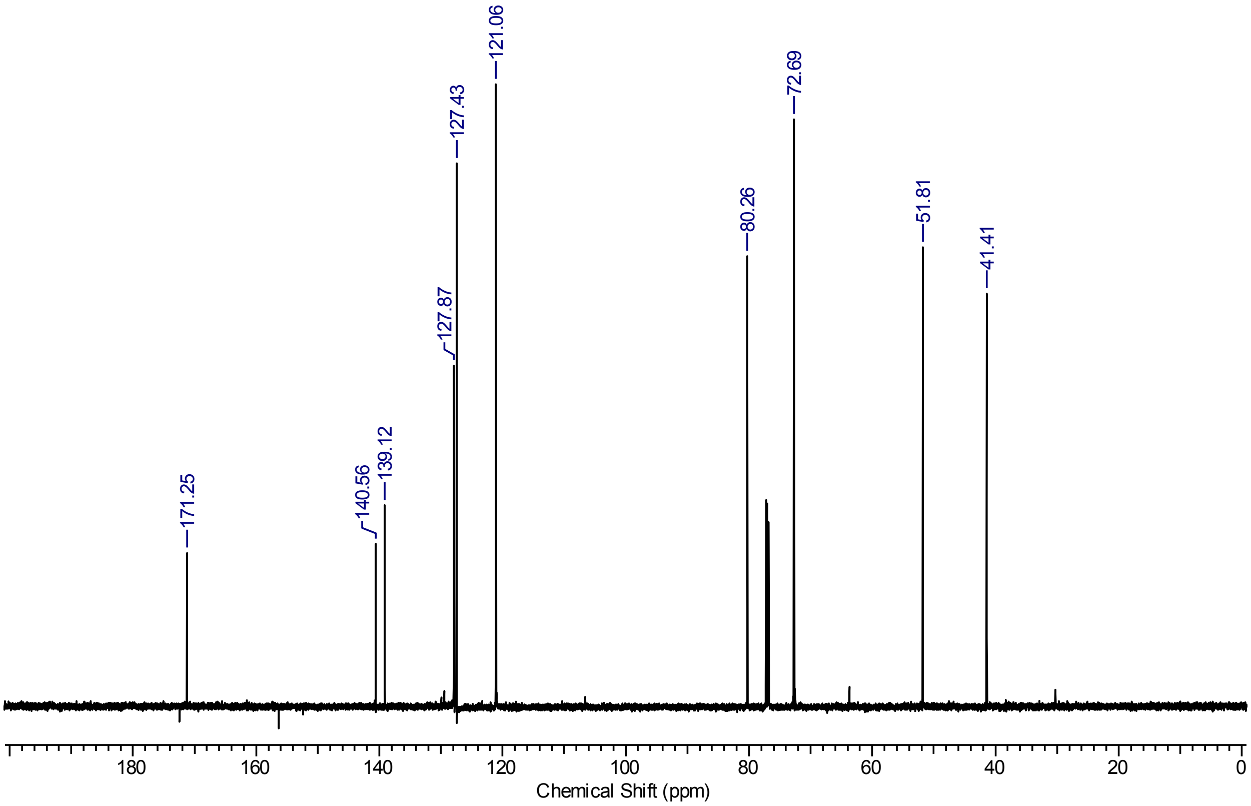

(*R*)-2,2,2-trichloroethyl 2-(1,3-dihydroisobenzofuran-1-yl)acetate (S50).

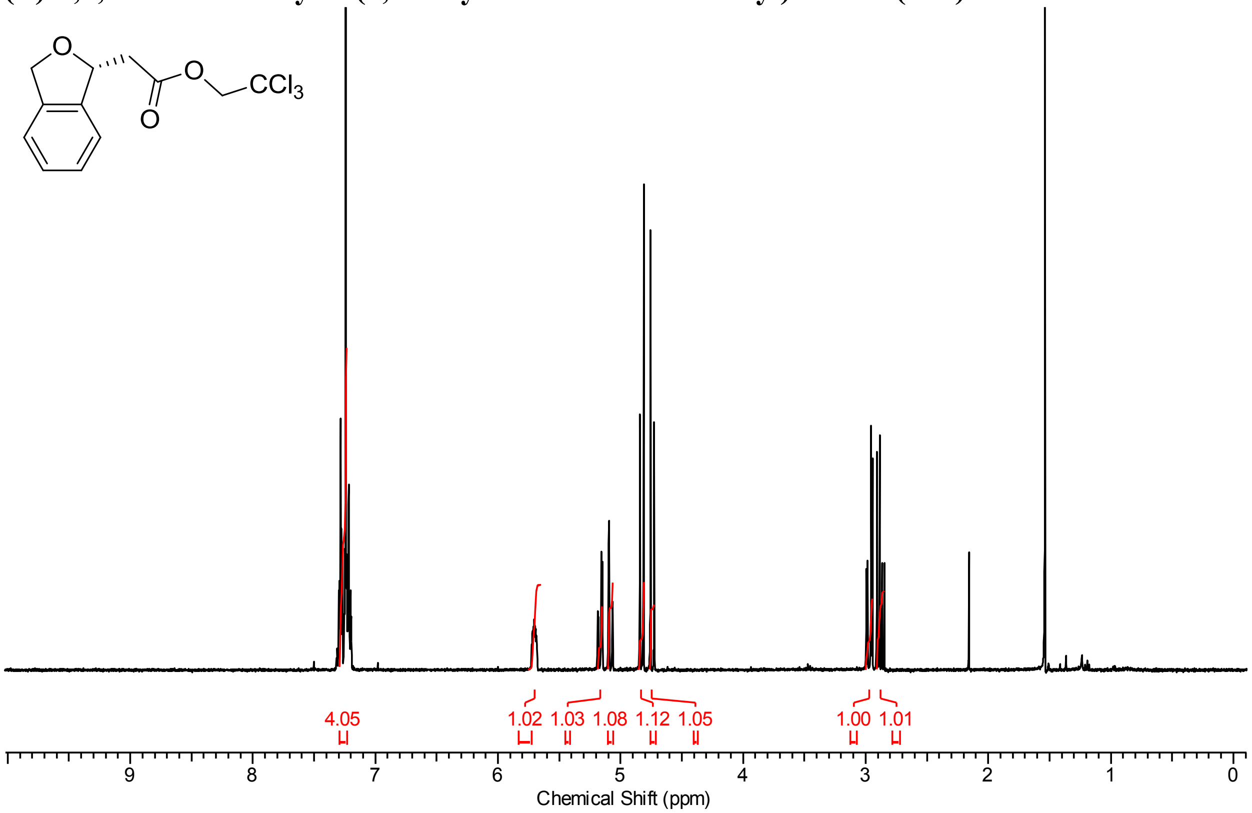

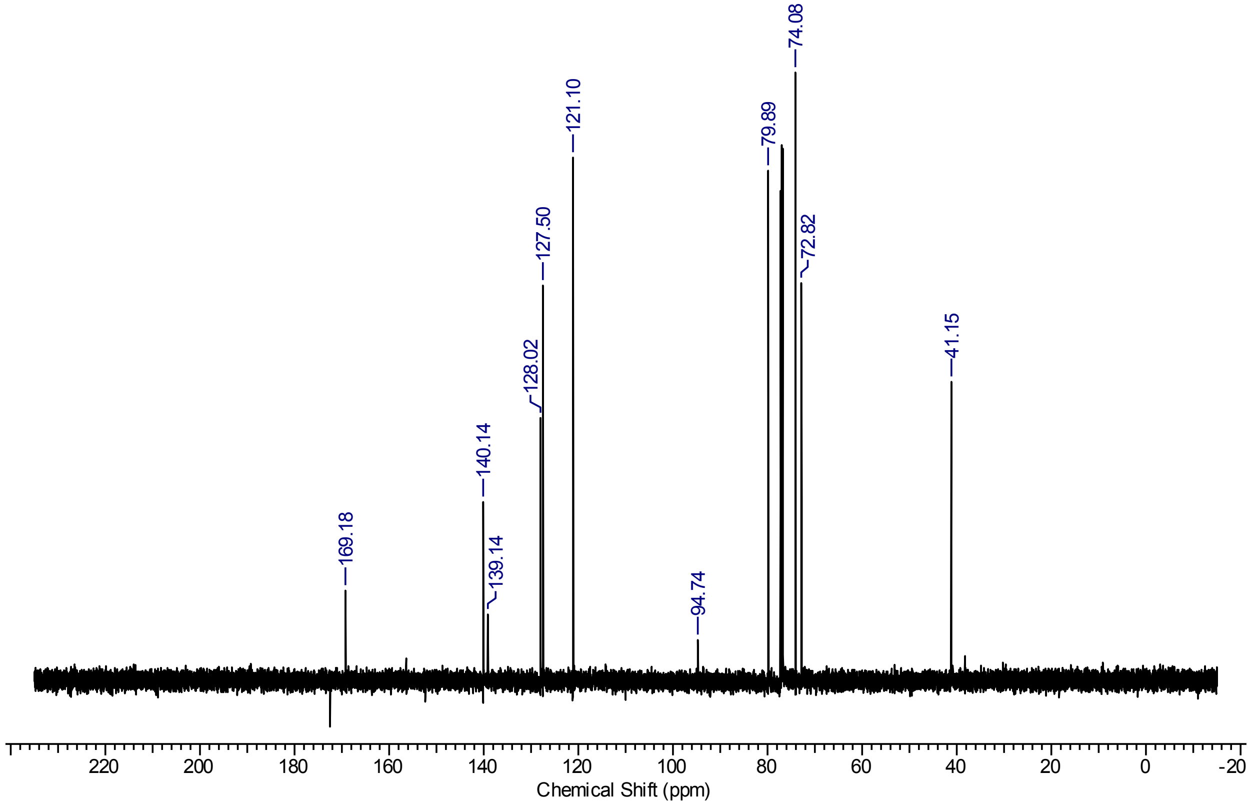

(*R*)-2-(trimethylsilyl)ethyl 2-(1,3-dihydroisobenzofuran-1-yl)acetate (S51).

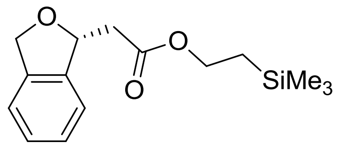

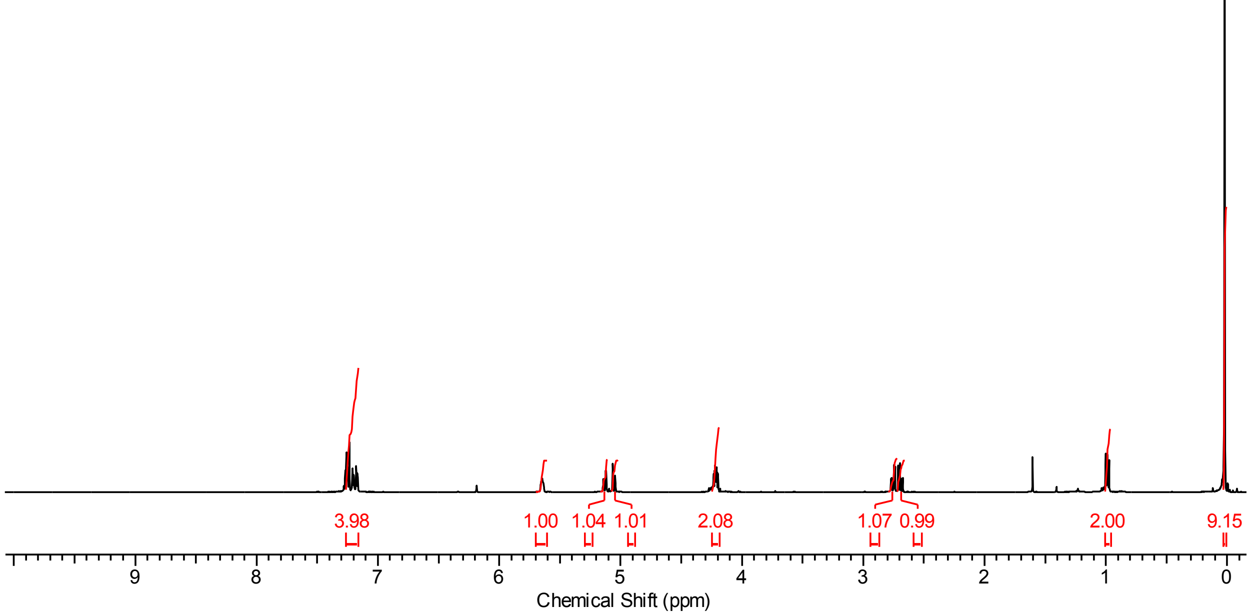

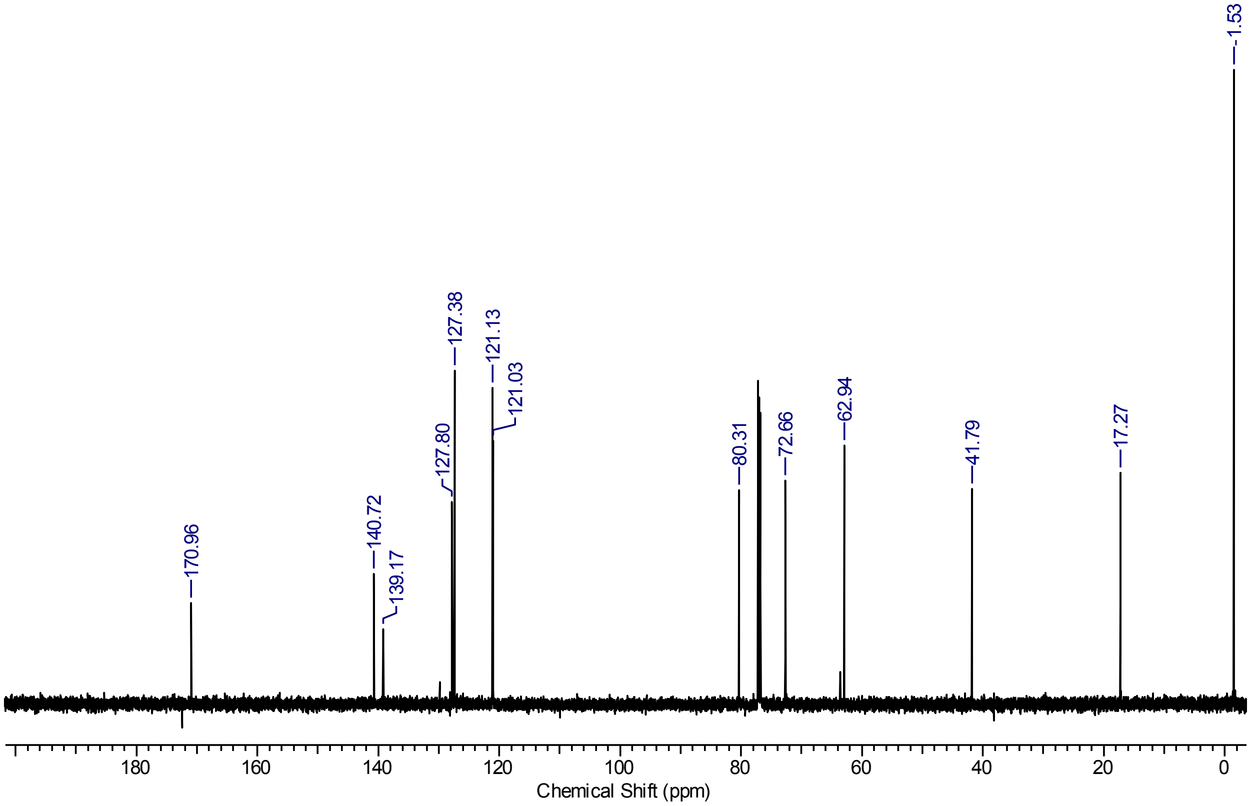

(*R*)-phenyl 2-(1,3-dihydroisobenzofuran-1-yl)acetate (S52).

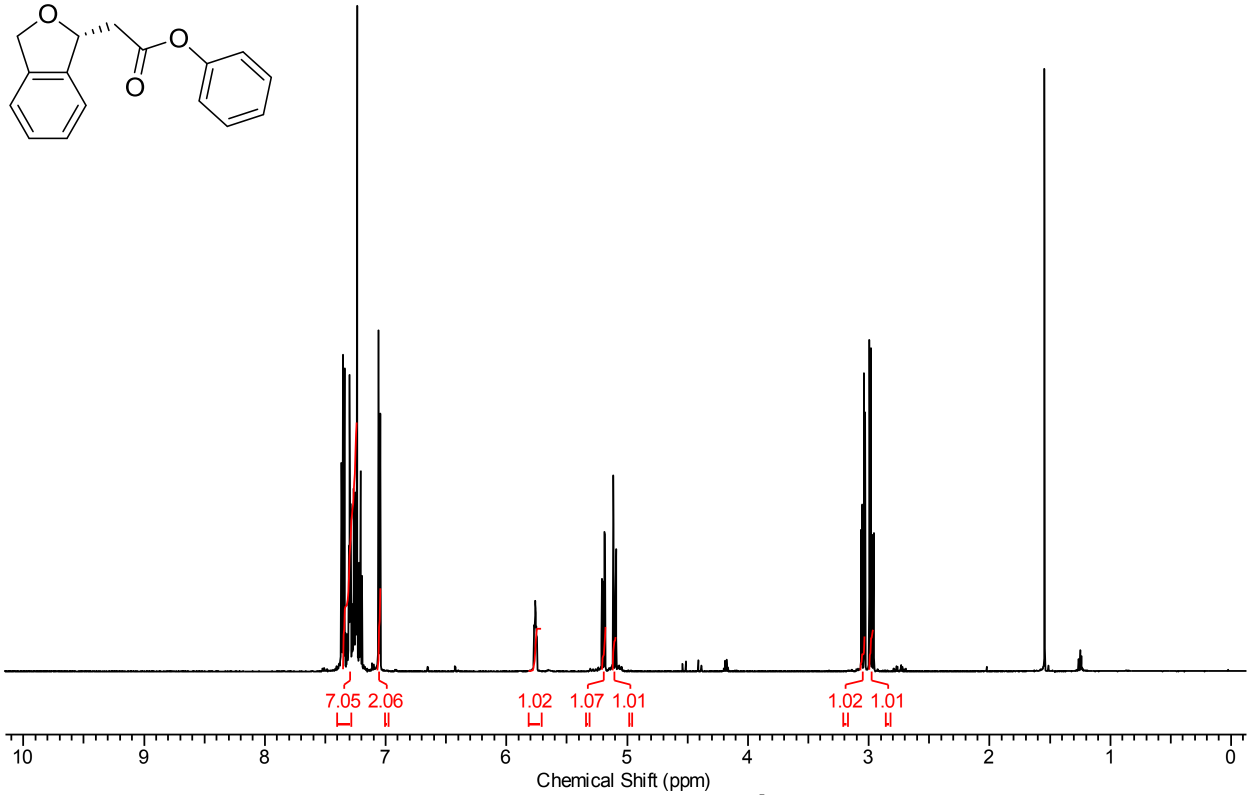

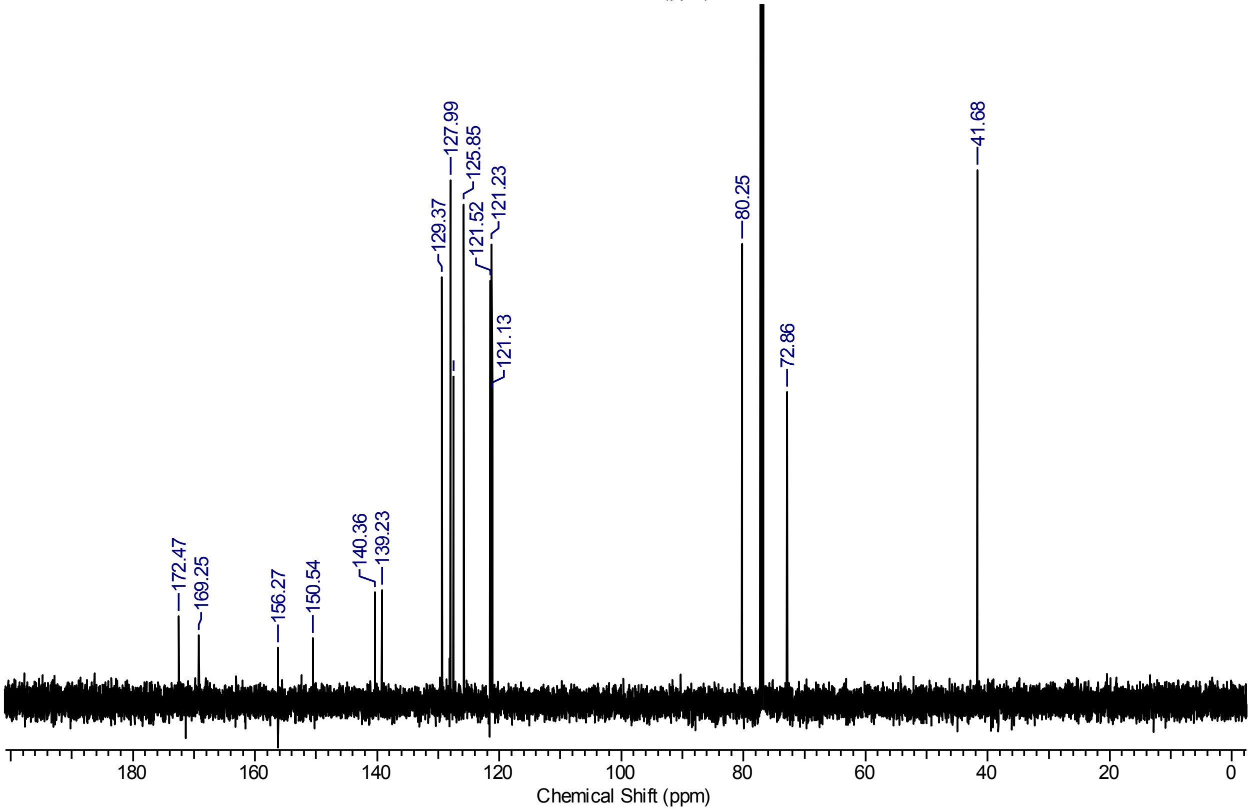

(*R*)-4-methoxyphenyl 2-(1,3-dihydroisobenzofuran-1-yl)acetate (2).

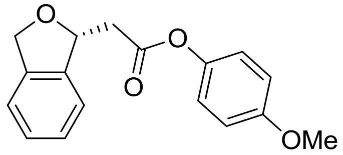

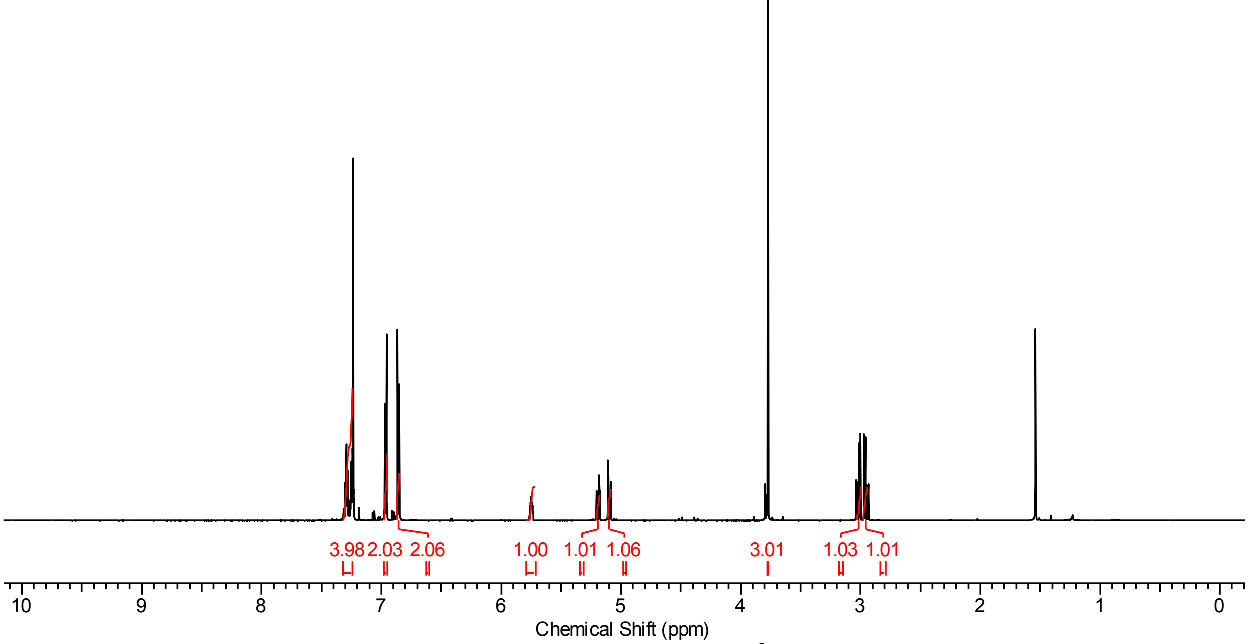

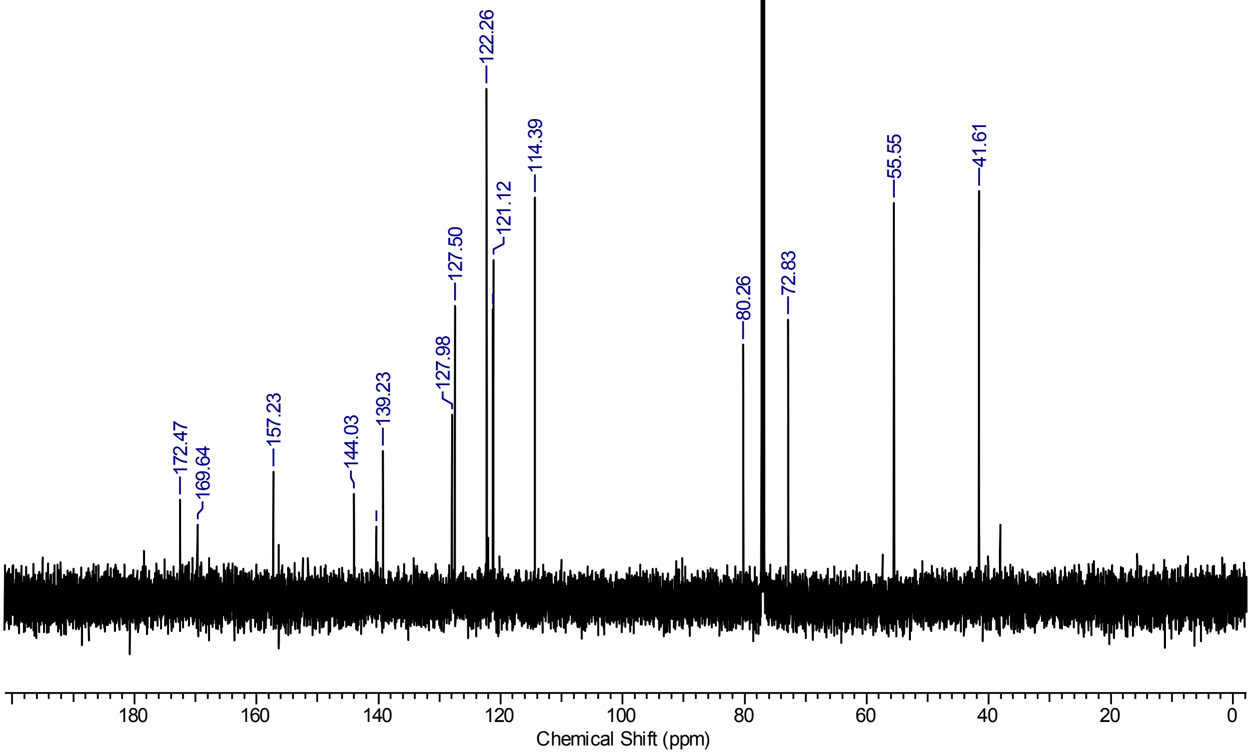

(*R*)-4-(trifluoromethyl)phenyl 2-(1,3-dihydroisobenzofuran-1-yl)acetate (S53).

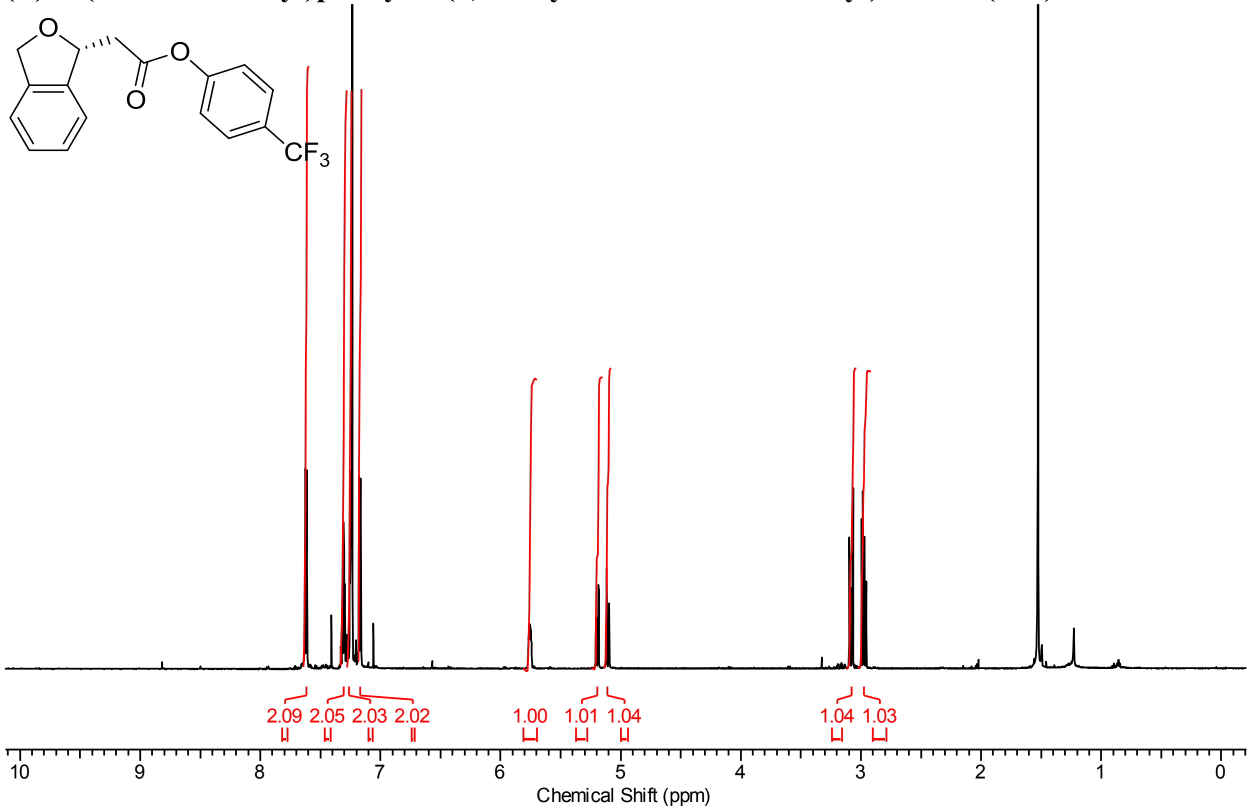

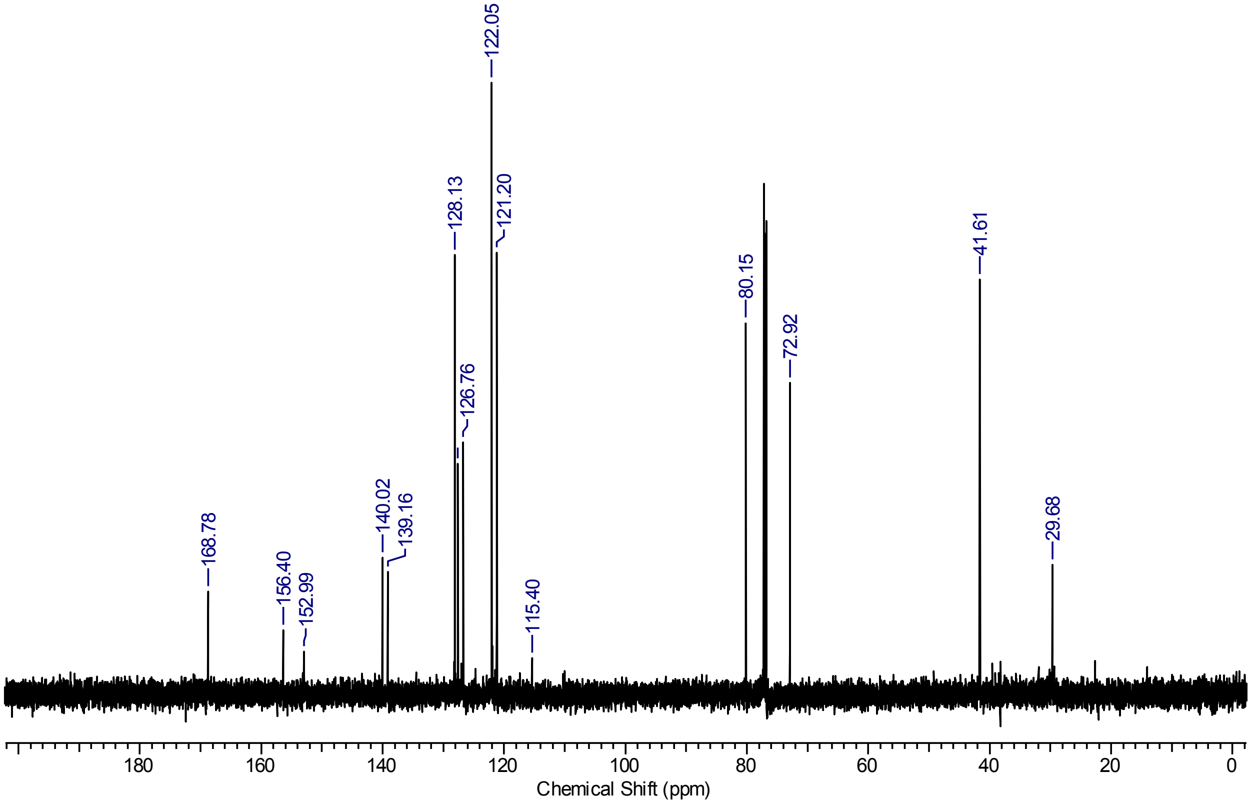

(*R*)-ethyl 2-(7-methoxy-1,3-dihydroisobenzofuran-1-yl)acetate (17).

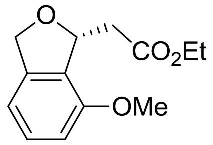

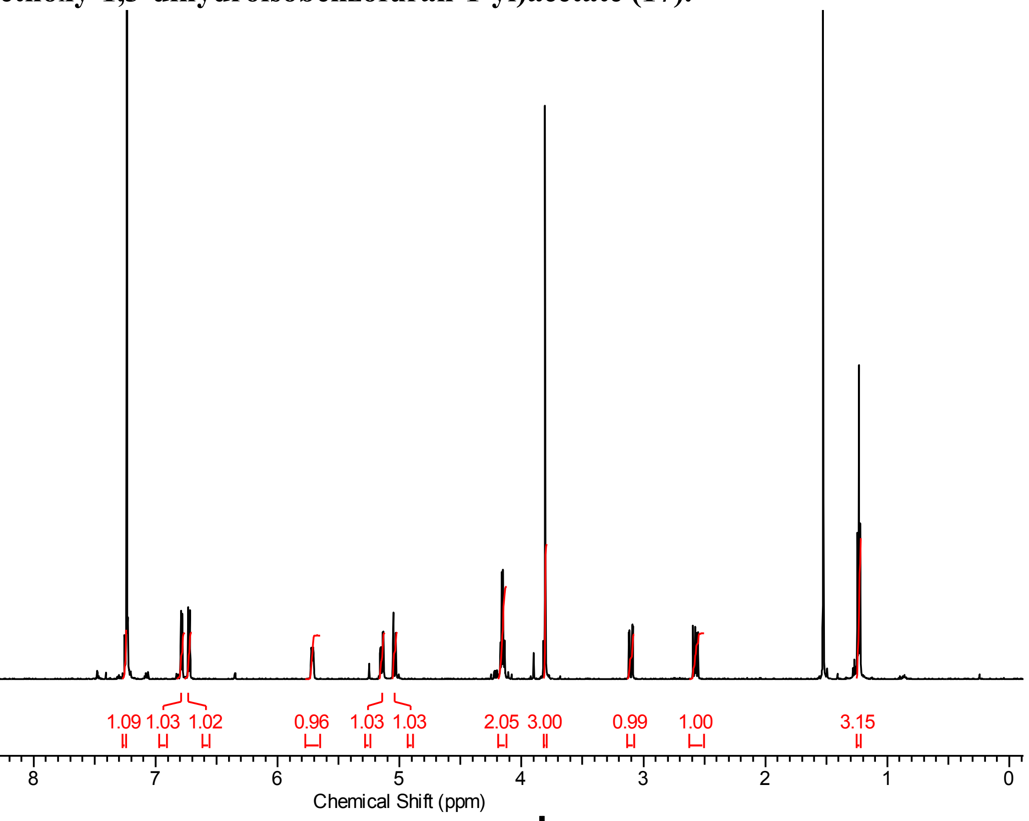

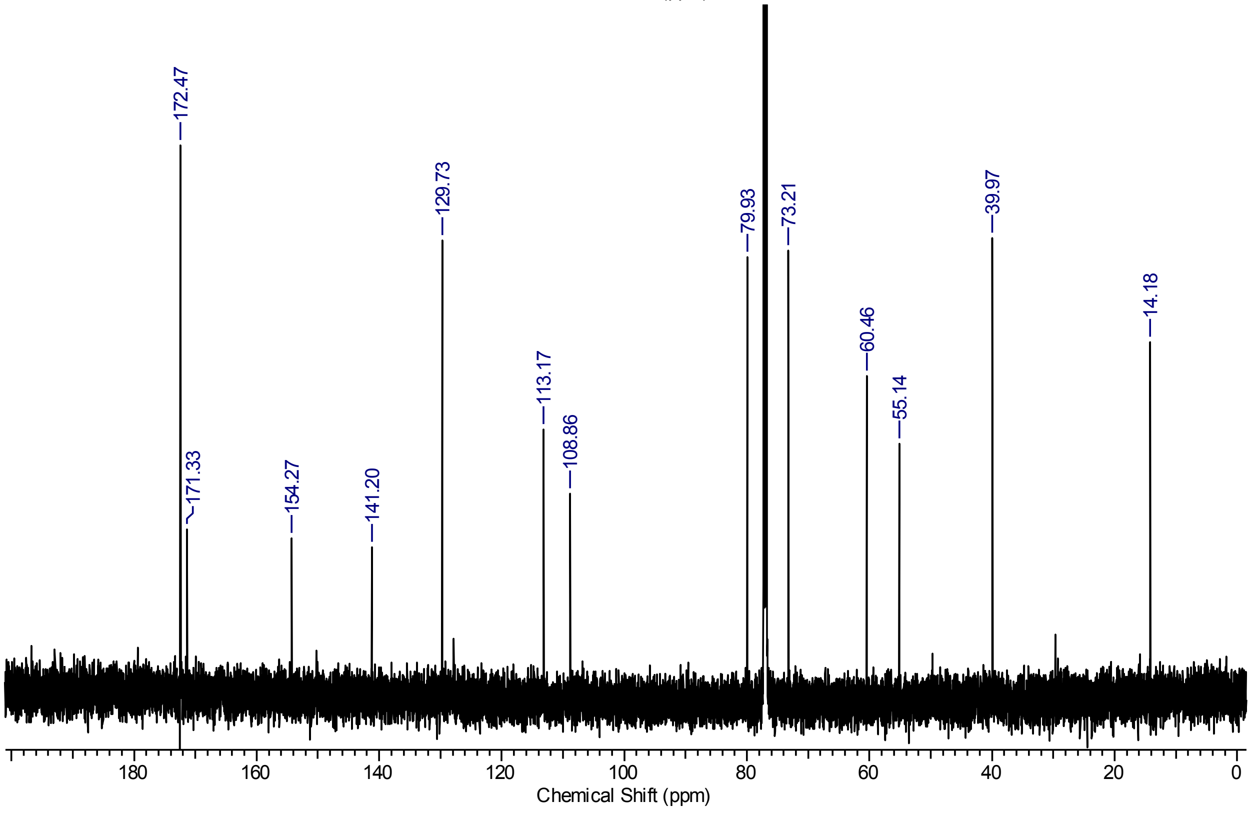

nOe spectra as evidence for **17** as the drawn regioisomer:

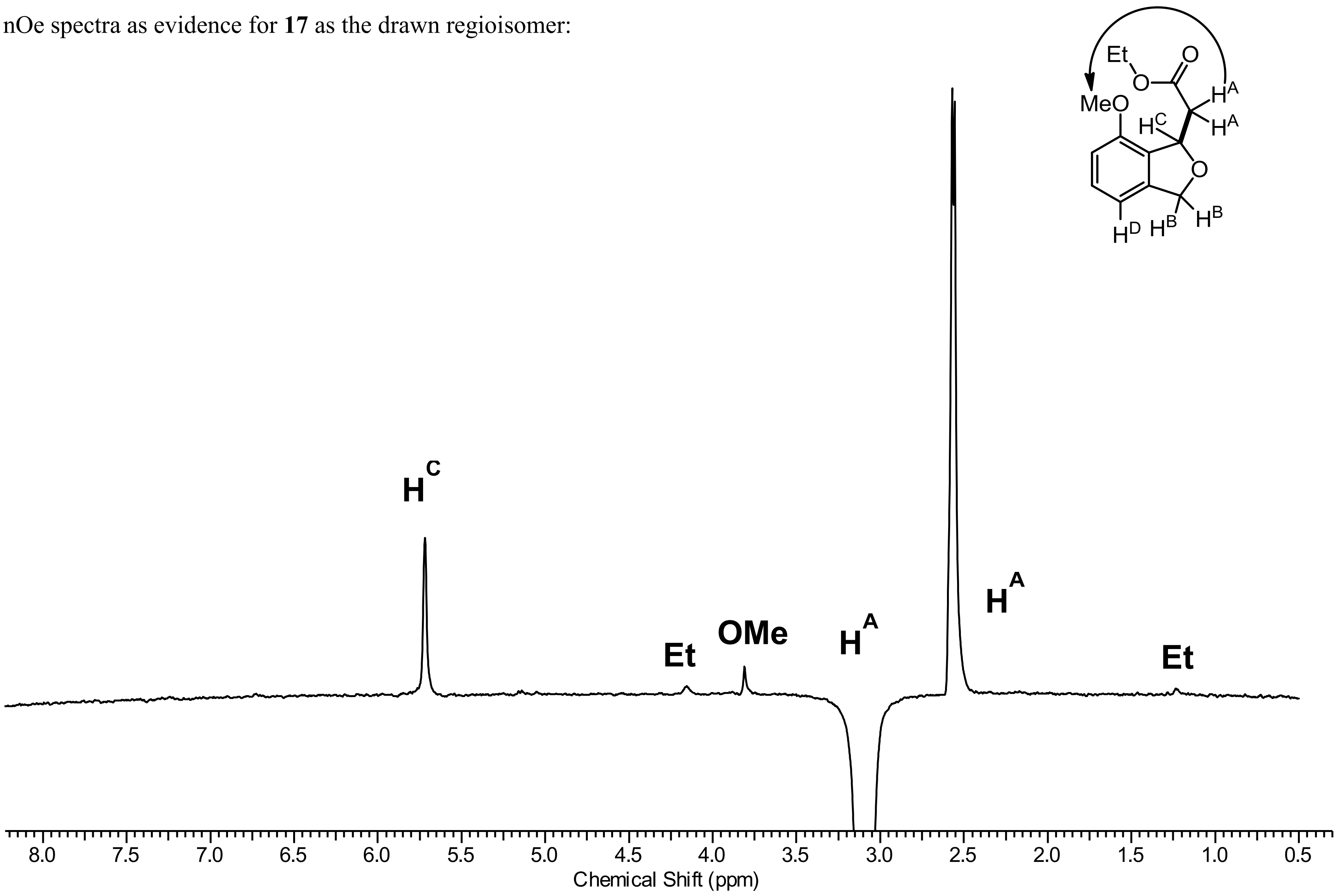

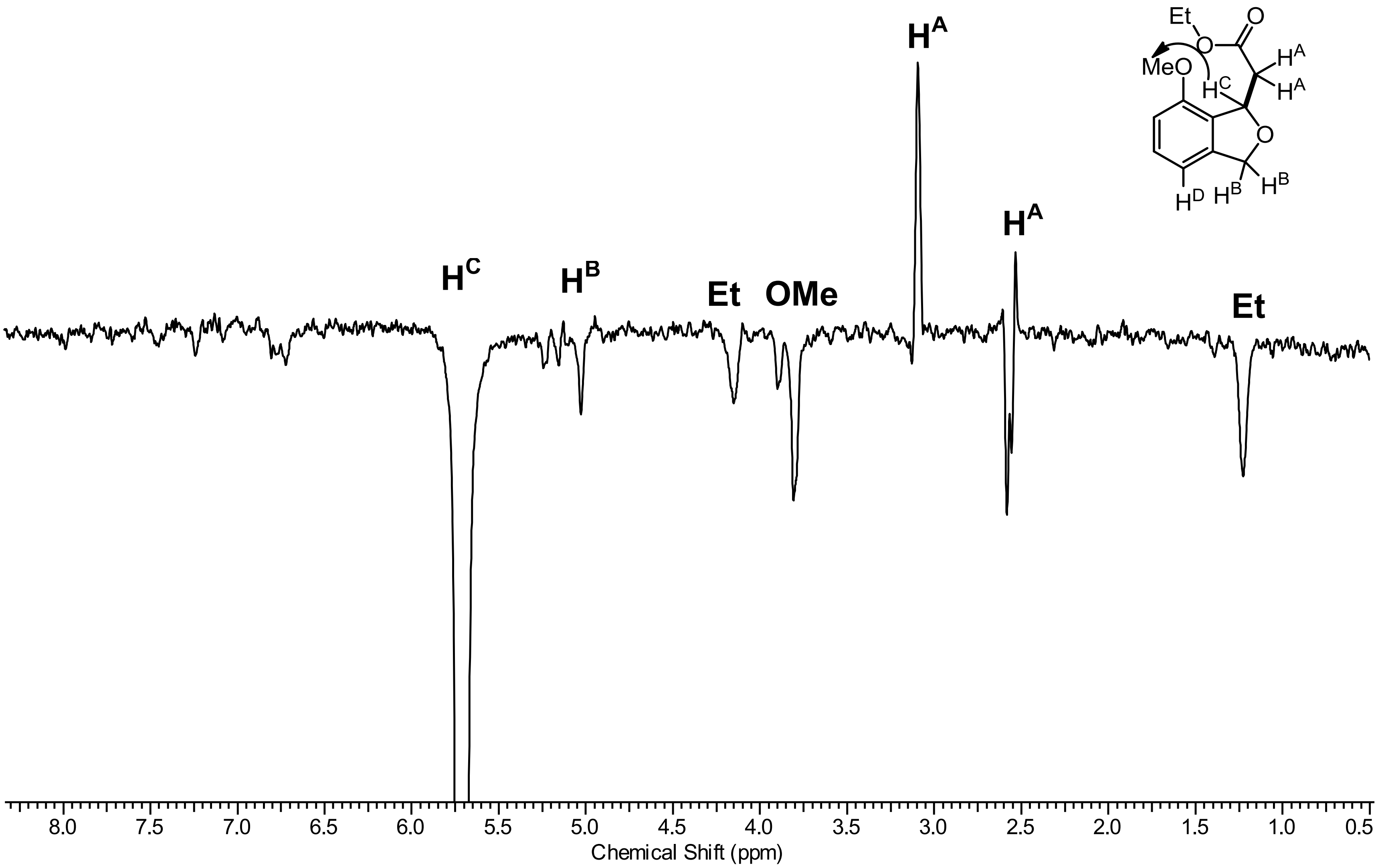

H^A
H^C
H^B
Et
OMe
H^A
Et
Et
O
MeO
O
H^C
H^A
H^A
O
H^D
H^B
H^B
8.0
7.5
7.0
6.5
6.0
5.5
5.0
4.5
4.0
3.5
3.0
2.5
2.0
1.5
1.0
0.5
Chemical Shift (ppm)

(*R*)-ethyl 2-(4-methoxy-1,3-dihydroisobenzofuran-1-yl)acetate (S54).

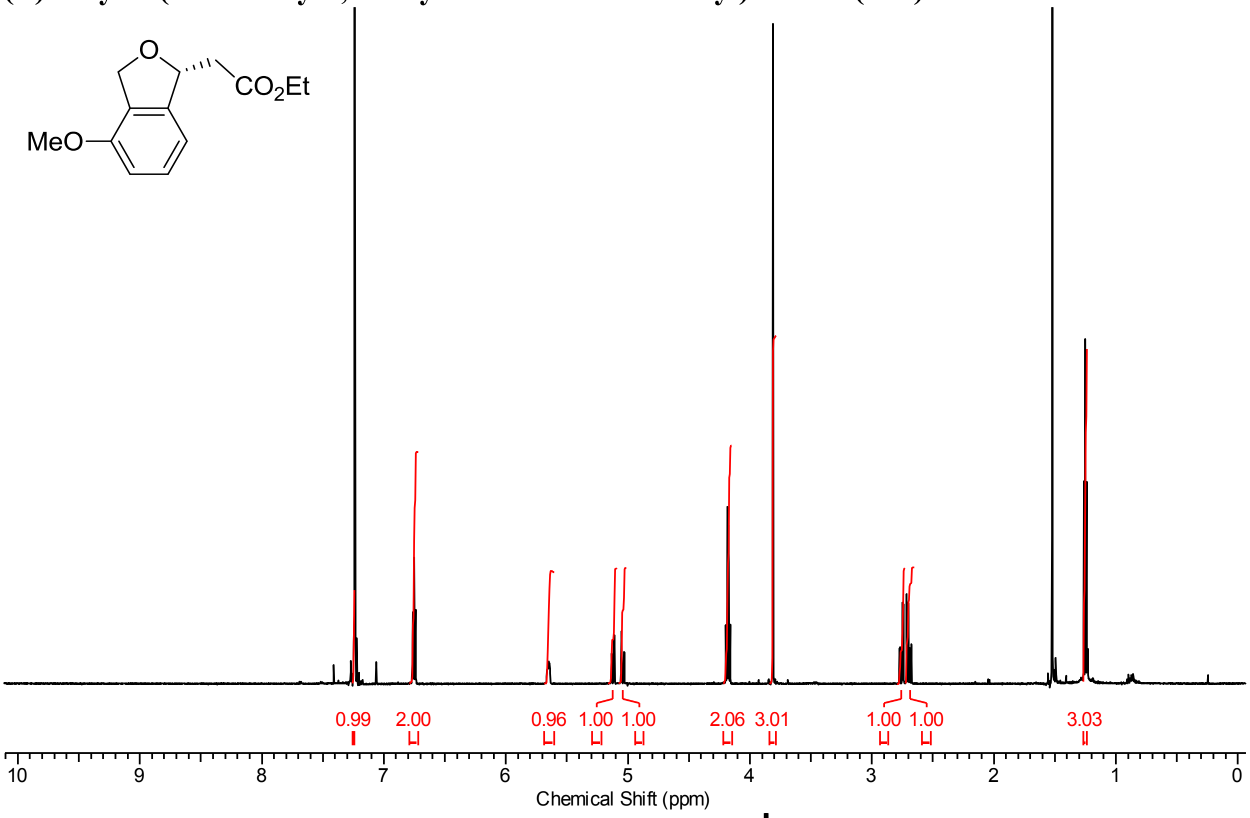

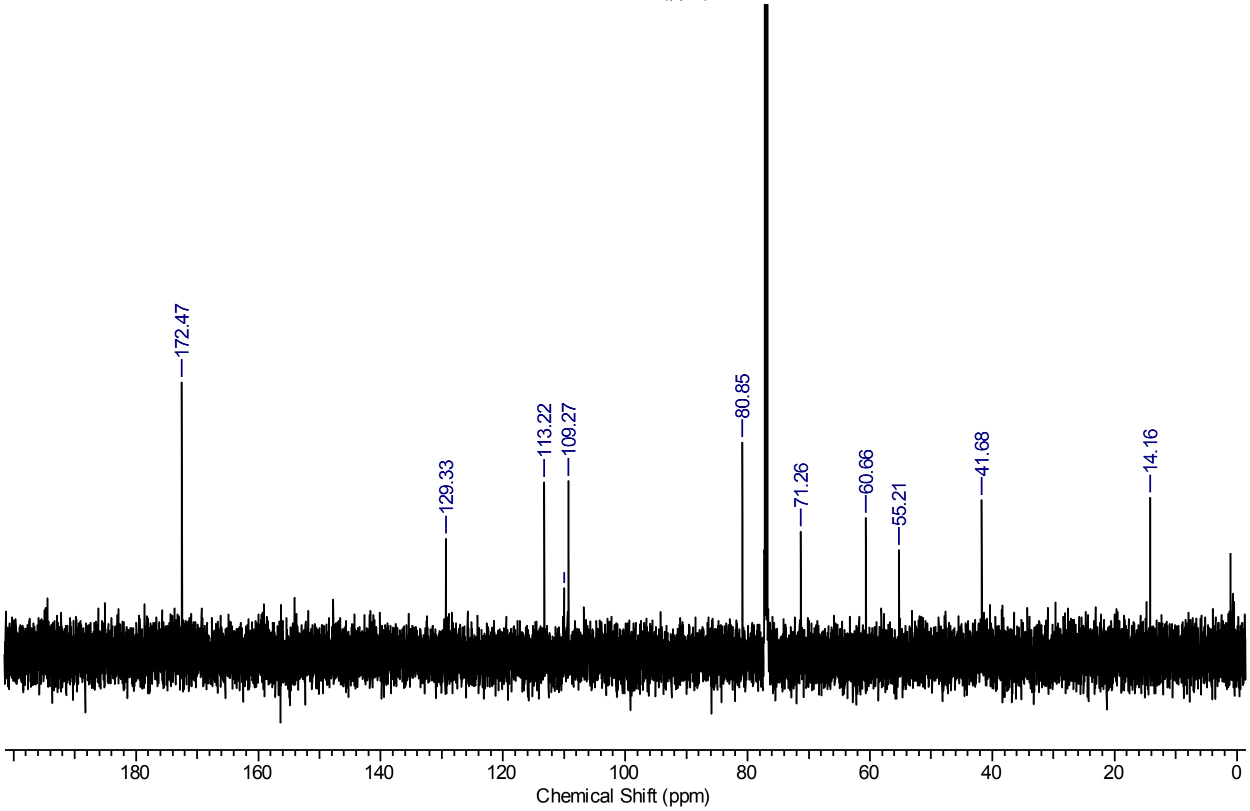

nOe spectra as evidence for **S54** as the drawn regioisomer:

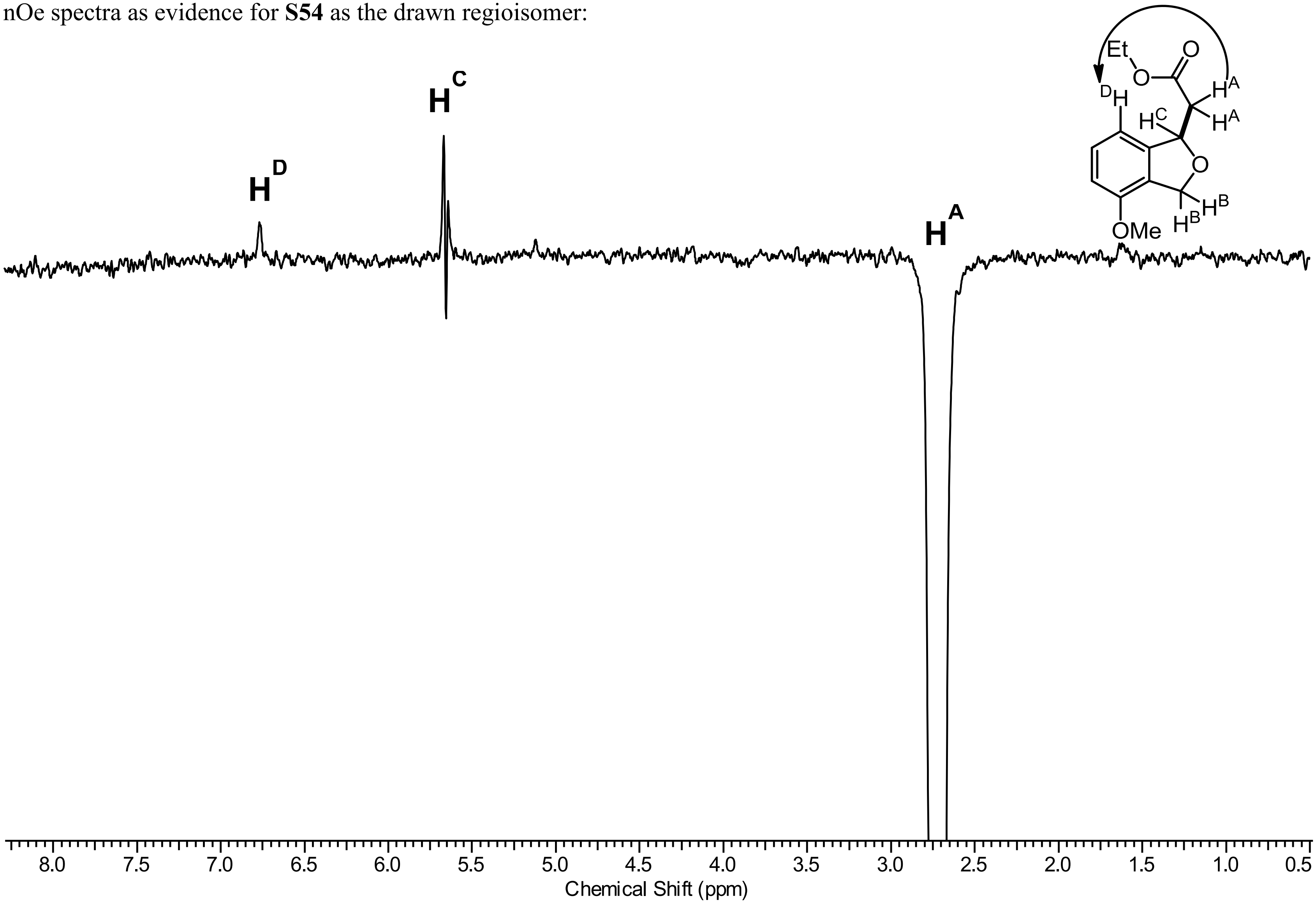

(*R*)-ethyl 2-(4-bromo-1,3-dihydroisobenzofuran-1-yl)acetate (18).

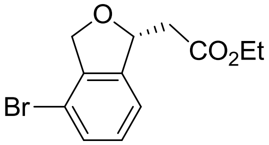

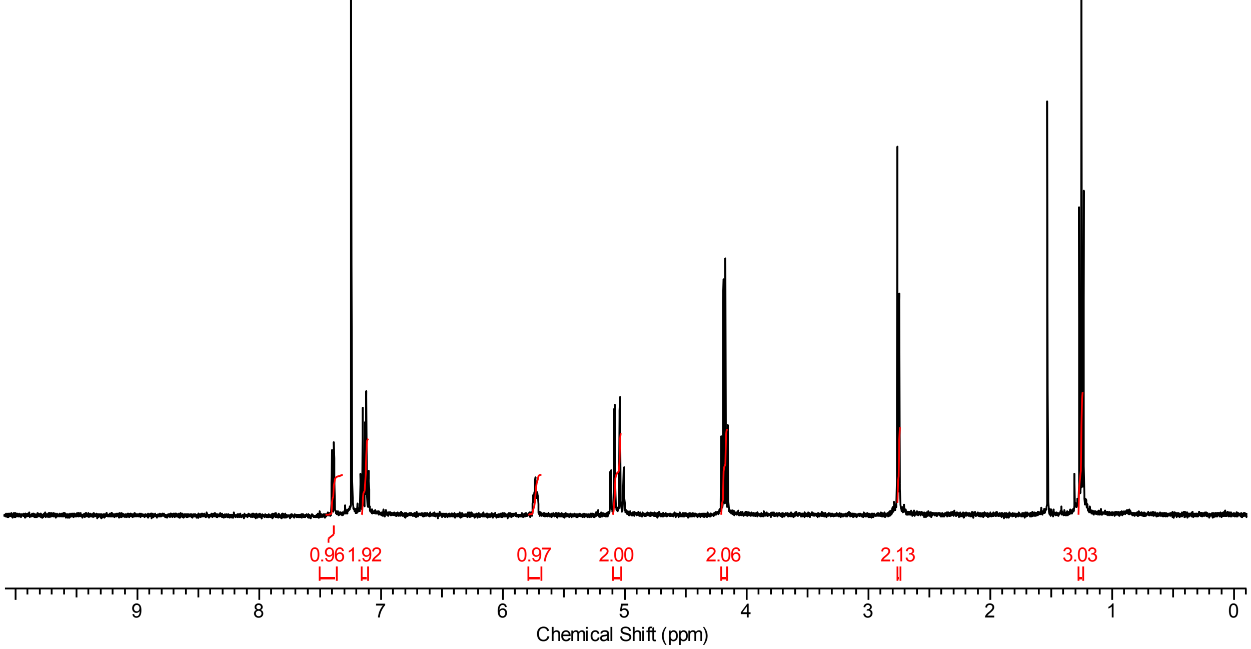

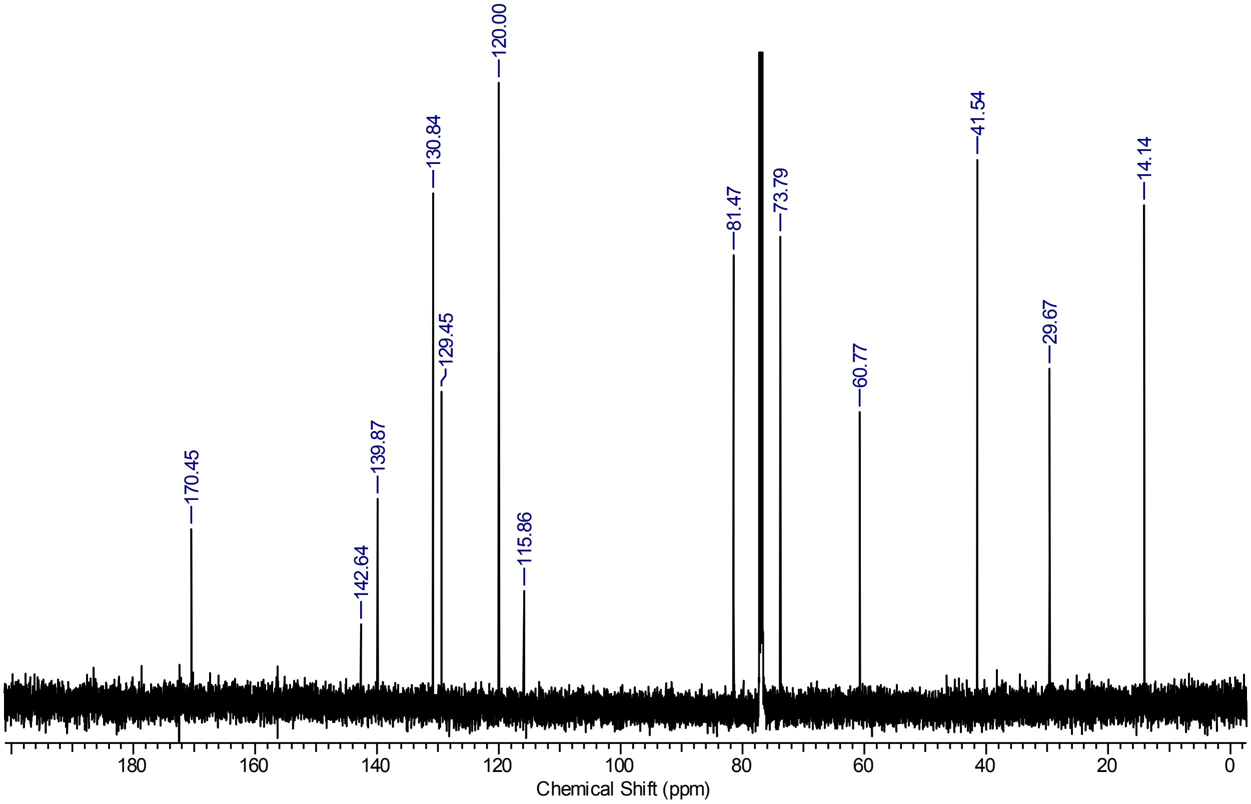

nOe spectra as evidence for **18** as the drawn regioisomer:

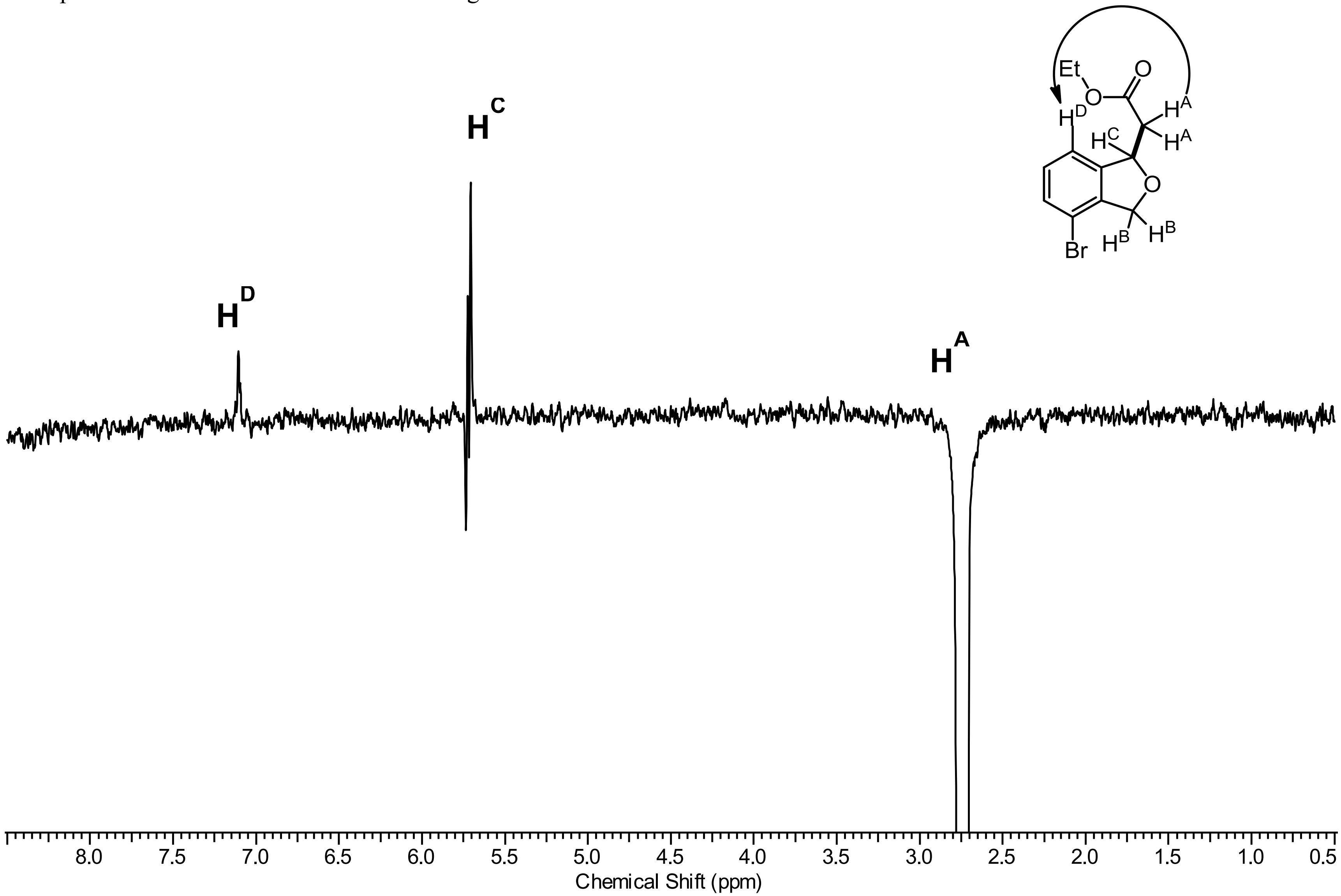

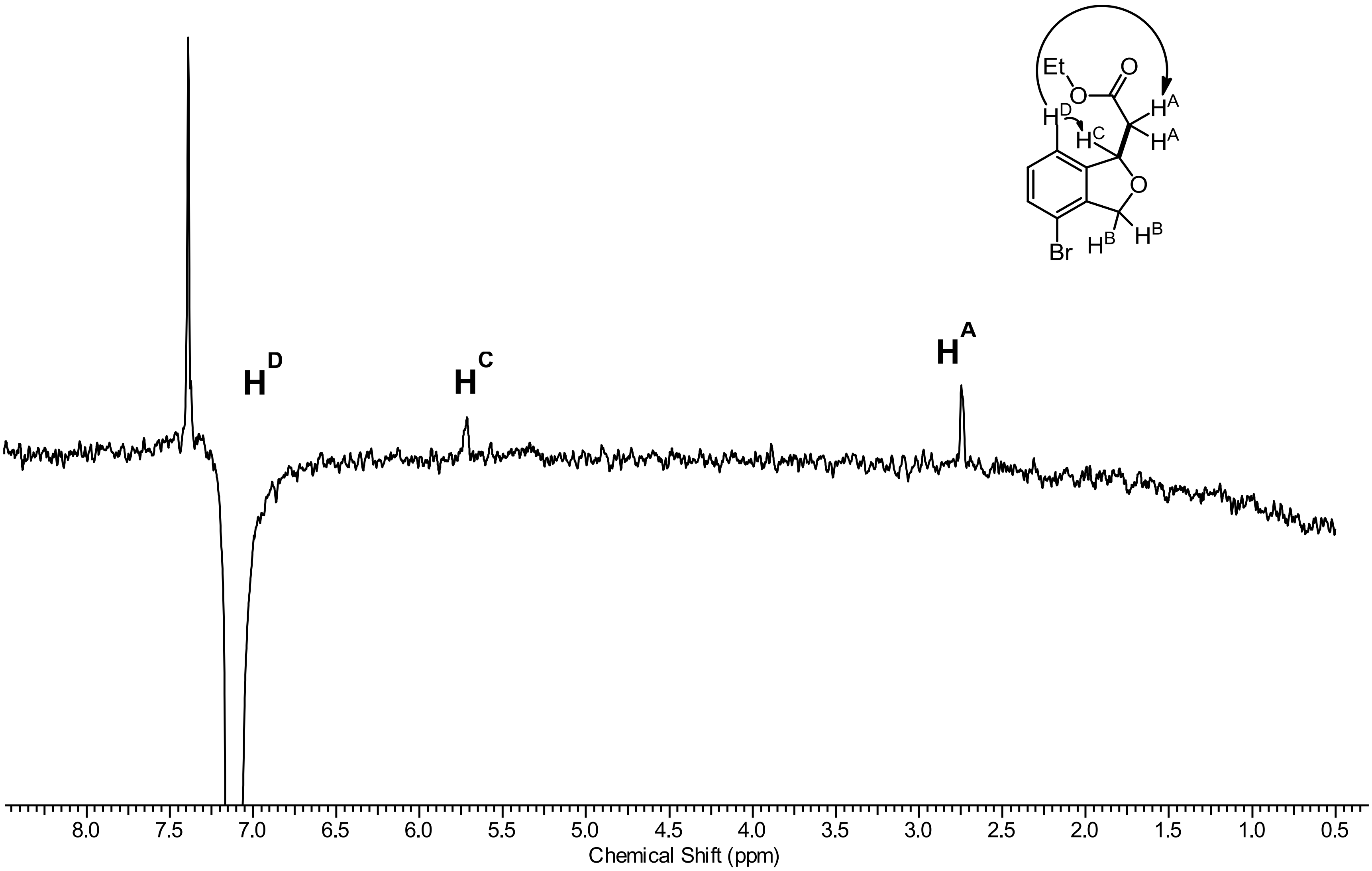

Et
O
O
H^A
H^D
H^C
H^A
O
Br
H^B
H^B
H^D
H^C
H^A
8.0
7.5
7.0
6.5
6.0
5.5
5.0
4.5
4.0
3.5
3.0
2.5
2.0
1.5
1.0
0.5
Chemical Shift (ppm)

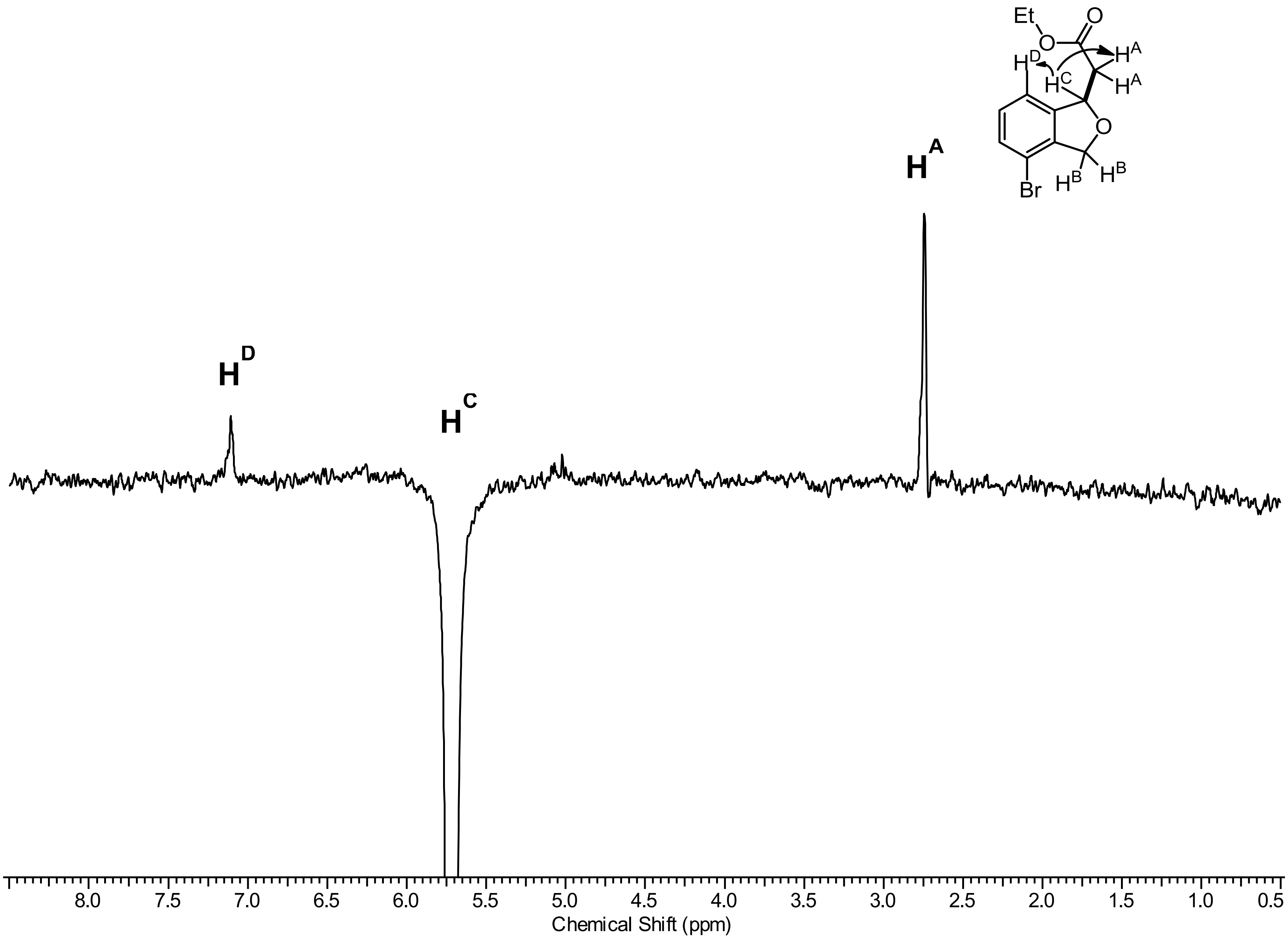
Et
O
O
H^D
H^C
H^A
H^A
O
Br
H^B
H^B
H^A
H^D
H^C
8.0
7.5
7.0
6.5
6.0
5.5
5.0
4.5
4.0
3.5
3.0
2.5
2.0
1.5
1.0
0.5
Chemical Shift (ppm)

(*R*)-ethyl 2-(7-bromo-1,3-dihydroisobenzofuran-1-yl)acetate (S55).

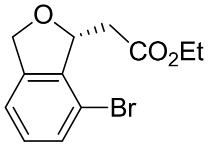

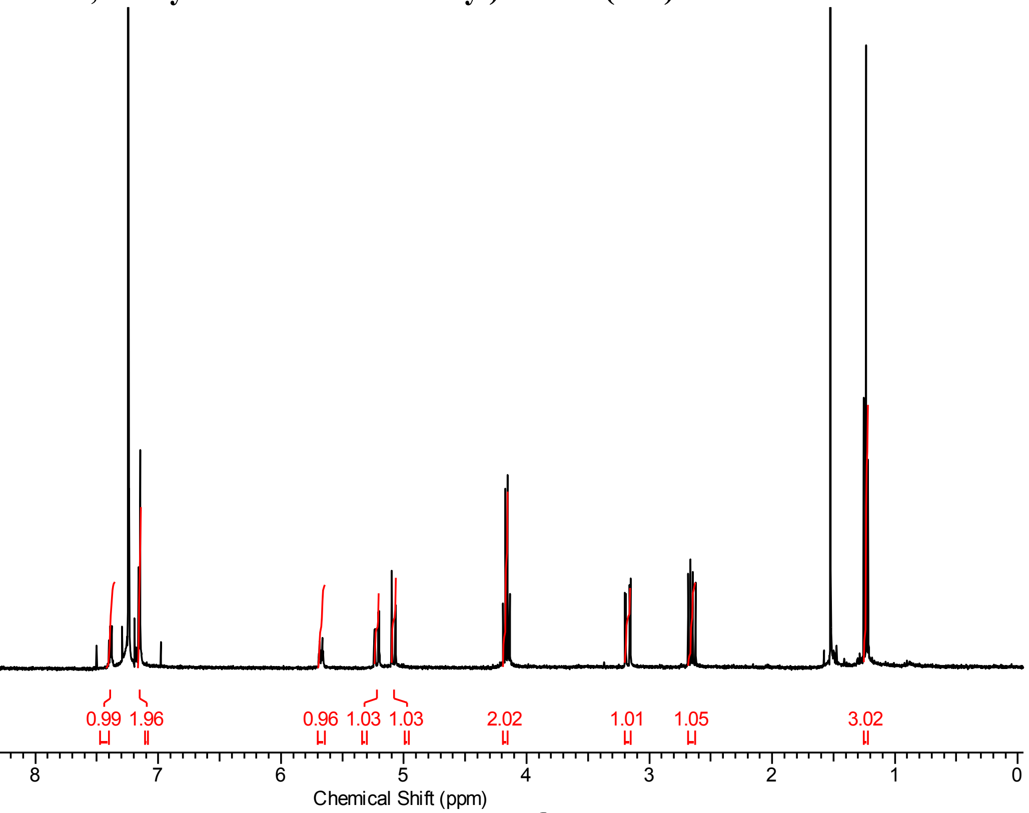

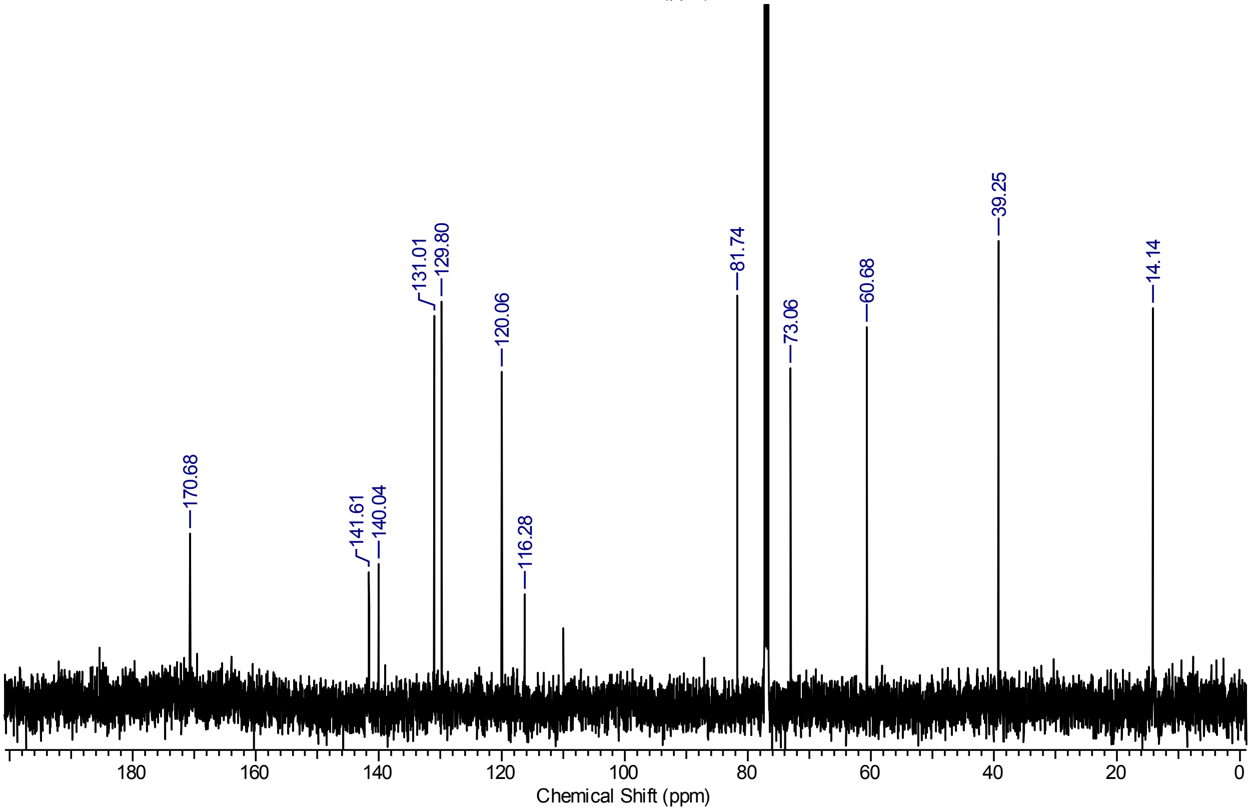

nOe spectra as evidence for **S55** as the drawn regioisomer:

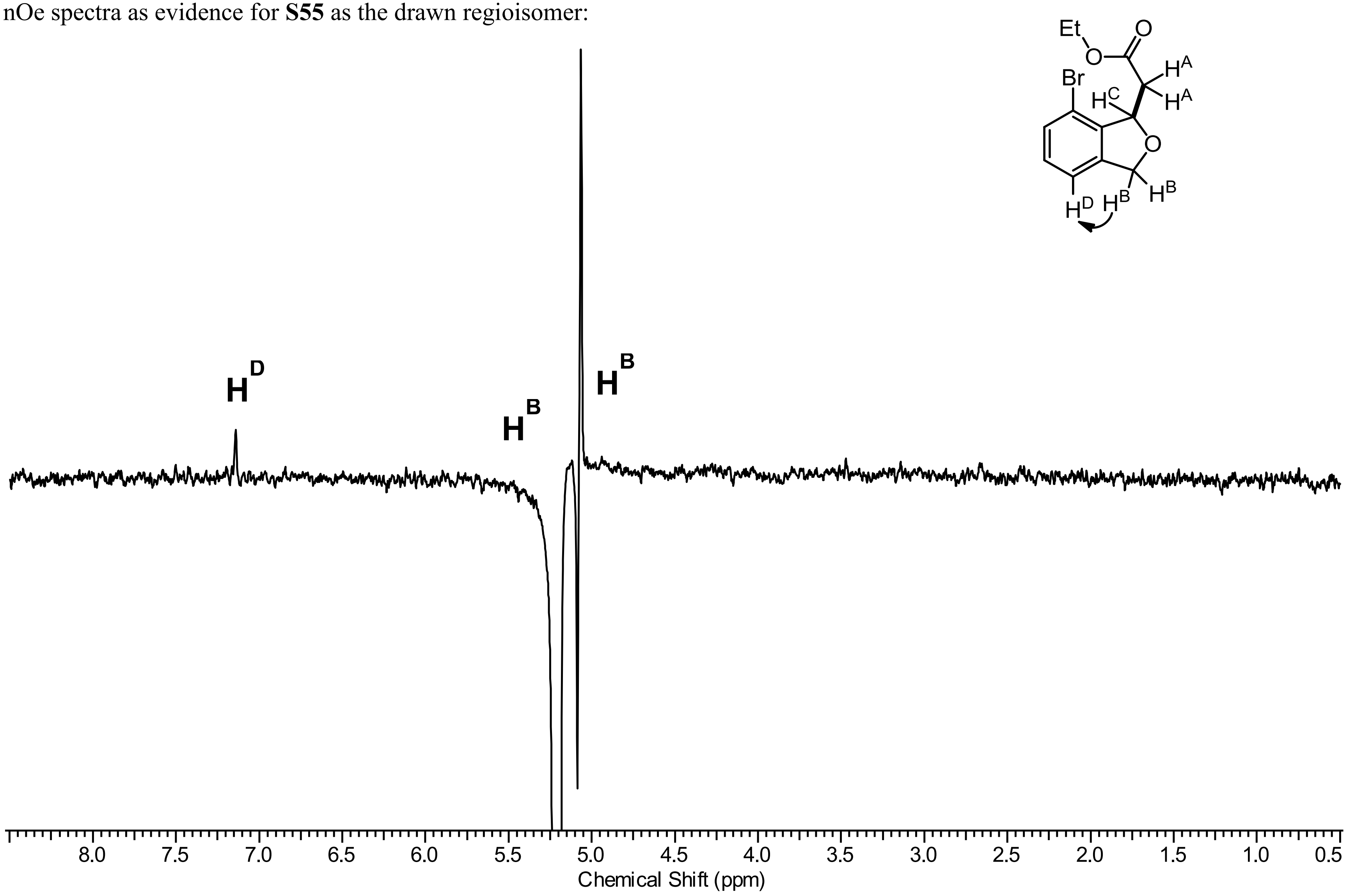

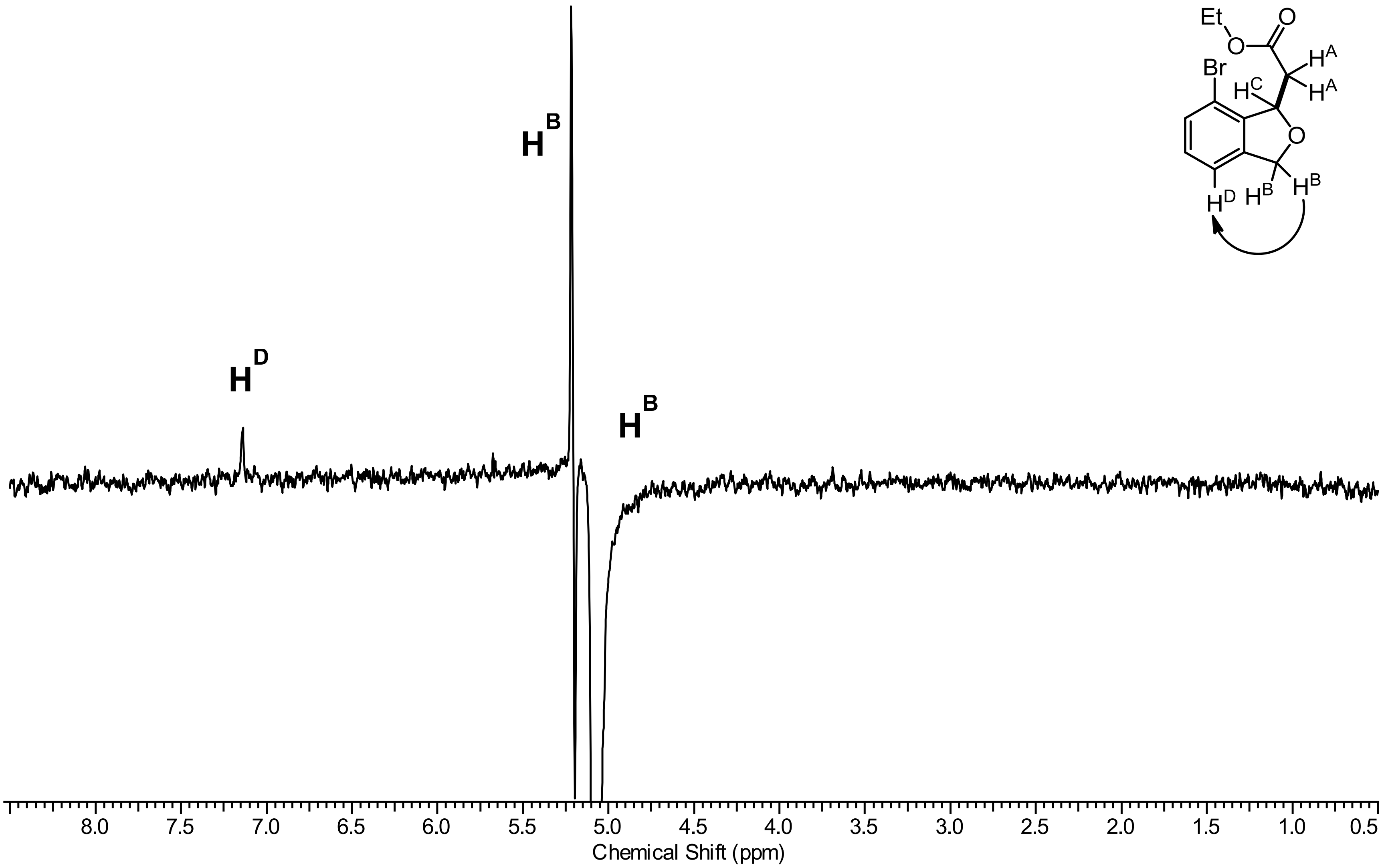
H^B
H^D
H^B
Et
O
O
H^A
Br
H^C
H^A
O
H^D
H^B
H^B
8.0
7.5
7.0
6.5
6.0
5.5
5.0
4.5
4.0
3.5
3.0
2.5
2.0
1.5
1.0
0.5
Chemical Shift (ppm)

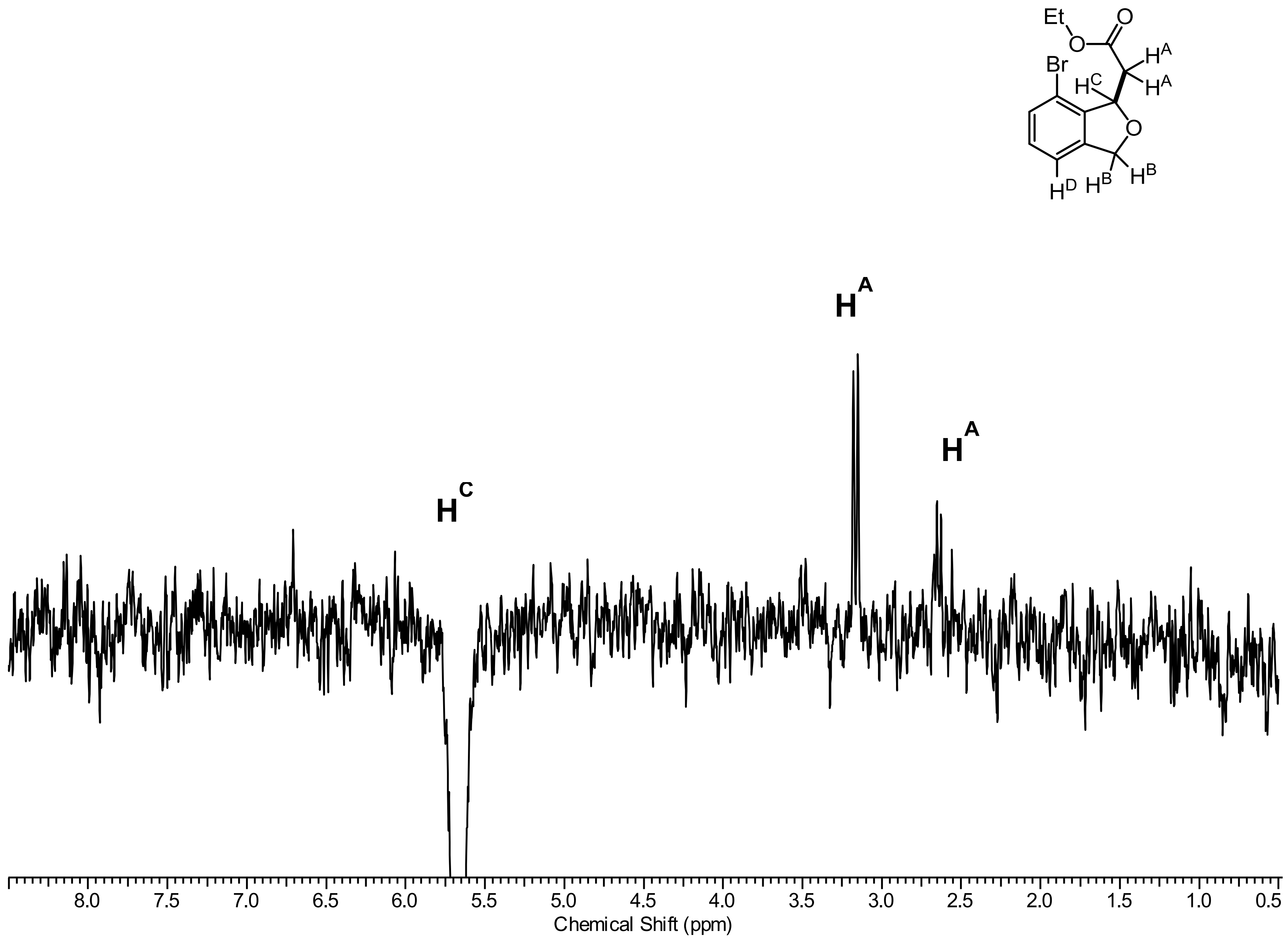
Et
O
O
Br
H^C
H^A
H^A
O
H^D
H^B
H^B
H^A
H^A
H^C
8.0 7.5 7.0 6.5 6.0 5.5 5.0 4.5 4.0 3.5 3.0 2.5 2.0 1.5 1.0 0.5
Chemical Shift (ppm)

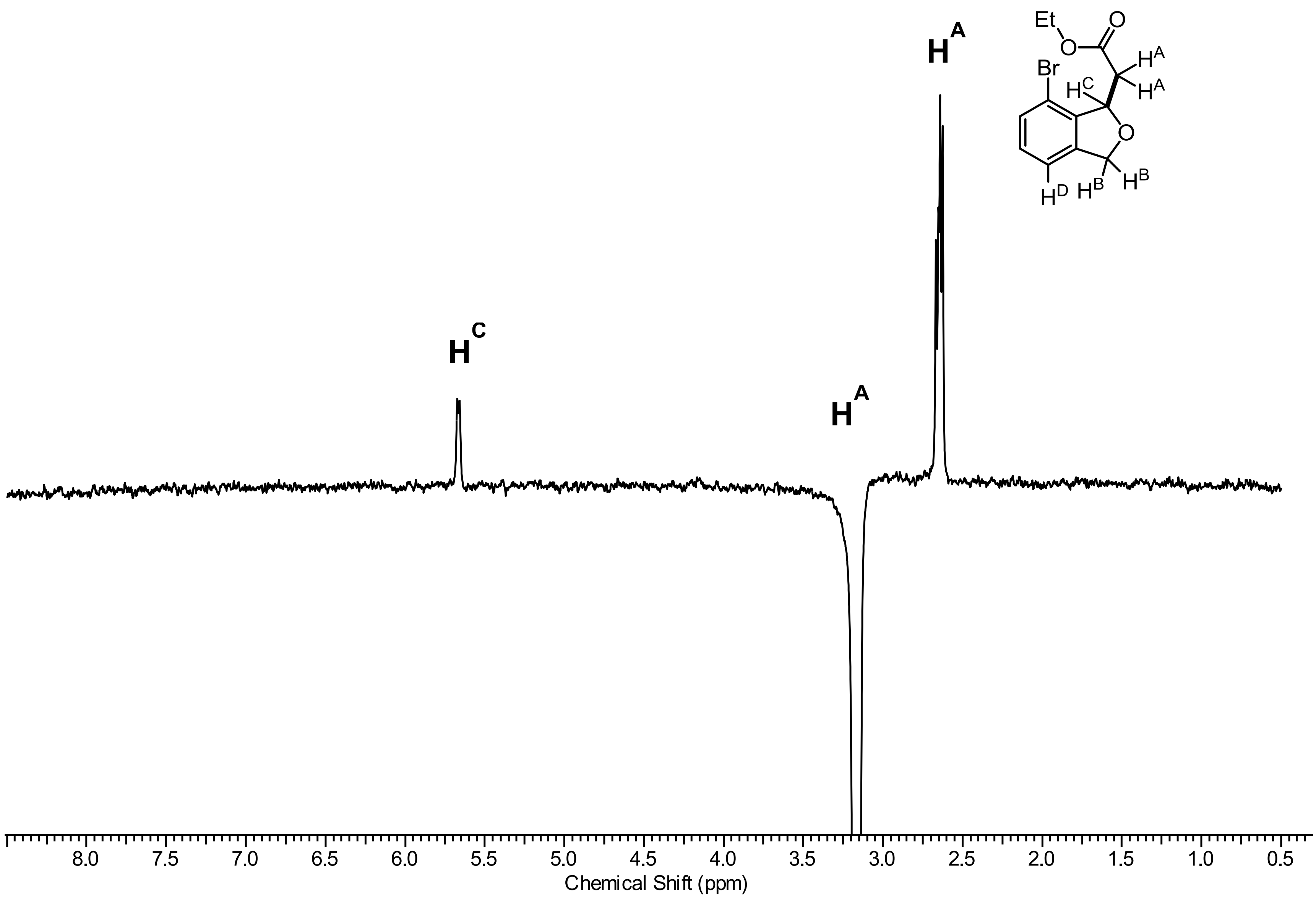
H^A
H^C
H^A
Et
O
O
Br
H^A
H^C
H^A
O
H^D
H^B
H^B
8.0
7.5
7.0
6.5
6.0
5.5
5.0
4.5
4.0
3.5
3.0
2.5
2.0
1.5
1.0
0.5
Chemical Shift (ppm)

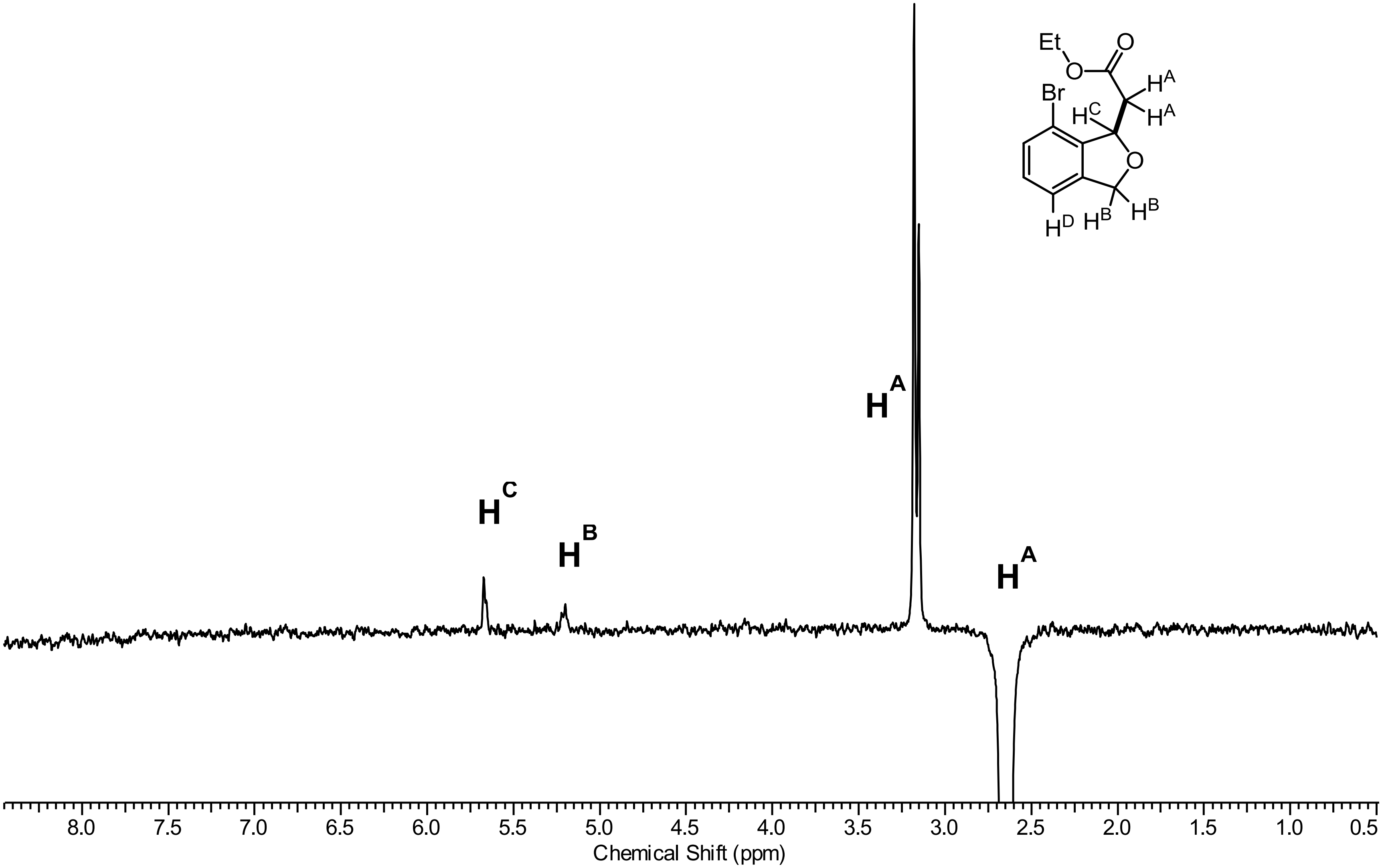

Et
O
O
Br
H^C
H^A
H^A
O
H^D
H^B
H^B
H^A
H^C
H^B
H^A
8.0
7.5
7.0
6.5
6.0
5.5
5.0
4.5
4.0
3.5
3.0
2.5
2.0
1.5
1.0
0.5
Chemical Shift (ppm)

(*R*)-ethyl 2-(4-phenyl-1,3-dihydroisobenzofuran-1-yl)acetate (20).

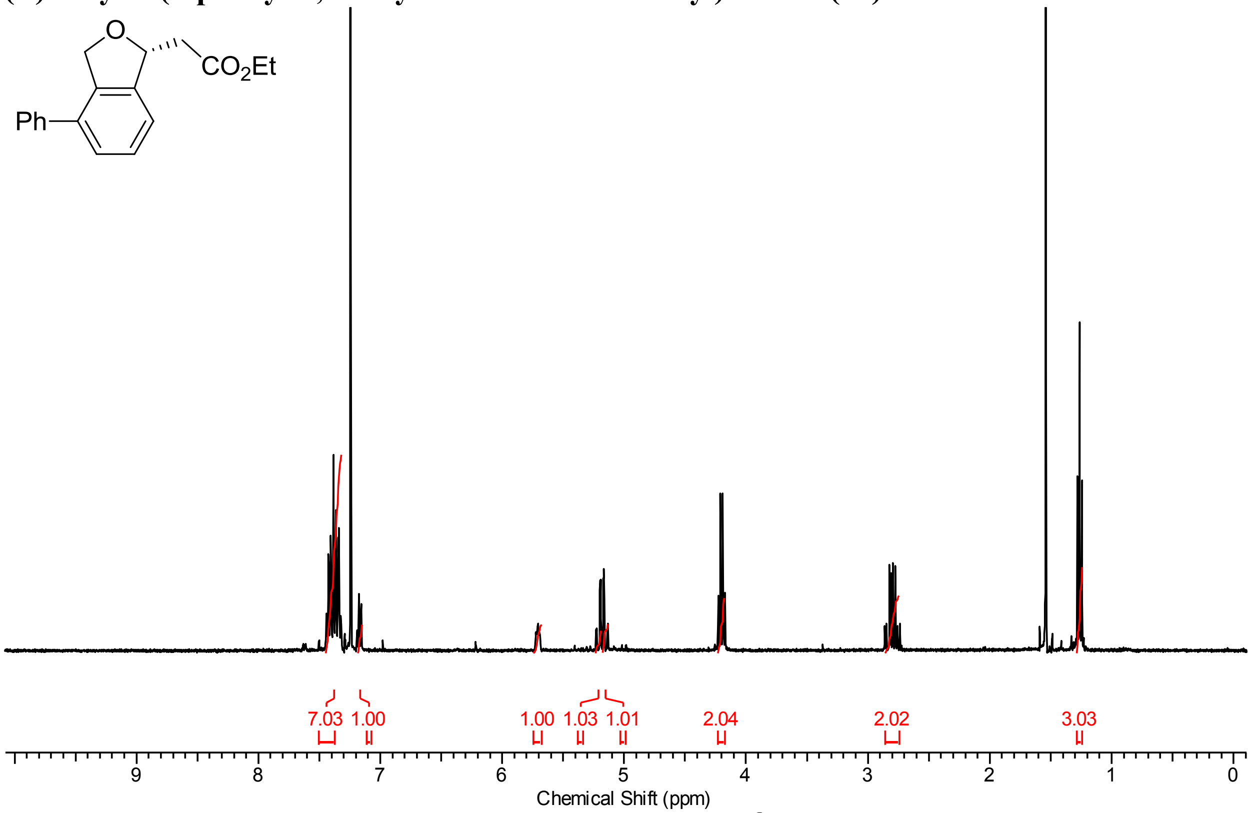

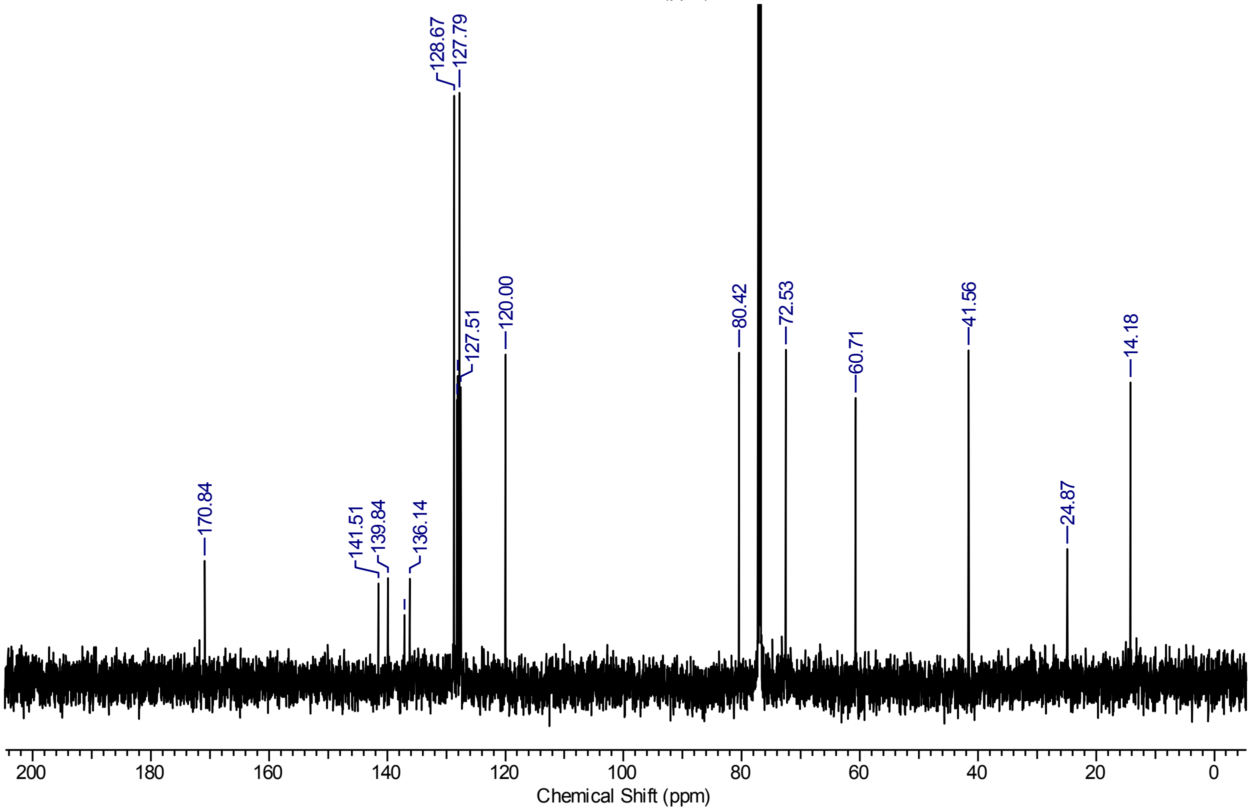

nOe spectra as evidence for **20** as the drawn regioisomer

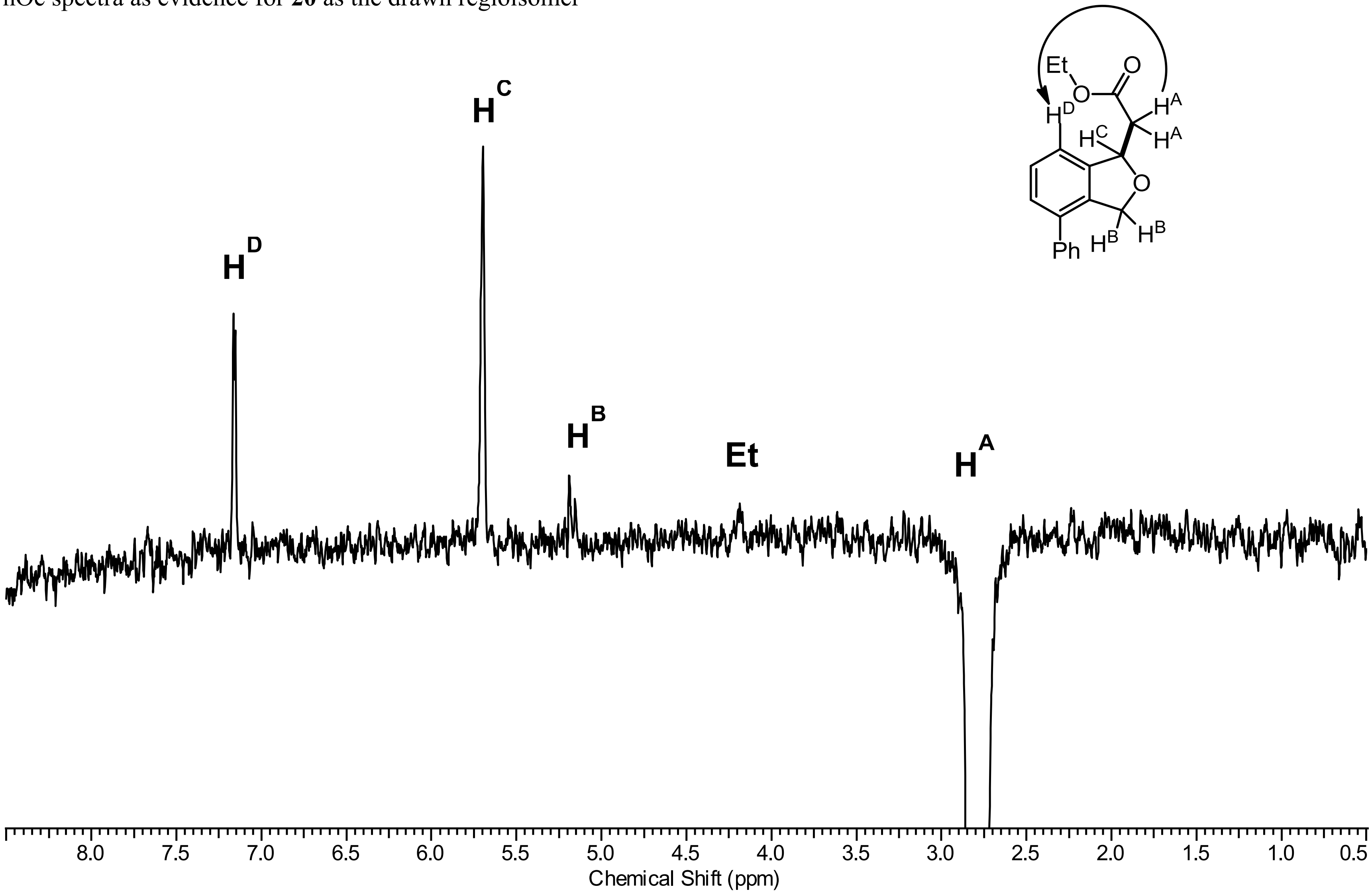

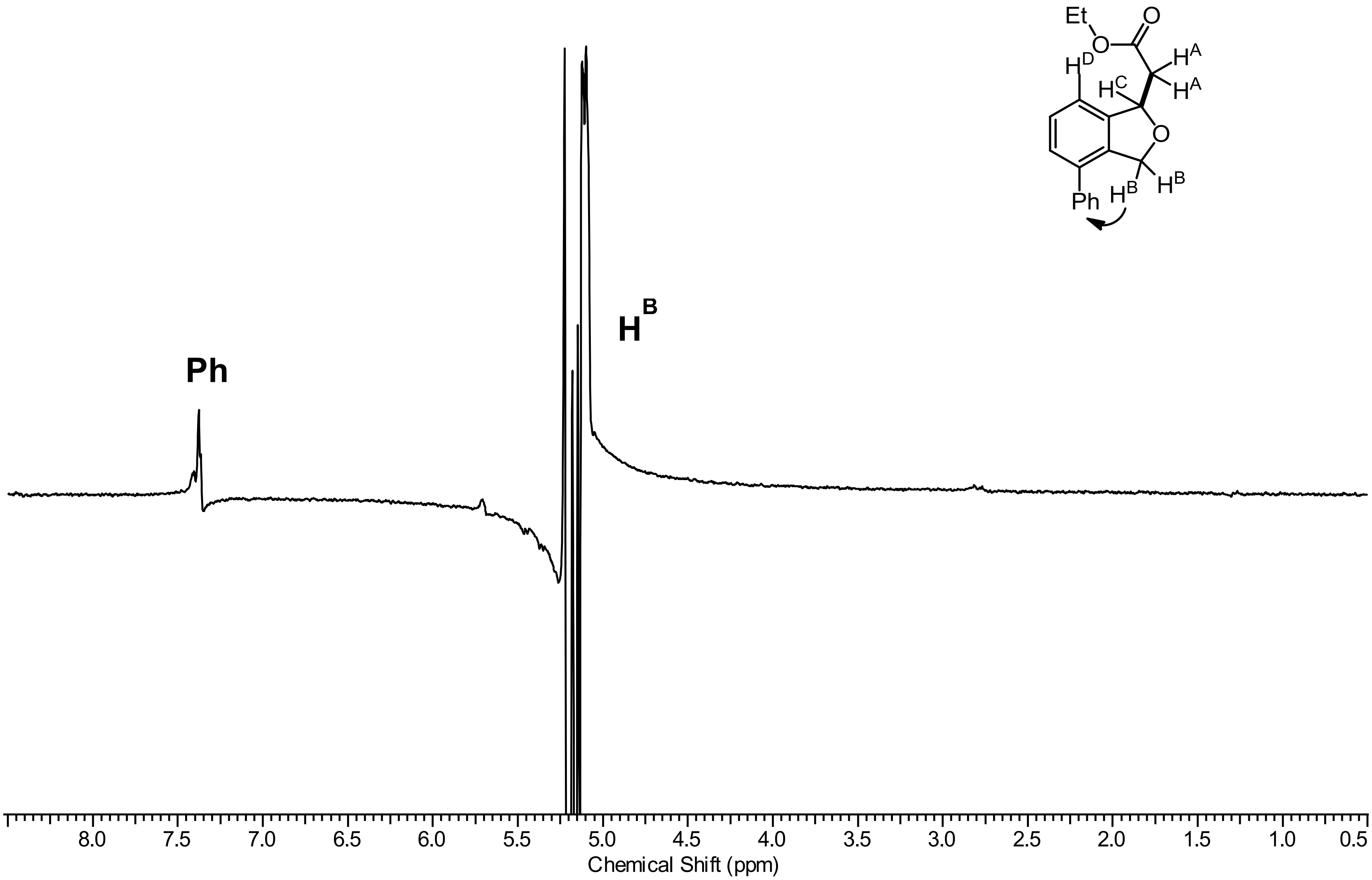

Et
O
O
H^D
H^A
H^C
H^A
O
H^B
H^B
Ph
H^B
Ph
8.0
7.5
7.0
6.5
6.0
5.5
5.0
4.5
4.0
3.5
3.0
2.5
2.0
1.5
1.0
0.5
Chemical Shift (ppm)

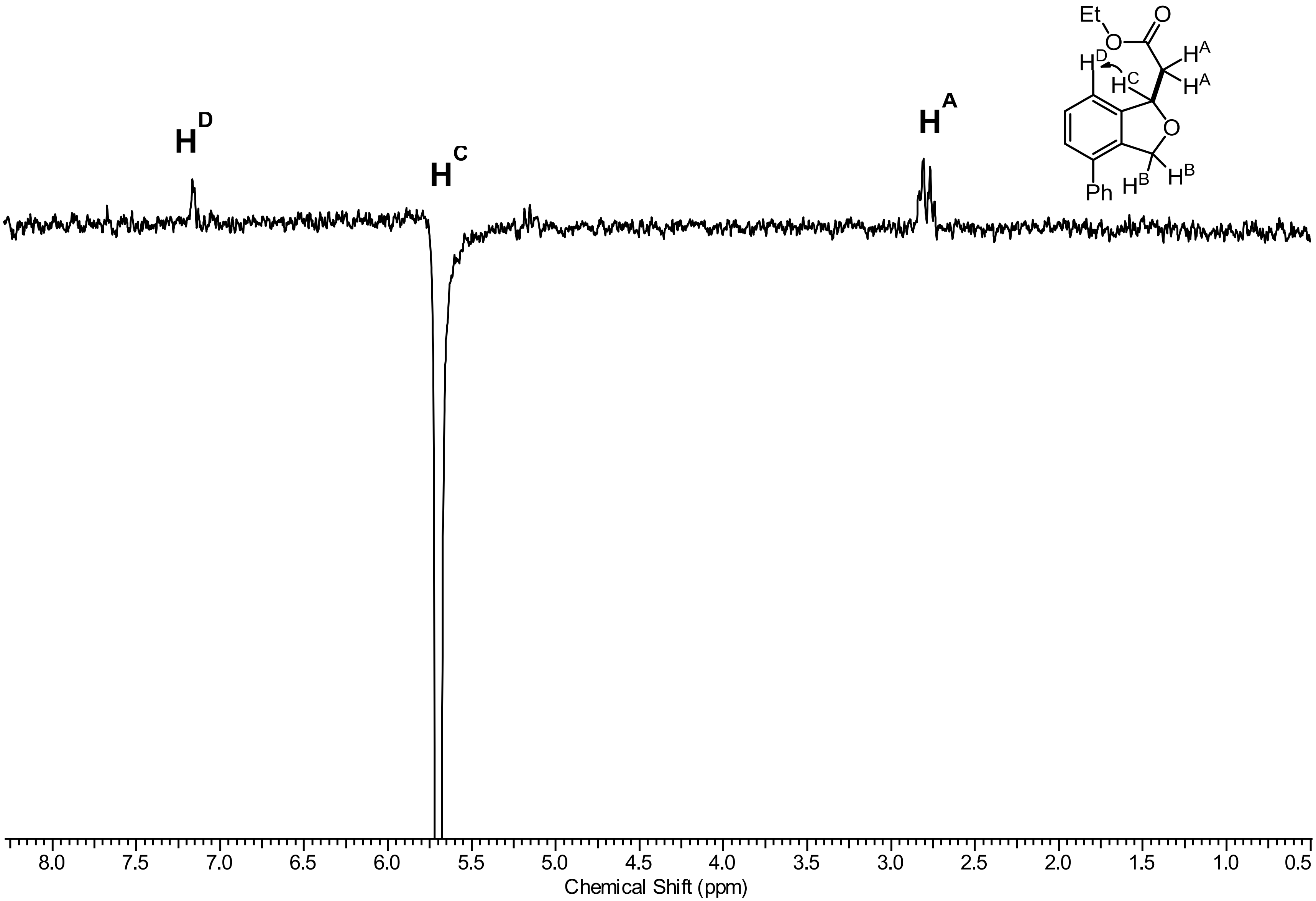

H^D
H^C
H^A
Et
O
O
H^D
H^C
H^A
H^A
O
H^B
H^B
Ph
8.0 7.5 7.0 6.5 6.0 5.5 5.0 4.5 4.0 3.5 3.0 2.5 2.0 1.5 1.0 0.5
Chemical Shift (ppm)

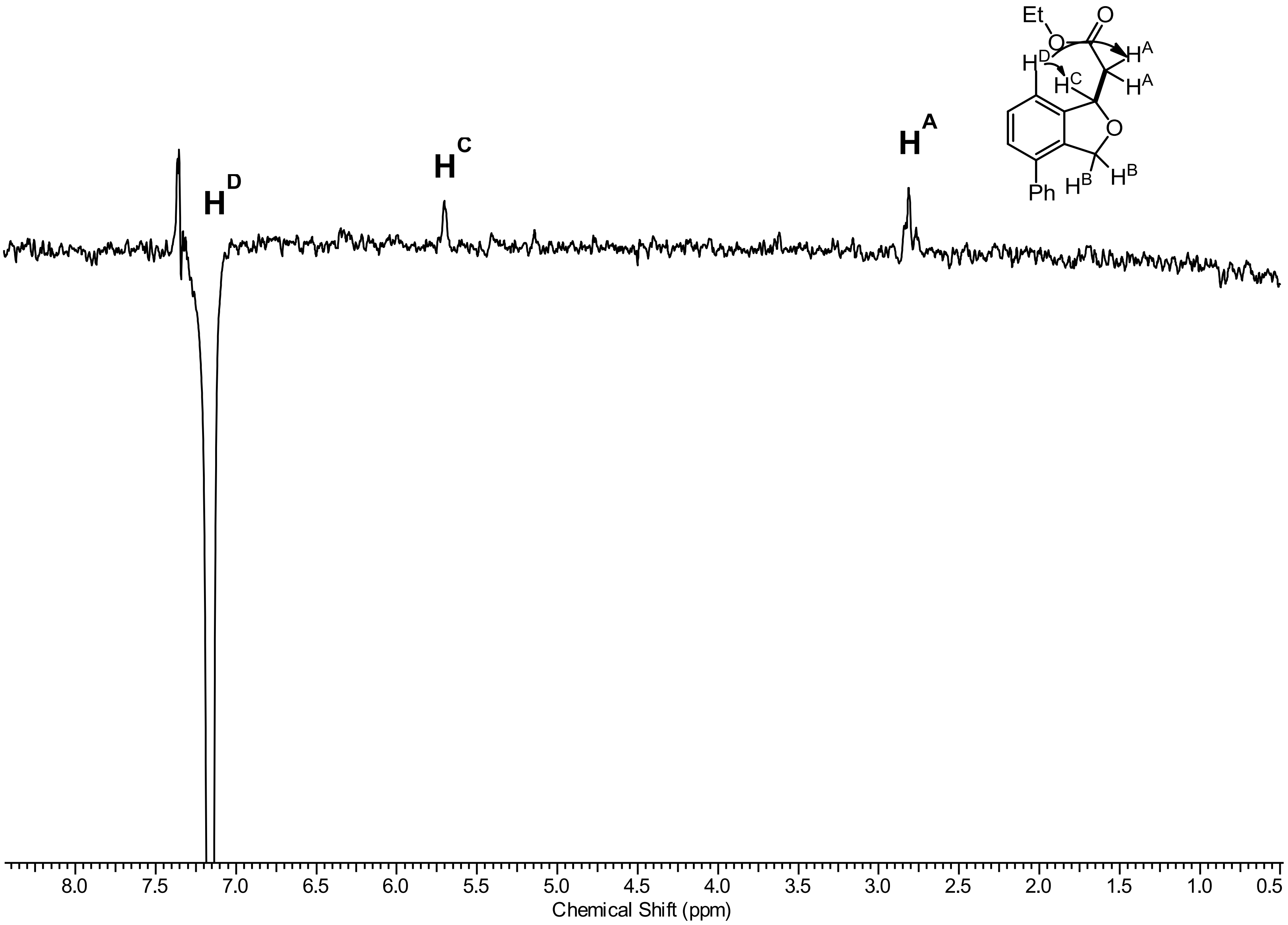

H^D
H^C
H^A
Et
O
O
H^D
H^C
H^A
H^A
O
Ph
H^B
H^B
8.0 7.5 7.0 6.5 6.0 5.5 5.0 4.5 4.0 3.5 3.0 2.5 2.0 1.5 1.0 0.5
Chemical Shift (ppm)

(*R*)-ethyl 2-(4-(pyrrolidin-1-yl)-1,3-dihydroisobenzofuran-1-yl)acetate (21).

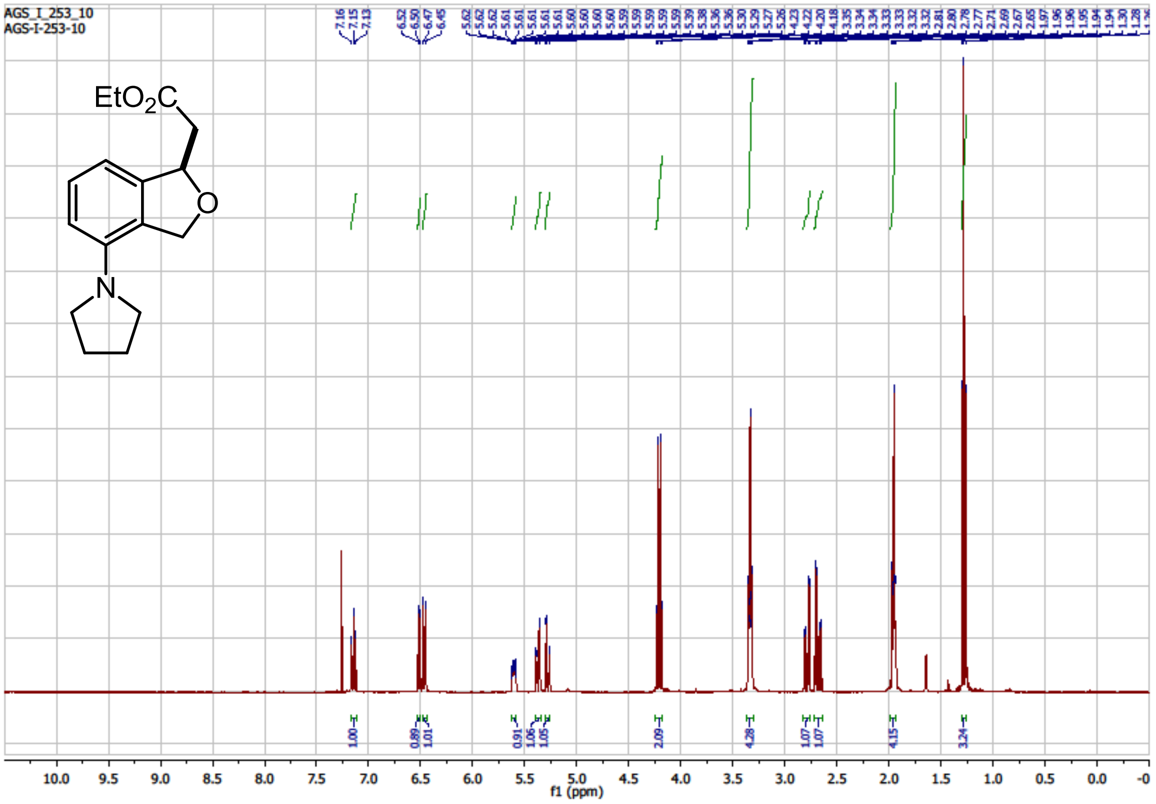

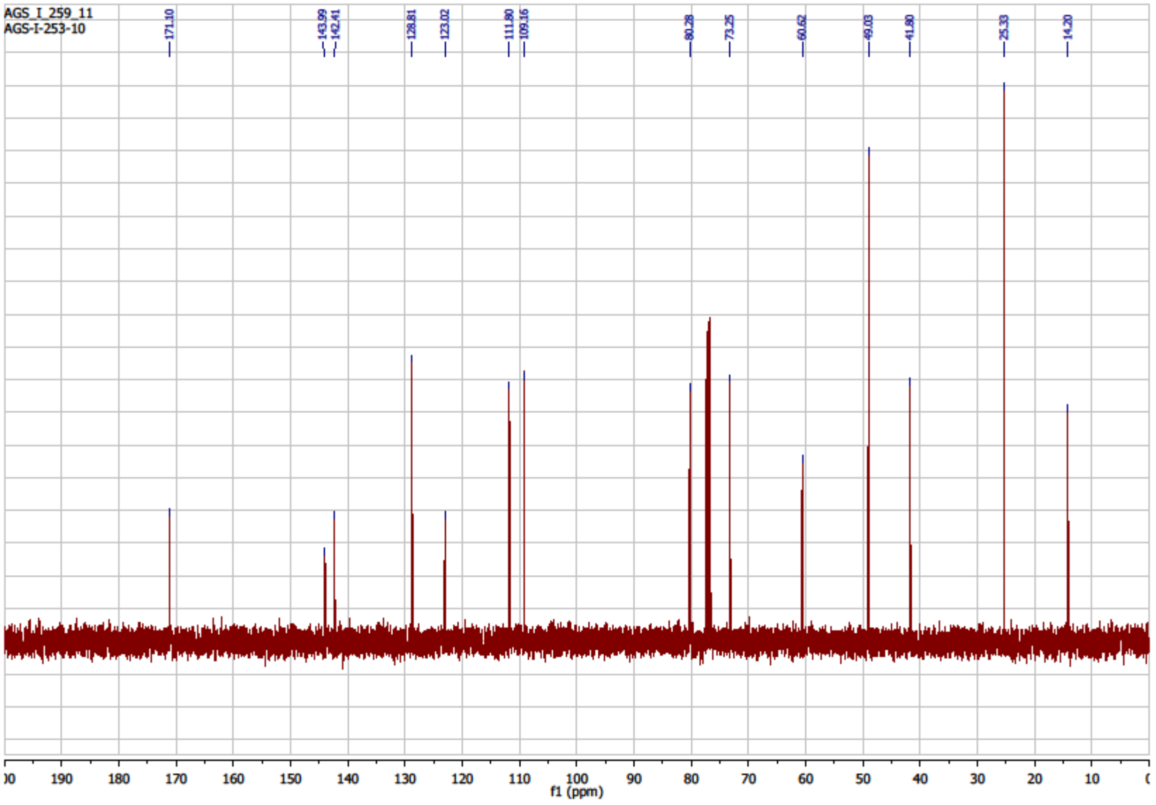

nOe spectra as evidence for **21** as the drawn regioisomer:

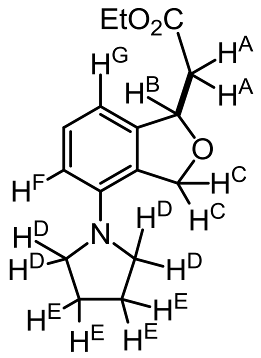

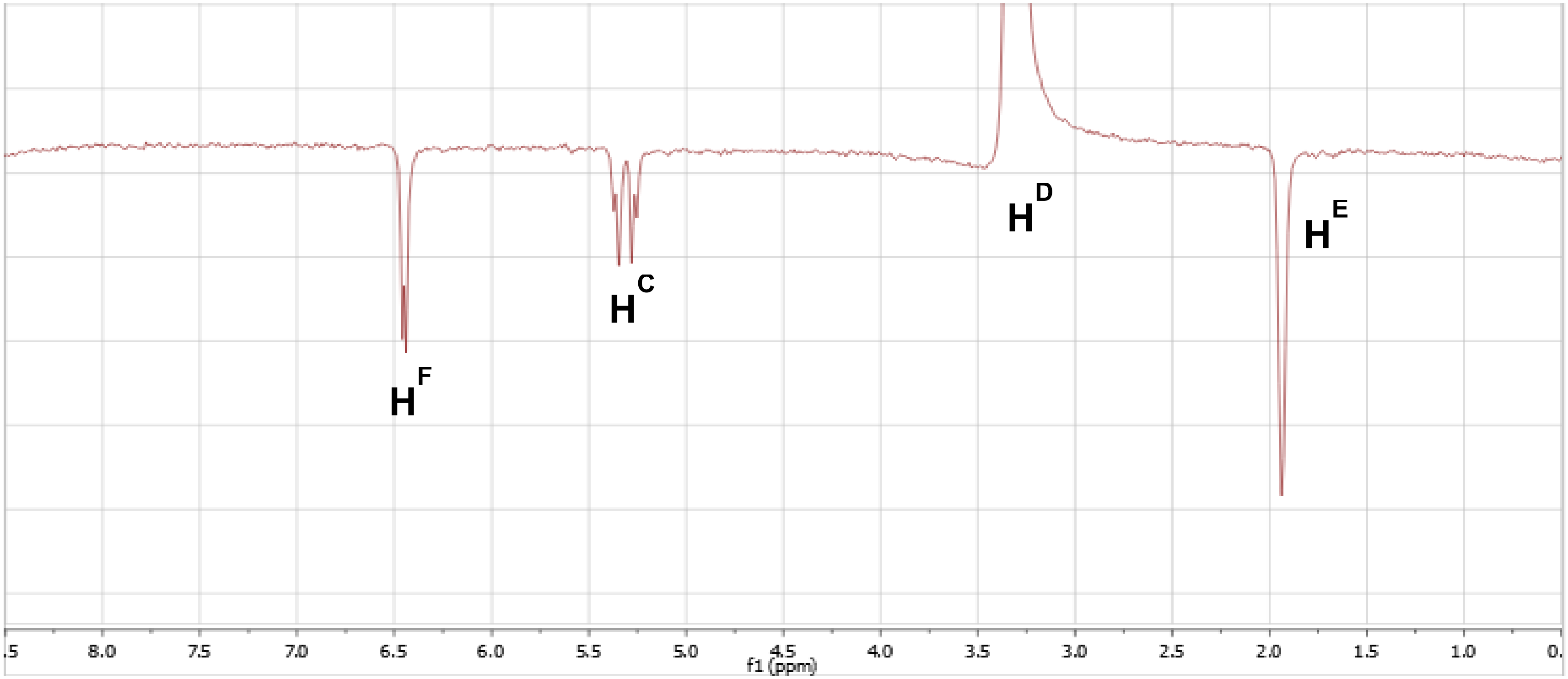

nOe spectra as evidence for **21** as the drawn regioisomer:

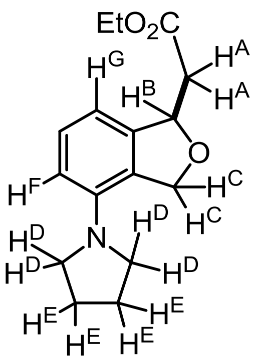

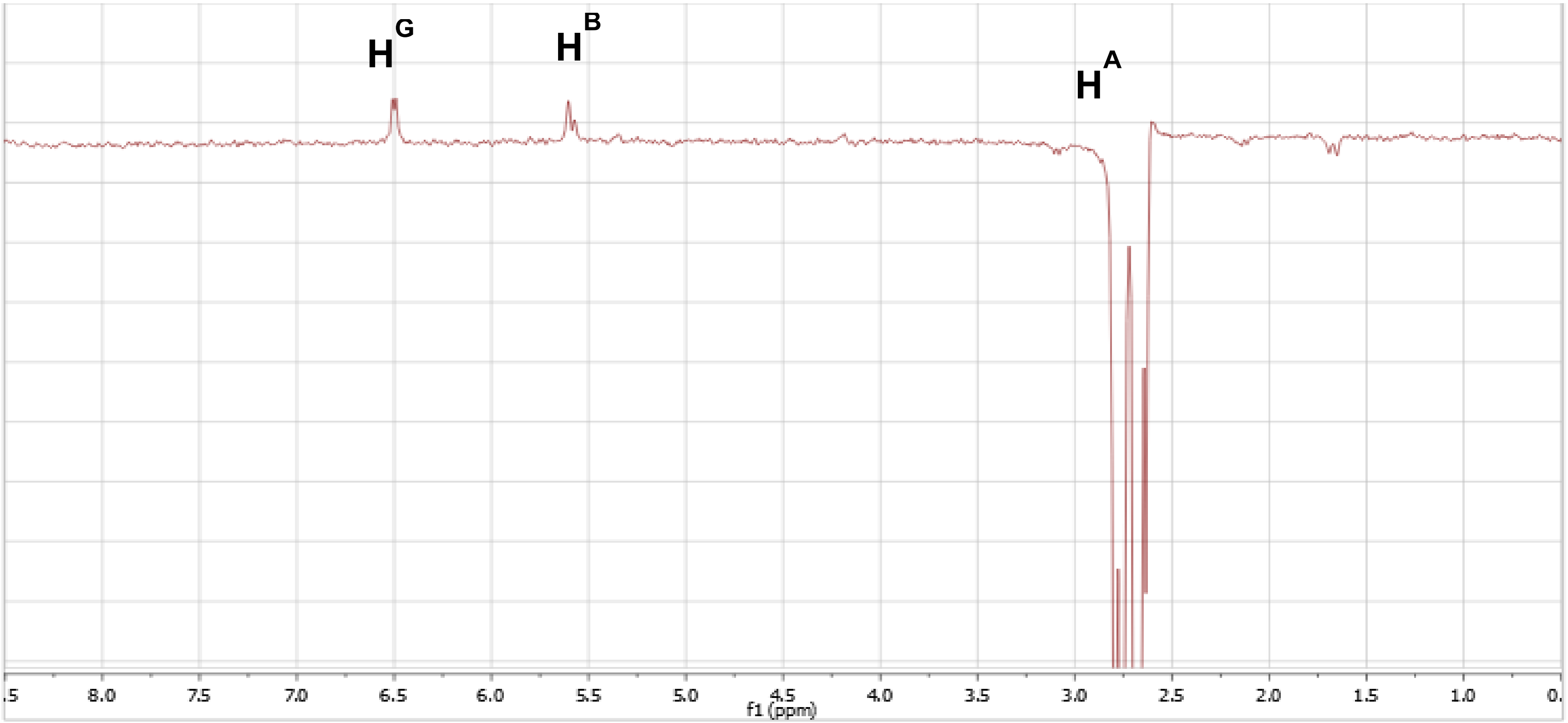

(*R*)-ethyl 2-(5-(pyrrolidin-1-yl)-1,3-dihydroisobenzofuran-1-yl)acetate, mix of regioisomers (22).

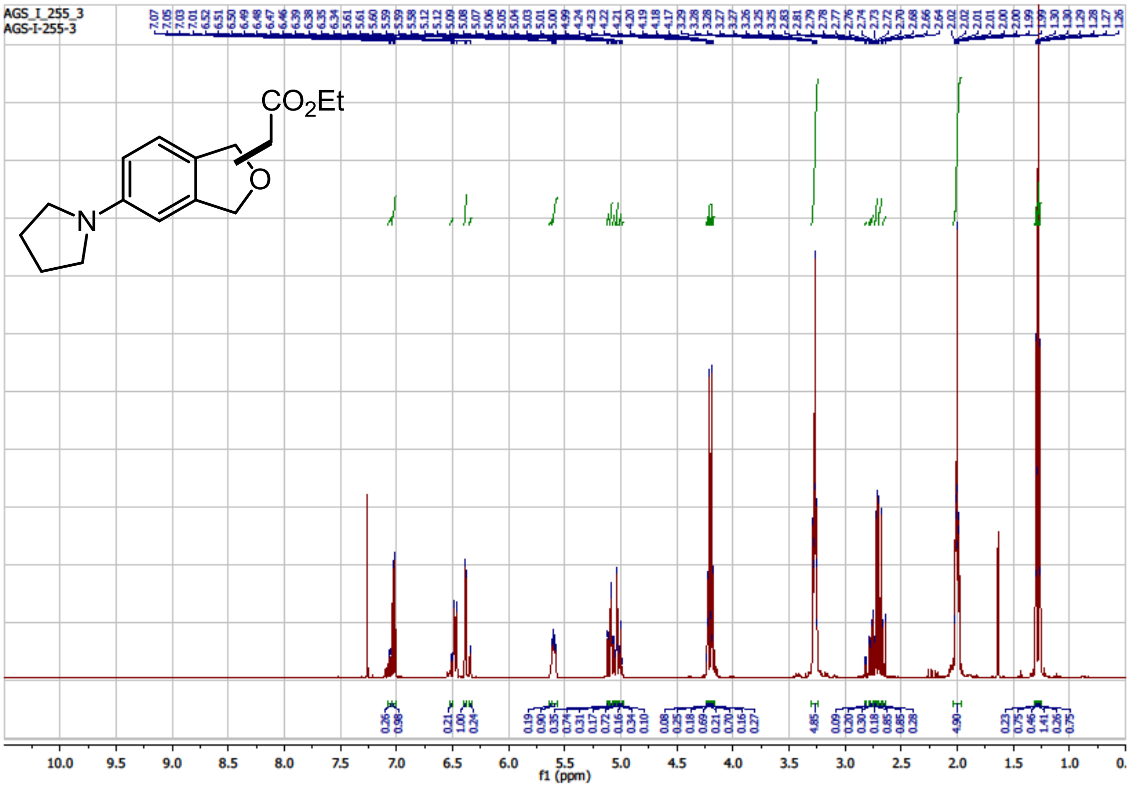

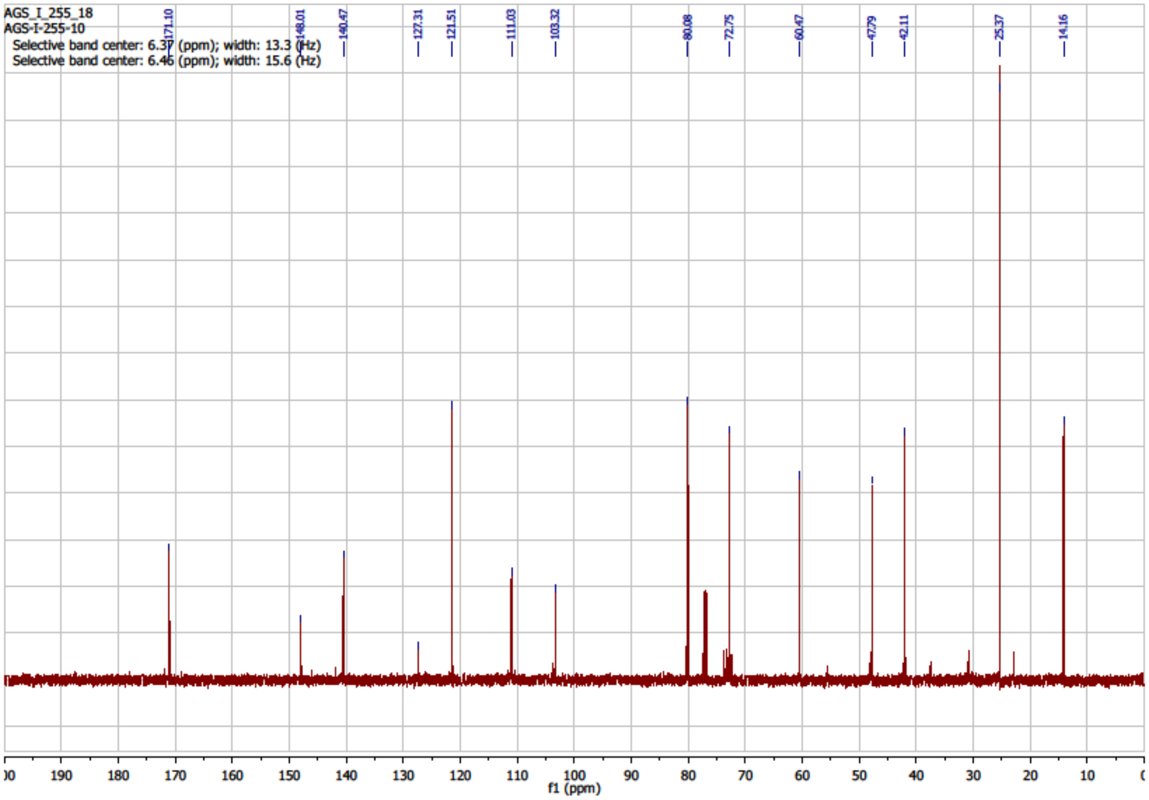

(*R*)-1-(1-(2-((*tert*-butyldiphenylsilyl)oxy)ethyl)-1,3-dihydroisobenzofuran-5-yl)pyrrolidine

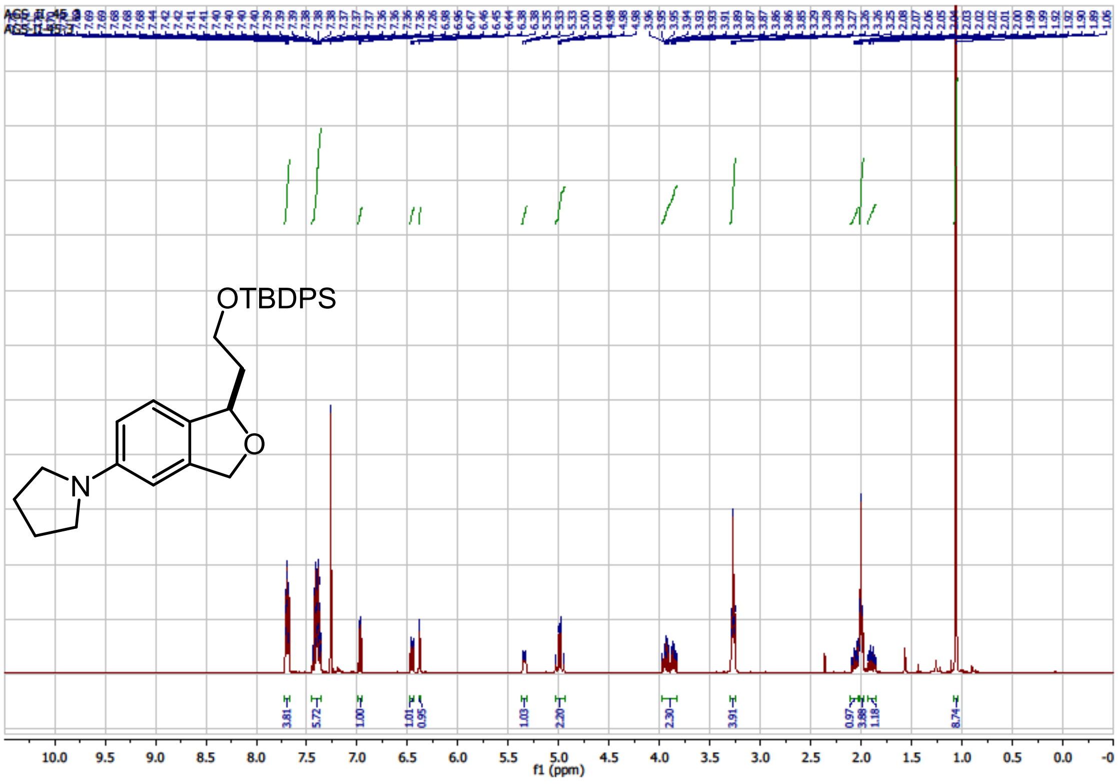

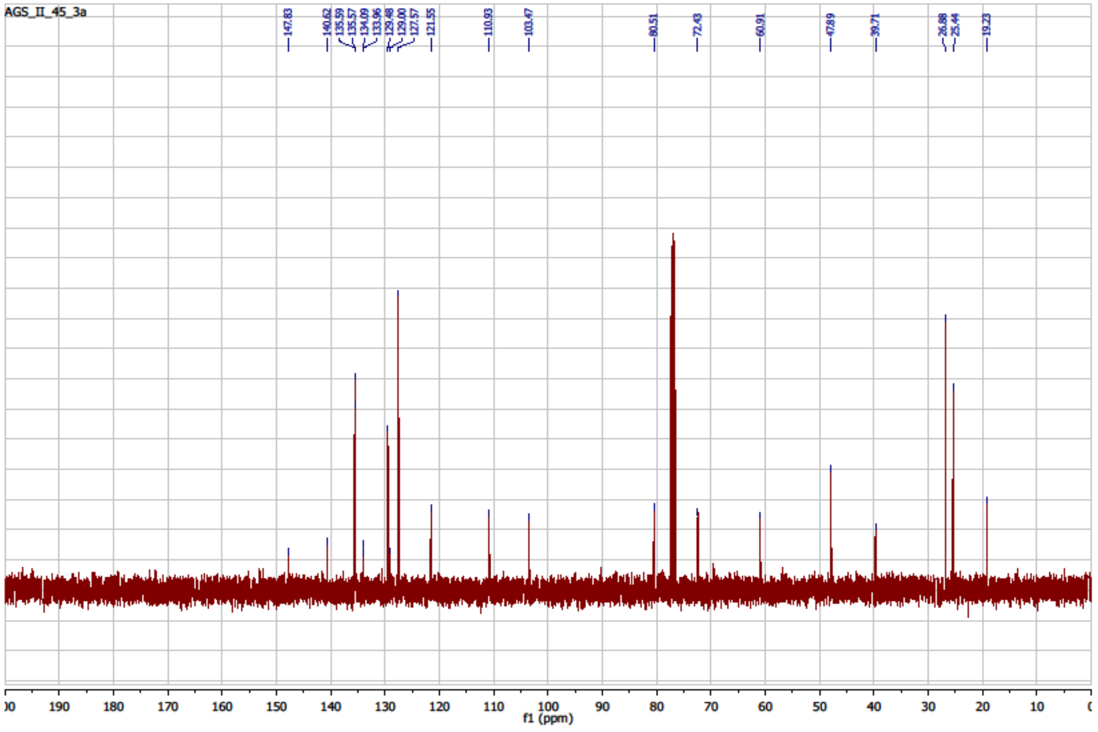

(*R*)-1-(3-(2-((*tert*-butyldiphenylsilyl)oxy)ethyl)-1,3-dihydroisobenzofuran-5-yl)pyrrolidine

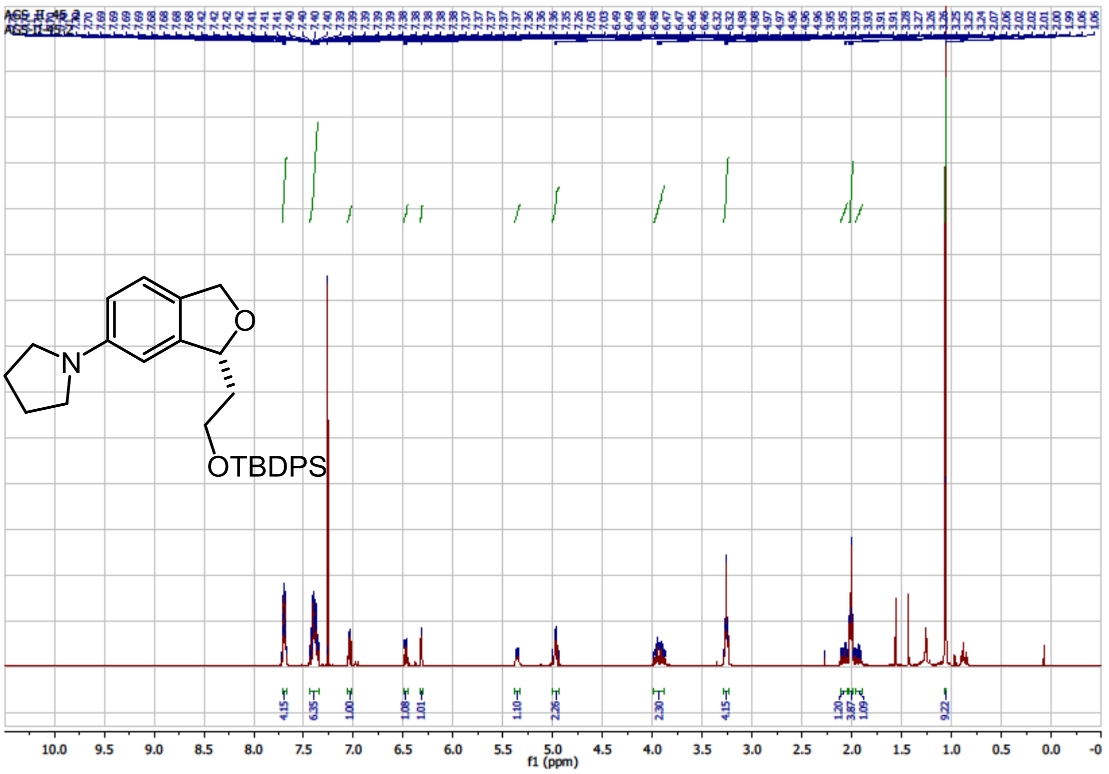

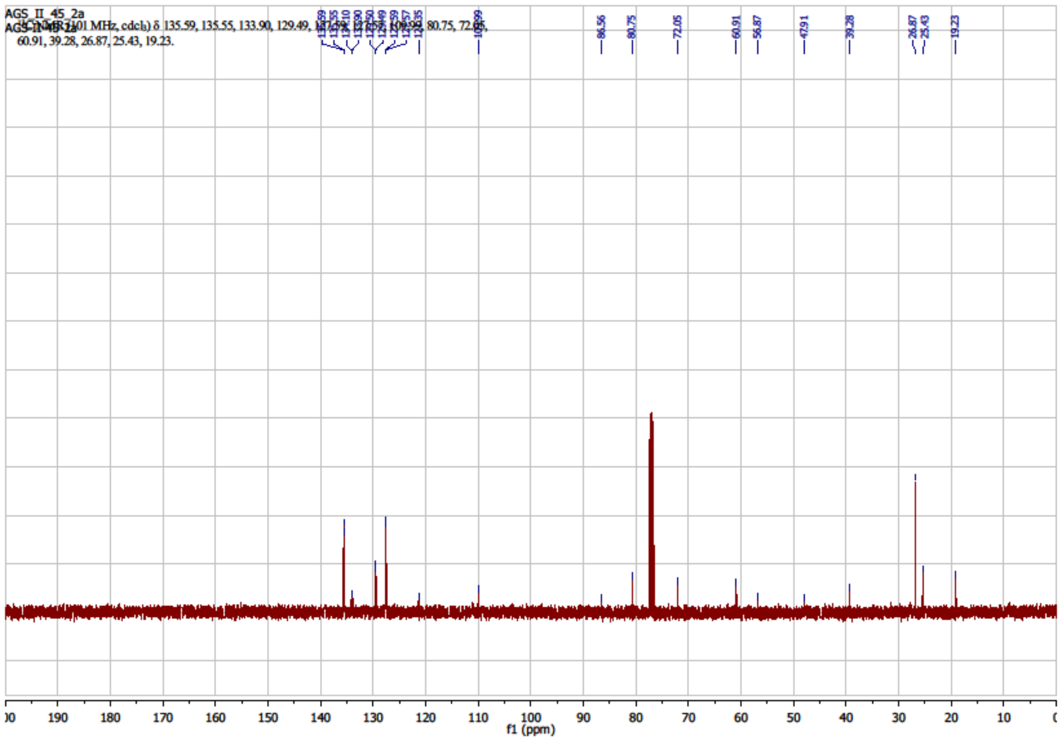

nOe spectrum as evidence for **22** as the major regioisomer:

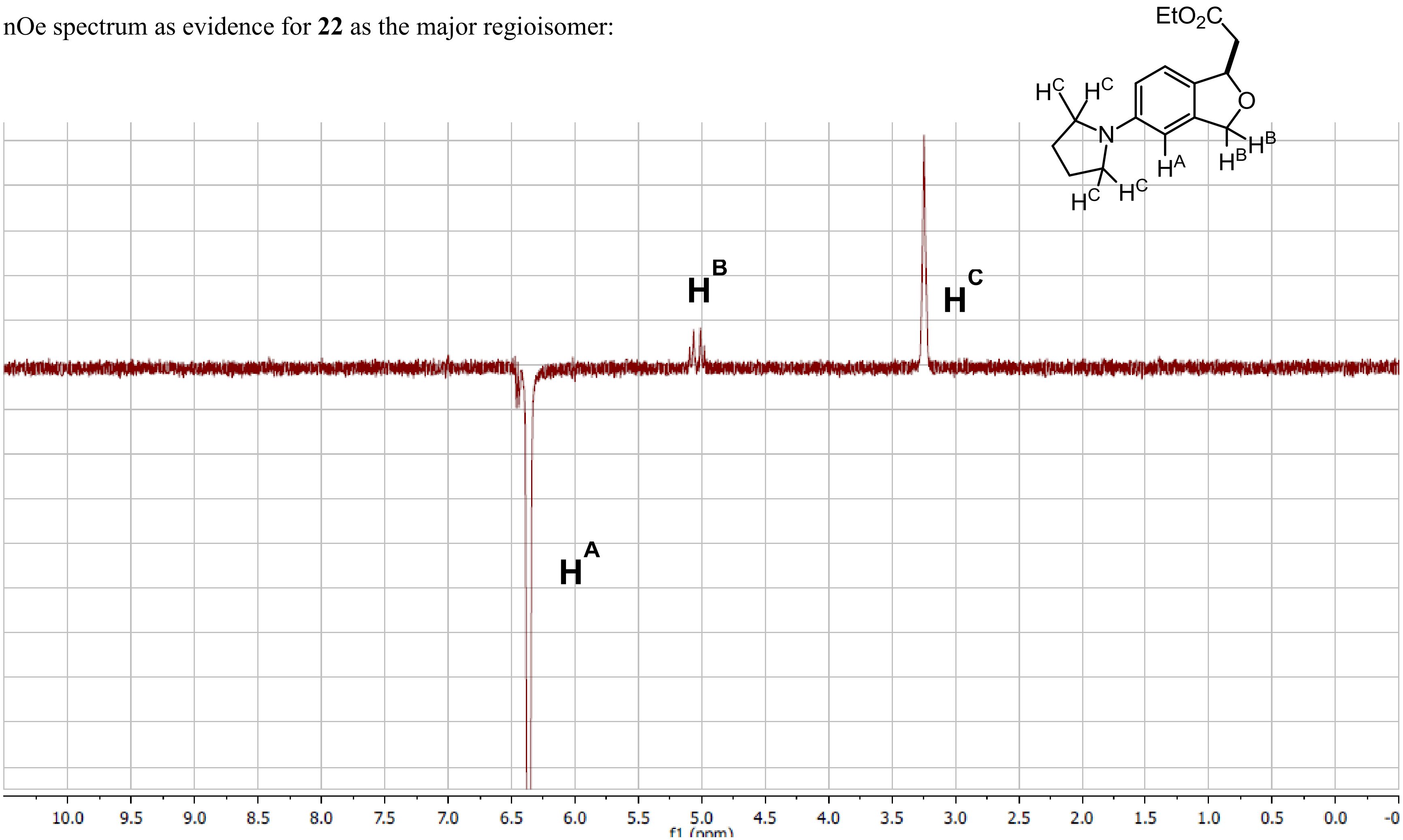

(*R*)-ethyl 2-(2,5-dihydrofuran-2-yl)acetate (23).

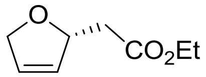

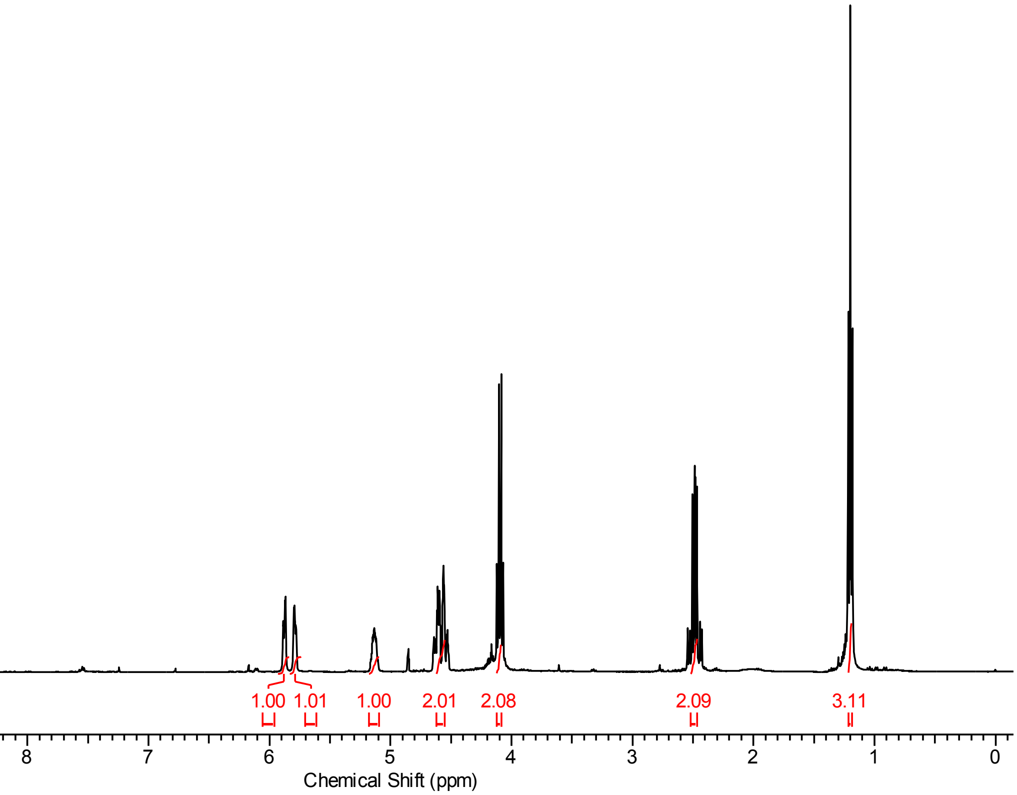

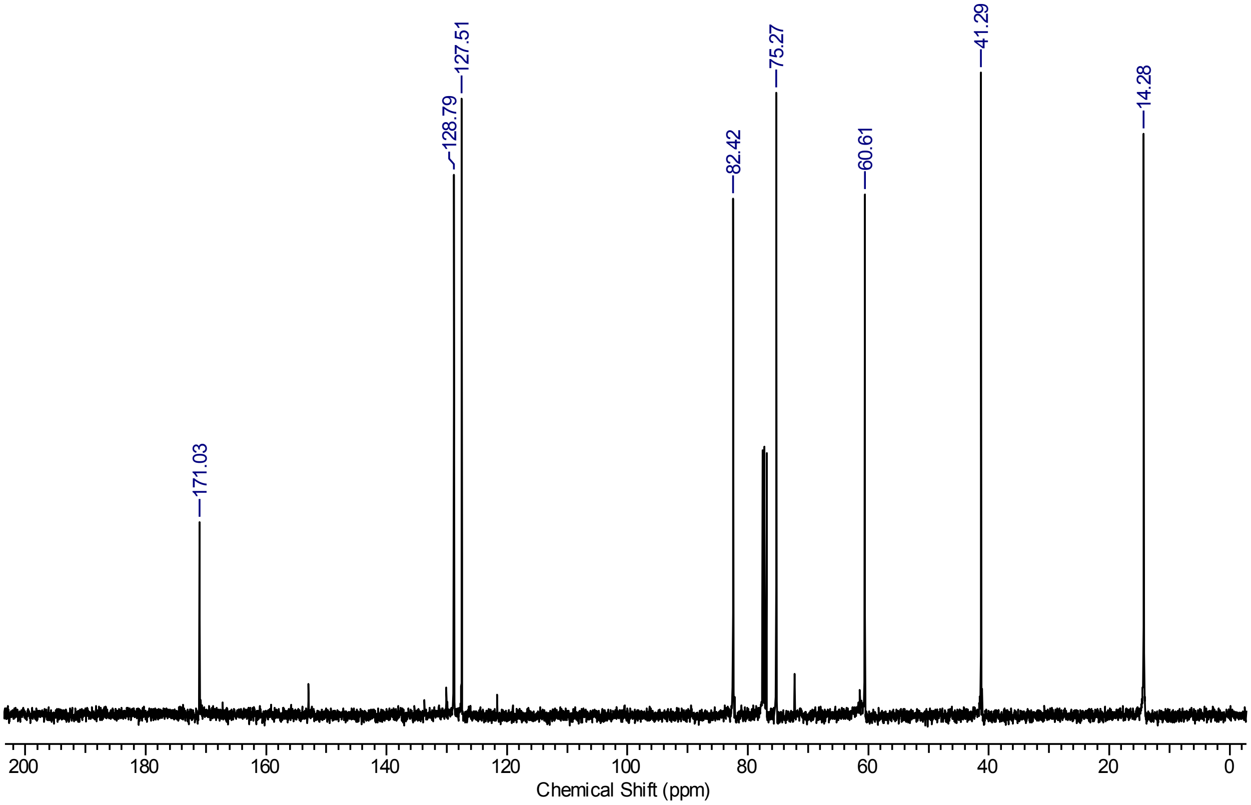

(*R*)-ethyl 2-(4-phenyl-2,5-dihydrofuran-2-yl)acetate (24).

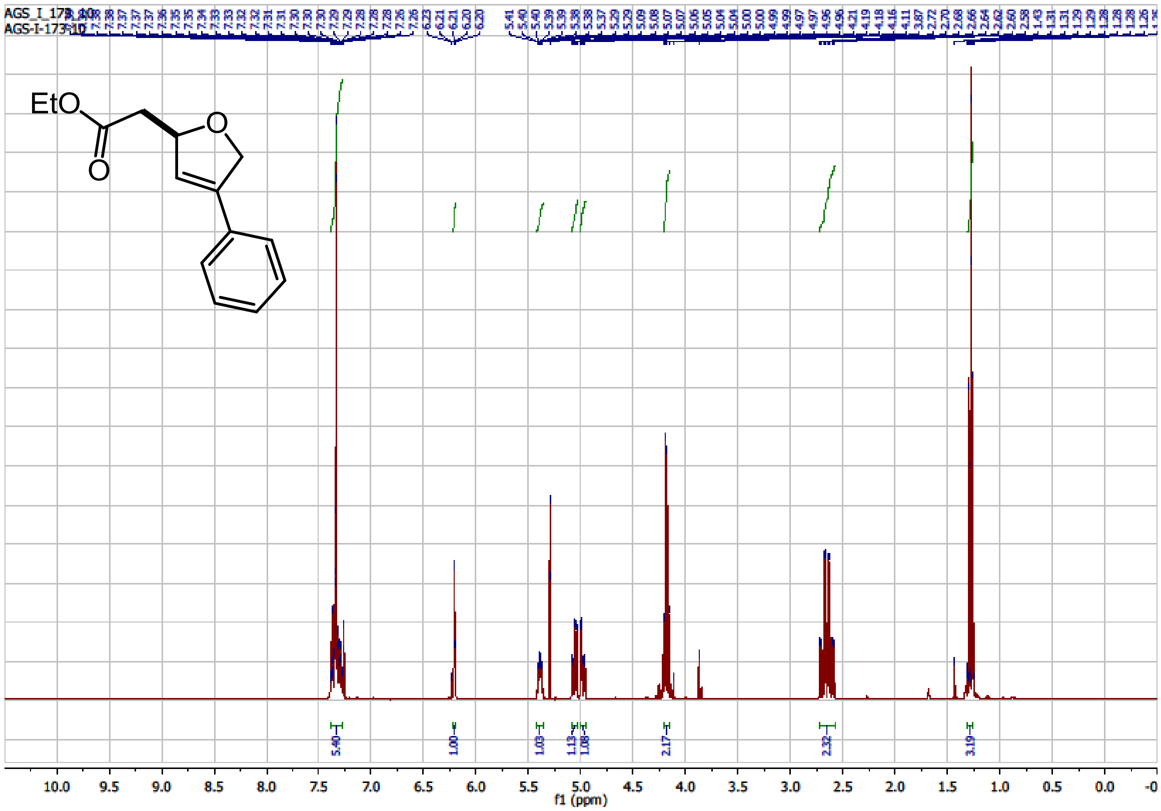

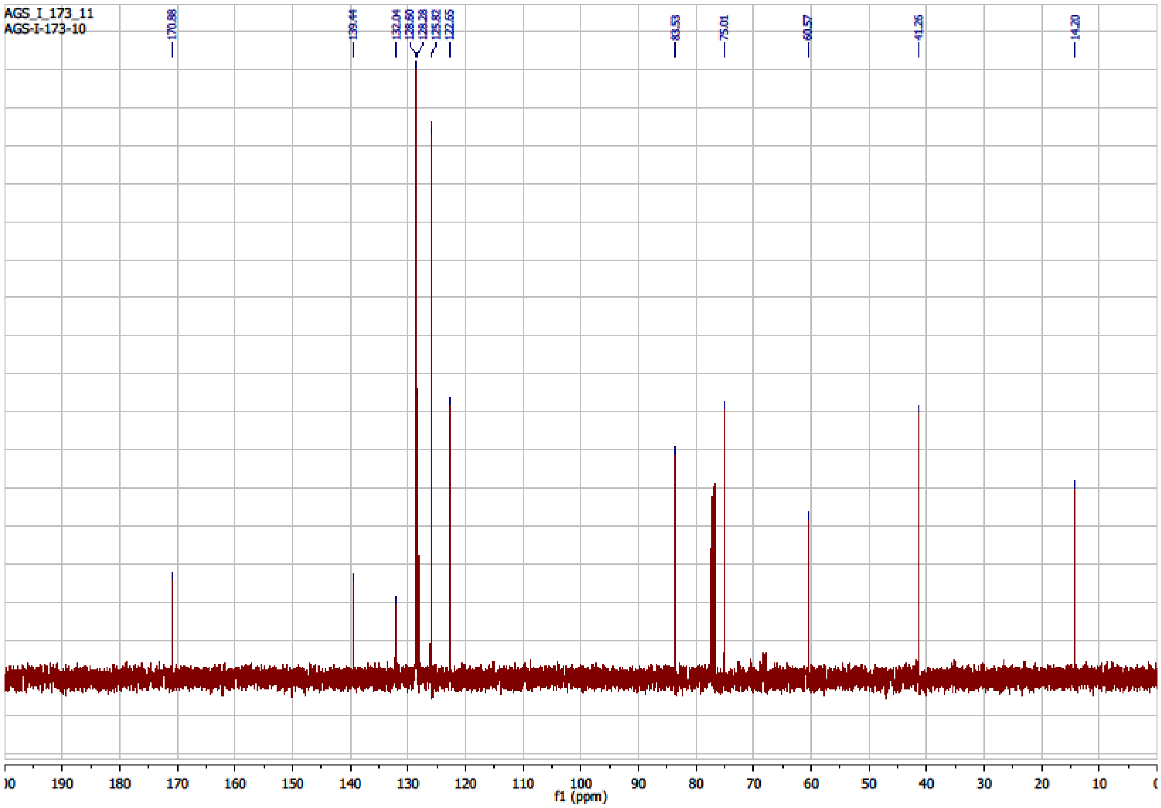

nOe spectra as evidence for **24** as the drawn regioisomer:

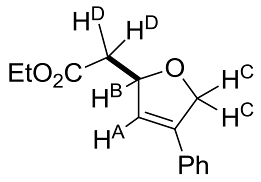

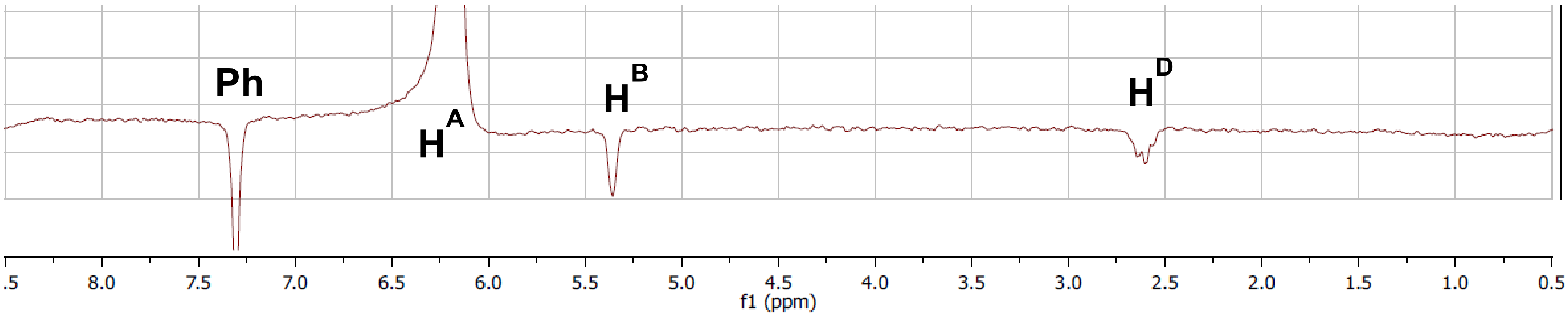

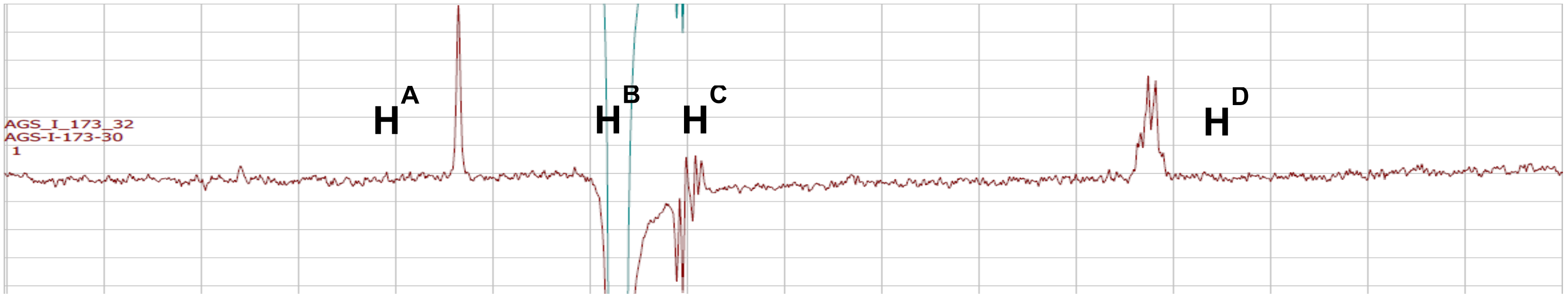

nOe spectra as evidence for **24** as the drawn regioisomer:

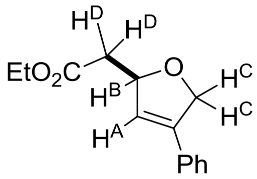

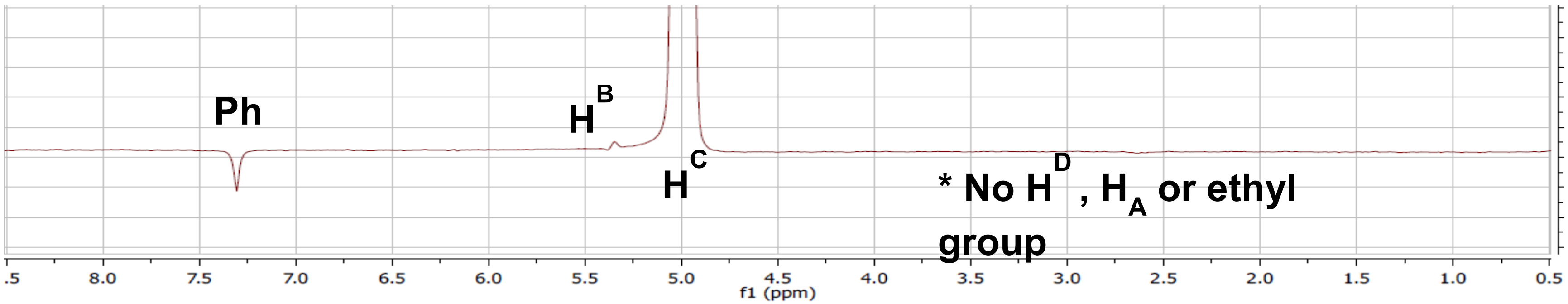

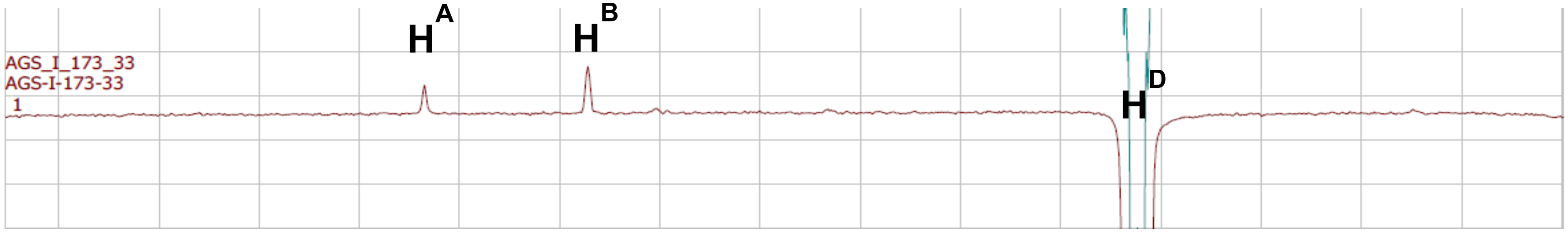

* No H^C or Ph

(*R*)-ethyl 2-(4-benzyl-2,5-dihydrofuran-2-yl)acetate (25).

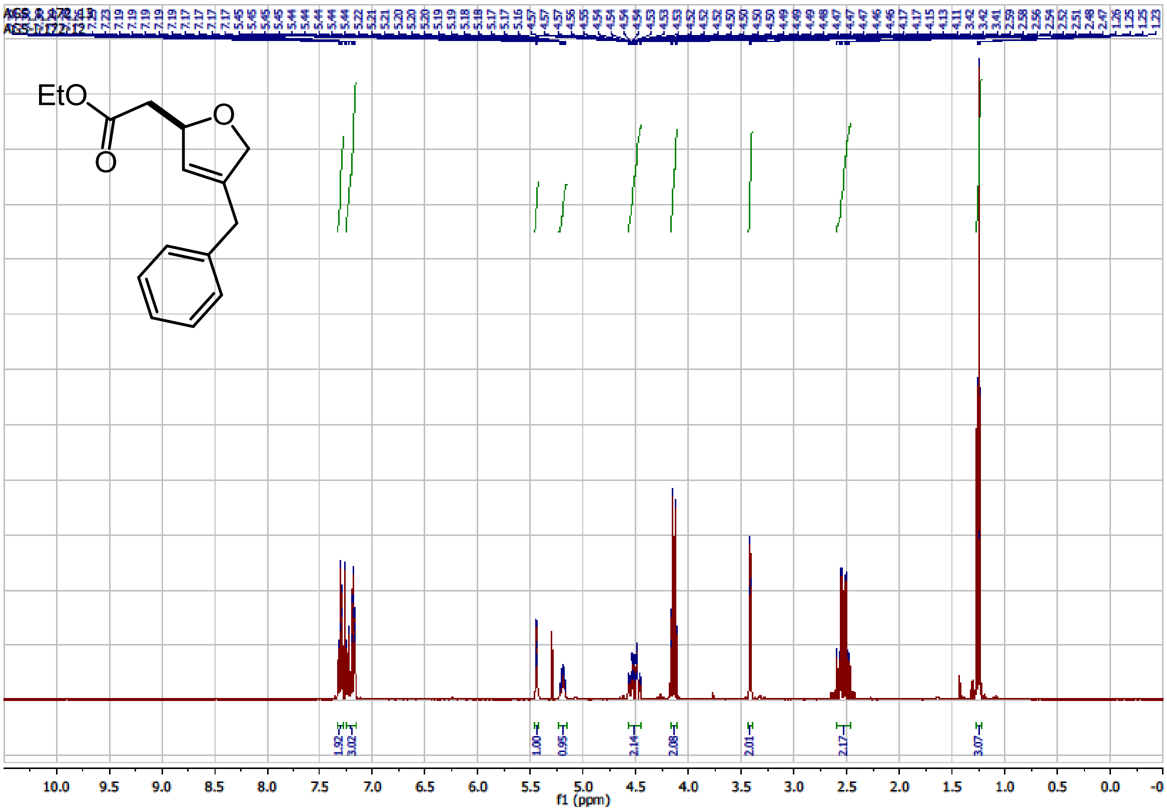

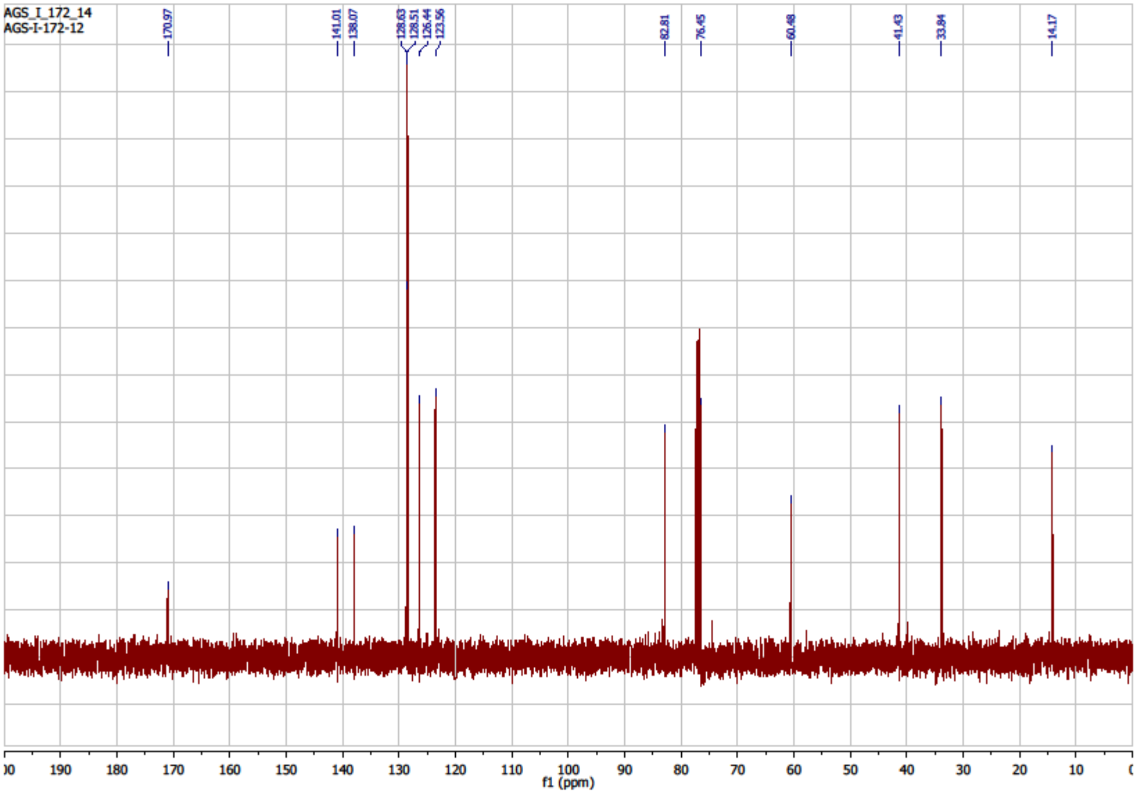

nOe spectra as evidence for drawn reg nOe spectra as evidence for **25** as the drawn regioisomer:

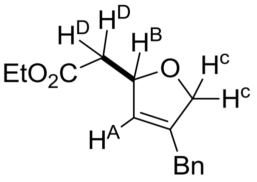

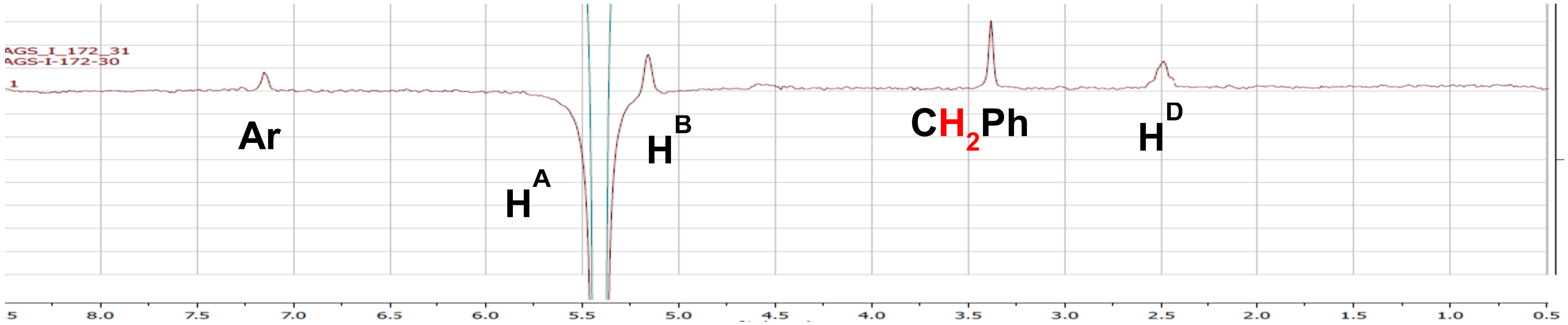

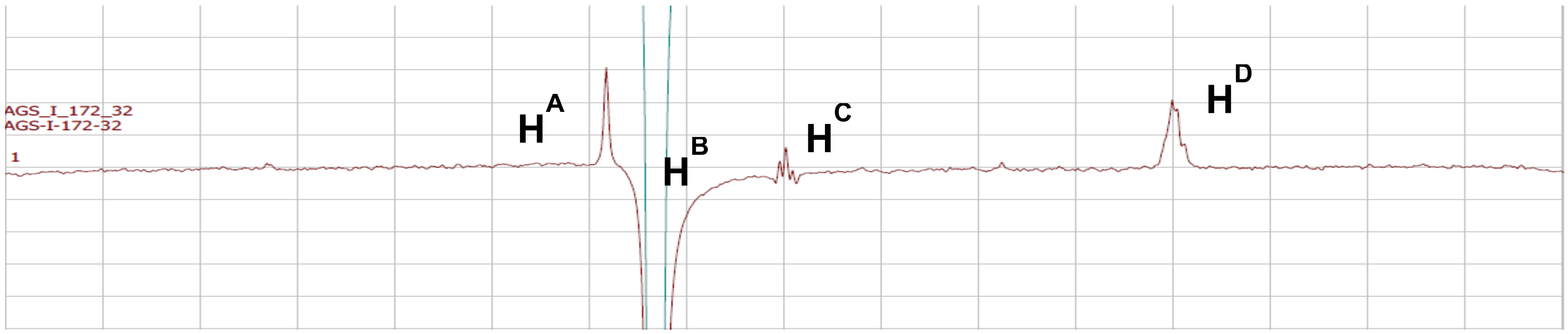

nOe spectra as evidence for drawn reg nOe spectra as evidence for **25** as the drawn regioisomer:

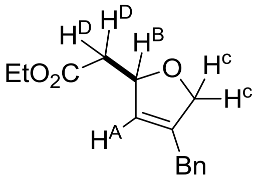

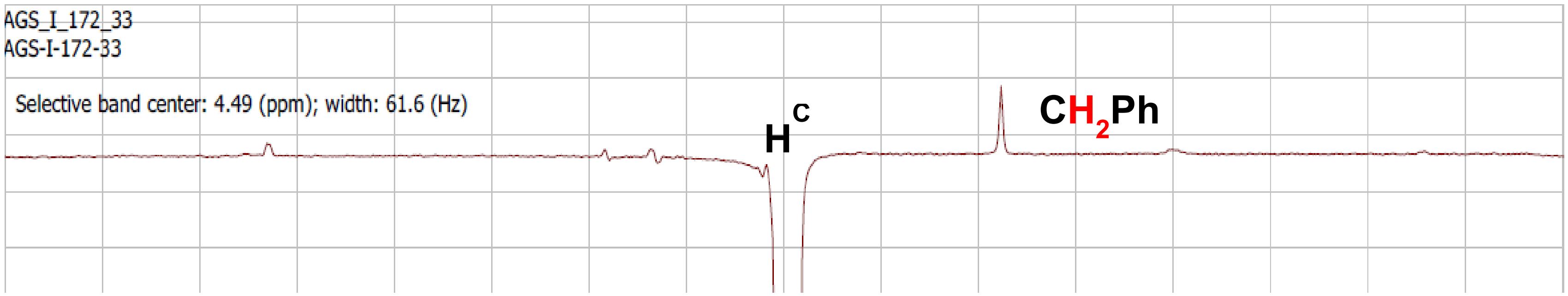

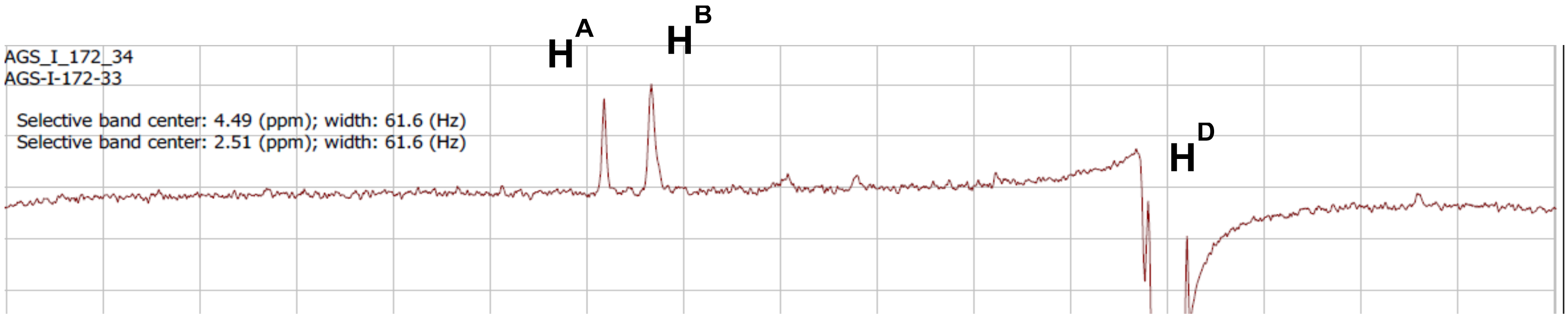

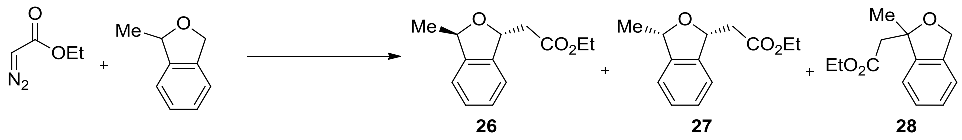

Chiral GC Traces

GC (CHIRASIL DEX, 120 → 140 °C, 1 °C/min) t_r(**28**) = 18.596 min, t_r(**26**) = 21.839 min, t_r(**27**) = 23.556 min

Time [Min]	Height [pA]	Area [pA.Min]	Area [%]
18.596	26.2	309.064	10.09
21.839	217.7	2590.15	84.58
23.558	12.0	163.278	5.33

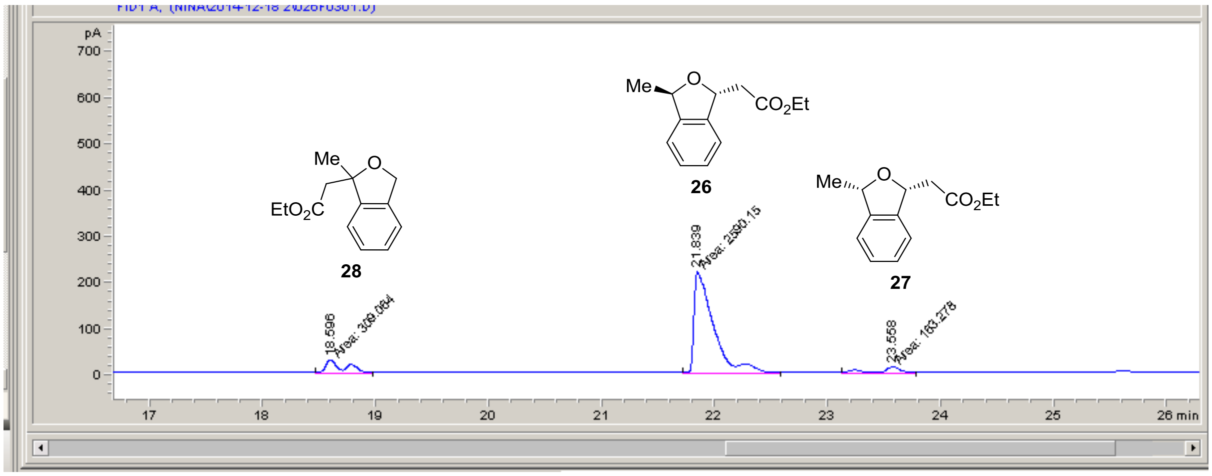

Crude ^{1}H NMR:

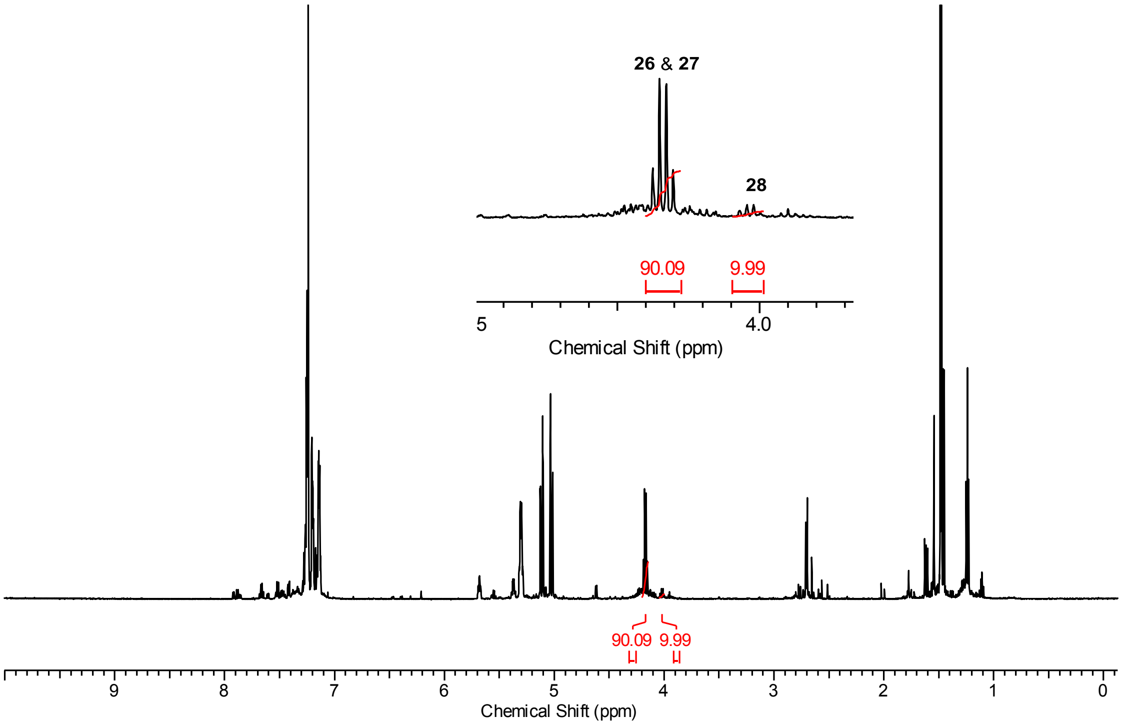

ethyl 2-((1*R*,3*R*)-3-methyl-1,3-dihydroisobenzofuran-1-yl)acetate (26).

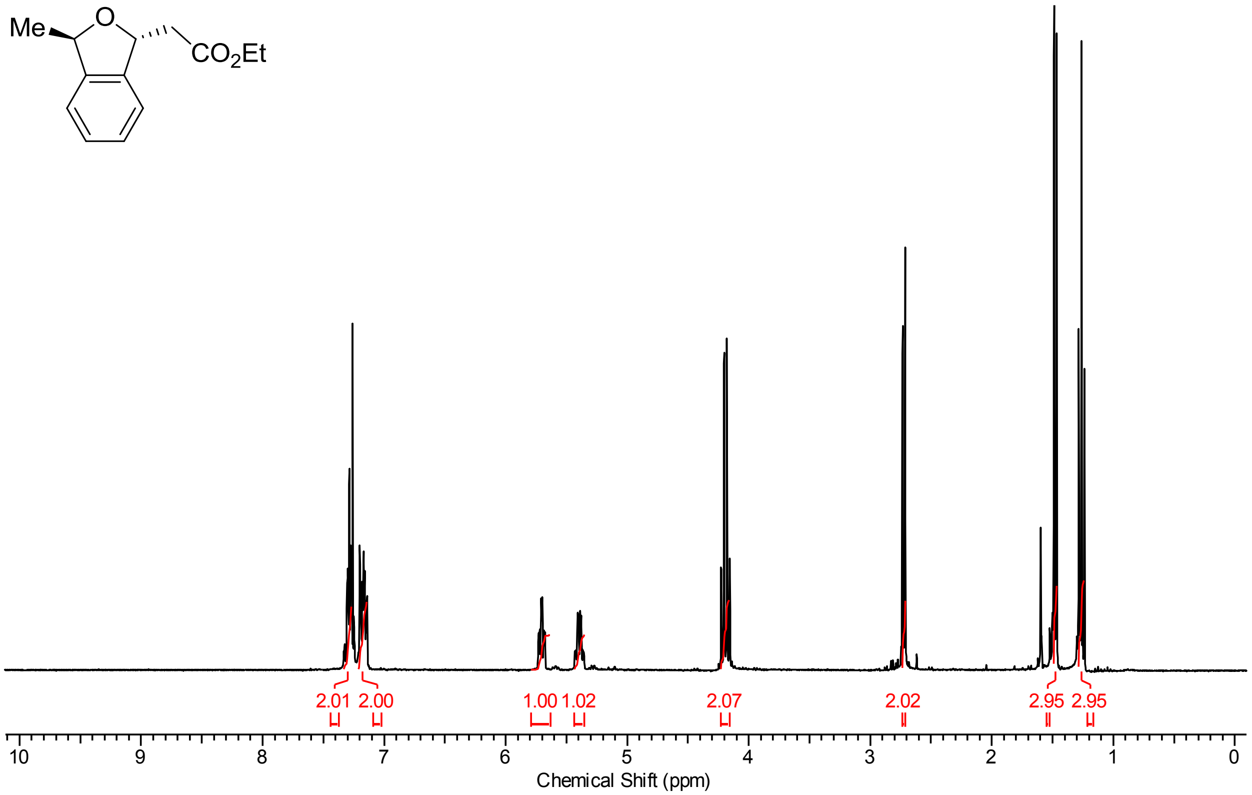

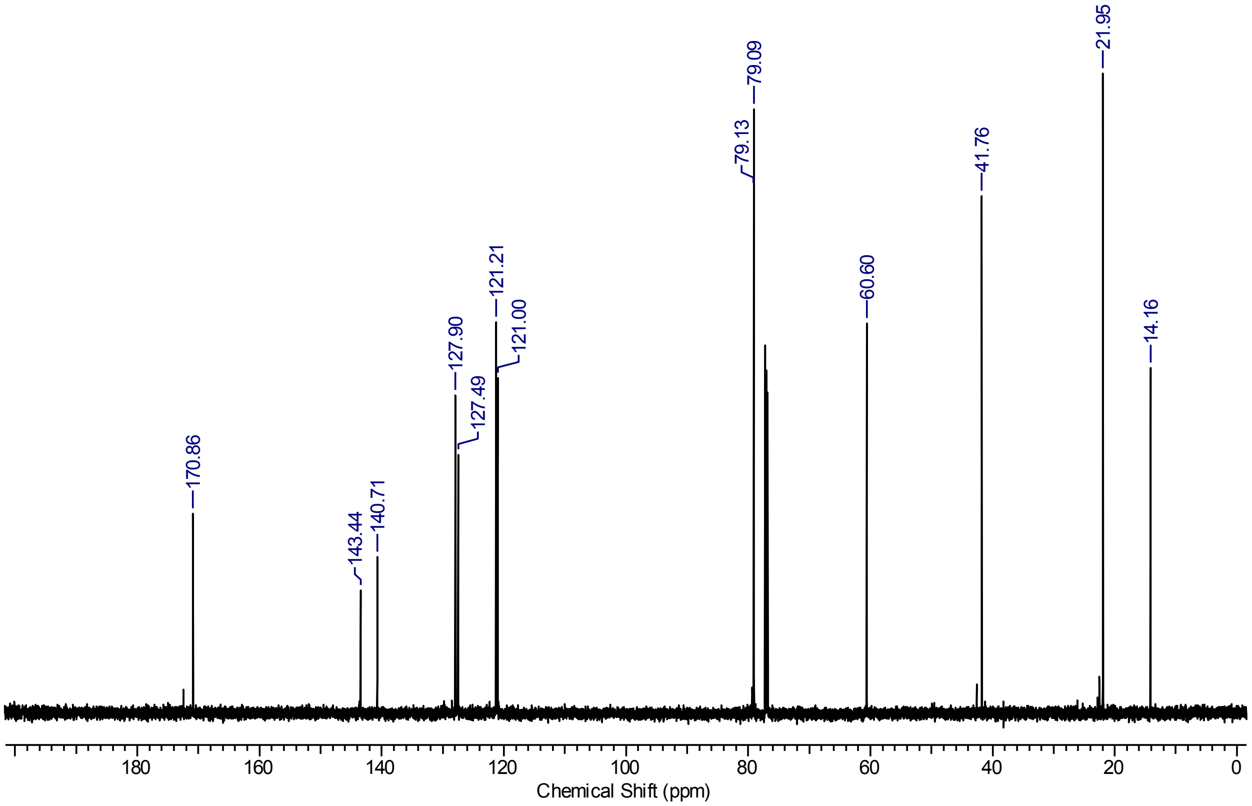

nOe spectra as evidence for **26** as the drawn regioisomer:

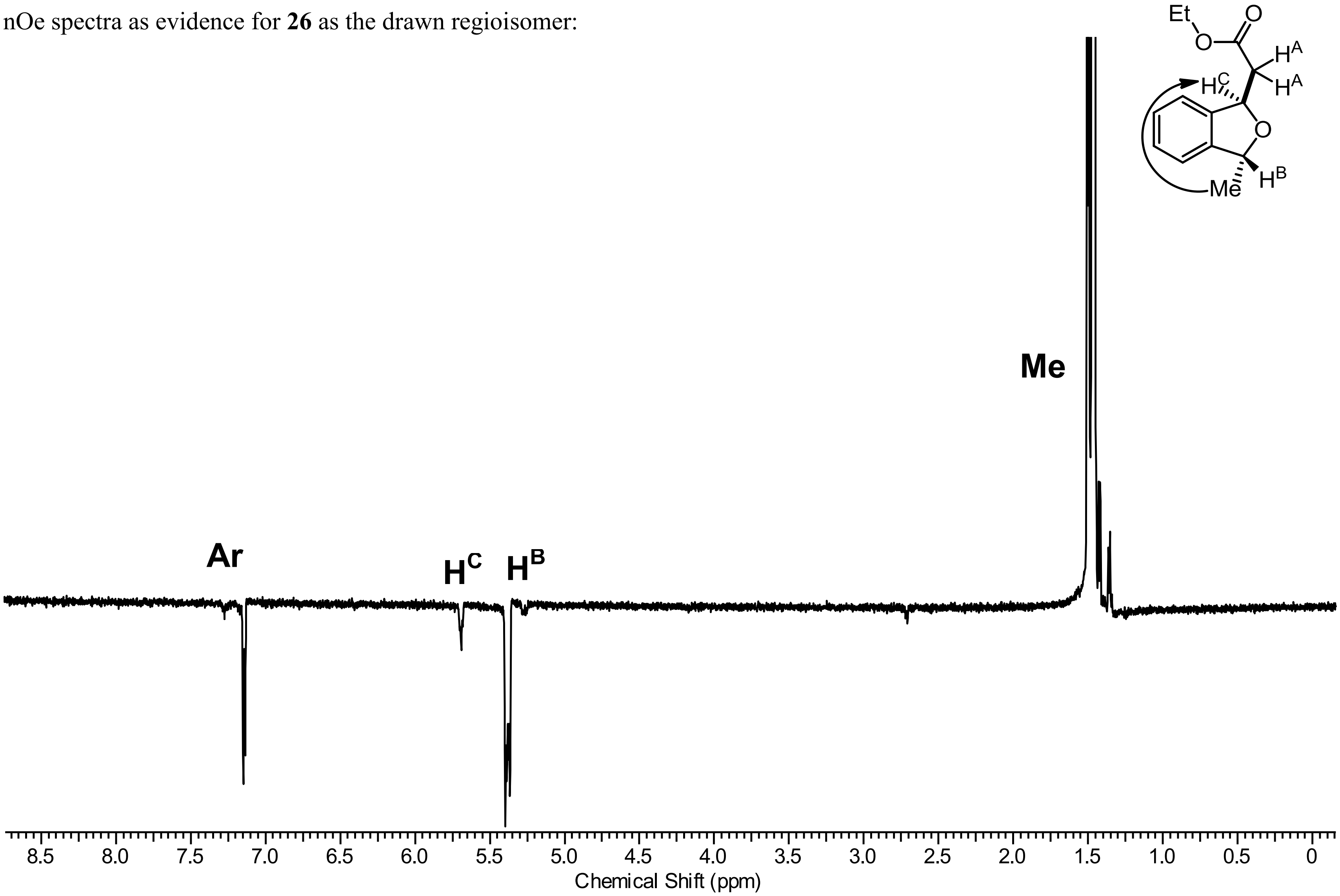

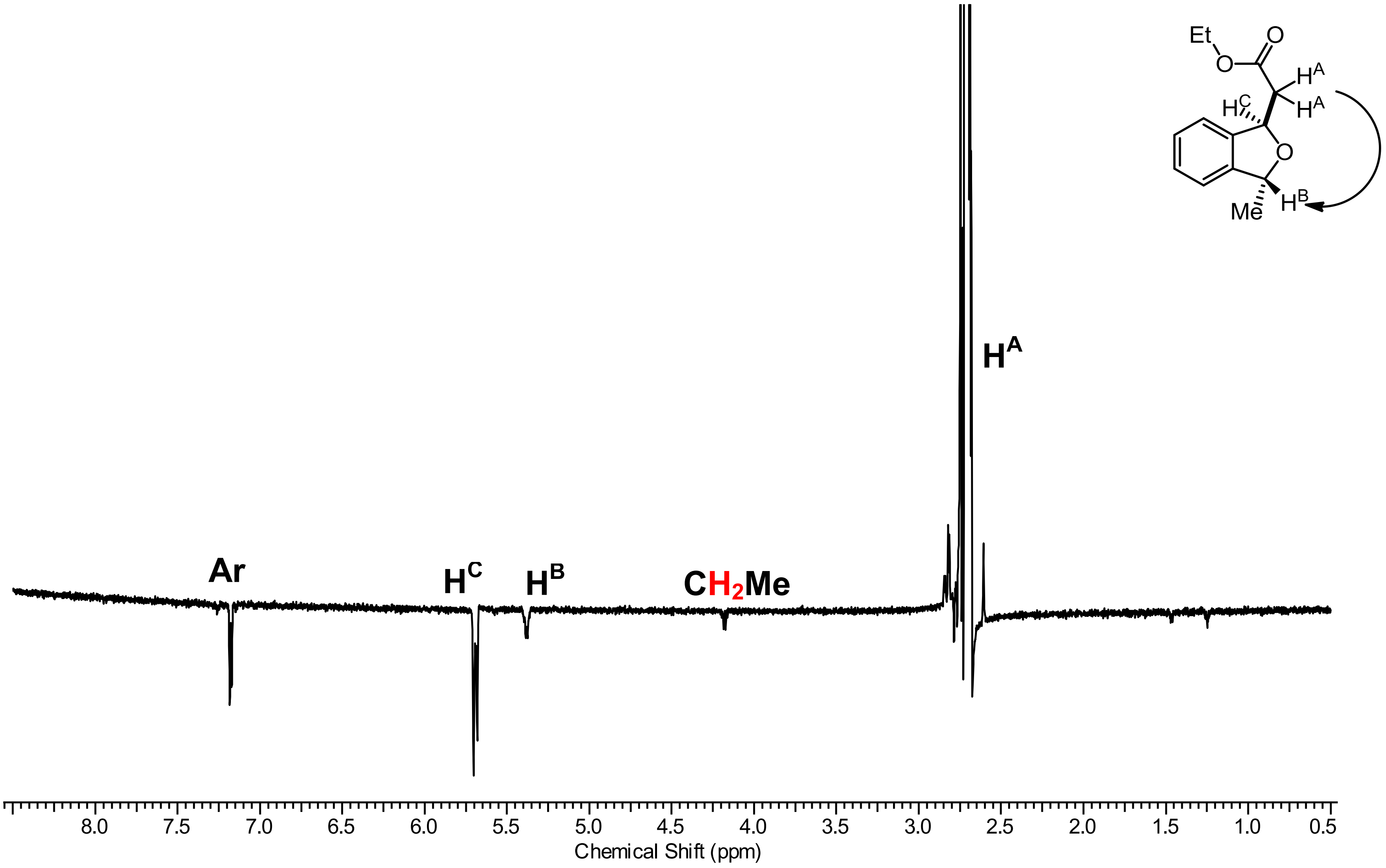

Et
O
O
H^A
H^A
H^C
O
H^B
Me
H^A
Ar
H^C
H^B
CH2Me
8.0
7.5
7.0
6.5
6.0
5.5
5.0
4.5
4.0
3.5
3.0
2.5
2.0
1.5
1.0
0.5
Chemical Shift (ppm)

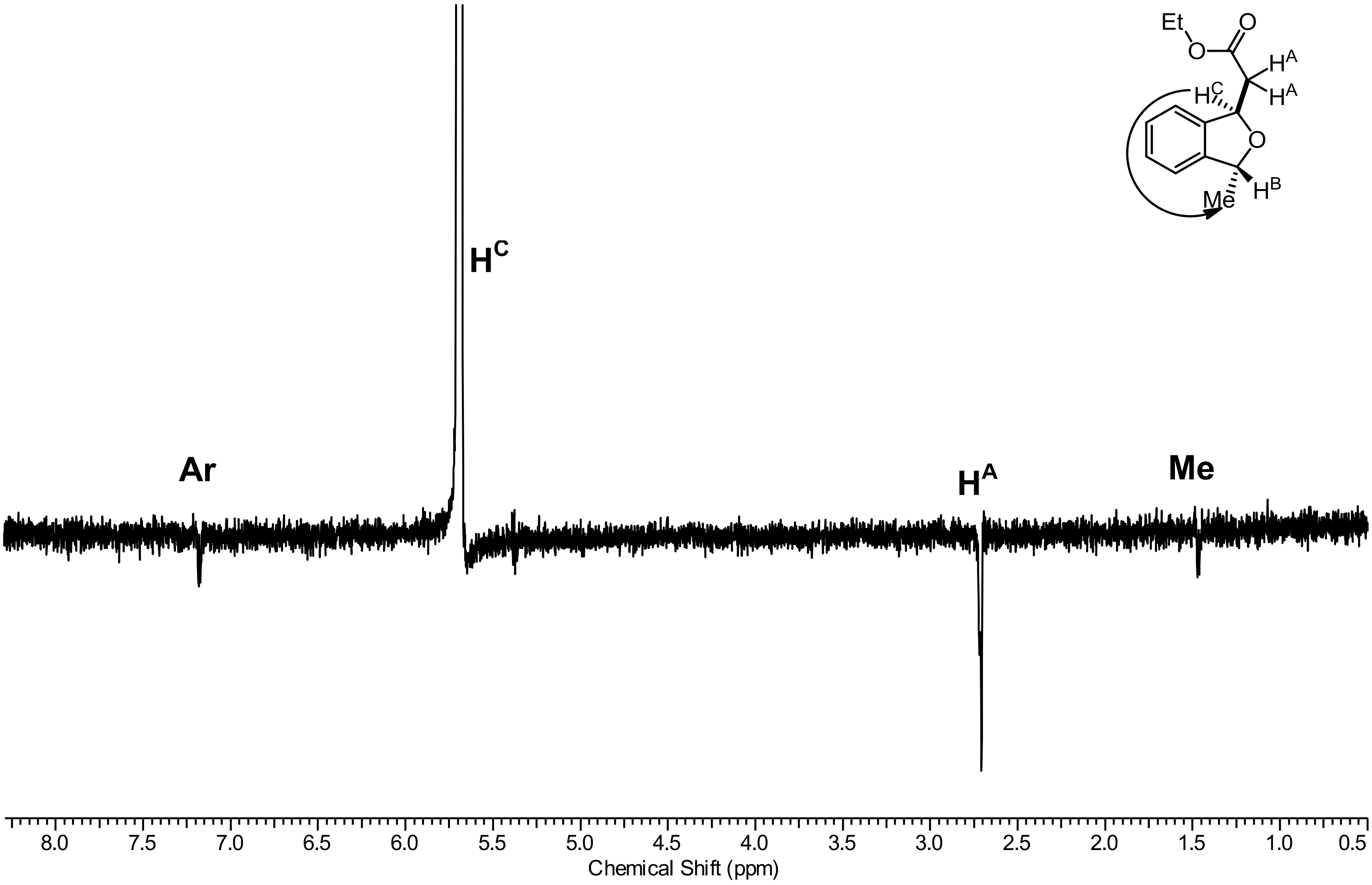

Et
O
O
H^A
H^A
H^C
O
H^B
Me
H^C
Ar
H^A
Me
8.0
7.5
7.0
6.5
6.0
5.5
5.0
4.5
4.0
3.5
3.0
2.5
2.0
1.5
1.0
0.5
Chemical Shift (ppm)

(*S*)-2-(tetrahydrofuran-2-yl)ethyl 3,5-dinitrobenzoate (S56).

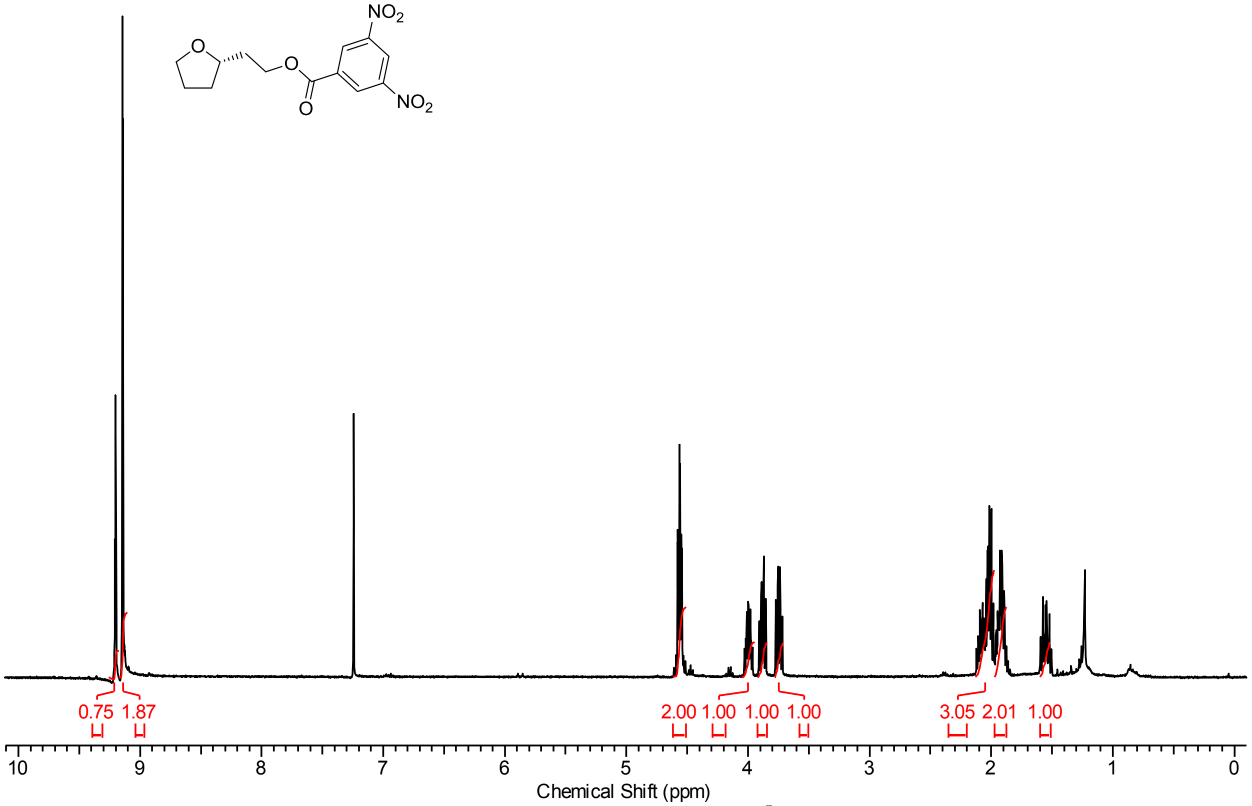

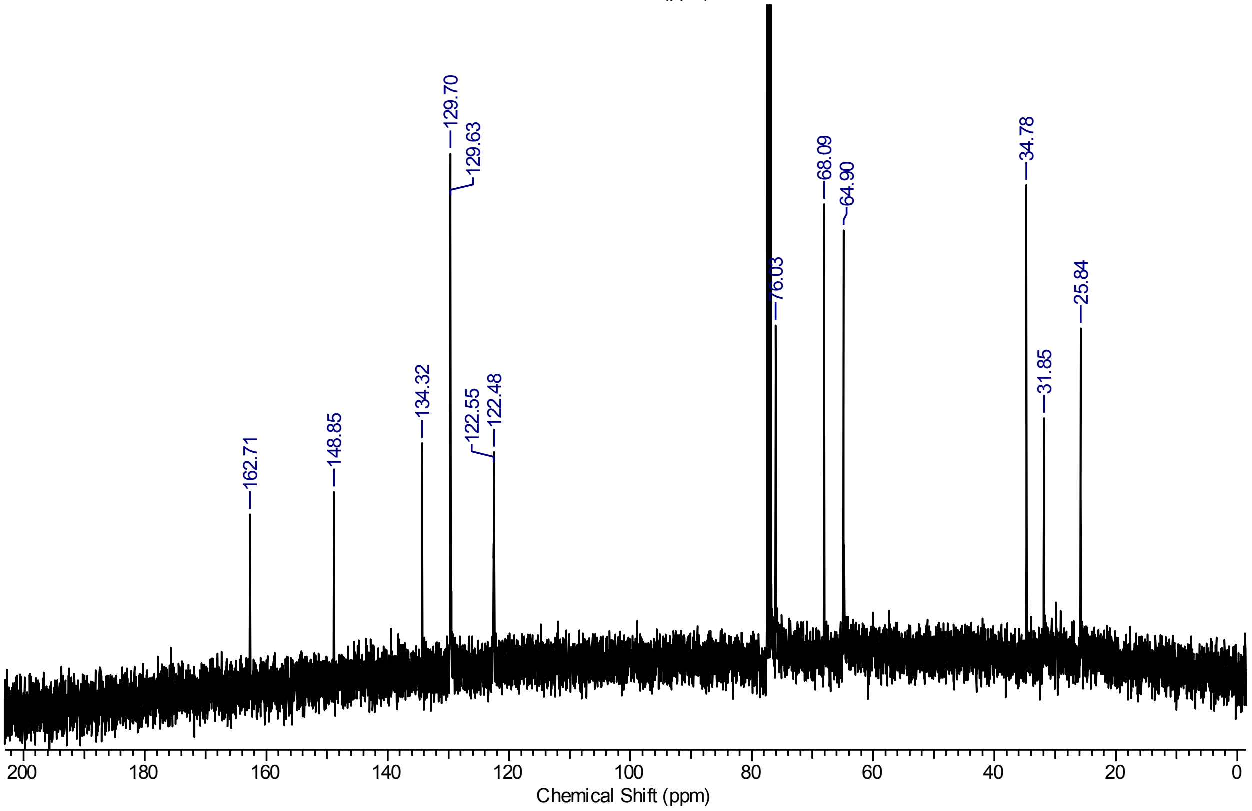

Supplement: Supplementary file 1 [file SC-007-C6SC00190D-s001.pdf]
